# Supplementary material for: Straightforward synthesis of functionalized γ-Lactams using impure CO2 stream as the carbon source
Source: Nat Commun. 2023 Nov 22;14:7604. doi: 10.1038/s41467-023-43289-w (PMC10663487; doi:10.1038/s41467-023-43289-w)
Supplement: Supplementary file 1 — Supplementary Information [file 41467_2023_43289_MOESM1_ESM.pdf]

Supplementary Information

Straightforward Synthesis of Functionalized γ-Lactams using Impure CO_2 Stream as the Carbon Source

Yuman Qin[1], Robin Cauwenbergh[1], Suman Pradhan[1,2], Rakesh Maiti[1,2], Philippe Franck[1], & Shoubhik Das[1,2]*

[1]Department of Chemistry, Universiteit Antwerpen, 2020 Antwerpen, Belgium.
[2]Department of Chemistry, University of Bayreuth, 95447 Bayreuth, Germany.

*Correspondence and Lead Contact: Shoubhik.Das@uni-bayreuth.de

Content

1. Materials and Methods S2
1.1 Materials and general procedures S2
1.2 Analytical methods S2
2. Starting Material Synthesis S3-S5
3. Experimental Procedures S5-S13
3.1 Setup for the photoredox synthesis of γ-lactam S5-S6
3.2 Optimization of conditions for reaction under visible-light irradiation S6-S7
3.3 Standard procedure for the synthesis of γ-lactam S8
3.4 Gram-scale reaction S8
3.5 Quenching experiments S8
3.6 Stern-Volmer fluorescence quenching experiments S9
3.7 Intercepting the α-amino radical S9
3.8 Intercepting the carbanion intermediate with D_2O S9-S10
3.9 Intercepting the carbanion intermediate with aldehyde S10-S11
3.10 Defluorinative alkylation of trifluoromethylakene S11
3.11 $^{13}CO_2$ labelling experiment S11-S12
3.12 Light on-off experiments S12
3.13 Time course experiments S13
4. Characterization of Starting Materials and Products S14-S36
5. ^{1}H NMR and ^{13}C NMR Spectra S37-S143

1. Materials and Methods

1.1 Materials and general procedures

Commercial reagents were used without purification and reactions were run under CO_2 atmosphere with exclusion of moisture from reagents using standard techniques for manipulating air-sensitive compounds. $CDCl_3$ was purchased from Chemfinder.com. DMF was purchased from Acros Organics-International. $[Ir(ppy)_2(dtbbpy)]PF_6$ was purchased from Sigma Aldrich. $KHCO_3$ was purchased from Acros Organics – International.

1.2 Analytical methods

Kessil lamps were purchased from Laser 2000 (UK) Ltd, with precise wavelengths (456 nm). ^1H NMR spectra (400 MHz) and ^{13}C NMR spectra (100 MHz) were recorded using Bruker Avance 400 spectrometer with $CDCl_3$ or DMSO-d_6 as solvent. NMR spectra were calibrated using the solvent residual signals ($CDCl_3$: δ ^1H = 7.26, δ ^{13}C = 77.00; DMSO-d_6: δ ^1H = 2.50, δ ^{13}C = 39.52). The following abbreviations were used to describe peak splitting patterns when appropriate: s = singlet, d = doublet, t = triplet, q = quartet, quint = quintet, dd = doublet of doublet, m = multiplet and br s = broad singlet. Analytical thin layer chromatography (TLC) was performed using pre-coated TLC sheets ALUGRAM® SIL G/UV254 (Machery-Nagel) and spots were visualized using UV light (254 nm). Flash chromatography was performed on an automated chromatography system (Biotage® or Combiflash®Rf) with on-line UV detection using commercial Silica Flash Cartridges and the indicated solvent and gradient system. High performance liquid chromatography (HPLC) of samples were prepared by dissolving 0.1−5.0 mg of the product in methanol or in acetonitrile and further diluting to a concentration of 10^{-5}−10^{-6} M with 50% methanol (or acetonitrile)/50% H_2O/0.1% formic acid.

2. Starting Material Synthesis

GP1 – General Procedure for Synthesis of N-alkylanilines

The synthesis of N-alkylanilines used in this work were prepared according to the methods shown below.

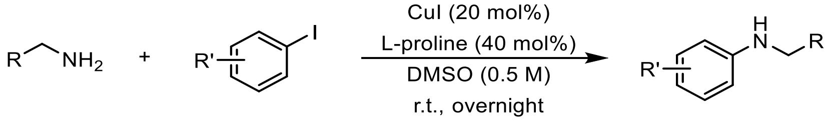

An oven-dried Schlenk flask equipped with a stirring bar was charged with CuI (1 mmol, 0.20 equiv.) and L-proline (2 mmol, 0.40 equiv.). The flask was then evacuated and backfilled with N_2 for 3 times. The DMSO (10 mL, 0.5M) and PhI (5 mmol, 1.0 equiv.) were sequentially added. The resulting solution was stirred for 5 min and the color turned out to blue. Then the amine (7.5 mmol, 1.5 equiv.) was added to the solution. The resulting mixture was stirred under N_2 at room temperature overnight. The reaction was quenched and diluted by water (20 mL) and EtOAc (20 mL). The layers were separated, and the aqueous layer was extracted with EtOAc (3 × 20mL). The combined organic layers were dried (Na_2SO_4), filtered and concentrated. The crude was purified by column chromatography.

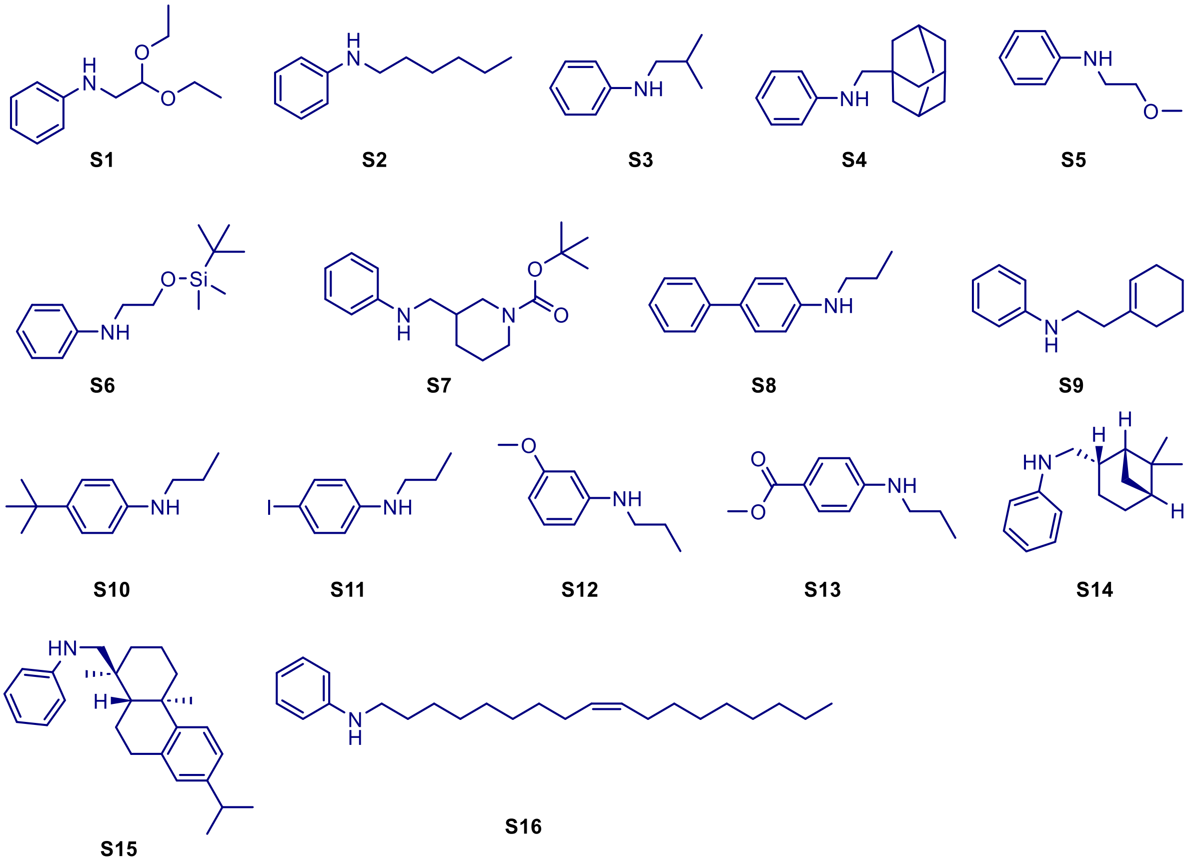

Supplementary Figure 1. The List of N-alkylanilines

GP2 – General Procedure for Synthesis of complex N-alkylanilines

The synthesis of complex N-alkylanilines used in this work were prepared according to the methods shown below.

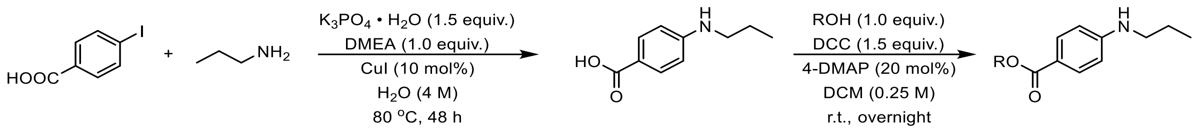

1 **2** **3** **4**

A 50 mL Schlenk flask equipped with a stirring bar was charged with *p*-Iodobenzoicacid (10 mmol, 1.0 equiv.), $K_3PO_4{\cdot}H_2O$ (15mmol, 1.5 equiv.), and CuI (1.0 mmol, 10 mol%). The flask was then evacuated and backfilled with N_2 for 3 times. Subsequently, *n*-Propyl amine (15mol, 1.5 equiv.), DMEA (10 mol, 1.0 equiv.), and water (2.5 mL, 4M) were added under N_2. The flask was transferred to oil bath (80℃) to react for 48h. After the reaction finished, the resulting mixture was cooled to room temperature and quenched by ice water. The concentrated HCl was dropwise added to the solution until the pH up to 5. Extracted the solution with ethyl acetate (50 mL × 4) and collected the organic layer. The combined organic phase was dried with $MgSO_4$ and concentrated under reduced pressure. The final residue (**3**) was obtained in 80 % yield and can be used to next step without purification.

A 50 mL Schlenk flask equipped with a stir bar was charged with **3** (7.5 mol, 1.5 equiv.), nature product alcohol (5 mol, 1.0 equiv.), DCC (7.5 mol, 1.5 equiv.), 4-DMAP (1.0 mol, 20 mol%) and DCM (20 mL, 0.25 M). The reaction was stirred for overnight at room temperature. The flask was opened, filtered the reaction mixture and washed the residue with DCM. The solution was collected and concentrated under reduced pressure. The product **4** was purified by flash chromatography and got in 70 % yield.

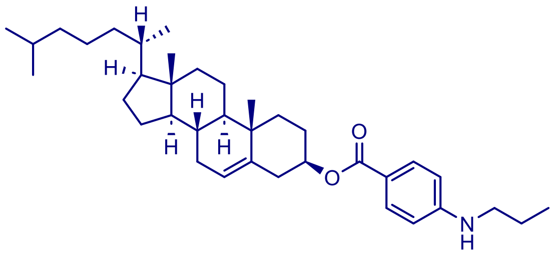

S17

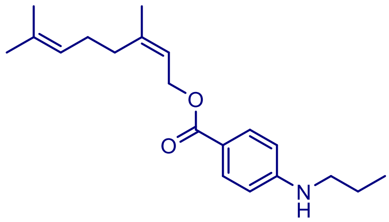

S18

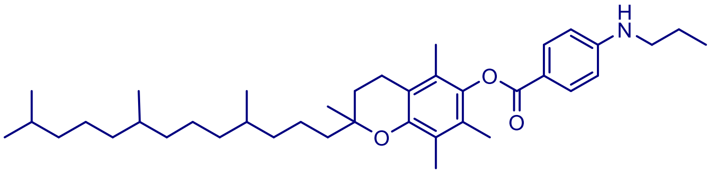

S19

Supplementary Figure 2. The list of complex N-alkylanilines

GP3 – General procedure for synthesis of alkenes

The synthesis of alkenes used in this work were prepared according to the methods shown below.

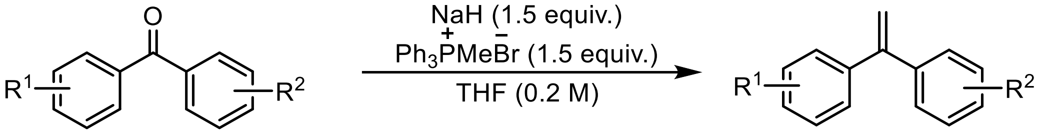

A 50 mL Schlenk flask equipped with a stirring bar was charged with methyltriphenylphosphonium bromide (1.5 equiv.) in THF, NaH (1.5 equiv.) was added under argon at 0 ºC and stirred for 30 min. Then the ketone in THF was added drop wise and stirred overnight. The reaction was quenched by water in ice bath. Extracted the mixture with ethyl acetate (50mL x 3) and combined the organic phase. The organic phase dried over anhydrous Na_2SO_4 and concentrated in vacuo. The residue was purified by silica gel column chromatography to afford pure styrene (85-99% yield).

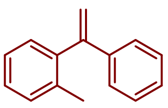

S20

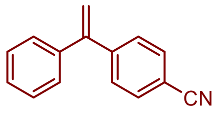

S21

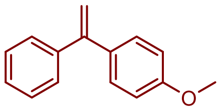

S22

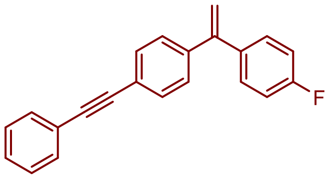

S23

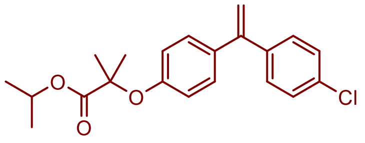

S24

Supplementary Figure 3. The list of alkenes

GP4 – General procedure for synthesis of complex alkenes

The synthesis of complex alkenes used in this work were prepared according to the methods shown below.

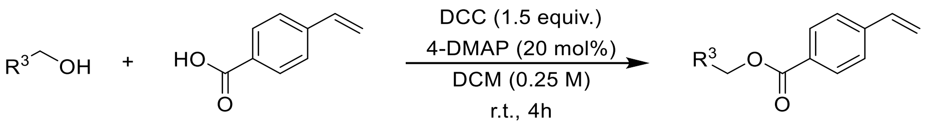

A 50 mL Schlenk flask equipped with a stir bar was charged with 4-vinylbenzoic acid (7.5 mol, 1.5 equiv.), nature product alcohol (5 mol, 1.0 equiv.), DCC (7.5 mol, 1.5 equiv.), 4-DMAP (1.0 mol, 20 mol%) and DCM (20 mL, 0.25 M). The reaction was stirred for 4h at room temperature. The flask was opened, filtered the reaction mixture and washed the residue with DCM. The solution was collected and concentrated under reduced pressure. The final product was purified by flash chromatography and got in 85 % yield.

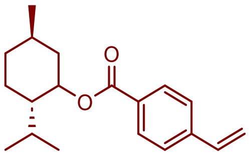

S25

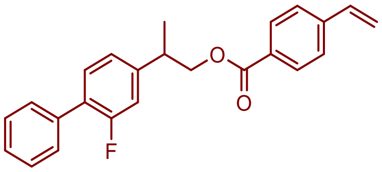

S26

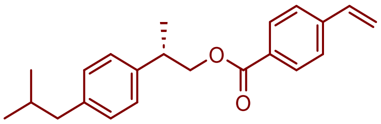

S27

Supplementary Figure 4. The list of complex alkenes

3. Experimental Procedures

3.1 Setup for the photoredox synthesis of γ-lactam

The reaction setup is depicted in **Supplementary Figure 5**. The reaction setup consists of a 456 nm Kessil lamp, cooling of the setup was performed by two commercially available 120 mm computer fans to keep the temperature around 27 ℃. The light intensity was measured by Ophir StarLite power meter with 3A probe head. The light intensity of Kessil lamp (λ = 456 nm) was 0.35 W/cm^2 and the distance between tube and light is 3 cm. The CO_2 was provided by a balloon filled with pure CO_2 gas and the distance between lamp and reaction tube was 2~3 cm.

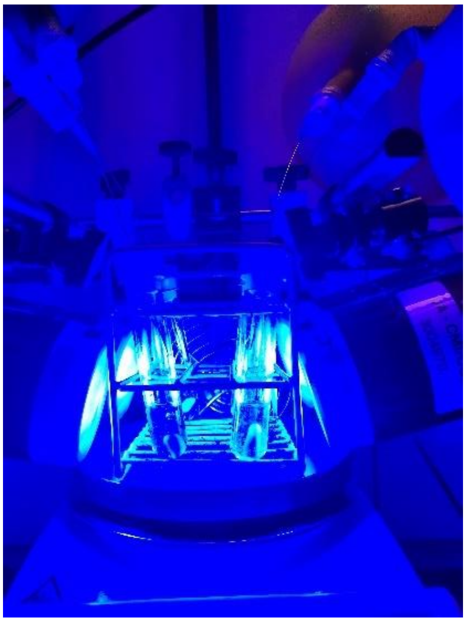

Supplementary Figure 5. Set up for the photoredox synthesis of γ-lactam

3.2 Optimization of conditions for reaction under visible-light irradiation

In a 10 mL vial equipped with a rubber plunger, 1,1-Diphenylethylene (0.2 mmol), N-Ethylaniline (0.24 mmol), $[Ir(dtbbpy)(ppy)_2]PF_6$ (0.2 mol%) and base (n mol%) were dissolved in solvent (2.0 mL). The mixture was bubbled with CO_2 (10 min) and irradiated with a 40 W Kessil lamp (λ= 456 nm, full intensity) for 20h. After irradiation, 1.0 mL of HCl solution (0.1 M) was added to the reaction. The resulting solution was extracted with EtOAc (5 × 3 mL). Yields were calculated by crude ^{1}H NMR with 1,3,5-trimethoxybenzene as the internal standard.

Supplementary Table 1. Screening of different base

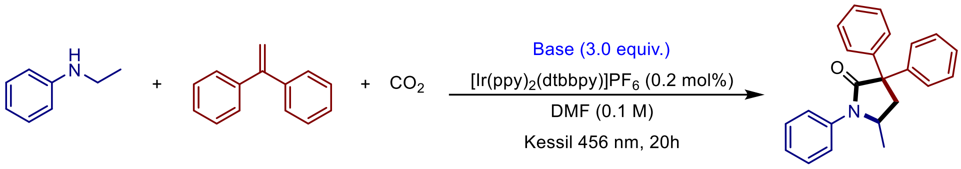

Entry	Base (3.0 equiv.)	Yield (%)
1	K_2CO_3	73
2	Na_2CO_3	68
3	Li_2CO_3	76
4	Cs_2CO_3	56
5	KOAc	30
6	K_3PO_4	76
7	^{t}BuOk	55
8	$KHCO_3$	81
9	DABCO	0
10	Et_3N	0
11	quinuclidine	0

Supplementary Table 2. Screening of different solvent

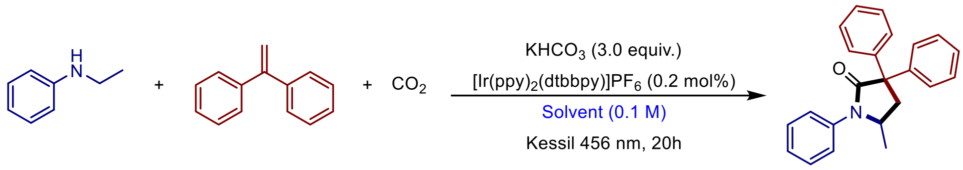

Entry	Solvent (0.1 M)	Yield (%)
1	DMA	78
2	DMSO	70
3	MeCN	0
4	DCE	0
5	1,4-Dioxane	0
6	EtOH	0
7	Toluene	0
8	THF	0

Supplementary Table 3. Screening of different $KHCO_3$ loading

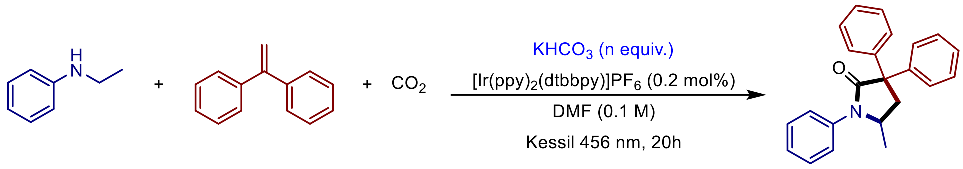

Entry	$KHCO_3$ (n equiv.)	Yield (%)
1	2.0	80
2	1.5	81
3	1.0	80
4	0.2	82

Supplementary Table 4. Screening of reaction temperature

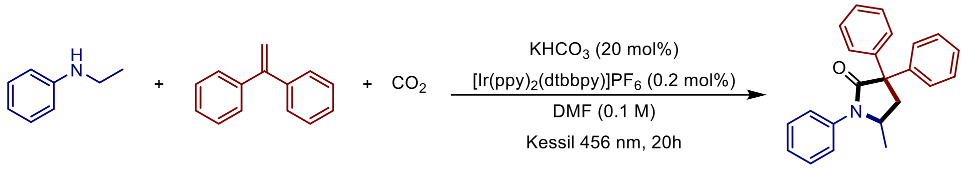

Entry	Temperature (℃)	Yield (%)
1	rt	98
2	50	70

3.3 Standard procedure for the synthesis of γ-lactam

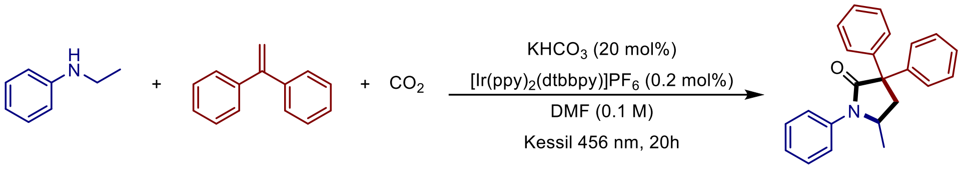

A dry 10 mL vial equipped with a stirring bar was charged with the 1,1-Diphenylethylene (0.2 mmol), N-Ethylaniline (0.24 mmol), $[Ir(ppy)_2(dtbbpy)]PF_6$ (0. 2 mol%), and $KHCO_3$ (20 mol%), then the solvent DMF (0.1 M) was added. The tube was capped with a rubber plunger, then the mixture was bubbled with CO_2 balloon (10 min). After the reaction mixture was thoroughly bubbled, the vial was placed approximately 3 cm from Kessil 456 nm lights, and the mixture was stirred under irradiation with fan for 20 hours. After that, and the mixture was diluted with HCl solution (0.1 M, 2.0 mL) and EtOAc (2.0 mL). 1,3,5-Trimethoxybenzene was added, and the layers were separated. The aqueous layer was extracted with EtOAc (2.0 mL x 3) and the combined organic layers were evaporated under reduced pressure (40 ºC water bath). The yield was analyzed by ^{1}H NMR spectroscopy.

3.4 Gram-scale reaction

The oven-dried two necks flask (150 mL) containing a stirring bar was charged with 1,1-Diphenylethylene (1.8 mL, 10 mmol), N-Ethylaniline (1.9 mL, 12 mmol), $[Ir(ppy)]_2(dtbbpy)]PF_6$ (25 mg, 6 μmol, 0.1 mol %), $KHCO_3$ (200mg, 20 mol%) and DMF (50 mL, 0.2 M). The flask was then bubbled with CO_2 for 10 min and equipped with a CO_2 balloon. The reaction was stirred at room temperature and irradiated with two 40 W kessil lights (456 nm, 3 cm away, with cooling fan to keep the reaction temperature at 25~30 °C) for 72h. The resulting mixture was quenched by 20 mL of 0.1N HCl (aq.) and diluted with 30 mL of EtOAc and then stirred for 2 min and extracted by EtOAc four times and the combined organic phases were concentrated in vacuo. The residue was purified by silica gel flash column chromatography to give the pure desired product in 92% yield.

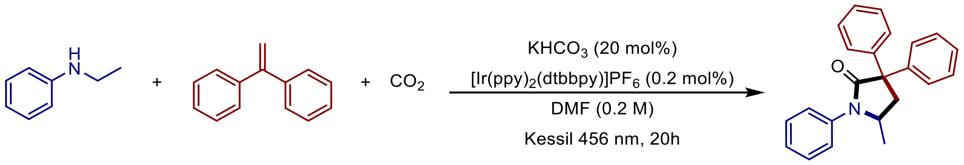

10 mmol **12 mmol** **92 %**

3.5 Quenching experiments

In an oven-dried 10 mL vial containing a stirring bar, 1,1-Diphenylethylene (0.2 mmol), N-Ethylaniline (0.24 mmol), $[Ir(ppy)_2(dtbbpy)]PF_6$ (0. 2 mol%), and $KHCO_3$ (20 mol%), quencher (x equiv.) then the solvent DMF (0.1 M) was added subsequently. The tube was capped with a rubber plunger. An CO_2 balloon was equipped to bubble for 10 min and keep the CO_2 atmosphere, then resulting mixture was stirred for 20 h under irradiation. The results shown in Table 5, when 2,2,6,6-tetramethyl-1-piperidinyloxyl (TEMPO) were applied to the reaction mixture, the yield dramatically decreased, which was the evidence of the radical pathway. No product was observed when $CuCl_2$ was used as the quencher, indicating the presence of SET (single electron transfer) step during the reaction.

Supplementary Table 5. Quenching experiments

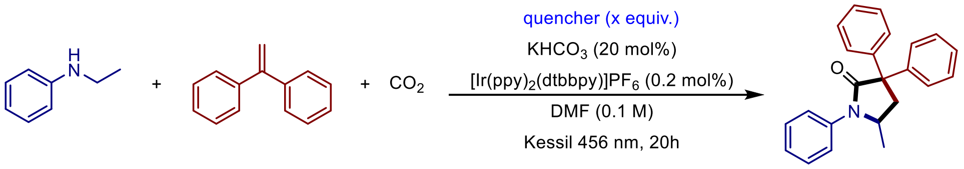

Entry	Quencher	Equiv.	Yield (%)	Conclusion
1	TEMPO	1.0	30	Radical
2	TEMPO	2.0	0	Radical
3	$CuCl_2$	1.0	0	SET

3.6 Stern-Volmer fluorescence quenching experiments

Fluorescence quenching experiments were measured on a SILVER-Nova-25 zAP-D spectrometer. The complex $[Ir(ppy)]_2(dtbbpy)PF_6$ was excited, and the emission spectrum λ_{max} = 470 nm was recorded. The luminescence of $[Ir(ppy)]_2(dtbbpy)PF_6$ at λ_{max} = 470 nm was readily quenched with N-Ethylaniline. The results shown in **Supplementary Figure 6**.

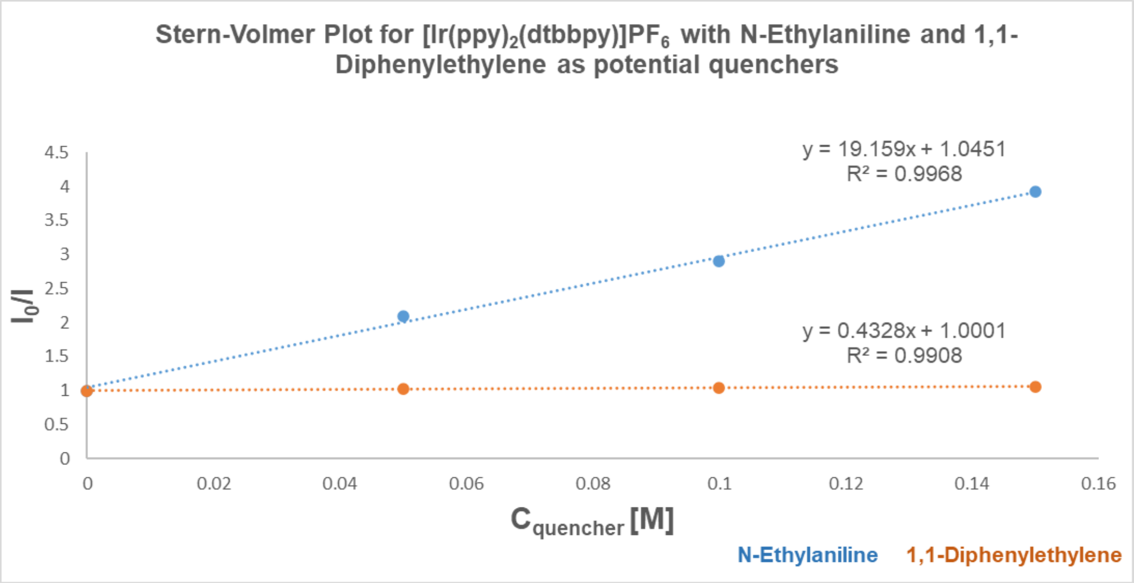

Supplementary Figure 6. Luminescence quenching experiments

3.7 Intercepting the α-amino radical

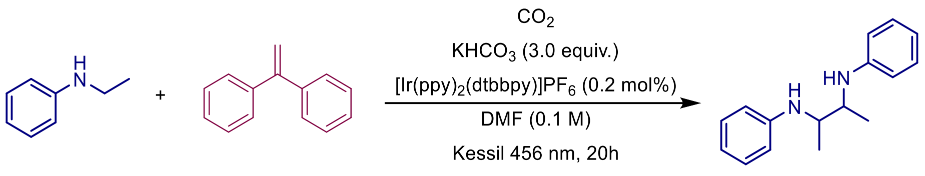

*N*2,*N*3-diphenylbutane-2,3-diamine (**7a**)

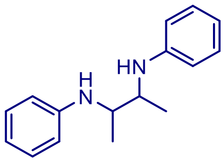

Colorless oil. **mixture of diastereomers and rotamers.** **^{1}H NMR** (400 MHz, $CDCl_3$) δ 7.22 – 7.12 (m, 13H), 6.71 (ddt, J = 8.0, 7.3, 0.9 Hz, 5H), 6.68 – 6.62 (m, 8H), 6.62 – 6.55 (m, 4H), 3.73 (q, J = 5.9 Hz, 2H), 3.66 – 3.54 (m, 10H), 1.22 (d, J = 5.5 Hz, 12H), 1.19 (d, J = 6.2 Hz, 6H). **^{13}C NMR** (100 MHz, $CDCl_3$) δ 147.59, 147.56, 129.38, 129.34, 117.60, 117.53, 113.77, 113.56, 52.59, 52.26, 17.01, 16.27. **HRMS–ESI** (m/z): $[M+H]^+$ calcd. for $C_{16}H_{21}N_2$, 241.1700; found, 241.1702.

3.8 Intercepting the carbanion intermediate with D_2O

A dry 10 mL vial equipped with a stirring bar was charged with the 1,1-Diphenylethylene (0.2 mmol), N-Ethylaniline (0.24 mmol), $[Ir(ppy)_2(dtbbpy)]PF_6$ (0. 2 mol%), and $KHCO_3$ (20 mol%), then D_2O (x equiv.) and the solvent DMF (0.1 M) was added. The tube was capped with a rubber plunger, then the mixture was bubbled with N_2 balloon (10 min). After the reaction mixture was thoroughly bubbled, the vial was placed approximately 3 cm from Kessil 456 nm lights. The lights were switched on and the mixture was stirred under irradiation with fan for 20 hours. The tube was opened, and the mixture was diluted with HCl solution (0.1 M, 2.0 mL) and EtOAc (2.0 mL). 1,3,5-Trimethoxybenzene was added, and the layers

were separated. The aqueous layer was extracted with EtOAc (2.0 mL x 3) and the combined organic layers were evaporated under reduced pressure (40 ºC water bath). The crude was solubilised in $CDCl_3$ and analyzed by ^1H NMR spectroscopy to determine the yield (**Supplementary Figure 7**). The results with different equivalent of D_2O shown in **Supplementary Table 6**.

Supplementary Table 6. The isotope labelling experiments with D_2O

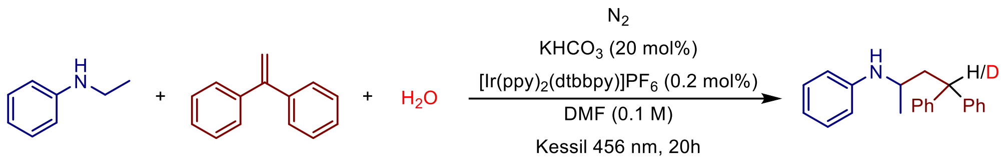

Entry	D_2O (equiv.)	H/D Yield (%)
1	0	82 (0 D)
2	5	86 (53 D)
3	10	98 (90 D)

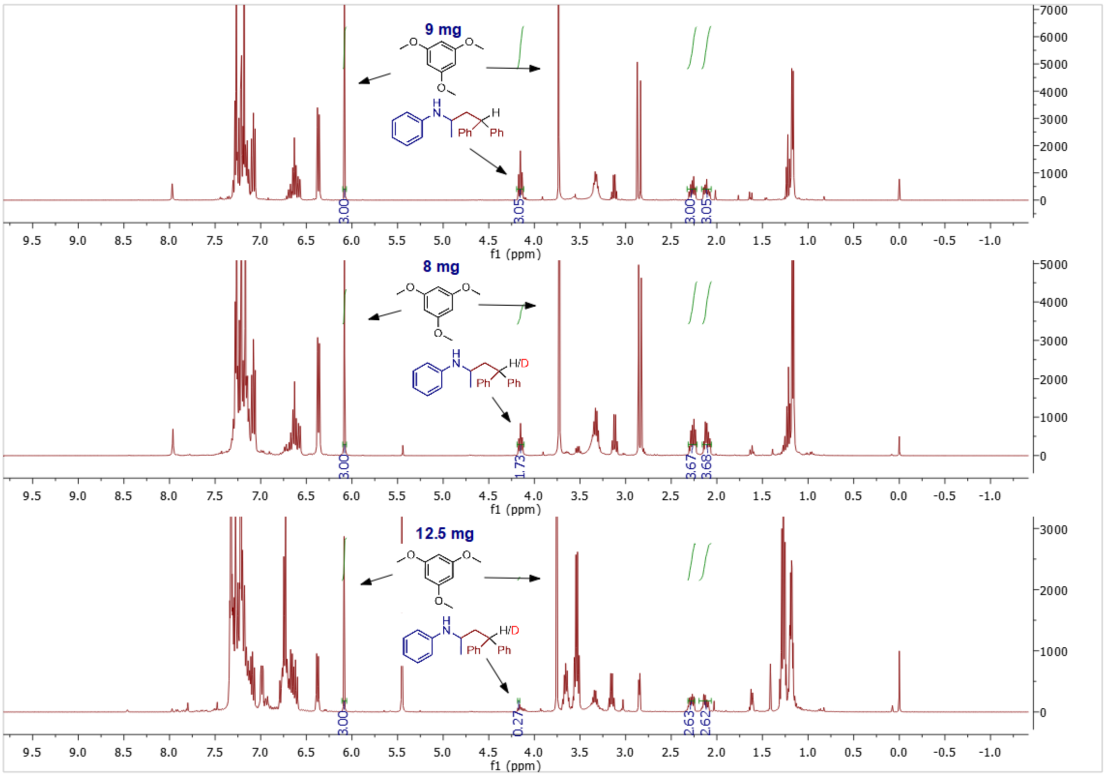

Supplementary Figure 7. Crude ^1H NMR for D_2O isotope experiments

3.9 Intercepting the carbanion intermediate with aldehyde

Following the general procedure, 1,1-Diphenylethylene (0.2 mmol), N-Ethylaniline (0.24 mmol), $[Ir(ppy)_2(dtbbpy)]PF_6$ (0. 2 mol%), and $KHCO_3$ (20 mol%), pentanal (0.4 mmol) then the solvent DMF (0.1 M) was added and reacted for 20h under N_2 atmosphere. The product was isolated by flash column chromatography in 65% yield. This result further supported the existence of benzylic carbanion.

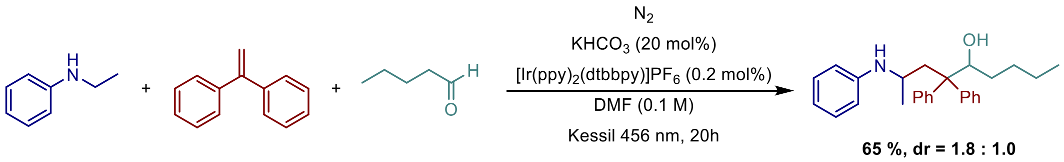

4,4-diphenyl-2-(phenylamino)undecan-5-ol (7b)

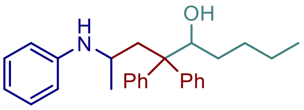

Colorless oil. **^{1}H NMR** (400 MHz, $CDCl_3$) δ 7.37 (ddd, J = 8.1, 6.7, 1.4 Hz, 5.6H), 7.30 (td, J = 8.2, 7.7, 3.8 Hz, 6H), 7.27 – 7.21 (m, 11H), 7.21 – 7.15 (m, 5.6H), 7.15 – 7.09 (m, 3.6H), 7.06 (dd, J = 8.5, 7.2 Hz, 2H), 6.74 (tt, J = 7.3, 1.1 Hz, 1.8H), 6.65 (tt, J = 7.3, 1.1 Hz, 1H), 6.51 – 6.46 (m, 3.6H), 6.31 – 6.24 (m, 2H), 4.44 (dd, J = 10.3, 1.3 Hz, 2.8H), 3.27 (td, J = 6.3, 4.8 Hz, 1H), 3.20 – 3.14 (m, 1.8H), 2.77 (dd, J = 14.6, 8.5 Hz, 2H), 2.48 – 2.35 (m, 1.8H), 2.19 (dd, J = 14.5, 1.6 Hz, 1.8H), 1.56 (dddd, J = 14.8, 8.4, 4.2, 2.5 Hz, 2H), 1.51 – 1.39 (m, 3.8H), 1.31 – 1.14 (m, 8.4H), 0.98 (t, J = 6.0 Hz, 8.4H), 0.79 (dt, J = 14.5, 7.1 Hz, 8.4H). **^{13}C NMR** (100 MHz, $CDCl_3$) δ 146.80, 146.68, 146.02, 145.92, 145.20, 144.20, 129.79, 129.26, 129.23, 129.18, 129.02, 128.95, 127.94, 127.87, 127.67, 127.61, 126.27, 126.04, 126.00, 119.07, 117.80, 115.63, 114.10, 75.50, 74.18, 56.24, 55.80, 47.94, 47.77, 46.62, 45.23, 33.54, 33.13, 29.16, 29.10, 22.97, 22.74, 22.68, 22.54, 14.07, 14.01. **HRMS–ESI** (m/z): $[M+H]^+$ calcd. for $C_{27}H_{34}NO$, 388.2635; found, 388.2647.

3.10 Defluorinative alkylation of trifluoromethylakene

A dry 10 mL vial equipped with a stirring bar was charged with the (3,3,3-trifluoroprop-1-en-2-yl)benzene (0.2 mmol), N-Ethylaniline (0.24 mmol), $[Ir(ppy)_2(dtbbpy)]PF_6$ (0. 2 mol%), and $KHCO_3$ (20 mol%), then the solvent DMF (0.1 M) was added. The tube was capped with a rubber plunger, then the mixture was bubbled with N_2 balloon (10 min). After that, the vial was placed approximately 3 cm from Kessil 456 nm lights and reacted for 20h. The product was isolated by flash column chromatography in 93% yield. This result provided a solid existence for the existence of benzylic carbanion.

N-(5,5-difluoro-4-phenylpent-4-en-2-yl)aniline (7c)

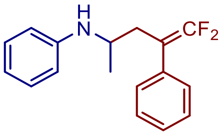

Colorless oil. **^{1}H NMR** (400 MHz, $CDCl_3$) δ 7.40 – 7.32 (m, 2H), 7.34 – 7.27 (m, 3H), 7.10 (dd, J = 8.6, 7.3 Hz, 2H), 6.73 – 6.59 (m, 1H), 6.40 (dd, J = 8.6, 1.1 Hz, 2H), 3.53 – 3.41 (m, 1H), 3.35 (br s, 1H), 2.84 – 2.68 (m, 1H), 2.43 (dddd, J = 14.3, 7.6, 2.5, 1.4 Hz, 1H), 1.17 (d, J = 6.3 Hz, 3H). **^{13}C NMR** (100 MHz, $CDCl_3$) δ 147.02, 129.23, 128.55, 128.40 (t, J = 3.6, 3.1 Hz), 127.53, 117.24, 113.20, 47.15 (dd, J = 3.7, 2.9, 2.8 Hz), 35.13 (d, J = 1.6 Hz), 20.39. **^{19}F NMR** (376 MHz, $CDCl_3$) δ -90.77 – -91.30 (m). **HRMS–ESI** (m/z): $[M+H]^+$ calcd. for $C_{17}H_{18}NF$, 274.1402; found, 274.1396.

3.11 $^{13}CO_2$ labelling experiment

The result of the $^{13}CO_2$-labeling experiment supports the proposed mechanism. When the reaction was carried out under $^{13}CO_2$ atmosphere, the ^{13}C labeled product was observed by HR-MS (**Supplementary Figure 8**). The result is consistent with the proposed mechanism, in which the carbonyl group in γ-lactam arises from the CO_2 molecule.

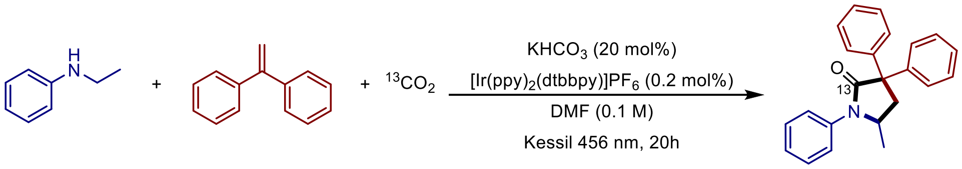

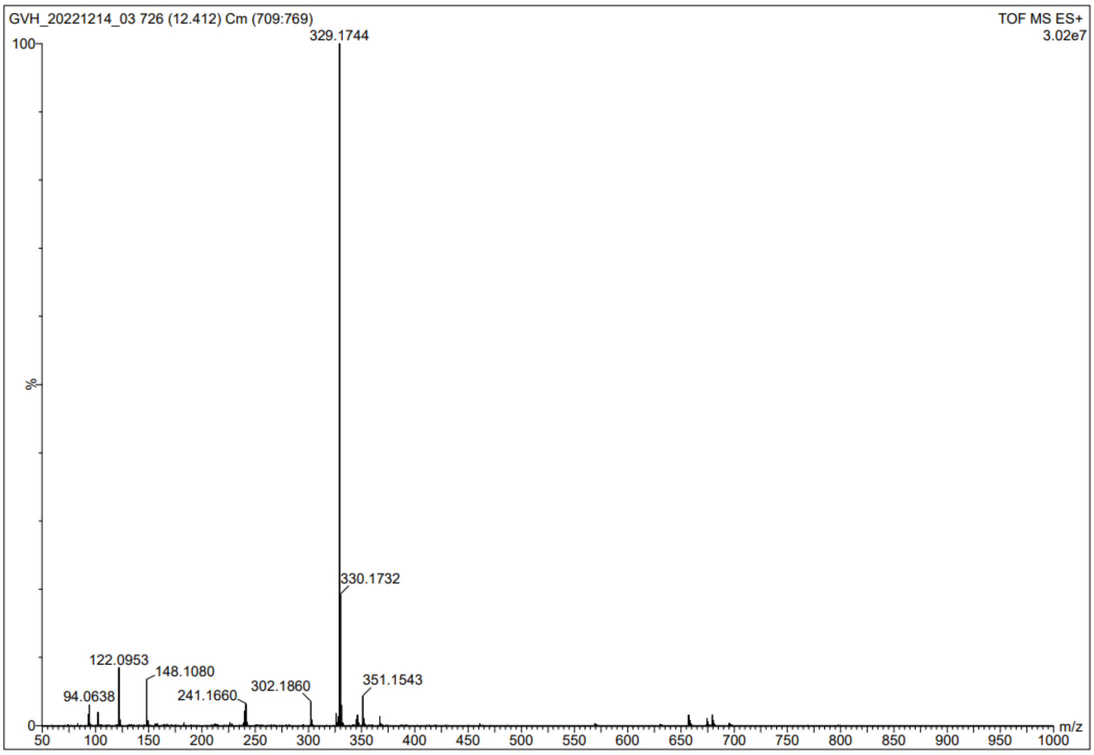

Supplementary Figure 8. HR-MS for $^{13}CO_2$ labelling experiment

3.12 Light on-off experiments

To eight 10 mL vials equipped with a magnetic stir bar were added 1,1-Diphenylethylene (0.2 mmol), N-Ethylaniline (0.24 mmol), $[Ir(ppy)_2(dtbbpy)]PF_6$ (0. 2 mol%), $KHCO_3$ (20 mol%) and DMF (1.0 mL) respectively, the tubes were sealed and equipped with CO_2 balloon for bubbling and keeping the CO_2 atmosphere. The reaction mixture was stirred at room temperature under kessil light (456 nm). Turn on/off the light every 2 hours and quenched one reaction with 0.1 M HCl at the same time until all the reactions were quenched. Each reaction mixture was extracted with ethyl acetate (3 x 2 mL). The combined organic layers were concentrated under reduced pressure (40 ºC water bath). The yields were determined ^{1}H NMR using 1,3,5-Trimethoxybenzene as an internal standard. As shown in **Supplementary Figure 9**, the reaction in the absence of Kessil light showed an extremely slow reaction rate, which highlights the importance of light irradiation.

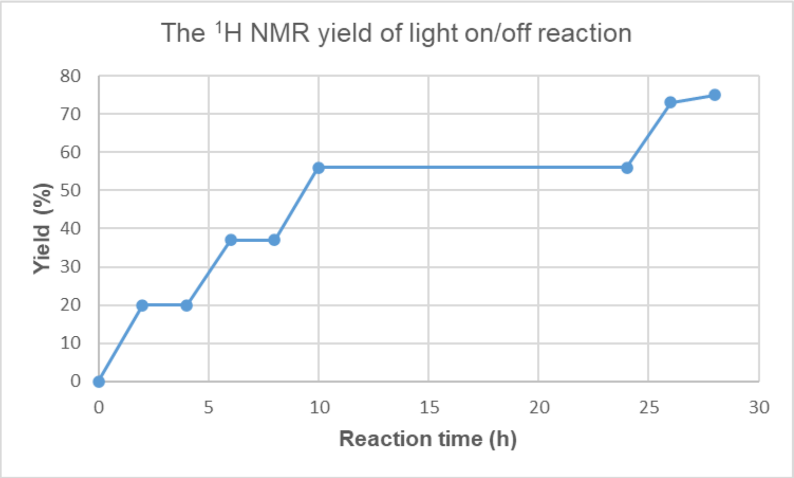

Supplementary Figure 9. The yield of light on/off experiments

3.13 Time course experiments

During the gram-scale reaction, 0.5 mL of the reaction mixture was taken out for ^{1}H NMR analysis after 12h, 24h, 48h, and 72h. As shown in **Supplementary Figure 10**, the yield of γ-lactam continuously increased with the time and the crude ^{1}H NMR spectra were followed in **Supplementary Figure 11**.

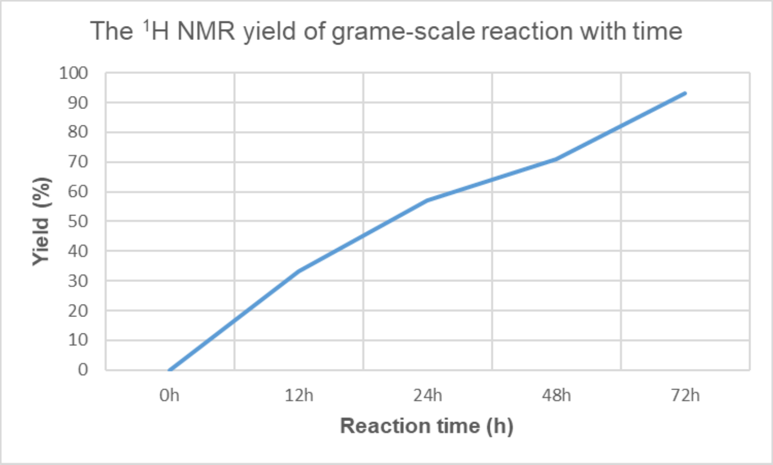

Supplementary Figure 10. The yield of time course experiments

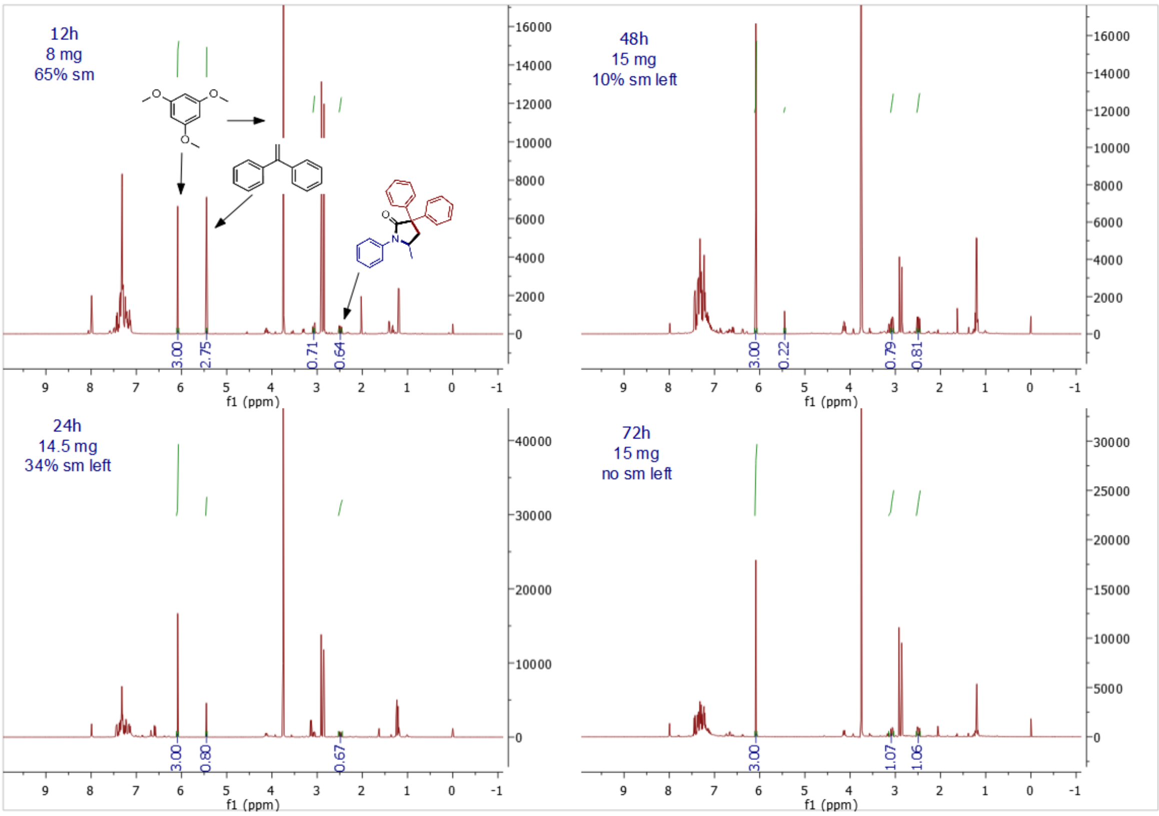

Supplementary Figure 11. The crude ^{1}H NMR of time course experiments of gram scale

4. Characterization of Starting Materials and Products.

N-(2,2-diethoxyethyl)aniline (S1)

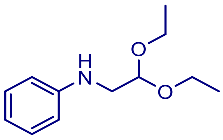

Colorless oil. ^{1}**H NMR** (400 MHz, $CDCl_3$) δ 7.17 (t, J = 5.5 Hz, 2H), 6.71 (td, J = 7.3, 1.1 Hz, 1H), 6.63 (d, J = 7.8 Hz, 2H), 4.68 (t, J = 5.5 Hz, 1H), 3.87 (br s, 1H), 3.72 (dq, J = 9.5, 7.0 Hz, 2H), 3.57 (dq, J = 9.5, 7.0 Hz, 2H), 3.25 (d, J = 5.5 Hz, 2H), 1.23 (t, J = 7.1 Hz, 6H). ^{13}**C NMR** (100 MHz, $CDCl_3$) δ 147.95, 129.22, 117.63, 113.09, 100.89, 62.30, 46.38, 15.35.

N-hexylaniline (S2)

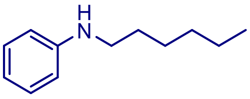

Colorless oil. ^{1}**H NMR** (400 MHz, $CDCl_3$) δ 7.16 (t, J = 7.9 Hz, 2H), 6.67 (t, J = 7.3 Hz, 1H), 6.59 (d, J = 7.7 Hz, 2H), 3.56 (br s, 1H), 3.09 (t, J = 7.1 Hz, 2H), 1.65 – 1.55 (m, 2H), 1.48 – 1.26 (m, 6H), 0.90 (t, J = 7.0 Hz, 3H). ^{13}**C NMR** (100 MHz, $CDCl_3$) δ 148.54, 129.17, 117.03, 112.66, 44.00, 31.64, 29.56, 26.84, 22.61, 14.01. **HRMS–ESI** (m/z): $[M+H]^+$ calcd. for $C_{12}H_{20}N$, 178.1591; found, 178.1582.

N-isobutylaniline (S3)

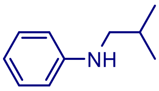

Colorless oil. ^{1}**H NMR** (400 MHz, $CDCl_3$) δ 7.15 (t, J = 7.7 Hz, 2H), 6.66 (t, J = 7.3 Hz, 1H), 6.58 (d, J = 8.5 Hz, 2H), 3.61 (br s, 1H), 2.91 (d, J = 6.9 Hz, 2H), 1.86 (dp, J = 13.3, 6.6 Hz, 1H), 0.97 (d, J = 7.0 Hz, 6H). ^{13}**C NMR** (100 MHz, $CDCl_3$) δ 148.55, 129.15, 116.90, 112.61, 51.78, 28.01, 20.43. **HRMS–ESI** (m/z): $[M+H]^+$ calcd. for $C_{10}H_{16}N$, 150.1278; found, 150.1270.

N-(adamantan-1-ylmethyl)aniline (S4)

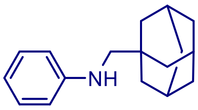

White solid. ^{1}**H NMR** (400 MHz, $CDCl_3$) δ 7.18 – 7.09 (m, 2H), 6.68 – 6.58 (m, 3H), 3.64 (br s, 1H), 2.79 (s, 1H), 2.00 (p, J = 3.2 Hz, 3H), 1.79 – 1.63 (m, 6H), 1.58 (d, J = 2.9 Hz, 6H). ^{13}**C NMR** (100 MHz, $CDCl_3$) δ 149.25, 129.16, 116.70, 112.57, 56.29, 40.77, 37.13, 33.91, 28.41. **HRMS–ESI** (m/z): $[M+H]^+$ calcd. for $C_{17}H_{24}N$, 242.1904; found, 242.1893.

N-(2-methoxyethyl)aniline (S5)

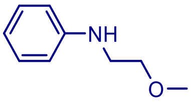

Colorless oil. ^{1}**H NMR** (400 MHz, $CDCl_3$) δ 7.17 (t, J = 7.1 Hz, 2H), 6.71(t, J = 7.3 Hz, 1H), 6.63 (d, J = 7.8 Hz, 2H), 4.00 (s, 1H), 3.60 (t, J = 5.3 Hz, 2H), 3.38 (s, 3H), 3.28 (t, J = 4.9 Hz, 2H). ^{13}**C NMR** (100 MHz, $CDCl_3$) δ 148.20, 129.16, 117.53, 113.04, 71.01, 58.67, 43.44. **HRMS–ESI** (m/z): $[M+H]^+$ calcd. for $C_9H_{14}NO$, 152.1070; found, 152.1063.

N-(2-((*tert*-butyldimethylsilyl)oxy)ethyl)aniline (S6)

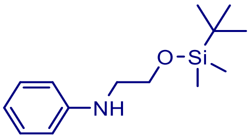

Colorless oil. **^{1}H NMR** (400 MHz, $CDCl_3$) δ 7.18 (t, J = 7.4 Hz, 2H), 6.71 (td, J = 7.3, 1.0 Hz, 1H), 6.63 (d, J = 7.8 Hz, 2H), 4.03 (br s, 1H), 3.82 (t, J = 5.5 Hz, 2H), 3.25 – 3.17 (m, 2H), 0.92 (s, 9H), 0.07 (s, 6H). **^{13}C NMR** (100 MHz, $CDCl_3$) δ 148.40, 129.20, 117.53, 113.22, 61.64, 46.03, 25.91, 18.31, -5.33. **HRMS–ESI** (m/z): $[M+H]^+$ calcd. for $C_{14}H_{26}NOSi$, 252.1779; found, 252.1784.

***tert*-butyl 3-((phenylamino)methyl)piperidine-1-carboxylate (S7)**

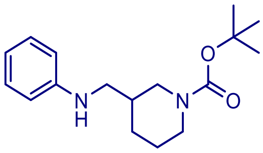

White solid. **^{1}H NMR** (400 MHz, $CDCl_3$) δ 7.21 – 7.10 (m, 2H), 6.69 (t, J = 7.3 Hz, 1H), 6.59 (d, J = 7.6 Hz, 2H), 4.12 (d, J = 7.1 Hz, 2H), 3.03 (d, J = 6.2 Hz, 2H), 2.69 (t, J = 12.8 Hz, 2H), 1.84 – 1.65 (m, 3H), 1.46 (s, 9 H), 1.18 (ddd, J = 12.3, 4.7, 1.7 Hz, 2H). **^{13}C NMR** (100 MHz, $CDCl_3$) δ 154.83, 148.19, 129.28, 117.29, 112.68, 79.37, 49.73, 36.07, 30.20, 28.47. **HRMS–ESI** (m/z): $[M+Na]^+$ calcd. for $C_{17}H_2N_2O_2Na$, 313.1886; found, 313.1884.

***N*-propyl-[1,1'-biphenyl]-4-amine (S8)**

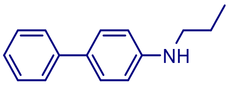

Colorless oil. **^{1}H NMR** (400 MHz, , $CDCl_3$) δ 7.53 (d, J = 7.1 Hz, 2H), 7.37 (t, J = 7.7 Hz, 2H), 7.28 – 7.18 (m, 1H), 7.04 (dd, J = 309.8, 8.6 Hz, 4H), 3.69 (br s, 1H), 3.10 (t, J = 7.1 Hz, 2H), 1.64 (h, J = 7.3 Hz, 2H), 1.00 (t, J = 7.4 Hz, 3H). **^{13}C NMR** (100 MHz, $CDCl_3$) δ 147.91, 141.31, 129.93, 128.58, 127.87, 126.21, 125.92, 112.89, 45.79, 22.72, 11.60. **HRMS–ESI** (m/z): $[M+H]^+$ calcd. for $C_{15}H_{18}N$, 212.1434; found, 212.1434.

***N*-(2-(cyclohex-1-en-1-yl)ethyl)aniline (S9)**

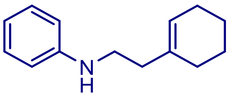

Colorless oil. **^{1}H NMR** (400 MHz, $CDCl_3$) δ 7.17 (ddt, J = 8.5, 4.5, 1.6 Hz, 2H), 6.73 – 6.65 (m, 1H), 6.64 – 6.57 (m, 2H), 5.53 (s, 1H), 3.61 (br s, 1H), 3.16 (td, J = 6.9, 2.9 Hz, 2H), 2.26 (t, J = 7.1 Hz, 2H), 1.99 (d, J = 30.3 Hz, 4H), 1.71 – 1.54 (m, 4H). **^{13}C NMR** (100 MHz, $CDCl_3$) δ 148.45, 134.91, 129.17, 123.52, 117.13, 112.82, 41.45, 37.66, 27.88, 25.26, 22.87, 22.41. **HRMS–ESI** (m/z): $[M+H]^+$ calcd. for $C_{14}H_{20}N$, 202.1591; found, 202.1593.

4-(*tert*-butyl)-*N*-propylaniline (S10)

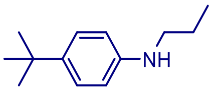

Colorless oil. **^{1}H NMR** (400 MHz, $CDCl_3$) δ 6.87 (dd, J = 254.5, 8.7 Hz, 4H), 3.06 (t, J = 7.1 Hz, 2H), 1.62 (h, J = 7.3 Hz, 2H), 1.27 (s, 9H), 0.98 (t, J = 7.4 Hz, 3H). **^{13}C NMR** (100 MHz, $CDCl_3$) δ 146.19, 139.84, 125.95, 112.42, 77.32, 77.00, 76.68, 46.07, 33.80, 31.55, 22.83, 11.64. **HRMS–ESI** (m/z): $[M+H]^+$ calcd. for $C_{13}H_{22}N$, 192.1747; found, 192.1749.

4-iodo-*N*-propylaniline (S11)

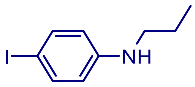

Brown oil. **^{1}H NMR** (400 MHz, $CDCl_3$) δ 6.85 (dd, J = 414.8, 8.4 Hz, 4H), 3.62 (br s, 1H), 2.99 (t, J = 7.2 Hz, 2H), 1.58 (h, J = 7.3 Hz, 2H), 0.96 (t, J = 6.9 Hz, 3H). **^{13}C NMR** (100 MHz, $CDCl_3$**)** δ 147.85, 137.50, 114.74, 77.18, 45.41, 22.41, 11.50.

3-methoxy-*N*-propylaniline (S12)

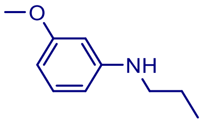

Colorless oil. **^{1}H NMR** (400 MHz, $CDCl_3$) δ 7.07 (t, J = 8.1 Hz, 1H), 6.28 – 6.20 (m, 2H), 6.17 (q, J = 2.3 Hz, 1H), 3.78 (s, 3H), 3.63 (br s, 1H), 3.08 (t, J = 7.1 Hz, 2H), 1.64 (h, J = 7.3 Hz, 2H), 1.00 (t, J = 7.4 Hz, 3H). **^{13}C NMR** (100 MHz, $CDCl_3$) δ 160.87, 149.91, 129.89, 105.92, 102.19, 98.63, 55.05, 45.79, 22.70, 11.60. **HRMS–ESI** (m/z): $[M+H]^+$ calcd. for $C_{10}H_{16}NO$, 166.1227; found, 166.1226.

methyl 4-(propylamino)benzoate (S13)

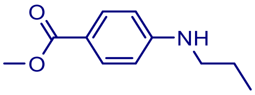

White solid**.** **^{1}H NMR** (400 MHz, $CDCl_3$) δ 7.19 (dd, J = 526.1, 8.8 Hz, 4H), 4.12 (br s, 1H), 3.84 (s, 3H), 3.13 (t, J = 7.1 Hz, 2H), 1.65 (q, J = 7.3 Hz, 2H), 1.00 (t, J = 7.4 Hz, 3H). **^{13}C NMR** (100 MHz, $CDCl_3$) δ 167.32, 152.12, 131.52, 118.04, 111.30, 51.44, 45.14, 22.52, 11.50. **HRMS–ESI** (m/z): $[M+H]^+$ calcd. for $C_{11}H_{16}NO_2$, 194.1176; found, 194.1179.

***N*-(((1R,2S,5R)-6,6-dimethylbicyclo[3.1.1]heptan-2-yl)methyl)aniline (S14)**

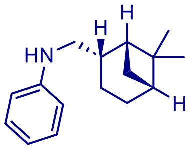

White solid. **^{1}H NMR** (400 MHz, $CDCl_3$) δ 7.20 – 7.13 (m, 2H), 6.67 (t, J = 7.3 Hz, 1H), 6.58 (d, J = 7.7 Hz, 2H), 3.65 (br s, 1H), 3.17 – 3.04 (m, 2H), 2.44 – 2.26 (m, 2H), 2.07 – 1.82 (m, 5H), 1.61 – 1.48 (m, 1H), 1.21 (s, 3H), 1.04 (s, 3H), 0.92 (d, J = 9.6 Hz, 1H). **^{13}C NMR** (100 MHz, $CDCl_3$) δ 148.58, 129.21, 116.96, 112.66, 49.99, 44.22, 41.52, 41.21, 38.74, 33.44, 28.07, 26.17, 23.34, 20.42. **HRMS–ESI** (m/z): $[M+H]^+$ calcd. for $C_{16}H_{24}N$, 230.1904; found, 230.1913.

***N*-(((1*S*,4a*R*,10a*S*)-7-isopropyl-1,4a-dimethyl-1,2,3,4,4a,9,10,10a-octahydrophenanthren-1-yl)methyl) aniline (S15)**

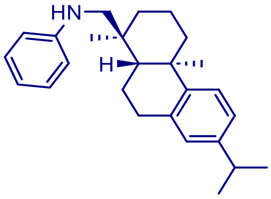

Colorless oil. **^{1}H NMR** (400 MHz, $CDCl_3$) δ 7.22 – 7.13 (m, 1H), 7.17 – 7.10 (m, 2H), 7.00 (dd, J = 8.2, 2.1 Hz, 1H), 6.91 – 6.86 (m, 1H), 6.69 – 6.60 (m, 1H), 6.63 – 6.56 (m, 2H), 3.58 (br s, 1H), 3.09 – 2.97 (m, 1H), 2.89 (ddt, J = 12.4, 5.8, 3.2 Hz, 2H), 2.86 – 2.74 (m, 2H), 2.30 (dt, J = 13.4, 3.7 Hz, 1H), 1.80 (d, J = 17.1 Hz, 3H), 1.73 – 1.58 (m, 3H), 1.50 – 1.39 (m, 2H), 1.23 (d, J = 1.9 Hz, 6H), 1.21 (s, 3H), 1.01 (s, 3H). **^{13}C NMR** (100 MHz, $CDCl_3$) δ 148.98, 147.31, 145.66, 134.70, 129.20, 126.85, 124.21, 123.89, 116.87, 112.63, 54.92, 45.35, 38.46, 37.50, 36.35, 33.45, 30.10, 25.28, 23.97, 19.29, 18.91, 18.76. **HRMS–ESI** (m/z): $[M+H]^+$ calcd. for $C_{26}H_{36}N$, 362.2843; found, 362.2833.

(*Z*)-*N*-(octadec-9-en-1-yl)aniline (S16)

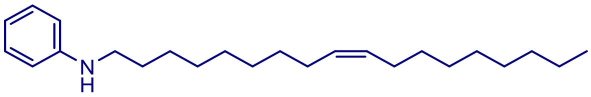

Colorless oil. **^{1}H NMR** (400 MHz, $CDCl_3$) δ 7.20 – 7.12 (m, 2H), 6.67 (t, J = 7.3 Hz, 1H), 6.59 (d, J = 7.9 Hz, 2H), 5.40 – 5.32 (m, 2H), 3.57 (br s, 1H), 3.09 (t, J = 7.1 Hz, 2H), 2.01 (q, J = 6.4 Hz, 3H), 1.61 (p, J = 7.2 Hz, 2H), 1.40 – 1.21 (m, 23H), 0.88 (t, J = 6.8 Hz, 3H). **^{13}C NMR** (100 MHz, $CDCl_3$) δ 148.54,

129.97, 129.79, 129.19, 117.06, 112.68, 44.01, 31.91, 29.77, 29.75, 29.61, 29.52, 29.48, 29.43, 29.33, 29.32, 29.24, 27.23, 27.19, 27.18, 22.68, 14.09.

(3*R*,8*R*,9*R*,10*S*,13*S*,14*R*,17*S*)-10,13-dimethyl-17-((*S*)-6-methylheptan-2-yl)-2,3,4,7,8,9,10,11,12, 13,14,15,16,17-tetradecahydro-1*H*-cyclopenta[*a*]phenanthren-3-yl-4-(propylamino)benzoate (S17)

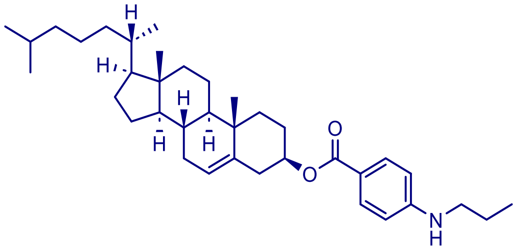

White solid. **^{1}H NMR** (400 MHz, $CDCl_3$) δ 7.21 (dd, J = 528.2, 8.7 Hz, 4H), 5.40 (d, J = 4.8 Hz, 1H), 4.88 – 4.71 (m, 1H), 4.07 (br s, 1H), 3.13 (q, J = 6.3, 5.7 Hz, 2H), 2.43 (d, J = 7.2 Hz, 2H), 2.07 – 1.93 (m, 3H), 1.94 – 1.74 (m, 2H), 1.74 – 1.61 (m, 3H), 1.62 – 1.42 (m, 7H), 1.34 (d, J = 8.0 Hz, 3H), 1.28 – 1.08 (m, 7H), 1.06 (s, 3H), 1.00 (t, J = 7.4 Hz, 6H), 0.92 (d, J = 6.5 Hz, 3H), 0.88 (d, J = 1.8 Hz, 3H), 0.86 (d, J = 1.8 Hz, 3H), 0.69 (s, 3H). **^{13}C NMR** (100 MHz, $CDCl_3$) δ 166.24, 151.99, 140.01, 131.49, 122.45, 118.84, 111.27, 73.64, 56.73, 56.18, 50.10, 45.20, 42.35, 39.79, 39.54, 38.40, 37.12, 36.69, 36.21, 35.81, 31.96, 31.93, 28.24, 28.02, 24.31, 23.85, 22.81, 22.56, 21.07, 19.40, 18.73, 11.87, 11.53. **HRMS–ESI** (m/z): $[M+H]^+$ calcd. for $C_{37}H_{58}NO_2$, 548.4463; found, 548.4460.

(Z)-3,7-dimethylocta-2,6-dien-1-yl 4-(propylamino)benzoate (S18)

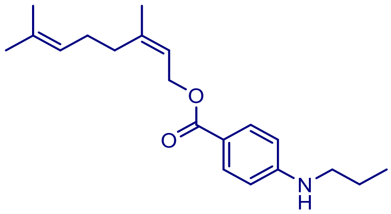

Colorless oil. **^{1}H NMR** (400 MHz, $CDCl_3$) δ 7.19 (dd, J = 533.2, 8.7 Hz, 4H), 5.47 (td, J = 7.1, 1.5 Hz, 1H), 5.12 (tdd, J = 6.8, 2.9, 1.5 Hz, 1H), 4.75 (dd, J = 7.2, 1.1 Hz, 2H), 4.15 – 4.00 (m, 1H), 3.13 (td, J = 7.1, 5.5 Hz, 2H), 2.21 – 2.03 (m, 4H), 1.78 (s, 3H), 1.67 (s, 3H), 1.66 – 1.62 (m, 2H), 1.60 (s, 3H), 1.00 (t, J = 7.4 Hz, 3H). **^{13}C NMR** (100 MHz, $CDCl_3$) δ 166.88, 152.04, 141.89, 132.06, 131.56, 123.71, 119.90, 118.45, 111.27, 60.88, 45.17, 32.25, 26.72, 25.66, 23.51, 22.55, 17.66, 11.52. **HRMS–ESI** (m/z): $[M+H]^+$ calcd. for $C_{20}H_{30}NO_2$, 316.2272; found, 316.2286.

2,5,7,8-tetramethyl-2-(4,8,12-trimethyltridecyl)chroman-6-yl 4-(propylamino)benzoate (S19)

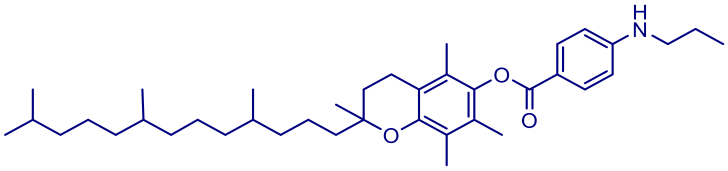

White solid. **^{1}H NMR** (400 MHz, $CDCl_3$) δ 7.32 (dd, J = 569.8, 8.3 Hz, 4H), 4.18 (t, J = 5.6 Hz, 1H), 3.18 (td, J = 7.1, 5.5 Hz, 2H), 2.60 (t, J = 6.9 Hz, 2H), 2.11 (s, 3H), 2.05 (s, 3H), 2.01 (s, 3H), 1.79 (dt, J = 11.4, 6.5 Hz, 2H), 1.68 (q, J = 7.2 Hz, 2H), 1.52 (d, J = 1.6 Hz, 4H), 1.39 (tt, J = 10.7, 5.4 Hz, 4H), 1.34 – 1.19 (m, 10H), 1.19 – 1.03 (m, 6H), 1.02 (t, J = 7.4 Hz, 3H), 0.86 (t, J = 7.1 Hz, 12H). **^{13}C NMR** (100 MHz, $CDCl_3$) δ 165.31, 152.49, 149.20, 132.24, 127.22, 125.38, 122.90, 117.38, 111.43, 74.97, 45.17, 39.39, 37.48, 37.41, 37.31, 32.81, 32.80, 32.75, 32.67, 27.99, 24.81, 24.46, 22.72, 22.62, 22.55, 21.07, 20.64, 19.76, 19.69, 19.65, 19.61, 13.05, 12.19, 11.82, 11.53. **HRMS–ESI** (m/z): $[M+H]^+$ calcd. for $C_{39}H_{62}NO_3$, 592.4725; found, 592.4721.

1-methyl-2-(1-phenylvinyl)benzene (S20)

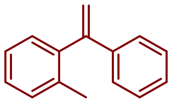

Colorless oil. **^{1}H NMR (400 MHz, $CDCl_3$)** *δ* 7.29 – 7.23 (m, 6H), 7.23 – 7.20 (m, 2H), 7.20 – 7.16 (m, 1H), 5.77 (d, J = 1.4 Hz, 1H), 5.19 (d, J = 1.4 Hz, 1H), 2.05 (s, 3H). **^{13}C NMR (100 MHz, $CDCl_3$)** *δ* 149.47, 141.63, 140.60, 136.12, 130.06, 130.00, 128.31, 127.54, 127.50, 126.47, 125.65, 114.81, 20.08.

4-(1-phenylvinyl)benzonitrile (S21)

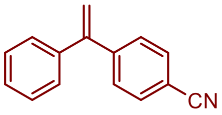

Colorless oil. **^{1}H NMR (400 MHz, $CDCl_3$)** δ 7.53 (dd, J = 73.9, 8.4 Hz, 4H), 7.38 – 7.32 (m, 3H), 7.31 – 7.22 (m, 2H), 5.56 (dd, J = 17.5, 0.8 Hz, 2H). **^{13}C NMR (100 MHz, $CDCl_3$)** *δ* 148.73, 146.08, 140.21, 132.06, 128.84, 128.45, 128.25, 128.15, 118.83, 116.67, 111.39.

1-methoxy-4-(1-phenylvinyl)benzene (S22)

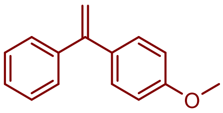

Colorless oil. **^{1}H NMR** (400 MHz, $CDCl_3$) δ 7.35 – 7.29 (m, 5H), 7.10 (dd, J = 163.4, 7.2 Hz, 4H), 5.37 (dd, J = 17.2, 1.3 Hz, 2H), 3.82 (s, 3H). **^{13}C NMR** (100 MHz, $CDCl_3$) *δ* 159.35, 149.53, 141.83, 134.00, 129.37, 128.30, 128.10, 127.62, 113.53, 112.90, 55.28. **HRMS–ESI** (m/z): $[M+H]^+$ calcd. for $C_{15}H_{15}O$, 211.1118; found, 211.1108.

1-fluoro-4-(1-(4-(phenylethynyl)phenyl)vinyl)benzene (S23)

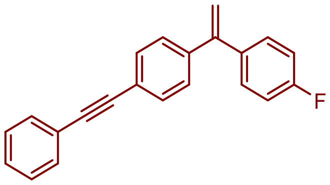

Colorless oil. **^{1}H NMR** (400 MHz, $CDCl_3$) *δ* 7.58 – 7.52 (m, 2H), 7.51 – 7.47 (m, 2H), 7.34 (dd, J = 5.1, 2.0 Hz, 3H), 7.31 – 7.26 (m, 4H), 7.02 (t, J = 8.7 Hz, 2H), 5.45 (dd, J = 20.2, 1.0 Hz, 2H). **^{13}C NMR** (100 MHz, $CDCl_3$) *δ* 163.82, 161.37, 148.51, 141.12, 137.21, 137.18, 131.56 (d, J = 12.1 Hz), 129.92 (d, J = 8.0 Hz), 128.30, 128.23 (d, J = 24.6 Hz), 123.03 (d, J = 44.0 Hz), 115.14 (d, J = 21.6 Hz), 114.74, 89.67 (d, J = 91.5 Hz). **^{19}F NMR** (376 MHz, $CDCl_3$) *δ* -114.39.

isopropyl 2-(4-(1-(4-chlorophenyl)vinyl)phenoxy)-2-methylpropanoate (S24)

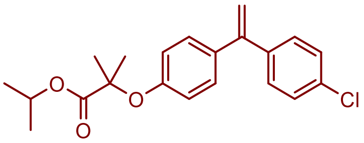

Colorless oil. **^{1}H NMR** (400 MHz, $CDCl_3$) *δ* 7.32 – 7.22 (m, 5H), 6.98 (dd, J = 152.6, 8.7 Hz, 4H), 5.36 (dd, J = 26.0, 1.2 Hz, 2H), 5.13 – 5.03 (m, 1H), 1.60 (s, 6H), 1.22 (d, J = 6.3 Hz, 6H). **^{13}C NMR** (100 MHz, $CDCl_3$) *δ* 173.60, 155.51, 148.38, 140.19, 134.48, 133.52, 129.59, 128.8j6, 128.29, 118.45, 113.54, 79.15, 68.97, 25.41, 21.56. **HRMS–ESI** (m/z): $[M+Na]^+$ calcd. for $C_{21}H_2O_3ClNa$, 381.1227; found, 381.1227.

(2S,5R)-2-isopropyl-5-methylcyclohexyl 4-vinylbenzoate (S25)

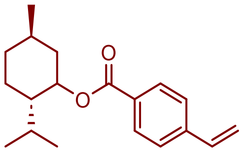

Colorless oil. **^{1}H NMR** (400 MHz, $CDCl_3$) δ 7.72 (dd, J = 216.9, 8.4 Hz, 4H), 6.75 (dd, J = 17.6, 10.9 Hz, 1H), 5.85 (d, J = 17.4 Hz, 1H), 5.37 (d, J = 10.9 Hz, 1H), 4.93 (td, J = 10.9, 4.4 Hz, 1H), 2.13 (dtd, J = 12.0, 3.9, 1.8 Hz, 1H), 2.01 – 1.86 (m, 1H), 1.79 – 1.68 (m, 2H), 1.56 (tt, J = 8.7, 1.9 Hz, 2H), 1.38 – 1.21 (m, 1H), 1.12 (dtd, J = 15.2, 12.4, 9.7 Hz, 1H), 0.92 (dd, J = 6.8, 5.2 Hz, 6JH), 0.80 (d, J = 7.0 Hz, 3H). **^{13}C NMR** (100 MHz, $CDCl_3$) δ 165.83, 141.74, 136.09, 129.85, 126.03, 116.27, 74.81, 55.74, 47.31, 41.00, 34.35, 31.45, 26.56, 25.47, 24.68, 23.73, 22.03, 20.74, 16.58. **HRMS–ESI** (m/z): $[M+Na]^+$ calcd. for $C_{19}H_2O_2Na$, 309.1825; found, 309.1822.

2-(2-fluoro-[1,1'-biphenyl]-4-yl)propyl 4-vinylbenzoate (S26)

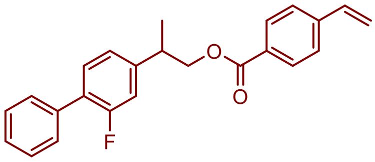

White solid. **^{1}H NMR** (400 MHz, $CDCl_3$) δ 7.95 (d, J = 8.4 Hz, 2H), 7.54 (dt, J = 8.2, 1.5 Hz, 2H), 7.50 – 7.42 (m, 4H), 7.42 – 7.31 (m, 2H), 7.22 – 6.98 (m, 2H), 6.74 (dd, J = 17.6, 10.9 Hz, 1H), 5.85 (d, J = 16.9 Hz, 1H), 5.38 (d, J = 10.2 Hz, 1H), 4.58 – 4.27 (m, 2H), 3.29 (q, J = 7.0 Hz, 1H), 1.42 (d, J = 7.1 Hz, 3H). **^{13}C NMR** (100 MHz, $CDCl_3$) δ 166.17, 161.00, 144.93, 144.86, 142.04, 136.01, 135.65, 130.70 (d, J = 4.0 Hz), 129.87, 129.28, 128.94 (d, J = 2.8 Hz), 128.41, 127.32 (d, J = 13.6 Hz), 126.14, 123.35 (d, J = 3.2 Hz), 116.52, 114.96 (d, J = 23.2 Hz), 77.32, 77.00, 76.68, 69.45, 38.70 (d, J = 1.1 Hz), 17.91. **^{19}F NMR** (376 MHz, $CDCl_3$) δ -117.99. **HRMS–ESI** (m/z): $[M+Na]^+$ calcd. for $C_{24}H_2O_2FNa$, 383.1417; found, 383.1405.

(S)-2-(4-isobutylphenyl)propyl 4-vinylbenzoate (S27)

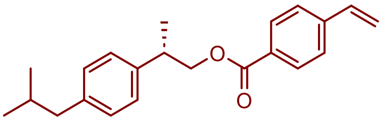

Colorless oil. **^{1}H NMR** (400 MHz, $CDCl_3$) δ 7.68 (dd, J = 201.5, 8.2 Hz, 4H), 7.14 (dd, J = 36.9, 7.7 Hz, 4H), 6.74 (dd, J = 17.6, 10.9 Hz, 1H), 5.84 (d, J = 17.6 Hz, 1H), 5.37 (d, J = 10.8 Hz, 1H), 4.38 (qdd, J = 10.8, 7.1, 1.6 Hz, 2H), 3.22 (h, J = 7.0 Hz, 1H), 2.45 (d, J = 6.8 Hz, 2H), 1.86 (dqd, J = 13.5, 6.8, 1.4 Hz, 1H), 1.38 (d, J = 7.0 Hz, 3H), 0.89 (d, J = 6.7 Hz, 6H). **^{13}C NMR** (100 MHz, $CDCl_3$) δ 166.22, 141.86, 140.34, 140.02, 136.04, 129.83, 129.53, 129.20, 127.01, 126.05, 116.36, 69.99, 45.03, 38.69, 30.18, 22.38, 22.35, 18.03. **HRMS–ESI** (m/z): $[M+Na]^+$ calcd. for $C_{22}H_{26}O_2Na$, 345.1825; found, 345.1834

5-methyl-1,3,3-triphenylpyrrolidin-2-one (3a)

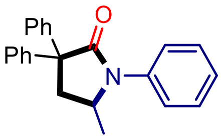

Colorless oil. **^{1}H NMR** (400 MHz, $CDCl_3$) δ 7.47 – 7.42 (m, 2H), 7.41 – 7.35 (m, 4H), 7.34 – 7.26 (m, 6H), 7.26 – 7.18 (m, 3H), 4.16 – 4.10 (m, 1H), 2.78 (ddd, J = 21.8, 12.8, 7.3 Hz, 2H), 1.20 (d, J = 6.1 Hz, 3H). **^{13}C NMR** (100 MHz, $CDCl_3$) δ 174.49, 143.86, 142.17, 137.42, 128.81, 128.50, 128.15, 128.04, 127.66, 126.98, 126.70, 126.00, 124.64, 58.05, 51.45, 43.21, 19.88. **HRMS–ESI** (m/z): $[M+H]^+$ calcd. for $C_{23}H_{22}NO$, 328.1696; found, 328.1694.

5-ethyl-1,3,3-triphenylpyrrolidin-2-one (3b)

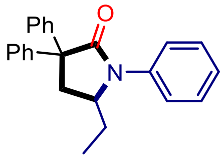

Colorless oil. **^{1}H NMR** (400 MHz, $CDCl_3$) δ 7.46 – 7.42 (m, 2H), 7.41 – 7.35 (m, 4H), 7.35 – 7.27 (m, 6H), 7.27 – 7.19 (m, 3H), 4.00 (tdd, J = 9.0, 5.8, 3.2 Hz, 1H), 2.78 (ddd, J = 21.9, 12.8, 7.4 Hz, 2H), 1.57 (dddd, J = 21.1, 16.3, 8.3, 5.3 Hz, 2H), 0.85 (t, J = 7.5 Hz, 3H). **^{13}C NMR** (100 MHz, $CDCl_3$) δ 174.71, 144.06, 142.26, 137.55, 128.81, 128.50, 128.16, 128.03, 127.69, 126.98, 126.69, 126.10, 124.83, 57.83, 56.65, 40.17, 25.91, 8.52. **HRMS–ESI** (m/z): $[M+H]^+$ calcd. for $C_{24}H_{24}NO$, 342.1852; found, 342.1842.

5-pentyl-1,3,3-triphenylpyrrolidin-2-one (3c)

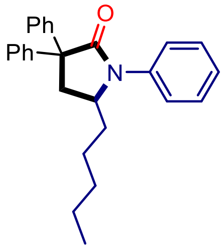

White solid. **^{1}H NMR** (400 MHz, $CDCl_3$) δ 7.46 – 7.42 (m, 2H), 7.38 (dd, J = 8.5, 7.2 Hz, 4H), 7.34 – 7.27 (m, 6H), 7.23 (ddd, J = 7.0, 3.7, 1.0 Hz, 3H), 4.07 – 4.00 (m, 1H), 2.78 (ddd, J = 21.8, 12.8, 7.4 Hz, 2H), 1.82 – 1.64 (m, 1H), 1.40 – 1.09 (m, 7H), 0.82 (t, J = 6.9 Hz, 3H). **^{13}C NMR** (100 MHz, $CDCl_3$) δ 174.63, 144.09, 142.25, 137.57, 128.82, 128.50, 128.17, 128.04, 127.68, 126.98, 126.68, 126.10, 124.85, 57.84, 55.77, 40.98, 33.34, 31.64, 24.19, 22.43, 13.84. **HRMS–ESI** (m/z): $[M+H]^+$ calcd. for $C_{27}H_{30}NO$, 384.2322; found, 384.2318.

5-isopropyl-1,3,3-triphenylpyrrolidin-2-one (3d)

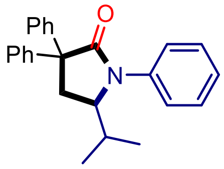

Colorless oil. **^{1}H NMR** (400 MHz, $CDCl_3$) δ 7.43 – 7.35 (m, 6H), 7.34 – 7.27 (m, 6H), 7.26 – 7.20 (m, 3H), 4.10 (ddd, J = 9.9, 5.9, 4.1 Hz, 1H), 2.66 (ddd, J = 22.8, 12.9, 7.9 Hz, 2H), 2.25 – 2.08 (m, 1H), 0.91 (d, J = 7.0 Hz, 3H), 0.72 (d, J = 6.7 Hz, 3H). **^{13}C NMR** (100 MHz, $CDCl_3$) δ 175.05, 144.37, 142.14, 137.50, 128.74, 128.52, 128.17, 128.08, 127.73, 127.03, 126.65, 126.06, 124.86, 59.59, 57.63, 33.98, 26.67, 18.22, 14.04. **HRMS–ESI** (m/z): $[M+H]^+$ calcd. for $C_{25}H_{26}NO$, 356.2009; found, 356.2017.

5-((3r,5r,7r)-adamantan-1-yl)-1,3,3-triphenylpyrrolidin-2-one (3e)

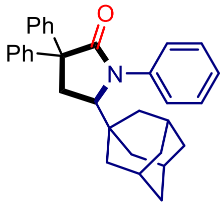

White solid. **^{1}H NMR** (400 MHz, $CDCl_3$) δ 7.42 – 7.37 (m, 4H), 7.36 – 7.28 (m, 6H), 7.28 – 7.22 (m, 3H), 7.22 – 7.17 (m, 2H), 3.80 (dd, J = 9.7, 6.3 Hz, 1H), 2.75 (ddd, J = 22.9, 13.2, 8.0 Hz, 2H), 1.85 (s, 3H), 1.60 (d, J = 12.1 Hz, 3H), 1.46 (d, J = 12.6 Hz, 6H), 1.37 (d, J = 12.0 Hz, 3H). **^{13}C NMR** (100 MHz, $CDCl_3$) δ 176.62, 144.60, 142.24, 140.97, 128.83, 128.50, 128.20, 128.09, 127.87, 127.24, 127.08,

127.03, 126.57, 65.61, 57.07, 38.64, 36.81, 36.78, 35.66, 28.19. **HRMS–ESI** (m/z): $[M+H]^+$ calcd. for $C_{32}H_{34}NO$, 448.2635; found, 448.2634.

1,3,3,5-tetraphenylpyrrolidin-2-one (3f)

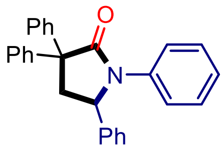

White solid. **^{1}H NMR** (400 MHz, $CDCl_3$) δ 7.44 (d, J = 7.4 Hz, 2H), 7.39 (d, J = 7.0 Hz, 2H), 7.37 – 7.30 (m, 6H), 7.29 – 7.24 (m, 2H), 7.24 – 7.15 (m, 7H), 7.07 – 7.02 (m, 1H), 5.10 (dd, J = 9.5, 5.9 Hz, 1H), 3.03 (ddd, J = 22.5, 13.0, 7.7 Hz, 2H). **^{13}C NMR** (100 MHz, $CDCl_3$) δ 175.09, 143.23, 142.04, 140.09, 137.81, 128.79, 128.74, 128.49, 128.21, 128.08, 127.83, 127.71, 127.22, 126.91, 126.81, 125.26, 123.35, 60.27, 58.47, 45.54. **HRMS–ESI** (m/z): $[M+H]^+$ calcd. for $C_{28}H_{24}NO$, 390.1852; found, 390.1855.

5-(((tert-butyldimethylsilyl)oxy)methyl)-1,3,3-triphenylpyrrolidin-2-one (3g)

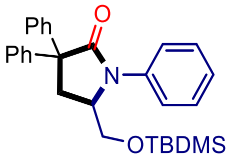

Colorless oil. **^{1}H NMR** (400 MHz, $CDCl_3$) δ 7.44 – 7.40 (m, 3H), 7.40 – 7.35 (m, 5H), 7.35 – 7.29 (m, 4H), 7.29 – 7.19 (m, 4H), 4.22 – 4.11 (m, 1H), 3.63 (ddd, J = 13.4, 10.7, 3.6 Hz, 2H), 3.01 – 2.84 (m, 2H), 0.82 (s, 9H), -0.12 (s, 3H), -0.20 (s, 3H). **^{13}C NMR** (100 MHz, $CDCl_3$) δ 175.10, 144.39, 142.30, 137.40, 128.79, 128.51, 128.22, 128.17, 127.87, 127.04, 126.67, 126.20, 124.85, 61.38, 58.10, 57.29, 37.10, 25.75, 18.13, -5.77, -5.90. **HRMS–ESI** (m/z): $[M+H]^+$ calcd. for $C_{29}H_{36}NO_2Si$, 458.2510; found, 458.2496.

5-(methoxymethyl)-1,3,3-triphenylpyrrolidin-2-one (3h)

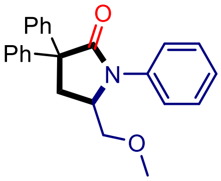

Colorless oil. **^{1}H NMR** (400 MHz, $CDCl_3$) δ 7.46 (dd, J = 8.4, 1.2 Hz, 2H), 7.41 – 7.33 (m, 6H), 7.33 – 7.27 (m, 4H), 7.26 – 7.20 (m, 3H), 4.18 (tdd, J = 9.4, 6.1, 3.2 Hz, 1H), 3.36 (ddd, J = 15.8, 9.8, 4.6 Hz, 2H), 3.18 (s, 3H), 2.95 (ddd, J = 21.3, 13.0, 7.3 Hz, 2H). **^{13}C NMR** (100 MHz, $CDCl_3$) δ 174.86, 143.76, 142.45, 137.42, 128.90, 128.48, 128.16, 128.06, 127.68, 126.97, 126.72, 126.35, 124.84, 71.98, 59.08, 57.73, 55.75, 37.90. **HRMS–ESI** (m/z): $[M+H]^+$ calcd. for $C_{24}H_{24}NO_2$, 358.1802; found, 358.1805.

5-(diethoxymethyl)-1,3,3-triphenylpyrrolidin-2-one (3i)

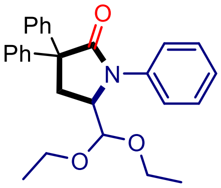

Colorless oil. **^{1}H NMR** (400 MHz, $CDCl_3$) δ 7.48 (dd, J = 5.3, 3.3 Hz, 2H), 7.37 (dd, J = 6.9, 3.1 Hz, 6H), 7.34 – 7.26 (m, 4H), 7.25 – 7.19 (m, 3H), 4.40 (d, J = 4.0 Hz, 1H), 4.26 (ddd, J = 7.7, 6.8, 4.0 Hz, 1H), 3.57 – 3.51 (m, 2H), 3.22 (ddq, J = 48.9, 9.3, 7.1 Hz, 2H), 3.00 (ddd, J = 20.1, 13.3, 7.2 Hz, 2H), 1.07 (t, J = 7.0 Hz, 3H), 0.97 (t, J = 7.0 Hz, 3H). **^{13}C NMR** (100 MHz, $CDCl_3$) δ 175.08, 143.88, 142.95, 138.02, 128.76, 128.42, 128.17, 128.11, 127.72, 126.87, 126.67, 126.13, 124.72, 101.70, 64.82, 64.14, 58.49, 57.54, 34.48, 15.22, 15.00. **HRMS–ESI** (m/z): $[M+H]^+$ calcd. for $C_{27}H_{30}NO_3$, 416.2220; found, 416.2230.

2-(5-oxo-1,4,4-triphenylpyrrolidin-2-yl)acetonitrile (3j)

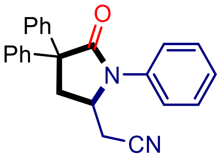

White solid. ^{1}**H NMR** (400 MHz, $CDCl_3$) δ 7.47 – 7.40 (m, 4H), 7.39 – 7.35 (m, 4H), 7.33 (dd, J = 6.0, 2.0 Hz, 3H), 7.31 – 7.26 (m, 4H), 4.31 (tdd, J = 8.1, 6.1, 3.4 Hz, 1H), 3.05 (ddd, J = 21.3, 13.1, 7.1 Hz, 2H), 2.58 (ddd, J = 24.4, 16.9, 5.4 Hz, 2H). **NMR** (100 MHz, $CDCl_3$) δ 174.42, 142.73, 141.14, 136.10, 129.55, 128.79, 128.54, 127.92, 127.61, 127.49, 127.30, 127.25, 124.93, 115.82, 57.82, 52.35, 40.08, 22.37. **HRMS–ESI** (m/z): $[M+H]^+$ calcd. for $C_{24}H_{21}N_2O$, 353.1648; found, 353.1642.

5-(cyclohex-1-en-1-ylmethyl)-1,3,3-triphenylpyrrolidin-2-one (3k)

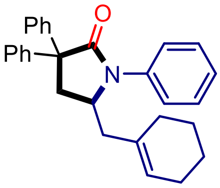

White solid. ^{1}**H NMR** (400 MHz, $CDCl_3$) δ 7.46 – 7.42 (m, 2H), 7.39 – 7.34 (m, 5H), 7.33 – 7.26 (m, 4H), 7.25 – 7.15 (m, 3H), 5.37 (dt, J = 4.4, 2.0 Hz, 1H), 4.21 (dtd, J = 8.0, 4.0, 2.0 Hz, 1H), 3.08 – 2.48 (m, 2H), 2.45 – 2.37 (m, 1H), 1.95 (dp, J = 5.9, 2.8 Hz, 2H), 1.89 – 1.74 (m, 3H), 1.63 – 1.45 (m, 4H). ^{13}**C NMR** (100 MHz, $CDCl_3$) δ 174.55, 144.10, 142.34, 137.53, 132.80, 128.76, 128.40, 128.12, 128.01, 127.62, 126.87, 126.63, 125.97, 124.75, 124.64, 57.79, 54.37, 42.37, 40.77, 28.70, 25.18, 22.72, 22.15. **HRMS–ESI** (m/z): $[M+H]^+$ calcd. for $C_{29}H_{30}NO$, 408.2322; found, 408.2327.

tert-butyl 3-5-oxo-1,4,4-triphenylpyrrolidin-2-yl)piperidine-1-carboxylate (3l)

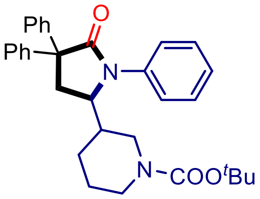

White solid. ^{1}**H NMR** (400 MHz, $CDCl_3$) δ 7.42 – 7.36 (m, 4H), 7.36 – 7.28 (m, 8H), 7.28 – 7.20 (m, 3H), 4.15 (dq, J = 8.5, 4.2, 3.2 Hz, 1H), 3.98 (d, J = 11.5 Hz, 2H), 2.81 (dd, J = 12.8, 6.0 Hz, 1H), 2.64 (t, J = 11.2 Hz, 1H), 2.52 (s, 1H), 2.50 – 2.39 (m, 1H), 1.91 (s, 1H), 1.65 – 1.51 (m, 2H), 1.42 (s, 9H), 1.15 (td, J = 12.1, 10.7, 8.3 Hz, 2H). ^{13}**C NMR** (100 MHz, $CDCl_3$) δ 174.92, 154.74, 144.03, 137.31, 128.90, 128.58, 128.25, 127.95, 127.62, 127.20, 126.78, 126.33, 124.78, 79.65, 57.59, 57.03, 35.40, 32.68, 28.40, 26.40, 26.30, 24.70, 22.86. **HRMS–ESI** (m/z): $[M+H]^+$ calcd. for $C_{32}H_{37}N_2O_3$, 497.2799; found, 497.2818.

1-(4-(tert-butyl)phenyl)-5-ethyl-3,3-diphenylpyrrolidin-2-one (3m)

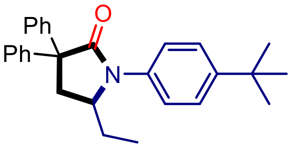

Colorless oil. ^{1}**H NMR** (400 MHz, $CDCl_3$) δ 7.44 (dt, J = 3.2, 1.9 Hz, 2H), 7.41 – 7.35 (m, 4H), 7.35 – 7.27 (m, 4H), 7.26 – 7.20 (m, 4H), 3.98 (tdd, J = 9.0, 5.8, 3.2 Hz, 1H), 2.76 (ddd, J = 21.9, 12.8, 7.4 Hz, 2H), 1.84 – 1.76 (m, 1H), 1.41 – 1.22 (m, 10H), 0.86 (t, J = 7.5 Hz, 3H). ^{13}**C NMR** (100 MHz, $CDCl_3$) δ 174.74, 148.98, 144.17, 142.39, 134.82, 128.48, 128.16, 128.07, 127.73, 126.95, 126.66, 125.73, 124.30, 57.82, 56.71, 40.27, 34.49, 31.34, 26.06, 8.60. **HRMS–ESI** (m/z): $[M+H]^+$ calcd. for $C_{28}H_{32}NO$, 398.2478; found, 398.2498.

1-([1,1'-biphenyl]-4-yl)-5-ethyl-3,3-diphenylpyrrolidin-2-one (3n)

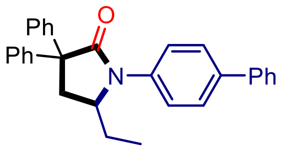

White solid. **^{1}H NMR** (400 MHz, $CDCl_3$) δ 7.60 (d, J = 8.6 Hz, 2H), 7.58 – 7.55 (m, 2H), 7.44 (ddd, J = 10.0, 5.3, 3.5 Hz, 3H), 7.41 – 7.36 (m, 5H), 7.35 – 7.28 (m, 5H), 7.27 – 7.21 (m, 2H), 4.04 (tdd, J = 9.0, 5.8, 3.1 Hz, 1H), 2.80 (ddd, J = 21.9, 12.8, 7.4 Hz, 2H), 1.90 – 1.80 (m, 1H), 1.46 – 1.32 (m, 1H), 0.88 (t, J = 7.5 Hz, 3H). **NMR** (100 MHz, $CDCl_3$) δ 174.82, 144.05, 142.21, 140.53, 138.97, 136.75, 128.75, 128.53, 128.20, 128.05, 127.70, 127.52, 127.27, 127.03, 126.73, 124.89, 57.89, 56.59, 40.13, 25.90, 8.54. **HRMS–ESI** (m/z): $[M+H]^+$ calcd. for $C_{30}H_{28}NO$, 418.2165; found, 418.2168.

methyl-4-(5-methyl-2-oxo-3,3-diphenylpyrrolidin-1-yl)benzoate (3o)

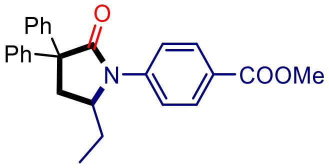

White solid. **^{1}H NMR** (400 MHz, $CDCl_3$) δ 8.06 (d, J = 8.7 Hz, 2H), 7.47 – 7.44 (m, 2H), 7.43 – 7.40 (m, 2H), 7.37 – 7.28 (m, 6H), 7.28 – 7.23 (m, 3H), 4.09 (tdd, J = 8.9, 5.9, 3.0 Hz, 1H), 3.91 (s, 3H), 2.81 (ddd, J = 21.8, 12.9, 7.5 Hz, 2H), 1.88 – 1.77 (m, 1H), 1.44 – 1.31 (m, 1H), 0.86 (t, J = 7.5 Hz, 3H). **^{13}C NMR** (100 MHz, $CDCl_3$) δ 174.88, 166.54, 143.75, 141.96, 141.81, 130.31, 128.63, 128.29, 128.01, 127.61, 127.23, 127.19, 126.90, 123.62, 58.00, 56.28, 52.10, 39.77, 25.62, 8.45. **HRMS–ESI** (m/z): $[M+H]^+$ calcd. for $C_{26}H_{26}NO_3$, 400.1907; found, 400.1924.

1-(4-iodophenyl)-5-methyl-3,3-diphenylpyrrolidin-2-one (3p)

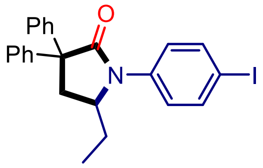

Colorless oil. **^{1}H NMR** (400 MHz, $CDCl_3$) δ 7.71 – 7.68 (m, 2H), 7.44 – 7.38 (m, 2H), 7.36 – 7.27 (m, 6H), 7.25 (dd, J = 6.5, 2.2 Hz, 2H), 7.13 – 7.06 (m, 2H), 3.98 (tdd, J = 9.0, 5.8, 3.2 Hz, 1H), 2.77 (ddd, J = 21.9, 12.8, 7.4 Hz, 2H), 1.88 – 1.72 (m, 1H), 1.45 – 1.26 (m, 1H), 0.85 (t, J = 7.5 Hz, 3H). **^{13}C NMR** (100 MHz, $CDCl_3$) δ 174.74, 143.78, 141.97, 137.91, 137.33, 128.59, 128.24, 128.00, 127.62, 127.13, 126.83, 126.39, 90.38, 57.89, 56.33, 39.96, 39.96, 25.69, 8.45. **HRMS–ESI** (m/z): $[M+H]^+$ calcd. for $C_{24}H_{23}NOI$, 468.0819; found, 468.0835.

1-(4-fluorophenyl)-5-methyl-3,3-diphenylpyrrolidin-2-one (3q)

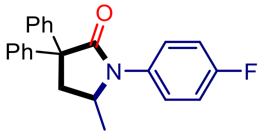

Colorless oil. **^{1}H NMR** (400 MHz, $CDCl_3$) δ 7.42 (d, J = 7.3 Hz, 2H), 7.37 – 7.33 (m, 2H), 7.32 – 7.29 (m, 3H), 7.29 – 7.21 (m, 5H), 7.11 – 6.99 (m, 2H), 4.07 (dt, J = 9.0, 5.9 Hz, 1H), 3.08 (dd, J = 12.8, 5.6 Hz, 1H), 2.48 (dd, J = 12.8, 9.0 Hz, 1H), 1.19 (d, J = 6.1 Hz, 3H). ^{13}C NMR (100 MHz, $CDCl_3$) δ 174.68, 160.67 (d, J = 245.7 Hz), 142.84 (d, J = 177.5 Hz), 133.36 (d, J = 3.0 Hz), 128.55, 128.18, 127.98, 127.61, 127.06, 126.76, 126.54, 126.45, 115.72 (d, J = 22.6 Hz), 57.98, 51.72, 43.25, 19.82. **^{19}F NMR** (376 MHz, $CDCl_3$) δ -115.74. **HRMS–ESI** (m/z): $[M+H]^+$ calcd. for $C_{23}H_{21}NOF$, 346.1602; found, 346.1588.

1-(4-chlorophenyl)-5-methyl-3,3-diphenylpyrrolidin-2-one (3r)

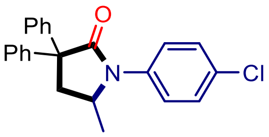

Colorless oil. **^{1}H NMR** (400 MHz, $CDCl_3$) δ 7.43 – 7.40 (m, 2H), 7.38 – 7.34 (m, 3H), 7.34 – 7.28 (m, 6H), 7.28 – 7.22 (m, 3H), 4.15 – 4.04 (m, 1H), 2.79 (ddd, J = 21.8, 12.8, 7.3 Hz, 2H), 1.21 (d, J = 6.1 Hz, 3H). **^{13}C NMR** (100 MHz, $CDCl_3$) δ 174.63, 143.62, 141.92, 135.98, 131.44, 129.01, 128.61, 128.24, 128.01, 127.62, 127.14, 126.85, 125.73, 58.09, 51.41, 43.14, 19.80. **HRMS–ESI** (m/z): $[M+H]^+$ calcd. for $C_{23}H_{21}NOCl$, 362.1306; found, 362.1311.

1-(4-bromophenyl)-5-methyl-3,3-diphenylpyrrolidin-2-one (3s)

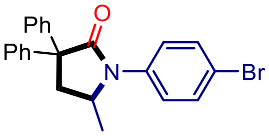

Yellow oil. **^{1}H NMR** (400 MHz, $CDCl_3$) δ 7.53 – 7.48 (m, 2H), 7.41 (dd, J = 8.2, 1.1 Hz, 2H), 7.37 – 7.27 (m, 6H), 7.27 – 7.19 (m, 4H), 4.10 (dt, J = 9.0, 5.9 Hz, 1H), 2.79 (ddd, J = 21.8, 12.8, 7.3 Hz, 2H), 1.21 (d, J = 6.1 Hz, 3H). **^{13}C NMR** (100 MHz, $CDCl_3$) δ 174.56, 143.58, 141.88, 136.48, 131.94, 128.59, 128.22, 127.99, 127.59, 127.12, 126.83, 125.99, 119.22, 58.08, 51.31, 43.08, 19.76. **HRMS–ESI** (m/z): $[M+H]^+$ calcd. for $C_{23}H_{21}NOBr$, 406.0801; found, 406.0784.

5-methyl-3,3-diphenyl-1-(4-(trifluoromethoxy)phenyl)pyrrolidin-2-one (3t)

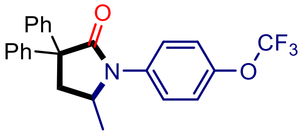

Colorless oil. **^{1}H NMR** (400 MHz, $CDCl_3$) δ 7.45 – 7.40 (m, 2H), 7.39 – 7.32 (m, 6H), 7.30 (t, J = 5.0 Hz, 2H), 7.27 – 7.21 (m, 4H), 4.13 (dp, J = 9.0, 6.0 Hz, 1H), 2.80 (ddd, J = 21.8, 12.8, 7.3 Hz, 2H), 1.23 (d, J = 6.1 Hz, 3H). **^{13}C NMR** (100 MHz, $CDCl_3$) δ 174.73, 146.72 (d, J = 1.8 Hz), 143.60, 141.85, 136.01, 128.62, 128.25, 128.00, 127.59, 127.16, 126.87, 125.68, 121.49, 58.07, 51.47, 43.12, 19.81. **^{19}F NMR** (376 MHz, $CDCl_3$) δ -57.96. **HRMS–ESI** (m/z): $[M+H]^+$ calcd. for $C_{24}H_{24}NO$, 412.1519; found, 412.1521.

5-methyl-3,3-diphenyl-1-(p-tolyl)pyrrolidin-2-one (3u)

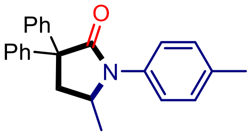

Colorless oil. **^{1}H NMR** (400 MHz, $CDCl_3$) δ 7.45 – 7.42 (m, 2H), 7.38 – 7.34 (m, 2H), 7.33 – 7.25 (m, 4H), 7.25 – 7.19 (m, 2H), 7.18 (s, 4H), 4.13 – 3.99 (m, 1H), 2.76 (ddd, J = 21.8, 12.7, 7.3 Hz, 2H), 2.33 (s, 3H), 1.18 (d, J = 6.1 Hz, 3H). **^{13}C NMR** (100 MHz, $CDCl_3$) δ 174.46, 143.90, 142.22, 135.79, 134.76, 129.42, 128.45, 128.10, 128.02, 127.66, 126.91, 126.63, 124.60, 58.00, 51.48, 43.26, 20.96, 19.86. **HRMS–ESI** (m/z): $[M+H]^+$ calcd. for $C_{24}H_{24}NO$, 342.1852; found, 342.1846.

5-methyl-3,3-diphenyl-1-(m-tolyl)pyrrolidin-2-one (3v)

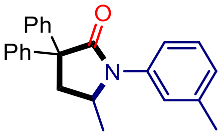

Colorless oil. **^{1}H NMR** (400 MHz, $CDCl_3$) δ 7.46 – 7.41 (m, 2H), 7.37 (dd, J = 5.4, 3.4 Hz, 2H), 7.34 – 7.28 (m, 4H), 7.24 (dt, J = 7.4, 5.0 Hz, 3H), 7.19 (s, 1H), 7.05 (t, J = 8.0 Hz, 2H), 4.15 – 4.07 (m, 1H), 2.78 (ddd, J = 21.8, 12.7, 7.3 Hz, 2H), 2.36 (s, 3H), 1.21 (d, J = 6.1 Hz, 3H). **^{13}C NMR** (100 MHz, $CDCl_3$)

δ 174.53, 143.92, 142.26, 138.78, 137.32, 128.63, 128.53, 128.17, 128.08, 127.72, 126.99, 126.93, 126.72, 125.65, 121.47, 58.10, 51.52, 43.28, 21.42, 19.96. **HRMS–ESI** (m/z): $[M+H]^+$ calcd. for $C_{24}H_{24}NO$, 342.1852; found, 342.1860.

1-(3-methoxyphenyl)-5-methyl-3,3-diphenylpyrrolidin-2-one (3w)

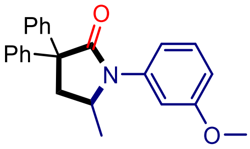

White solid. **^{1}H NMR** (400 MHz, $CDCl_3$) δ 7.43 (dt, J = 3.2, 1.9 Hz, 2H), 7.37 – 7.31 (m, 4H), 7.31 – 7.24 (m, 4H), 7.24 – 7.20 (m, 1H), 6.95 (t, J = 2.2 Hz, 1H), 6.85 (ddd, J = 7.9, 1.8, 0.8 Hz, 1H), 6.77 (ddd, J = 8.3, 2.5, 0.7 Hz, 1H), 3.97 (tdd, J = 9.0, 5.8, 3.1 Hz, 1H), 3.78 (s, 3H), 2.77 (ddd, J = 21.9, 12.8, 7.4 Hz, 2H), 1.86 – 1.75 (m, 1H), 1.42 – 1.29 (m, 1H), 0.85 (t, J = 7.5 Hz, 3H). **NMR** (100 MHz, $CDCl_3$) δ 174.67, 159.99, 143.91, 142.29, 138.73, 129.39, 128.49, 128.15, 128.03, 127.65, 126.96, 126.69, 116.64, 111.83, 110.86, 57.89, 56.68, 55.34, 40.00, 25.83, 8.53. **HRMS–ESI** (m/z): $[M+H]^+$ calcd. for $C_{25}H_{26}NO_2$, 372.1958; found, 372.1946.

1-(3,5-dichlorophenyl)-5-methyl-3,3-diphenylpyrrolidin-2-one (3x)

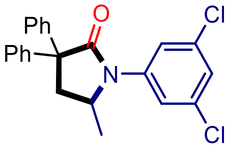

Colorless oil. **^{1}H NMR** (400 MHz, $CDCl_3$) δ 7.41 – 7.37 (m, 2H), 7.36 – 7.28 (m, 8H), 7.28 – 7.23 (m, 2H), 7.21 (t, J = 1.8 Hz, 1H), 4.15 – 4.08 (m, 1H), 2.80 (ddd, J = 21.7, 12.9, 7.3 Hz, 2H), 1.25 (d, J = 6.1 Hz, 3H). **^{13}C NMR** (100 MHz, $CDCl_3$) δ 174.74, 143.28, 141.54, 139.37, 135.12, 128.70, 128.31, 127.94, 127.51, 127.29, 126.99, 125.85, 122.41, 58.12, 51.32, 42.83, 19.74. **HRMS–ESI** (m/z): $[M+H]^+$ calcd. for $C_{23}H_{20}NOCl_2$, 396.0916; found, 396.0914.

5-methyl-3,3-diphenyl-1-(pyridin-2-yl)pyrrolidin-2-one (3y)

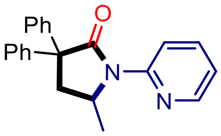

Colorless oil. **^{1}H NMR** (400 MHz, $CDCl_3$) δ 8.39 (ddd, J = 4.9, 1.9, 0.8 Hz, 1H), 8.14 (dt, J = 8.4, 0.9 Hz, 1H), 7.71 (ddd, J = 8.4, 7.4, 2.0 Hz, 1H), 7.51 – 7.42 (m, 2H), 7.34 (ddd, J = 6.2, 2.5, 0.7 Hz, 3H), 7.31 – 7.28 (m, 2H), 7.28 – 7.23 (m, 3H), 7.23 – 7.18 (m, 1H), 7.06 (ddd, J = 7.3, 4.9, 1.0 Hz, 1H), 4.67 (dd, J = 13.5, 6.6 Hz, 1H), 2.83 (ddd, J = 232.3, 13.0, 7.1 Hz, 2H), 1.31 (d, J = 6.1 Hz, 3H). **^{13}C NMR** (100 MHz, $CDCl_3$) δ 175.27, 151.17, 147.69, 143.94, 142.48, 137.40, 128.44, 128.30, 128.07, 127.69, 126.96, 126.83, 120.10, 118.16, 58.73, 50.87, 42.12, 20.31. **HRMS–ESI** (m/z): $[M+H]^+$ calcd. for $C_{22}H_{21}N_2O$, 329.1648; found, 329.1656.

3,3,5-triphenyl-1-(pyridin-2-yl)pyrrolidin-2-one (3z)

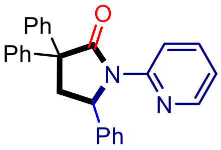

White solid. **^{1}H NMR** (400 MHz, $CDCl_3$) δ 8.15 – 8.06 (m, 2H), 7.67 (ddd, J = 8.6, 7.4, 1.9 Hz, 1H), 7.44 – 7.40 (m, 2H), 7.39 – 7.33 (m, 3H), 7.33 – 7.26 (m, 4H), 7.23 (t, J = 4.7 Hz, 1H), 7.19 (d, J = 4.4 Hz, 4H), 7.18 – 7.10 (m, 1H), 6.93 (ddd, J = 7.4, 5.0, 0.9 Hz, 1H), 5.60 (dd, J = 8.9, 6.6 Hz, 1H), 3.05 (ddd, J = 22.2, 13.2, 7.7 Hz, 2H). **^{13}C NMR** (100 MHz, $CDCl_3$) δ 175.74, 150.90, 147.69, 143.08, 142.20, 141.85, 137.26, 128.69, 128.42, 128.22, 128.09, 127.64, 127.19, 127.09, 126.92, 126.24, 120.14, 118.19, 59.03, 58.92, 44.54. **HRMS–ESI** (m/z): $[M+H]^+$ calcd. for $C_{27}H_{23}N_2O$, 391.1805; found, 391.1806.

1,3,3-triphenylpyrrolidin-2-one (3aa)

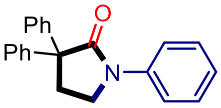

Colorless oil. **^{1}H NMR** (400 MHz, $CDCl_3$) *δ* 7.68 (dd, J = 8.7, 1.0 Hz, 2H), 7.39 (dd, J = 5.3, 3.3 Hz, 6H), 7.36 – 7.29 (m, 4H), 7.28 – 7.25 (m, 3H), 7.17 (dt, J = 14.8, 4.7 Hz, 1H), 3.80 (t, J = 6.4 Hz, 2H), 2.89 (t, J = 6.4 Hz, 2H). **^{13}C NMR** (100 MHz, $CDCl_3$) *δ* 174.36, 142.48, 139.63, 128.85, 128.74, 128.40, 127.93, 127.73, 127.02, 126.70, 124.68, 119.96, 58.94, 45.22, 34.12. **HRMS–ESI** (m/z): $[M+H]^+$ calcd. for $C_{22}H_{20}NO$, 314.1539; found, 314.1531.

3,3,5,5-tetraphenyl-1-(pyridin-2-yl)azepan-2-one (3ab)

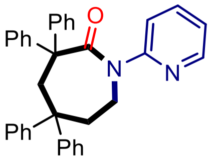

White solid. **^{1}H NMR** (400 MHz, $CDCl_3$) *δ* 8.41 (ddd, J = 4.9, 1.9, 0.8 Hz, 1H), 8.25 – 8.17 (m, 1H), 7.69 (ddd, J = 8.4, 7.4, 2.0 Hz, 1H), 7.43 – 7.37 (m, 3H), 7.37 – 7.31 (m, 5H), 7.28 (dt, J = 4.6, 1.9 Hz, 1H), 7.24 – 7.14 (m, 7H), 7.12 (dd, J = 5.0, 3.6 Hz, 1H), 7.06 (ddd, J = 7.3, 4.9, 0.9 Hz, 1H), 7.00 (dd, J = 5.2, 3.3 Hz, 2H), 4.69 – 4.55 (m, 1H), 4.08 (dd, J = 10.5, 5.6 Hz, 1H), 2.96 (dd, J = 13.3, 7.2 Hz, 1H), 2.88 (ddd, J = 13.4, 10.5, 3.0 Hz, 1H), 2.59 (dd, J = 13.3, 5.9 Hz, 1H), 1.77 (ddd, J = 13.4, 10.2, 5.6 Hz, 1H). **^{13}C NMR** (100 MHz, $CDCl_3$) *δ* 175.18, 151.16, 147.67, 145.08, 144.27, 142.57, 142.31, 137.50, 128.56, 128.51, 128.40, 128.32, 128.29, 128.03, 127.74, 127.61, 126.94, 126.91, 126.59, 126.22, 120.05, 117.51, 58.81, 54.13, 48.23, 39.16, 38.87. **HRMS–ESI** (m/z): $[M+H]^+$ calcd. for $C_{35}H_{31}N_2O$, 495.2431; found, 495.2411.

4-(2-oxo-3,3-diphenyl-5-propylpyrrolidin-1-yl)benzoic acid (3ac)

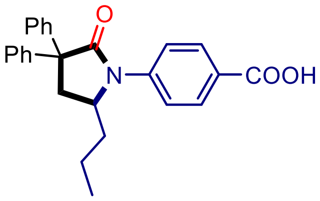

Yellow solid. **^{1}H NMR** (400 MHz, $CDCl_3$) *δ* 7.80 (dd, J = 256.1, 8.7 Hz, 4H), 7.41 (d, J = 7.3 Hz, 2H), 7.37 – 7.28 (m, 6H), 7.28 – 7.23 (m, 2H), 4.14 (dtd, J = 8.7, 5.8, 3.0 Hz, 1H), 3.10 (dd, J = 12.9, 5.9 Hz, 1H), 2.56 (dd, J = 12.9, 8.8 Hz, 1H), 1.85 – 1.70 (m, 1H), 1.48 – 1.17 (m, 3H), 0.86 (t, J = 7.1 Hz, 3H). **^{13}C NMR** (100 MHz, $CDCl_3$) *δ* 174.90, 171.42, 143.60, 142.52, 141.78, 130.94, 128.60, 128.26, 127.96, 127.55, 127.18, 126.88, 126.34, 123.65, 77.32, 77.00, 76.68, 58.05, 55.28, 40.53, 35.21, 17.77, 13.96. **HRMS–ESI** (m/z): $[M+H]^+$ calcd. for $C_{26}H_{25}NO_3$, 400.1907; found, 400.1924.

3,3-bis(4-fluorophenyl)-5-methyl-1-phenylpyrrolidin-2-one (4a)

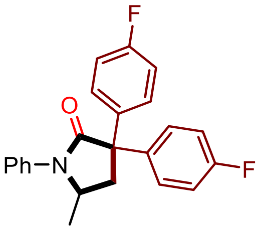

Colorless oil. **^{1}H NMR** (400 MHz, $CDCl_3$) δ 7.45 – 7.36 (m, 4H), 7.34 – 7.28 (m, 4H), 7.26 – 7.21 (m, 1H), 7.05 – 6.92 (m, 4H), 4.12 (dt, J = 9.0, 5.9 Hz, 1H), 3.03 (dd, J = 12.8, 5.6 Hz, 1H), 2.44 (dd, J = 12.8, 9.0 Hz, 1H), 1.21 (d, J = 6.1 Hz, 3H). **^{13}C NMR** (100 MHz, $CDCl_3$) δ 174.22, 162.98 (d, J = 13.3 Hz), 160.53 (d, J = 12.8 Hz), 139.47 (d, J = 3.2 Hz), 137.69 (d, J = 3.3 Hz), 137.14, 129.58 (d, J = 8.0 Hz), 129.23 (d, J = 8.0 Hz), 128.92, 126.28, 124.65, 115.44 (d, J = 21.3 Hz), 115.03 (d, J = 21.3 Hz),

56.93, 51.47, 43.33, 19.85. **^{19}F NMR** (376 MHz, $CDCl_3$) δ -115.38, -115.97. **HRMS–ESI** (m/z): $[M+H]^+$ calcd. for $C_{23}H_{20}NOF_2$, 364.1508; found, 364.1508.

5-methyl-1,3-diphenyl-3-(o-tolyl)pyrrolidin-2-one (4b)

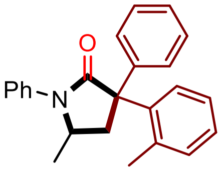

Colorless oil. **mixture of diastereomers and rotamers. ^{1}H NMR** (400 MHz, $CDCl_3$) δ 7.52 – 7.48 (m, 1H), 7.43 – 7.39 (m, 3H), 7.39 – 7.35 (m, 4H), 7.35 – 7.28 (m, 9H), 7.25 (d, J = 5.1 Hz, 6H), 7.22 – 7.16 (m, 5H), 7.14 (d, J = 7.0 Hz, 4H), 4.48 – 4.34 (m, 1H), 4.07 (dt, J = 8.8, 6.1 Hz, 1.3H), 3.43 (dd, J = 13.6, 6.8 Hz, 1H), 3.05 – 2.70 (m, 2.6H), 2.30 (dd, J = 13.5, 8.1 Hz, 1H), 2.06 (s, 3.9H), 1.91 (s, 3H), 1.26 (d, J = 6.1 Hz, 3.6 H), 1.23 (d, J = 6.1 Hz, 3.0 H). **^{13}C NMR** (100 MHz, $CDCl_3$) δ 174.77, 174.42, 143.46, 141.72, 141.56, 140.91, 137.66, 137.36, 136.92, 136.67, 132.94, 132.53, 128.86, 128.82, 128.46, 128.11, 127.98, 127.89, 127.59, 127.32, 127.01, 126.98, 126.74, 126.02, 126.00, 125.75, 125.70, 124.62, 124.57, 59.45, 58.57, 52.21, 51.19, 41.24, 40.28, 21.60, 21.33, 20.64, 19.88.

5-3-(4-methoxyphenyl)-5-methyl-1,3-diphenylpyrrolidin-2-one (4c)

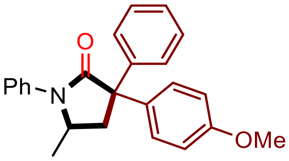

White solid. **mixture of diastereomers and rotamers. ^{1}H NMR** (400 MHz, $CDCl_3$) δ 7.46 – 7.39 (m, 2H), 7.39 – 7.34 (m, 6H), 7.34 – 7.26 (m, 11H), 7.26 – 7.18 (m, 5H), 6.91 – 6.75 (m, 4H), 4.12 (tt, J = 8.7, 5.9 Hz, 2H), 3.78 (s, 3H), 3.77 (s, 3H), 3.04 (td, J = 12.7, 5.7 Hz, 2H), 2.46 (ddd, J = 18.3, 12.7, 9.0 Hz, 2H), 1.21 (dd, J = 6.1, 1.2 Hz, 6H). **^{13}C NMR** (100 MHz, $CDCl_3$) δ 174.77, 174.75, 158.51, 158.31, 144.28, 142.65, 137.48, 137.43, 135.87, 133.86, 129.11, 128.80, 128.78, 128.61, 128.52, 128.47, 128.13, 127.96, 127.56, 126.88, 126.61, 125.97, 124.65, 124.61, 113.86, 113.54, 76.68, 57.47, 57.42, 55.21, 55.20, 51.50, 51.38, 43.41, 43.27, 19.90, 19.85. **HRMS–ESI** (m/z): $[M+H]^+$ calcd. for $C_{24}H_{24}NO_2$, 358.1802; found, 358.1813.

5-3-(4-fluorophenyl)-5-methyl-1-phenyl-3-(4-(phenylethynyl)phenyl)pyrrolidin-2-one (4d)

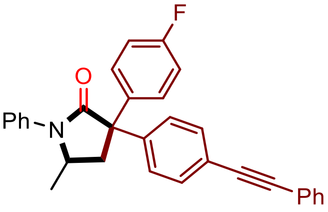

White solid. **mixture of diastereomers and rotamers. ^{1}H NMR** (400 MHz, $CDCl_3$) δ 7.50 (dddd, J = 10.7, 8.6, 5.6, 2.4 Hz, 8H), 7.40 (ddd, J = 8.9, 4.6, 2.8 Hz, 8H), 7.33 (qd, J = 7.0, 4.6 Hz, 14H), 7.28 – 7.21 (m, 2H), 7.01 (q, J = 8.5 Hz, 4H), 4.14 (s, 2H), 3.06 (ddd, J = 12.8, 5.7, 3.8 Hz, 2H), 2.47 (dd, J = 12.8, 8.9 Hz, 2H), 1.22 (d, J = 6.1 Hz, 6H). **^{13}C NMR** (100 MHz, $CDCl_3$) δ 174.03, 174.02, 163.11, 162.99, 160.66, 160.55, 143.99, 142.19, 139.10, 139.06, 137.49, 137.45, 137.20, 137.18, 131.85, 131.61, 131.52, 129.69 (d, J = 8.0 Hz), 129.35 (d, J = 8.0 Hz), 128.95, 128.33, 128.31, 128.23, 127.97, 127.61, 126.31, 126.28, 124.72, 124.66, 123.21 (d, J = 12.9 Hz), 122.10 (d, J = 37.0 Hz), 115.47 (d, J = 21.3 Hz), 115.07 (d, J = 21.3 Hz), 89.73 (d, J = 31.1 Hz), 57.50, 57.43, 51.60, 51.50, 43.14, 43.10, 19.91, 19.89. **^{19}F NMR** (376 MHz, $CDCl_3$) δ -115.35, -115.92.**HRMS–ESI** (m/z): $[M+H]^+$ calcd. for $C_{31}H_{25}NOF$, 446.1915; found, 446.1902.

4-(5-methyl-2-oxo-1,4-diphenylpyrrolidin-3-yl)benzonitrile (4e)

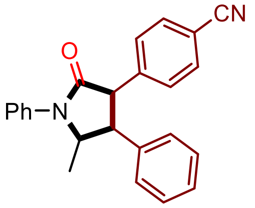

White soild. **Isomer 1**. **^{1}H NMR** (400 MHz, $CDCl_3$) δ 7.60 (dd, J = 9.7, 8.0 Hz, 4H), 7.47 – 7.39 (m, 4H), 7.37 (td, J = 7.6, 7.0, 1.1 Hz, 2H), 7.32 – 7.26 (m, 3H), 7.25 – 7.18 (m, 1H), 4.68 – 4.58 (m, 1H), 4.40 (d, J = 12.2 Hz, 1H), 4.05 (dd, J = 12.2, 7.7 Hz, 1H), 0.96 (d, J = 6.6 Hz, 3H). **^{13}C NMR** (100 MHz, $CDCl_3$) δ 171.48, 142.60, 137.73, 135.92, 132.32, 129.60, 129.22, 128.94, 128.16, 125.79, 122.65, 118.67, 111.27, 58.13, 50.84, 50.34, 14.94. **Isomer 2**. **^{1}H NMR** (400 MHz, $CDCl_3$) δ 7.58 (d, J = 8.3 Hz, 2H), 7.44 (dd, J = 8.4, 7.2 Hz, 2H), 7.40 – 7.33 (m, 4H), 7.33 – 7.22 (m, 6H), 4.32 (dq, J = 8.9, 6.0 Hz, 1H), 4.09 (d, J = 11.6 Hz, 1H), 3.08 (dd, J = 11.6, 8.9 Hz, 1H), 1.22 (d, J = 6.0 Hz, 3H). **^{13}C NMR** (100 MHz, $CDCl_3$) δ 171.49, 143.20, 137.81, 136.89, 132.35, 129.35, 129.15, 129.05, 128.01, 127.95, 126.54, 124.87, 118.71, 111.19, 60.15, 57.21, 56.26, 18.44. **HRMS–ESI** (m/z): $[M+H]^+$ calcd. for $C_{24}H_{21}N_2O$, 353.1649; found, 353.1657.

5-methyl-1-phenyl-3-(4-(trifluoromethyl)phenyl)pyrrolidin-2-one (4f)

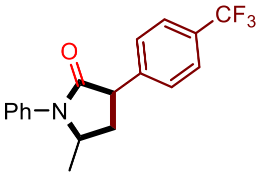

Colorless oil. **Isomer 1. ^{1}H NMR** (400 MHz, $CDCl_3$) δ 7.62 – 7.58 (m, 2H), 7.51 (d, J = 7.8 Hz, 2H), 7.46 – 7.42 (m, 2H), 7.42 – 7.38 (m, 2H), 7.22 (t, J = 7.4 Hz, 1H), 4.44 (dqd, J = 7.7, 6.3, 3.6 Hz, 1H), 4.04 (t, J = 8.8 Hz, 1H), 2.49 (ddd, J = 12.9, 8.7, 7.7 Hz, 1H), 2.31 (ddd, J = 12.8, 9.0, 3.7 Hz, 1H), 1.32 (d, J = 6.3 Hz, 3H). **^{13}C NMR** (100 MHz, $CDCl_3$) δ 172.85, 143.32 (d, J = 1.4 Hz), 137.58, 129.41 (d, J = 32.5 Hz), 129.08, 128.43, 125.75, 125.64 (q, J = 3.8 Hz), 123.18, 53.69, 47.75, 35.71, 19.55. **^{19}F NMR** (376 MHz, $CDCl_3$) δ -62.55. **Isomer 2. ^{1}H NMR** (400 MHz, $CDCl_3$) δ 7.63 (d, J = 8.0 Hz, 2H), 7.50 (d, J = 8.0 Hz, 2H), 7.45 – 7.38 (m, 2H), 7.36 – 7.31 (m, 2H), 7.29 – 7.19 (m, 1H), 4.32 (dp, J = 8.8, 6.2 Hz, 1H), 3.90 (dd, J = 11.2, 8.9 Hz, 1H), 2.86 (ddd, J = 12.8, 9.0, 6.4 Hz, 1H), 1.88 (ddd, J = 12.7, 11.2, 8.7 Hz, 1H), 1.27 (d, J = 6.1 Hz, 3H). **^{13}C NMR** (100 MHz, $CDCl_3$) δ 173.20, 143.25 (d, J = 1.2 Hz), 137.18, 129.39 (d, J = 32.4 Hz), 128.95, 128.59, 126.22, 125.58 (q, J = 3.8 Hz), 124.67, 53.02, 48.26, 36.75, 20.63. **^{19}F NMR** (376 MHz, $CDCl_3$) δ -62.53. **HRMS–ESI** (m/z): $[M+H]^+$ calcd. for $C_{18}H_{16}NOF_3$, 320.1257; found, 320.1244.

3-(4-chlorophenyl)-5-methyl-1-phenylpyrrolidin-2-one (4g)

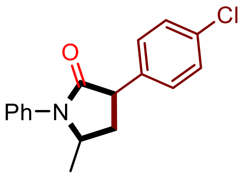

Colorless oil. **Isomer 1. ^{1}H NMR** (400 MHz, $CDCl_3$) δ 7.51 (d, J = 7.8 Hz, 2H), 7.40 (dd, J = 8.4, 7.5 Hz, 2H), 7.34 – 7.28 (m, 2H), 7.27 – 7.16 (m, 3H), 4.48 – 4.37 (m, 1H), 3.95 (t, J = 8.7 Hz, 1H), 2.45 (ddd, J = 12.8, 8.6, 7.7 Hz, 1H), 2.28 (ddd, J = 12.8, 8.9, 3.8 Hz, 1H), 1.31 (d, J = 6.3 Hz, 3H). **^{13}C NMR** (100 MHz, $CDCl_3$) δ 173.24, 137.76, 137.68, 132.94, 129.37, 129.04, 128.83, 125.64, 123.15, 53.58, 47.32, 35.85, 19.56. **Isomer 2. ^{1}H NMR** (400 MHz, $CDCl_3$) δ 7.43 – 7.38 (m, 2H), 7.37 – 7.29 (m, 6H), 7.23 (ddt, J = 7.9, 6.8, 1.3 Hz, 1H), 4.29 (dp, J = 8.7, 6.2 Hz, 1H), 3.82 (dd, J = 11.2, 8.9 Hz, 1H), 2.83 (ddd, J = 12.7, 8.9, 6.4 Hz, 1H), 1.84 (ddd, J = 12.8, 11.2, 8.7 Hz, 1H), 1.26 (d, J = 6.1 Hz, 3H). **^{13}C NMR** (100 MHz, $CDCl_3$) δ 173.56, 137.74, 137.31, 132.96, 129.54, 128.92, 128.79, 126.11, 124.64, 52.94, 47.86, 36.88, 20.66. **HRMS–ESI** (m/z): $[M+H]^+$ calcd. for $C_{17}H_{17}NOCl$, 286.0994; found, 286.0998.

4-(5-methyl-2-oxo-1-phenylpyrrolidin-3-yl)benzonitrile (4h)

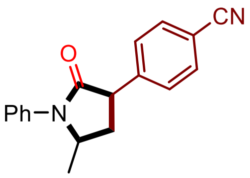

Colorless oil. **Isomer 1.** **^{1}H NMR** (400 MHz, $CDCl_3$) *δ* 7.64 (d, J = 8.3 Hz, 2H), 7.50 (d, J = 7.7 Hz, 2H), 7.44 (d, J = 9.5 Hz, 2H), 7.43 – 7.38 (m, 2H), 7.25 – 7.18 (m, 1H), 4.44 (dqd, J = 7.7, 6.3, 3.5 Hz, 1H), 4.04 (t, J = 8.9 Hz, 1H), 2.48 (ddd, J = 12.9, 8.8, 7.7 Hz, 1H), 2.32 (ddd, J = 12.7, 9.0, 3.5 Hz, 1H), 1.33 (d, J = 6.3 Hz, 3H). **^{13}C NMR** (100 MHz, $CDCl_3$) *δ* 172.33, 144.64, 137.41, 132.46, 129.12, 128.91, 125.89, 123.19, 118.70, 111.07, 53.72, 47.87, 35.43, 19.53. **Isomer 2**. **^{1}H NMR** (400 MHz, $CDCl_3$) *δ* 7.58 (dd, J = 64.9, 8.3 Hz, 4H), 7.46 – 7.38 (m, 2H), 7.35 – 7.30 (m, 2H), 7.29 – 7.21 (m, 1H), 4.32 (dt, J = 8.8, 6.2 Hz, 1H), 3.91 (dd, J = 11.4, 8.8 Hz, 1H), 2.86 (ddd, J = 12.8, 8.9, 6.3 Hz, 1H), 1.86 (ddd, J = 12.8, 11.3, 8.8 Hz, 1H), 1.27 (d, J = 6.1 Hz, 3H). **^{13}C NMR** (100 MHz, $CDCl_3$) *δ* 172.75, 144.56, 137.01, 132.40, 129.06, 128.99, 126.36, 124.70, 118.76, 111.04, 53.06, 48.39, 36.57, 20.60. **HRMS–ESI** (m/z): $[M+H]^+$ calcd. for $C_{18}H_{17}N_2O$, 277.1336; found, 277.1326.

3-(2-chlorophenyl)-5-methyl-1-phenylpyrrolidin-2-one (4i)

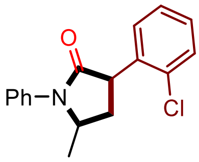

Colorless oil. **Isomer 1.** **^{1}H NMR** (400 MHz, $CDCl_3$) *δ* 7.56 – 7.52 (m, 2H), 7.45 – 7.37 (m, 3H), 7.27 – 7.17 (m, 4H), 4.53 – 4.25 (m, 2H), 2.35 (dd, J = 8.9, 5.7 Hz, 2H), 1.32 (d, J = 6.3 Hz, 3H). **^{13}C NMR** (100 MHz, $CDCl_3$) *δ* 173.10, 137.75, 137.55, 134.28, 129.76, 129.33, 129.07, 128.36, 127.23, 125.68, 123.29, 53.57, 46.09, 35.26, 19.63. **Isomer 1.** **^{1}H NMR** (400 MHz, $CDCl_3$) *δ* 7.46 – 7.36 (m, 6H), 7.33 – 7.19 (m, 3H), 4.34 (dt, J = 8.5, 6.3 Hz, 1H), 4.26 (dd, J = 10.9, 9.3 Hz, 1H), 2.92 (ddd, J = 12.7, 9.3, 6.7 Hz, 1H), 1.76 (ddd, J = 12.8, 10.9, 8.5 Hz, 1H), 1.24 (d, J = 6.1 Hz, 3H). **^{13}C NMR** (100 MHz, $CDCl_3$) *δ* 173.34, 137.76, 137.42, 134.30, 129.80, 129.75, 128.93, 128.37, 127.29, 126.08, 124.69, 53.21, 46.95, 36.25, 20.62. **HRMS–ESI** (m/z): $[M+H]^+$ calcd. for $C_{17}H_{17}NOCl$, 286.0994; found, 286.0998.

methyl 4-(5-methyl-2-oxo-1-phenylpyrrolidin-3-yl)benzoate (4j)

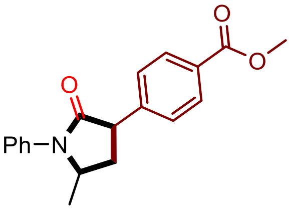

Colorless oil. **Isomer 1.** **^{1}H NMR** (400 MHz, $CDCl_3$) *δ* 8.02 (d, J = 8.4 Hz, 2H), 7.51 (d, J = 8.1 Hz, 2H), 7.40 (t, J = 8.5 Hz, 4H), 7.21 (ddt, J = 7.8, 7.0, 1.2 Hz, 1H), 4.47 – 4.38 (m, 1H), 4.04 (t, J = 8.7 Hz, 1H), 3.90 (s, 3H), 2.49 (ddd, J = 12.8, 8.4, 7.6 Hz, 1H), 2.31 (ddd, J = 12.9, 9.0, 3.8 Hz, 1H), 1.32 (d, J = 6.4 Hz, 3H). **^{13}C NMR** (100 MHz, $CDCl_3$) *δ* 172.98, 166.84, 144.55, 137.62, 130.01, 129.06, 129.00, 128.07, 125.72, 123.26, 53.70, 52.04, 47.95, 35.78, 19.62. **Isomer 2.** **^{1}H NMR** (400 MHz, $CDCl_3$) *δ* 8.05 (d, J = 8.3 Hz, 2H), 7.47 – 7.44 (m, 2H), 7.41 (dd, J = 8.4, 7.2 Hz, 2H), 7.36 – 7.32 (m, 2H), 7.27 – 7.22 (m, 1H), 4.31 (dt, J = 8.6, 6.2 Hz, 1H), 3.96 – 3.86 (m, 4H), 2.86 (ddd, J = 12.8, 9.0, 6.4 Hz, 1H), 1.88 (ddd, J = 12.8, 11.1, 8.6 Hz, 1H), 1.26 (d, J = 6.1 Hz, 3H). **^{13}C NMR** (100 MHz, $CDCl_3$) *δ* 173.31, 166.90, 144.53, 137.25, 129.95, 128.97, 128.93, 128.25, 126.16, 124.67, 53.06, 52.05, 48.47, 36.80, 20.65. **HRMS–ESI** (m/z): $[M+H]^+$ calcd. for $C_{19}H_{20}N_3O$, 310.1438; found, 310.1441.

3-([1,1'-biphenyl]-4-yl)-5-methyl-1-phenylpyrrolidin-2-one (4k)

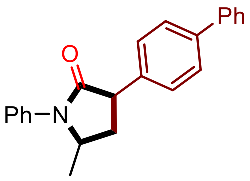

White solid. **Isomer 1.** **^{1}H NMR** (400 MHz, $CDCl_3$) δ 7.63 – 7.52 (m, 6H), 7.45 – 7.36 (m, 6H), 7.35 – 7.29 (m, 1H), 7.21 (t, J = 7.4 Hz, 1H), 4.46 (dddd, J = 8.6, 6.3, 3.9, 1.9 Hz, 1H), 4.02 (t, J = 8.6 Hz, 1H), 2.53 (ddd, J = 12.8, 8.3, 7.7 Hz, 1H), 2.31 (ddd, J = 12.9, 9.0, 3.9 Hz, 1H), 1.33 (d, J = 6.3 Hz, 3H). **^{13}C NMR** (100 MHz, $CDCl_3$) δ 173.76, 140.89, 140.08, 138.42, 137.85, 129.01, 128.71, 128.39, 127.50, 127.19, 127.08, 125.52, 123.17, 53.65, 47.74, 36.04, 19.66. **Isomer 2.** **^{1}H NMR** (400 MHz, $CDCl_3$) δ 7.63 – 7.56 (m, 4H), 7.48 – 7.42 (m, 4H), 7.43 – 7.38 (m, 2H), 7.38 – 7.31 (m, 3H), 7.25 – 7.17 (m, 1H), 4.32 (dt, J = 8.6, 6.2 Hz, 1H), 3.89 (dd, J = 11.1, 9.0 Hz, 1H), 2.87 (ddd, J = 12.7, 9.0, 6.5 Hz, 1H), 1.93 (ddd, J = 12.7, 11.1, 8.6 Hz, 1H), 1.28 (d, J = 6.1 Hz, 3H). **^{13}C NMR** (100 MHz, $CDCl_3$) δ 174.02, 140.98, 140.08, 138.39, 137.45, 128.87, 128.72, 128.58, 127.46, 127.18, 127.11, 125.98, 124.62, 52.95, 48.24, 36.96, 20.71. **HRMS–ESI** (m/z): $[M+H]^+$ calcd. for $C_{23}H_{23}NO$, 328.1696; found, 328.1709.

5-methyl-3-(naphthalen-2-yl)-1-phenylpyrrolidin-2-one (4l)

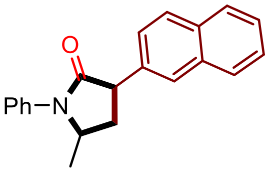

White solid. **^{1}H NMR** (400 MHz, $CDCl_3$) δ 7.89 – 7.81 (m, 4H), 7.54 – 7.44 (m, 3H), 7.43 – 7.36 (m, 3H), 7.26 – 7.22 (m, 2H), 4.35 (dp, J = 8.6, 6.2 Hz, 1H), 4.02 (dd, J = 10.9, 9.0 Hz, 1H), 2.91 (ddd, J = 12.8, 9.0, 6.5 Hz, 1H), 1.99 (ddd, J = 12.8, 11.0, 8.6 Hz, 1H), 1.29 (d, J = 6.2 Hz, 3H). **^{13}C NMR** (100 MHz, $CDCl_3$) δ 174.04, 137.49, 136.80, 133.51, 132.61, 128.91, 128.47, 127.80, 127.62, 126.98, 126.21, 126.07, 126.02, 125.74, 124.68, 53.09, 48.70, 37.03, 20.75. **HRMS–ESI** (m/z): $[M+H]^+$ calcd. for $C_{21}H_{20}NO$, 302.1540; found, 302.1540.

3,5-dimethyl-3-(naphthalen-2-yl)-1-phenylpyrrolidin-2-one (4m)

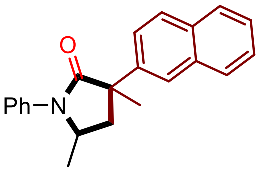

White solid. **Isomer 1.** **^{1}H NMR** (400 MHz, $CDCl_3$) δ 7.85 – 7.74 (m, 4H), 7.56 (dd, J = 8.6, 2.0 Hz, 1H), 7.47 – 7.42 (m, 2H), 7.42 – 7.36 (m, 2H), 7.34 – 7.28 (m, 2H), 7.22 (dt, J = 7.9, 1.3 Hz, 1H), 4.04 (dt, J = 9.3, 6.0 Hz, 1H), 2.91 (dd, J = 12.8, 5.8 Hz, 1H), 1.93 (dd, J = 12.8, 9.3 Hz, 1H), 1.73 (s, 3H), 1.19 (d, J = 6.1 Hz, 3H). **^{13}C NMR** (100 MHz, $CDCl_3$) δ 176.50, 140.44, 137.44, 133.25, 132.26, 128.87, 128.51, 128.08, 127.38, 126.11, 125.99, 125.84, 124.74, 124.54, 124.42, 51.66, 49.96, 44.32, 26.53, 20.13. **Isomer 2.** **^{1}H NMR** (400 MHz, $CDCl_3$) δ 8.00 (d, J = 1.9 Hz, 1H), 7.92 – 7.78 (m, 3H), 7.66 (dd, J = 8.7, 2.0 Hz, 1H), 7.54 – 7.37 (m, 6H), 7.24 – 7.18 (m, 1H), 4.38 – 4.25 (m, 1H), 2.59 (dd, J = 12.8, 7.2 Hz, 1H), 2.33 (dd, J = 12.8, 6.5 Hz, 1H), 1.69 (s, 3H), 1.14 (d, J = 6.2 Hz, 3H). **^{13}C NMR** (100 MHz, $CDCl_3$) δ 176.80, 142.34, 137.73, 133.27, 132.20, 128.92, 128.26, 128.10, 127.41, 126.01, 125.83, 125.75, 124.93, 124.70, 124.20, 51.99, 49.19, 43.40, 25.60, 20.14. **HRMS–ESI** (m/z): $[M+H]^+$ calcd. for $C_{22}H_{22}NO$, 316.1696; found, 316.1702.

3,5-dimethyl-1,3-diphenylpyrrolidin-2-one (4n)

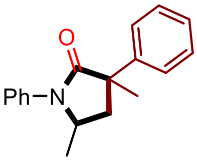

Colorless oil. **^{1}H NMR** (400 MHz, $CDCl_3$) δ 7.58 – 7.53 (m, 2H), 7.45 – 7.38 (m, 4H), 7.35 (d, J = 8.0 Hz, 2H), 7.25 – 7.18 (m, 2H), 4.38 – 4.21 (m, 1H), 2.53 (dd, J = 12.8, 7.1 Hz, 1H), 2.23 (dd, J = 12.8, 6.6 Hz, 1H), 1.61 (s, 3H), 1.13 (d, J = 6.2 Hz, 3H). **^{13}C NMR** (100 MHz, $CDCl_3$) δ 176.88, 145.09, 137.74, 128.90, 128.46, 126.60, 126.31, 125.80, 124.19, 51.88, 49.02, 43.54, 25.63, 20.10. **HRMS–ESI** (m/z): $[M+H]^+$ calcd. for $C_{18}H_{20}NO$, 266.1540; found, 266.1551.

3-(4-chlorophenyl)-3,5-dimethyl-1-phenylpyrrolidin-2-one (4o)

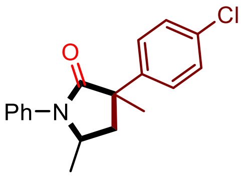

Colorless oil. **Isomer 1. ^{1}H NMR** (400 MHz, $CDCl_3$) δ 7.42 – 7.36 (m, 2H), 7.36 – 7.31 (m, 2H), 7.31 – 7.26 (m, 4H), 7.23 (ddt, J = 8.6, 7.1, 1.3 Hz, 1H), 4.01 (dt, J = 9.2, 6.0 Hz, 1H), 2.76 (dd, J = 12.9, 5.9 Hz, 1H), 1.87 (dd, J = 12.9, 9.2 Hz, 1H), 1.62 (s, 3H), 1.18 (d, J = 6.1 Hz, 3H). **^{13}C NMR** (100 MHz, $CDCl_3$) δ 176.14, 141.80, 137.27, 132.71, 128.91, 128.75, 127.54, 126.11, 124.66, 51.61, 49.36, 44.16, 26.63, 20.17. **Isomer 1. ^{1}H NMR** (400 MHz, $CDCl_3$) δ 7.50 (d, J = 8.6 Hz, 2H), 7.44 – 7.37 (m, 4H), 7.33 (d, J = 8.6 Hz, 2H), 7.25 – 7.19 (m, 1H), 4.30 (ddt, J = 13.2, 7.0, 6.2 Hz, 1H), 2.52 (dd, J = 12.8, 7.0 Hz, 1H), 2.17 (dd, J = 12.8, 6.9 Hz, 1H), 1.58 (s, 3H), 1.14 (d, J = 6.2 Hz, 3H). **^{13}C NMR** (100 MHz, $CDCl_3$) δ 176.43, 143.51, 137.54, 132.52, 128.95, 128.55, 127.83, 125.99, 124.27, 51.86, 48.56, 43.32, 25.71, 20.16. **HRMS–ESI** (m/z): $[M+H]^+$ calcd. for $C_{18}H_{19}NOCl$, 300.1150; found, 300.1160.

5-methyl-1,3-diphenyl-3-(pyridin-2-yl)pyrrolidin-2-one (4p)

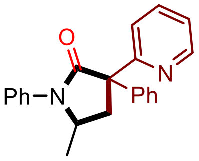

White solid. **mixture of diastereomers and rotamers. ^{1}H NMR** (400 MHz, $CDCl_3$) δ 8.58 (dddd, J = 13.1, 4.8, 1.9, 1.0 Hz, 2H), 7.52 (dtd, J = 11.0, 7.7, 1.9 Hz, 2H), 7.36 (d, J = 7.1 Hz, 2H), 7.32 – 7.27 (m, 4H), 7.27 – 7.24 (m, 2H), 7.20 (ddt, J = 7.6, 5.1, 1.9 Hz, 2H), 7.13 – 7.03 (m, 8H), 6.62 (tdt, J = 7.3, 3.9, 1.1 Hz, 2H), 6.41 (d, J = 7.7 Hz, 2H), 6.36 (d, J = 7.9 Hz, 2H), 4.39 – 4.21 (m, 2H), 3.42 – 3.26 (m, 2H), 2.56 – 2.36 (m, 2H), 2.34 – 2.10 (m, 2H), 1.19 (dd, J = 9.0, 6.2 Hz, 6H). ^{13}C NMR (100 MHz, $CDCl_3$) δ 163.68, 163.35, 149.23, 149.09, 147.64, 147.55, 143.66, 143.53, 136.36, 136.32, 129.14, 128.56, 128.52, 128.21, 128.08, 126.55, 126.49, 123.26, 123.08, 121.31, 121.30, 116.88, 116.78, 113.24, 113.19, 50.50, 50.47, 46.93, 46.87, 42.77, 42.70, 21.31, 21.02. **HRMS–ESI** (m/z): $[M+H]^+$ calcd. for $C_{22}H_{21}N_2O$, 329.1649; found, 329.1643.

(3*S*,8*S*,9*S*,10*R*,13*R*,14*S*,17*R*)-10,13-dimethyl-17-((*R*)-6-methylheptan-2-yl)-2,3,4,7,8,9,10,11,12,13,14,15,16,17-tetradecahydro-1*H*-cyclopenta[a]phenanthren-3-yl-4-(5-ethyl-2-oxo-3,3-diphenylpyrrolidin-1-yl)benzoate (5a)

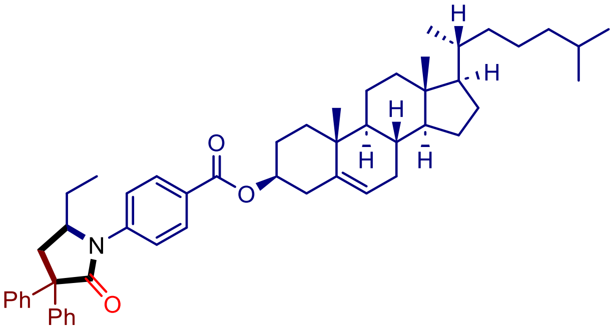

White solid. **^{1}H NMR** (400 MHz, $CDCl_3$) δ 7.25 (dd, J = 459.7, 8.7 Hz, 4H), 7.40 – 7.30 (m, 7H), 7.30 – 7.25 (m, 3H), 5.40 (d, J = 5.0 Hz, 1H), 5.03 – 4.66 (m, 1H), 4.23 (s, 1H), 3.40 – 3.05 (m, 2H), 2.56 – 2.37 (m, 3H), 1.91 (tddd, J = 31.4, 19.0, 12.1, 6.6 Hz, 6H), 1.75 – 1.57 (m, 2H), 1.54 – 1.45 (m, 4H), 1.35 (s, 4H), 1.31 – 1.07 (m, 8H), 1.06 (s, 3H), 1.06 – 0.94 (m, 3H), 0.97 – 0.88 (m, 6H), 0.87 (dd, J = 6.6, 1.8 Hz, 6H), 0.69 (s, 3H). **^{13}C NMR** (100 MHz, $CDCl_3$) δ 176.10, 165.81, 146.39, 142.04, 141.82,

139.84, 130.98, 129.07, 128.66, 127.57, 127.49, 127.41, 127.30, 122.60, 116.25, 95.17, 74.06, 58.17, 56.73, 56.18, 50.09, 47.71, 42.35, 39.78, 39.54, 38.31, 37.08, 36.68, 36.21, 35.81, 31.95, 31.92, 30.10, 28.24, 28.02, 27.96, 24.31, 23.85, 22.81, 22.56, 21.07, 19.39, 18.73, 11.87, 8.08. This compound is hard to protonate, so we can't find the signal in HRMS.

(*R*)-5-((1*S*,4a*R*,10a*S*)-7-isopropyl-1,4a-dimethyl-1,2,3,4,4a,9,10,10a-octahydrophenanthren-1-yl)-1,3, 3-triphenylpyrrolidin-2-one (5b)

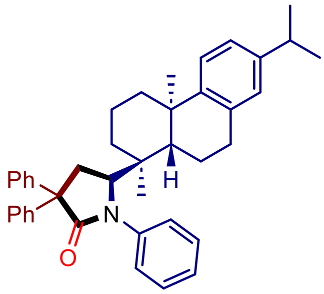

Colorless oil. **^{1}H NMR** (400 MHz, $CDCl_3$) δ 7.44 – 7.36 (m, 4H), 7.35 – 7.24 (m, 5H), 7.24 – 7.17 (m, 1H), 7.11 (h, J = 8.0, 7.6 Hz, 4H), 6.92 (dd, J = 12.5, 7.7 Hz, 2H), 6.82 (dd, J = 8.2, 2.0 Hz, 1H), 6.60 (d, J = 1.9 Hz, 1H), 4.37 (dd, J = 9.0, 6.7 Hz, 1H), 2.97 (dd, J = 13.5, 6.7 Hz, 1H), 2.80 – 2.69 (m, 1H), 2.60 (ddd, J = 29.2, 15.4, 8.2 Hz, 2H), 2.18 – 2.04 (m, 2H), 1.78 – 1.58 (m, 6H), 1.56 – 1.44 (m, 2H), 1.17 (d, J = 6.9 Hz, 6H), 1.08 (s, 3H), 0.91 (s, 3H). **^{13}C NMR** (100 MHz, $CDCl_3$) δ 177.31, 147.00, 145.09, 144.65, 142.37, 141.21, 133.90, 128.94, 128.48, 128.17, 128.02, 127.86, 127.03, 126.97, 126.49, 126.18, 123.15, 123.09, 62.22, 57.07, 42.35, 40.70, 37.76, 37.47, 36.48, 33.33, 30.46, 28.09, 25.22, 24.04, 23.86, 18.33, 18.29, 18.06. **HRMS–ESI** (m/z): $[M+H]^+$ calcd. for $C_{41}H_{46}NO$, 568.3574; found, 568.3585.

(*Z*)-5-(heptadec-8-en-1-yl)-1,3,3-triphenylpyrrolidin-2-one (5c)

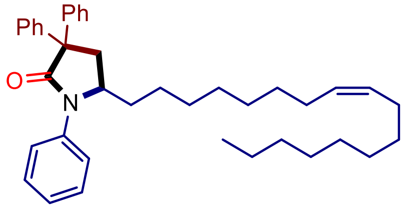

White solid. **^{1}H NMR** (400 MHz, $CDCl_3$) δ 7.44 (dd, J = 8.3, 1.1 Hz, 2H), 7.38 (dd, J = 10.5, 4.9 Hz, 4H), 7.34 – 7.26 (m, 6H), 7.26 – 7.18 (m, 3H), 5.41 – 5.22 (m, 2H), 4.07 – 3.98 (m, 1H), 2.78 (ddd, J = 21.8, 12.8, 7.4 Hz, 2H), 1.99 (dt, J = 13.1, 6.6 Hz, 3H), 1.80 – 1.67 (m, 1H), 1.37 – 1.12 (m, 24H), 0.87 (t, J = 6.8 Hz, 3H). **^{13}C NMR** (100 MHz, $CDCl_3$) δ 174.61, 144.07, 142.22, 137.55, 129.96, 129.66, 128.80, 128.48, 128.15, 128.02, 127.66, 126.96, 126.66, 126.08, 124.82, 57.82, 55.74, 40.94, 33.31, 31.85, 29.71, 29.62, 29.47, 29.44, 29.41, 29.39, 29.26, 29.02, 27.17, 27.10, 24.48, 22.63, 14.06. **HRMS–ESI** (m/z): $[M+H]^+$ calcd. for $C_{39}H_{52}NO$, 550.4043; found, 550.4022.

(*S*)-5-((1*R*,2*S*,5*R*)-6,6-dimethylbicyclo[3.1.1]heptan-2-yl)-1,3,3-triphenylpyrrolidin-2-one (5d)

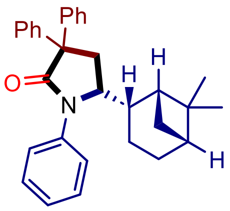

White solid. **^{1}H NMR** (400 MHz, $CDCl_3$) δ 7.51 – 7.48 (m, 2H), 7.41 – 7.35 (m, 2H), 7.35 – 7.32 (m, 3H), 7.31 – 7.24 (m, 6H), 7.21 (ddt, J = 8.4, 6.1, 1.4 Hz, 2H), 4.27 (ddd, J = 9.9, 6.8, 5.6 Hz, 1H), 2.89 (ddd, J = 18.6, 13.1, 6.2 Hz, 2H), 2.10 – 1.99 (m, 2H), 1.89 (tt, J = 10.7, 4.3 Hz, 1H), 1.80 – 1.68 (m, 3H), 1.58 – 1.51 (m, 1H), 1.47 (dt, J = 6.5, 3.3 Hz, 1H), 0.97 (s, 3H), 0.74 (s, 3H), 0.50 (d, J = 9.7 Hz, 1H). **^{13}C NMR** (100 MHz, $CDCl_3$) δ 175.39, 144.04, 143.46, 139.90, 128.82, 128.36, 128.29, 127.79, 126.78, 126.74, 126.42, 126.02, 61.06, 57.31, 46.91, 42.79, 40.81, 39.48, 37.89, 33.61, 27.27, 26.08, 23.33, 18.63. **HRMS–ESI** (m/z): $[M+H]^+$ calcd. for $C_{31}H_{34}NO$, 436.2635; found, 436.2634.

2,5,7,8-tetramethyl-2-(4,8,12-trimethyltridecyl)chroman-6-yl-4-(5-ethyl-2-oxo-3,3-diphenylpyrolidin-1-yl)benzoate (5e)

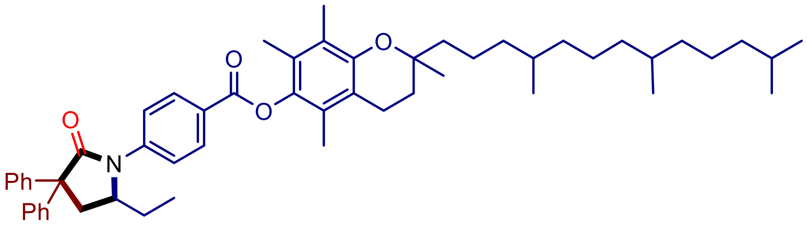

Colorless oil. **^{1}H NMR** (400 MHz, Chloroform-d) *δ* 7.37 (dd, J = 513.6, 8.8 Hz, 4H), 7.40 – 7.32 (m, 8H), 7.28 (dddd, J = 8.0, 4.9, 3.5, 1.5 Hz, 2H), 4.34 (s, 1H), 3.38 – 3.05 (m, 2H), 2.60 (t, J = 6.8 Hz, 2H), 2.52 (dq, J = 14.9, 7.4 Hz, 1H), 2.11 (s, 3H), 2.04 (s, 3H), 2.00 (s, 3H), 1.95 (dd, J = 14.4, 7.4 Hz, 1H), 1.80 (dd, J = 15.1, 8.1 Hz, 2H), 1.55 – 1.48 (m, 2H), 1.39 (pt, J = 9.5, 6.2 Hz, 4H), 1.34 – 1.18 (m, 12H), 1.17 – 1.04 (m, 6H), 0.96 (t, J = 7.4 Hz, 3H), 0.86 (t, J = 7.0 Hz, 12H). **^{13}C NMR** (100 MHz, $CDCl_3$) *δ* 176.08, 164.93, 149.31, 147.04, 141.98, 141.81, 140.70, 131.68, 129.11, 128.66, 127.62, 127.50, 127.41, 127.30, 127.06, 125.25, 122.98, 120.87, 117.37, 116.18, 94.99, 77.32, 77.00, 76.68, 75.01, 58.12, 47.86, 39.38, 37.58, 37.47, 37.40, 37.30, 32.80, 32.78, 30.10, 27.97, 24.81, 24.80, 24.45, 22.71, 22.62, 21.05, 20.63, 19.75, 19.69, 19.66, 19.60, 13.04, 12.19, 11.82, 8.12. This compound is hard to protonate, so we can't find the signal in HRMS.

(*Z*)-3,7-dimethylocta-2,6-dien-1-yl 4-(5-ethyl-2-oxo-3,3-diphenylpyrrolidin-1-yl)benzoate (5f)

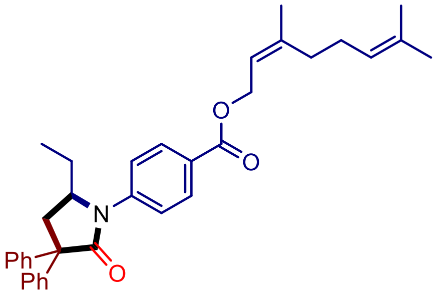

Colorless oil. **^{1}H NMR** (400 MHz, $CDCl_3$) *δ* 8.07 (d, J = 8.7 Hz, 2H), 7.43 (t, J = 7.8 Hz, 4H), 7.36 – 7.29 (m, 5H), 7.29 – 7.22 (m, 4H), 5.48 (t, J = 7.2 Hz, 1H), 5.14 – 5.09 (m, 1H), 4.80 (d, J = 6.8 Hz, 2H), 4.08 (tdd, J = 8.9, 6.0, 2.9 Hz, 1H), 2.80 (ddd, J = 21.8, 12.9, 7.4 Hz, 2H), 2.22 – 2.05 (m, 4H), 1.88 – 1.74 (m, 4H), 1.67 (s, 3H), 1.60 (s, 3H), 1.43 – 1.32 (m, 1H), 0.86 (t, J = 7.5 Hz, 3H). **^{13}C NMR** (100 MHz, $CDCl_3$) *δ* 174.84, 166.08, 143.73, 142.74, 142.00, 141.69, 132.20, 130.32, 128.62, 128.60, 128.29, 128.02, 127.62, 127.17, 126.89, 123.61, 123.57, 119.28, 61.63, 58.01, 56.27, 39.76, 32.26, 26.70, 25.68, 25.59, 23.53, 17.67, 8.43. **HRMS–ESI** (m/z): $[M+H]^+$ calcd. for $C_{35}H_{40}NO_2$, 522.3003; found, 522.2986.

(1*S*,2*S*,5*R*)-2-isopropyl-5-methylcyclohexyl 4-(5-methyl-2-oxo-1-phenylpyrrolidin-3-yl)benzoate (5g)

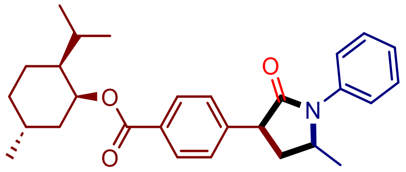

White solid. **^{1}H NMR** (400 MHz, $CDCl_3$) *δ* 8.02 (d, J = 8.3 Hz, 2H), 7.51 (ddd, J = 8.5, 2.0, 1.2 Hz, 2H), 7.44 – 7.35 (m, 4H), 7.21 (t, J = 7.4 Hz, 1H), 4.92 (td, J = 10.8, 4.4 Hz, 1H), 4.43 (dtd, J = 12.6, 6.2, 3.8 Hz, 1H), 4.03 (t, J = 8.7 Hz, 1H), 2.57 – 2.42 (m, 1H), 2.30 (ddd, J = 12.8, 9.0, 3.8 Hz, 1H), 2.16 – 2.05 (m, 1H), 1.94 (ddtd, J = 9.9, 7.1, 4.1, 2.9, 1.6 Hz, 1H), 1.78 – 1.67 (m, 2H), 1.62 – 1.47 (m, 2H), 1.32 (d, J = 6.3 Hz, 3H), 1.21 – 1.02 (m, 2H), 0.96 – 0.87 (m, 7H), 0.78 (d, J = 6.9 Hz, 3H). **^{13}C NMR** (100 MHz, $CDCl_3$) *δ* 173.05, 173.03, 165.81, 144.35, 144.28, 137.66, 130.02, 130.00, 129.72, 129.05, 128.00, 127.98, 125.68, 123.23, 123.20, 74.80, 53.69, 48.01, 47.97, 47.30, 40.97, 35.87, 35.75, 34.34, 31.44, 26.54, 23.71, 22.02, 20.72, 19.62, 16.56. **^{1}H NMR** (400 MHz, $CDCl_3$) *δ* 8.14 – 8.00 (m, 2H), 7.47 – 7.42 (m, 2H), 7.42 – 7.37 (m, 2H), 7.36 – 7.31 (m, 2H), 7.28 – 7.20 (m, 1H), 4.93 (td, J = 10.9, 4.4 Hz, 1H),

4.56 – 4.15 (m, 1H), 3.90 (dd, J = 11.1, 9.0 Hz, 1H), 2.85 (ddd, J = 12.8, 9.0, 6.4 Hz, 1H), 2.18 – 2.05 (m, 1H), 2.02 – 1.83 (m, 2H), 1.73 (dt, J = 11.7, 2.9 Hz, 2H), 1.62 – 1.51 (m, 2H), 1.26 (d, J = 6.1 Hz, 3H), 1.11 (dt, J = 12.2, 1.6 Hz, 2H), 0.92 (t, J = 6.9 Hz, 7H), 0.79 (d, J = 7.0 Hz, 3H). **^{13}C NMR** (100 MHz, $CDCl_3$) *δ* 173.35, 173.32, 165.85, 144.33, 144.25, 137.26, 129.95, 129.93, 129.69, 128.90, 128.17, 128.15, 126.10, 124.63, 74.78, 53.02, 53.01, 48.52, 48.45, 47.31, 40.97, 36.81, 36.65, 35.41, 34.34, 31.44, 26.56, 26.55, 23.72, 22.02, 20.71, 20.65, 16.57. **HRMS–ESI** (m/z): $[M+H]^+$ calcd. for $C_{28}H_{36}NO_3$, 434.2690; found, 434.2686.

Isopropyl-2-(4-(3-(4-chlorophenyl)-5-methyl-2-oxo-1-phenylpyrrolidin-3-yl)phenoxy)-2-methylpr opanoa te (5h)

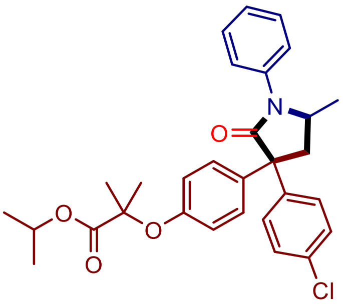

White solid. **mixture of diastereomers and rotamers.** **^{1}H NMR** (400 MHz, Chloroform-d) *δ* 7.39 (td, J = 7.9, 7.4, 1.0 Hz, 4H), 7.35 – 7.30 (m, 4H), 7.29 – 7.22 (m, 12H), 7.22 – 7.16 (m, 2H), 6.84 – 6.73 (m, 4H), 5.06 (h, J = 6.3 Hz, 2H), 4.11 (ddp, J = 9.2, 5.9, 3.1 Hz, 2H), 3.08 – 2.85 (m, 2H), 2.41 (ddd, J = 28.9, 12.8, 8.9 Hz, 2H), 1.57 (d, J = 1.2 Hz, 12H), 1.35 – 1.09 (m, 18H). **^{13}C NMR** (100 MHz, $CDCl_3$) *δ* 174.32, 174.29, 173.60, 173.47, 154.76, 154.45, 142.59, 140.98, 137.26, 136.82, 134.59, 132.92, 132.65, 129.43, 129.05, 128.89, 128.87, 128.62, 128.58, 128.29, 128.22, 126.18, 126.15, 124.70, 124.58, 118.76, 118.64, 79.11, 79.08, 68.88, 68.87, 57.02, 51.49, 43.27, 43.15, 25.49, 25.44, 25.37, 25.36, 21.55, 21.52, 19.89, 19.84. **HRMS–ESI** (m/z): $[M+H]^+$ calcd. for $C_{30}H_{33}NO_4Cl$, 506.2093; found, 506.2097.

2-(2-fluoro-[1,1'-biphenyl]-4-yl)propyl 4-(5-methyl-2-oxo-1-phenylpyrrolidin-3-yl)benzoate (5i)

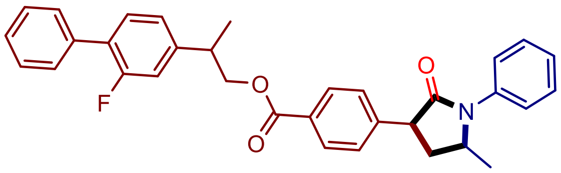

White solid. **Isomer 1.** **^{1}H NMR** (400 MHz, $CDCl_3$) *δ* 7.97 (d, J = 8.3 Hz, 2H), 7.57 – 7.49 (m, 4H), 7.47 – 7.30 (m, 8H), 7.23 – 7.18 (m, 1H), 7.16 – 6.99 (m, 2H), 4.47 – 4.37 (m, 3H), 4.02 (t, J = 8.7 Hz, 1H), 3.28 (q, J = 7.0 Hz, 1H), 2.48 (dt, J = 12.9, 8.0 Hz, 1H), 2.29 (ddd, J = 12.8, 9.0, 3.8 Hz, 1H), 1.41 (d, J = 7.0 Hz, 3H), 1.31 (d, J = 6.3 Hz, 3H). **^{13}C NMR** (100 MHz, $CDCl_3$) *δ* 172.94, 166.14, 160.97, 158.50, 144.87 (d, J = 7.3 Hz), 144.67, 137.60, 135.63, 130.67 (d, J = 4.0 Hz), 129.99, 129.05, 128.93 (d, J = 2.9 Hz), 128.37, 128.11, 127.51, 127.27 (d, J = 13.5 Hz), 125.70, 123.34 (d, J = 3.2 Hz), 123.22, 114.90 (d, J = 23.2 Hz), 69.40, 53.68, 47.96, 38.66 (d, J = 1.4 Hz), 35.73, 19.60, 17.85. **^{19}F NMR** (376 MHz, $CDCl_3$) δ -118.18. **Isomer 2.** **^{1}H NMR** (400 MHz, $CDCl_3$) *δ* 8.00 (d, J = 8.3 Hz, 2H), 7.55 (dt, J = 8.1, 1.5 Hz, 2H), 7.47 – 7.37 (m, 7H), 7.38 – 7.30 (m, 3H), 7.28 – 7.20 (m, 1H), 7.17 – 7.07 (m, 2H), 4.49 – 4.39 (m, 2H), 4.30 (qd, J = 6.2, 2.5 Hz, 1H), 3.89 (dd, J = 11.1, 9.0 Hz, 1H), 3.29 (q, J = 7.0 Hz, 1H), 2.84 (ddd, J = 12.8, 9.0, 6.4 Hz, 1H), 1.87 (ddd, J = 12.7, 11.1, 8.7 Hz, 1H), 1.42 (d, J = 7.0 Hz, 3H), 1.25 (d, J = 6.1 Hz, 3H). **^{13}C NMR** (100 MHz, $CDCl_3$) *δ* 173.27, 166.21, 160.98, 158.52, 144.90 (d, J = 7.3 Hz), 144.65, 137.24, 135.66, 130.69 (d, J = 4.0 Hz), 129.95, 128.96, 128.92, 128.39, 128.29, 127.52, 127.29 (d, J = 13.6 Hz), 126.15, 124.65, 123.36 (d, J = 3.1 Hz), 114.92 (d, J = 23.0 Hz), 69.41, 53.03, 48.49, 38.69 (d, J = 1.5 Hz), 36.77, 20.64, 17.87. **^{19}F NMR** (376 MHz, $CDCl_3$) *δ* -118.16. **HRMS–ESI** (m/z): $[M+H]^+$ calcd. for $C_{33}H_{31}NO_3F$, 508.2283; found,508.2261.

(*R*)-2-(4-isobutylphenyl)propyl 4-(5-methyl-2-oxo-1-phenylpyrrolidin-3-yl)benzoate (5j)

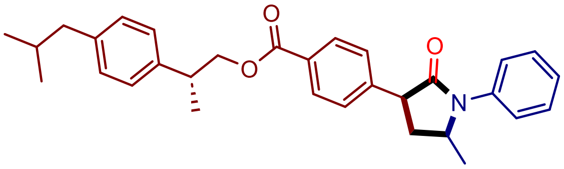

White solid. **Isomer 1.** **^{1}H NMR** (400 MHz, $CDCl_3$) δ 7.96 (d, J = 8.4 Hz, 2H), 7.59 – 7.46 (m, 2H), 7.46 – 7.32 (m, 4H), 7.28 – 7.15 (m, 3H), 7.15 – 6.98 (m, 2H), 4.49 – 4.30 (m, 3H), 4.02 (t, J = 8.7 Hz, 1H), 3.20 (q, J = 7.0 Hz, 1H), 2.52 – 2.39 (m, 3H), 2.29 (ddd, J = 12.9, 9.0, 3.8 Hz, 1H), 1.85 (dt, J = 13.5, 6.8 Hz, 1H), 1.37 (d, J = 7.0 Hz, 3H), 1.31 (d, J = 6.3 Hz, 3H), 0.89 (d, J = 6.7 Hz, 6H). **^{13}C NMR** (100 MHz, $CDCl_3$) δ 172.97, 166.21, 144.47, 140.30, 139.99, 137.61, 129.97, 129.20, 129.18, 129.04, 128.01, 126.98, 125.68, 123.21, 69.96, 53.66, 47.94, 45.01, 38.65, 35.75, 30.15, 22.37, 22.35, 19.59, 17.98. **Isomer 2.** **^{1}H NMR** (400 MHz, $CDCl_3$) δ 7.99 (d, J = 8.4 Hz, 2H), 7.47 – 7.37 (m, 4H), 7.37 – 7.31 (m, 2H), 7.28 – 7.21 (m, 1H), 7.19 (d, J = 8.1 Hz, 2H), 7.10 (d, J = 8.1 Hz, 2H), 4.45 – 4.35 (m, 2H), 4.34 – 4.24 (m, 1H), 3.89 (dd, J = 11.1, 9.0 Hz, 1H), 3.22 (h, J = 7.0 Hz, 1H), 2.84 (ddd, J = 12.8, 9.0, 6.5 Hz, 1H), 2.45 (d, J = 7.2 Hz, 2H), 1.97 – 1.76 (m, 2H), 1.38 (d, J = 7.0 Hz, 3H), 1.25 (d, J = 6.1 Hz, 3H), 0.90 (d, J = 6.6 Hz, 6H). **^{13}C NMR** (100 MHz, $CDCl_3$) δ 173.29, 166.28, 144.46, 140.34, 140.00, 137.25, 129.92, 129.20, 128.91, 128.19, 126.99, 126.12, 124.63, 69.97, 53.01, 48.45, 45.02, 38.67, 36.75, 30.16, 22.38, 22.36, 20.64, 17.99. **HRMS–ESI** (m/z): $[M+H]^+$ calcd. for $C_{31}H_{36}NO_3$, 470.2690; found, 470.2684.

(1*R*,2*R*,5*S*)-2-isopropyl-5-methylcyclohexyl 4-((5*R*)-5-((1*S*,4a*R*,10a*S*)-7-isopropyl-1,4a-dimethyl-1,2,3,4,4a,9,10,10a-octahydrophenanthren-1-yl)-2-oxo-1-phenylpyrrolidin-3-yl)benzoate (5k)

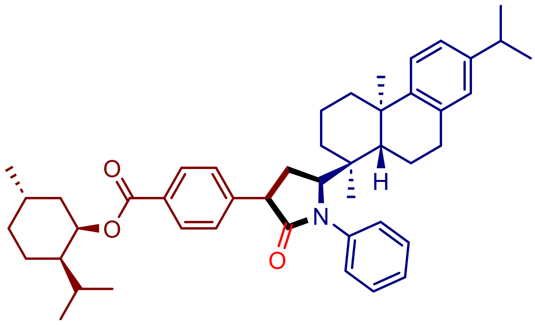

White solid. **^{1}H NMR** (400 MHz, $CDCl_3$) δ 7.98 (d, J = 8.4 Hz, 2H), 7.32 (d, J = 8.3 Hz, 2H), 7.30 – 7.25 (m, 4H), 7.18 (ddt, J = 8.5, 5.6, 3.2 Hz, 1H), 7.04 (d, J = 8.2 Hz, 1H), 6.90 (dd, J = 8.1, 2.0 Hz, 1H), 6.56 (d, J = 1.9 Hz, 1H), 4.90 (td, J = 10.9, 4.4 Hz, 1H), 4.45 (d, J = 9.3 Hz, 1H), 4.04 (dd, J = 11.7, 8.7 Hz, 1H), 2.81 – 2.71 (m, 1H), 2.68 (dd, J = 13.6, 8.8 Hz, 1H), 2.49 – 2.31 (m, 2H), 2.26 (dt, J = 12.8, 3.4 Hz, 1H), 2.11 (dtd, J = 12.0, 3.9, 1.6 Hz, 1H), 1.92 (td, J = 7.0, 2.7 Hz, 1H), 1.83 – 1.61 (m, 6H), 1.60 – 1.45 (m, 6H), 1.37 – 1.22 (m, 2H), 1.19 (d, J = 6.9 Hz, 6H), 1.15 (s, 3H), 1.11 – 1.02 (m, 2H), 1.00 (s, 3H), 0.90 (dd, J = 11.3, 6.8 Hz, 6H), 0.77 (d, J = 6.9 Hz, 3H). **^{13}C NMR** (100 MHz, $CDCl_3$) δ 175.33, 165.86, 146.66, 145.31, 145.18, 140.11, 134.27, 129.86, 129.57, 128.87, 128.25, 126.49, 126.46, 126.40, 123.65, 123.36, 74.76, 64.75, 48.16, 47.30, 43.39, 42.93, 40.98, 38.11, 37.63, 34.35, 33.34, 32.10, 32.05, 31.44, 28.10, 26.54, 25.72, 23.99, 23.88, 23.73, 22.02, 20.70, 19.14, 18.64, 18.39, 16.57. **HRMS–ESI** (m/z): $[M+Na]^+$ calcd. for $C_{46}H_{59}NO_3Na$, 696.4387; found, 696.4385.

2-(2-fluoro-[1,1'-biphenyl]-4-yl)propyl 4-((5*R*)-5-((1*S*,4a*R*,10a*S*)-7-isopropyl-1,4a-dimethyl-1,2,3,4,4a,9,10,10a-octahydrophenanthren-1-yl)-2-oxo-1-phenylpyrrolidin-3-yl)benzoate (5l)

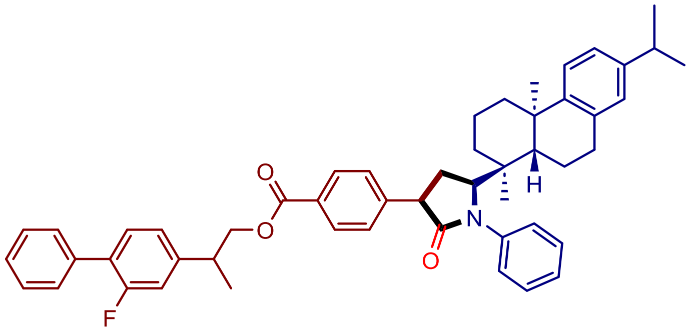

White solid. **^{1}H NMR** (400 MHz, $CDCl_3$) δ 7.93 (d, J = 8.2 Hz, 2H), 7.54 (dt, J = 8.2, 1.5 Hz, 2H), 7.45 – 7.39 (m, 2H), 7.39 – 7.36 (m, 1H), 7.35 – 7.30 (m, 3H), 7.30 – 7.25 (m, 4H), 7.22 – 7.14 (m, 1H), 7.15 – 7.08 (m, 2H), 7.08 – 7.00 (m, 1H), 6.90 (dd, J = 8.2, 2.0 Hz, 1H), 6.56 (d, J = 2.0 Hz, 1H), 4.51 – 4.30 (m, 3H), 4.04 (dd, J = 11.7, 8.7 Hz, 1H), 3.26 (p, J = 6.9 Hz, 1H), 2.77 (h, J = 6.9 Hz, 1H), 2.65 (d, J = 8.8 Hz, 1H), 2.51 – 2.30 (m, 2H), 2.26 (d, J = 12.9 Hz, 1H), 1.77 (ddt, J = 11.9, 7.1, 3.6 Hz, 2H), 1.63 (dd, J = 10.2, 6.0 Hz, 2H), 1.53 (dd, J = 4.3, 2.6 Hz, 3H), 1.40 (d, J = 7.0 Hz, 3H), 1.28 (ddd, J = 18.2, 10.8, 6.9 Hz, 2H), 1.18 (d, J = 6.8 Hz, 6H), 1.14 (s, 3H), 1.00 (s, 3H). **^{13}C NMR** (100 MHz, $CDCl_3$) δ 175.22, 166.21, 160.98, 158.51, 146.65, 145.40 (d, J = 15.8 Hz), 144.89 (d, J = 7.6 Hz), 140.07, 135.66, 134.25, 130.68 (d, J = 3.9 Hz), 129.84, 128.95 (d, J = 2.9 Hz), 128.88, 128.83, 128.38, 127.51, 127.35, 127.21, 126.49, 126.48, 126.38, 123.64, 123.36, 114.91 (d, J = 23.0 Hz), 77.32, 77.00, 76.68, 69.40, 64.77, 48.11, 43.39, 42.94, 38.67, 38.11, 37.63, 33.35, 32.14, 31.99, 28.11, 25.72, 24.00, 23.88, 19.15, 18.65, 18.38, 17.86. **^{19}F NMR** (376 MHz, $CDCl_3$) δ -117.99. **HRMS–ESI** (m/z): $[M+Na]^+$ calcd. for $C_{51}H_{54}NO_3FNa$, 770.3979; found, 770.3980.

(*R*)-2-(4-isobutylphenyl)propyl 4-((5*R*)-5-((1*S*,4a*R*,10a*S*)-7-isopropyl-1,4a-dimethyl-1,2,3,4,4a,9,10,10a-octahydrophenanthren-1-yl)-2-oxo-1-phenylpyrrolidin-3-yl)benzoate (5m)

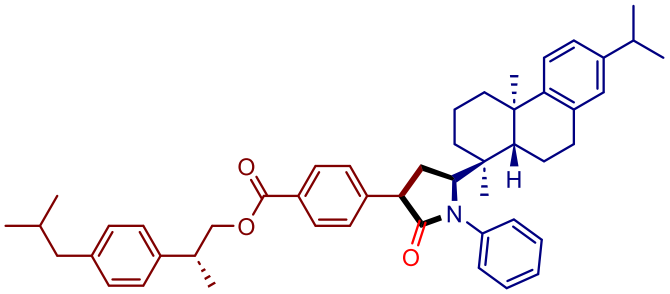

White solid. **^{1}H NMR** (400 MHz, $CDCl_3$) δ 7.92 (d, J = 8.3 Hz, 2H), 7.33 – 7.26 (m, 6H), 7.21 – 7.14 (m, 3H), 7.11 – 7.06 (m, 2H), 7.04 (d, J = 8.2 Hz, 1H), 6.90 (dd, J = 8.2, 2.0 Hz, 1H), 6.56 (d, J = 2.0 Hz, 1H), 4.44 (d, J = 9.3 Hz, 1H), 4.42 – 4.28 (m, 2H), 4.03 (dd, J = 11.7, 8.7 Hz, 1H), 3.19 (q, J = 7.0 Hz, 1H), 2.77 (dq, J = 13.8, 6.9 Hz, 1H), 2.67 (dd, J = 13.6, 8.8 Hz, 1H), 2.44 (d, J = 7.2 Hz, 2H), 2.43 – 2.31 (m, 2H), 2.26 (dt, J = 12.9, 3.4 Hz, 1H), 1.91 – 1.74 (m, 3H), 1.63 (dd, J = 9.5, 6.4 Hz, 2H), 1.57 – 1.48 (m, 3H), 1.36 (d, J = 7.0 Hz, 3H), 1.33 – 1.22 (m, 2H), 1.19 (d, J = 6.9 Hz, 6H), 1.15 (s, 3H), 1.00 (s, 3H), 0.89 (d, J = 6.6 Hz, 6H). **^{13}C NMR** (100 MHz, $CDCl_3$) δ 175.24, 166.29, 146.66, 145.32, 145.28, 140.34, 140.09, 140.01, 134.26, 129.83, 129.19, 129.08, 128.88, 128.28, 127.00, 126.49, 126.47, 126.39, 123.65, 123.36, 69.97, 64.76, 48.10, 45.04, 43.39, 42.94, 38.67, 38.11, 37.63, 33.35, 32.14, 31.99, 30.17, 28.11, 25.72, 24.00, 23.88, 22.39, 22.38, 19.15, 18.66, 18.38, 17.99. **HRMS–ESI** (m/z): $[M+Na]^+$ calcd. for $C_{49}H_{59}NO_3Na$, 732.4387; found, 732.4384.

5. ^{1}H NMR and ^{13}C NMR Spectra.

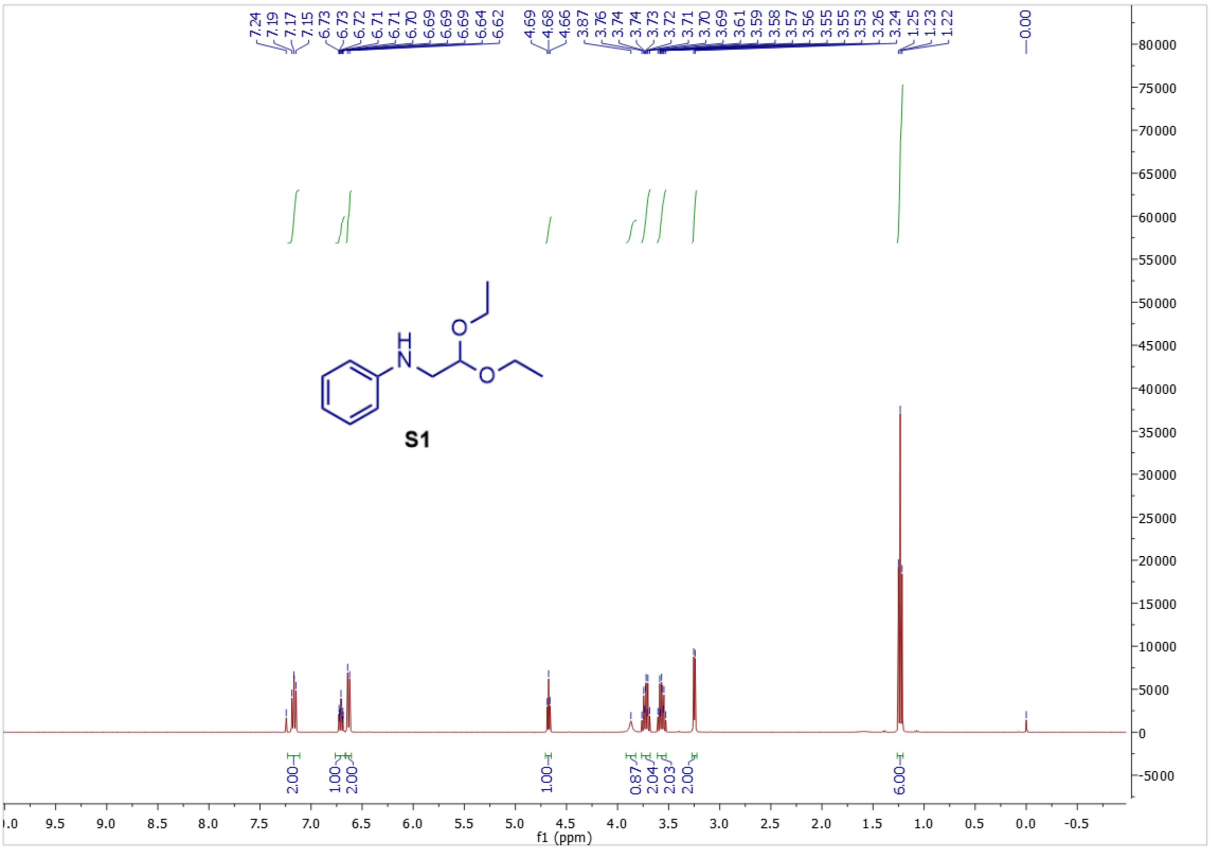

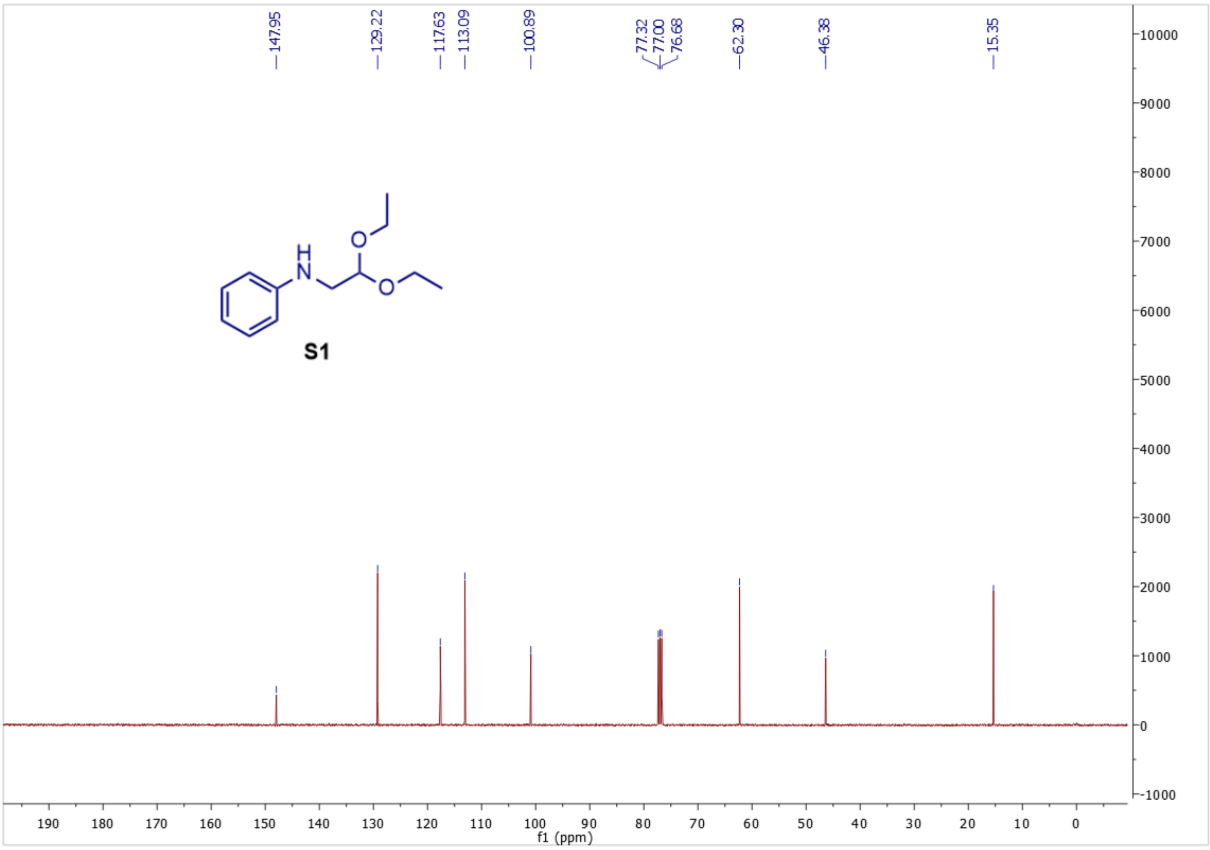

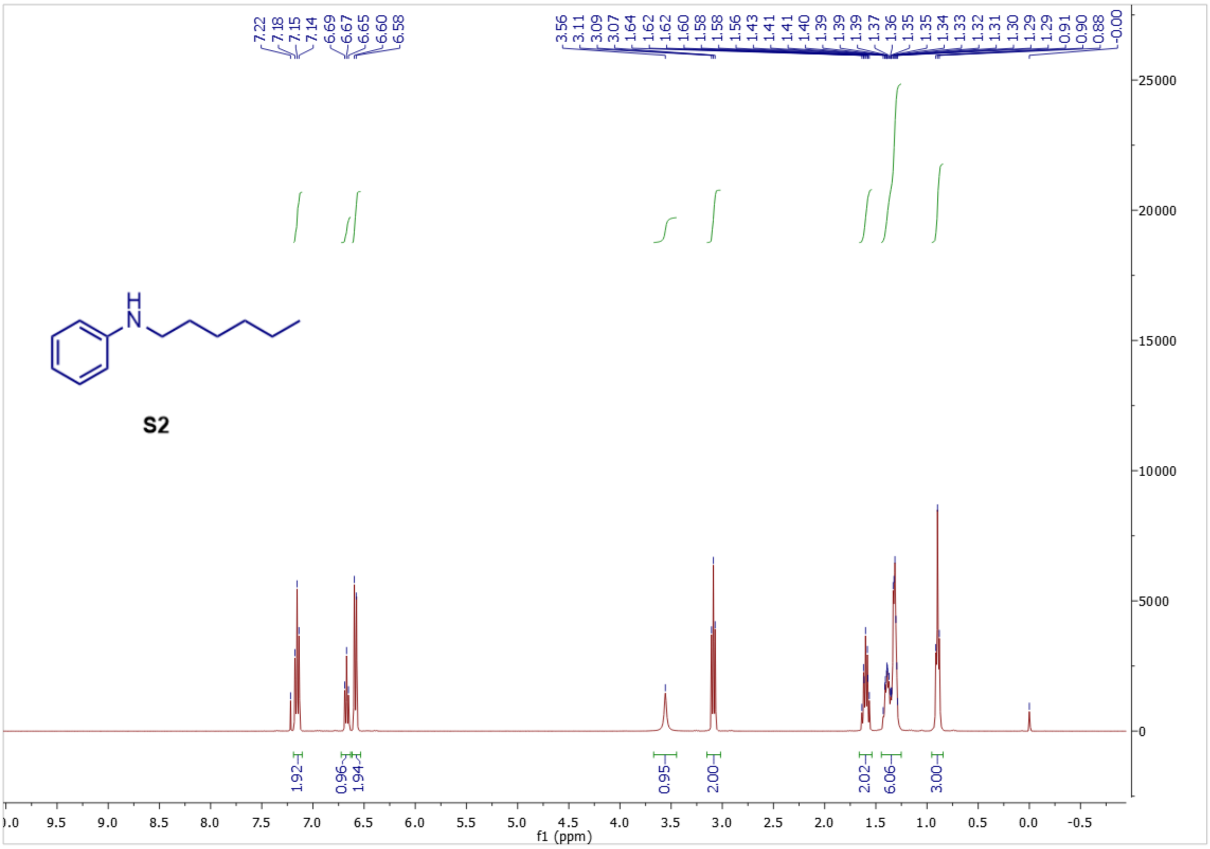

7.22
7.18
7.15
7.14
6.69
6.67
6.65
6.60
6.58
3.56
3.11
3.09
3.07
1.64
1.62
1.62
1.60
1.58
1.58
1.58
1.56
1.43
1.41
1.41
1.40
1.39
1.39
1.39
1.37
1.36
1.35
1.35
1.34
1.33
1.32
1.31
1.30
1.29
1.29
0.91
0.90
0.88
-0.00
S2
1.92
0.96
1.94
0.95
2.00
2.02
6.06
3.00
f1 (ppm)

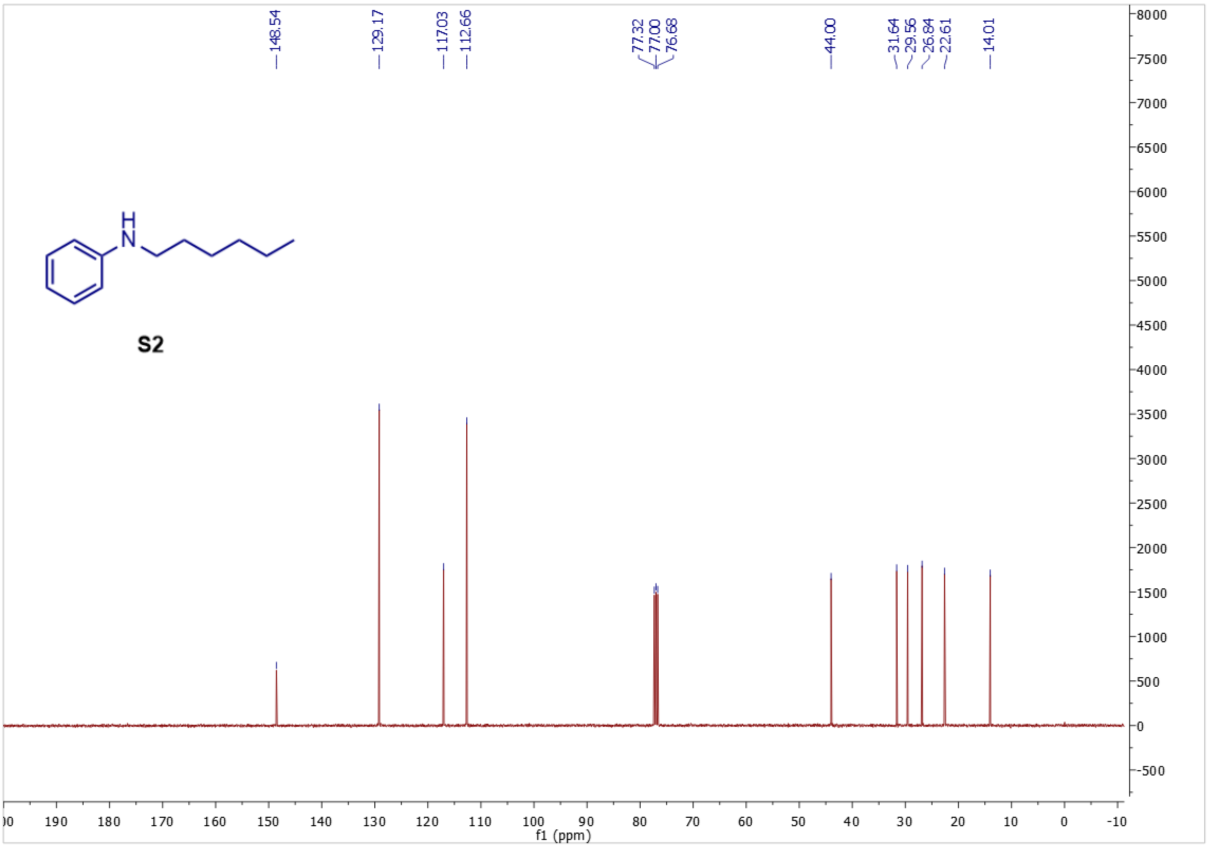

148.54
129.17
117.03
112.66
77.32
77.00
76.68
44.00
31.64
29.56
26.84
22.61
14.01
S2
f1 (ppm)

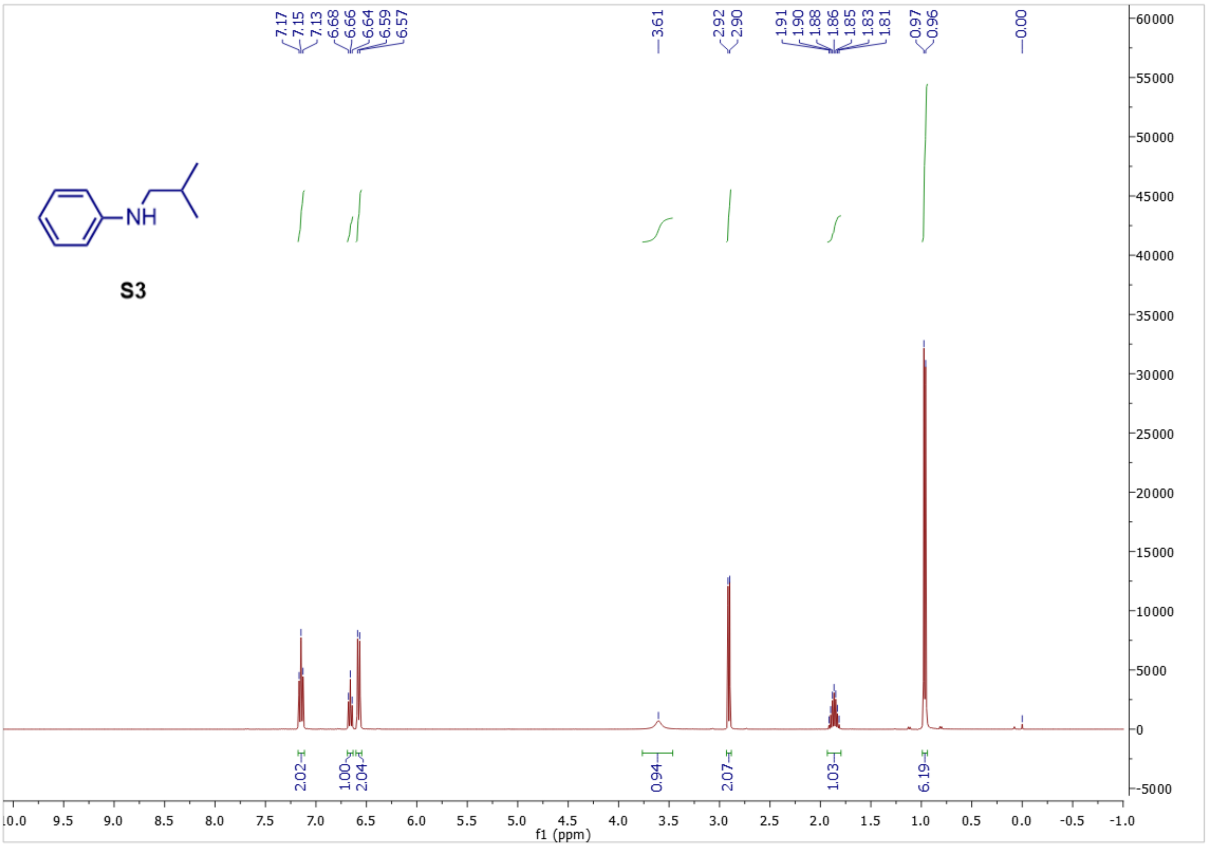

S3
NH
f1 (ppm)

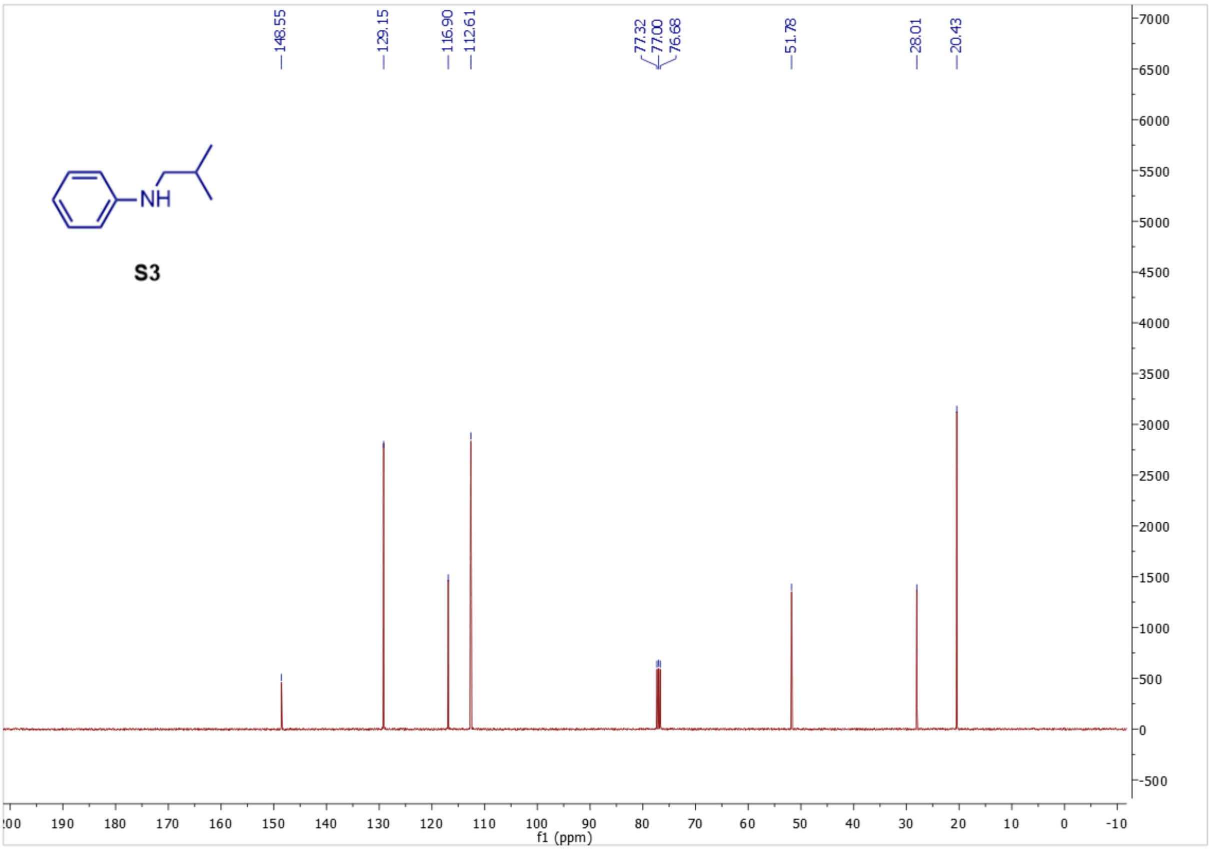

S3
NH
f1 (ppm)

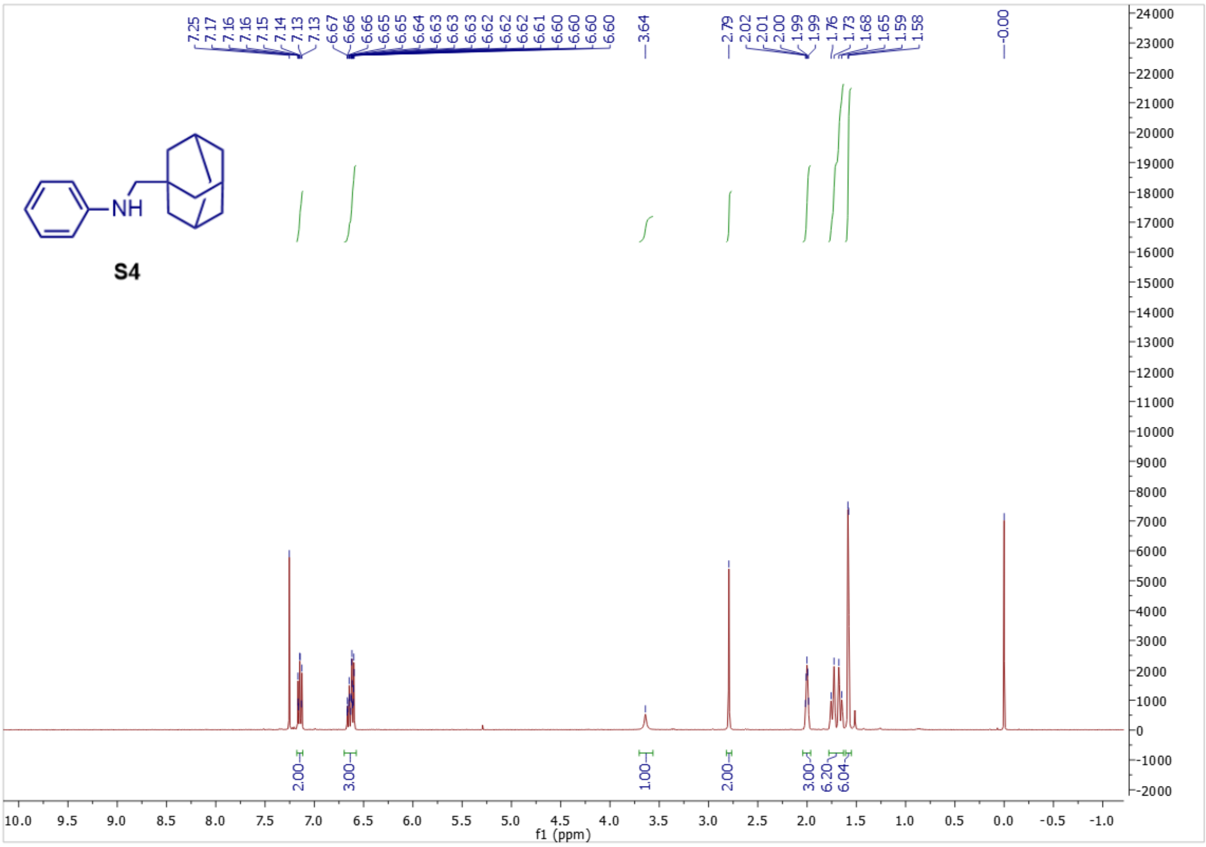
7.25
7.17
7.16
7.16
7.15
7.14
7.13
7.13
6.67
6.66
6.65
6.65
6.64
6.63
6.63
6.63
6.62
6.62
6.62
6.61
6.60
6.60
6.60
6.60
3.64
2.79
2.02
2.01
2.00
1.99
1.99
1.76
1.73
1.68
1.65
1.59
1.58
0.00
NH
S4
2.00
3.00
1.00
2.00
3.00
6.20
6.04
f1 (ppm)

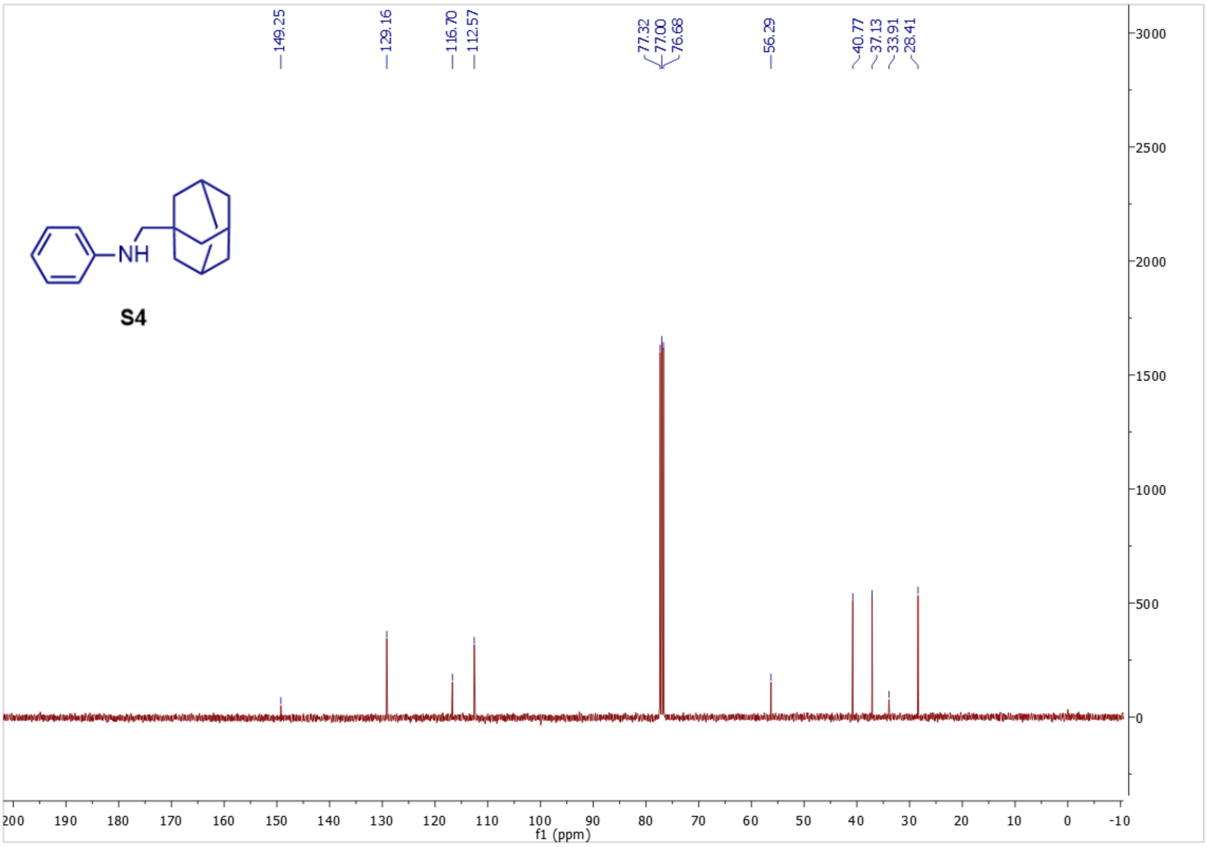
149.25
129.16
116.70
112.57
77.32
77.00
76.68
56.29
40.77
37.13
33.91
28.41
NH
S4
f1 (ppm)

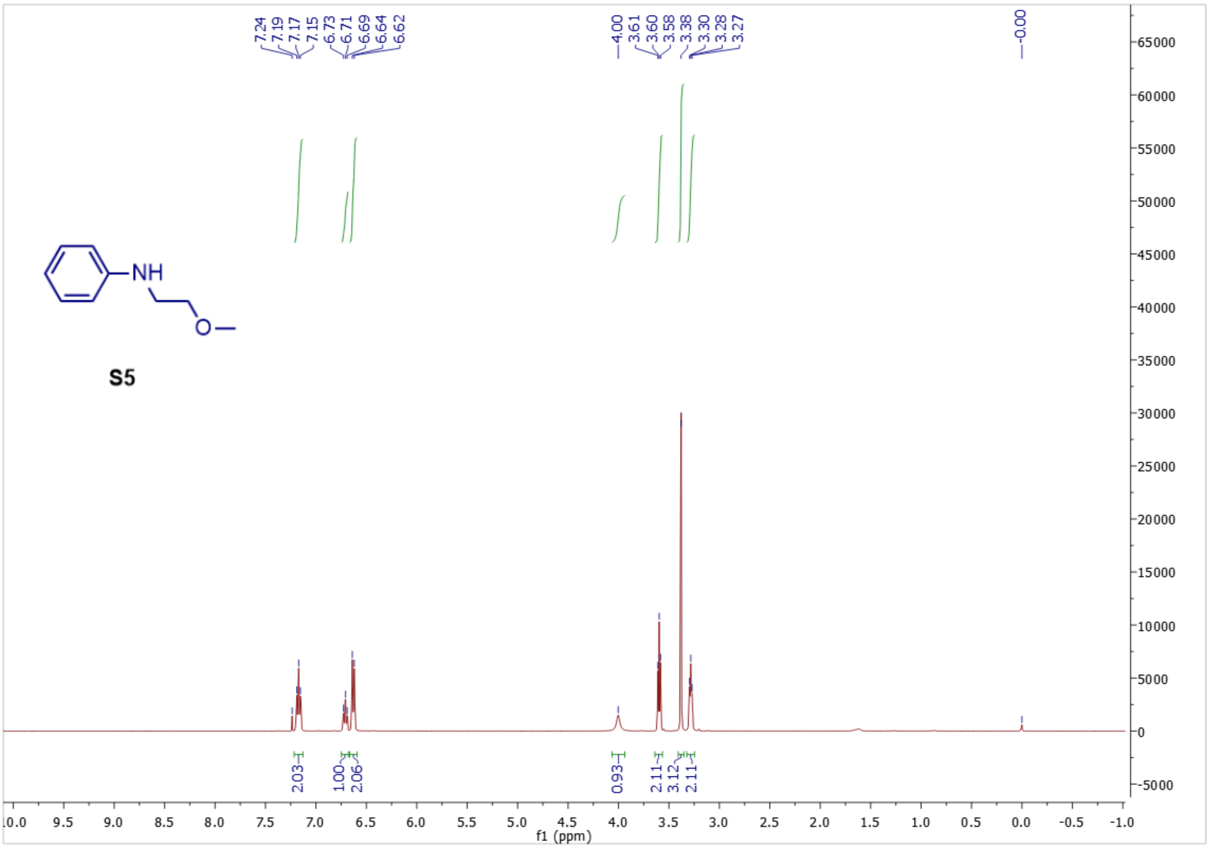
7.24
7.19
7.17
7.15
6.73
6.71
6.69
6.64
6.62
4.00
3.61
3.60
3.58
3.38
3.30
3.28
3.27
-0.00
NH
O
2.03
1.00
2.06
0.93
2.11
3.12
2.11
f1 (ppm)

S5

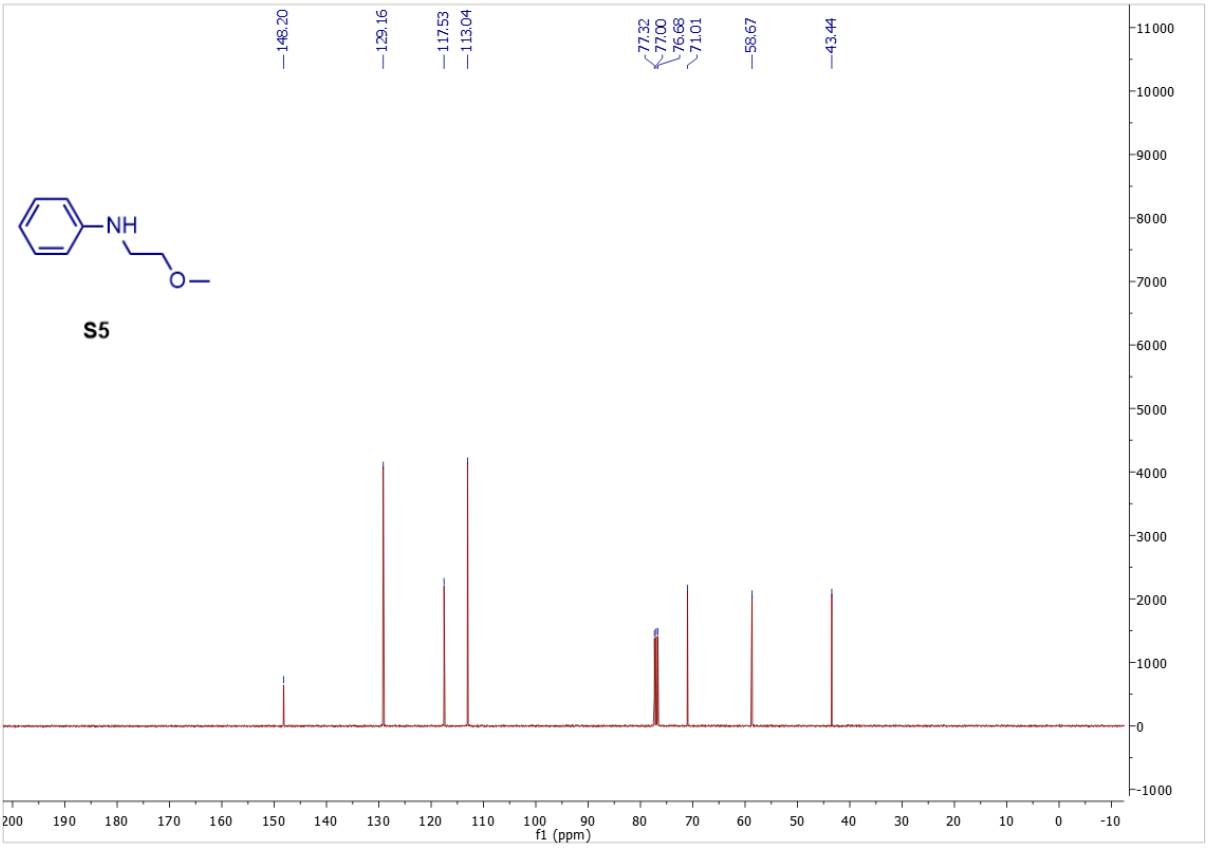
148.20
129.16
117.53
113.04
77.32
77.00
76.68
71.01
58.67
43.44
NH
O
f1 (ppm)

S5

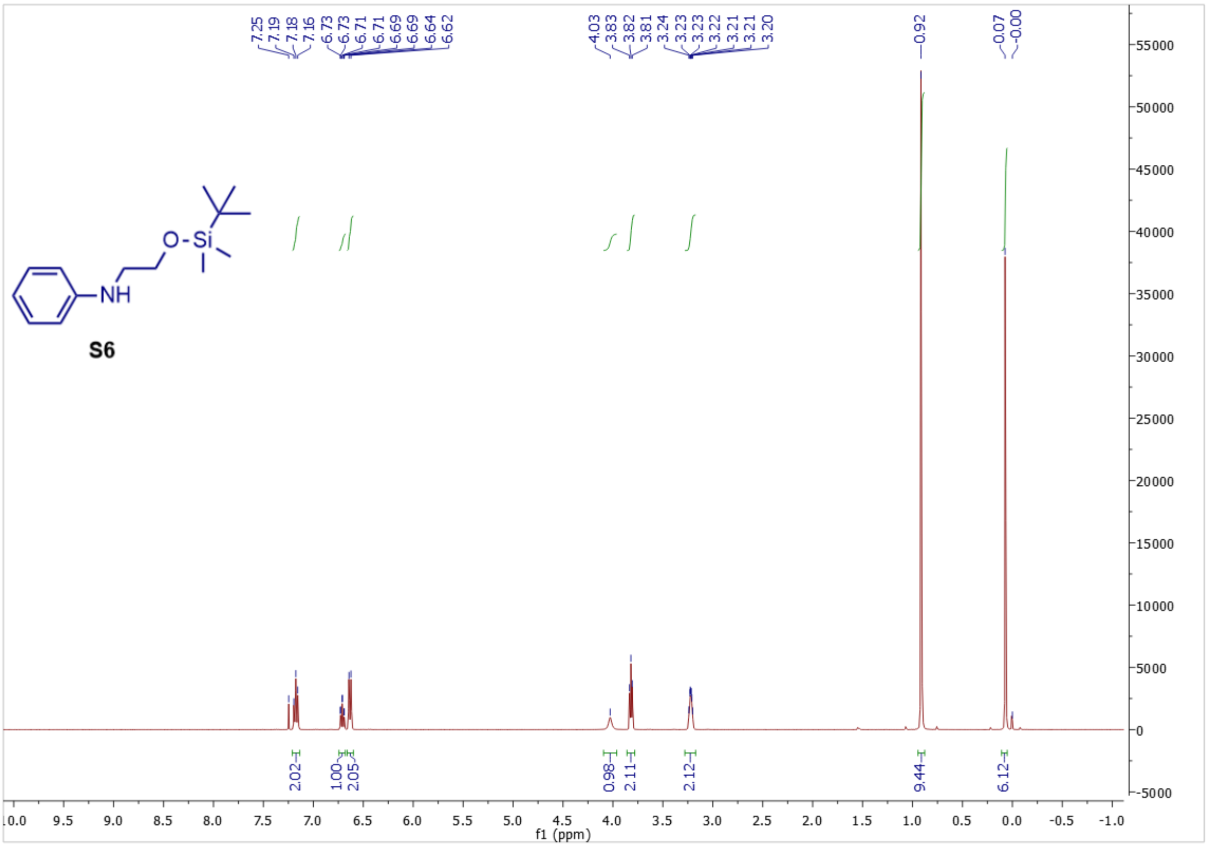
7.25
7.19
7.18
7.16
6.73
6.73
6.71
6.71
6.69
6.69
6.64
6.62
4.03
3.83
3.82
3.81
3.24
3.23
3.23
3.22
3.21
3.21
3.20
0.92
0.07
0.00
O-Si
NH
S6
2.02
1.00
2.05
0.98
2.11
2.12
9.44
6.12
f1 (ppm)

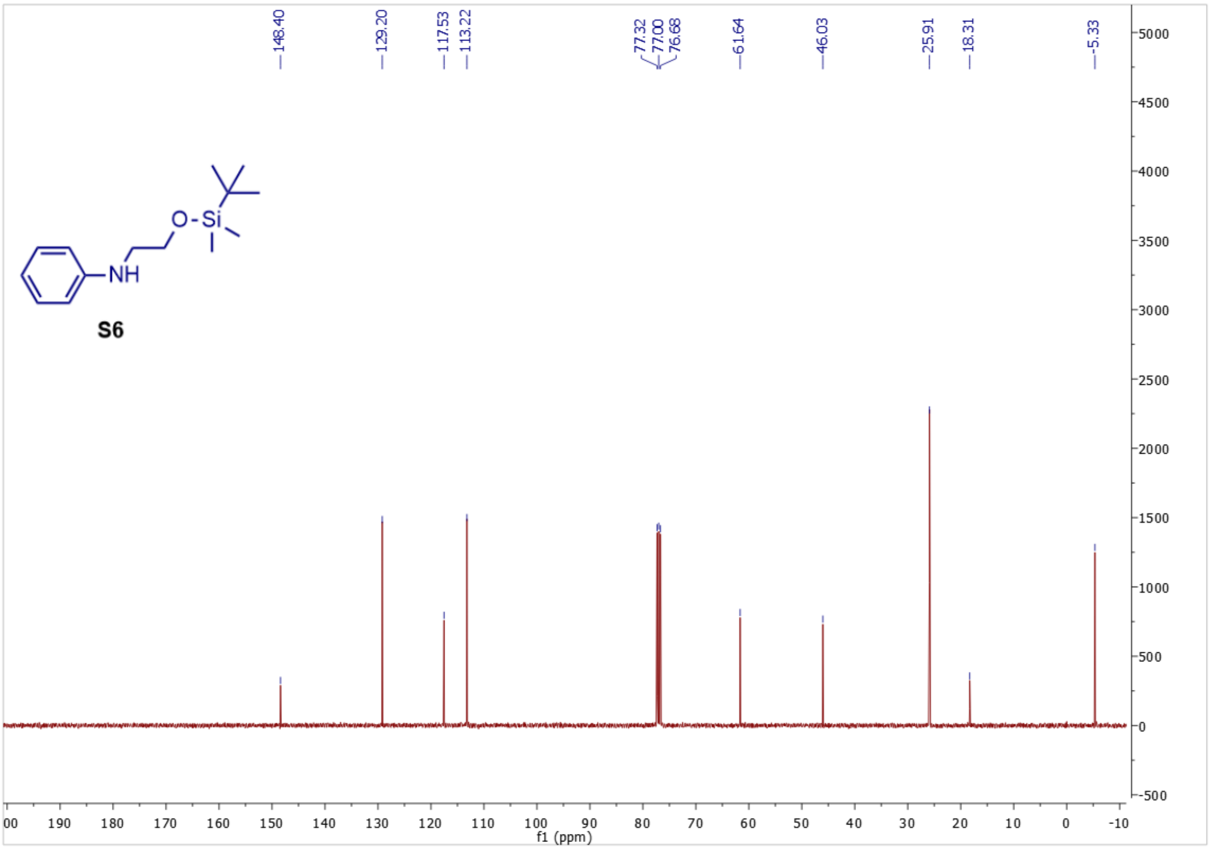
148.40
129.20
117.53
113.22
77.32
77.00
76.68
61.64
46.03
25.91
18.31
-5.33
O-Si
NH
S6
f1 (ppm)

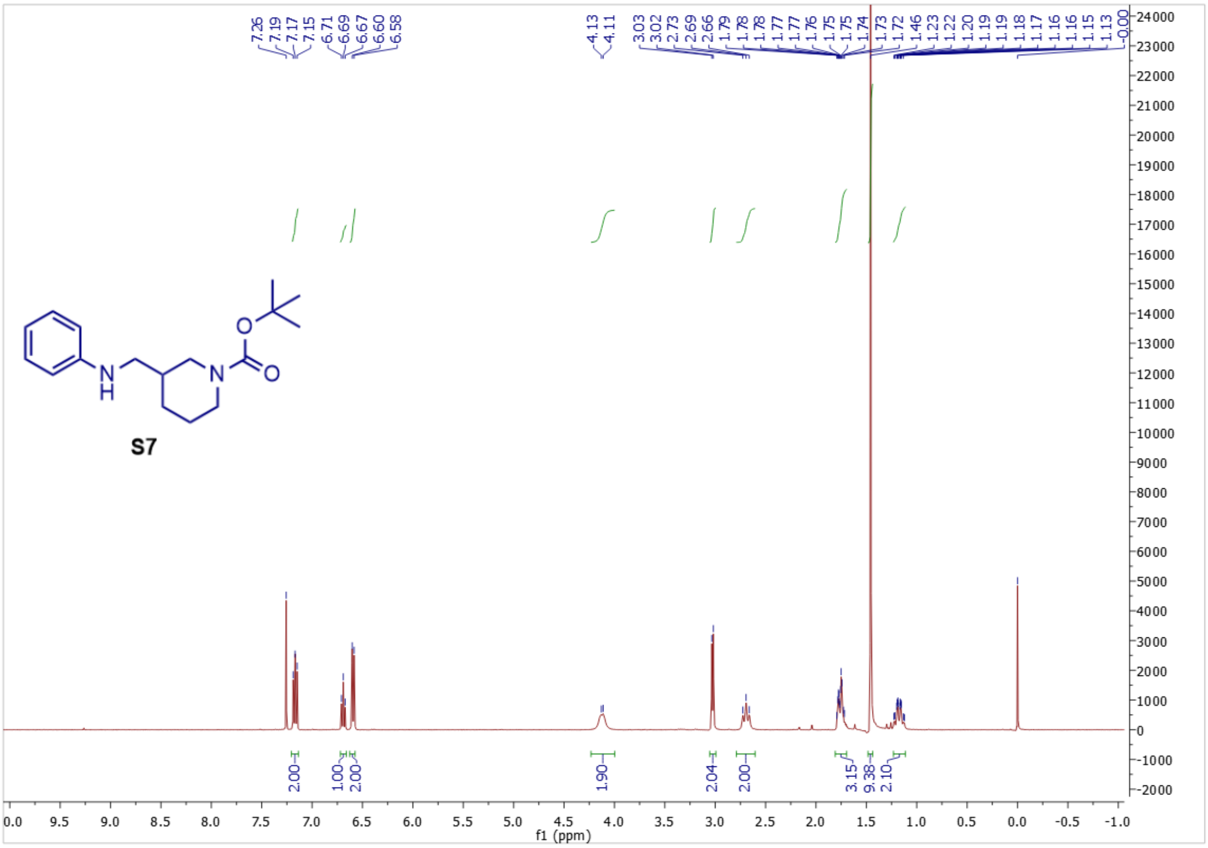
7.26
7.19
7.17
7.15
6.71
6.69
6.67
6.60
6.58
4.13
4.11
3.03
3.02
2.73
2.69
2.66
1.46
S7
f1 (ppm)

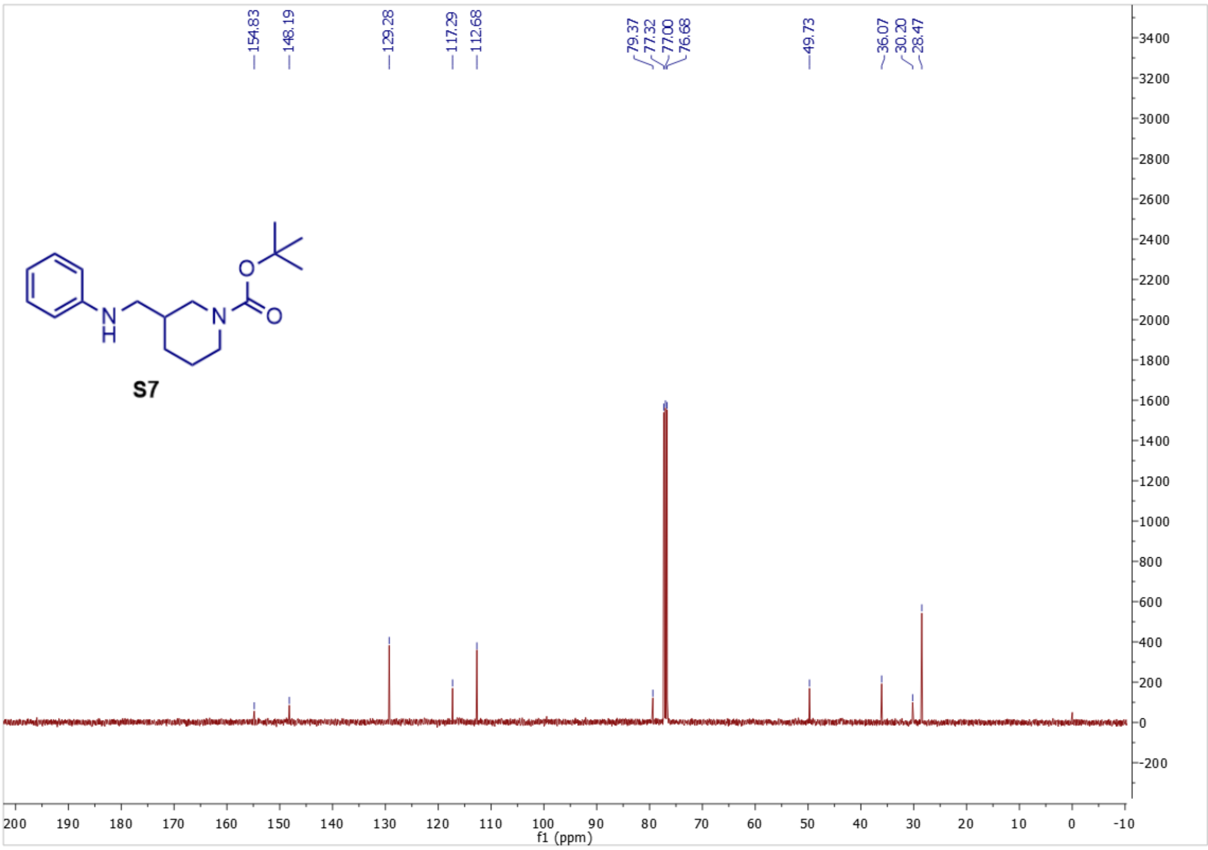
154.83
148.19
129.28
117.29
112.68
79.37
77.32
77.00
76.68
49.73
36.07
30.20
28.47
S7
f1 (ppm)

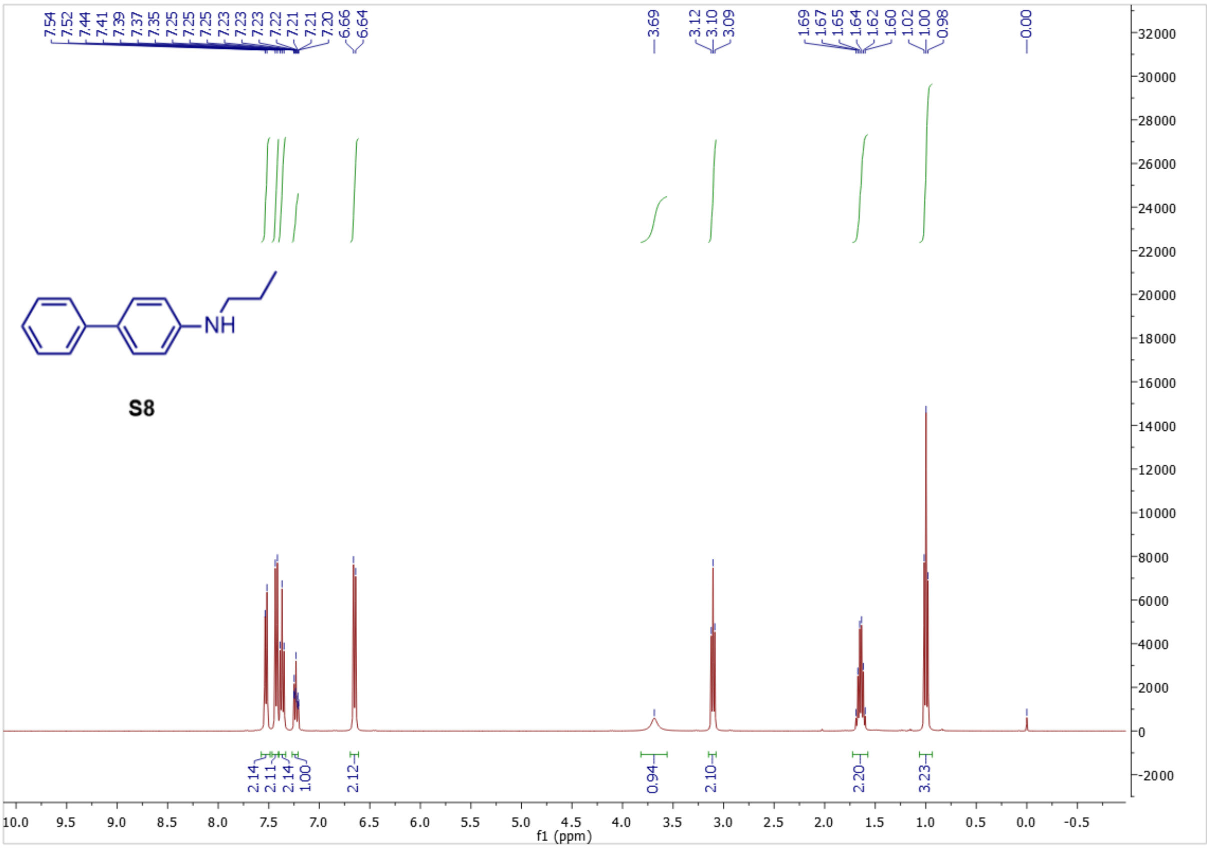

7.54
7.52
7.44
7.41
7.39
7.37
7.35
7.25
7.25
7.25
7.23
7.23
7.23
7.22
7.21
7.21
7.20
6.66
6.64
3.69
3.12
3.10
3.09
1.69
1.67
1.65
1.64
1.62
1.60
1.02
1.00
0.98
0.00
NH
2.14
2.11
2.14
1.00
2.12
0.94
2.10
2.20
3.23
f1 (ppm)

S8

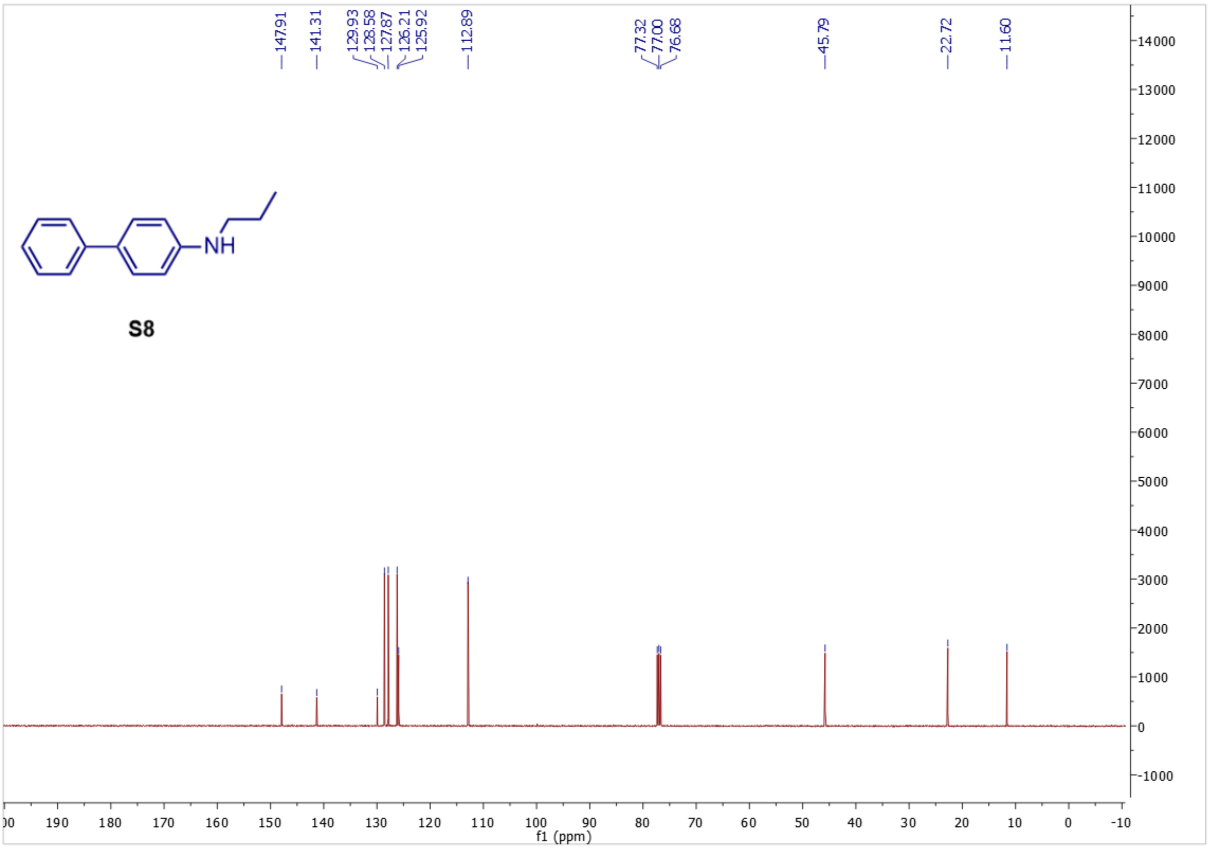

147.91
141.31
129.93
128.58
127.87
126.21
125.92
112.89
77.32
77.00
76.68
45.79
22.72
11.60
NH
f1 (ppm)

S8

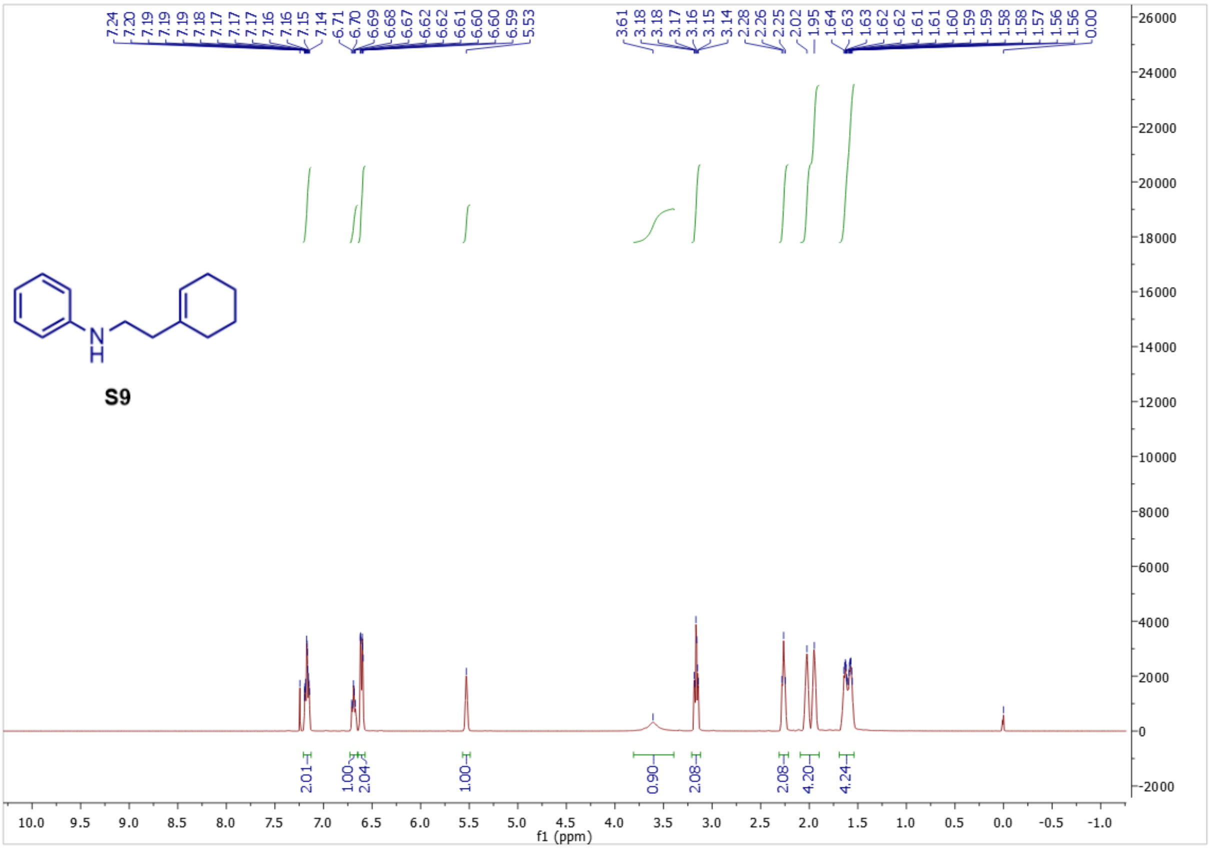
7.24
7.20
7.19
7.19
7.19
7.18
7.17
7.17
7.17
7.16
7.16
7.15
7.14
6.71
6.70
6.69
6.68
6.67
6.62
6.62
6.61
6.60
6.60
6.59
5.53
3.61
3.18
3.18
3.17
3.16
3.15
3.14
2.28
2.26
2.25
2.02
1.95
1.64
1.63
1.63
1.62
1.62
1.61
1.61
1.60
1.59
1.59
1.58
1.58
1.57
1.56
1.56
0.00
N
H
S9
2.01
1.00
2.04
1.00
0.90
2.08
2.08
4.20
4.24
f1 (ppm)

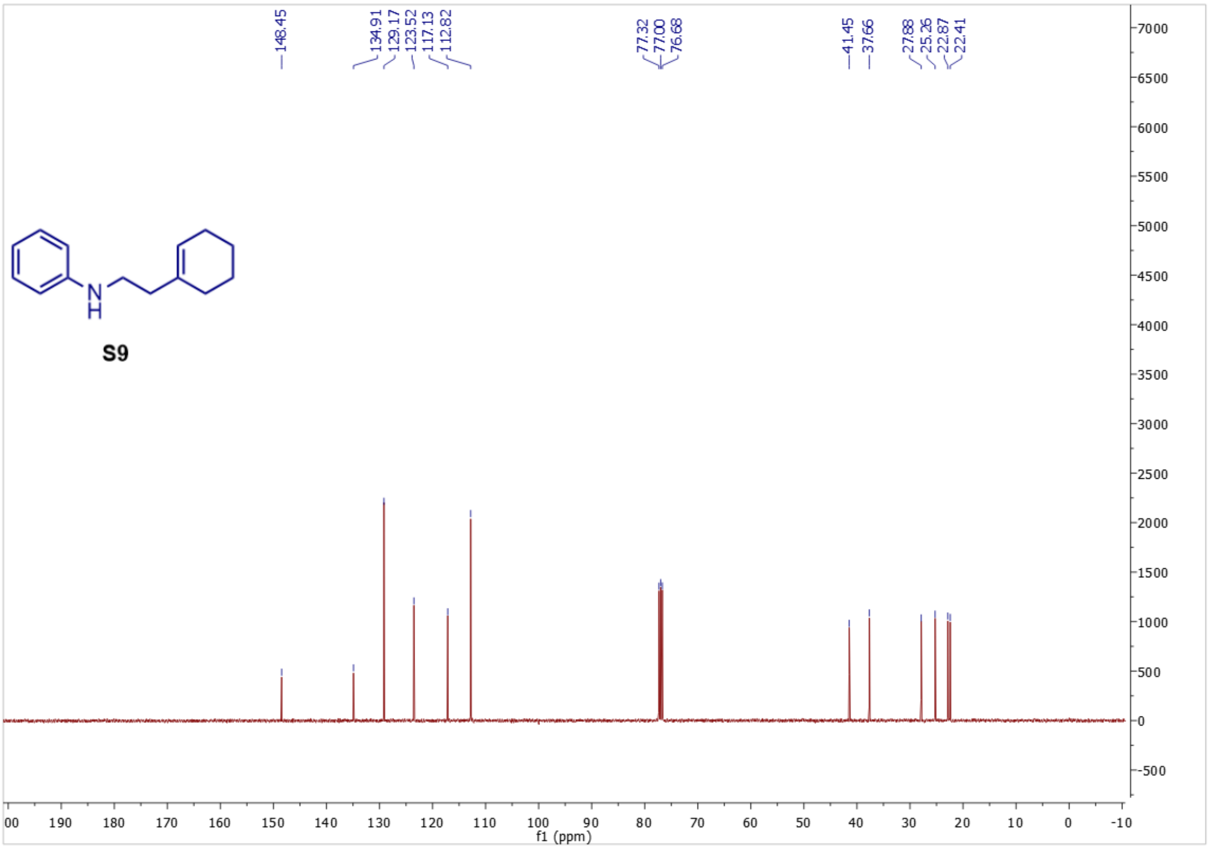
148.45
134.91
129.17
123.52
117.13
112.82
77.32
77.00
76.68
41.45
37.66
27.88
25.26
22.87
22.41
N
H
S9
f1 (ppm)

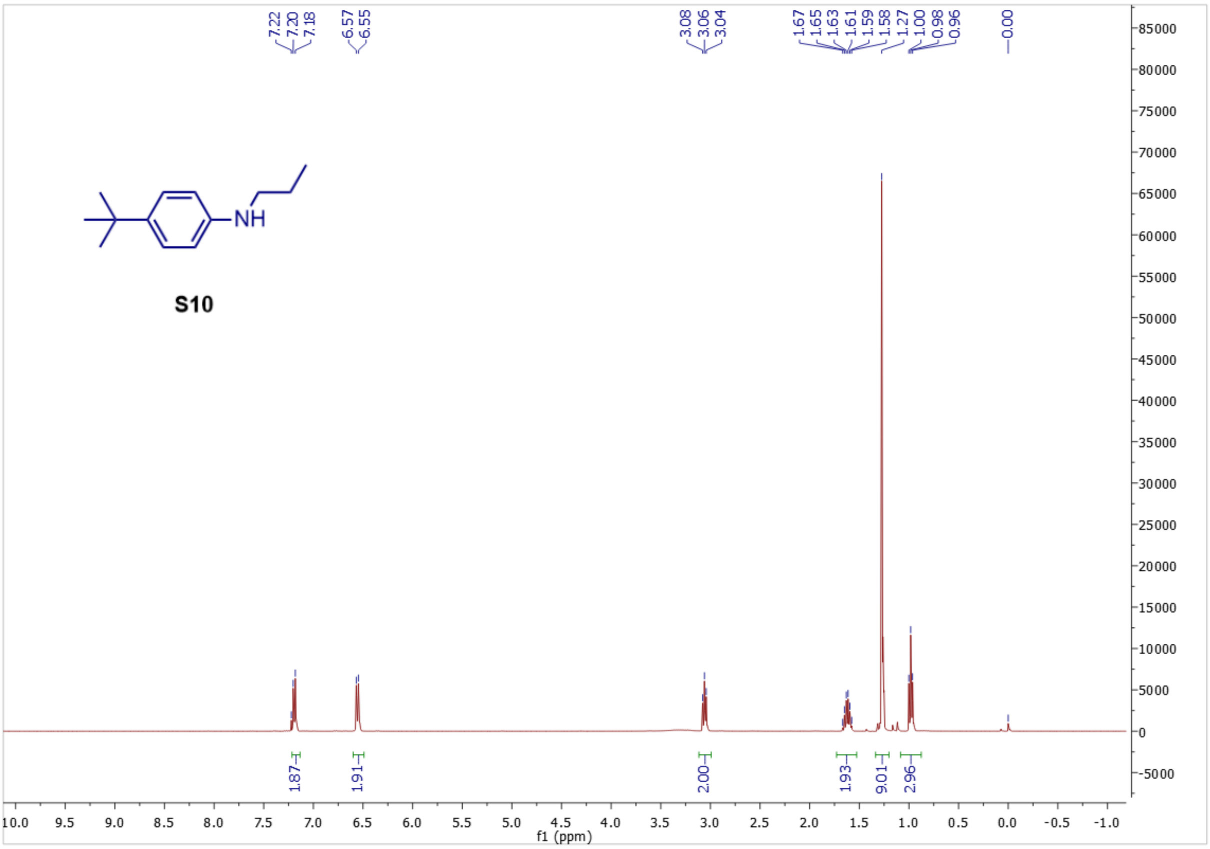

7.22
7.20
7.18
6.57
6.55
3.08
3.06
3.04
1.67
1.65
1.63
1.61
1.59
1.58
1.27
1.00
0.98
0.96
0.00
NH
S10
1.87
1.91
2.00
1.93
9.01
2.96
f1 (ppm)

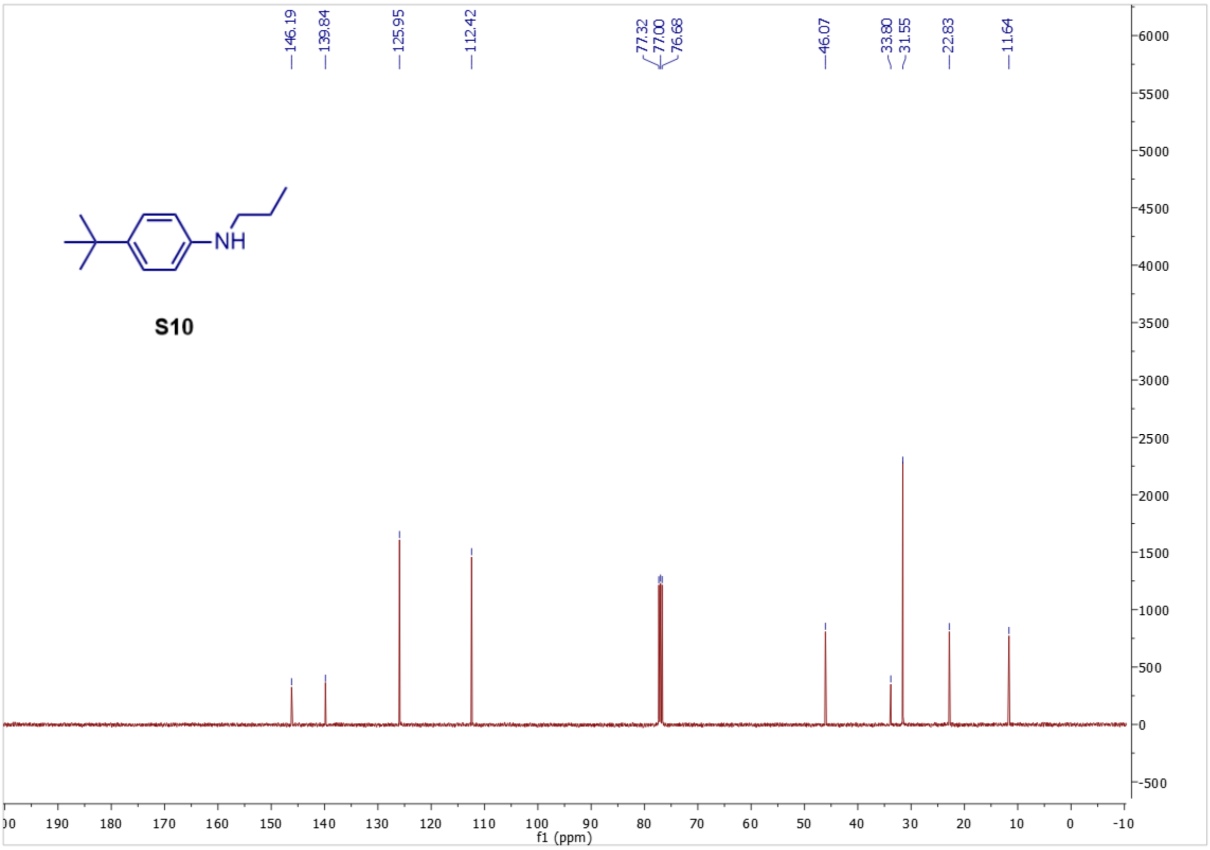

146.19
139.84
125.95
112.42
77.32
77.00
76.68
46.07
33.80
31.55
22.83
11.64
NH
S10
f1 (ppm)

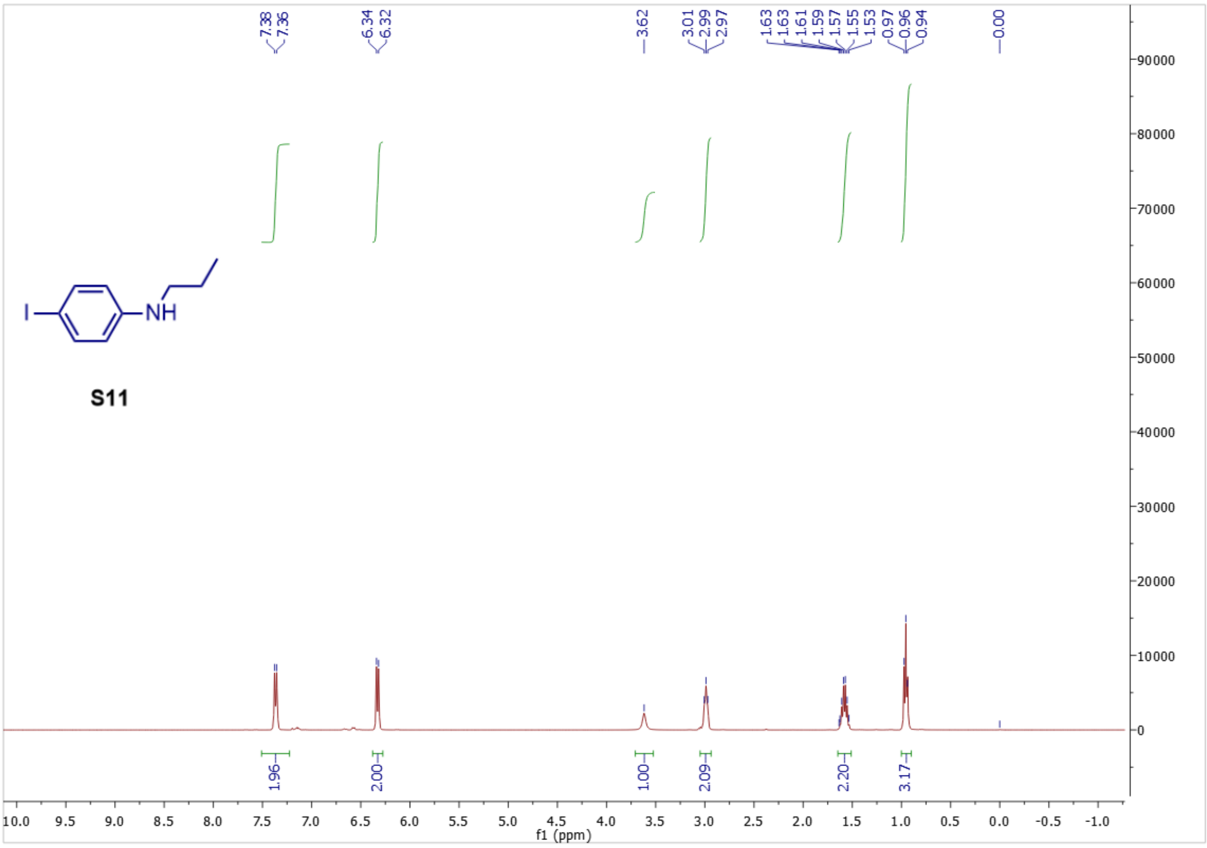

7.38
7.36
6.34
6.32
3.62
3.01
2.99
2.97
1.63
1.63
1.61
1.59
1.57
1.55
1.53
0.97
0.96
0.94
0.00
S11
1.96
2.00
1.00
2.09
2.20
3.17
f1 (ppm)

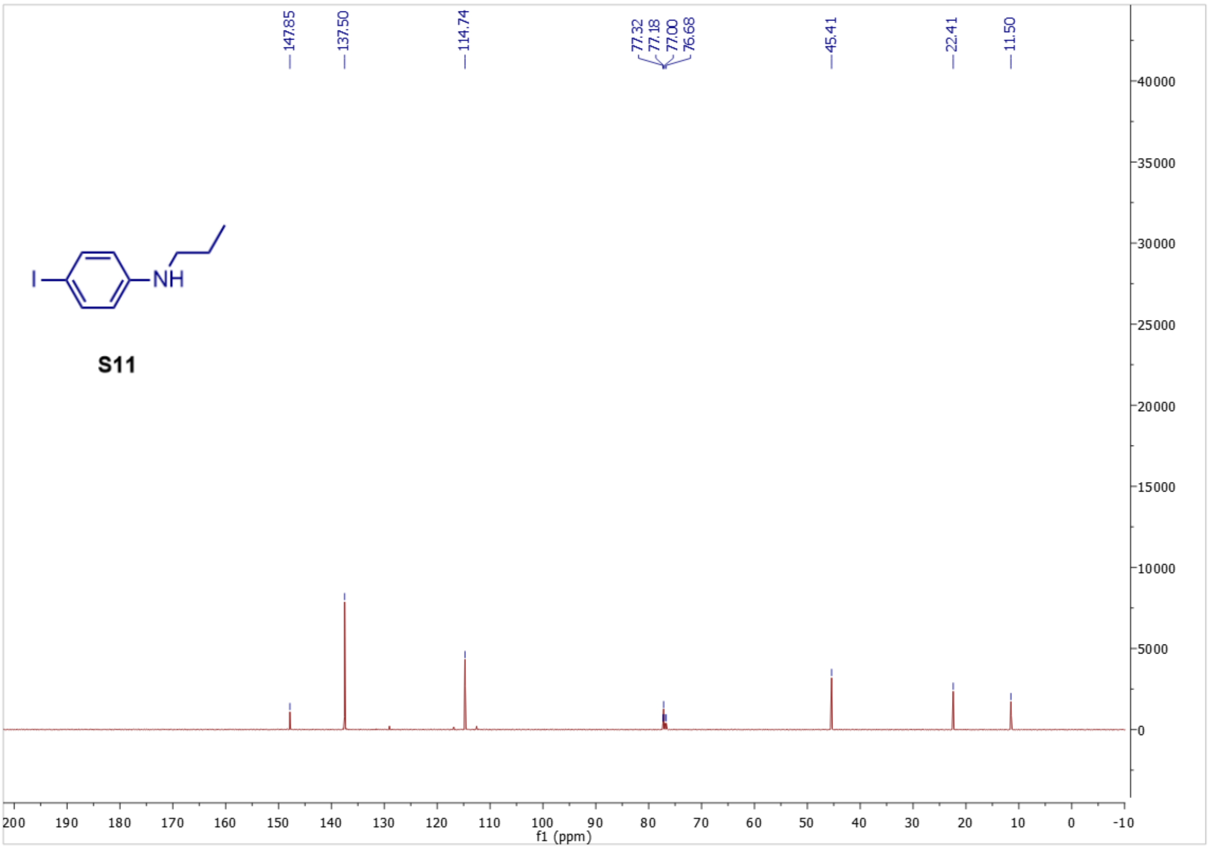

147.85
137.50
114.74
77.32
77.18
77.00
76.68
45.41
22.41
11.50
S11
f1 (ppm)

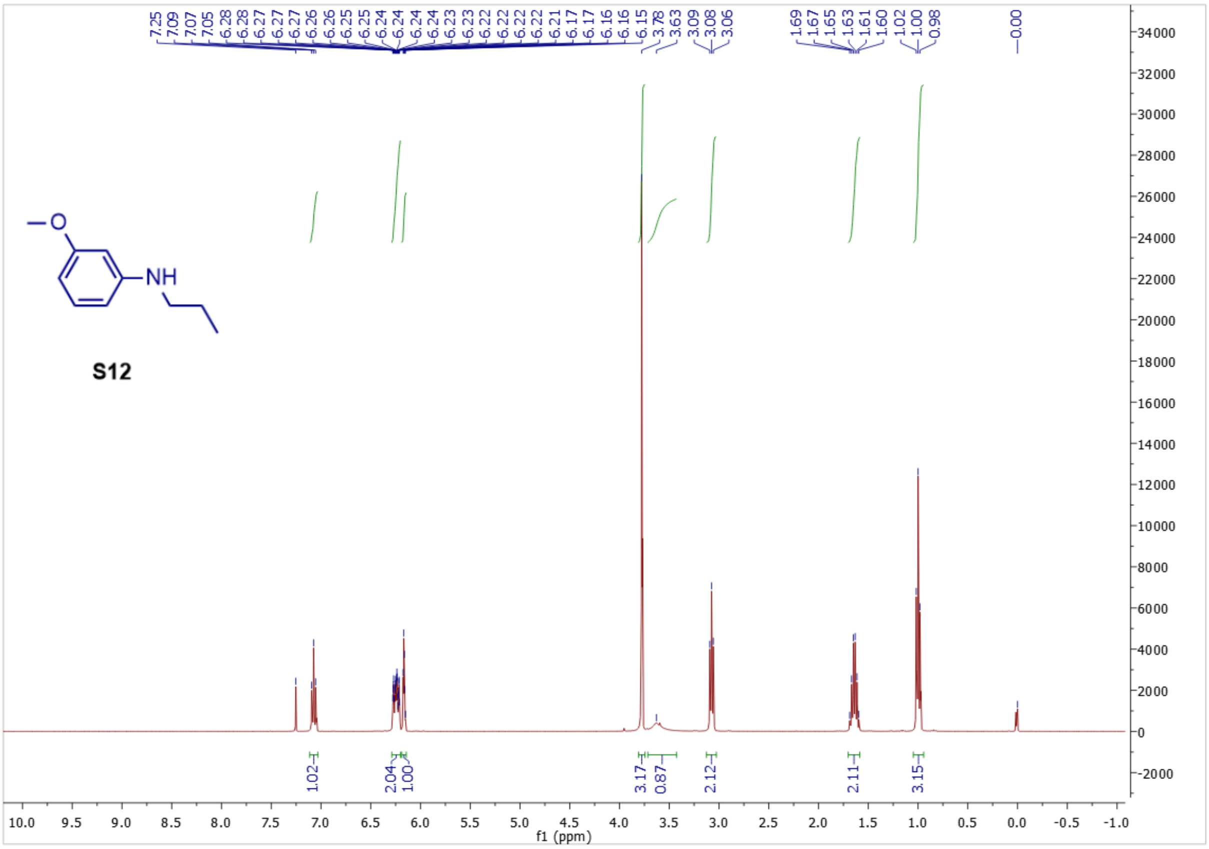

7.25
7.09
7.07
7.05
6.28
6.28
6.27
6.27
6.27
6.26
6.26
6.25
6.25
6.24
6.24
6.24
6.24
6.23
6.23
6.22
6.22
6.22
6.22
6.21
6.17
6.17
6.16
6.16
6.15
3.78
3.63
3.09
3.08
3.06
1.69
1.67
1.65
1.63
1.61
1.60
1.02
1.00
0.98
0.00
NH
S12
1.02
2.04
1.00
3.17
0.87
2.12
2.11
3.15
f1 (ppm)

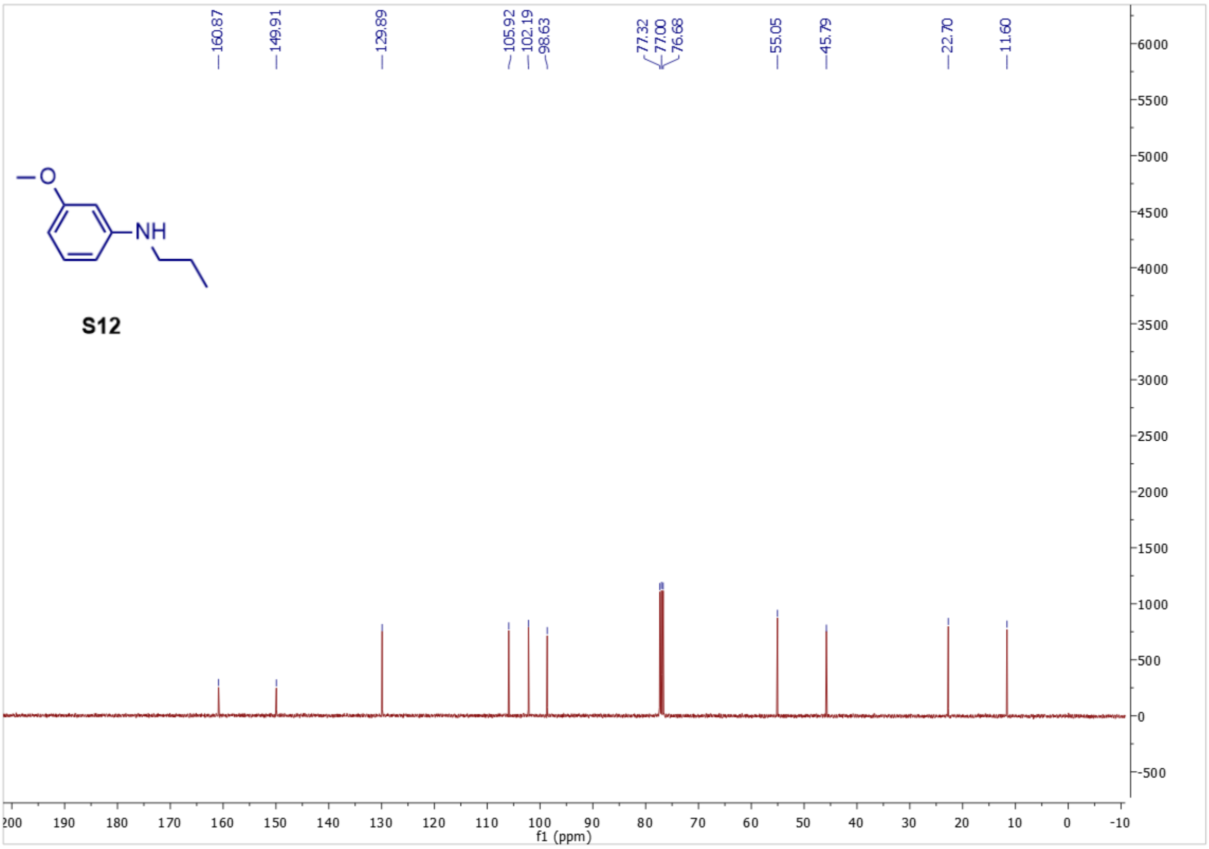

160.87
149.91
129.89
105.92
102.19
98.63
77.32
77.00
76.68
55.05
45.79
22.70
11.60
NH
S12
f1 (ppm)

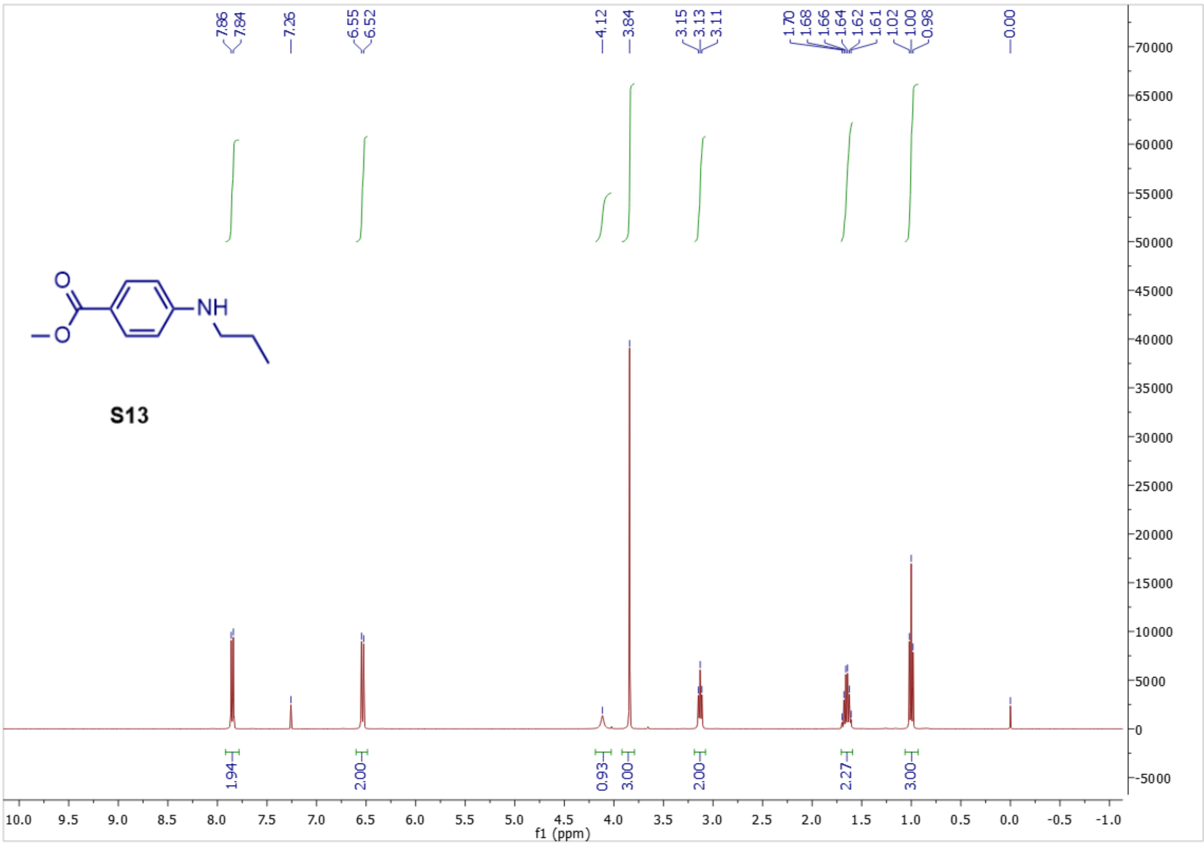
7.86
7.84
7.26
6.55
6.52
4.12
3.84
3.15
3.13
3.11
1.70
1.68
1.66
1.64
1.62
1.61
1.02
1.00
0.98
0.00
NH
O
O
S13
1.94
2.00
0.93
3.00
2.00
2.27
3.00
f1 (ppm)

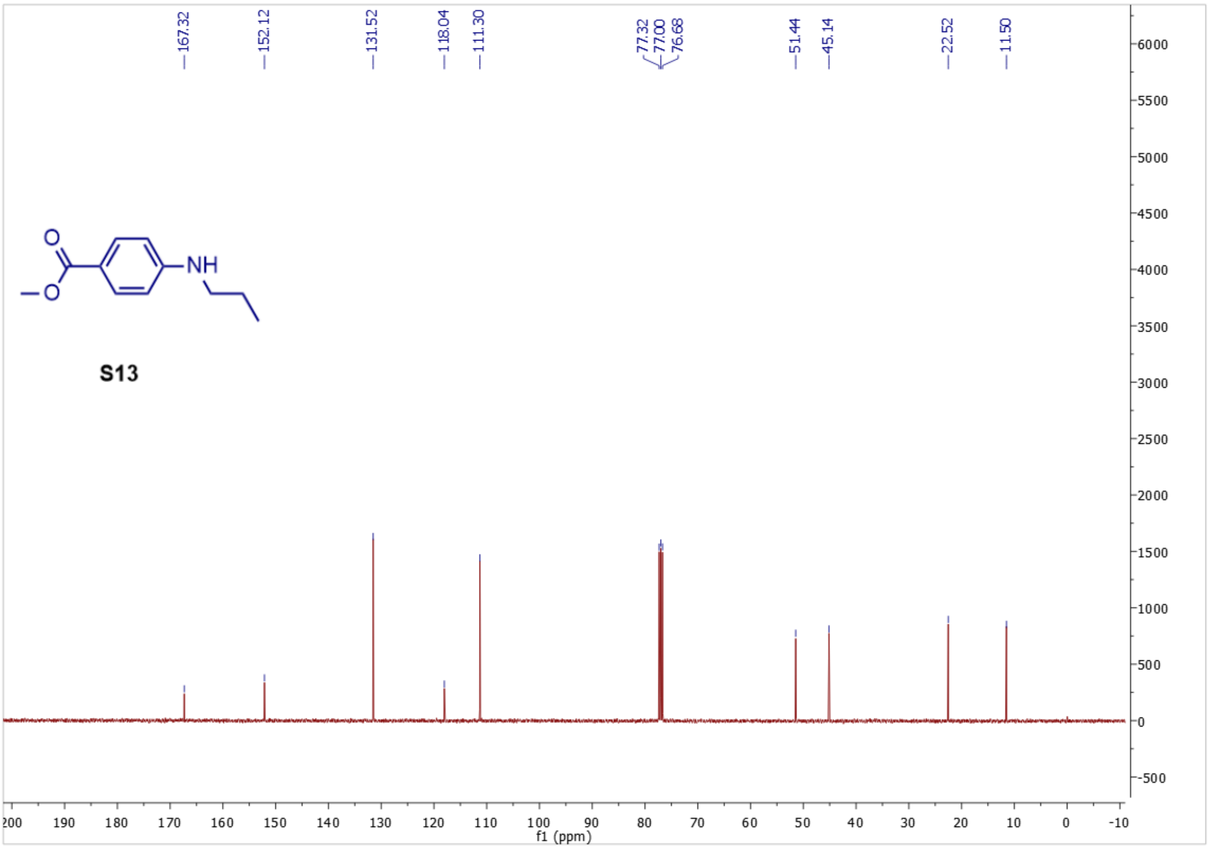
167.32
152.12
131.52
118.04
111.30
77.32
77.00
76.68
51.44
45.14
22.52
11.50
NH
O
O
S13
f1 (ppm)

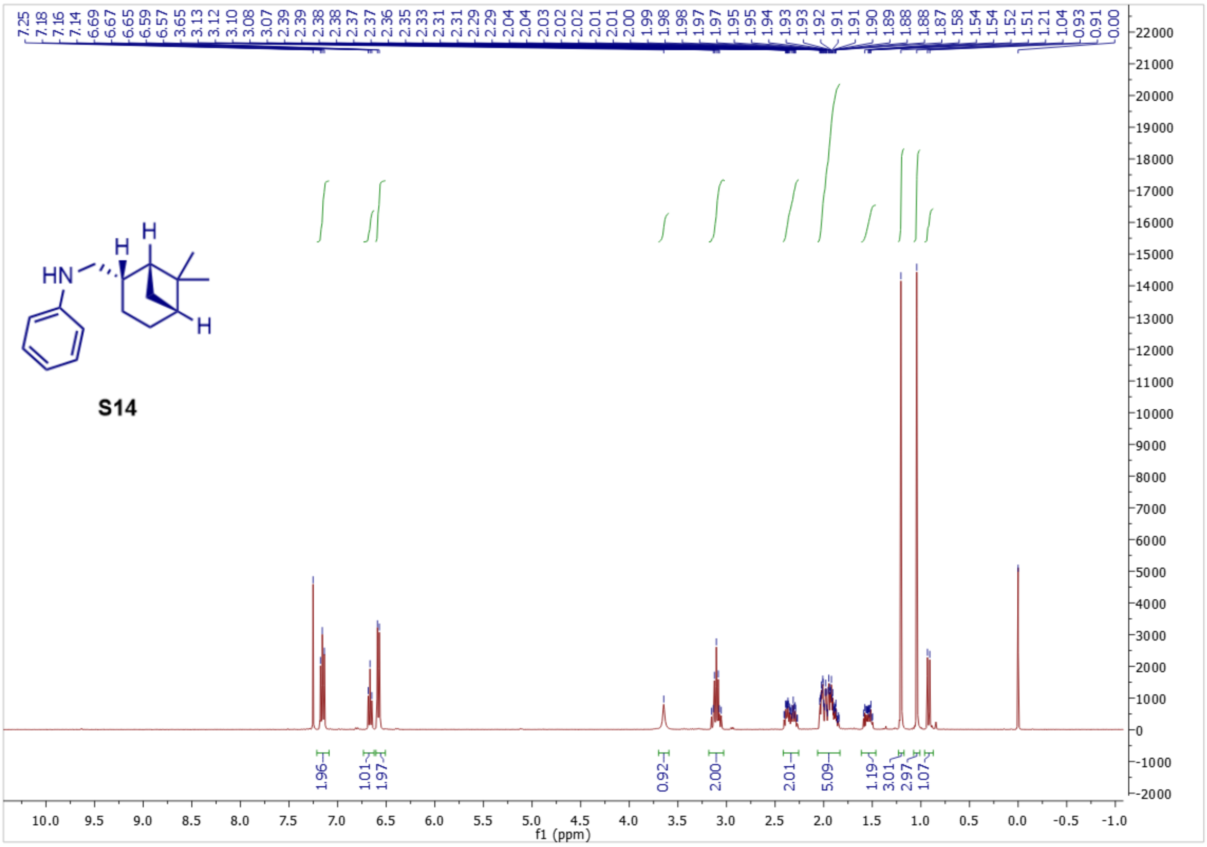
7.25 7.18 7.16 7.14 6.69 6.67 6.65 6.59 6.57 3.65 3.13 3.12 3.10 3.08 3.07 2.39 2.39 2.38 2.38 2.37 2.37 2.36 2.35 2.33 2.31 2.31 2.29 2.29 2.04 2.04 2.03 2.02 2.02 2.01 2.01 2.00 1.99 1.98 1.98 1.97 1.97 1.95 1.95 1.94 1.93 1.93 1.92 1.91 1.91 1.90 1.89 1.88 1.88 1.87 1.58 1.54 1.54 1.52 1.51 1.21 1.04 0.93 0.91 0.00
HN
H
H
H
S14
1.96
1.01
1.97
0.92
2.00
2.01
5.09
1.19
3.01
2.97
1.07
f1 (ppm)

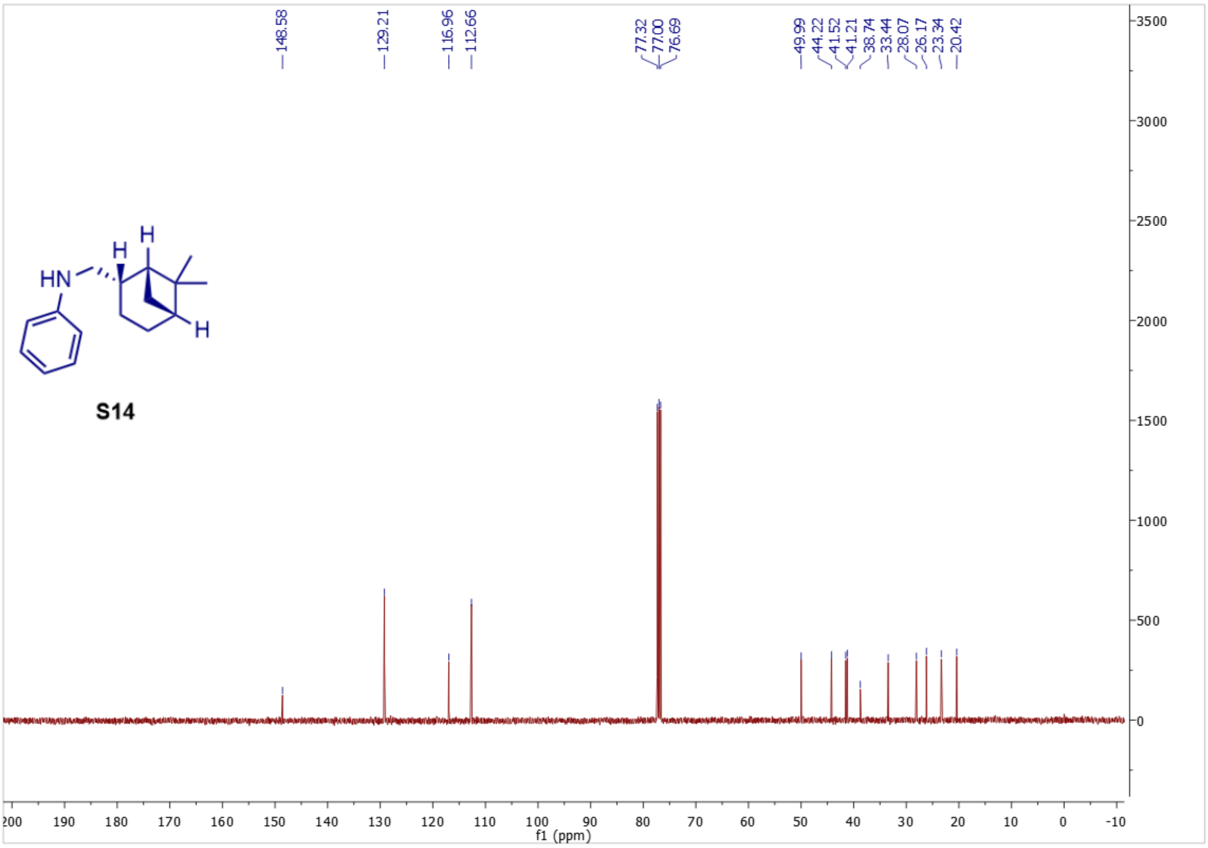
148.58
129.21
116.96
112.66
77.32 77.00 76.69
49.99 44.22 41.52 41.21 38.74 33.44 28.07 26.17 23.34 20.42
HN
H
H
H
S14
f1 (ppm)

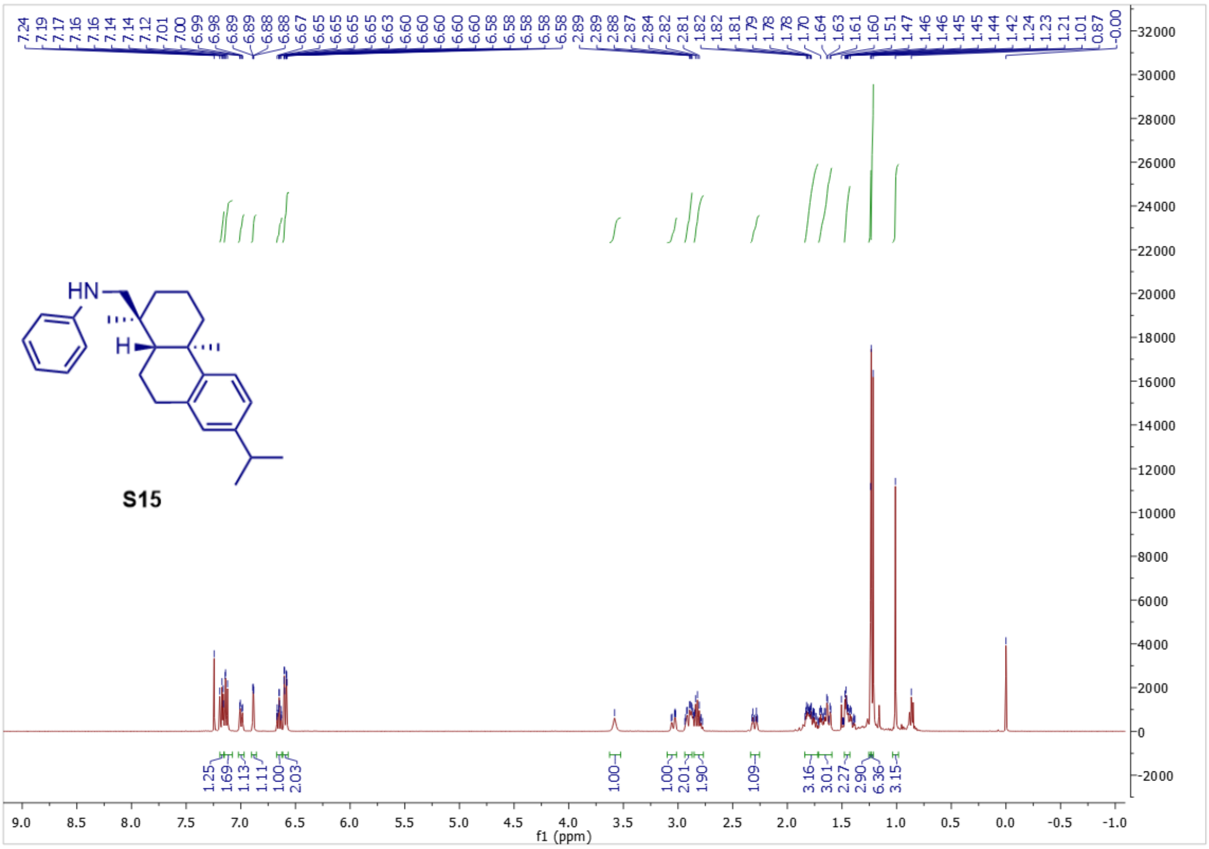

S15

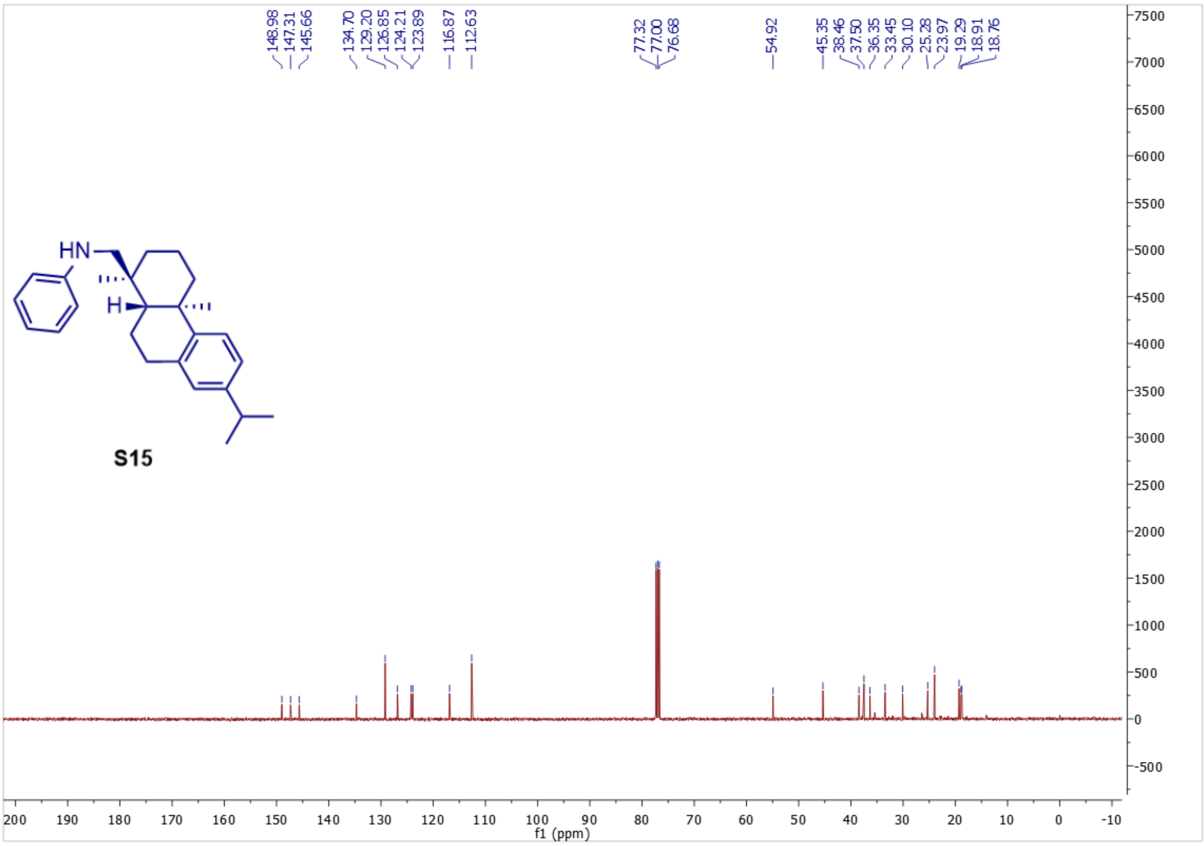

S15

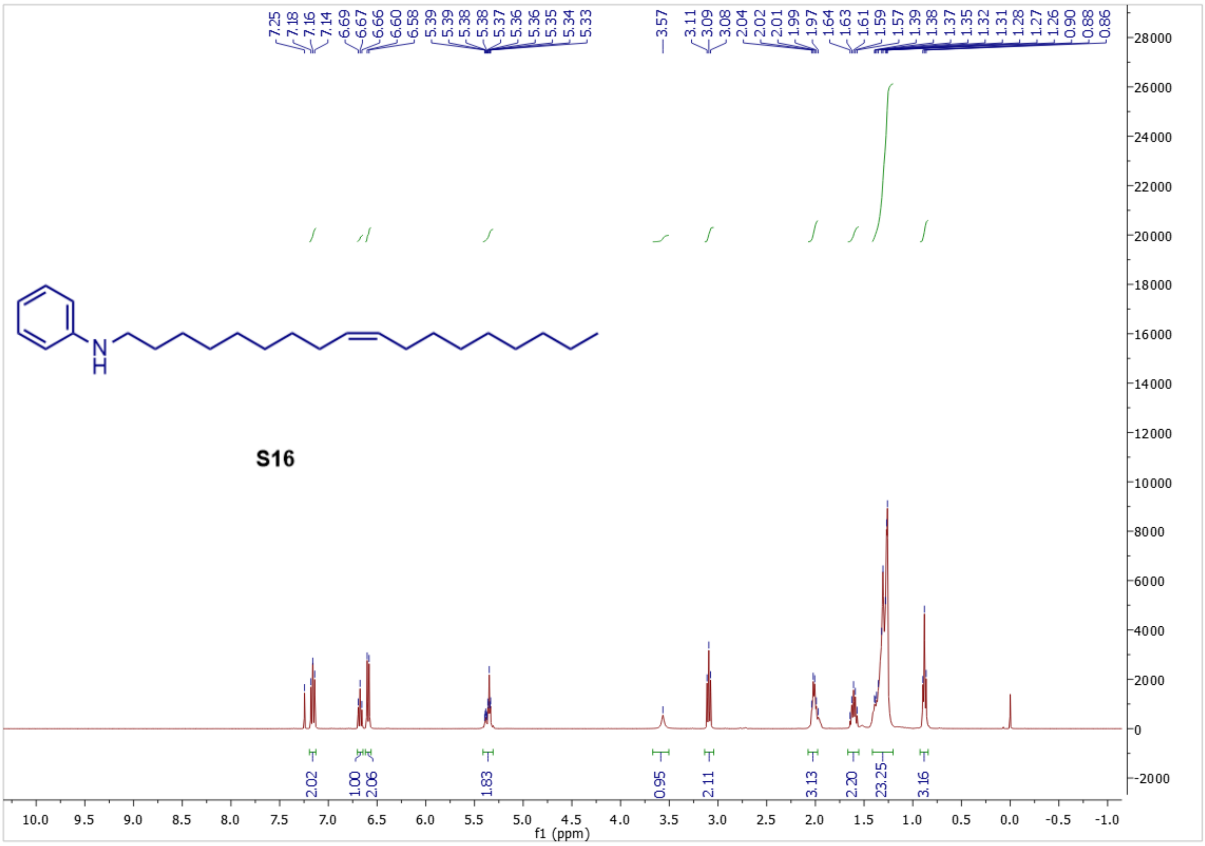

7.25
7.18
7.16
7.14
6.69
6.67
6.66
6.60
6.58
5.39
5.39
5.38
5.38
5.37
5.36
5.35
5.34
5.33
3.57
3.11
3.09
3.08
2.04
2.02
2.01
1.99
1.97
1.64
1.63
1.61
1.59
1.57
1.39
1.38
1.37
1.35
1.32
1.31
1.28
1.27
1.26
0.90
0.88
0.86
N
H
S16
2.02
1.00
2.06
1.83
0.95
2.11
3.13
2.20
23.25
3.16
f1 (ppm)

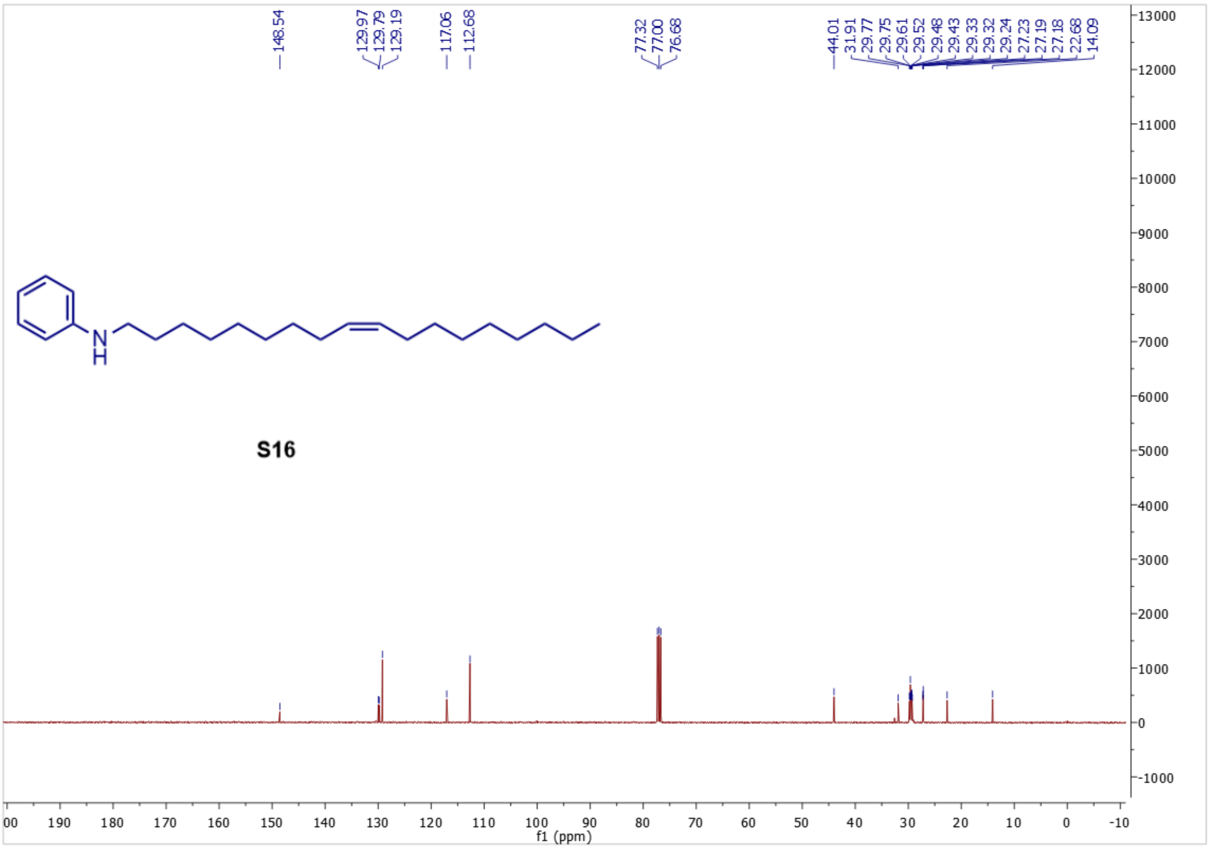

148.54
129.97
129.79
129.19
117.06
112.68
77.32
77.00
76.68
44.01
31.91
29.77
29.75
29.61
29.52
29.48
29.43
29.33
29.32
29.24
27.23
27.19
27.18
22.68
14.09
N
H
S16
f1 (ppm)

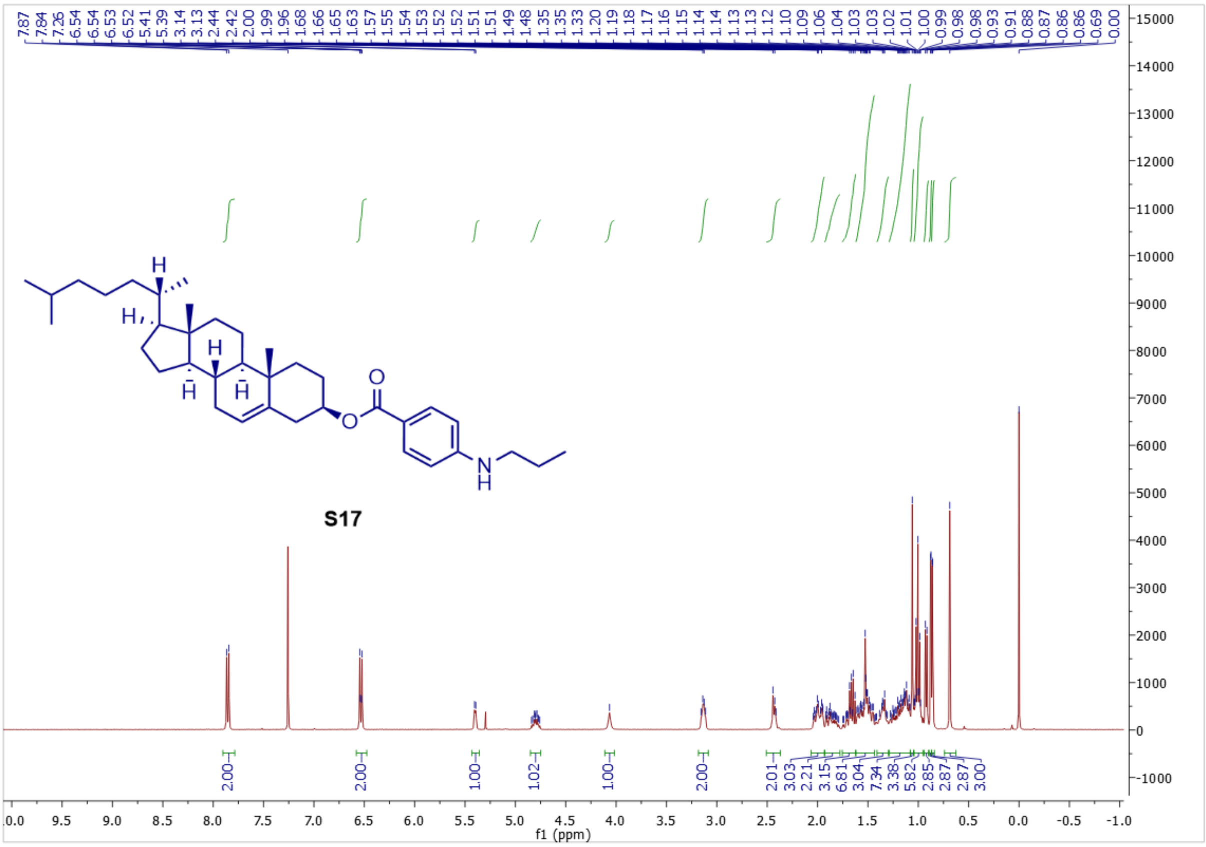
S17
f1 (ppm)

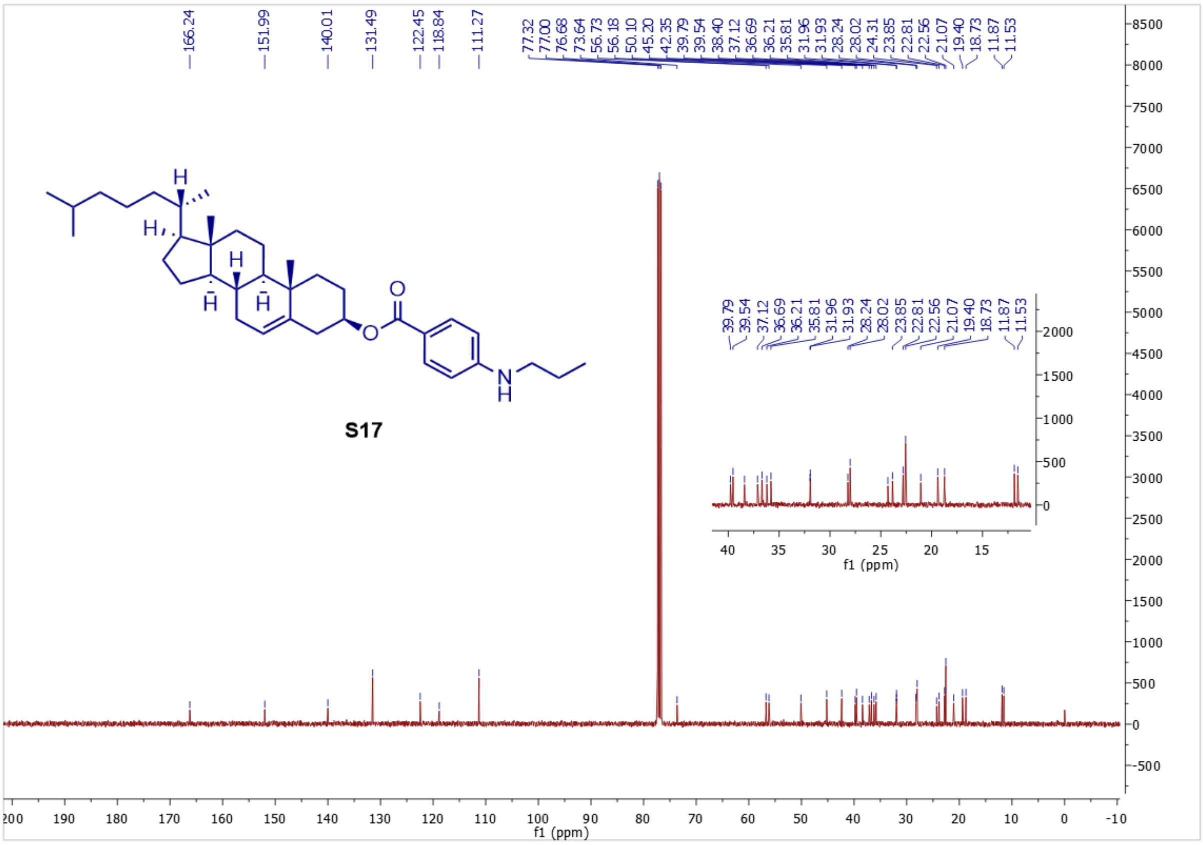
S17
f1 (ppm)

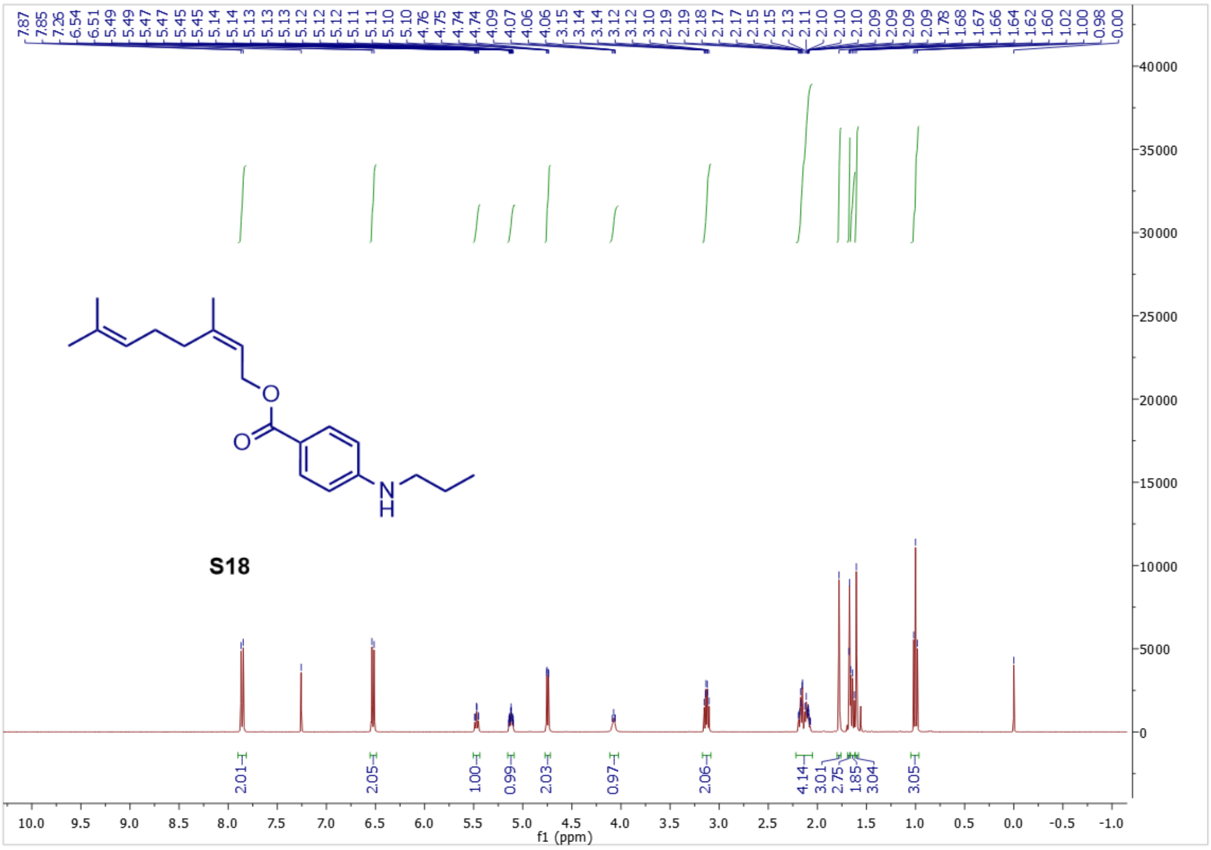

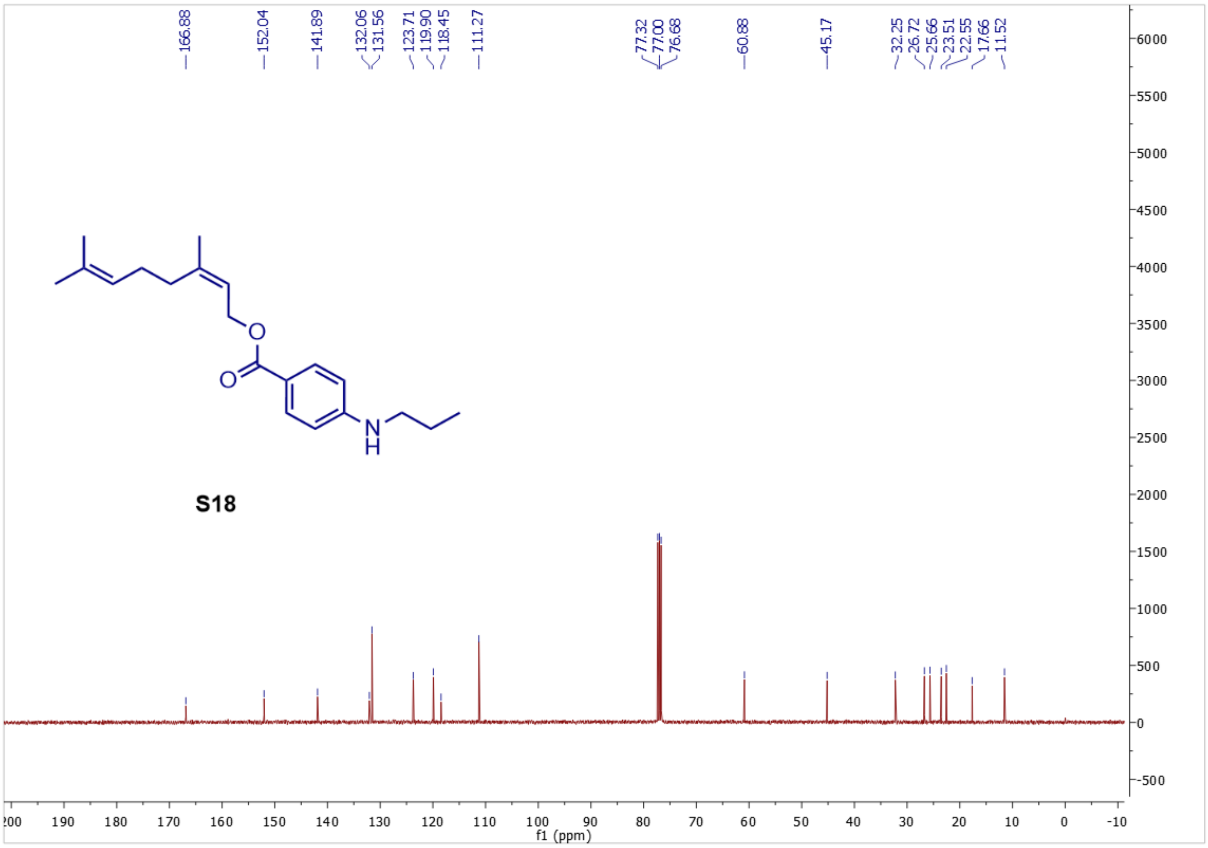

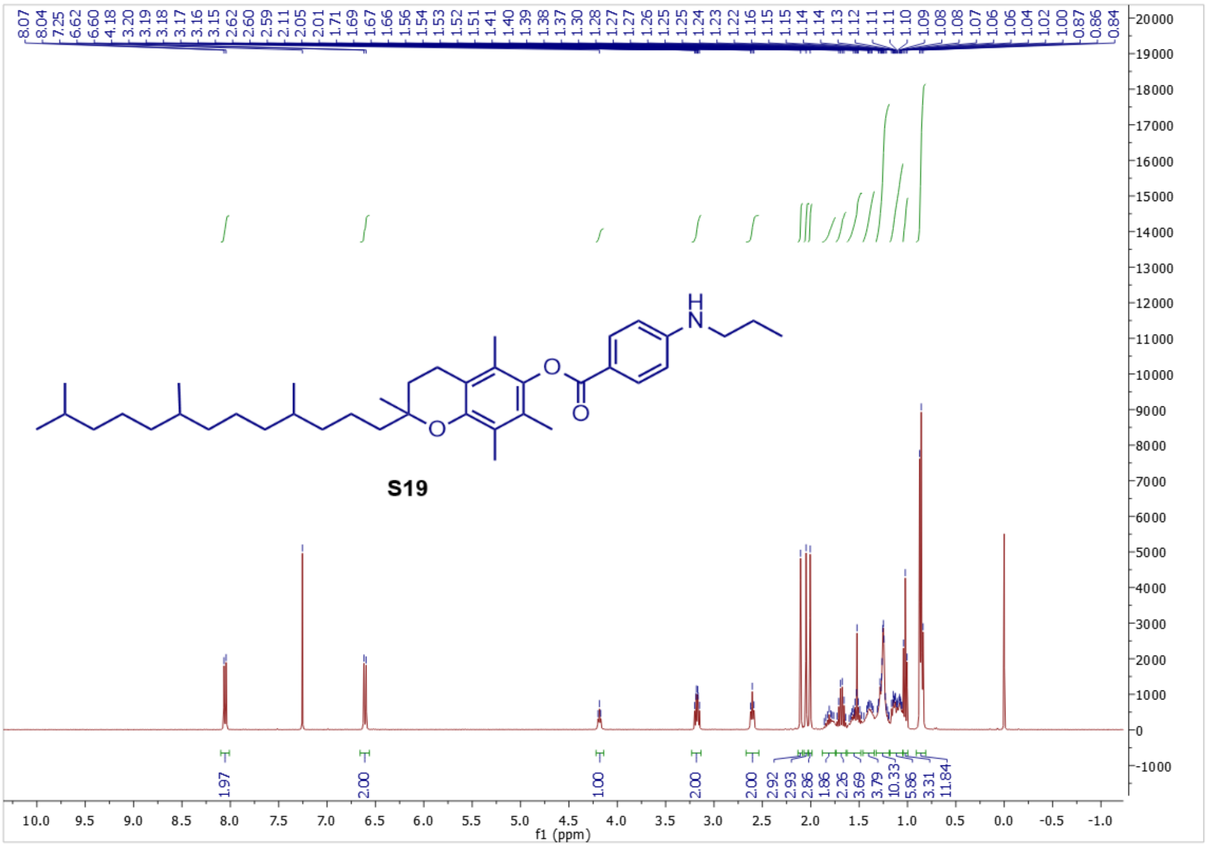

8.07
8.04
7.25
6.62
6.60
4.18
3.20
3.19
3.18
3.17
3.16
3.15
2.62
2.60
2.59
2.11
2.05
2.01
1.71
1.69
1.67
1.66
1.56
1.54
1.53
1.52
1.51
1.41
1.40
1.39
1.38
1.37
1.30
1.28
1.27
1.27
1.26
1.25
1.25
1.24
1.23
1.22
1.16
1.15
1.15
1.14
1.14
1.13
1.12
1.11
1.11
1.10
1.09
1.08
1.08
1.07
1.06
1.06
1.04
1.02
1.00
0.87
0.86
0.84
S19
1.97
2.00
1.00
2.00
2.00
2.92
2.93
2.86
1.86
2.26
3.69
3.79
10.33
5.86
3.31
11.84
f1 (ppm)

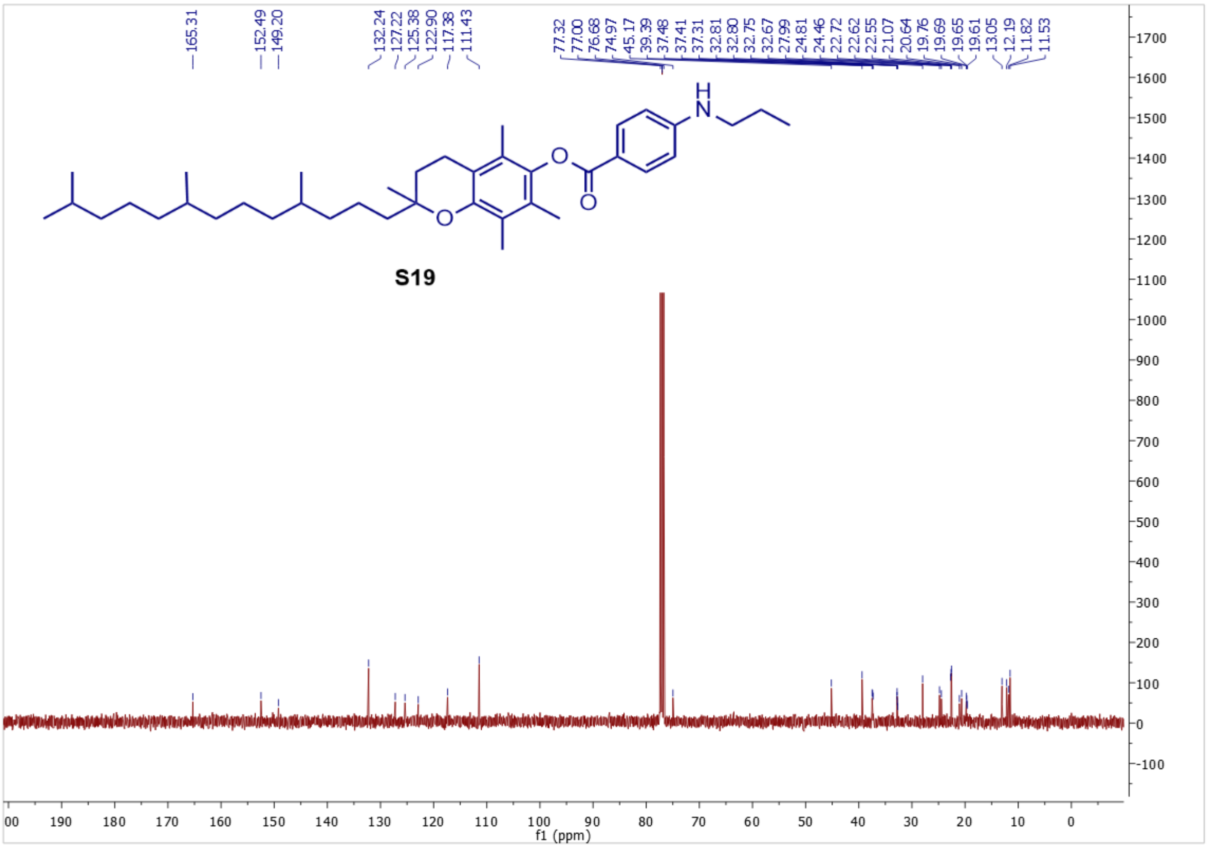

165.31
152.49
149.20
132.24
127.22
125.38
122.90
117.38
111.43
77.32
77.00
76.68
74.97
45.17
39.39
37.48
37.41
37.31
32.81
32.80
32.75
32.67
27.99
24.81
24.46
22.72
22.62
22.55
21.07
20.64
19.76
19.69
19.65
19.61
13.05
12.19
11.82
11.53
S19
f1 (ppm)

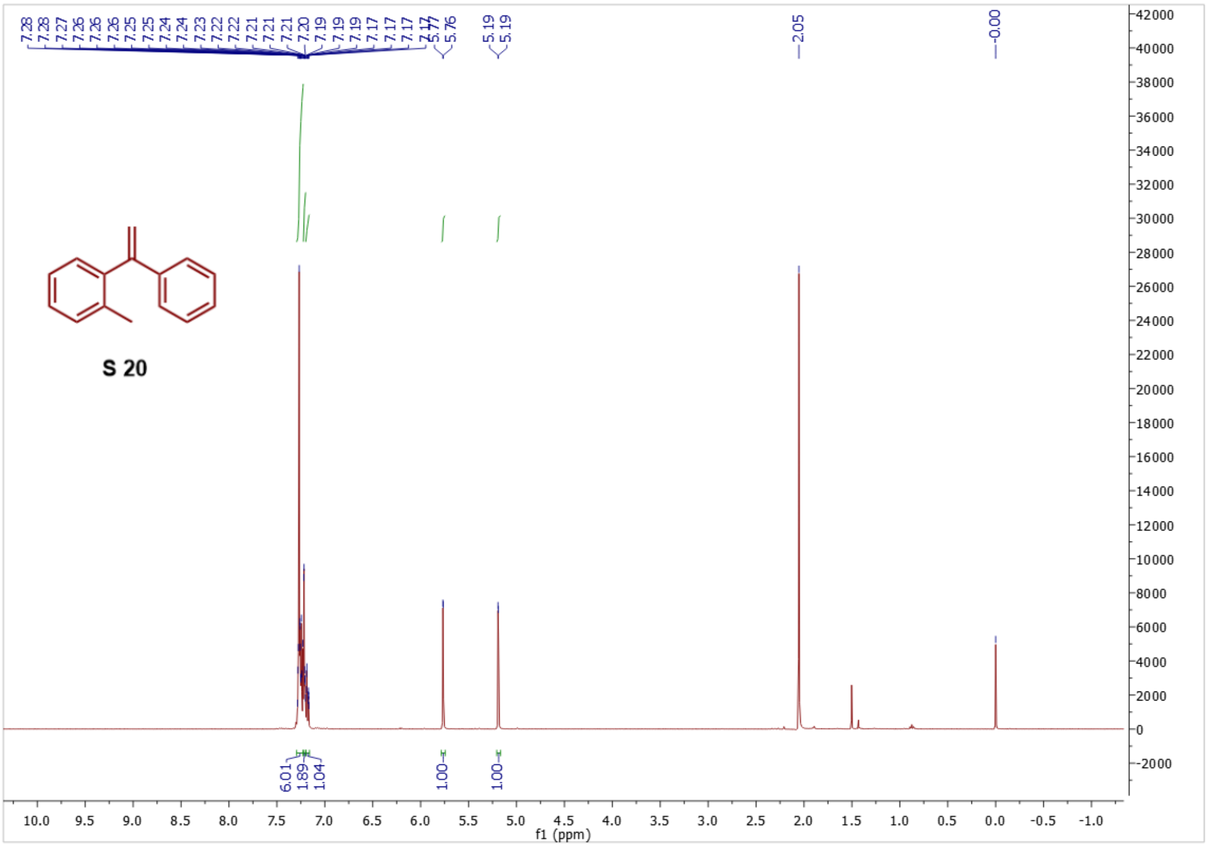

7.28
7.28
7.27
7.26
7.26
7.26
7.25
7.25
7.24
7.24
7.23
7.22
7.22
7.21
7.21
7.21
7.20
7.19
7.19
7.19
7.17
7.17
7.17
5.77
5.76
5.19
5.19
2.05
0.00
S 20
6.01
1.89
1.04
1.00
1.00
f1 (ppm)

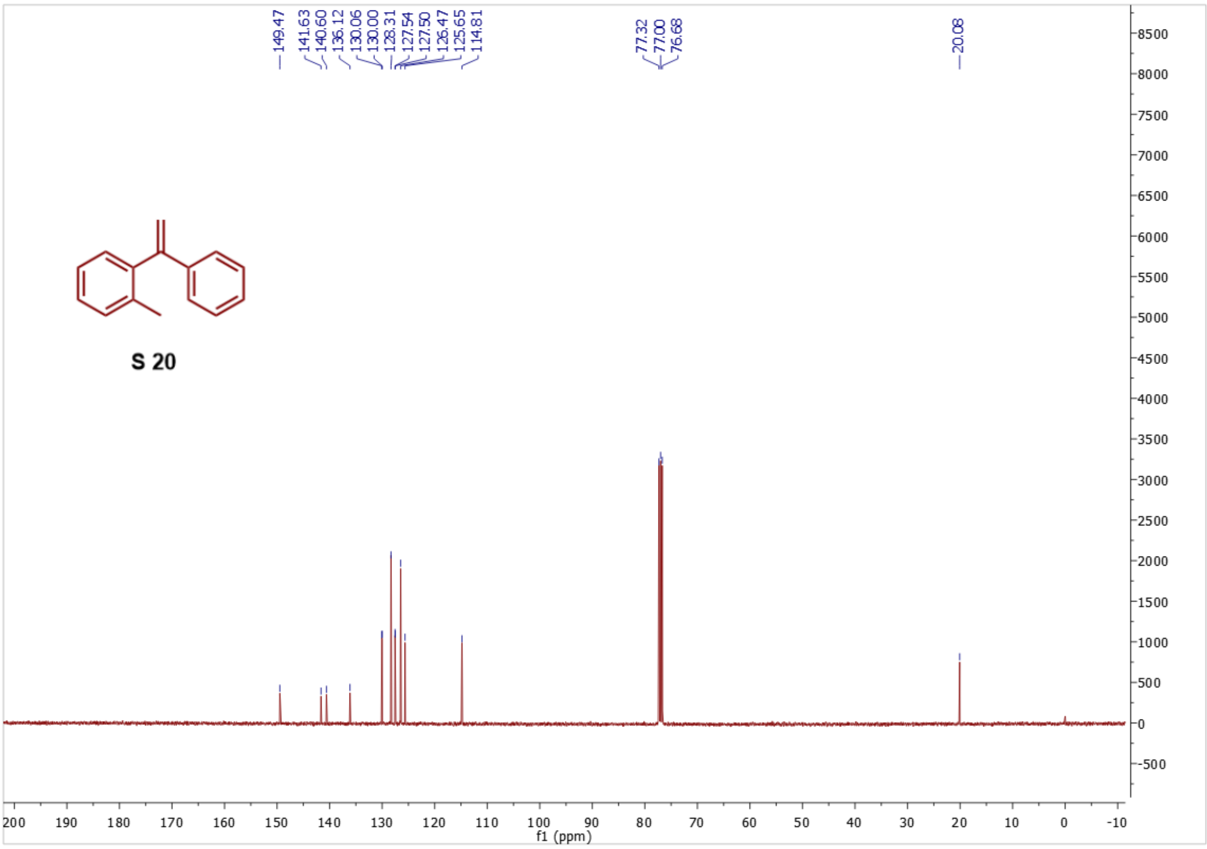

149.47
141.63
140.60
136.12
130.06
130.00
128.31
127.54
127.50
126.47
125.65
114.81
77.32
77.00
76.68
20.08
S 20
f1 (ppm)

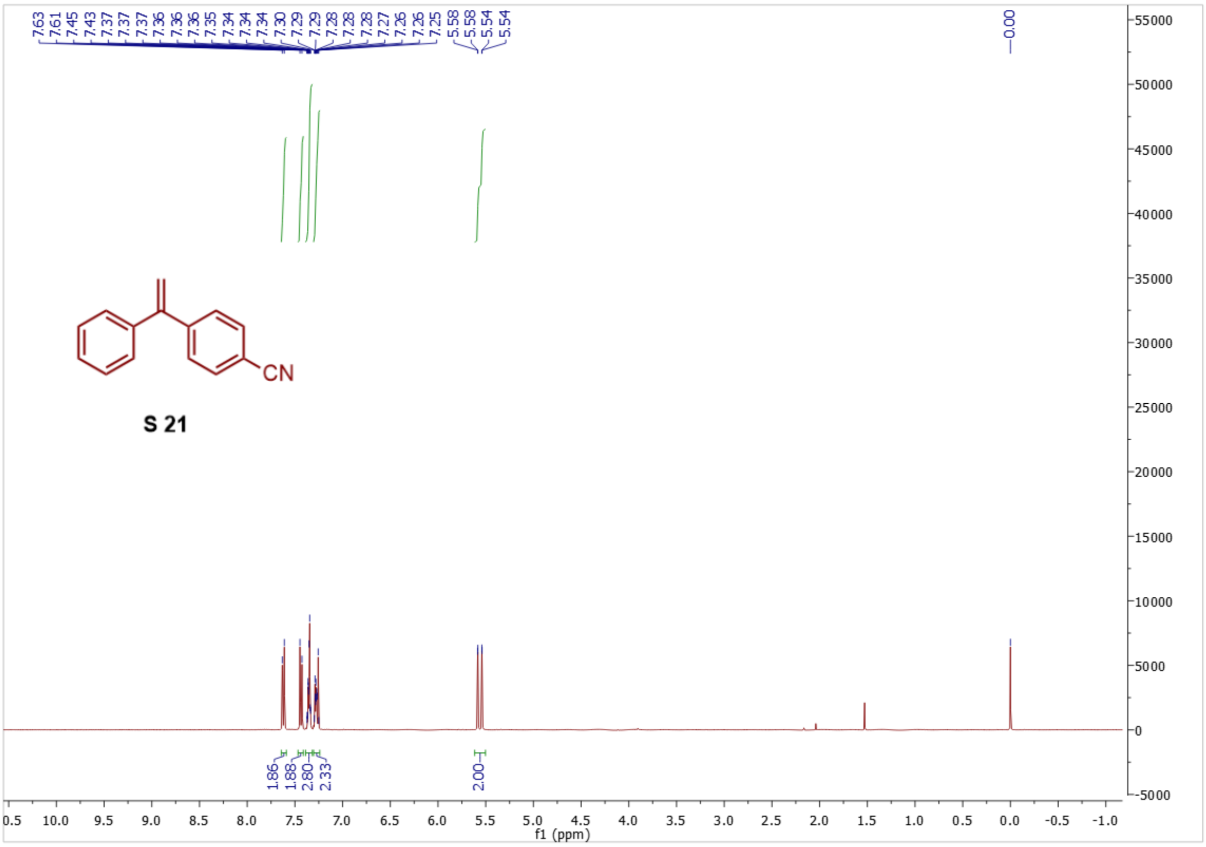
7.63
7.61
7.45
7.43
7.37
7.37
7.37
7.36
7.36
7.36
7.35
7.34
7.34
7.34
7.30
7.29
7.29
7.28
7.28
7.28
7.27
7.26
7.26
7.25
5.58
5.58
5.54
5.54
0.00
S 21
1.86
1.88
2.80
2.33
2.00
f1 (ppm)

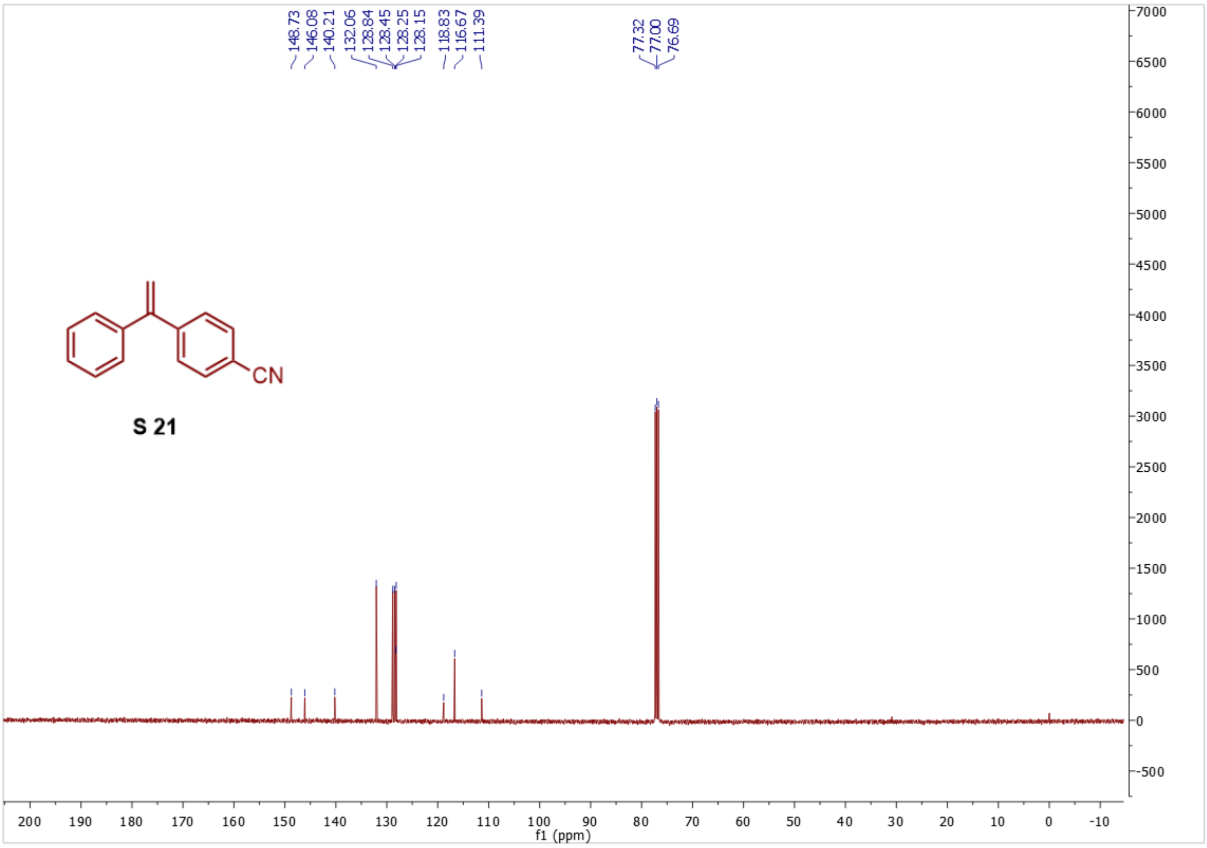
148.73
146.08
140.21
132.06
128.84
128.45
128.25
128.15
118.83
116.67
111.39
77.32
77.00
76.69
S 21
f1 (ppm)

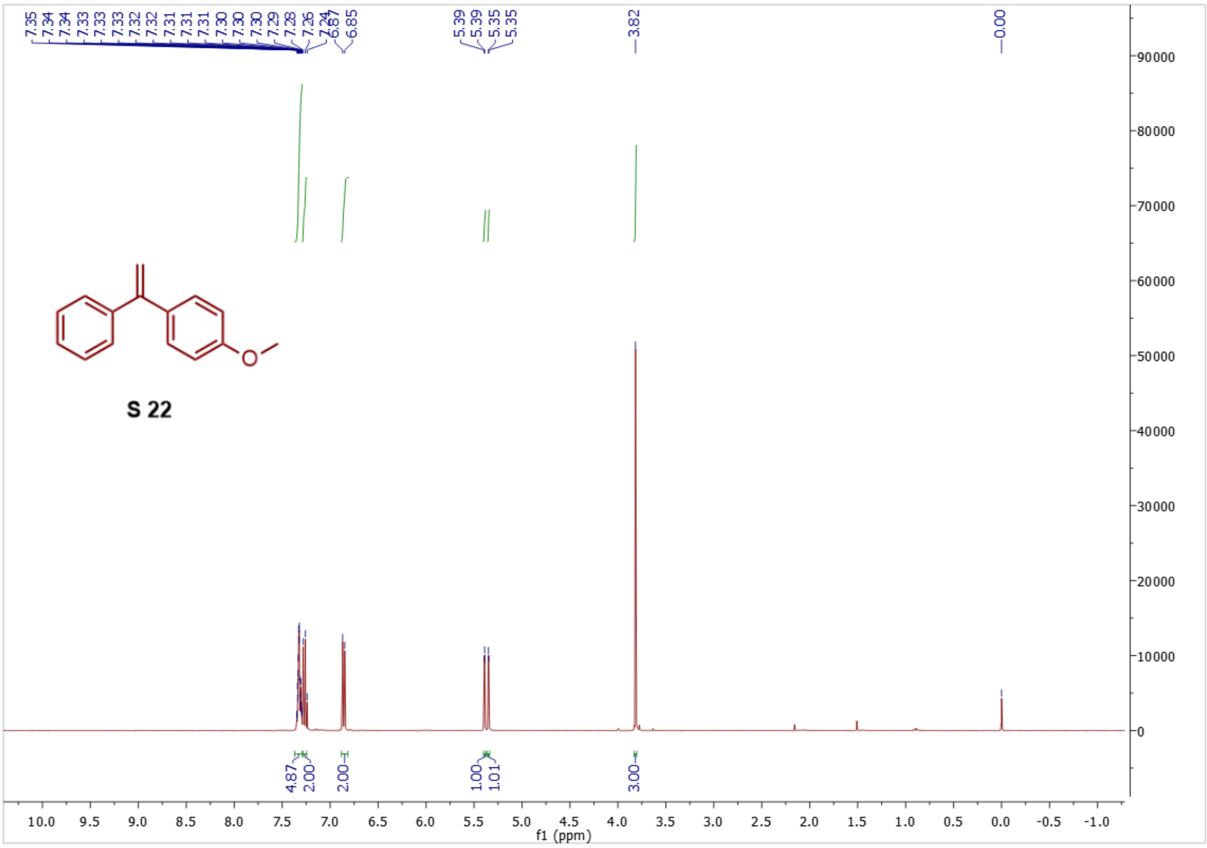
7.35
7.34
7.34
7.33
7.33
7.33
7.32
7.32
7.31
7.31
7.31
7.30
7.30
7.30
7.29
7.28
7.26
7.24
6.87
6.85
5.39
5.39
5.35
5.35
3.82
0.00
S 22
4.87
2.00
2.00
1.00
1.01
3.00
f1 (ppm)

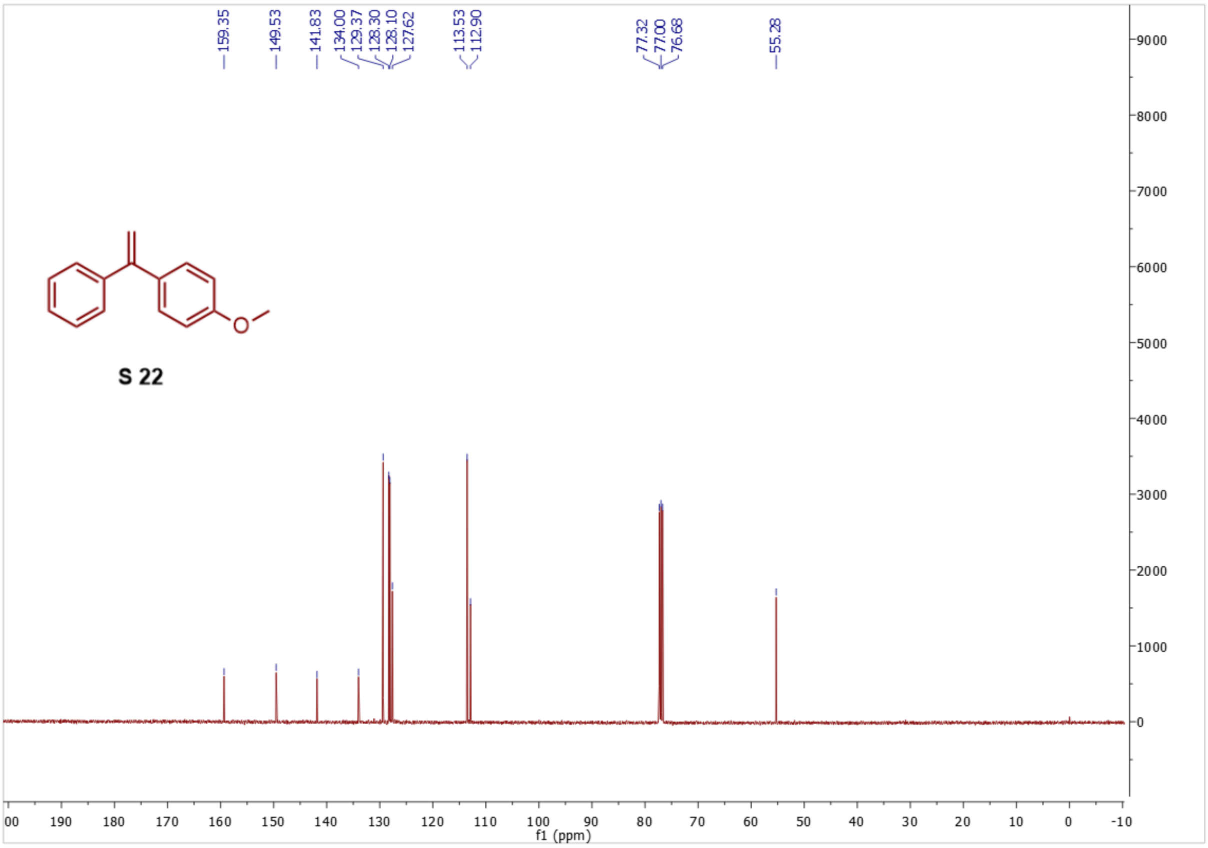
159.35
149.53
141.83
134.00
129.37
128.30
128.10
127.62
113.53
112.90
77.32
77.00
76.68
55.28
S 22
f1 (ppm)

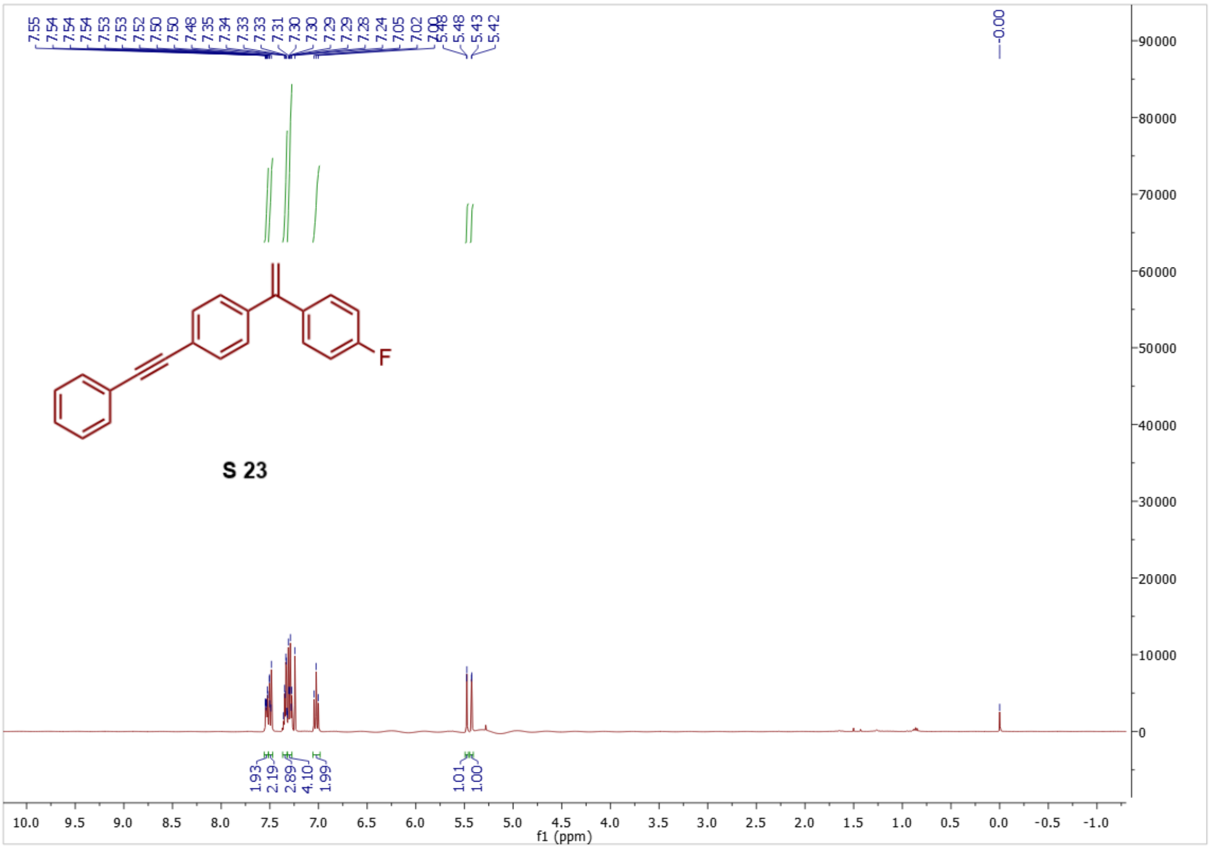
7.55
7.54
7.54
7.54
7.53
7.53
7.52
7.50
7.50
7.48
7.35
7.34
7.33
7.33
7.31
7.30
7.30
7.29
7.29
7.28
7.24
7.05
7.02
7.00
5.48
5.43
5.42
0.00
1.93
2.19
2.89
4.10
1.99
1.01
1.00
F
S 23
f1 (ppm)

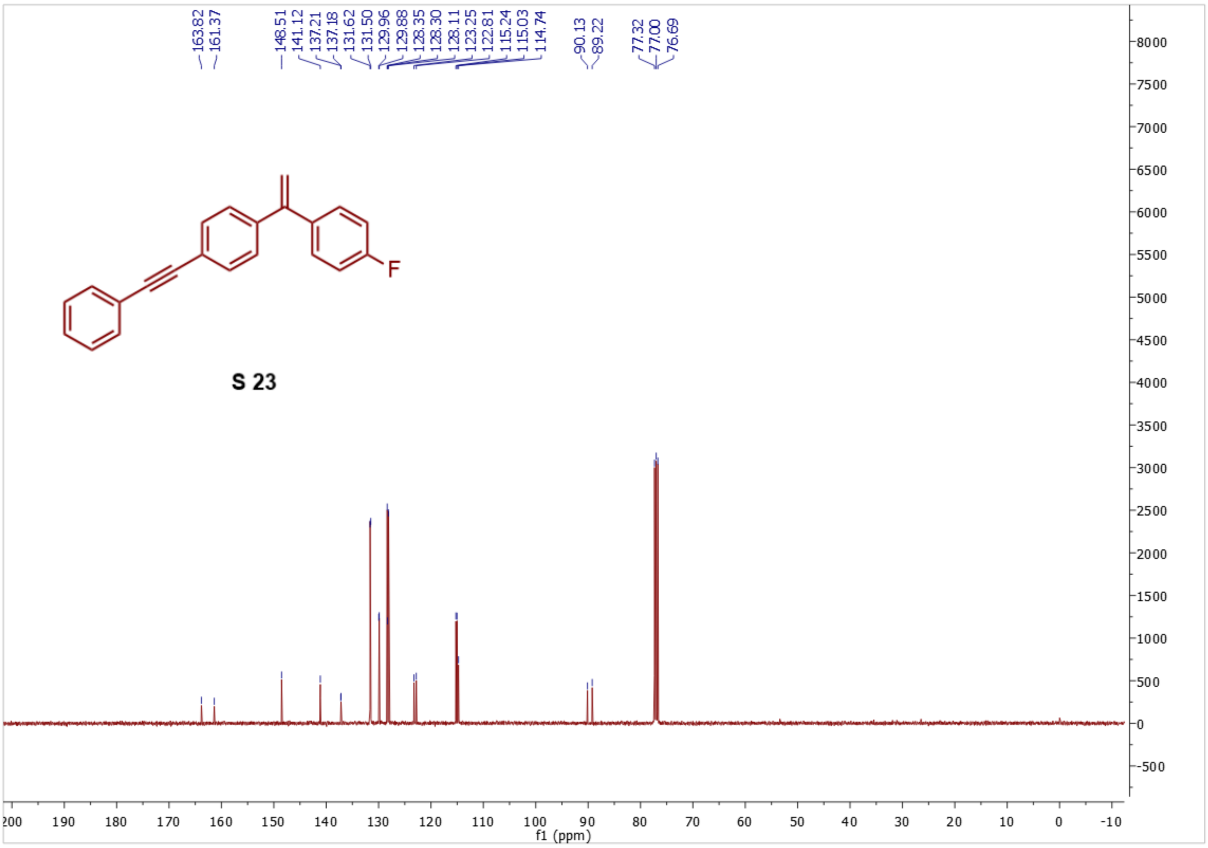
163.82
161.37
148.51
141.12
137.21
137.18
131.62
131.50
129.96
129.88
128.35
128.30
128.11
123.25
122.81
115.24
115.03
114.74
90.13
89.22
77.32
77.00
76.69
F
S 23
f1 (ppm)

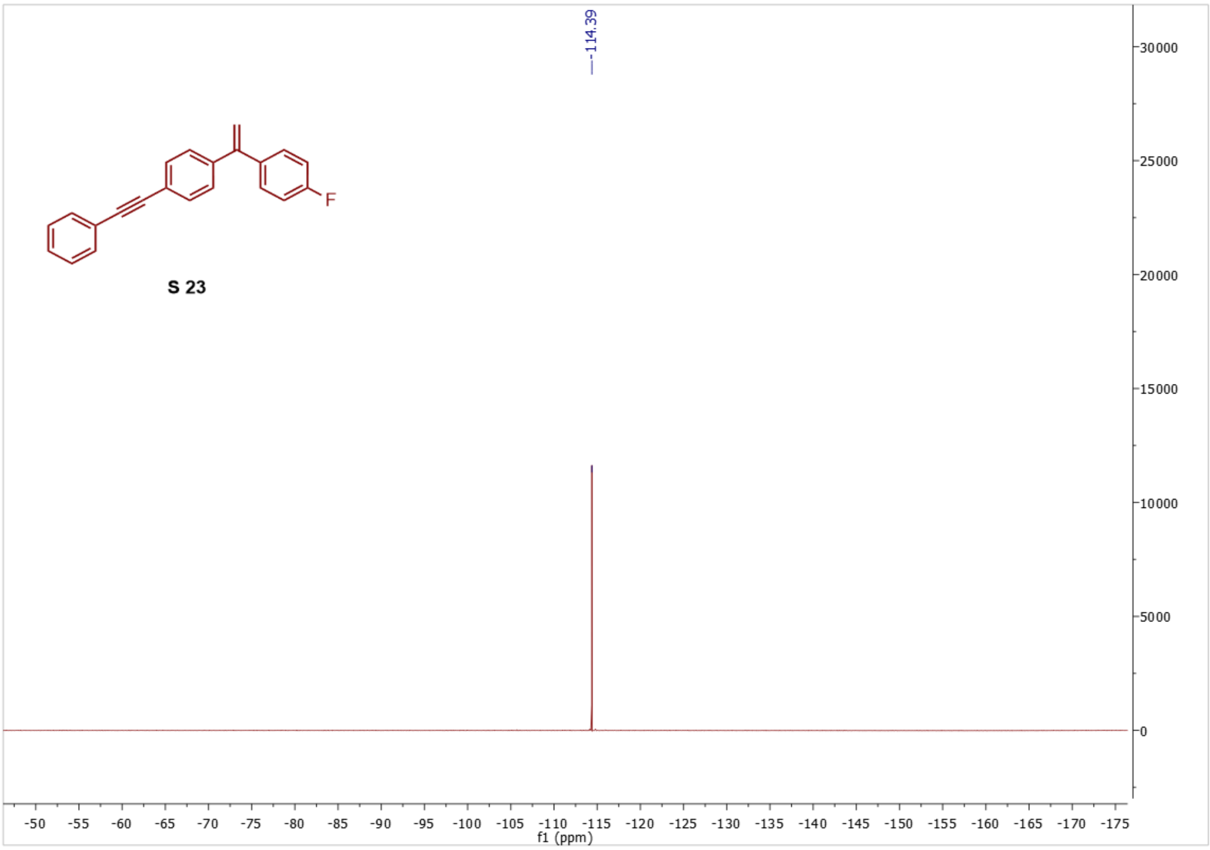

S 23

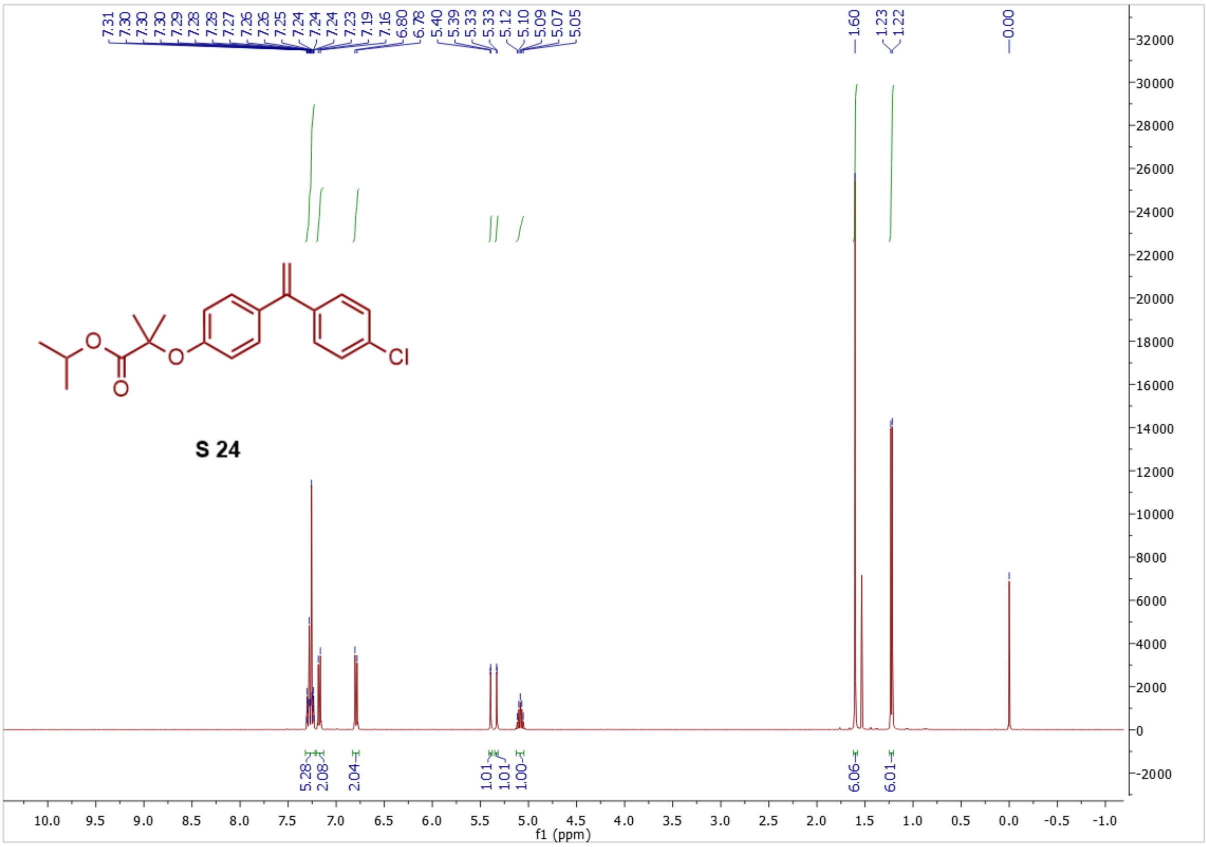

S 24

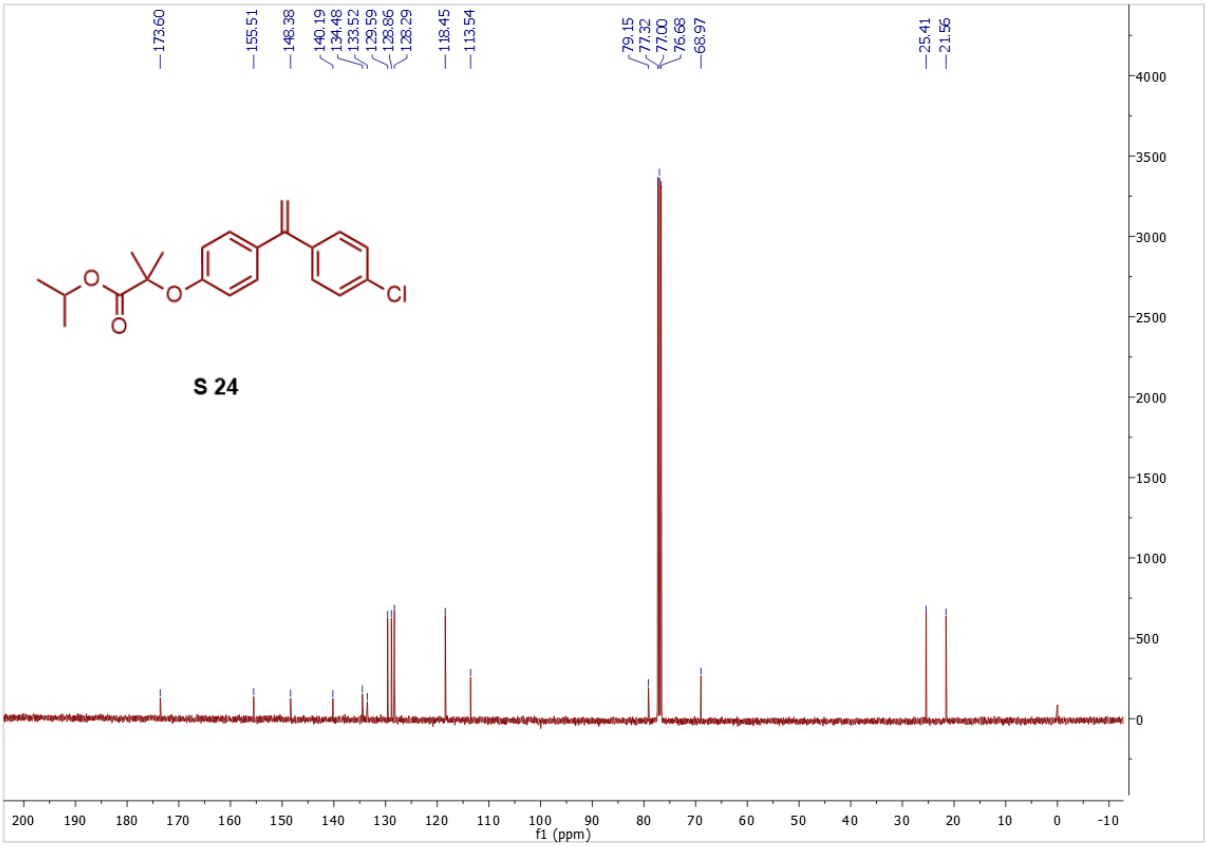
173.60
155.51
148.38
140.19
134.48
133.52
129.59
128.86
128.29
118.45
113.54
79.15
77.32
77.00
76.68
68.97
25.41
21.56
Cl
O
f1 (ppm)

S 24

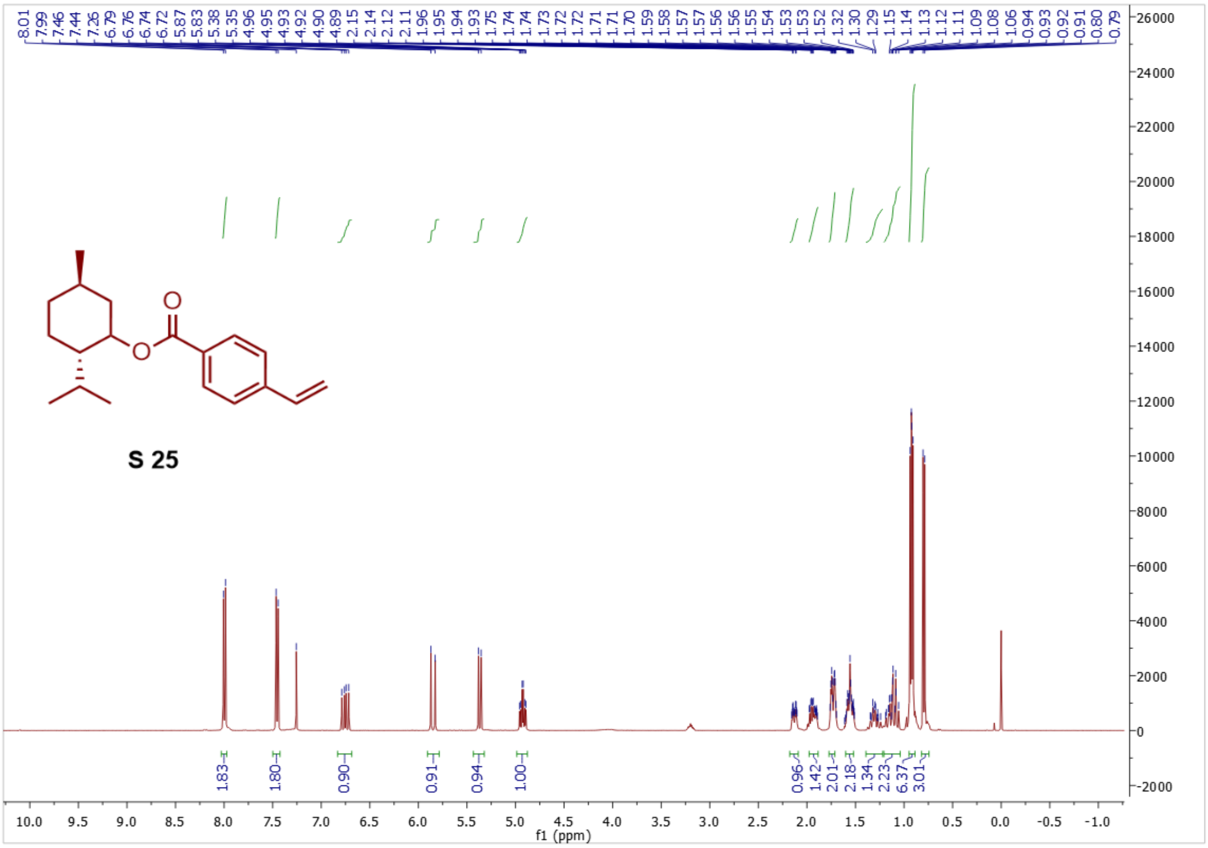
8.01
7.99
7.46
7.44
7.26
6.79
6.76
6.74
6.72
5.87
5.83
5.38
5.35
4.96
4.95
4.93
4.92
4.90
4.89
2.15
2.14
2.12
2.11
1.96
1.95
1.94
1.93
1.75
1.74
1.74
1.73
1.72
1.72
1.71
1.71
1.70
1.59
1.58
1.57
1.57
1.56
1.56
1.55
1.54
1.53
1.53
1.52
1.32
1.30
1.29
1.15
1.14
1.13
1.12
1.11
1.09
1.08
1.06
0.94
0.93
0.92
0.91
0.80
0.79
1.83
1.80
0.90
0.91
0.94
1.00
0.96
1.42
2.01
2.18
1.34
2.23
6.37
3.01
O
f1 (ppm)

S 25

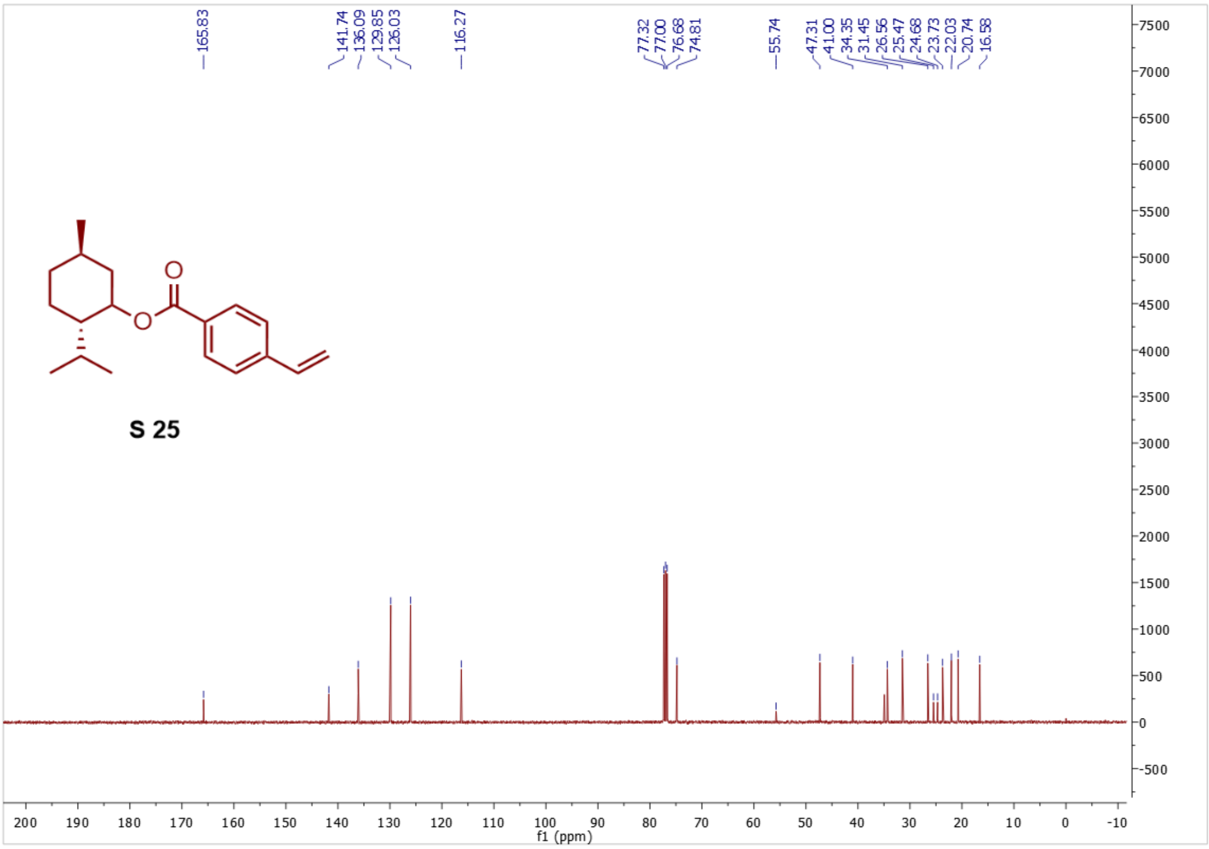

S 25

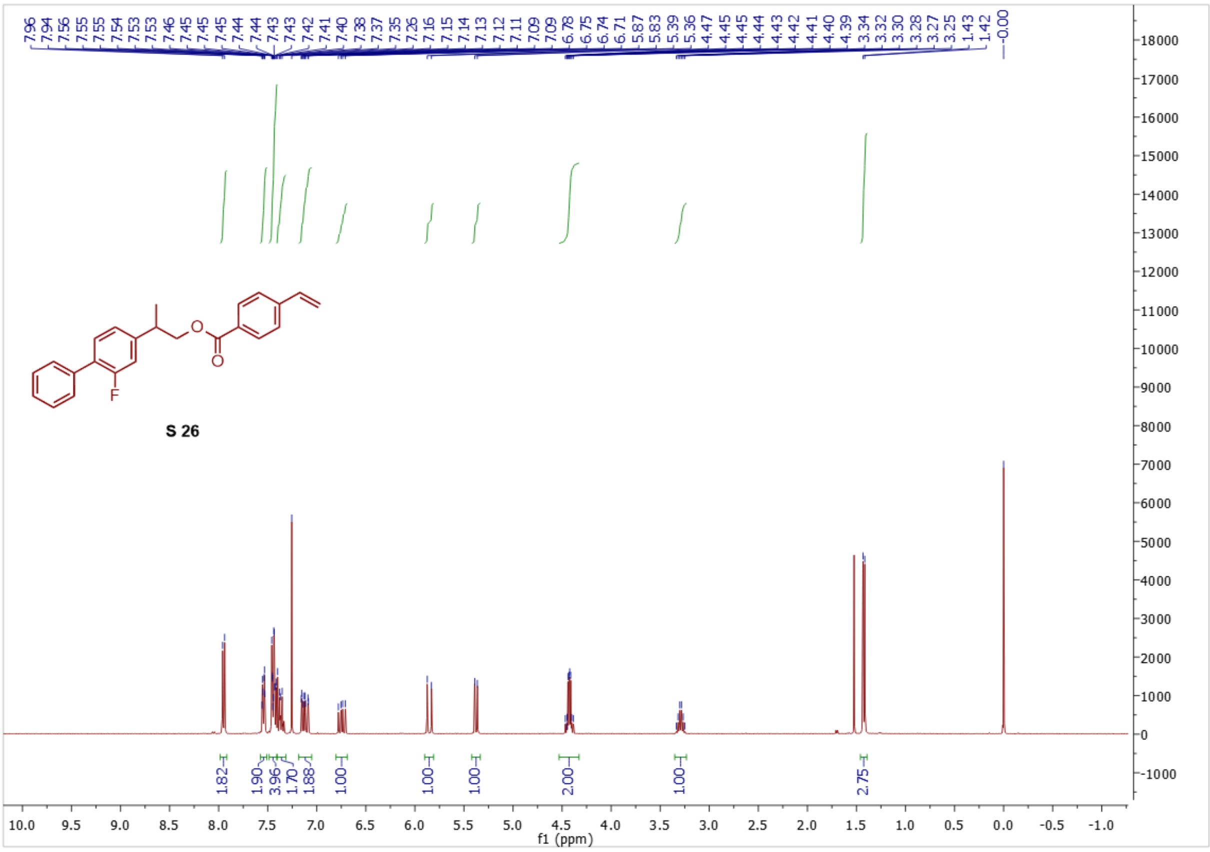

S 26

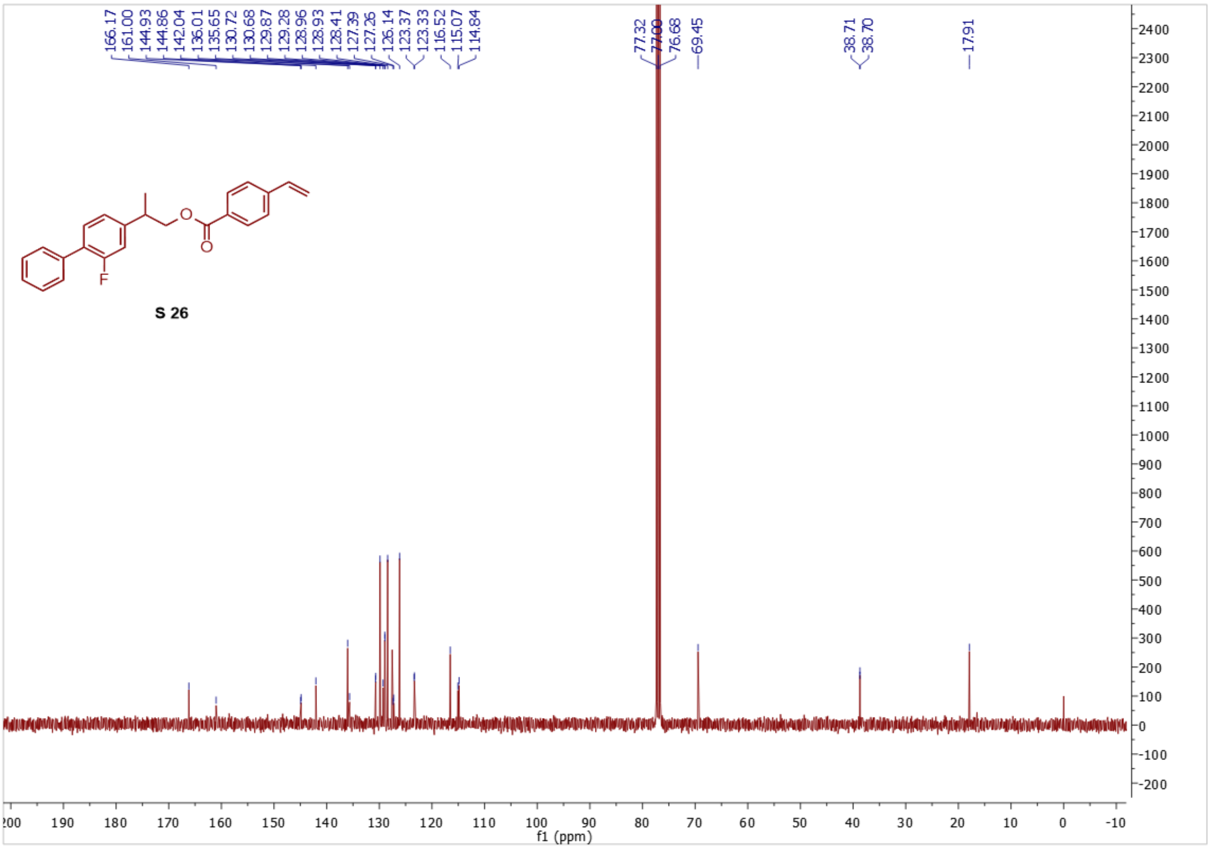
166.17
161.00
144.93
144.86
142.04
136.01
135.65
130.72
130.68
129.87
129.28
128.96
128.93
128.41
127.39
127.26
126.14
123.37
123.33
116.52
115.07
114.84
77.32
77.00
76.68
69.45
38.71
38.70
17.91
F
S 26
f1 (ppm)

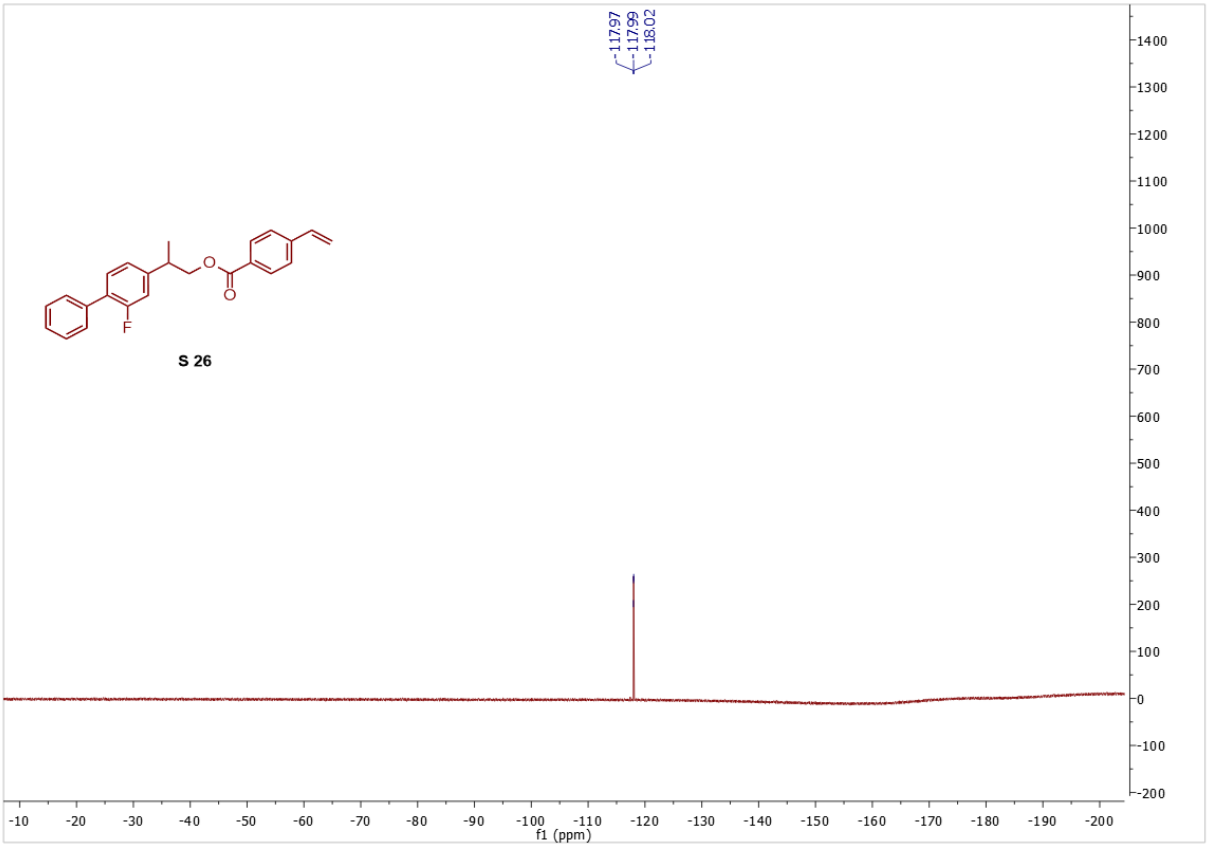
-117.97
-117.99
-118.02
F
S 26
f1 (ppm)

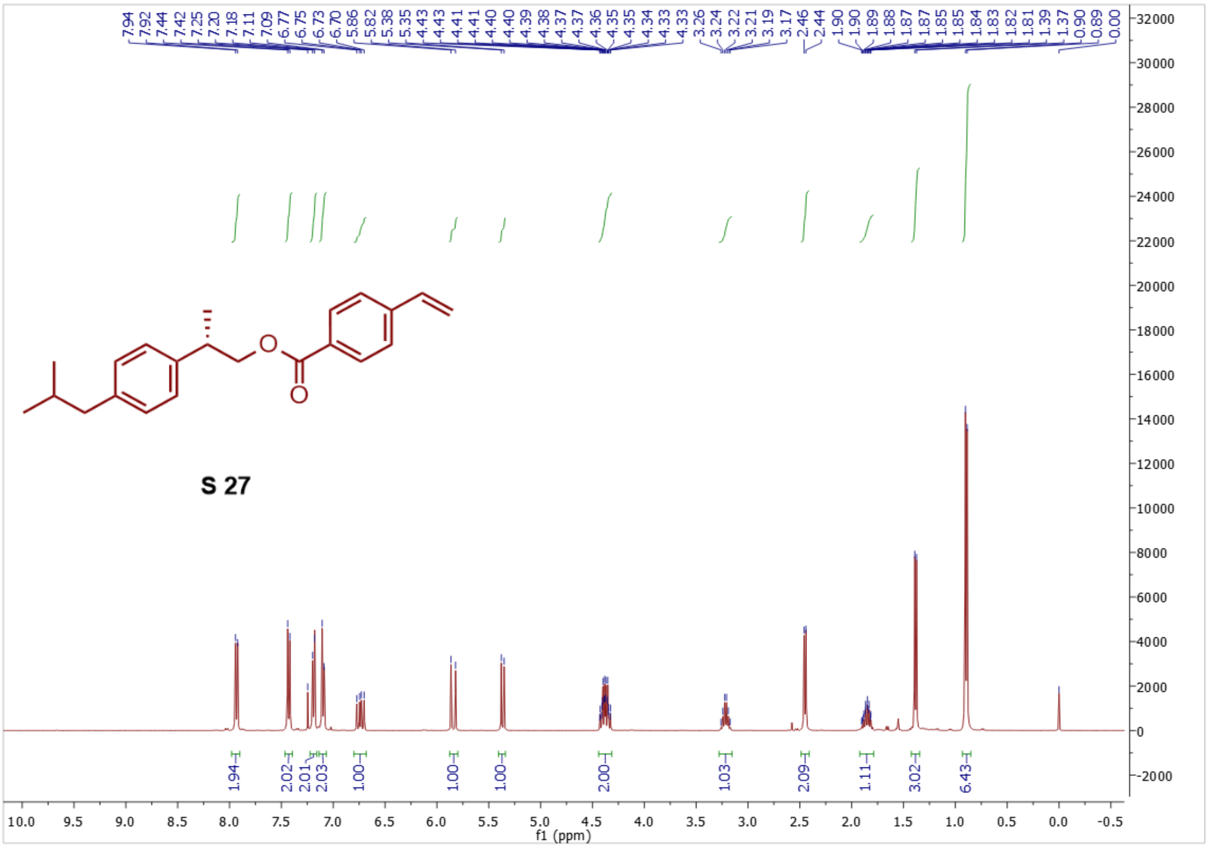

S 27

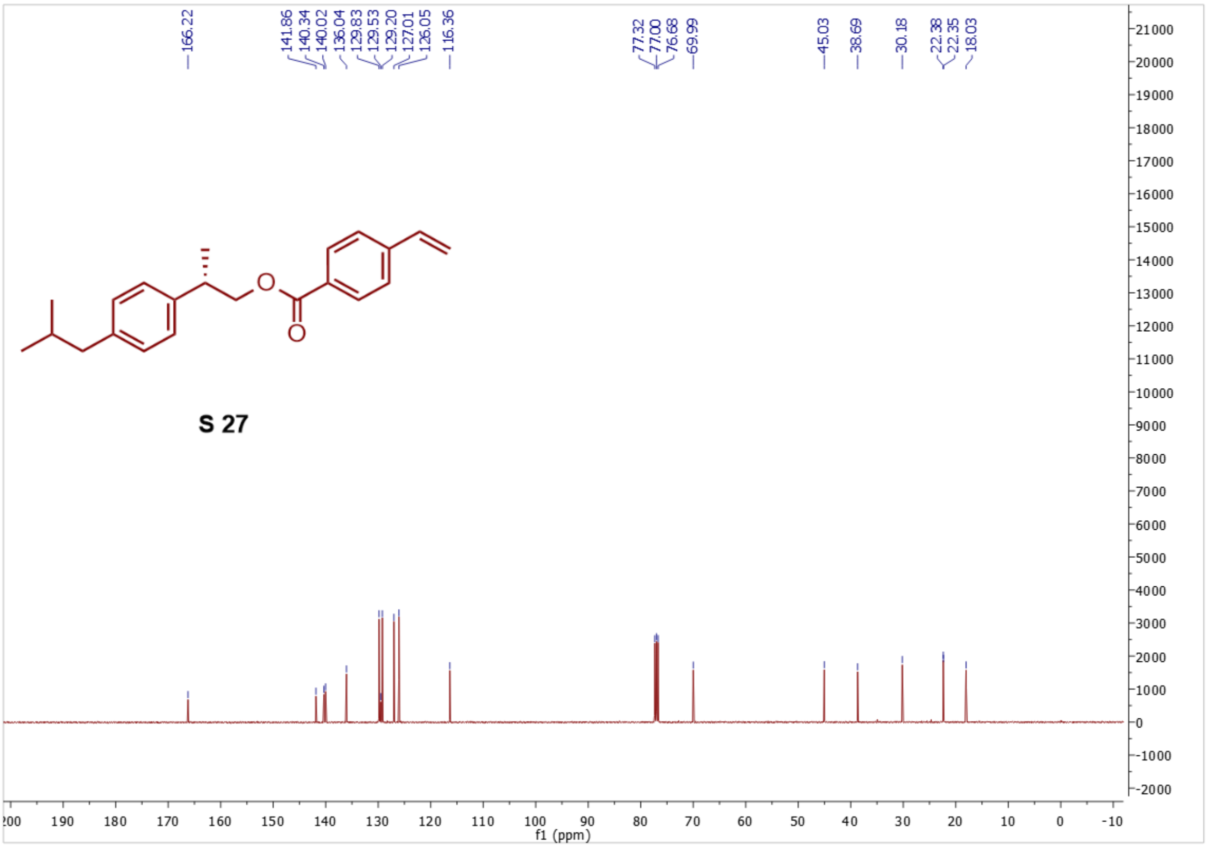

S 27

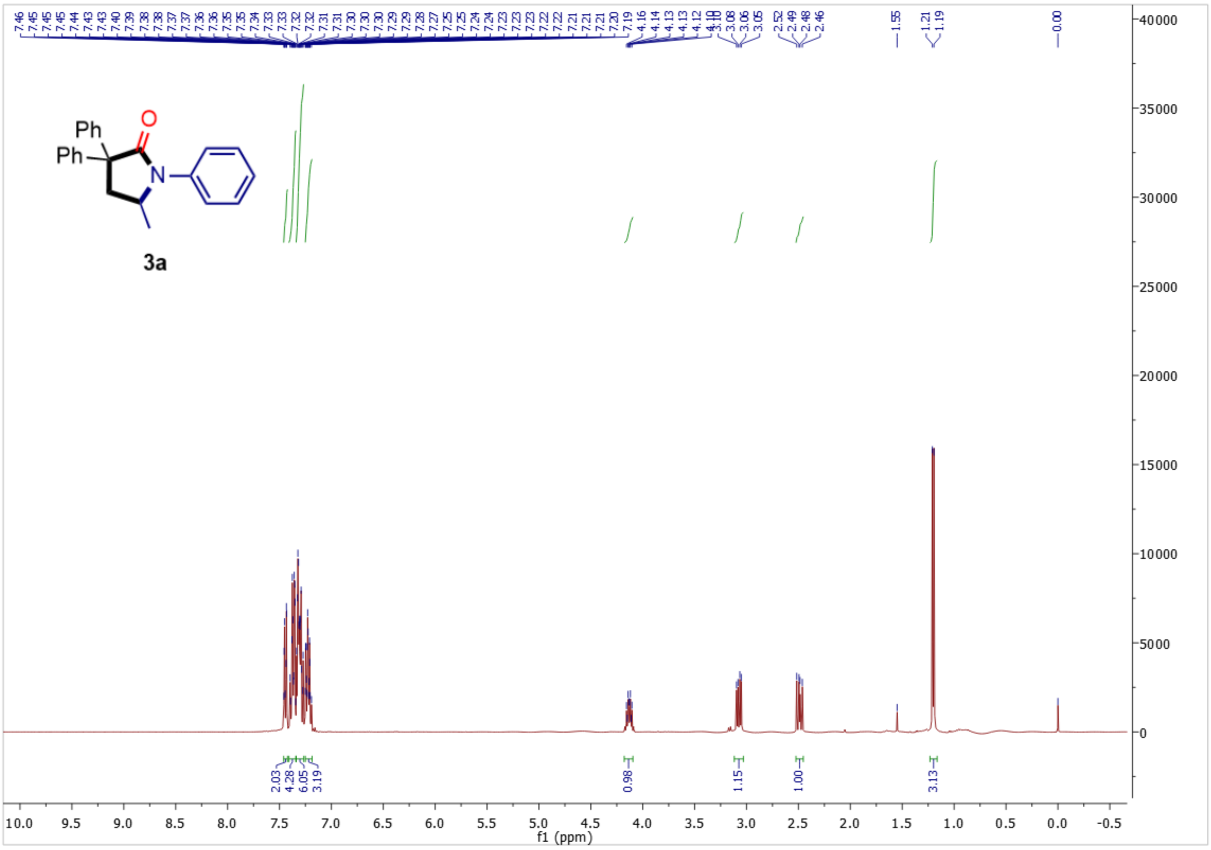

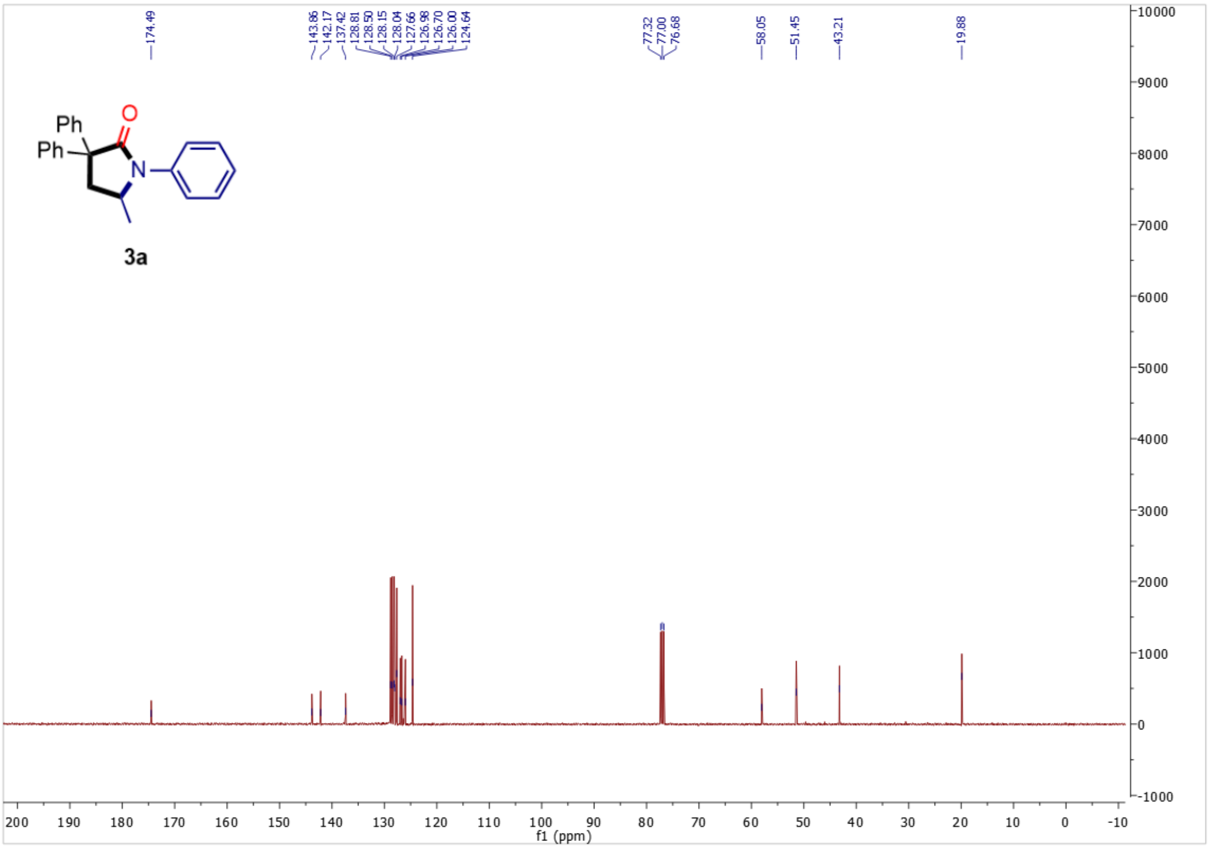

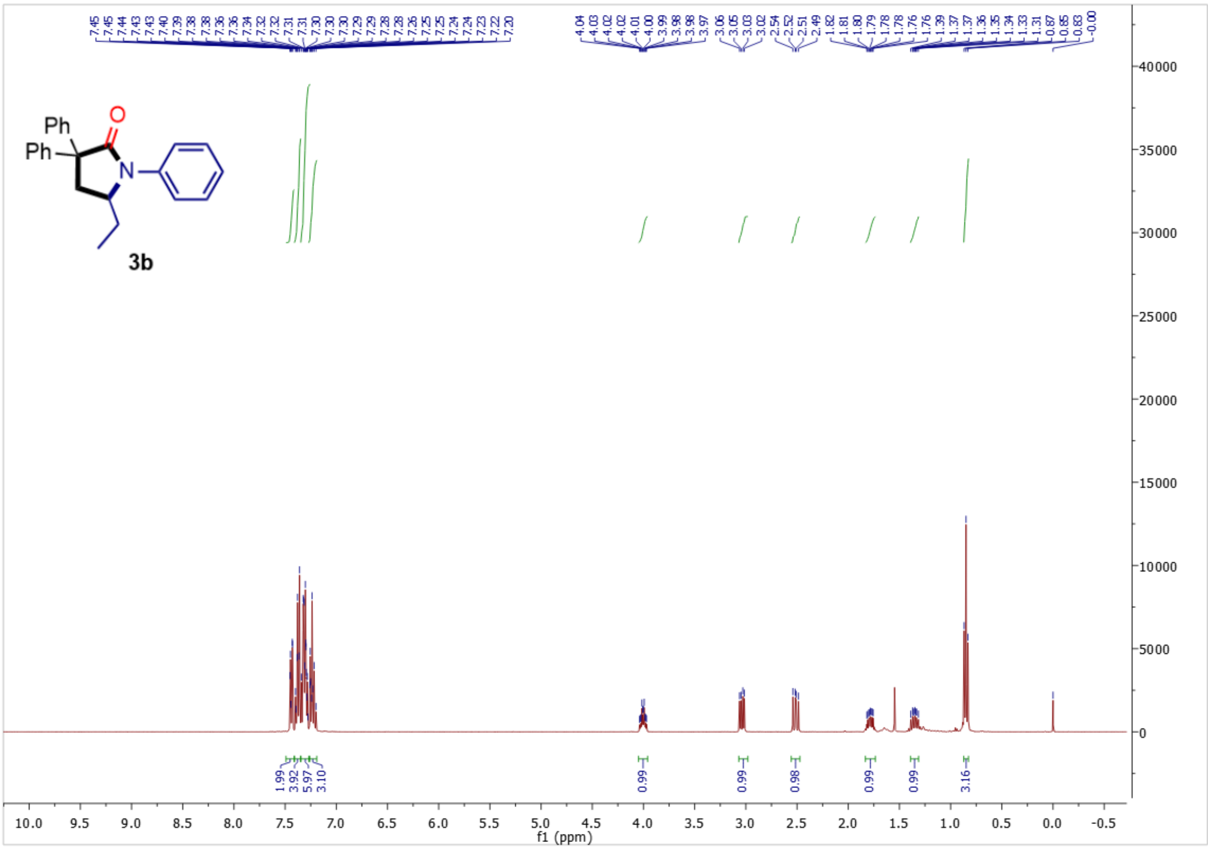

Ph
Ph
O
N
3b
1.99
3.92
5.97
3.10
0.99
0.99
0.98
0.99
0.99
3.16
f1 (ppm)

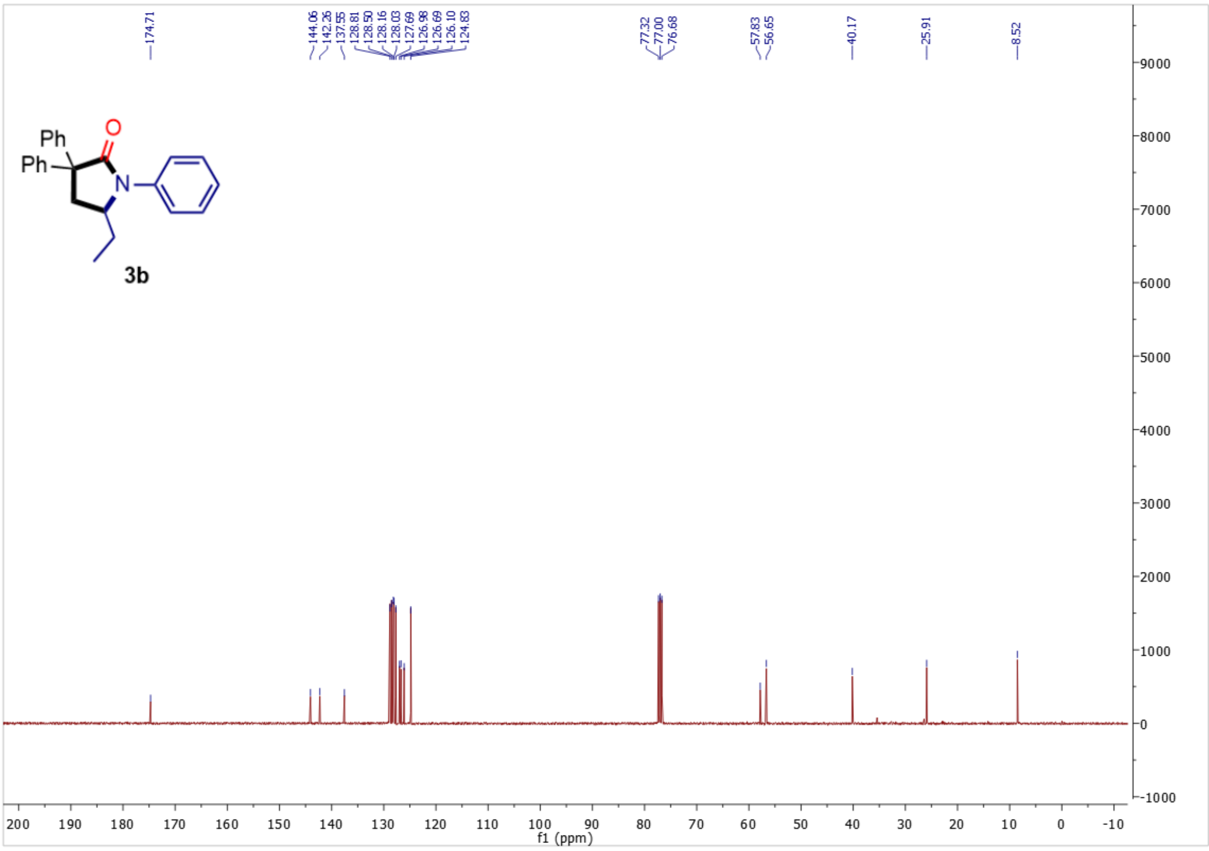

174.71
144.06
142.26
137.55
128.81
128.50
128.16
128.03
127.69
126.98
126.69
126.10
124.83
77.32
77.00
76.68
57.83
56.65
40.17
25.91
8.52
Ph
Ph
O
N
3b
f1 (ppm)

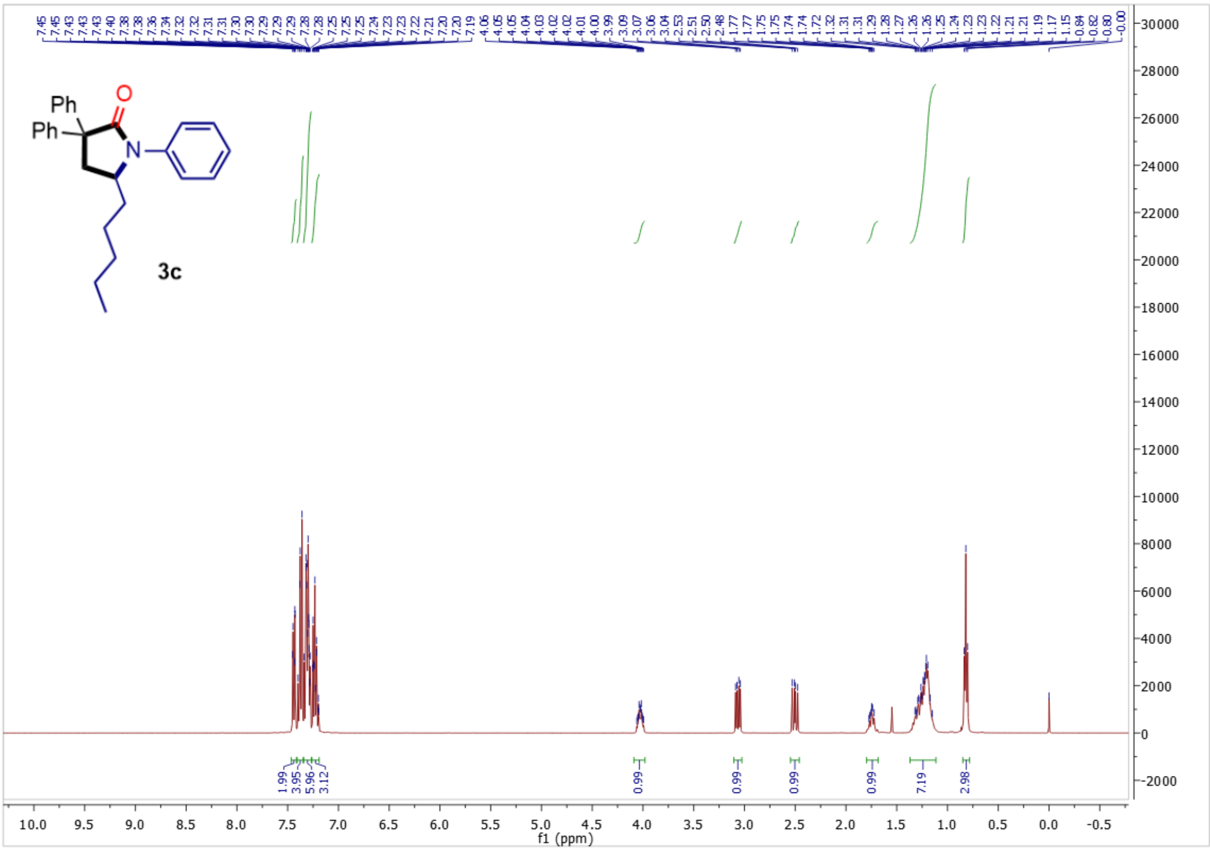

Ph
Ph
O
N
3c
f1 (ppm)

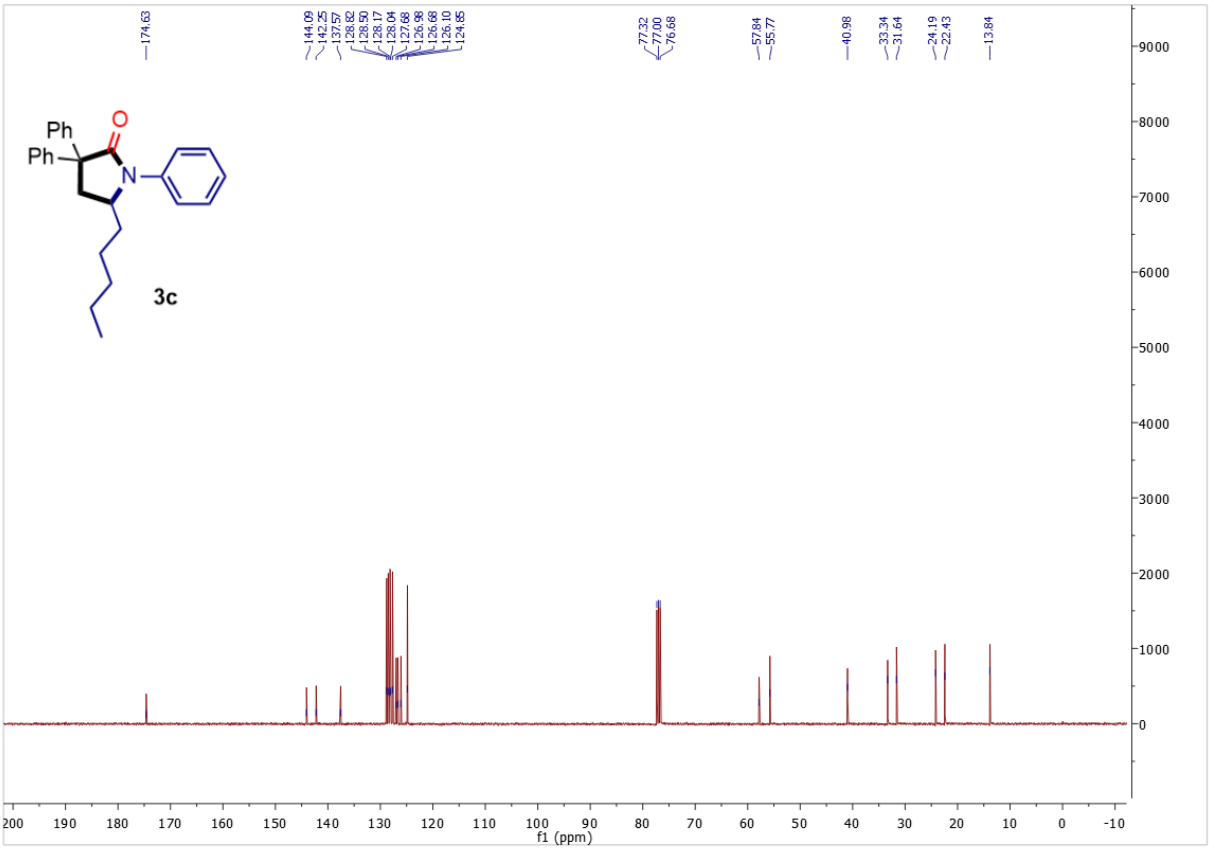

Ph
Ph
O
N
3c
174.63
144.09
142.25
137.57
128.82
128.50
128.17
128.04
127.68
126.98
126.68
126.10
124.85
77.32
77.00
76.68
57.84
55.77
40.98
33.34
31.64
24.19
22.43
13.84
f1 (ppm)

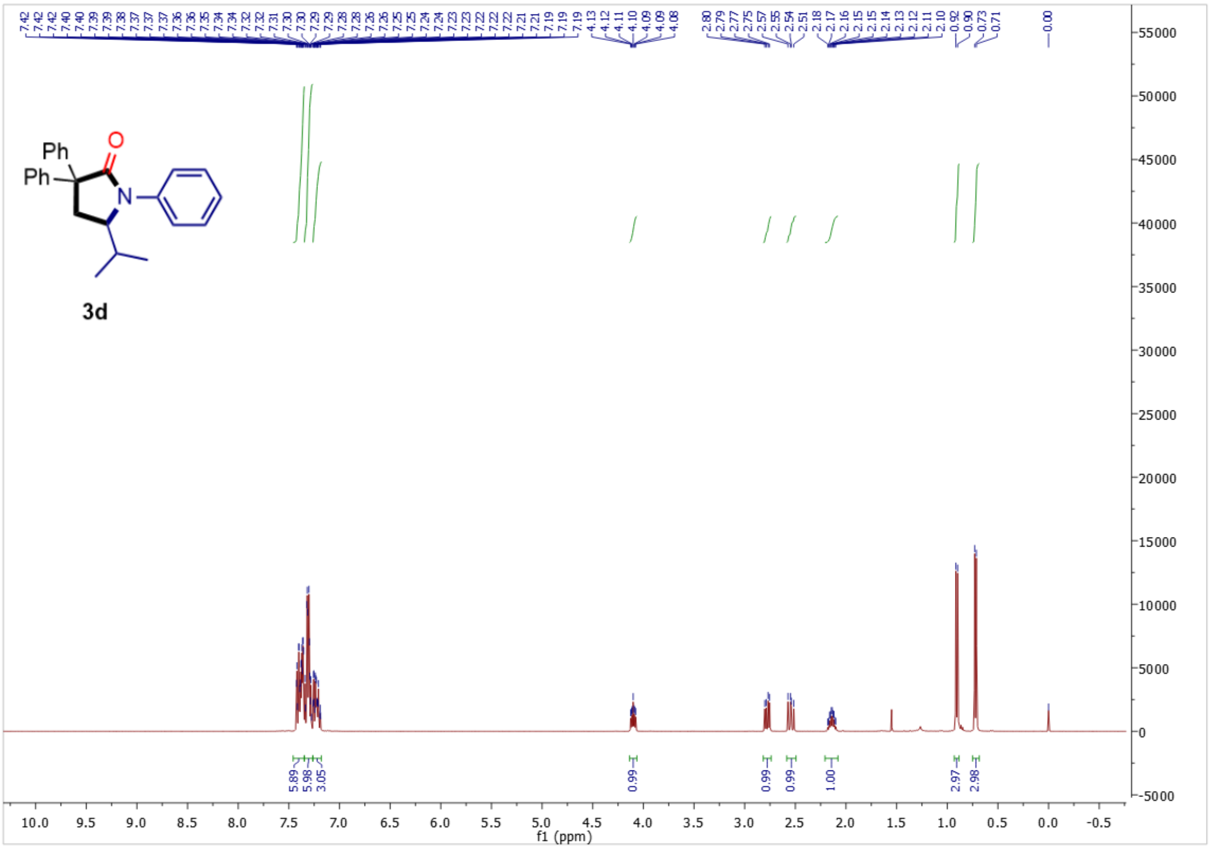
3d
f1 (ppm)

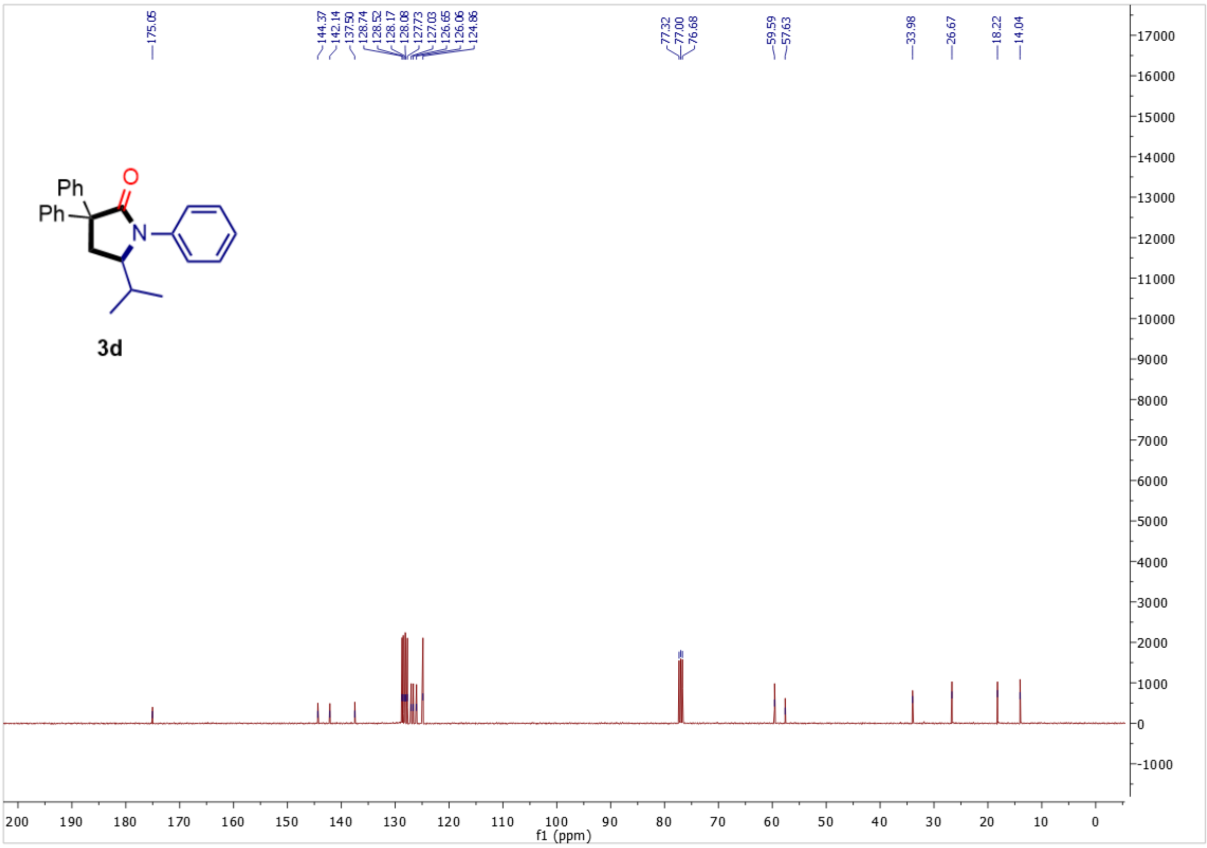
3d
f1 (ppm)

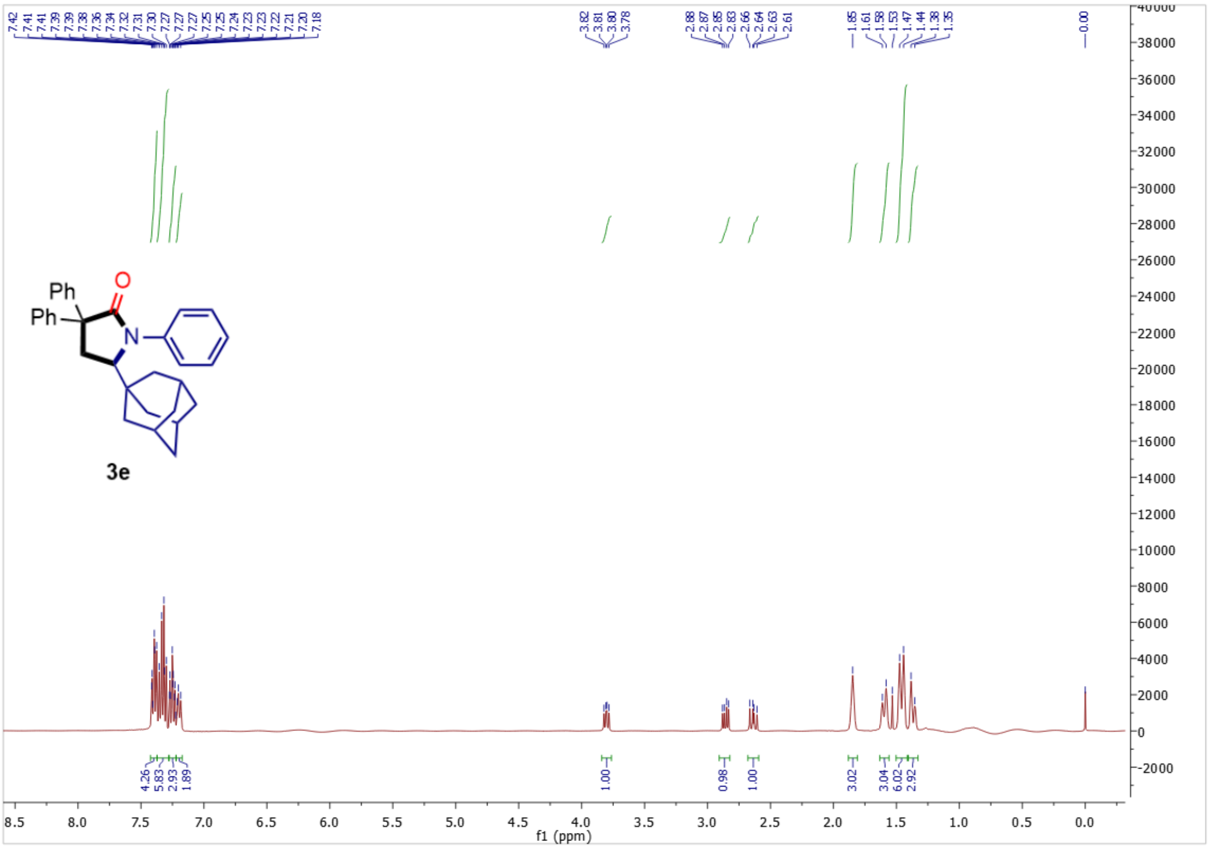

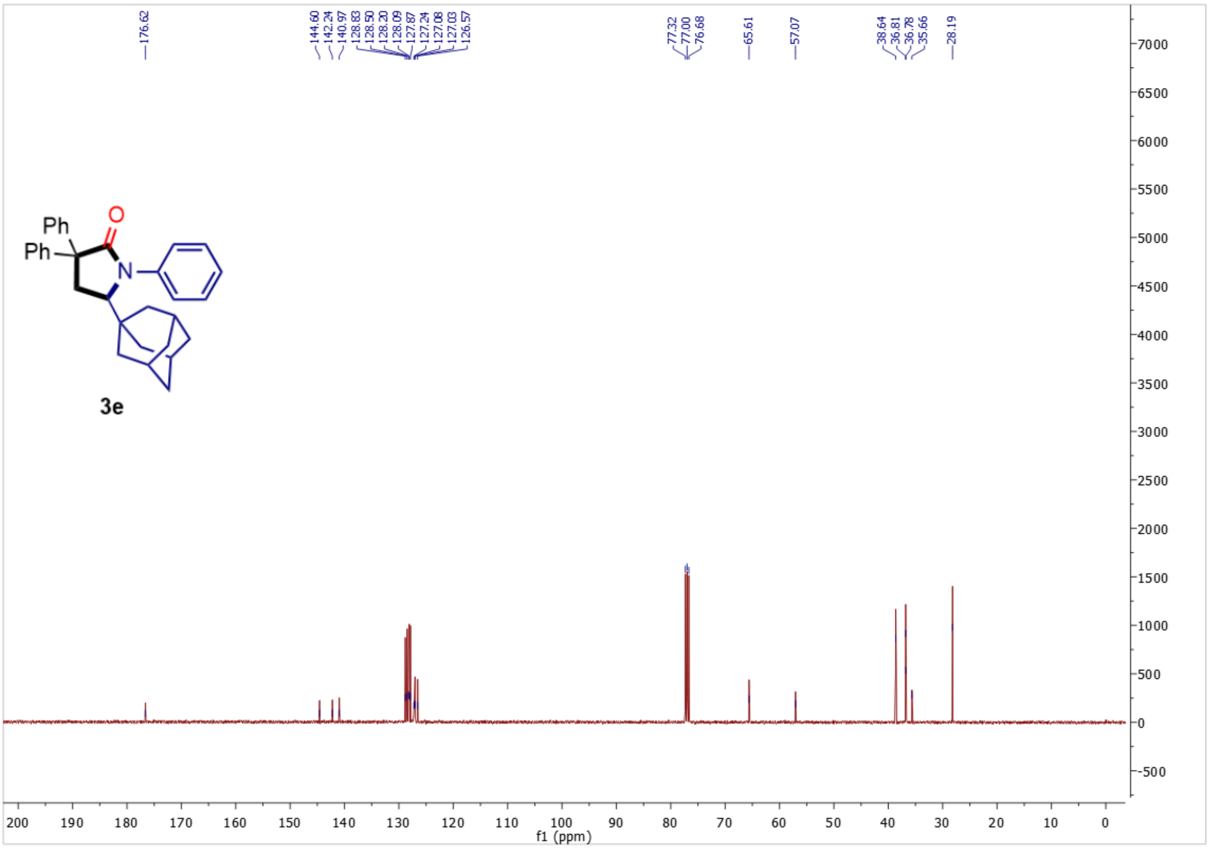

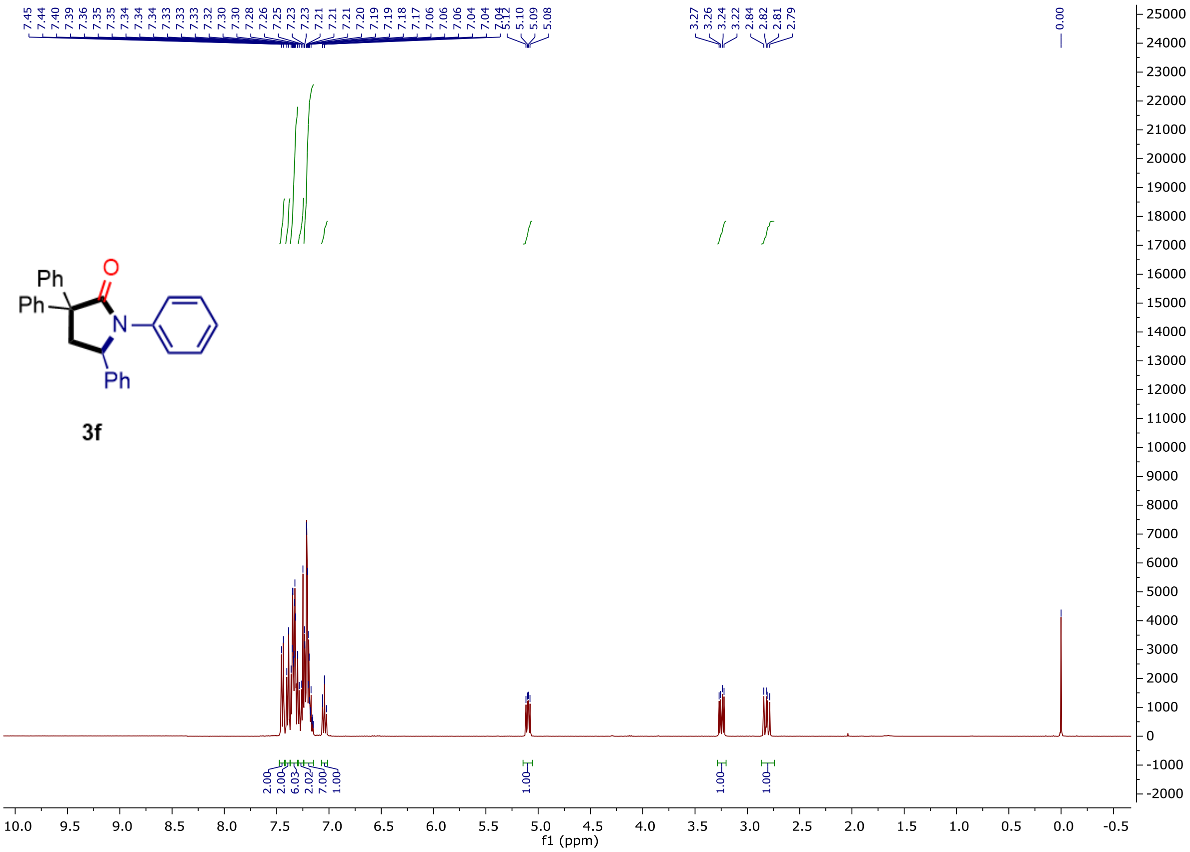

3f

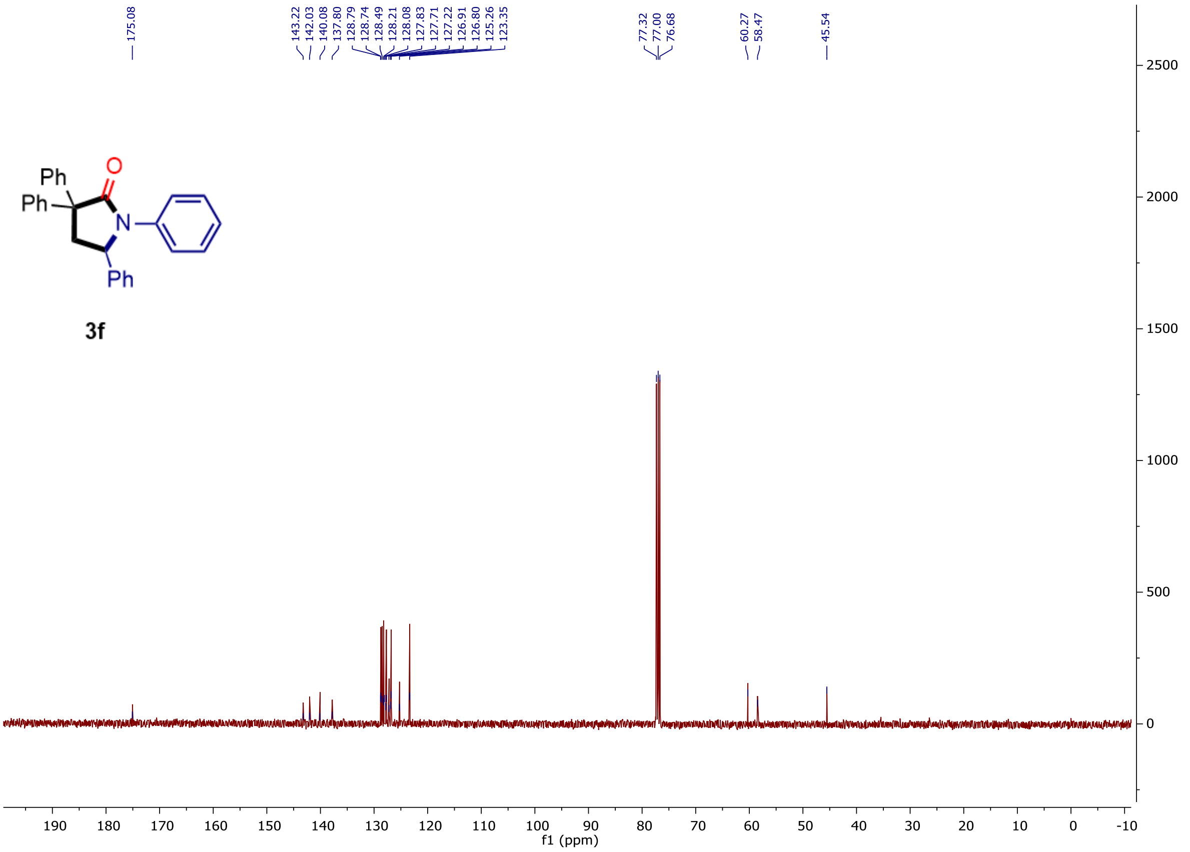

3f

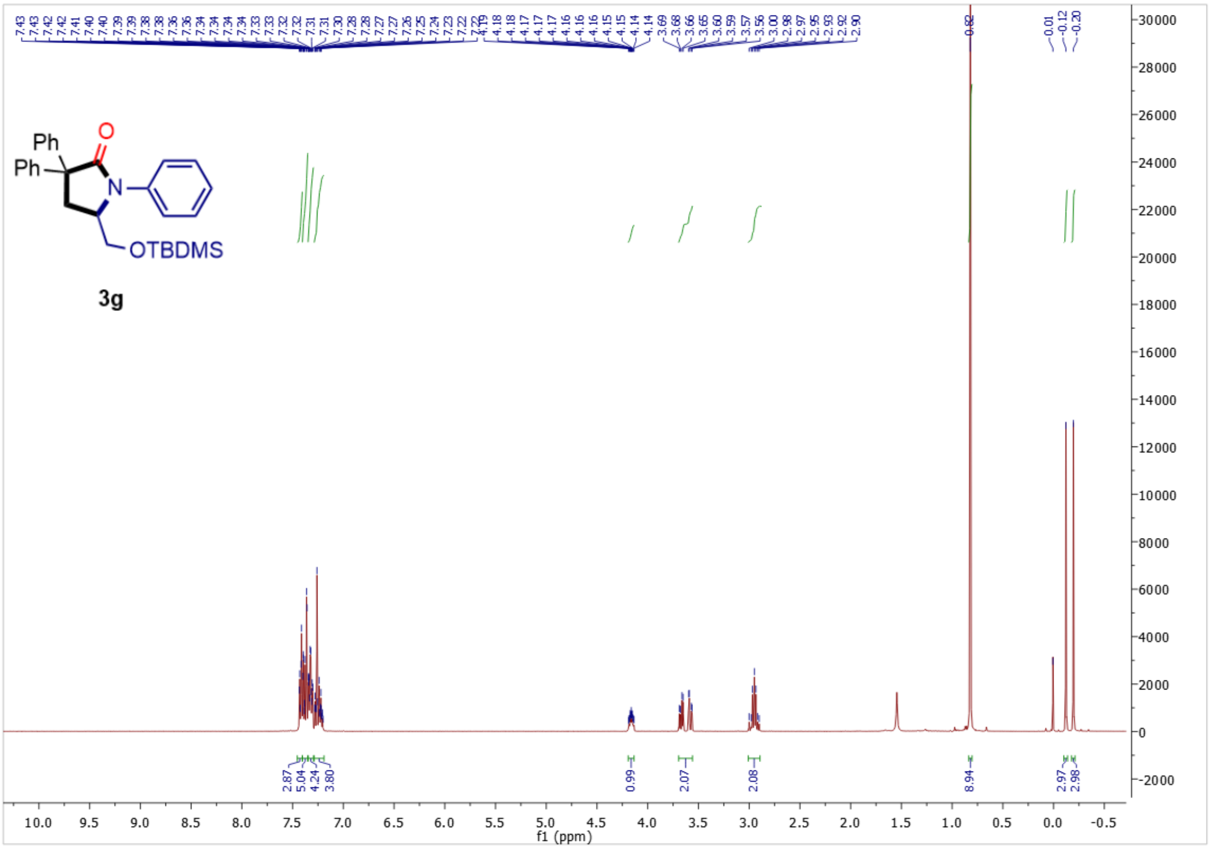

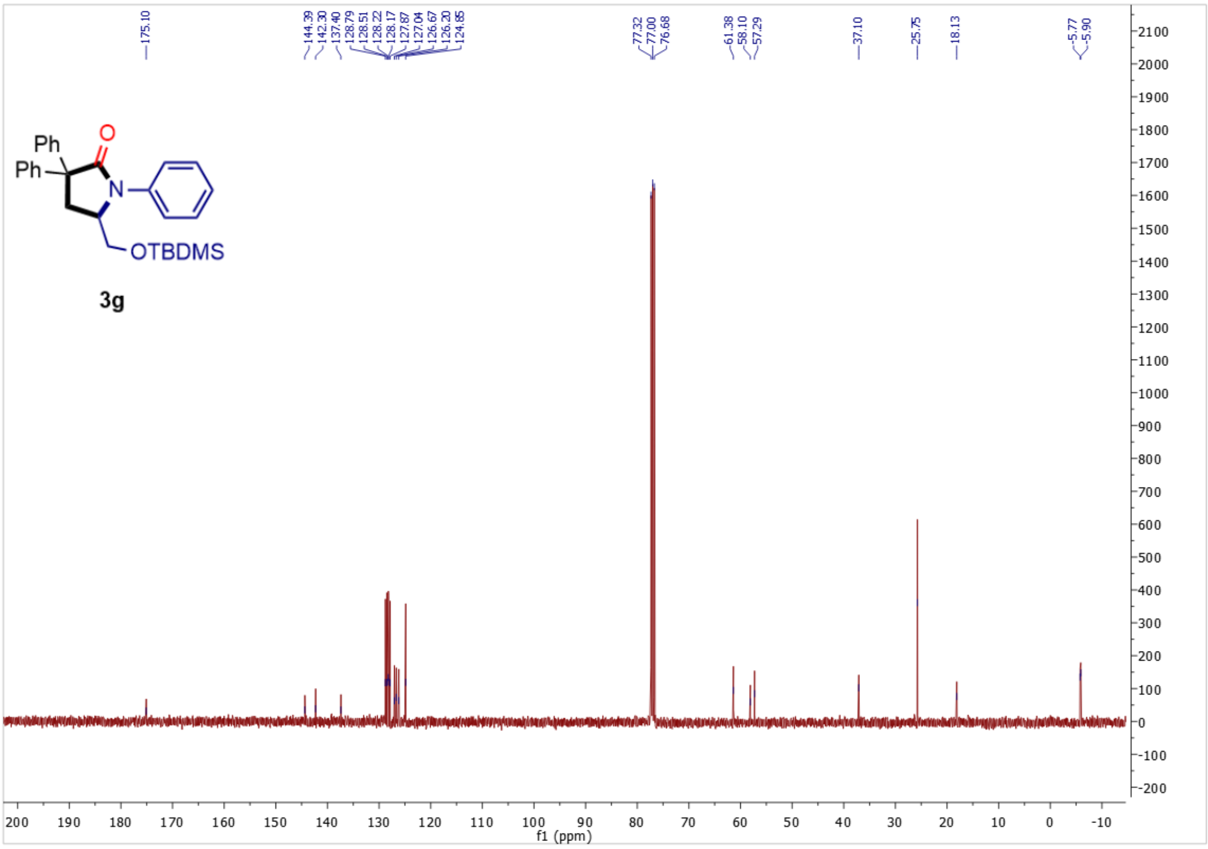

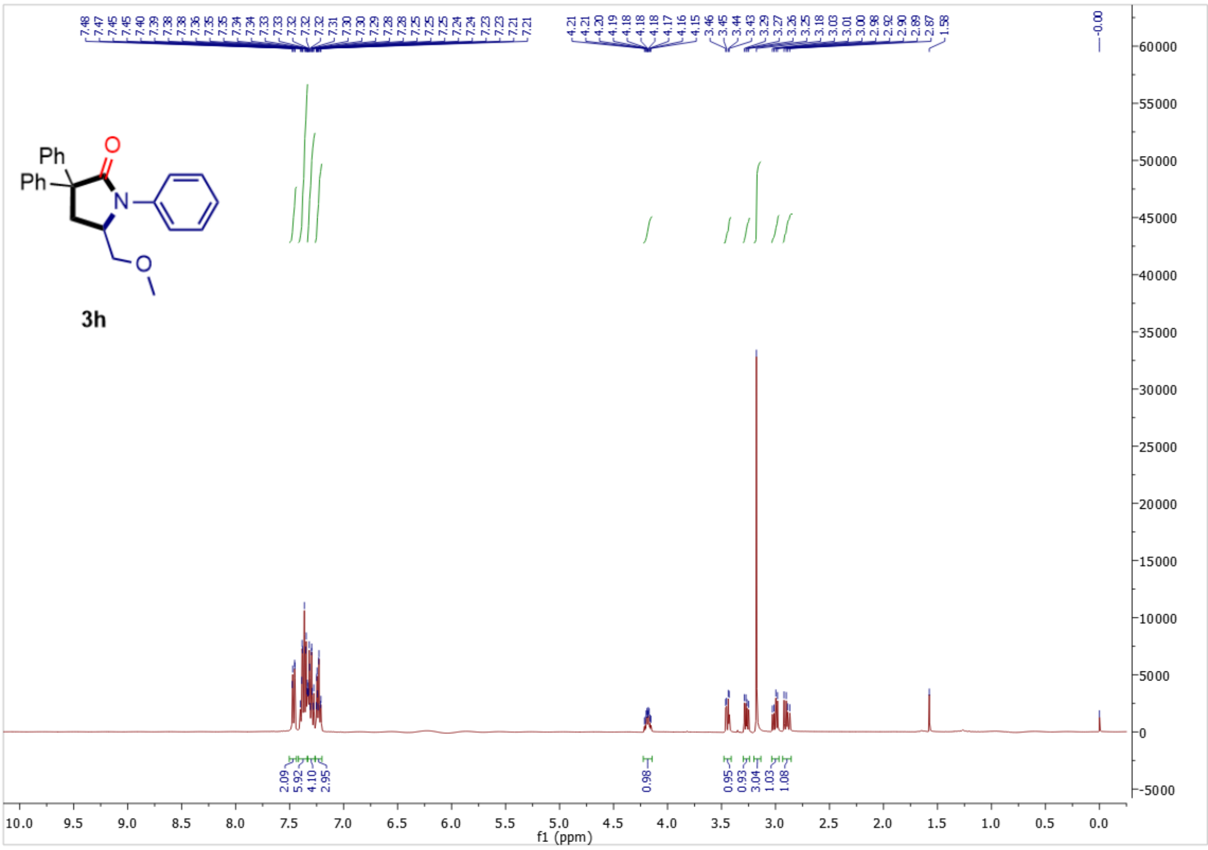

Ph
Ph
O
N
O
3h
f1 (ppm)

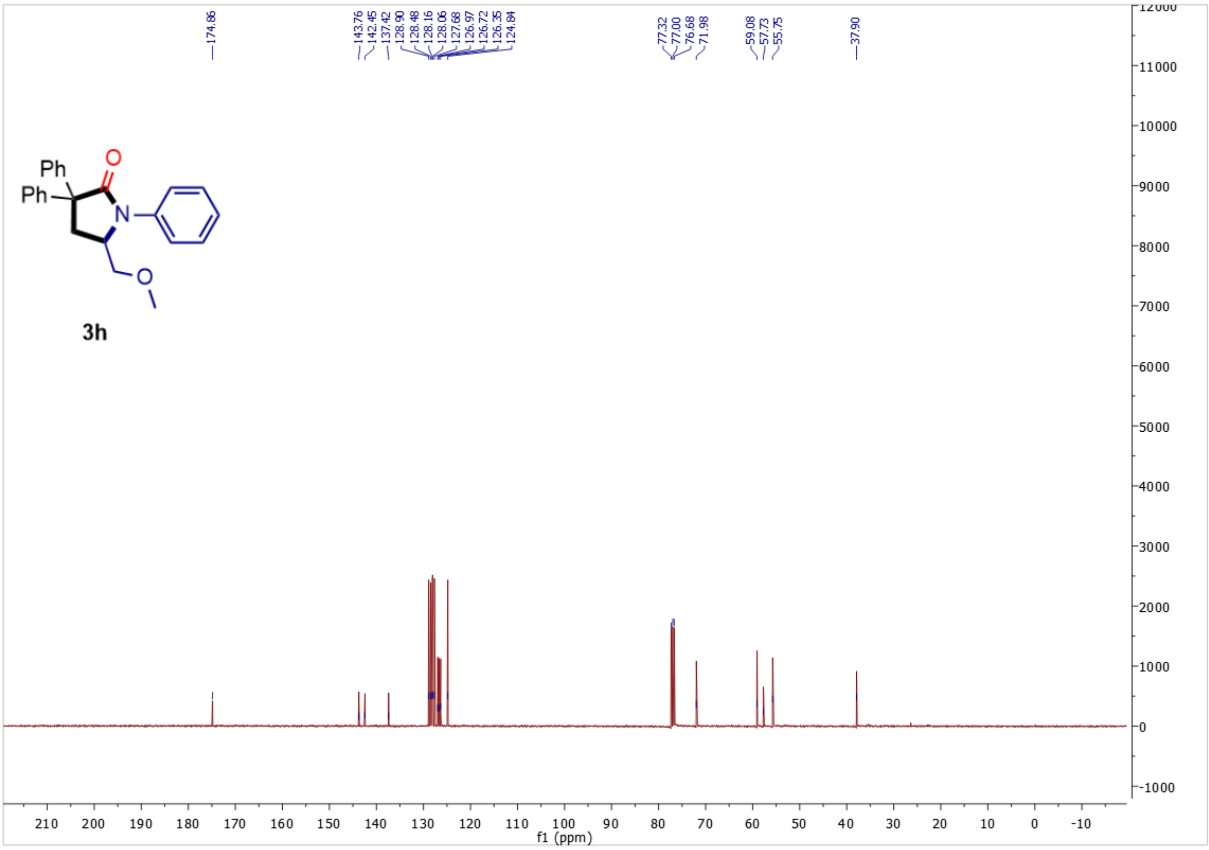

Ph
Ph
O
N
O
3h
f1 (ppm)

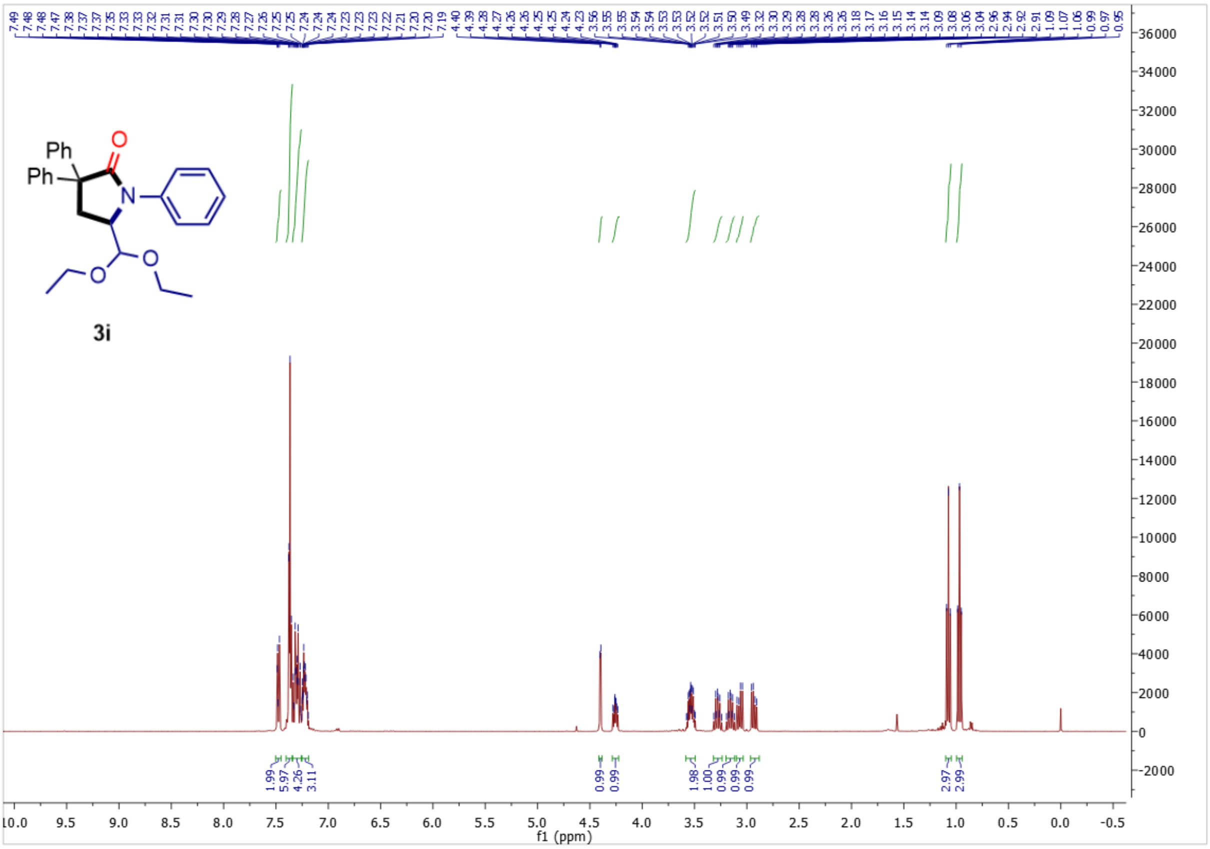

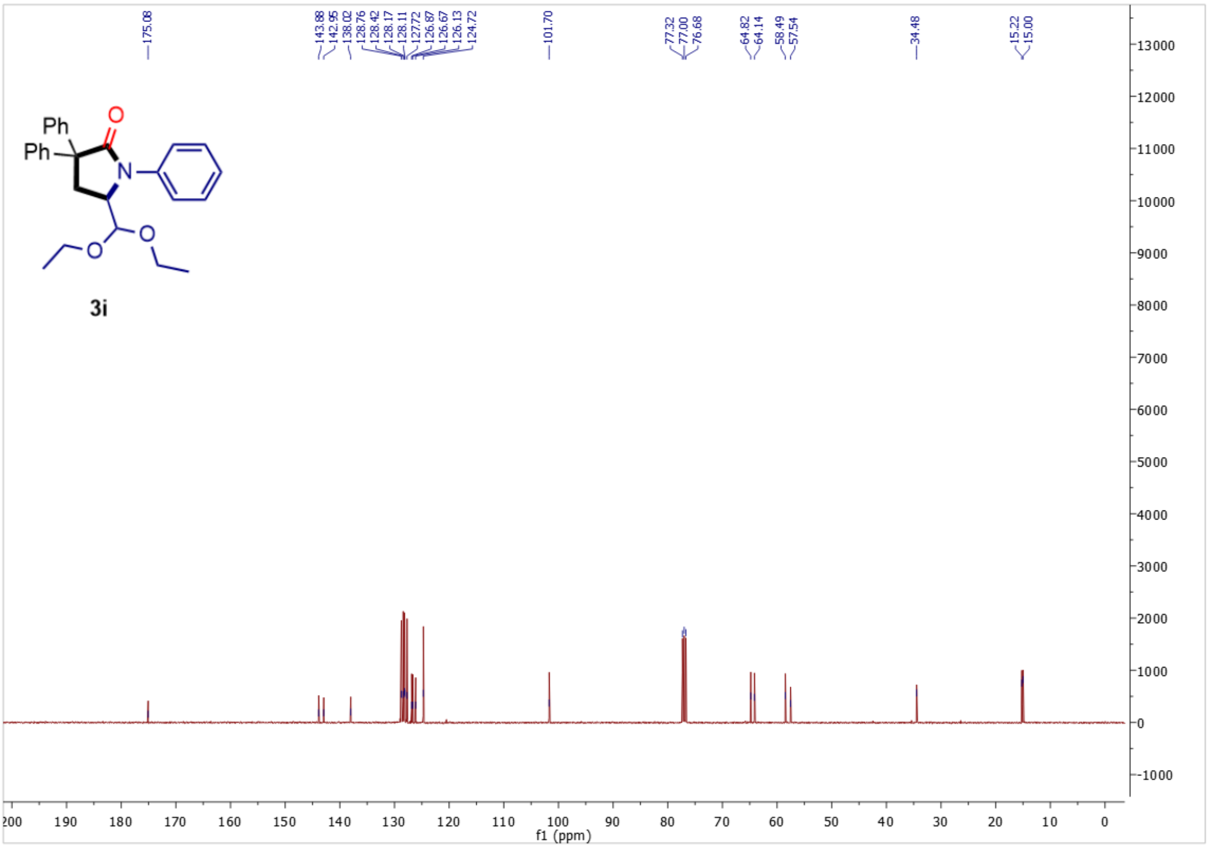

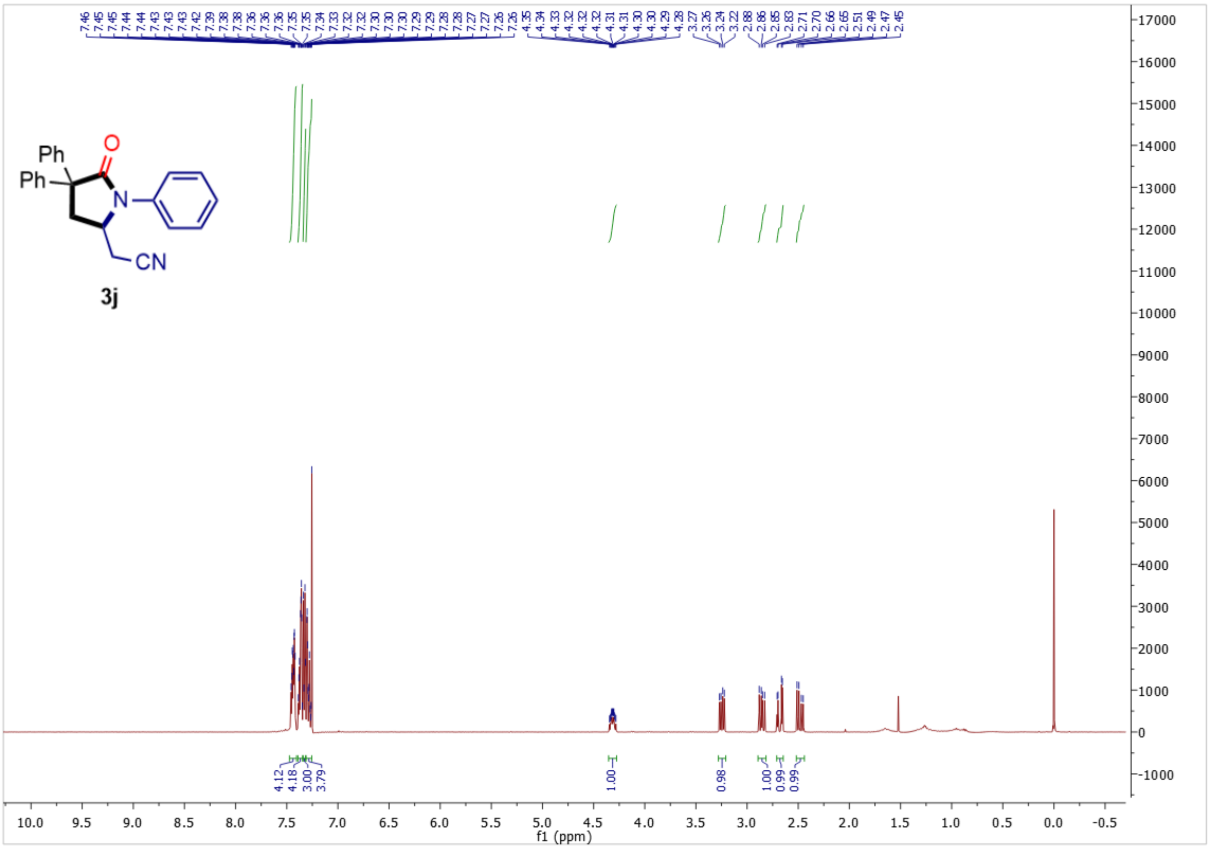

Ph
Ph
O
N
CN
3j
4.12
4.18
3.00
3.79
1.00
0.98
1.00
0.99
0.99
f1 (ppm)

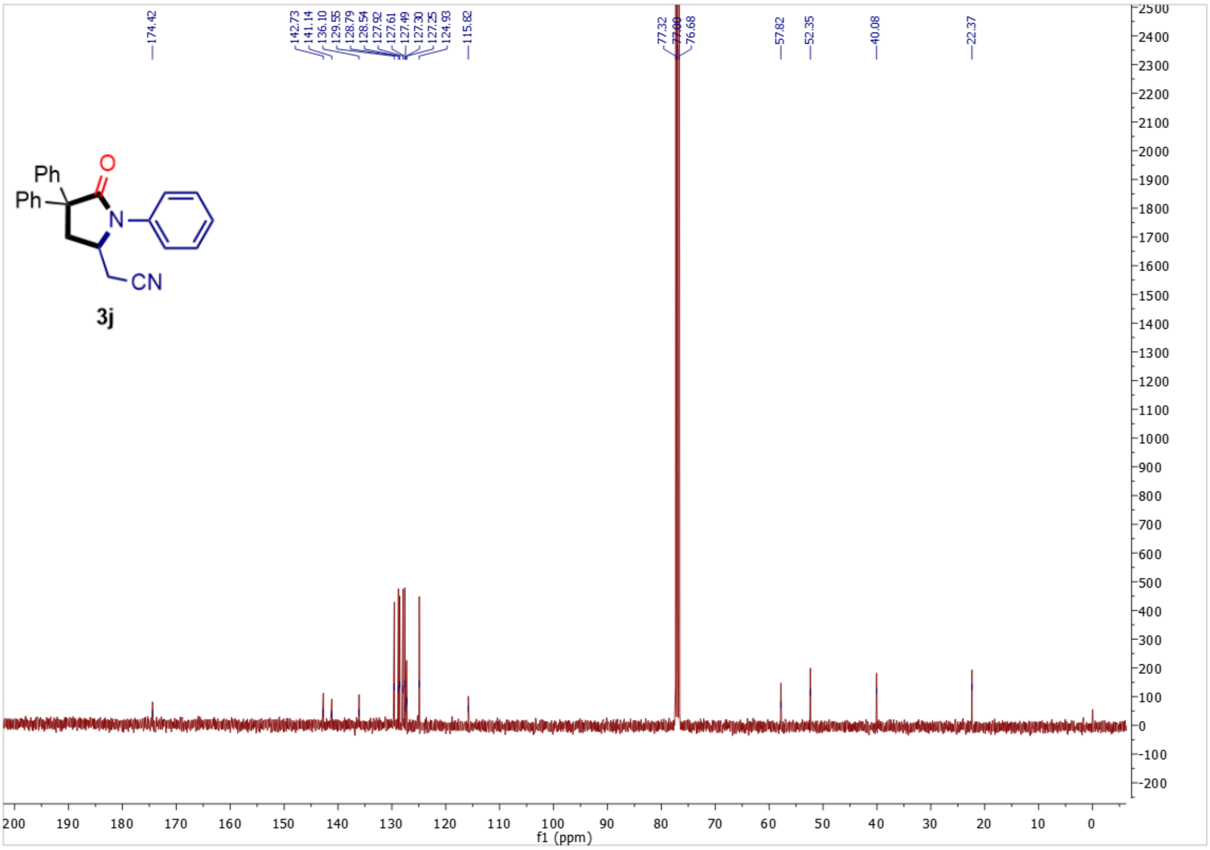

174.42
142.73
141.14
136.10
129.55
128.79
128.54
127.92
127.61
127.49
127.30
127.25
124.93
115.82
77.32
77.00
76.68
57.82
52.35
40.08
22.37
Ph
Ph
O
N
CN
3j
f1 (ppm)

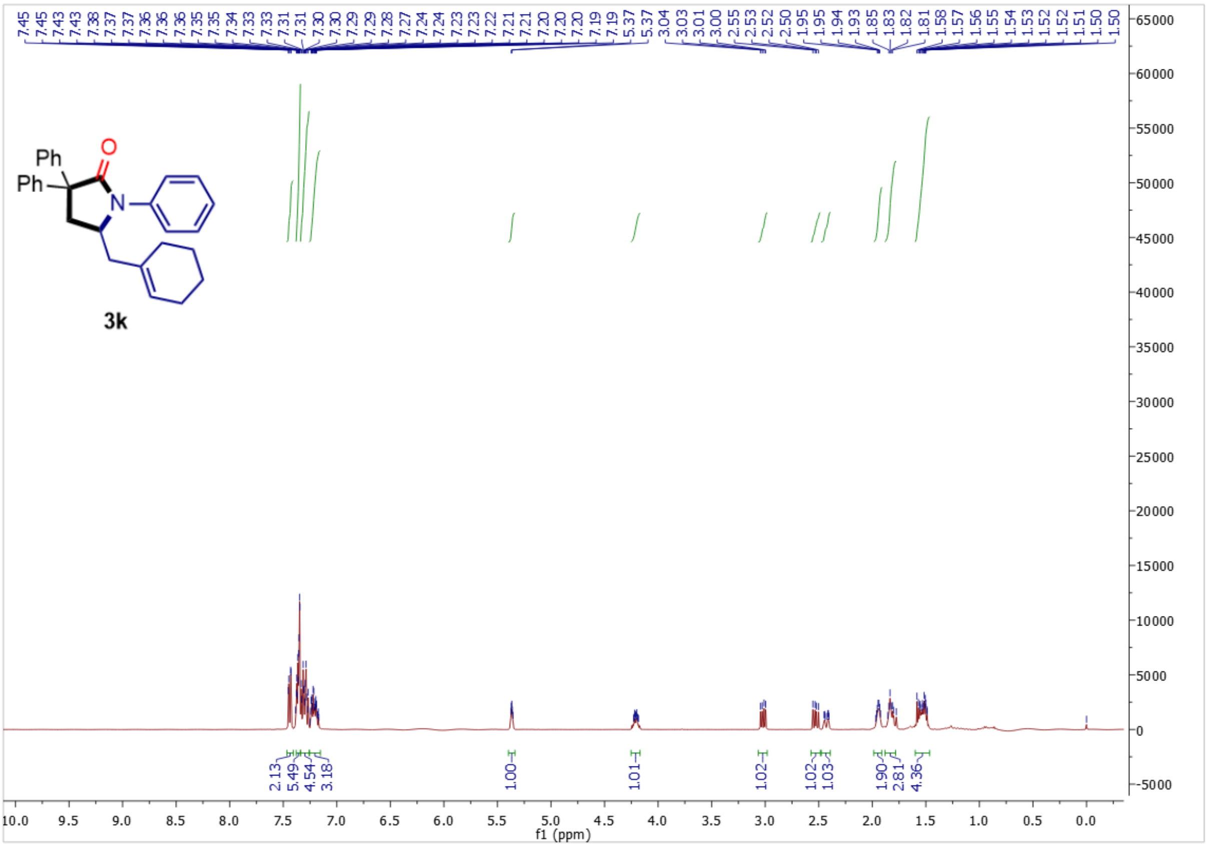

3k

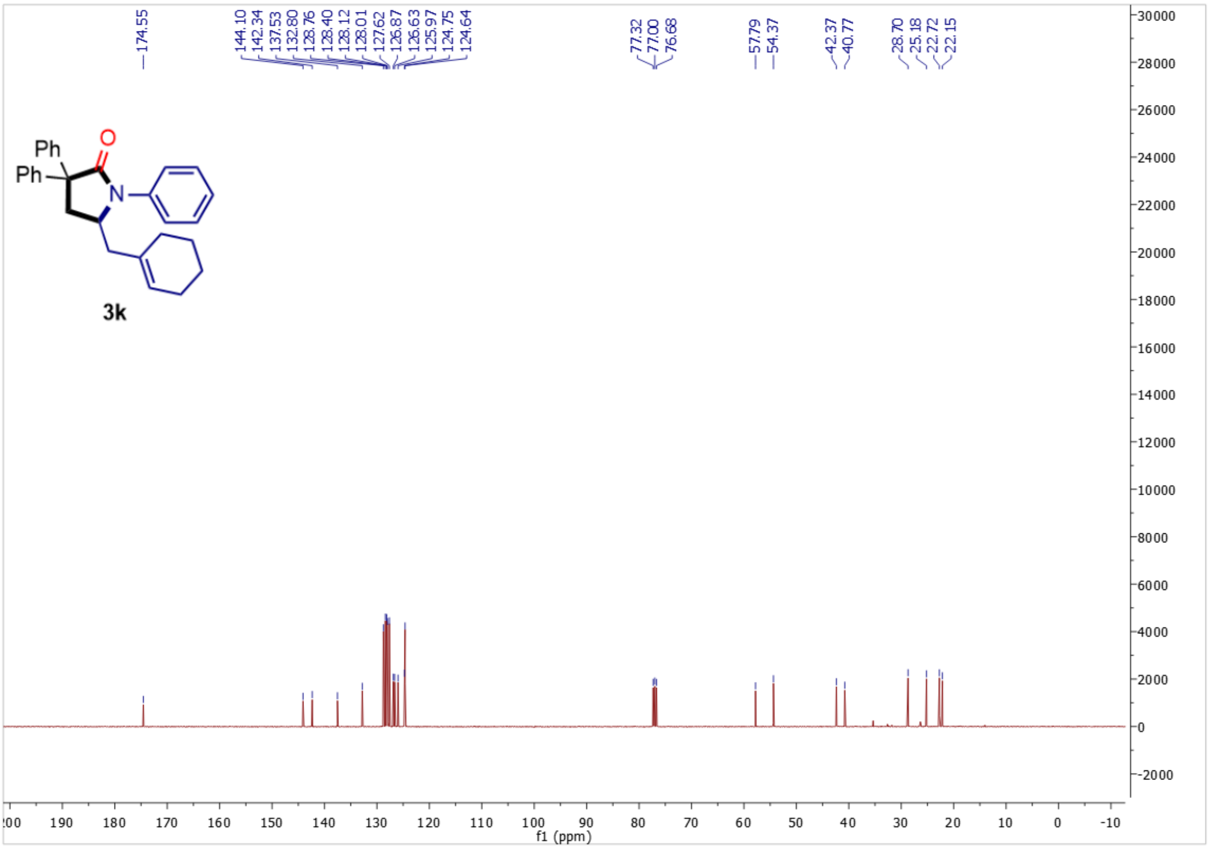

3k

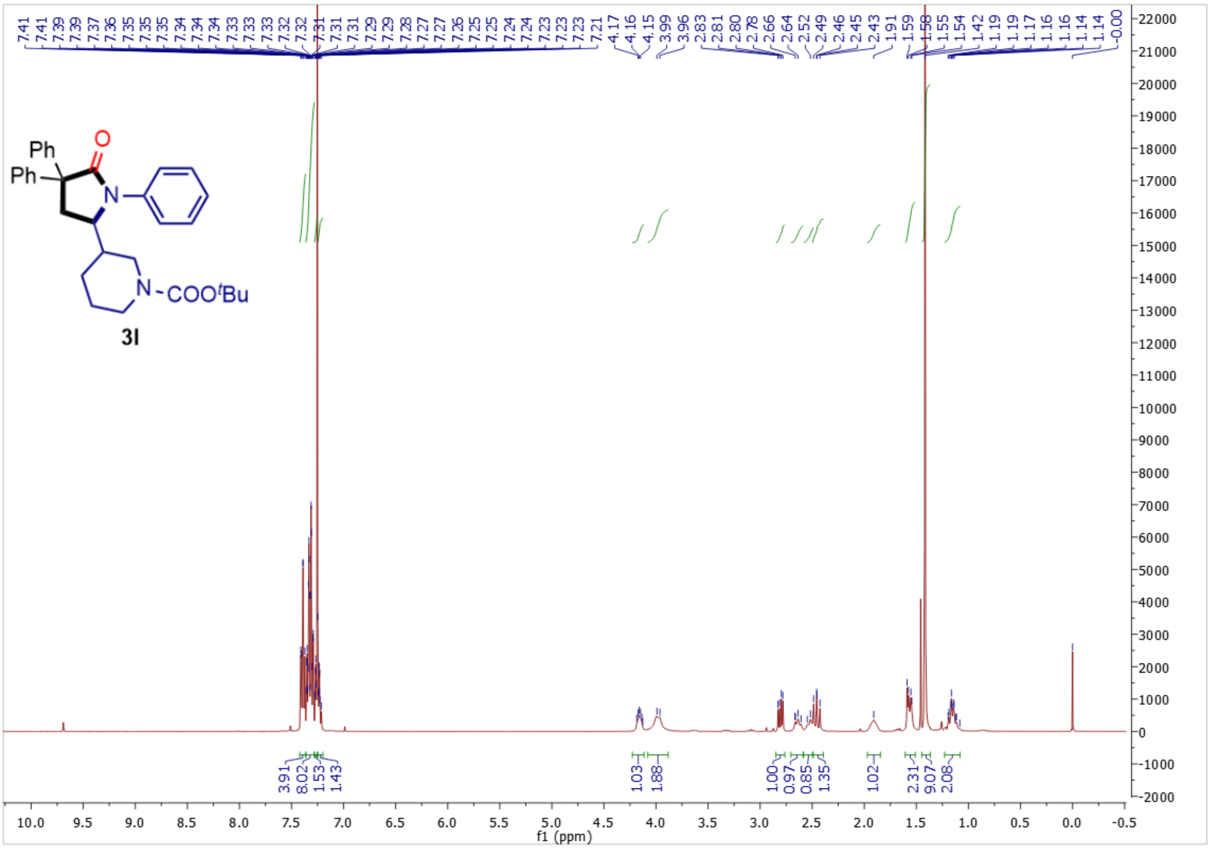

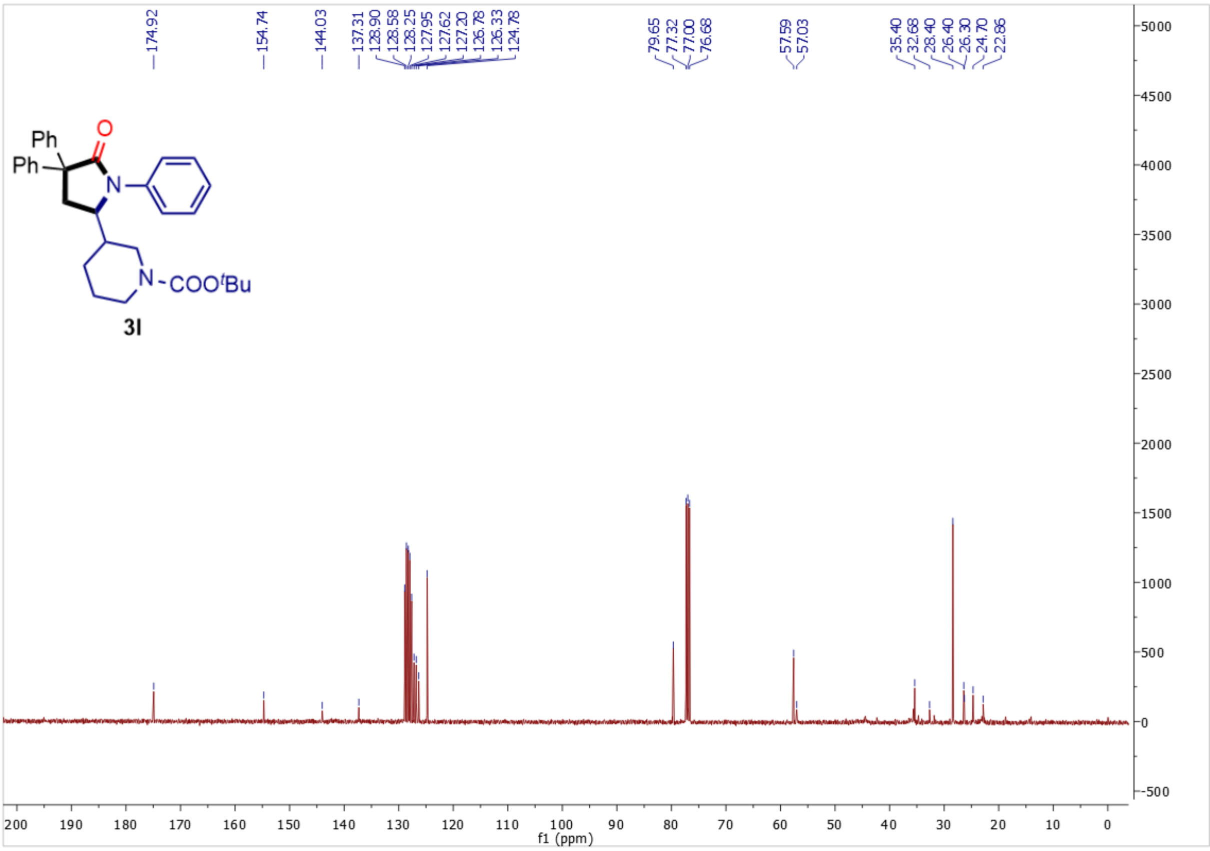

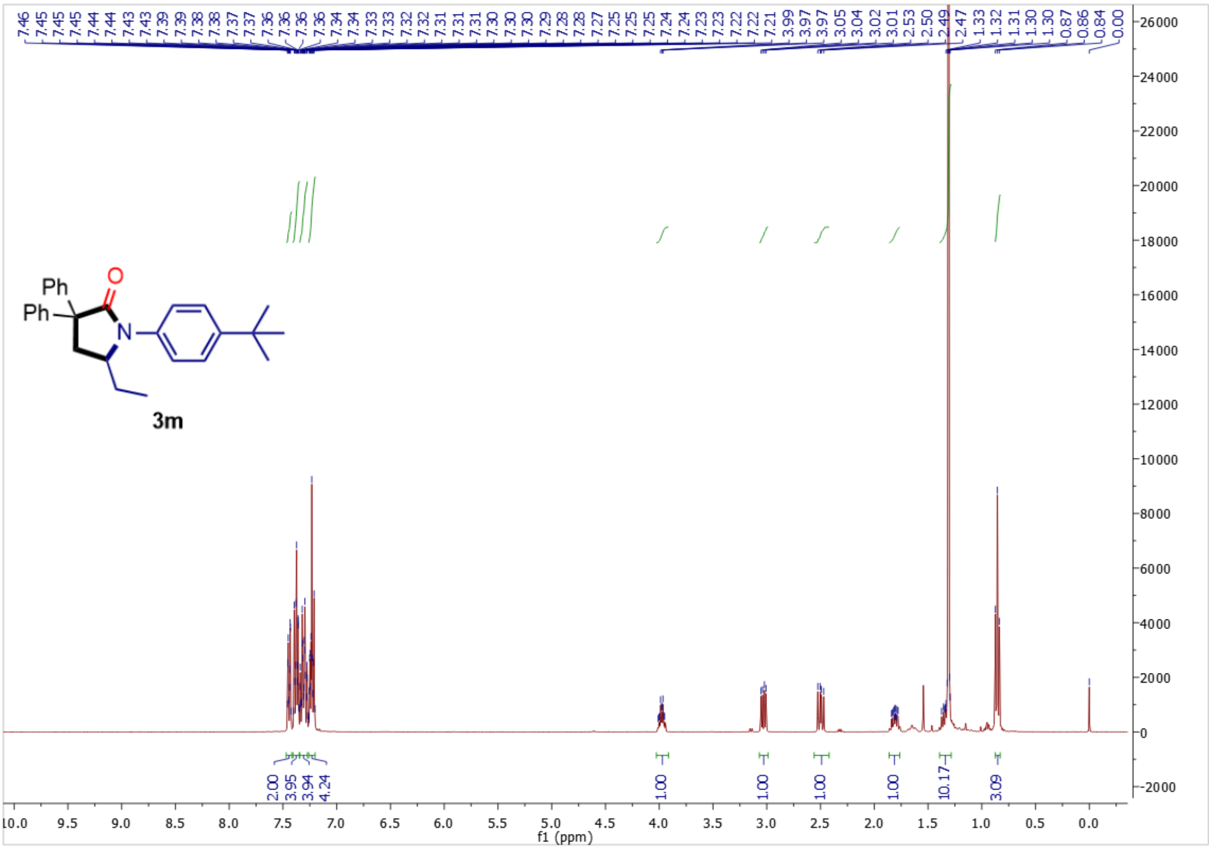

Ph
Ph
O
N
3m
f1 (ppm)

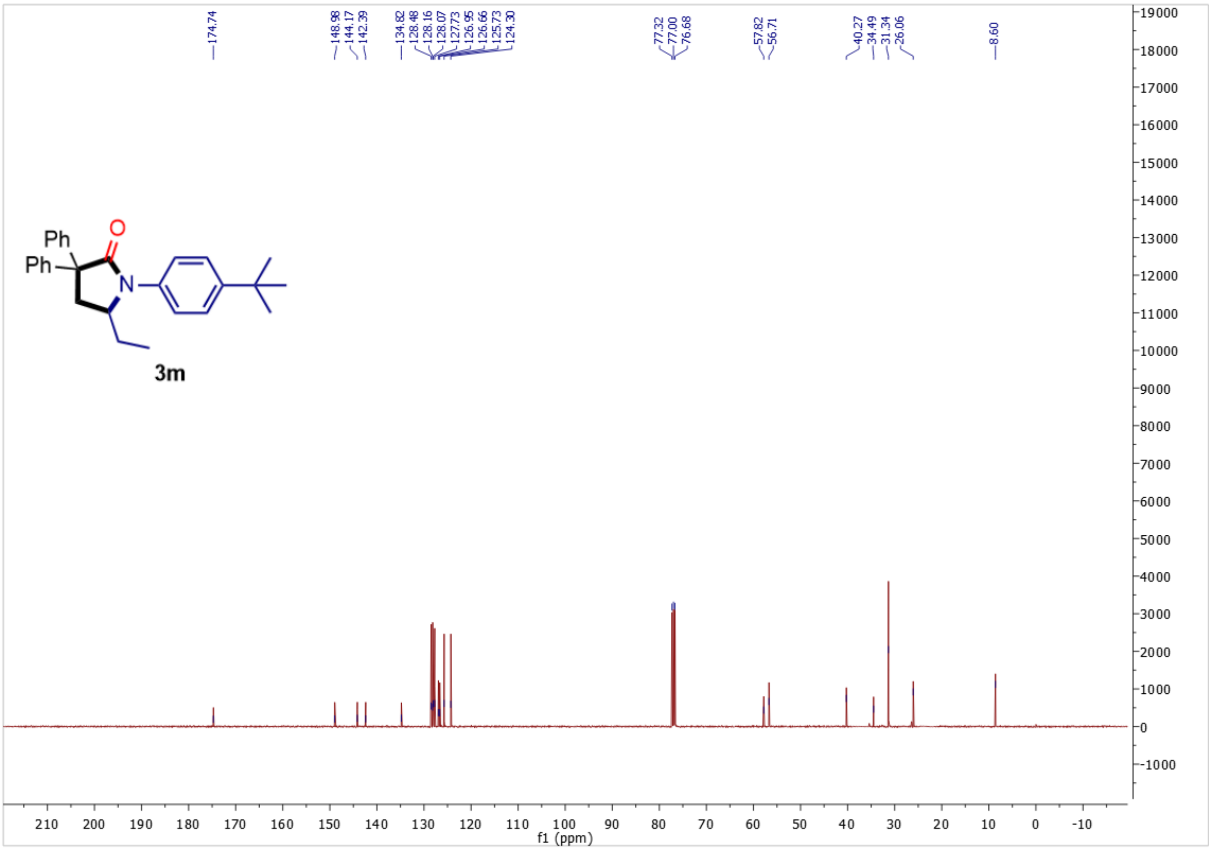

Ph
Ph
O
N
3m
f1 (ppm)

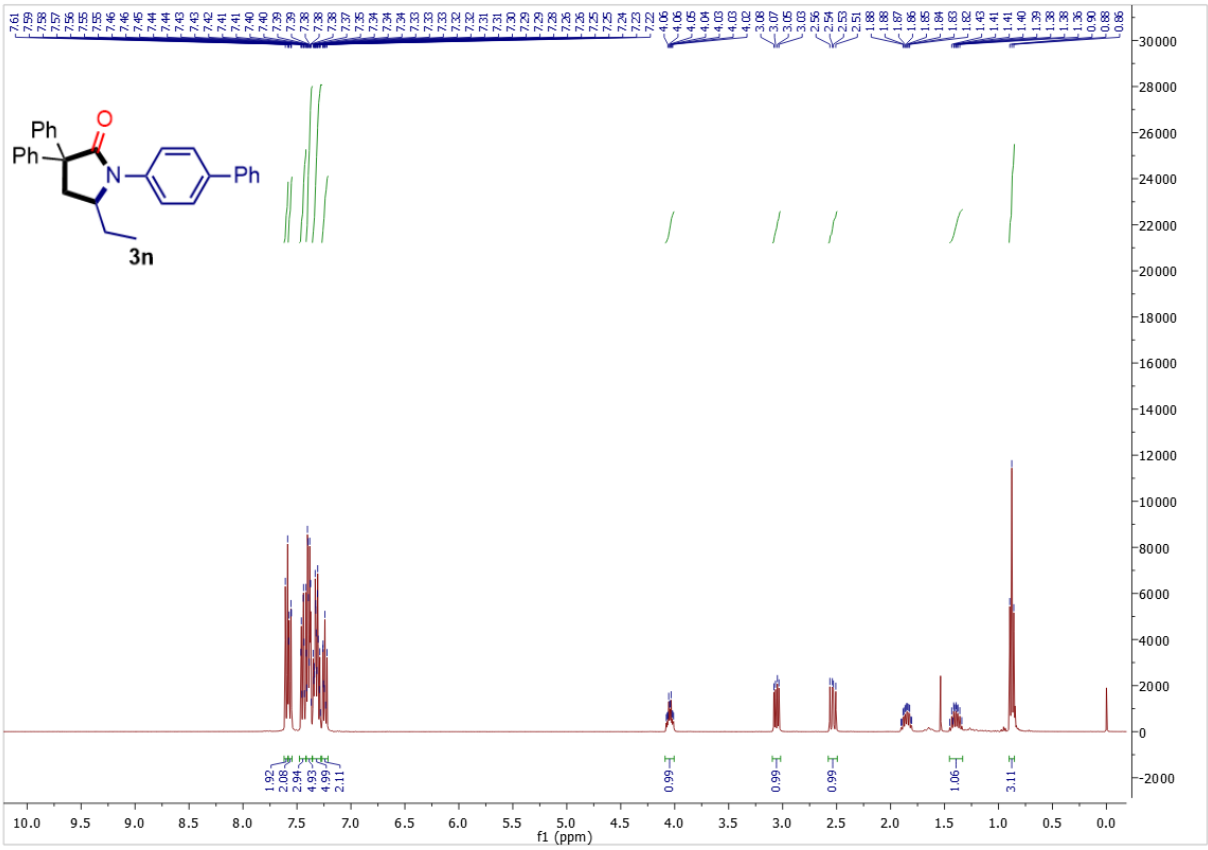

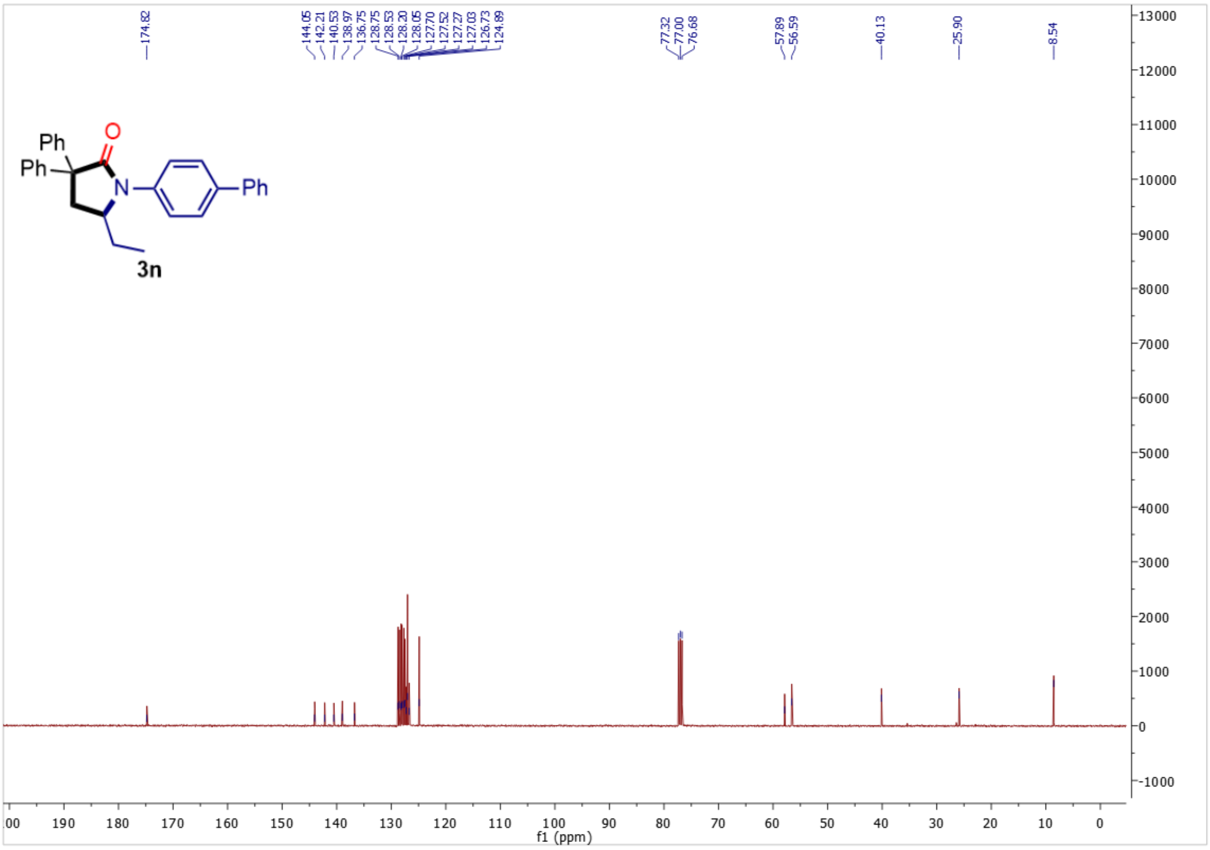

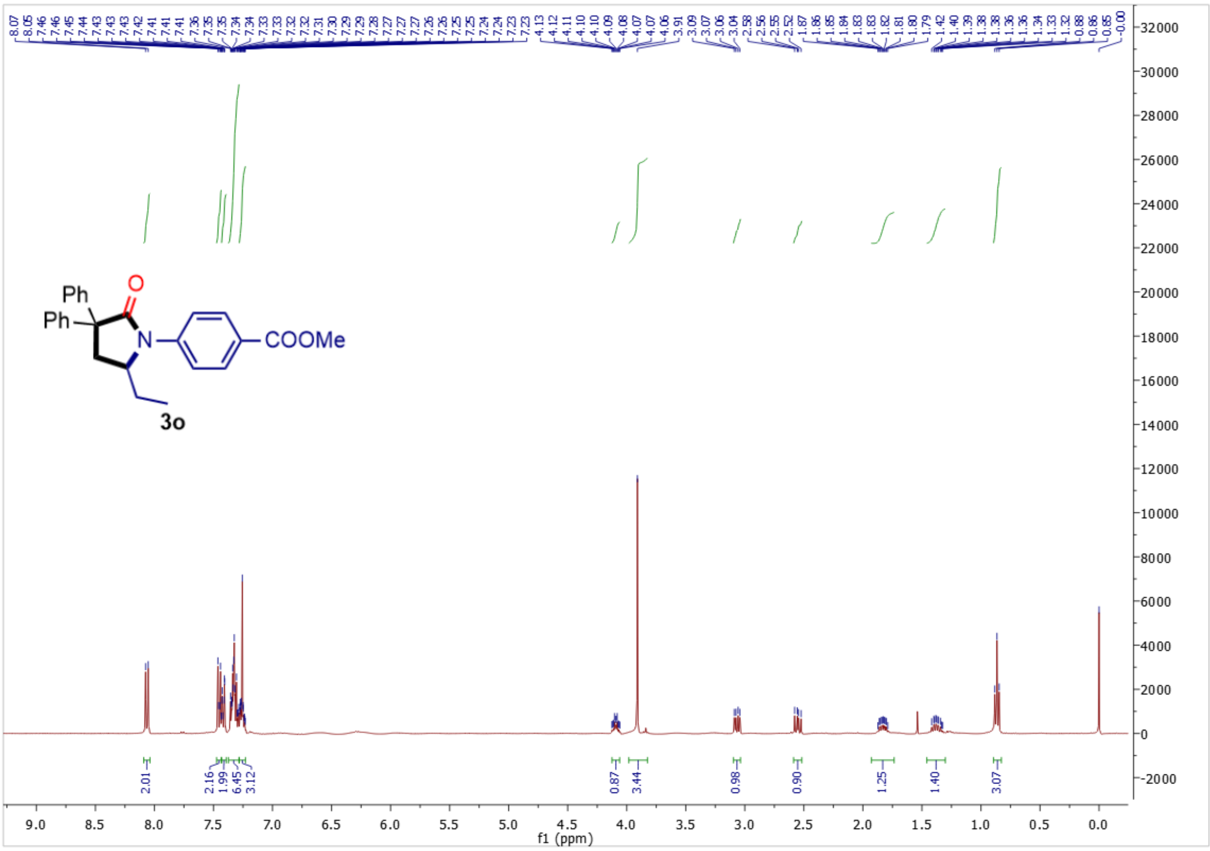

3o

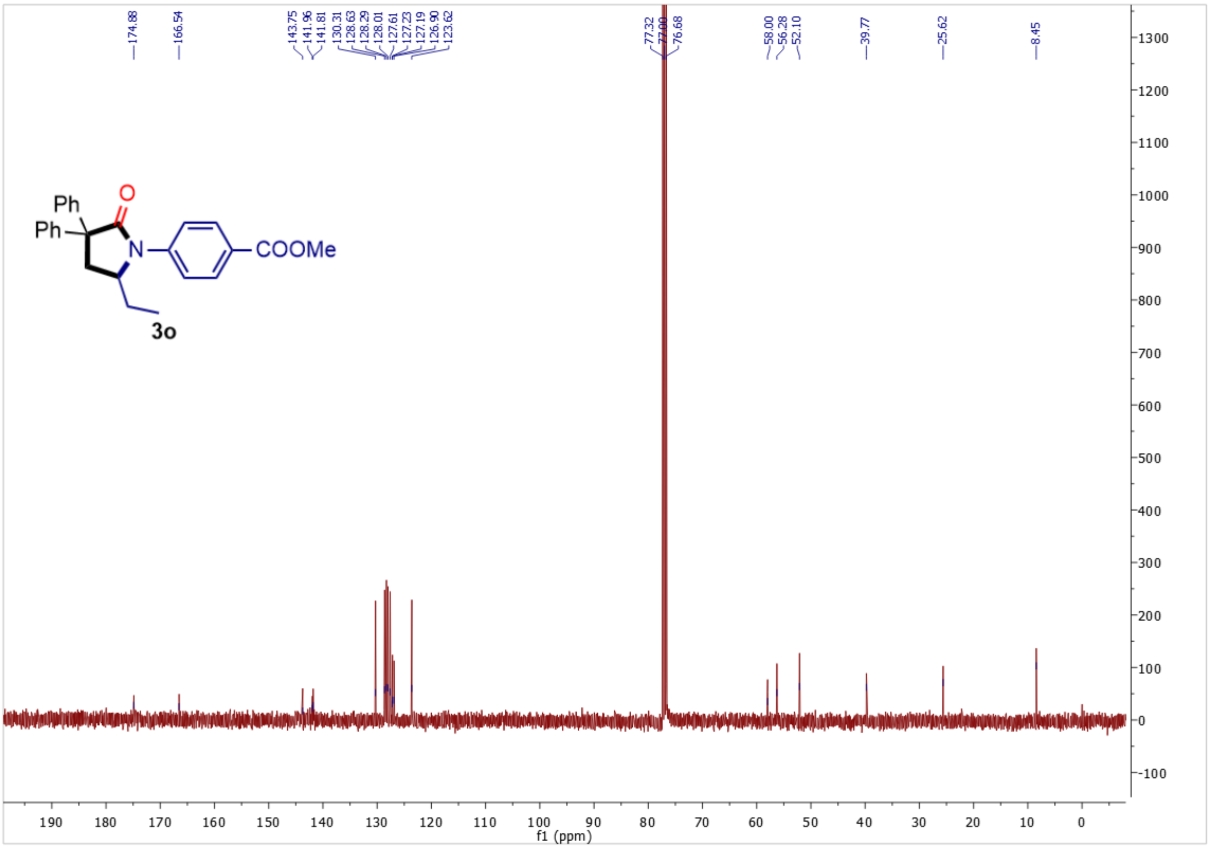

3o

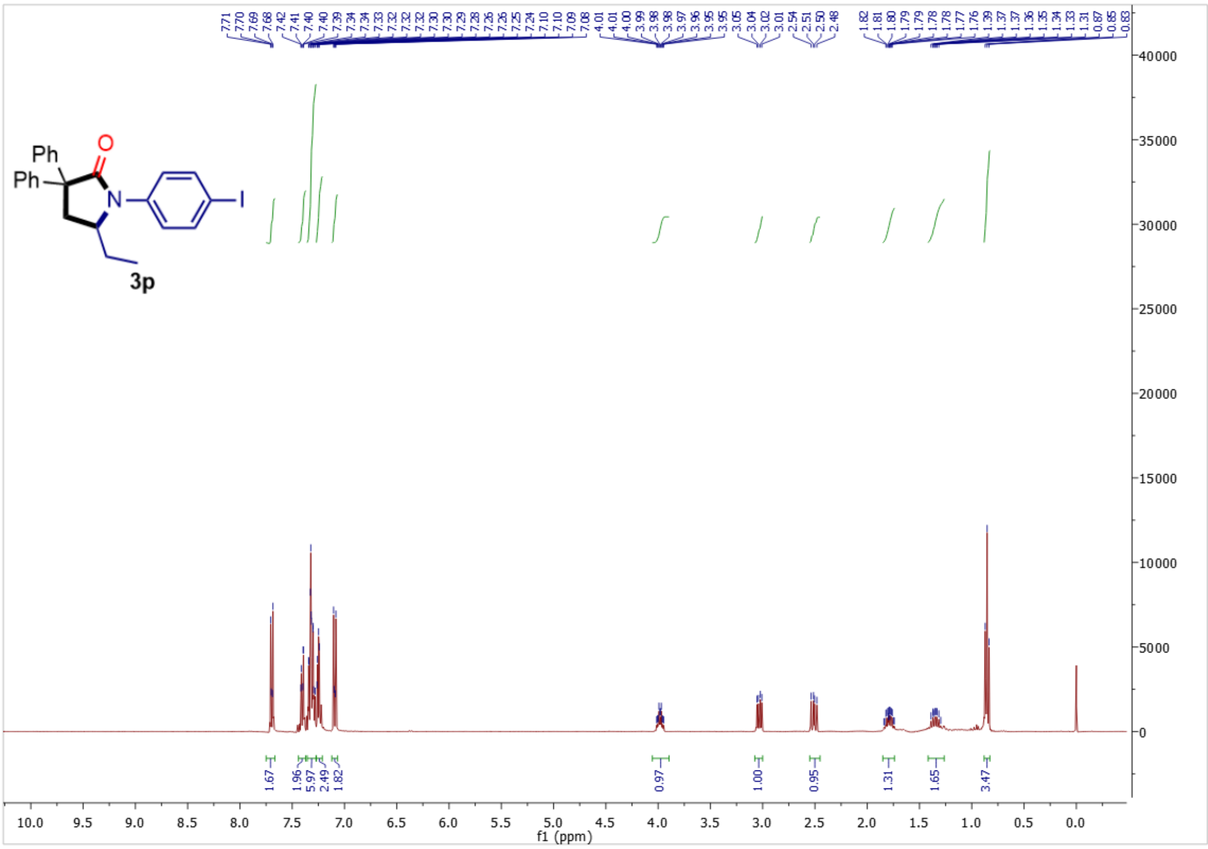

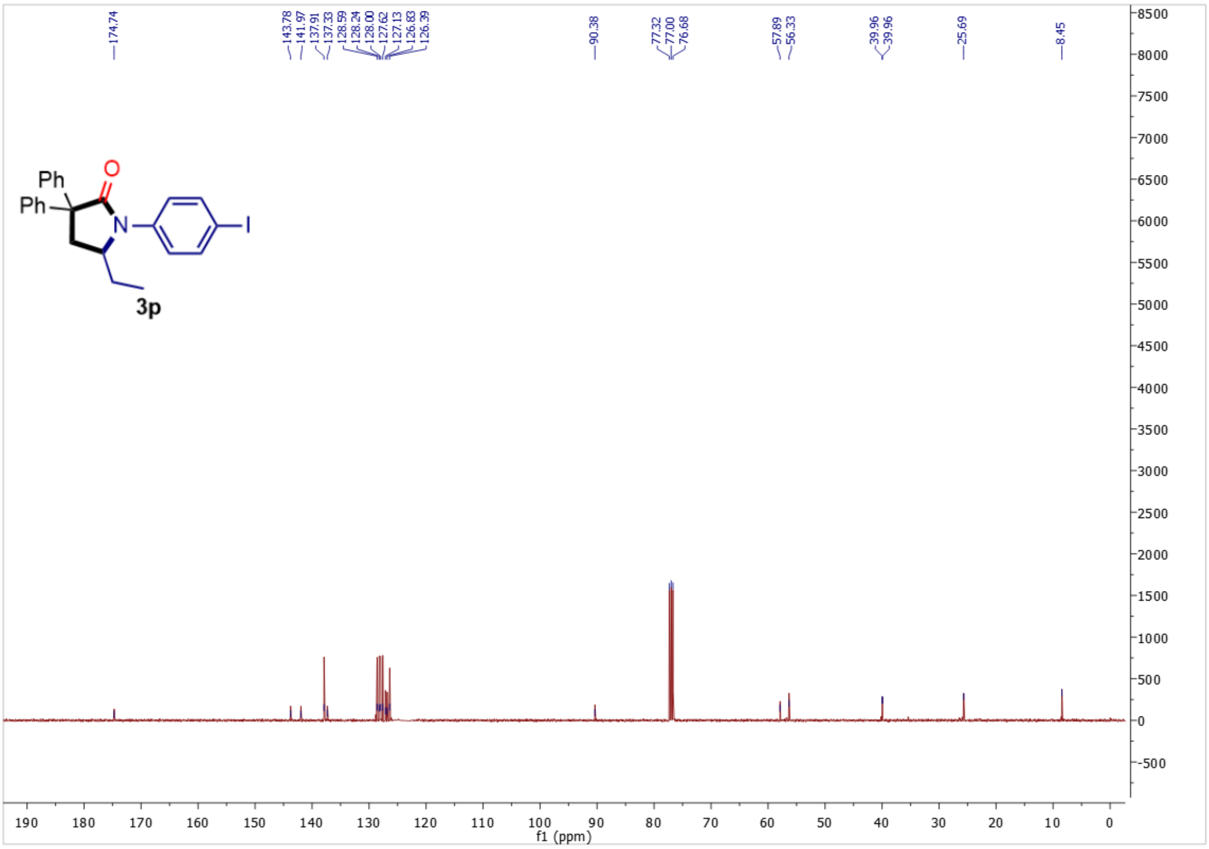

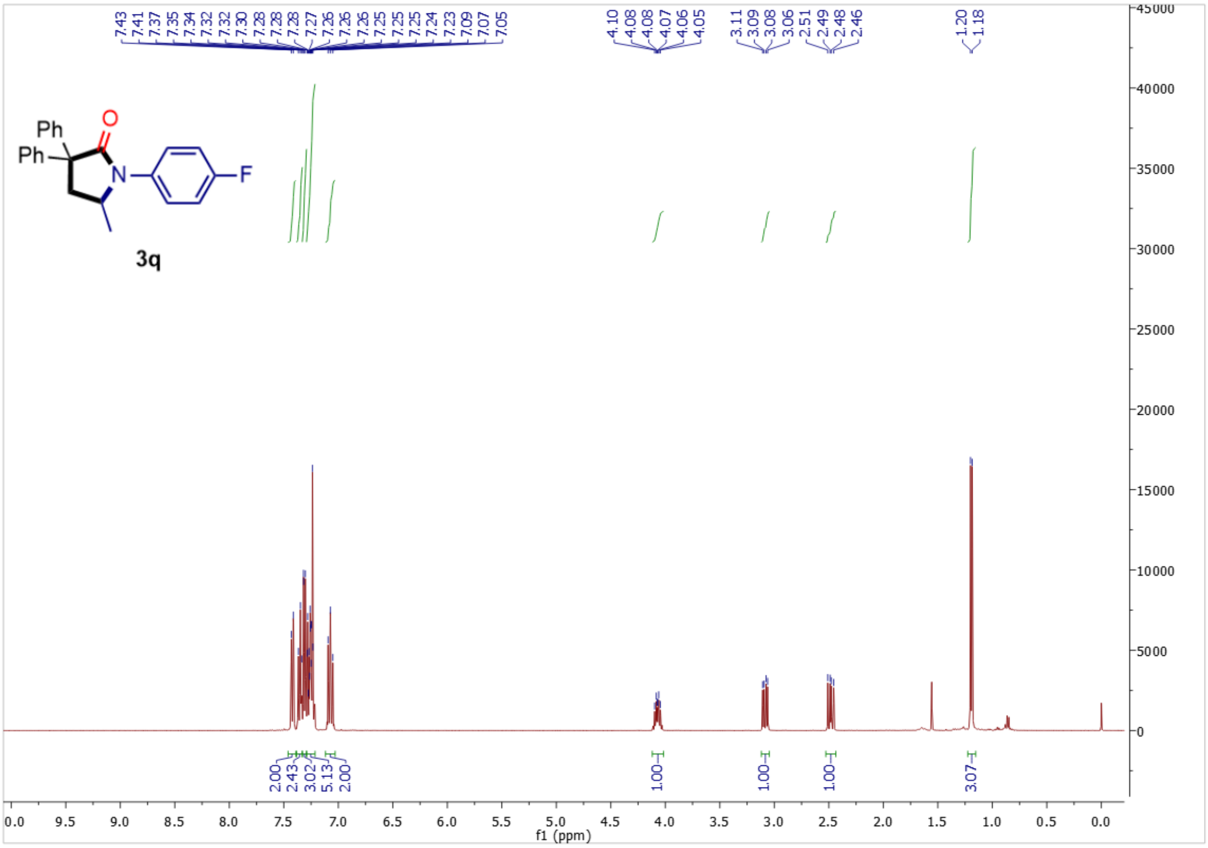

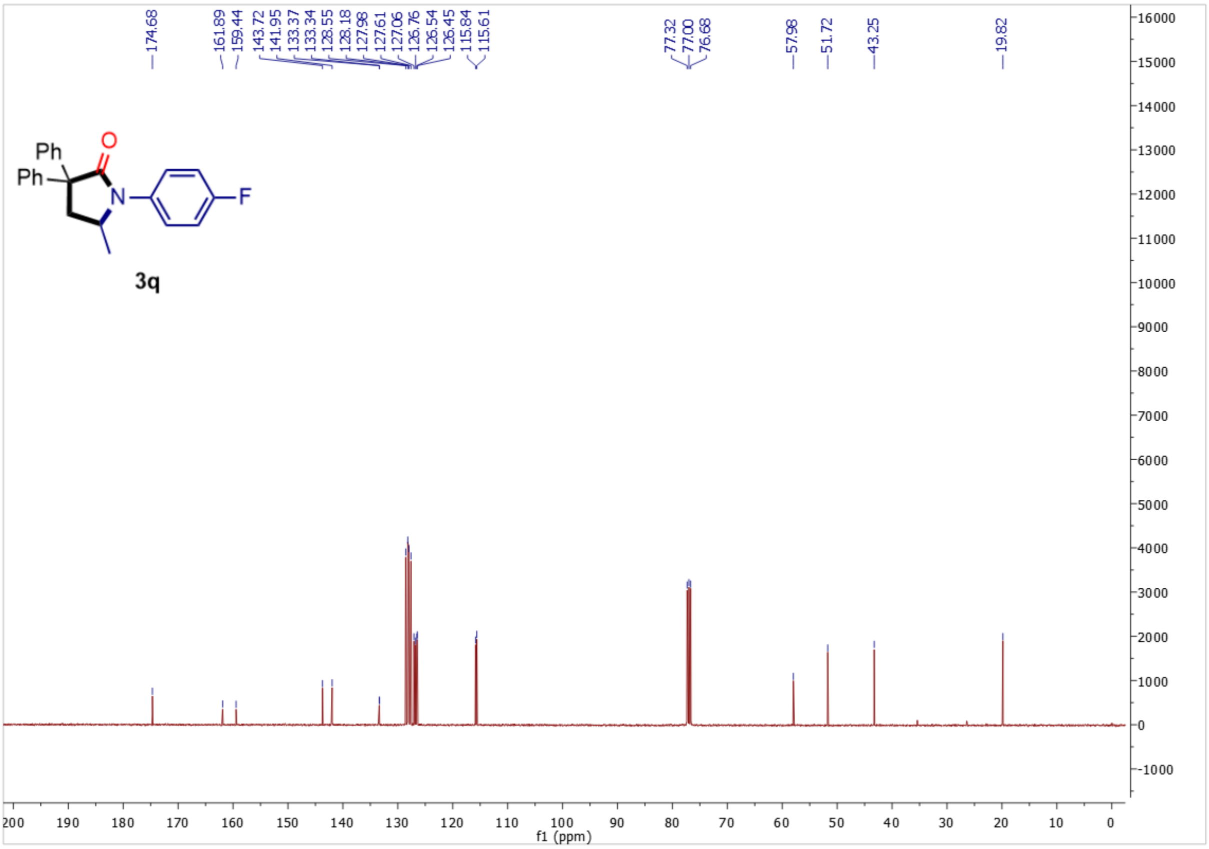

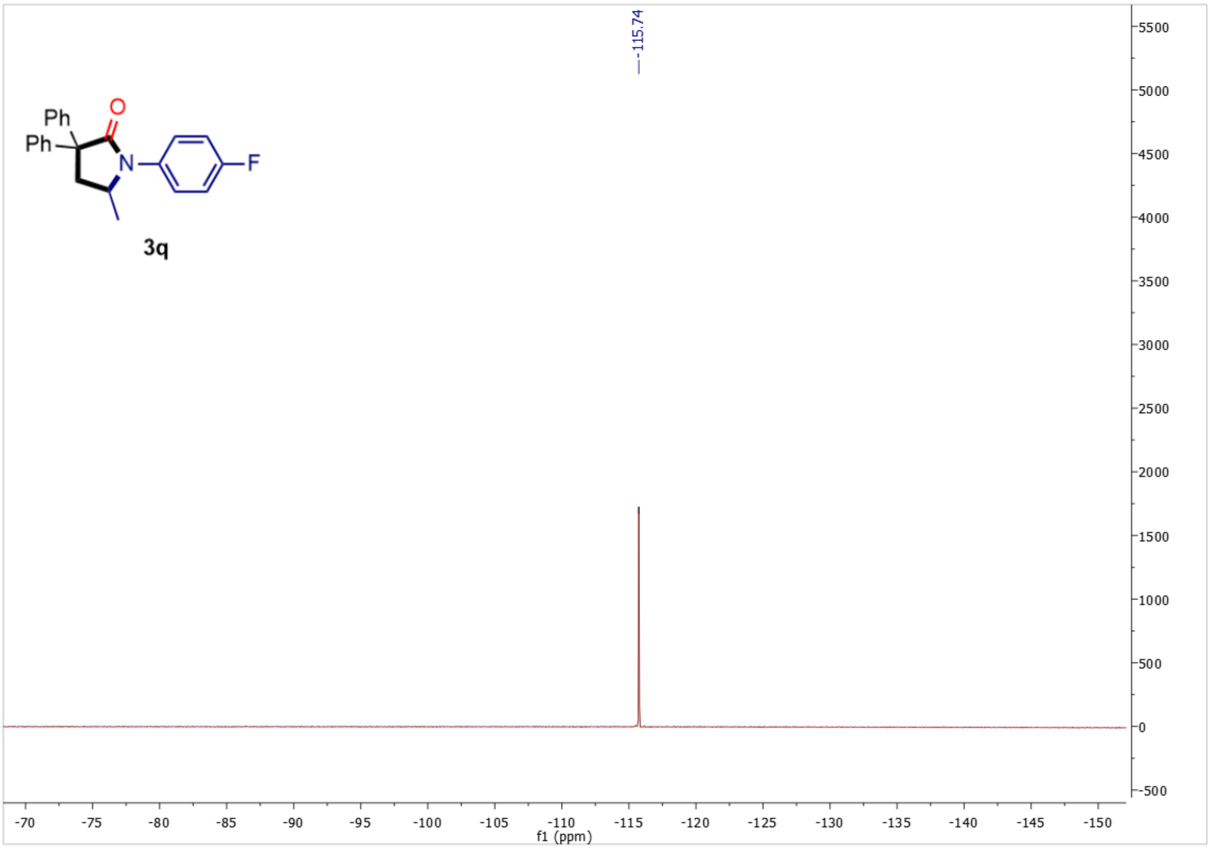

Ph
Ph
O
N
F
3q
-115.74
5500
5000
4500
4000
3500
3000
2500
2000
1500
1000
500
0
-500
-70 -75 -80 -85 -90 -95 -100 -105 -110 -115 -120 -125 -130 -135 -140 -145 -150
f1 (ppm)

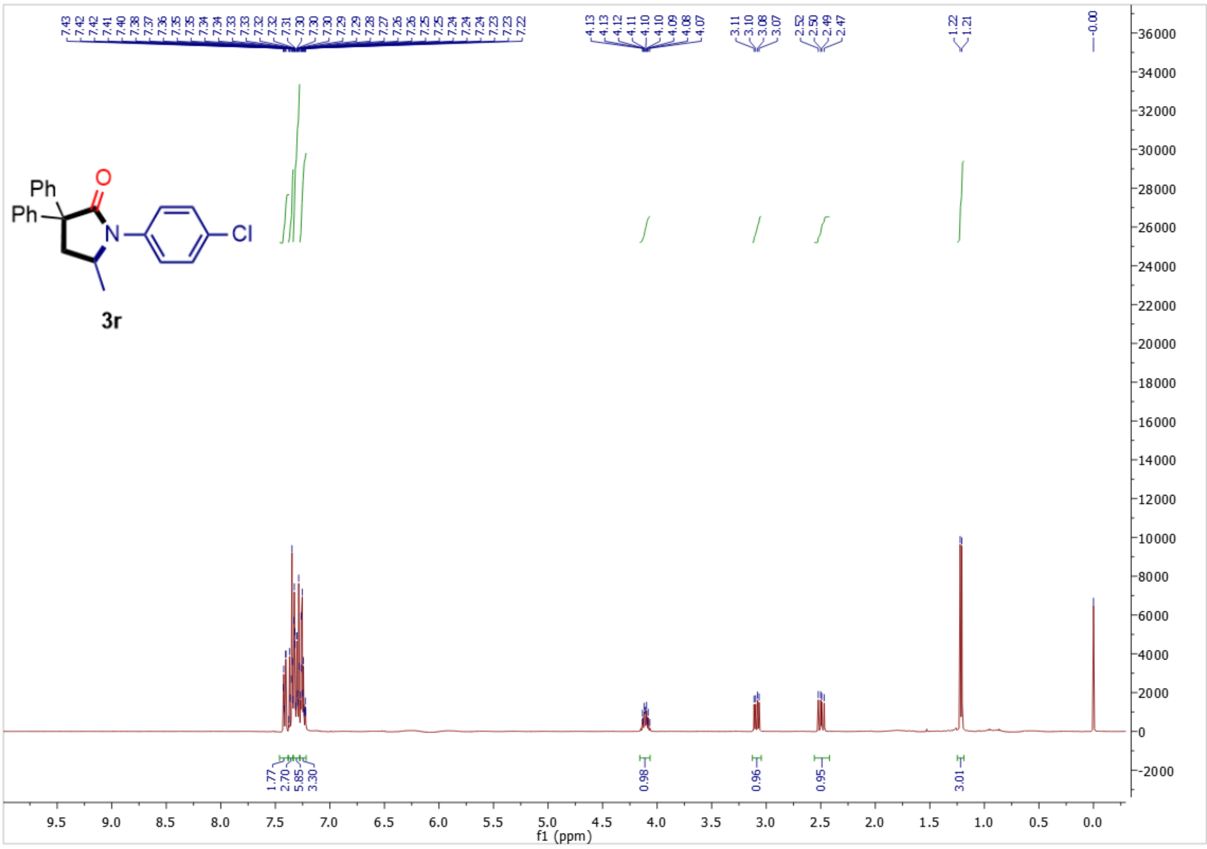

Ph
Ph
O
N
Cl
3r
7.43 7.42 7.42 7.41 7.40 7.38 7.37 7.36 7.35 7.35 7.34 7.34 7.33 7.33 7.32 7.32 7.31 7.30 7.30 7.30 7.29 7.29 7.28 7.27 7.26 7.26 7.25 7.24 7.24 7.23 7.23 7.22
4.13 4.13 4.12 4.11 4.10 4.10 4.09 4.08 4.07
3.11 3.10 3.08 3.07
2.52 2.50 2.49 2.47
1.22 1.21
-0.00
1.77 2.70 5.85 3.30
0.98
0.96
0.95
3.01
36000
34000
32000
30000
28000
26000
24000
22000
20000
18000
16000
14000
12000
10000
8000
6000
4000
2000
0
-2000
9.5 9.0 8.5 8.0 7.5 7.0 6.5 6.0 5.5 5.0 4.5 4.0 3.5 3.0 2.5 2.0 1.5 1.0 0.5 0.0
f1 (ppm)

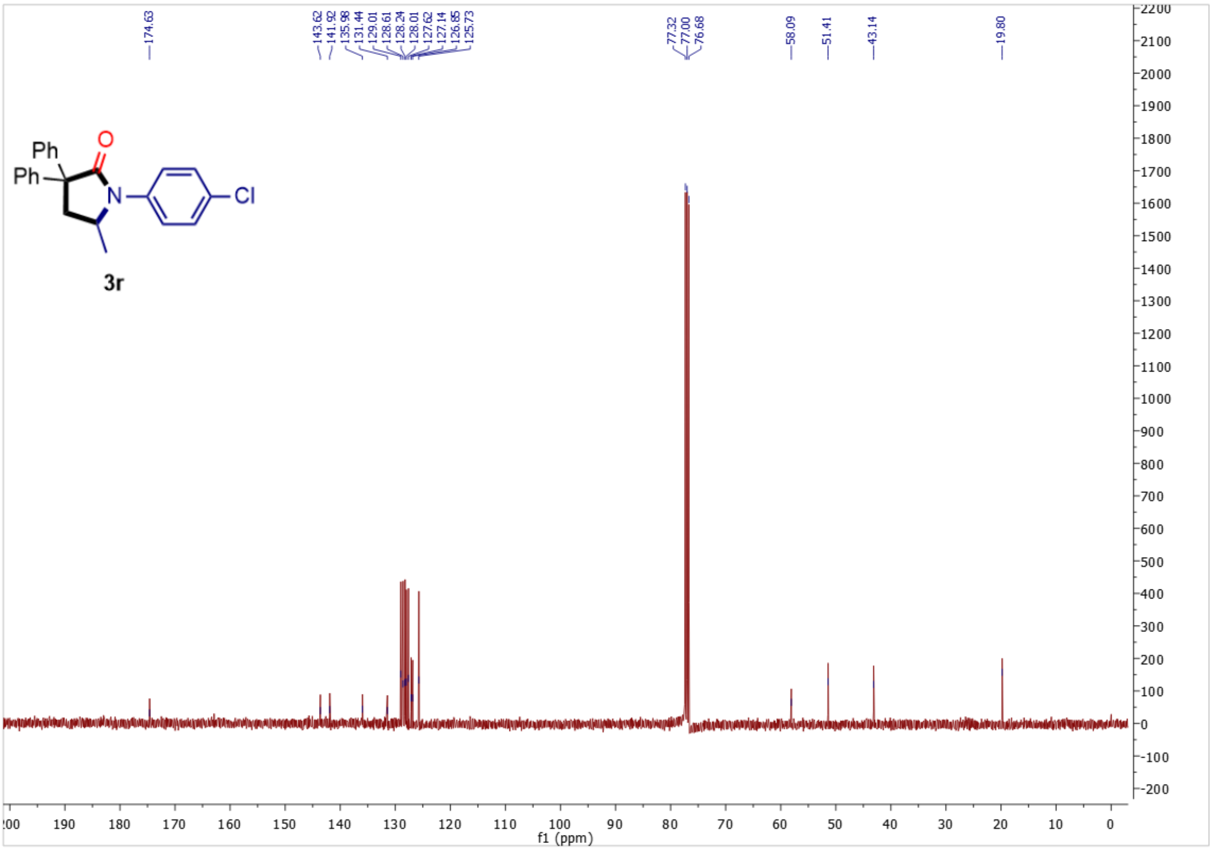
174.63
143.62
141.92
135.98
131.44
129.01
128.61
128.24
128.01
127.62
127.14
126.85
125.73
77.32
77.00
76.68
58.09
51.41
43.14
19.80
Ph
Ph
O
N
Cl
3r
f1 (ppm)

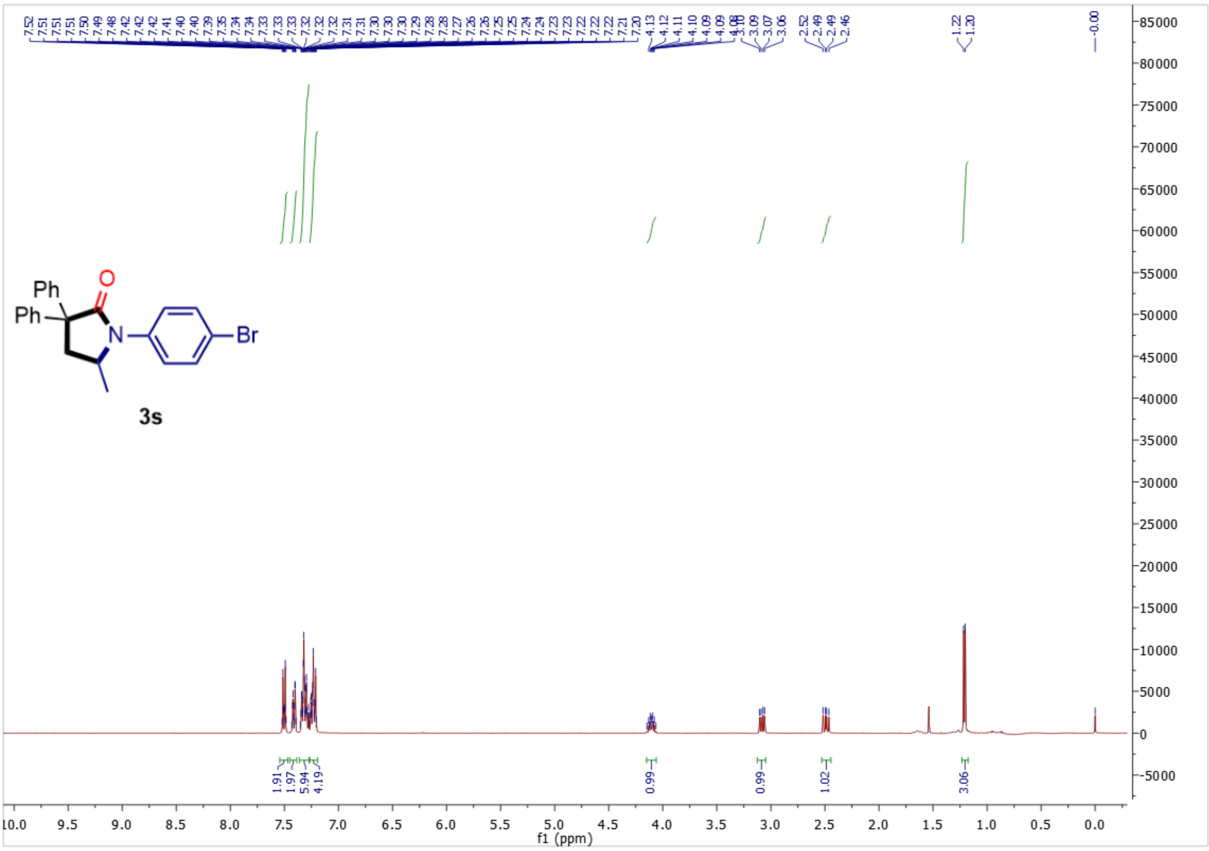
Ph
Ph
O
N
Br
3s
1.91
1.97
5.94
4.19
0.99
0.99
1.02
3.06
f1 (ppm)

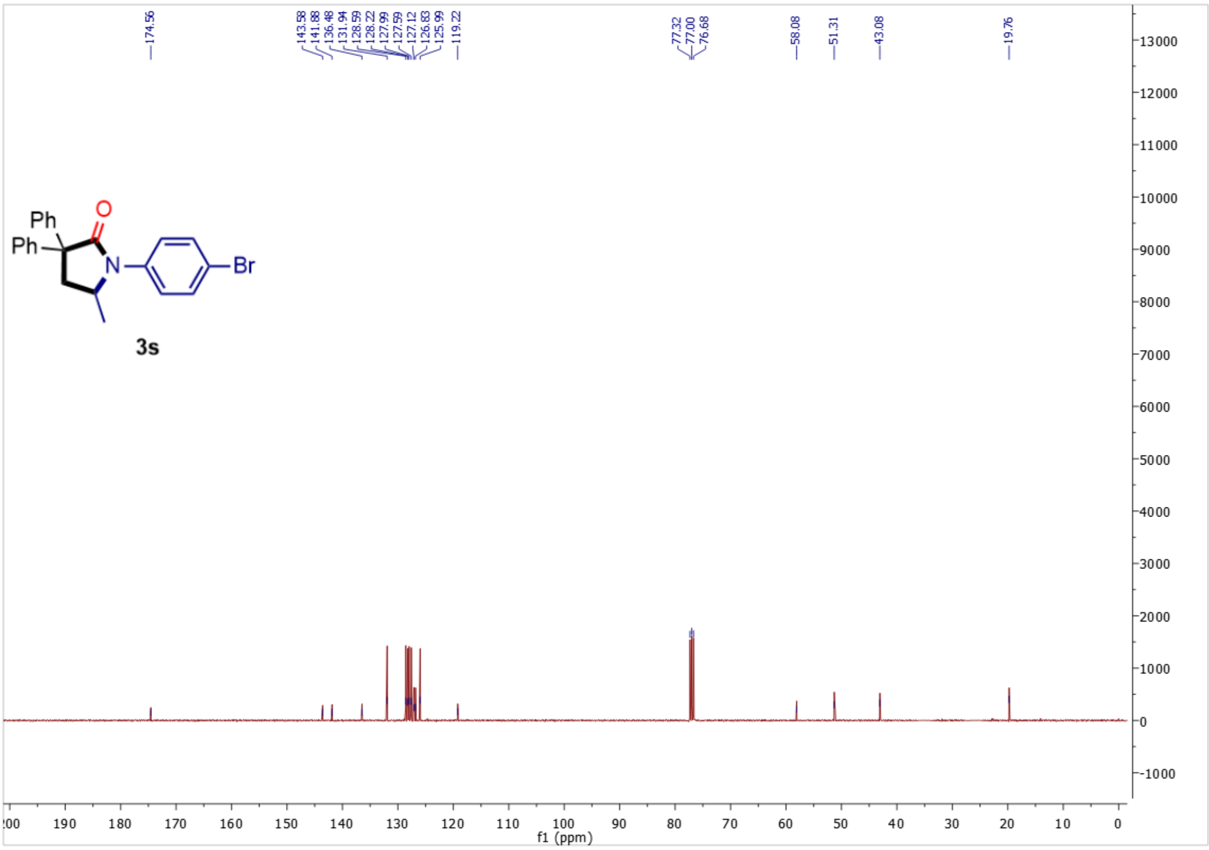
174.56
143.58
141.88
136.48
131.94
128.59
128.22
127.99
127.59
127.12
126.83
125.99
119.22
77.32
77.00
76.68
58.08
51.31
43.08
19.76
Ph
Ph
O
N
Br
3s
f1 (ppm)

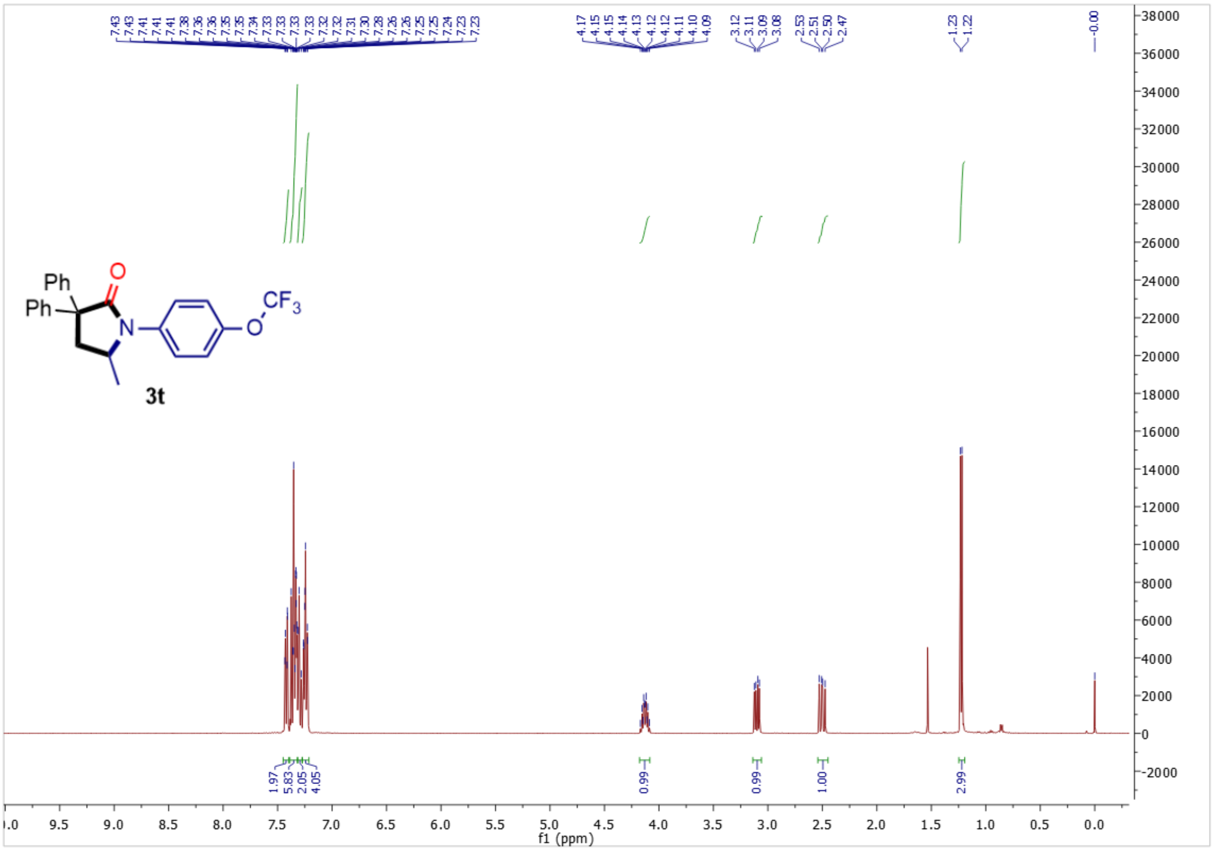
Ph
Ph
O
N
O
CF3
3t
f1 (ppm)

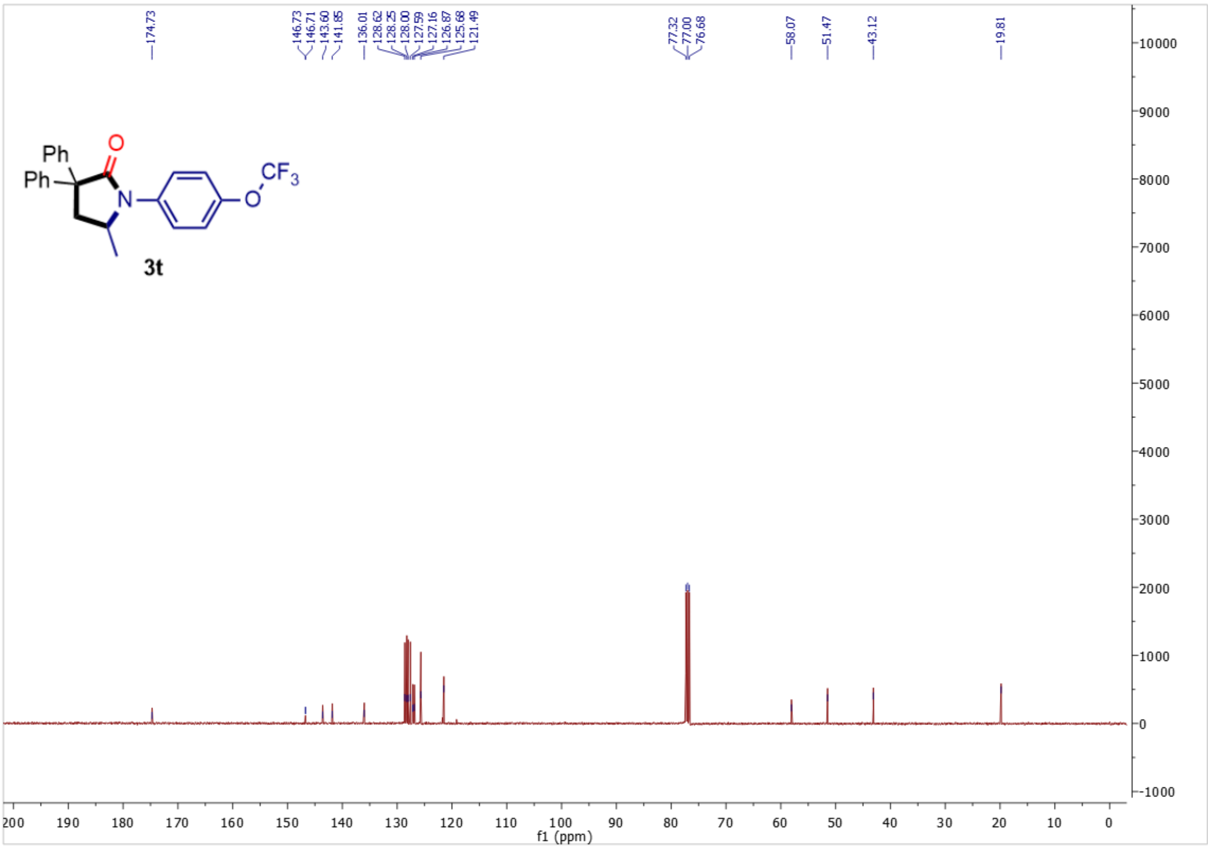
174.73
146.73
146.71
143.60
141.85
136.01
128.62
128.25
128.00
127.59
127.16
126.87
125.68
121.49
77.32
77.00
76.68
58.07
51.47
43.12
19.81
Ph
Ph
O
N
O
CF3
3t
f1 (ppm)

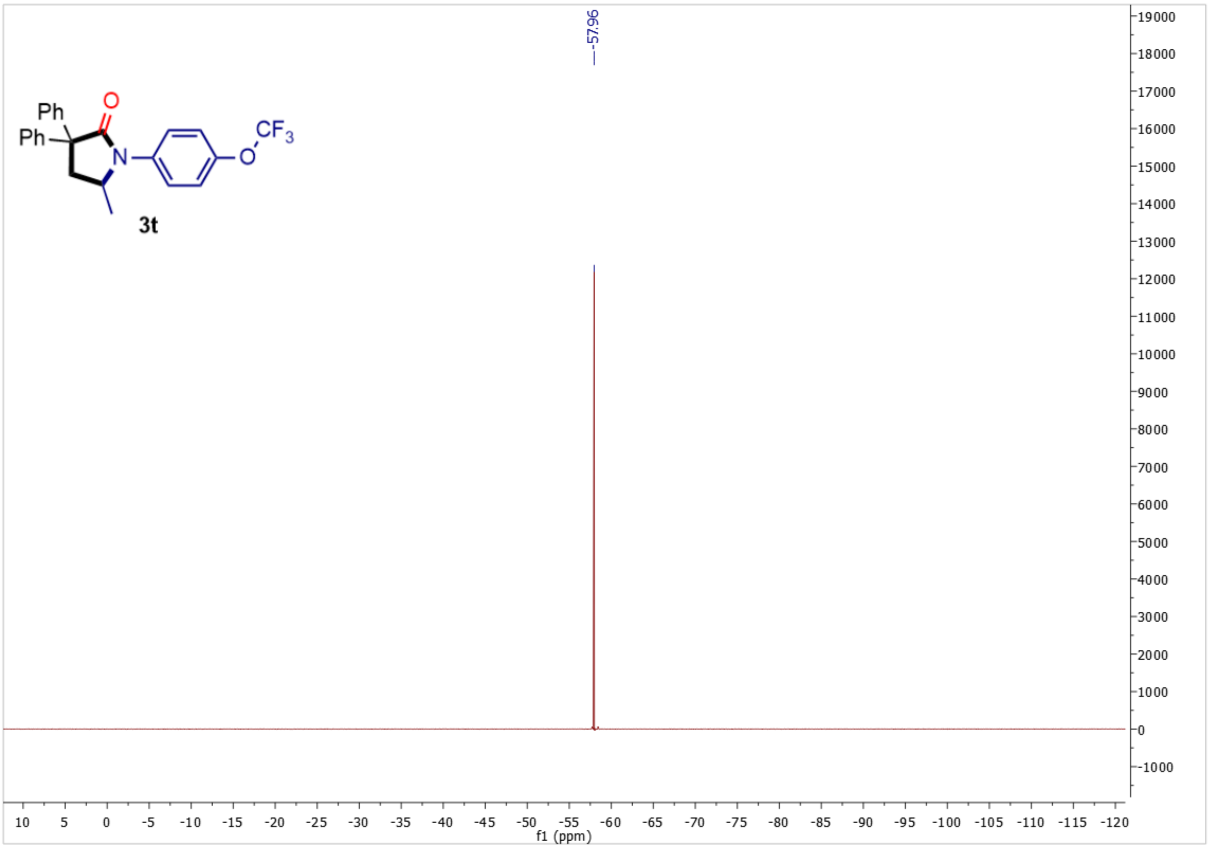
-57.96
Ph
Ph
O
N
O
CF3
3t
f1 (ppm)

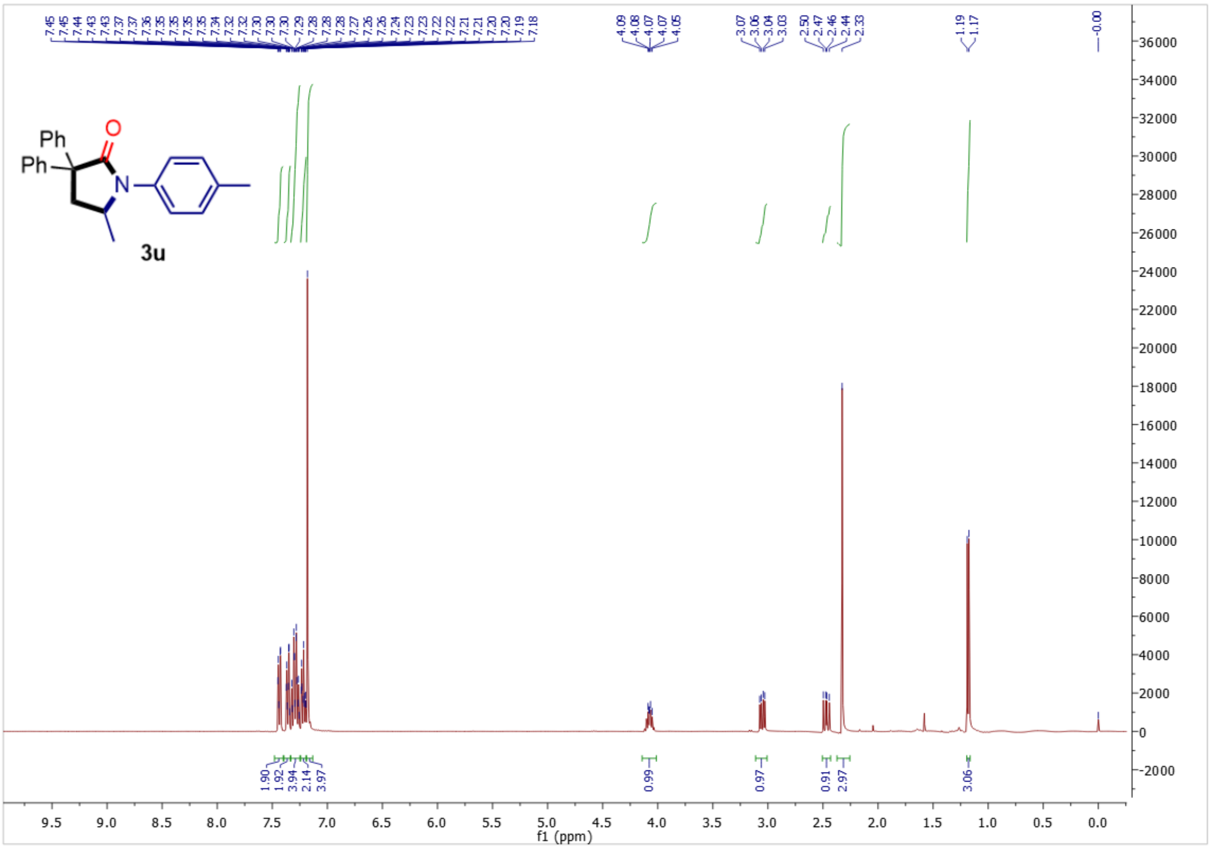
3u
f1 (ppm)

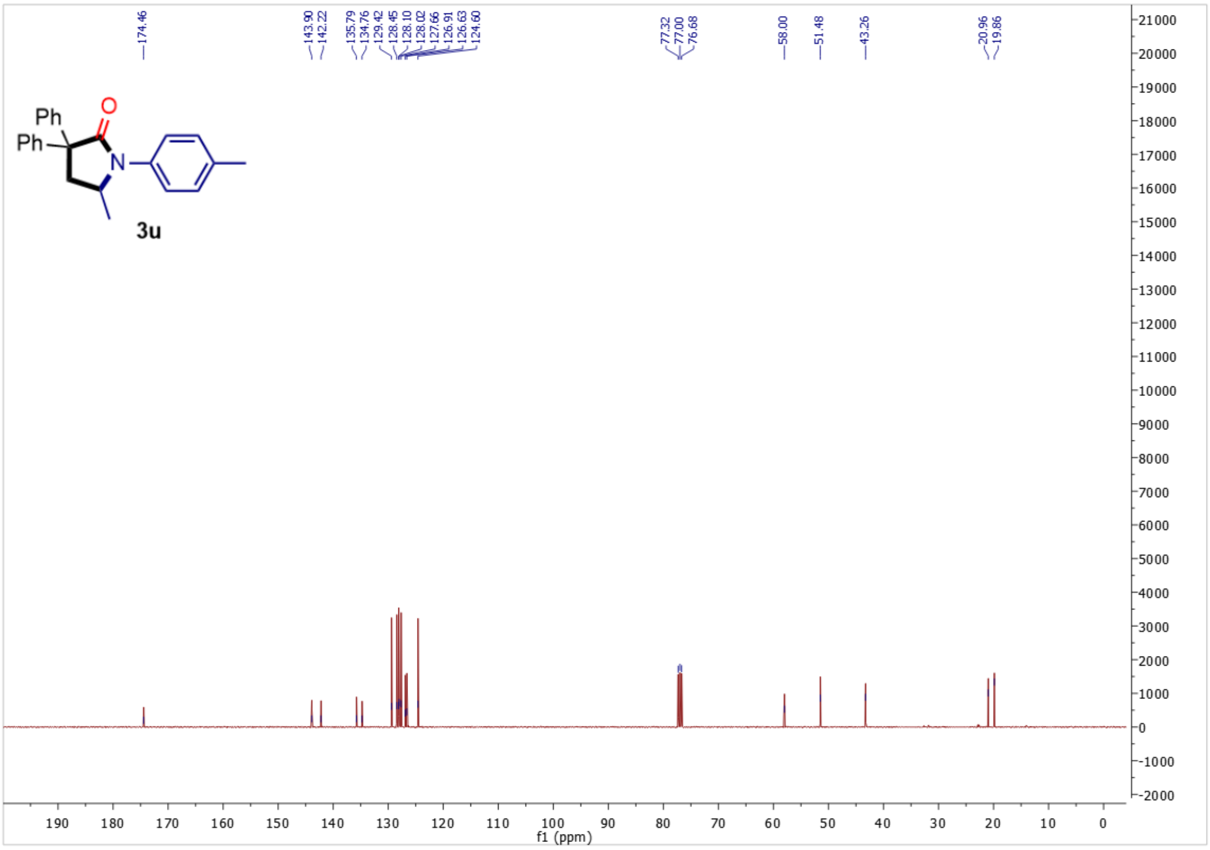
3u
f1 (ppm)

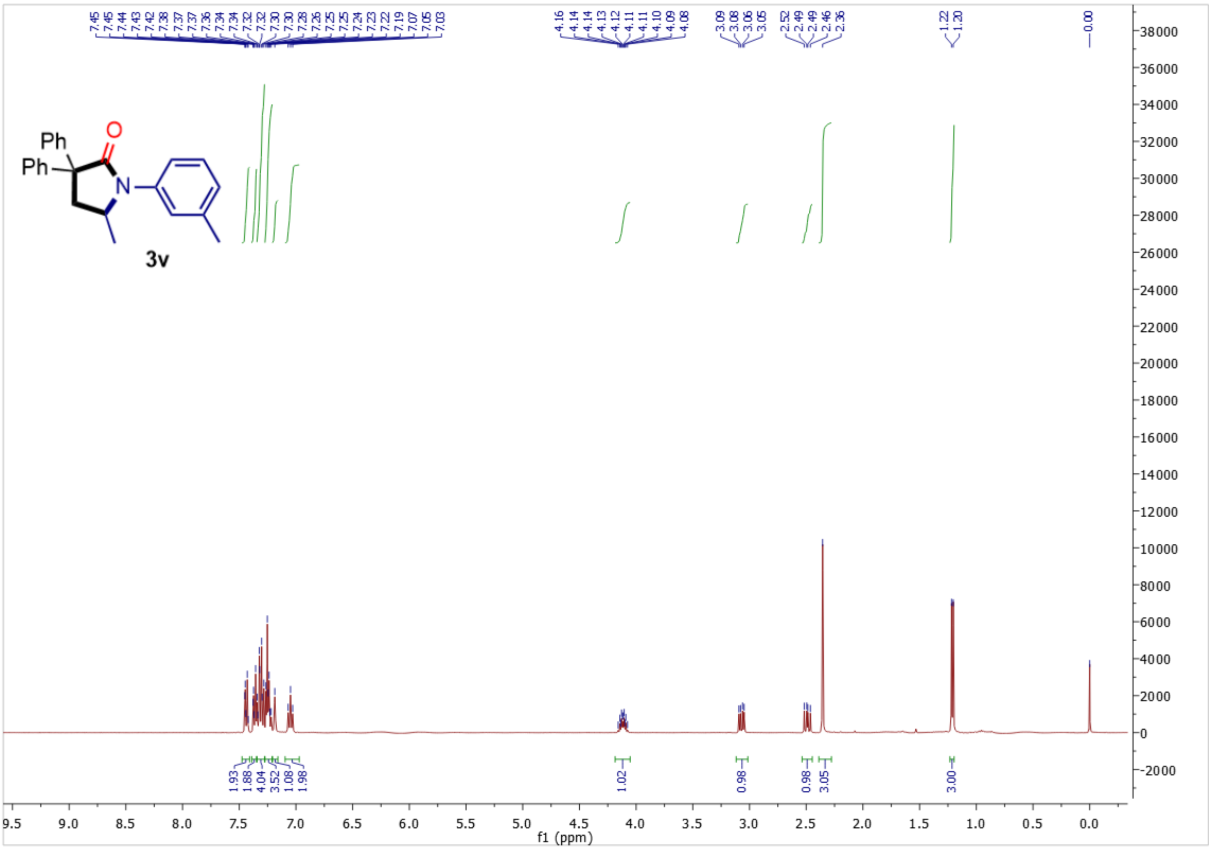

3v
f1 (ppm)

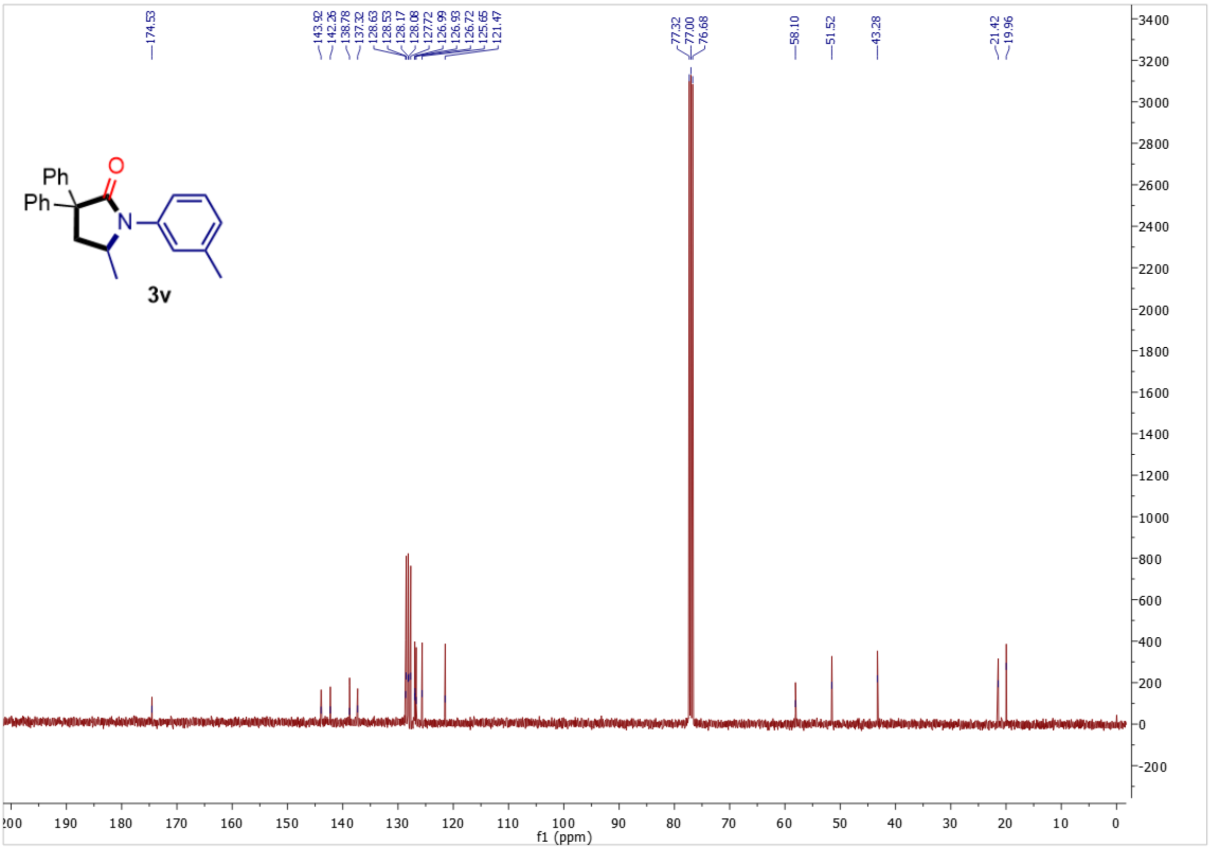

3v
f1 (ppm)

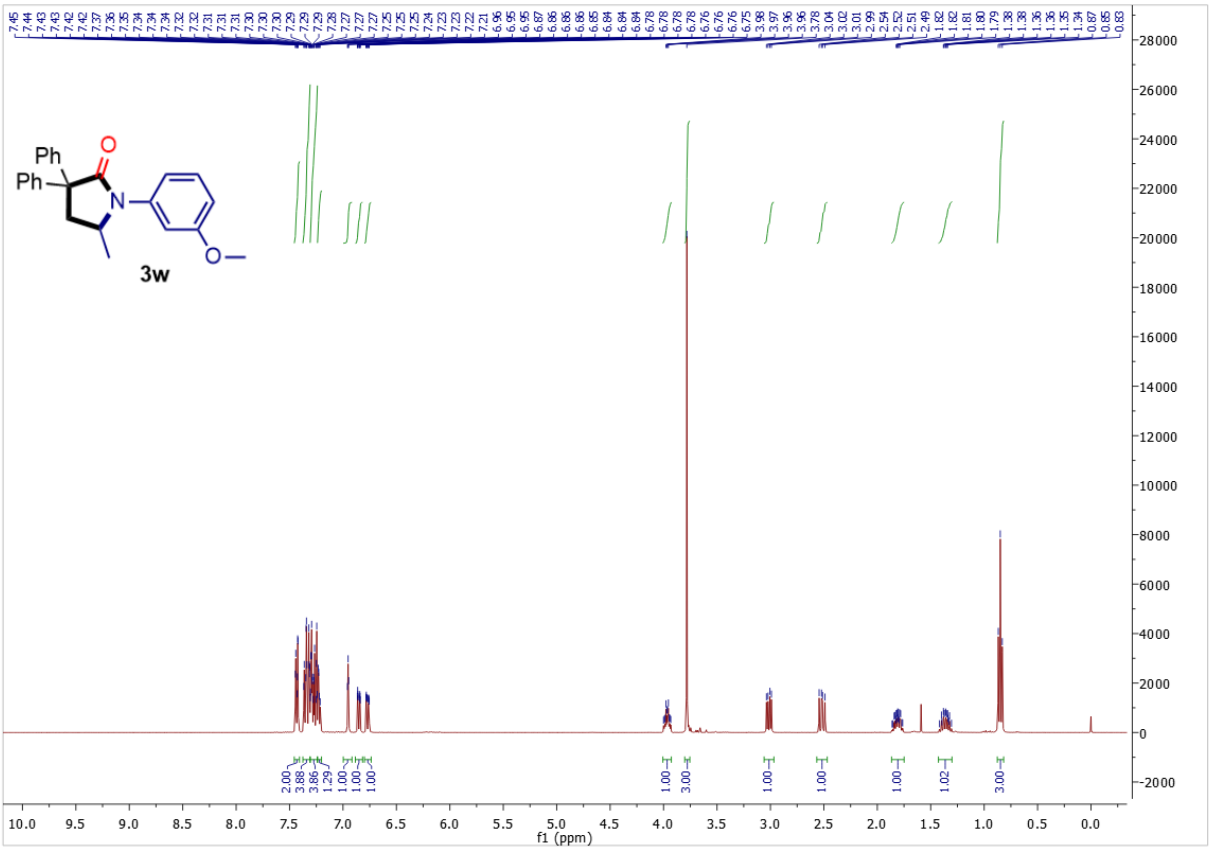

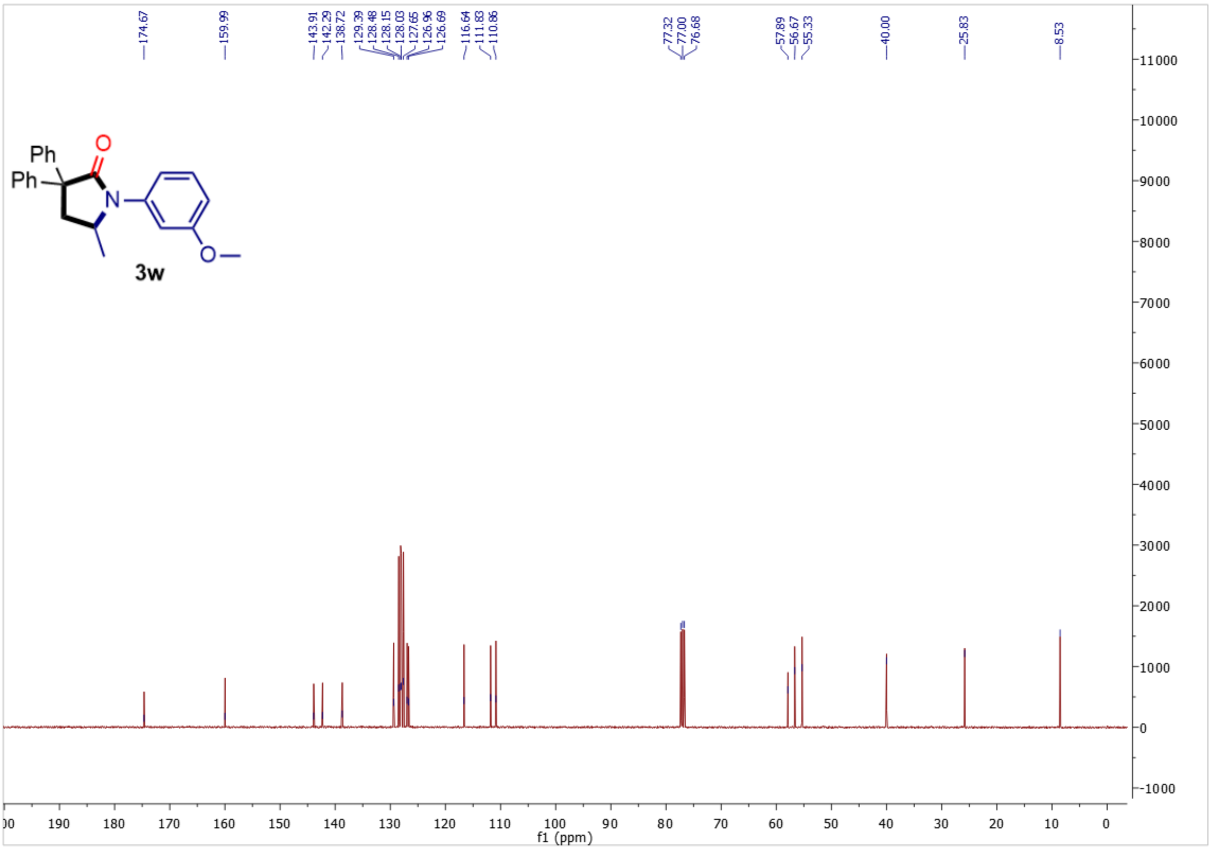

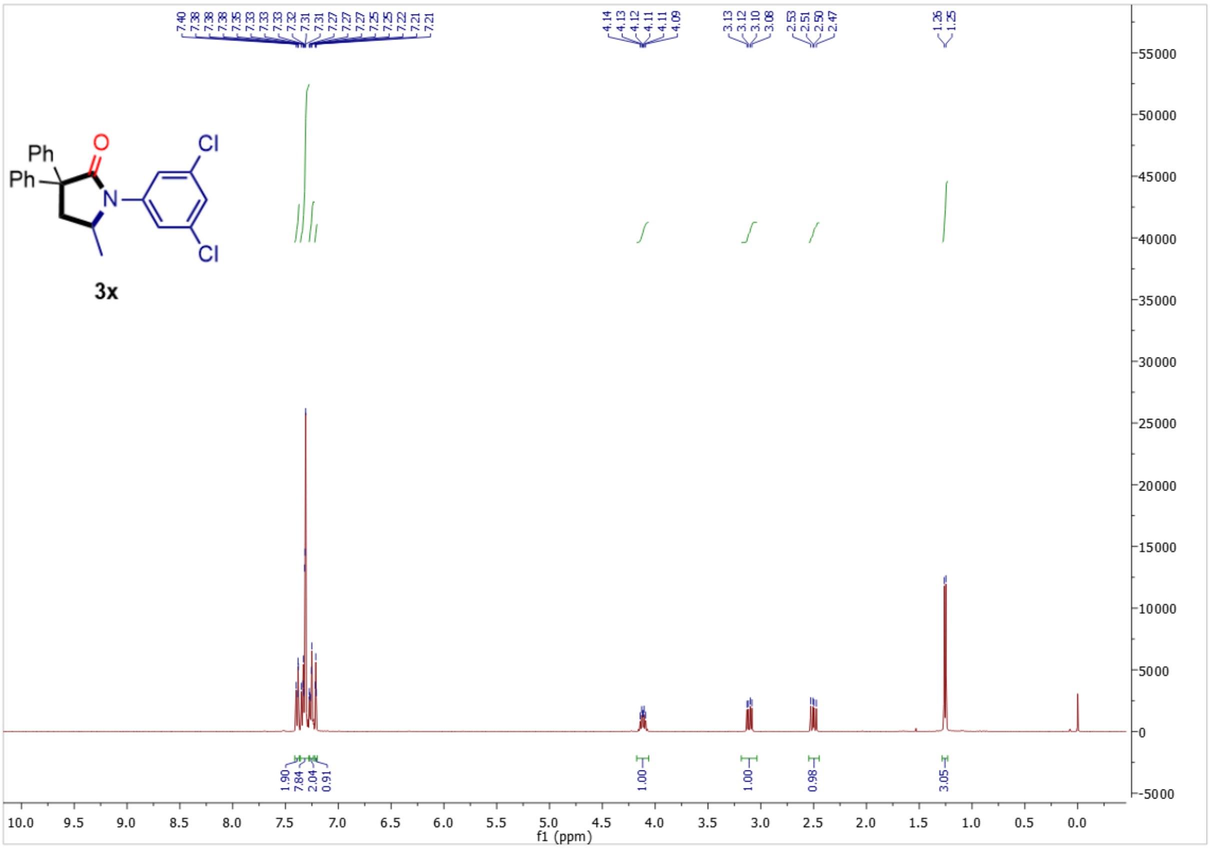
7.40
7.38
7.38
7.38
7.35
7.33
7.33
7.33
7.32
7.31
7.31
7.27
7.27
7.27
7.25
7.25
7.22
7.21
7.21
4.14
4.13
4.12
4.11
4.11
4.09
3.13
3.12
3.10
3.08
2.53
2.51
2.50
2.47
1.26
1.25
Ph
Ph
O
N
Cl
Cl
3x
1.90
7.84
2.04
0.91
1.00
1.00
0.98
3.05
f1 (ppm)

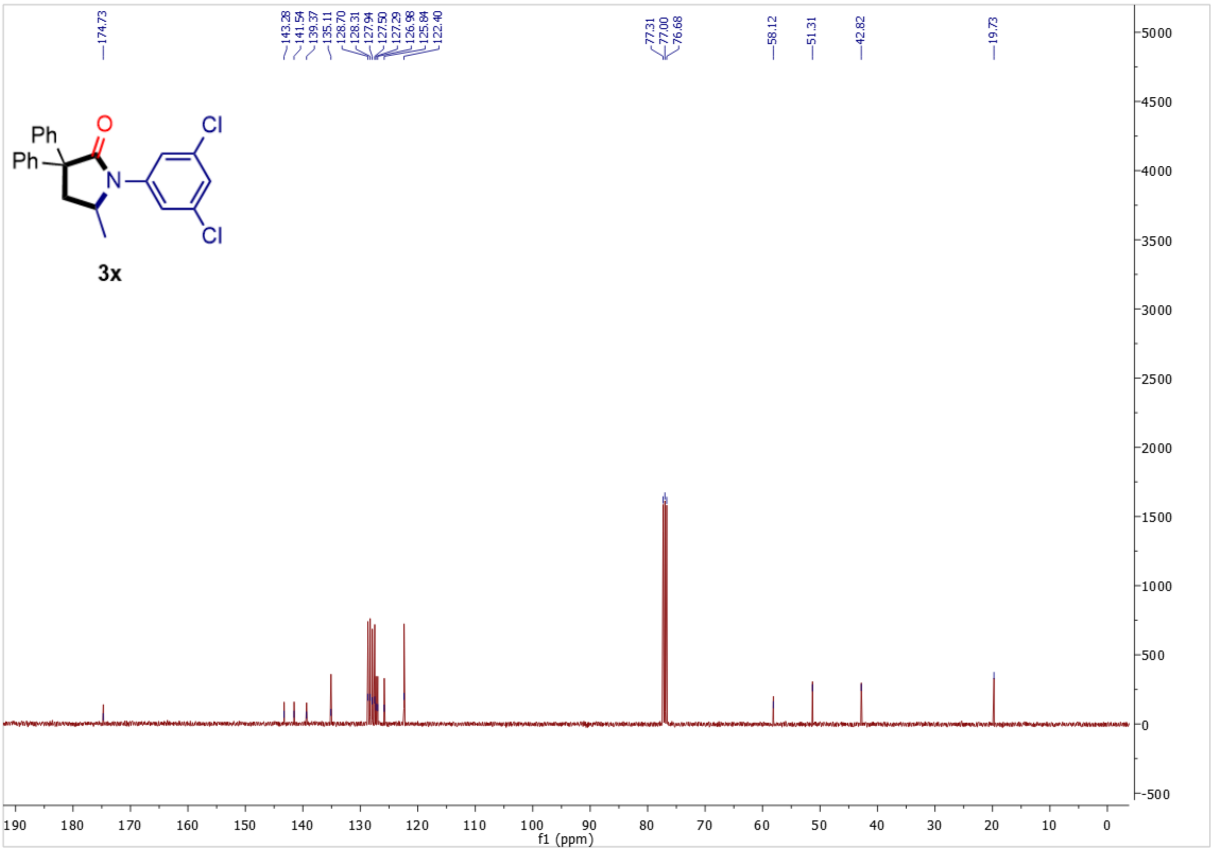
174.73
143.28
141.54
139.37
135.11
128.70
128.31
127.94
127.50
127.29
126.98
125.84
122.40
77.31
77.00
76.68
58.12
51.31
42.82
19.73
Ph
Ph
O
N
Cl
Cl
3x
f1 (ppm)

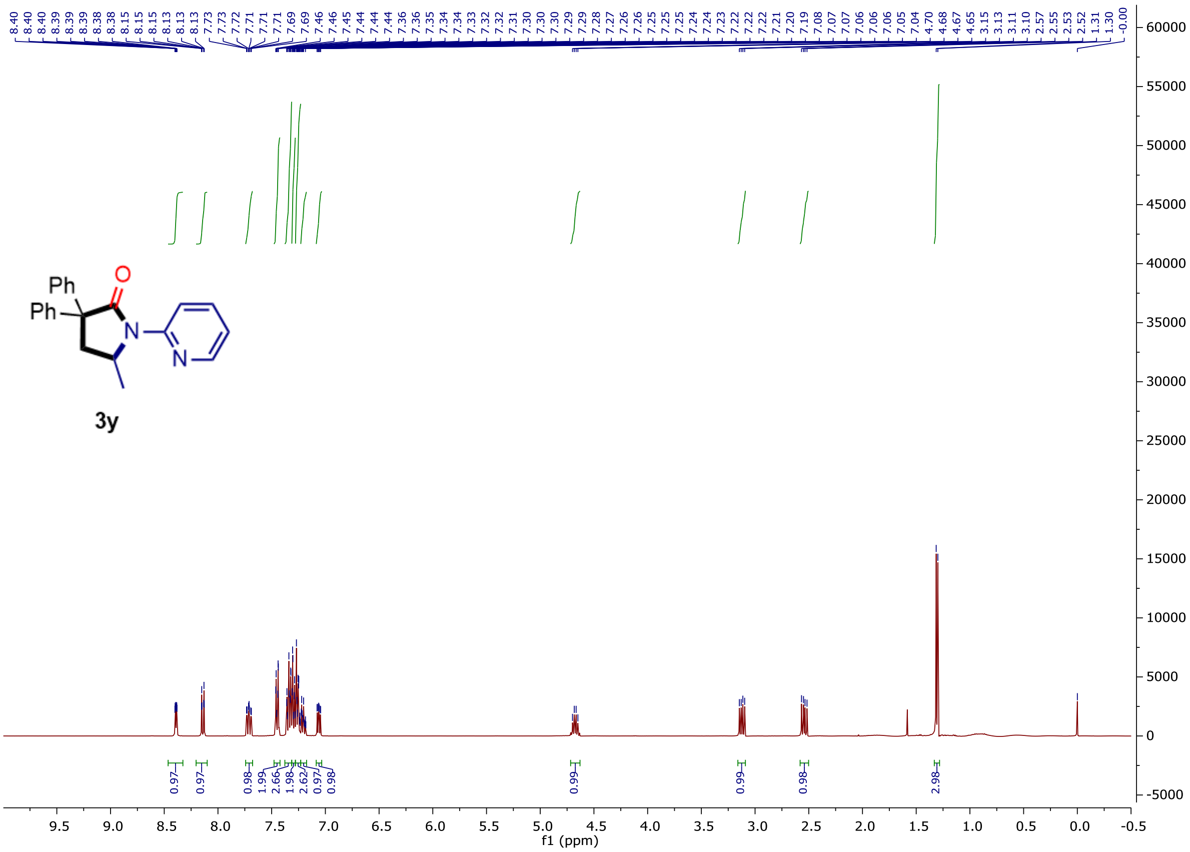
8.40 8.40 8.40 8.39 8.39 8.38 8.38 8.15 8.15 8.15 8.13 8.13 8.13 7.73 7.73 7.72 7.71 7.71 7.71 7.69 7.69 7.46 7.45 7.44 7.44 7.44 7.36 7.35 7.34 7.34 7.33 7.32 7.32 7.31 7.30 7.30 7.30 7.29 7.29 7.28 7.27 7.26 7.26 7.25 7.25 7.25 7.24 7.23 7.22 7.22 7.22 7.21 7.20 7.19 7.08 7.07 7.06 7.06 7.06 7.05 7.04 4.70 4.68 4.67 4.65 3.15 3.13 3.11 3.10 2.57 2.55 2.53 2.52 1.31 1.30 -0.00
0.97 0.97 0.98 1.99 2.66 1.98 2.62 0.97 0.98 0.99 0.99 0.98 2.98
Ph
Ph
O
N
N
3y
f1 (ppm)

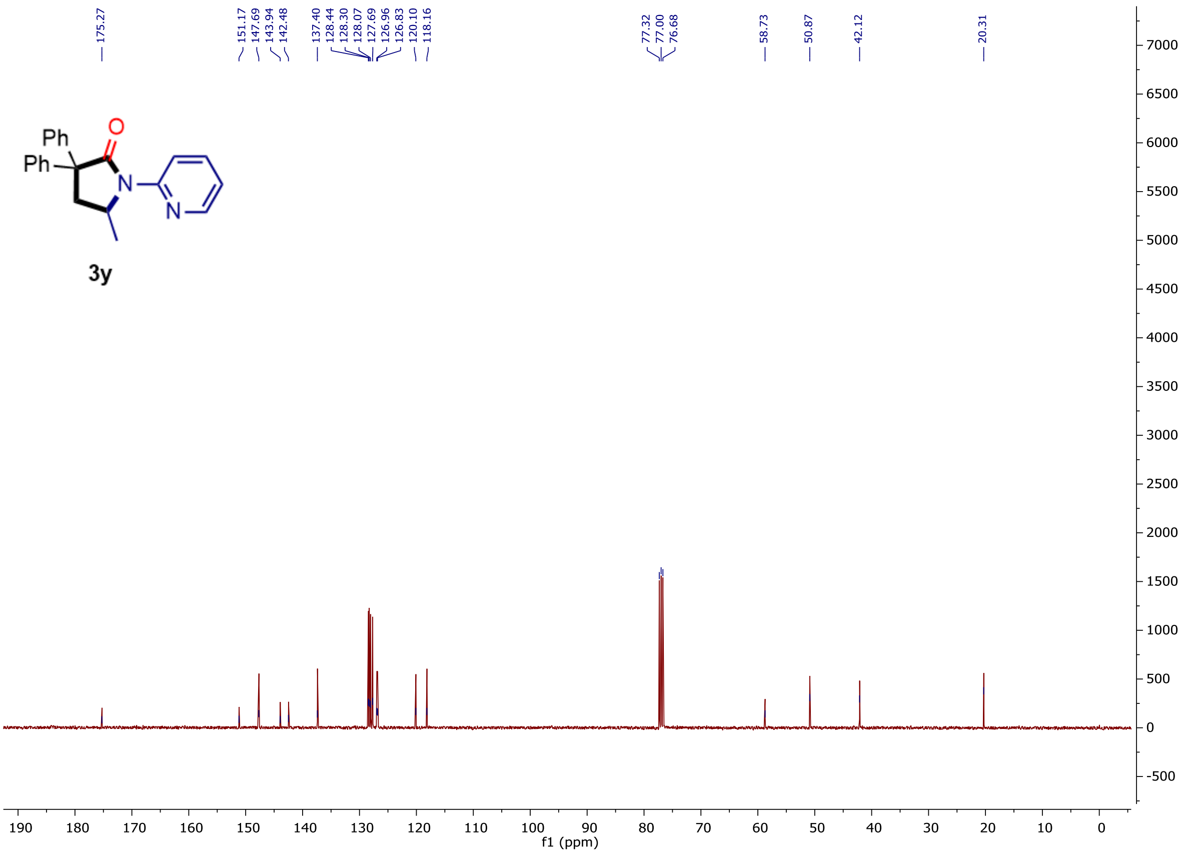
175.27
151.17 147.69 143.94 142.48
137.40 128.44 128.30 128.07 127.69 126.96 126.83 120.10 118.16
77.32 77.00 76.68
58.73
50.87
42.12
20.31
Ph
Ph
O
N
N
3y
f1 (ppm)

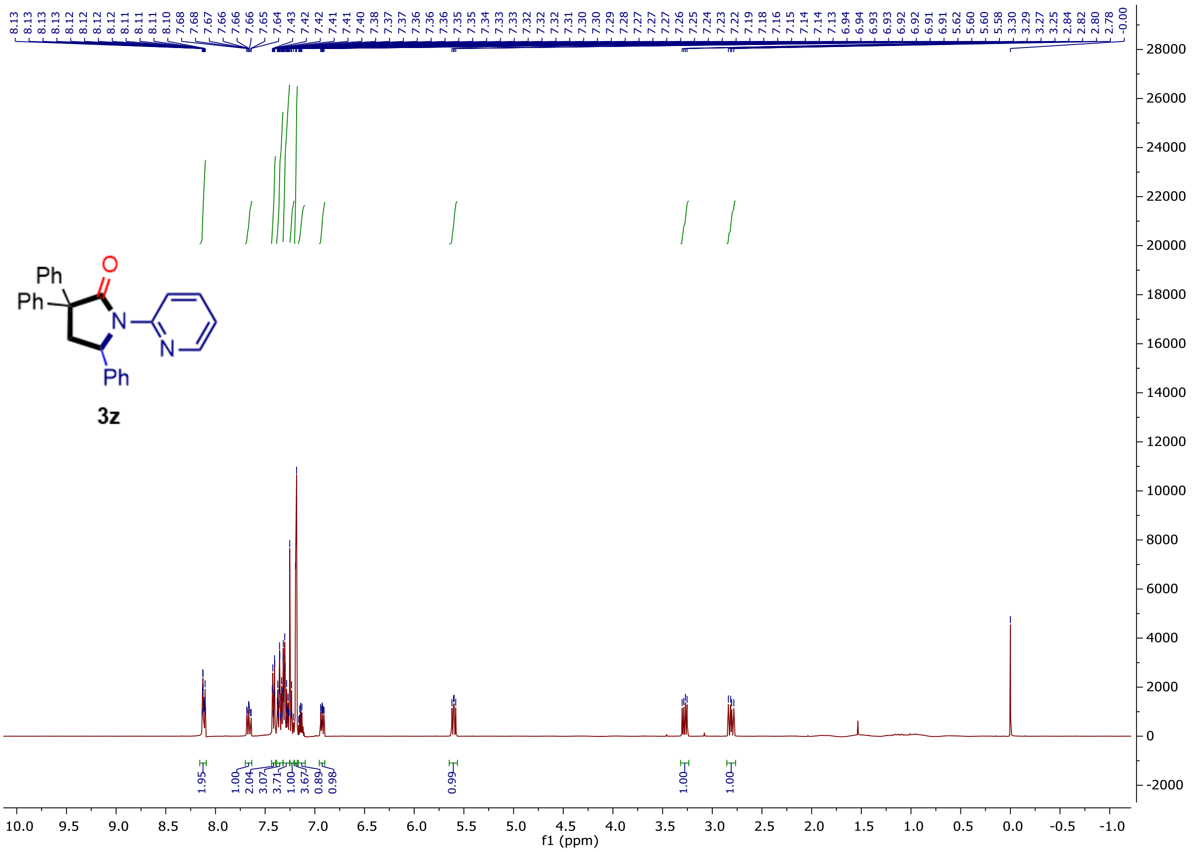
8.13 8.13 8.13 8.13 8.12 8.12 8.12 8.12 8.11 8.11 8.11 8.10 7.68 7.67 7.66 7.66 7.66 7.65 7.64 7.43 7.42 7.42 7.41 7.41 7.40 7.38 7.37 7.37 7.36 7.36 7.36 7.35 7.35 7.34 7.33 7.33 7.32 7.32 7.32 7.31 7.30 7.30 7.29 7.28 7.27 7.27 7.27 7.26 7.25 7.24 7.23 7.22 7.19 7.18 7.16 7.15 7.14 7.14 7.13 6.94 6.94 6.93 6.93 6.92 6.92 6.91 6.91 5.62 5.60 5.60 5.58 3.30 3.29 3.27 3.25 2.84 2.82 2.80 2.78 -0.00
Ph
Ph
O
N
N
Ph
3z
1.95 1.00 2.04 3.07 3.71 1.00 3.67 0.89 0.98 0.99 1.00 1.00
f1 (ppm)

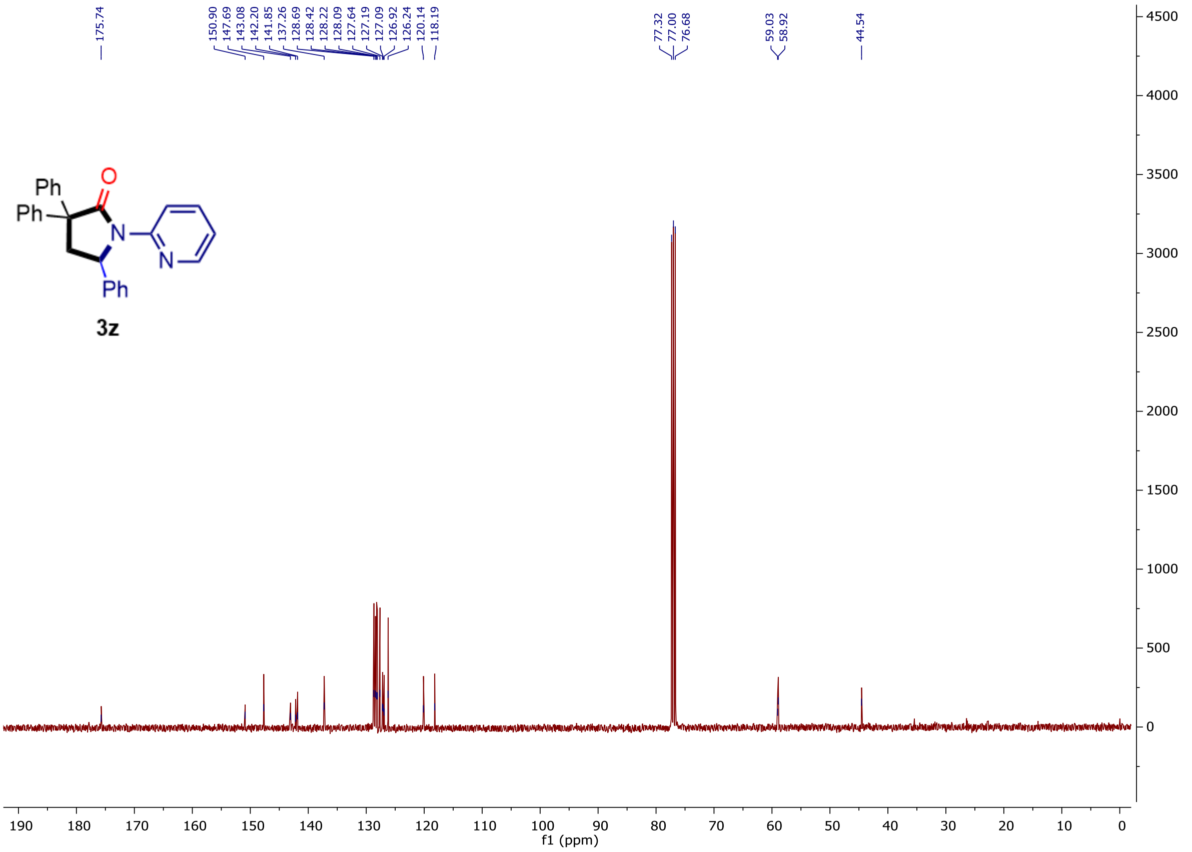
175.74
150.90 147.69 143.08 142.20 141.85 137.26 128.69 128.42 128.22 128.09 127.64 127.19 127.09 126.92 126.24 120.14 118.19
77.32 77.00 76.68
59.03 58.92
44.54
Ph
Ph
O
N
N
Ph
3z
f1 (ppm)

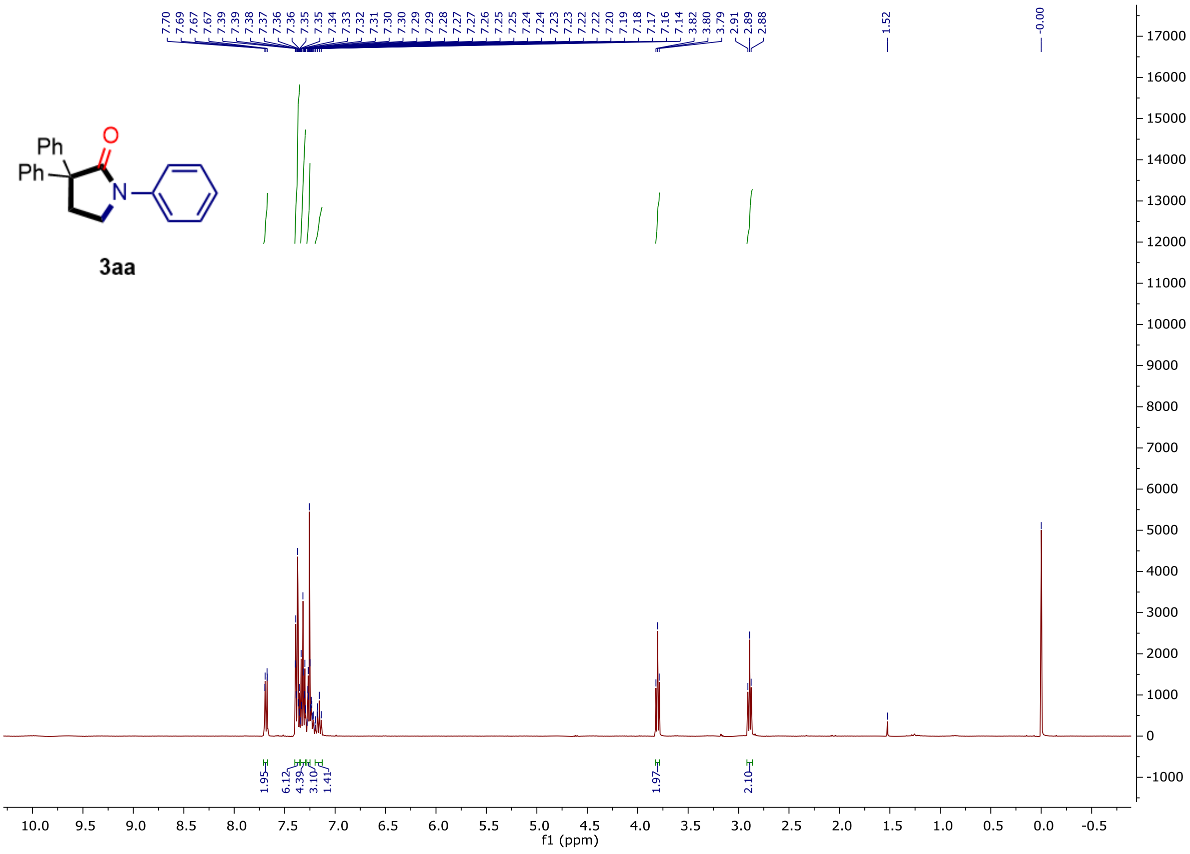

7.70 7.69 7.67 7.67 7.39 7.39 7.38 7.37 7.36 7.35 7.35 7.34 7.33 7.32 7.31 7.30 7.30 7.29 7.29 7.28 7.27 7.27 7.26 7.25 7.25 7.24 7.24 7.23 7.23 7.22 7.22 7.20 7.19 7.18 7.17 7.16 7.14 3.82 3.80 3.79 2.91 2.89 2.88
1.52
-0.00
Ph
Ph
O
N
3aa
1.95
6.12
4.39
3.10
1.41
1.97
2.10
f1 (ppm)

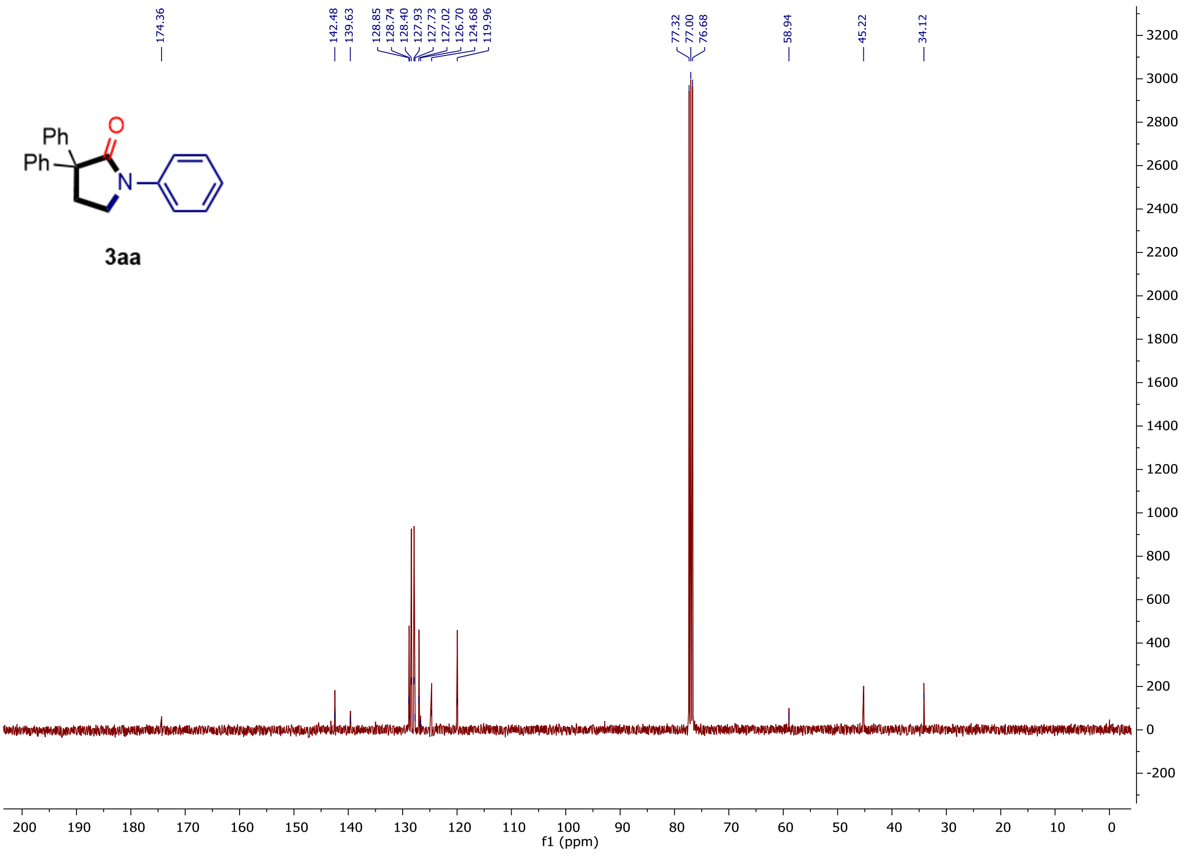

174.36
142.48 139.63
128.85 128.74 128.40 127.93 127.73 127.02 126.70 124.68 119.96
77.32 77.00 76.68
58.94
45.22
34.12
Ph
Ph
O
N
3aa
f1 (ppm)

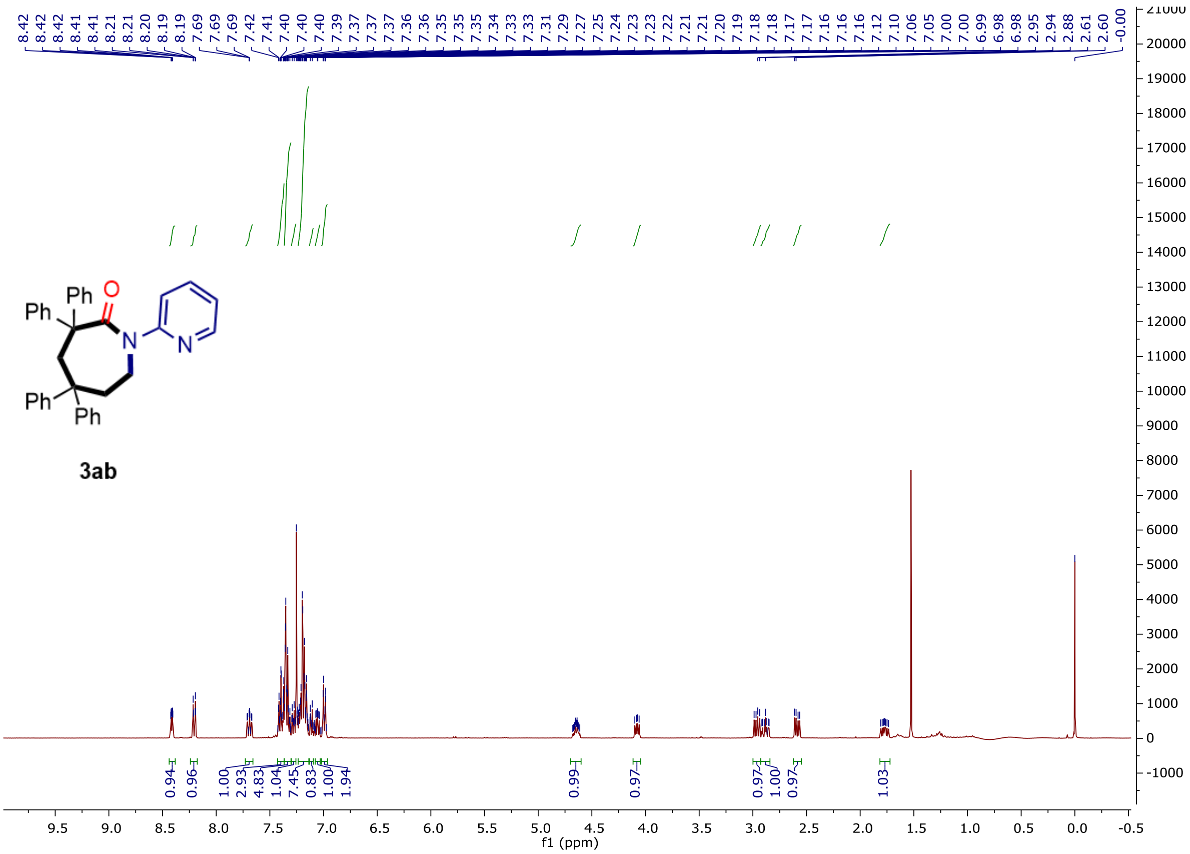

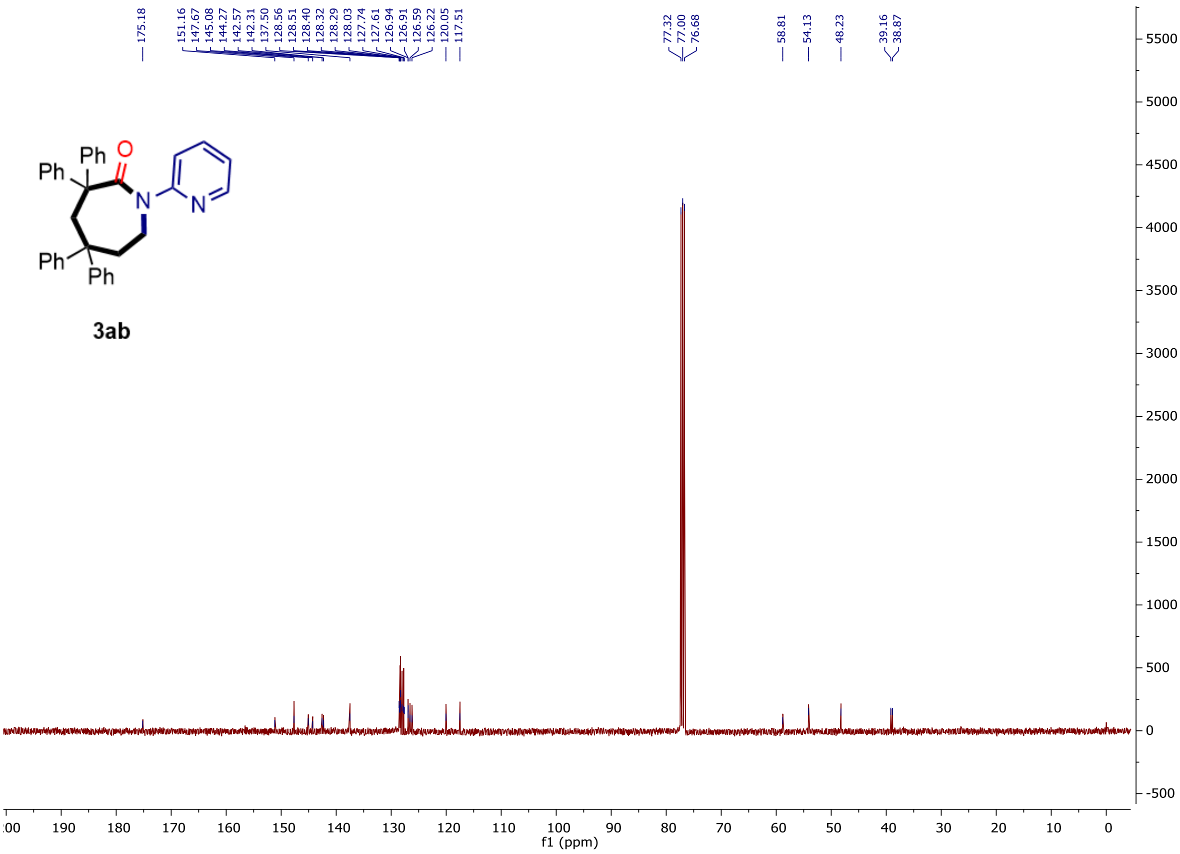

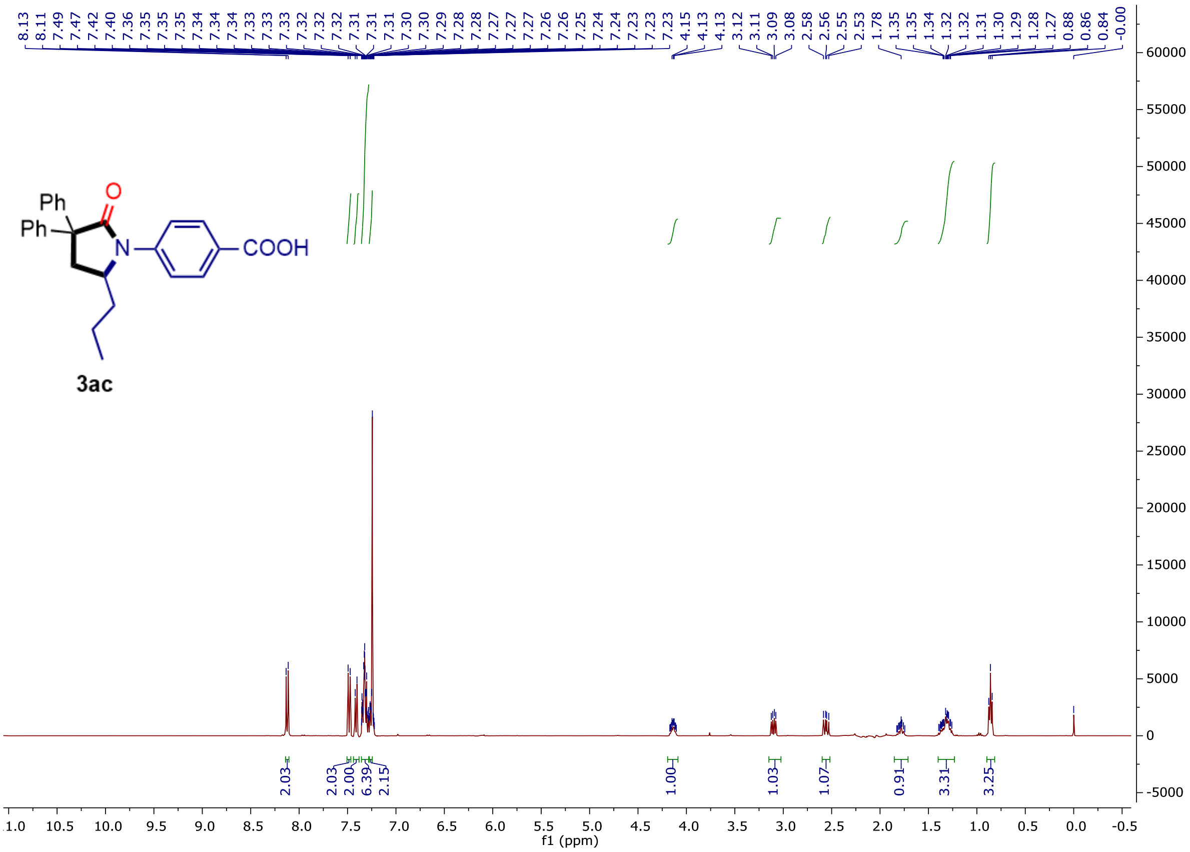

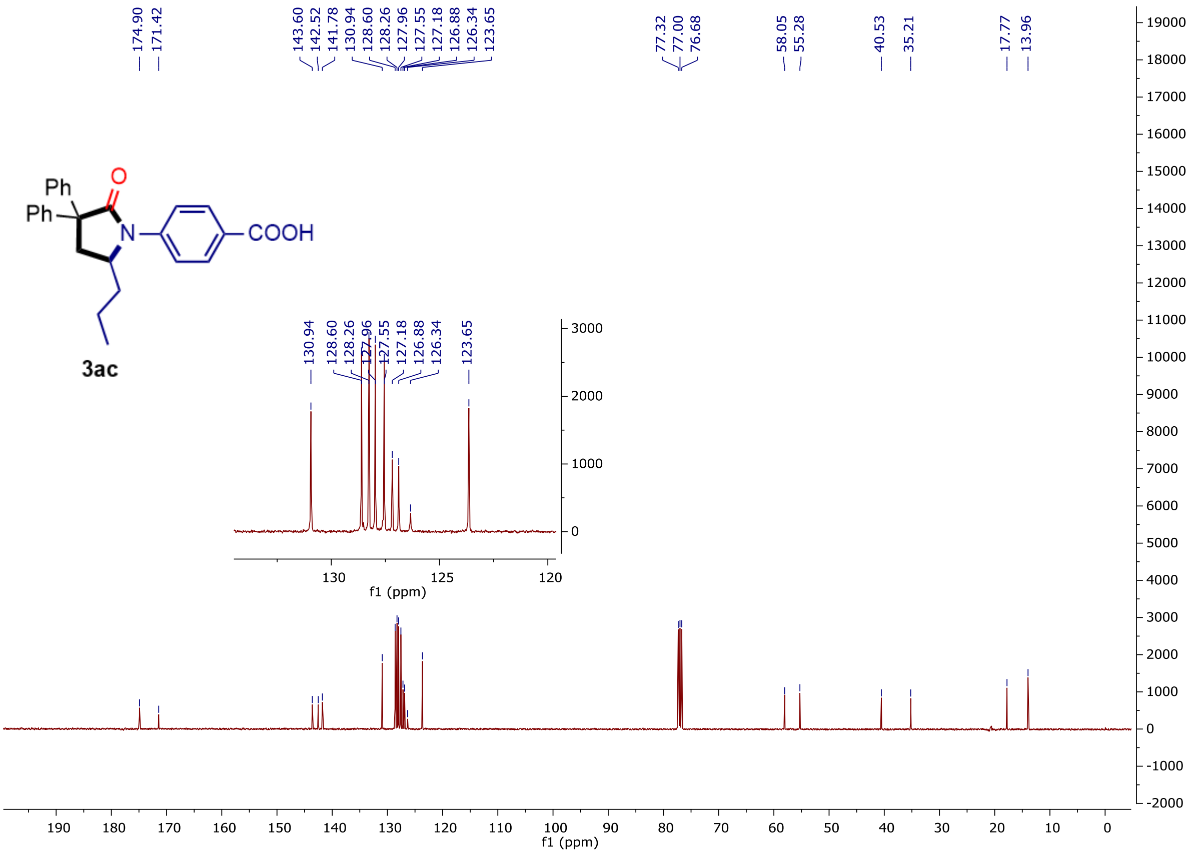

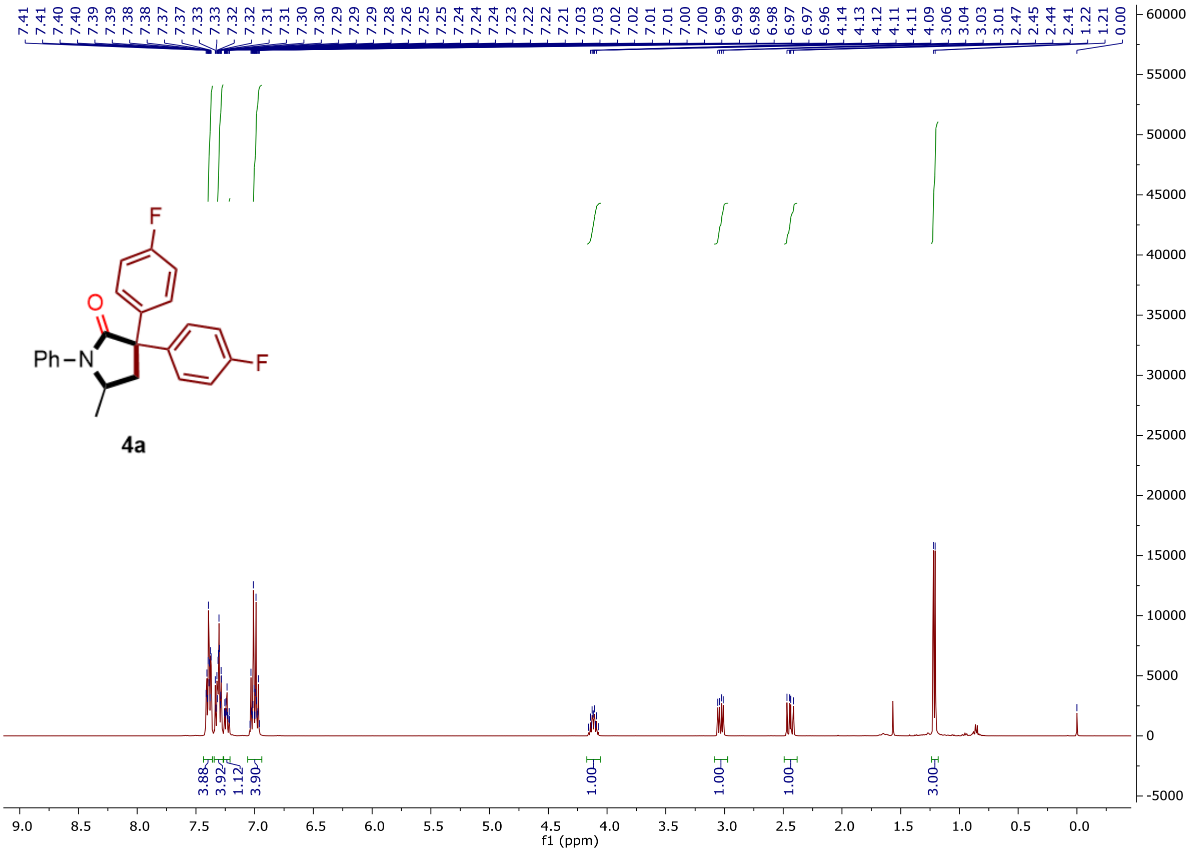

7.41
7.41
7.40
7.39
7.39
7.38
7.38
7.37
7.37
7.33
7.33
7.32
7.32
7.31
7.31
7.30
7.30
7.29
7.29
7.29
7.28
7.26
7.25
7.25
7.24
7.24
7.24
7.23
7.22
7.22
7.21
7.03
7.03
7.02
7.02
7.01
7.01
7.00
7.00
6.99
6.99
6.98
6.98
6.97
6.97
6.96
4.14
4.13
4.12
4.11
4.11
4.09
3.06
3.04
3.03
3.01
2.47
2.45
2.44
2.41
1.22
1.21
0.00
F
O
Ph–N
F
4a
3.88
3.92
1.12
3.90
1.00
1.00
1.00
3.00
f1 (ppm)

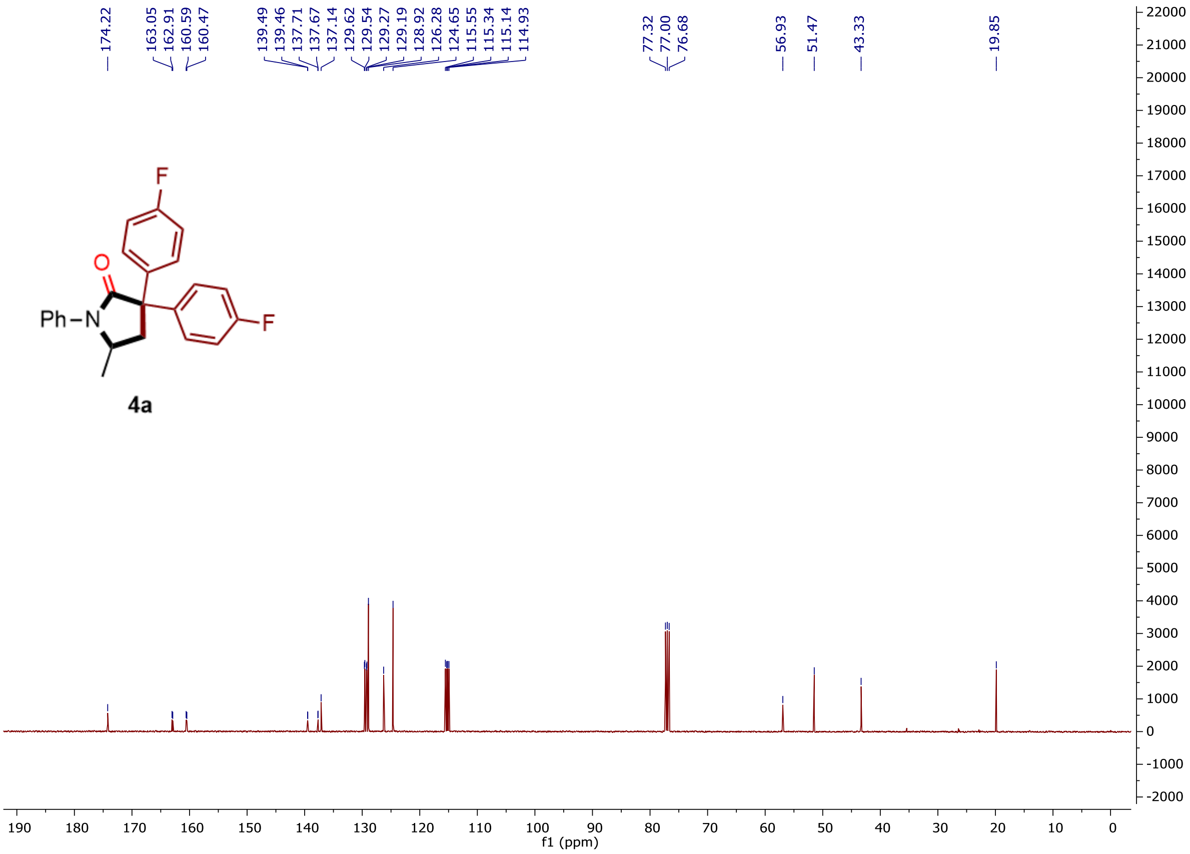

174.22
163.05
162.91
160.59
160.47
139.49
139.46
137.71
137.67
137.14
129.62
129.54
129.27
129.19
128.92
126.28
124.65
115.55
115.34
115.14
114.93
77.32
77.00
76.68
56.93
51.47
43.33
19.85
F
O
Ph–N
F
4a
f1 (ppm)

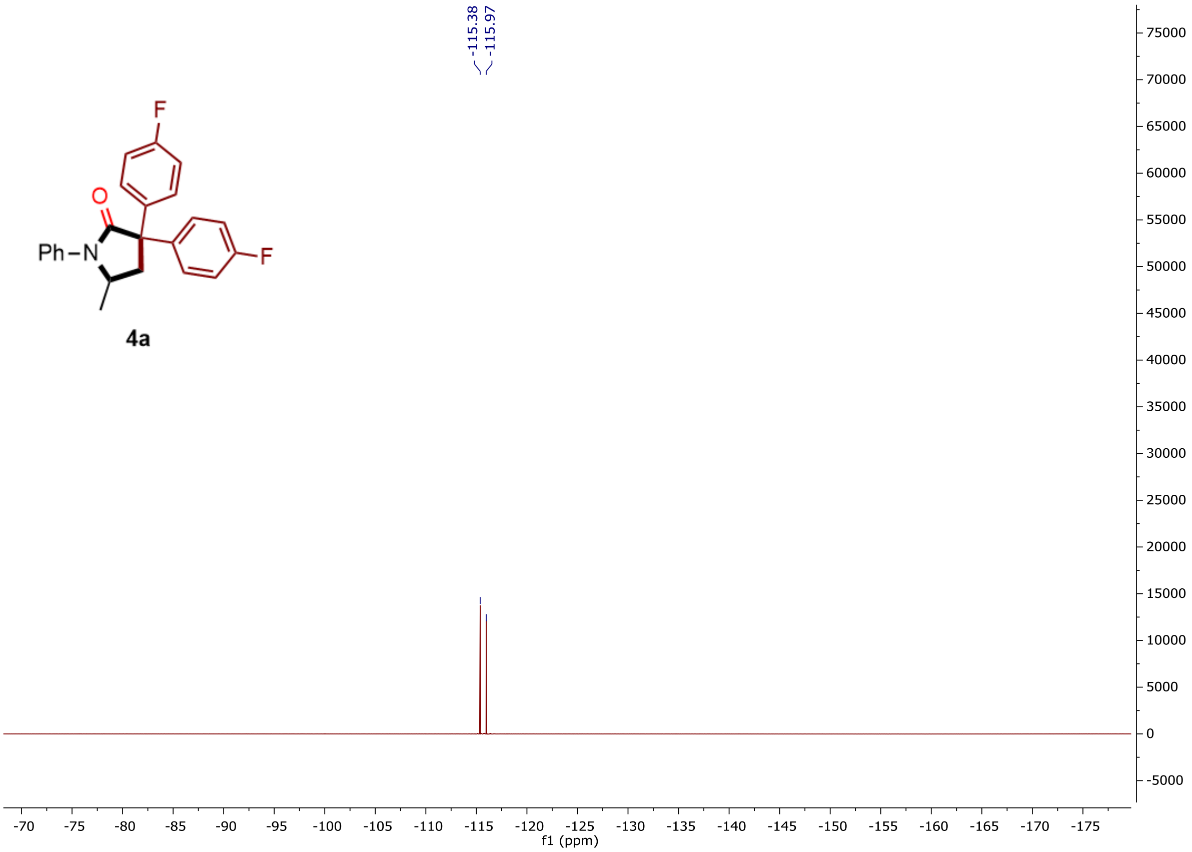
-115.38
-115.97
F
O
Ph
N
4a
f1 (ppm)

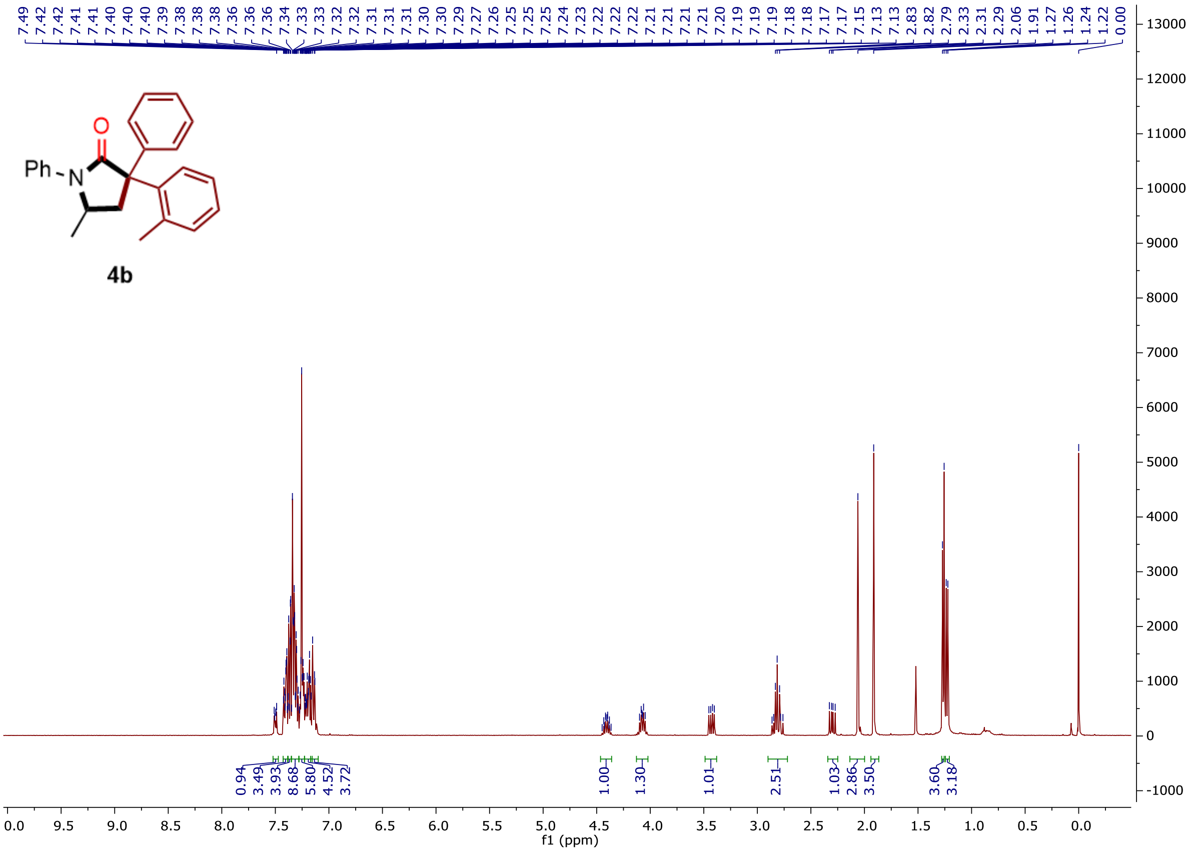
7.49 7.42 7.42 7.41 7.41 7.40 7.40 7.39 7.38 7.38 7.36 7.36 7.34 7.33 7.33 7.32 7.32 7.31 7.31 7.30 7.30 7.29 7.27 7.26 7.25 7.25 7.24 7.23 7.22 7.22 7.22 7.21 7.21 7.21 7.21 7.20 7.19 7.19 7.18 7.17 7.15 7.13 7.13 2.83 2.82 2.79 2.33 2.31 2.29 2.06 1.91 1.27 1.26 1.24 1.22 0.00
O
Ph
N
4b
0.94 3.49 3.93 8.68 5.80 4.52 3.72 1.00 1.30 1.01 2.51 1.03 2.86 3.50 3.60 3.18
f1 (ppm)

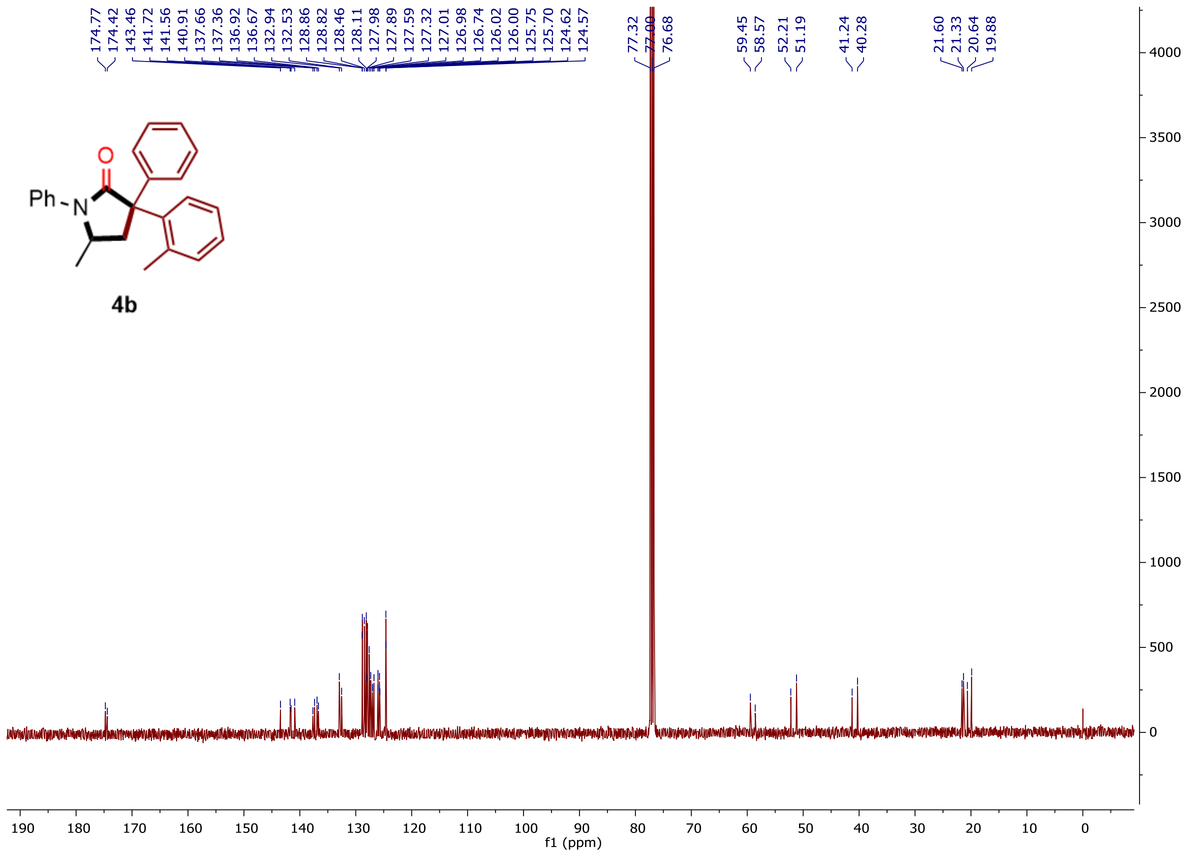
174.77
174.42
143.46
141.72
141.56
140.91
137.66
137.36
136.92
136.67
132.94
132.53
128.86
128.82
128.46
128.11
127.98
127.89
127.59
127.32
127.01
126.98
126.74
126.02
126.00
125.75
125.70
124.62
124.57
77.32
77.00
76.68
59.45
58.57
52.21
51.19
41.24
40.28
21.60
21.33
20.64
19.88
Ph
N
O
4b
f1 (ppm)

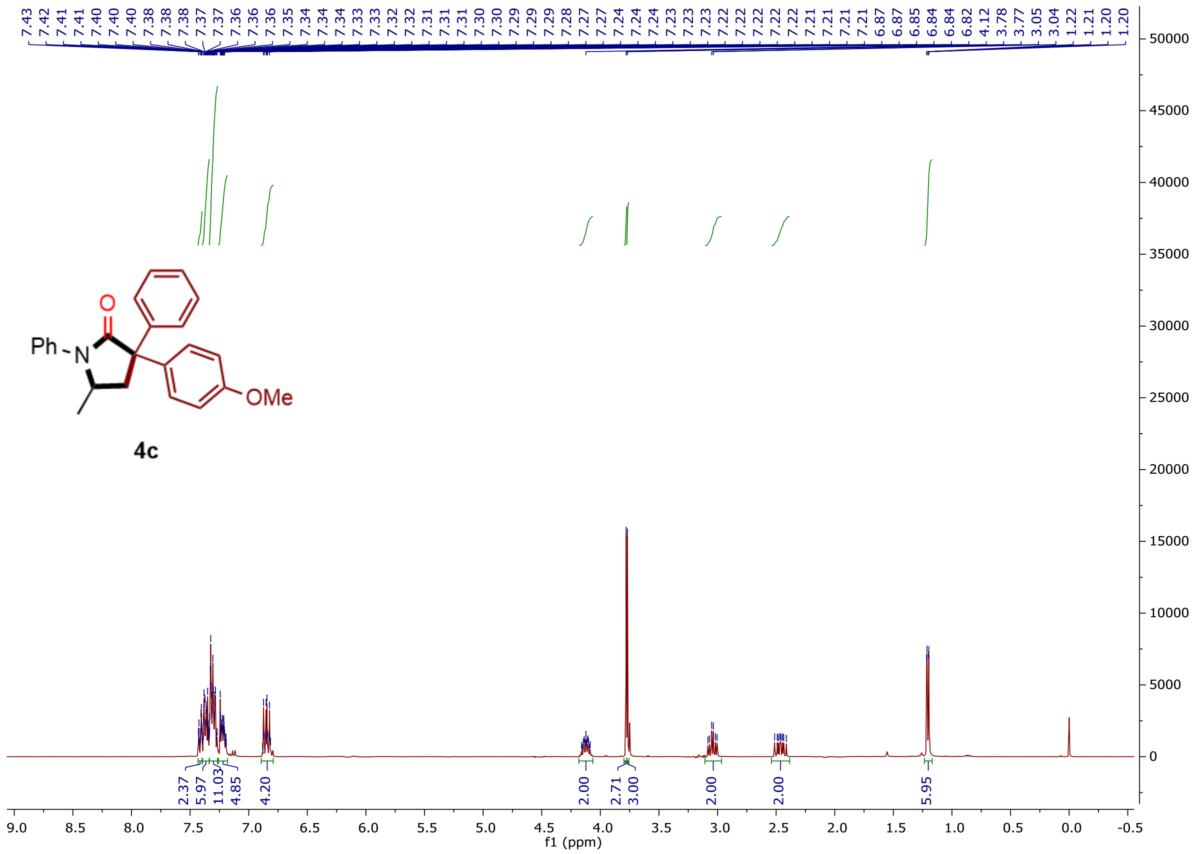
7.43
7.42
7.41
7.41
7.40
7.40
7.40
7.38
7.38
7.38
7.37
7.37
7.36
7.36
7.35
7.34
7.34
7.34
7.33
7.33
7.32
7.32
7.31
7.31
7.31
7.30
7.30
7.29
7.29
7.28
7.27
7.24
7.24
7.24
7.23
7.23
7.23
7.22
7.22
7.22
7.22
7.22
7.21
7.21
7.21
7.21
6.87
6.87
6.85
6.84
6.82
4.12
3.78
3.77
3.05
3.04
1.22
1.21
1.20
1.20
2.37
5.97
11.03
4.85
4.20
2.00
2.71
3.00
2.00
2.00
5.95
Ph
N
O
OMe
4c
f1 (ppm)

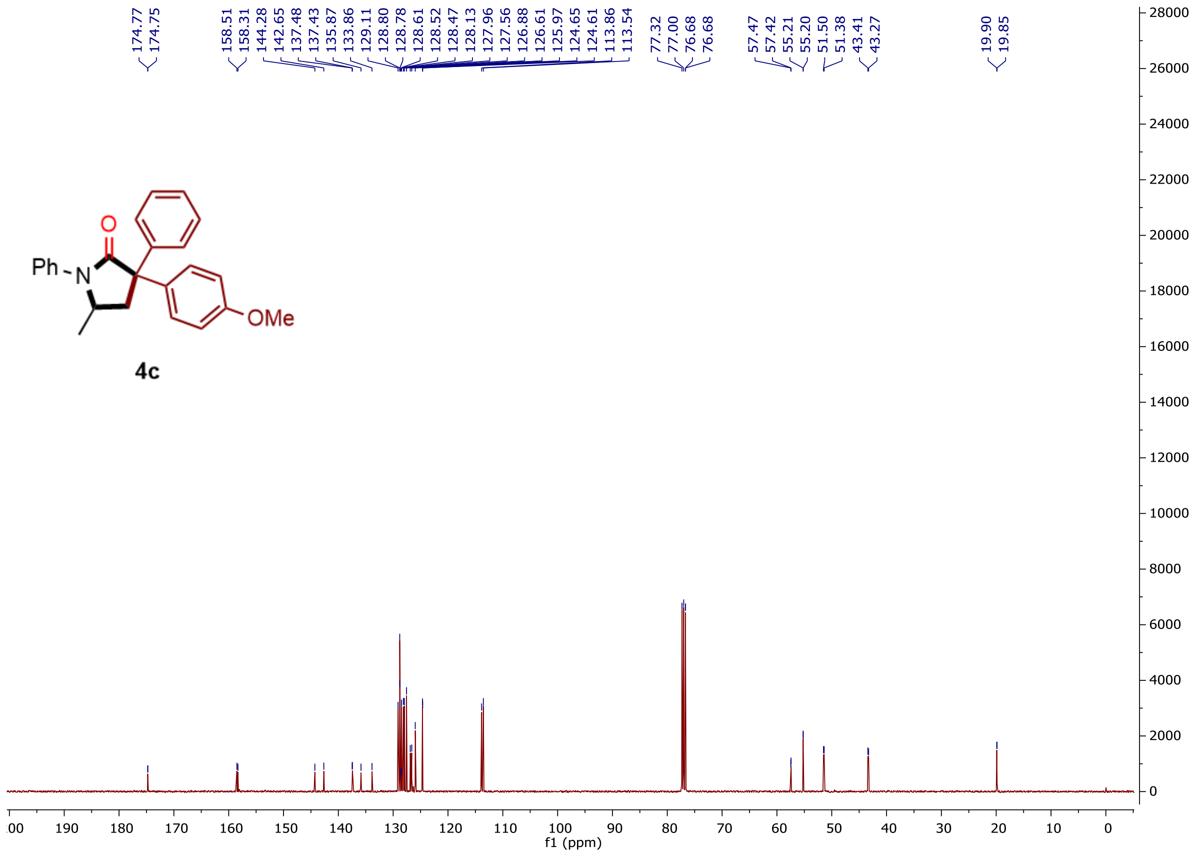
174.77
174.75
158.51
158.31
144.28
142.65
137.48
137.43
135.87
133.86
129.11
128.80
128.78
128.61
128.52
128.47
128.13
127.96
127.56
126.88
126.61
125.97
124.65
124.61
113.86
113.54
77.32
77.00
76.68
76.68
57.47
57.42
55.21
55.20
51.50
51.38
43.41
43.27
19.90
19.85
Ph
N
O
OMe
4c
f1 (ppm)

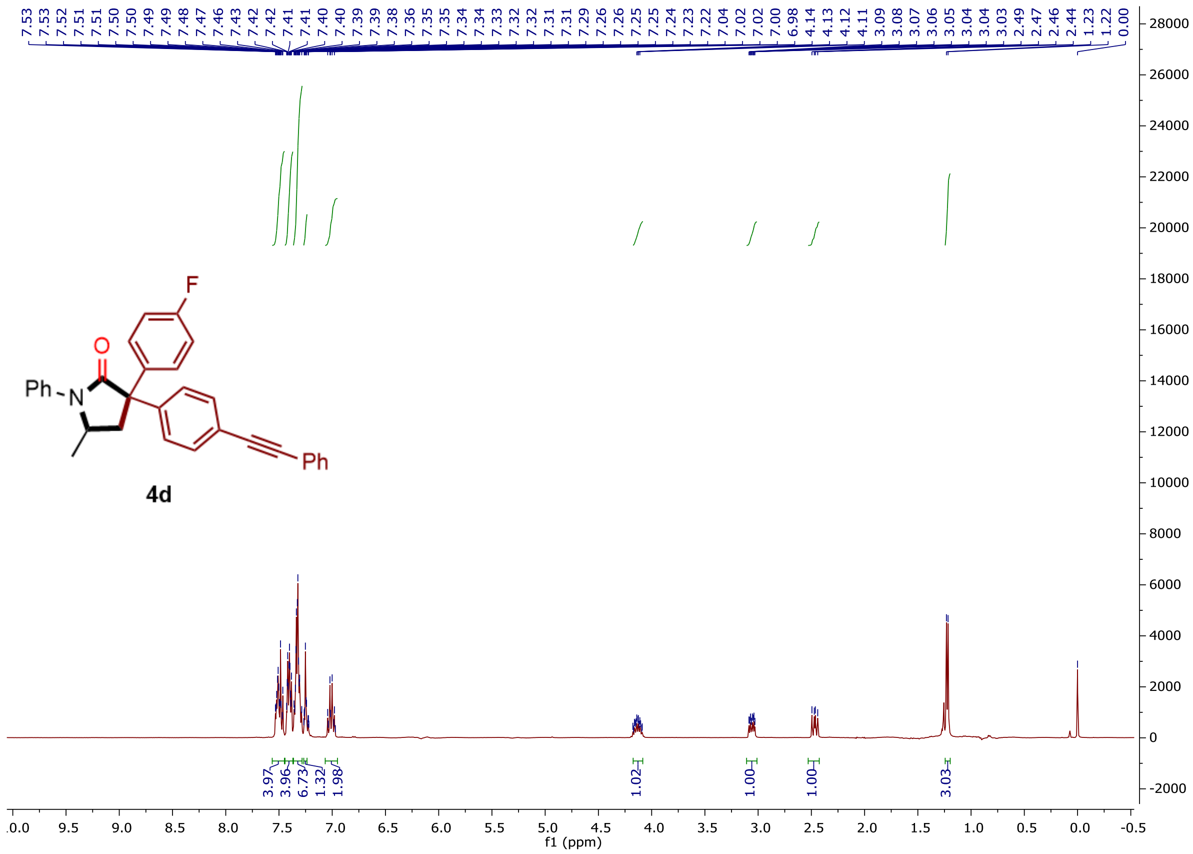
7.53
7.53
7.52
7.51
7.51
7.50
7.49
7.49
7.48
7.47
7.46
7.43
7.42
7.41
7.41
7.40
7.39
7.39
7.38
7.36
7.35
7.35
7.34
7.33
7.32
7.32
7.31
7.31
7.29
7.26
7.26
7.25
7.25
7.24
7.23
7.22
7.04
7.02
7.02
7.00
6.98
4.14
4.13
4.12
4.11
3.09
3.08
3.07
3.06
3.05
3.04
3.04
3.03
2.49
2.47
2.46
2.44
1.23
1.22
0.00
F
O
Ph
N
Ph
4d
3.97
3.96
6.73
1.32
1.98
1.02
1.00
1.00
3.03
f1 (ppm)

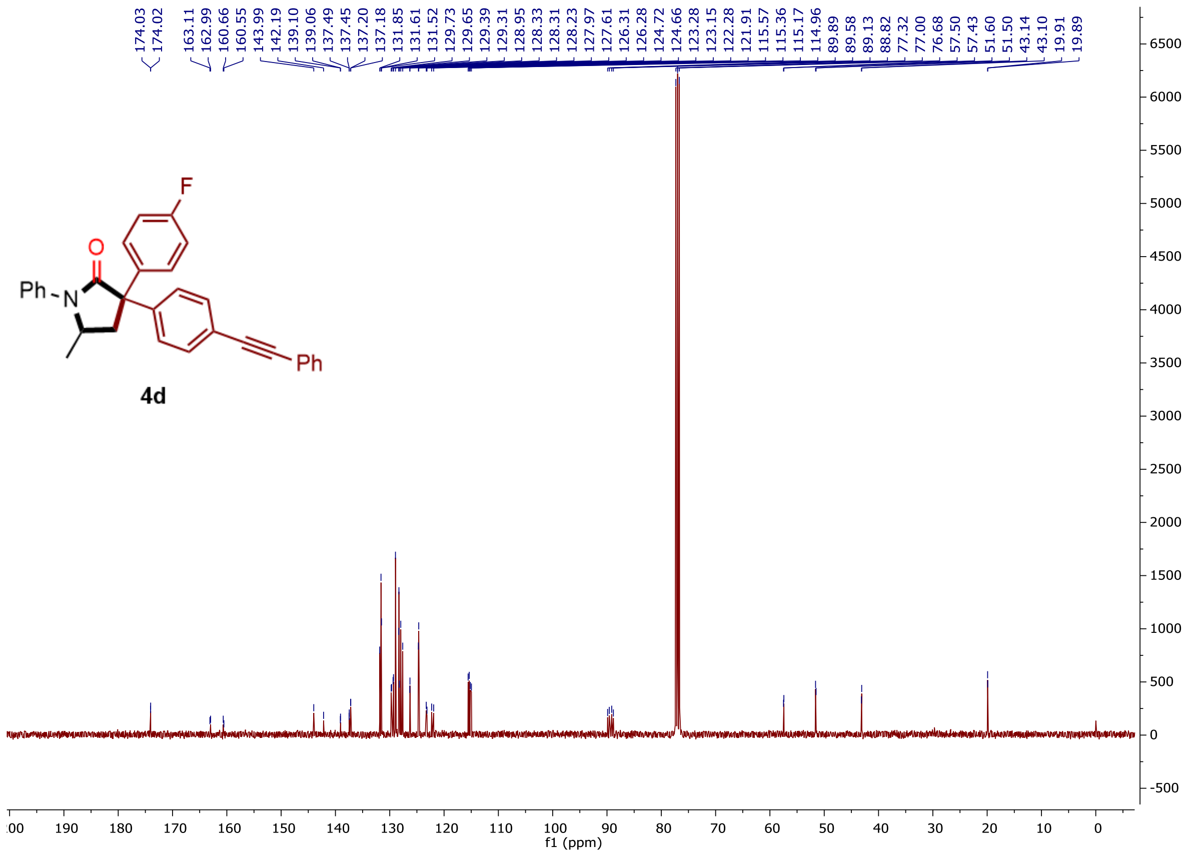
174.03
174.02
163.11
162.99
160.66
160.55
143.99
142.19
139.10
139.06
137.49
137.45
137.20
137.18
131.85
131.61
131.52
129.73
129.65
129.39
129.31
128.95
128.33
128.31
128.23
127.97
127.61
126.31
126.28
124.72
124.66
123.28
123.15
122.28
121.91
115.57
115.36
115.17
114.96
89.89
89.58
89.13
88.82
77.32
77.00
76.68
57.50
57.43
51.60
51.50
43.14
43.10
19.91
19.89
F
O
Ph
N
Ph
4d
f1 (ppm)

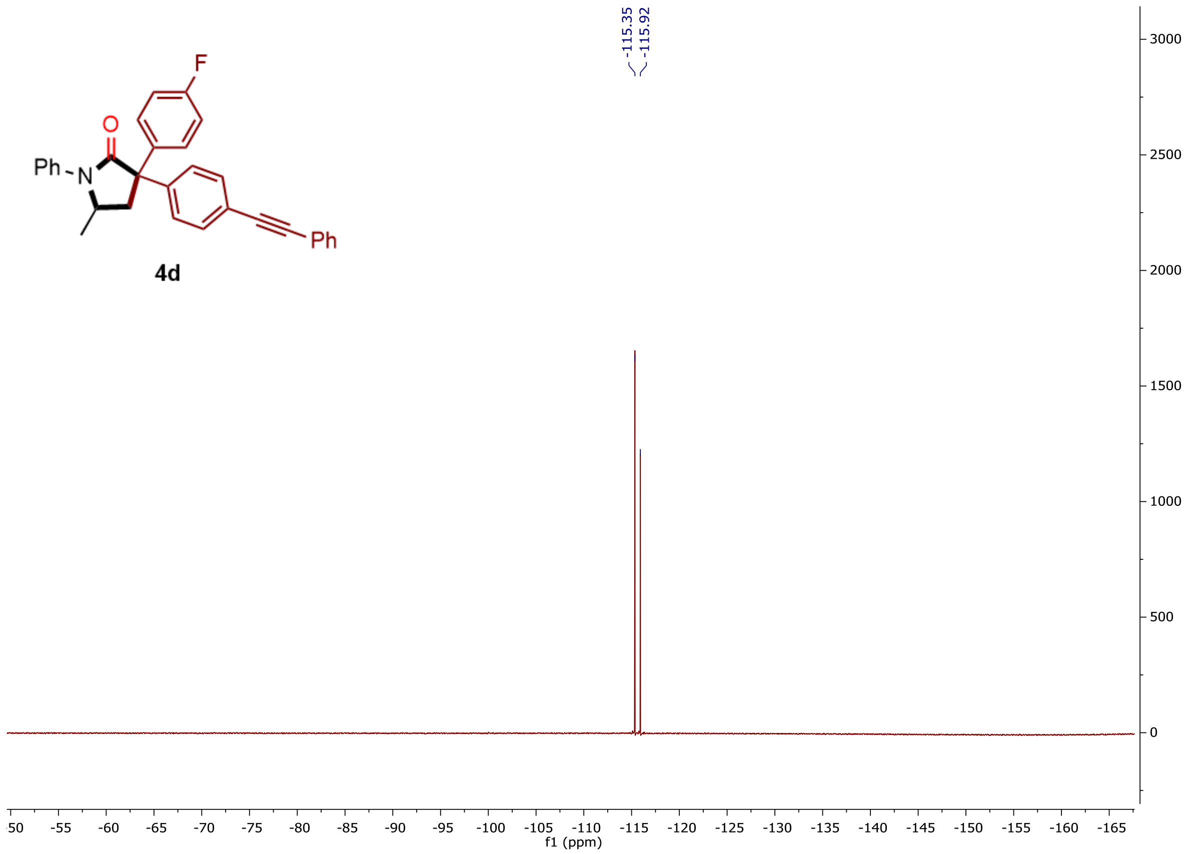
-115.35
-115.92
F
O
Ph
N
Ph
4d
f1 (ppm)

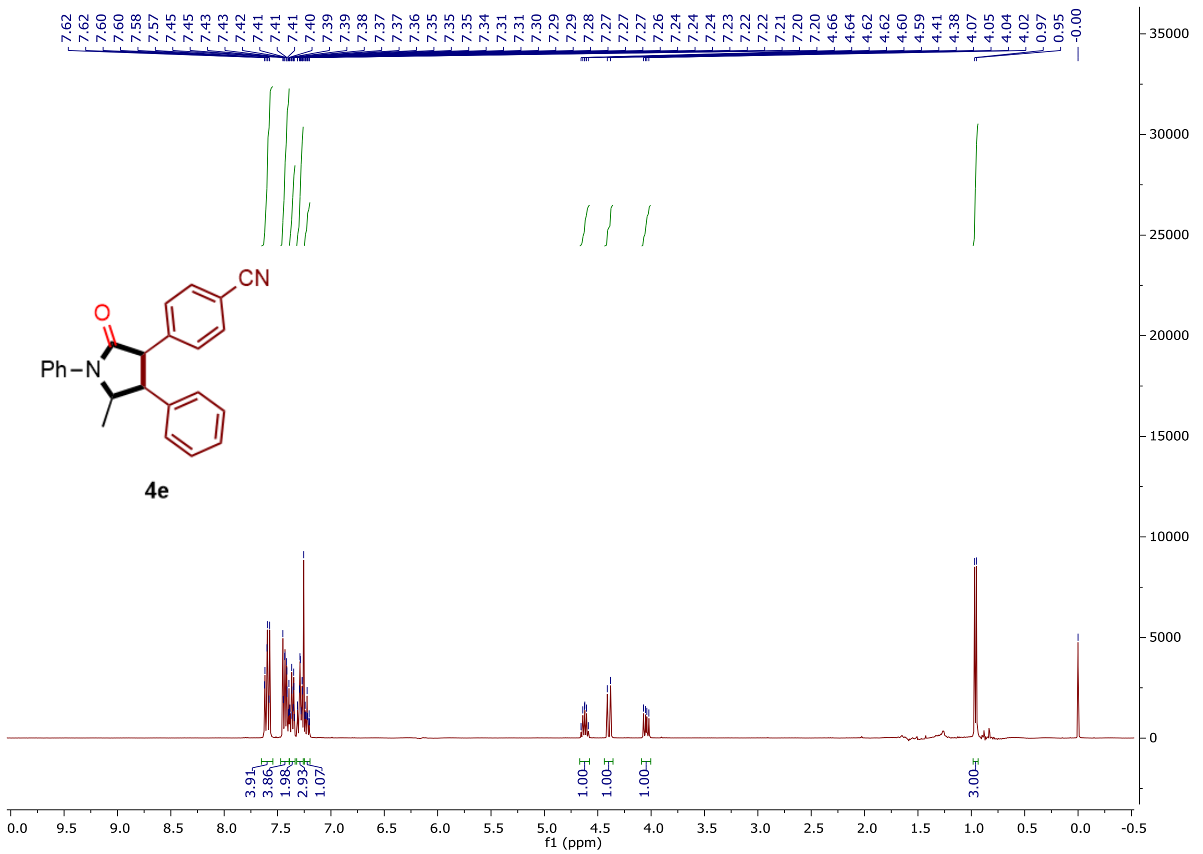

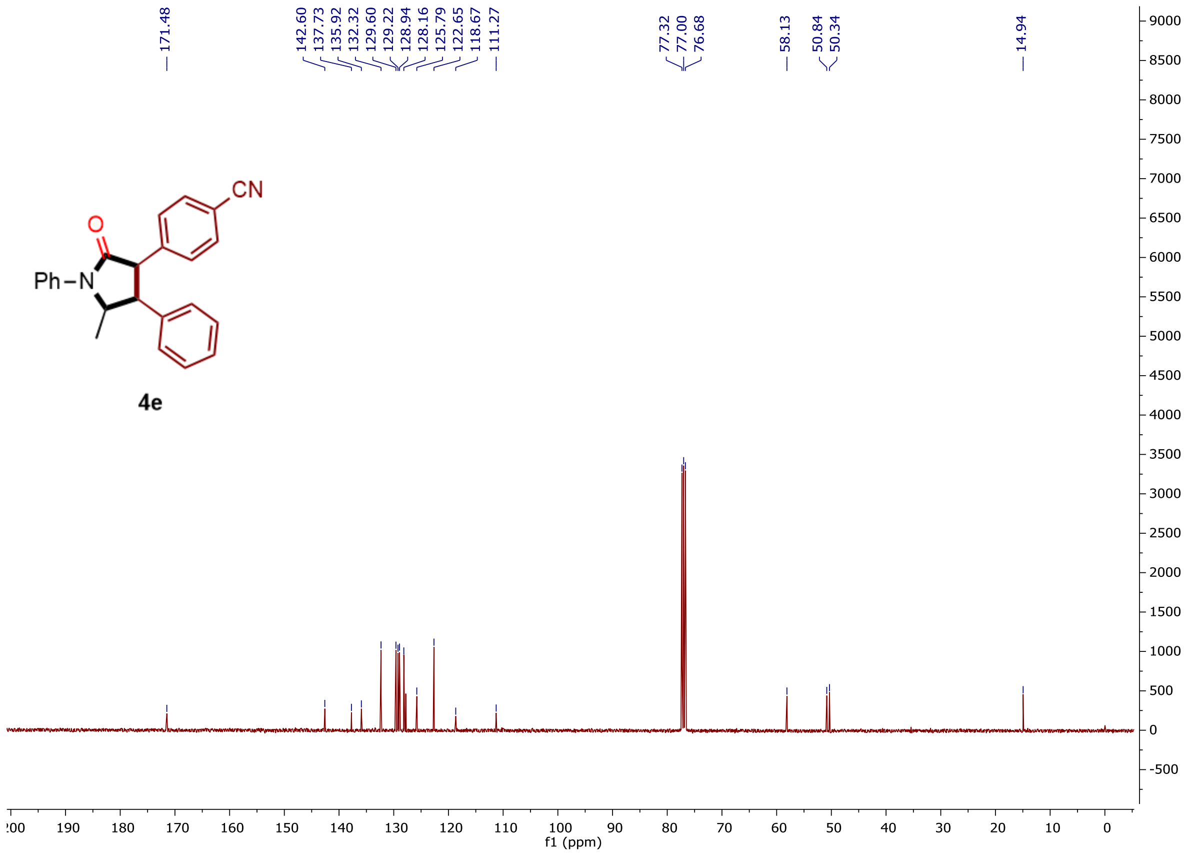

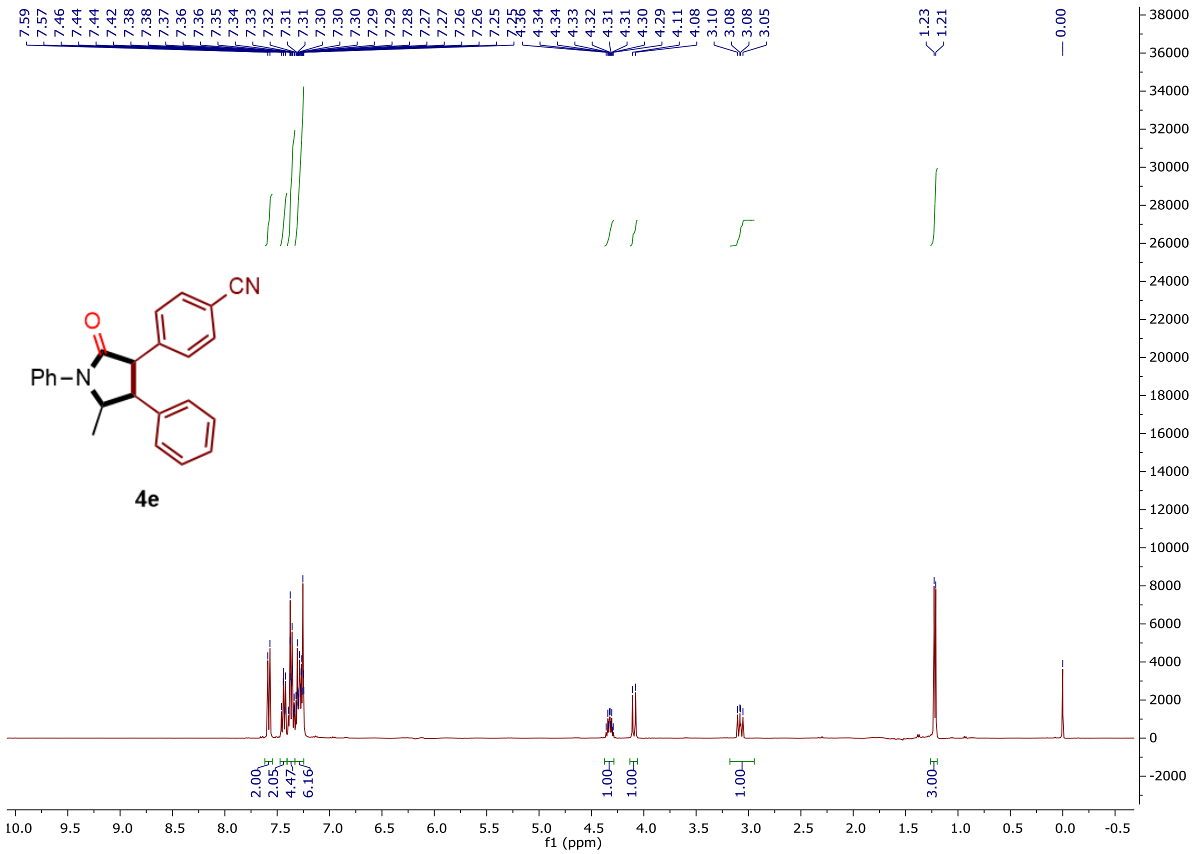
7.59 7.57 7.46 7.44 7.44 7.42 7.38 7.38 7.37 7.36 7.36 7.35 7.34 7.33 7.32 7.31 7.31 7.30 7.30 7.30 7.29 7.29 7.28 7.27 7.27 7.26 7.26 7.25 7.25 4.36 4.34 4.34 4.33 4.32 4.31 4.31 4.30 4.29 4.11 4.08 3.10 3.08 3.08 3.05
1.23 1.21
0.00
CN
O
Ph–N
4e
2.00 2.05 4.47 6.16
1.00 1.00
1.00
3.00
f1 (ppm)

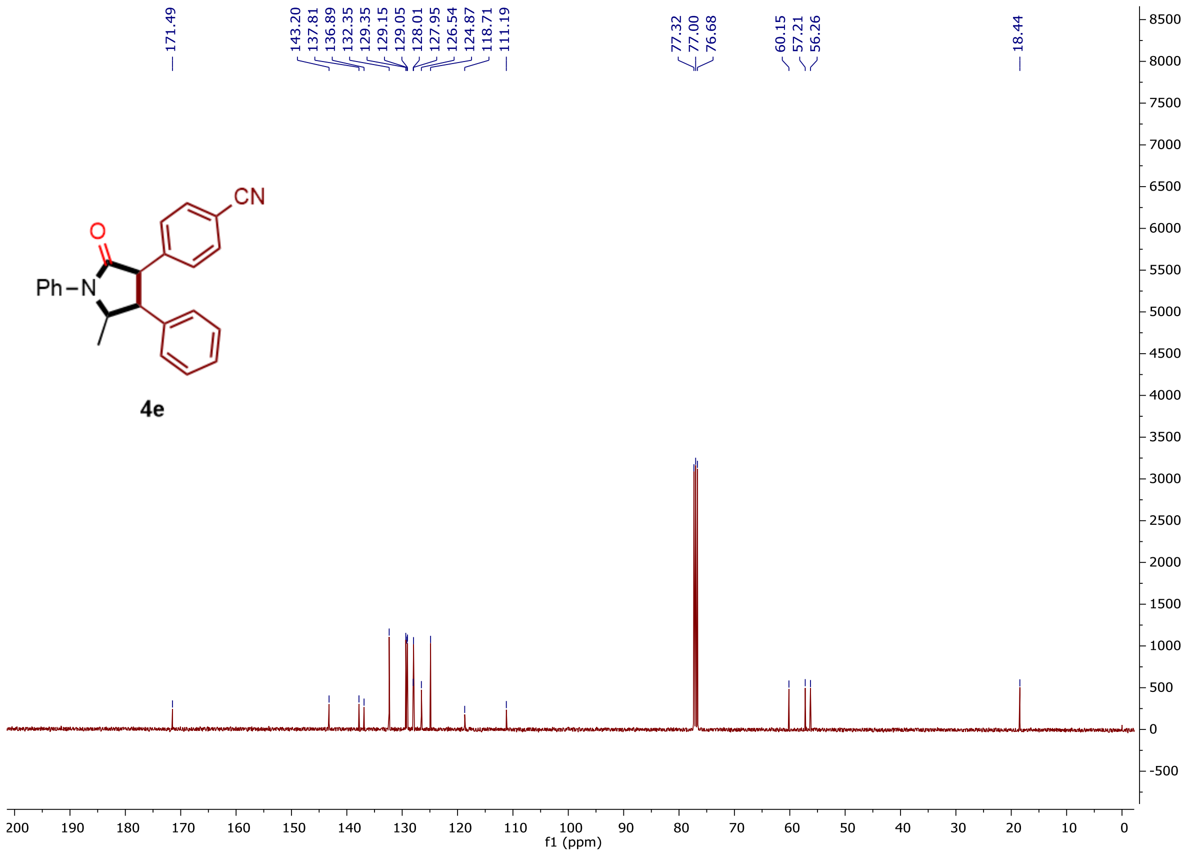
171.49
143.20 137.81 136.89 132.35 129.15 129.05 128.01 127.95 126.54 124.87 118.71 111.19
77.32 77.00 76.68
60.15 57.21 56.26
18.44
CN
O
Ph–N
4e
f1 (ppm)

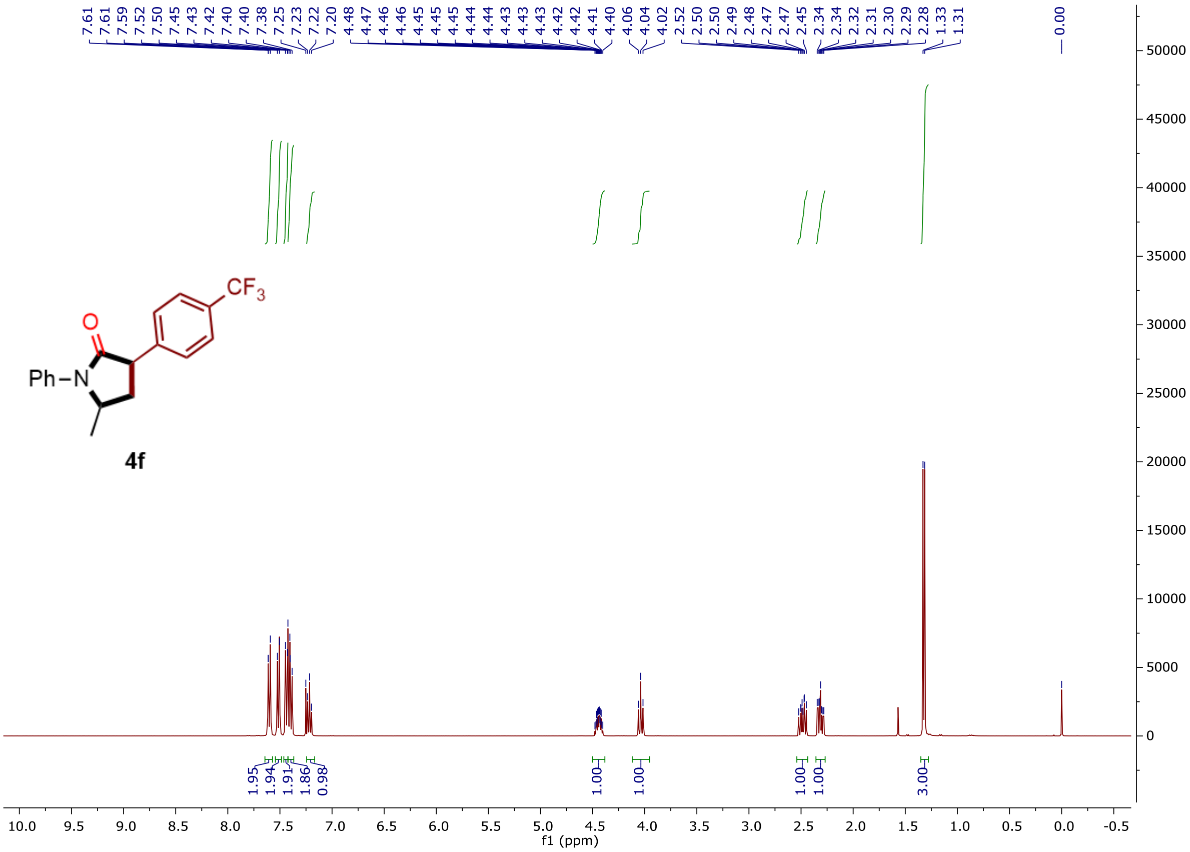

7.61
7.61
7.59
7.52
7.50
7.45
7.43
7.42
7.40
7.40
7.38
7.25
7.23
7.22
7.20
4.48
4.47
4.46
4.45
4.45
4.44
4.44
4.43
4.43
4.43
4.42
4.42
4.41
4.40
4.06
4.04
4.02
2.52
2.50
2.50
2.49
2.48
2.47
2.47
2.45
2.34
2.34
2.32
2.31
2.30
2.29
2.28
1.33
1.31
0.00
CF3
O
Ph–N
1.95
1.94
1.91
1.86
0.98
1.00
1.00
1.00
1.00
3.00
f1 (ppm)

4f

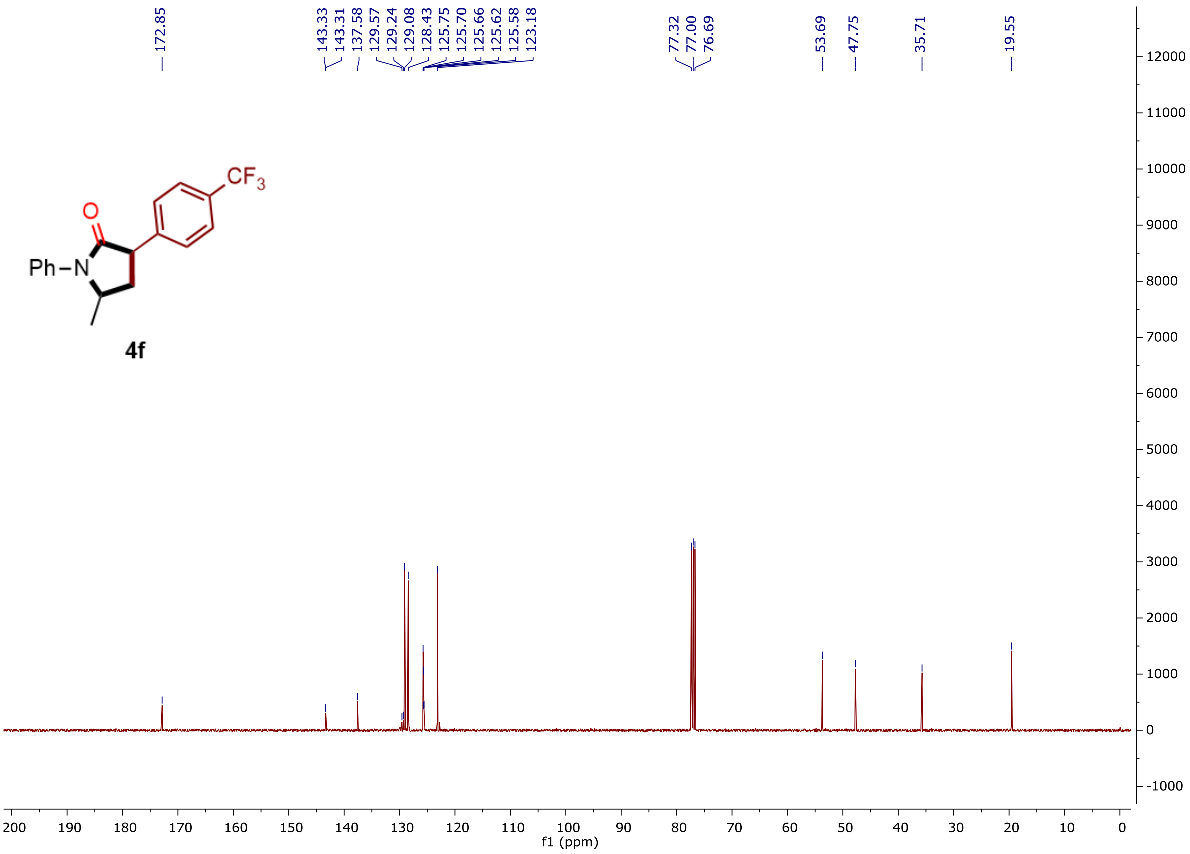

172.85
143.33
143.31
137.58
129.57
129.24
129.08
128.43
125.75
125.70
125.66
125.62
125.58
123.18
77.32
77.00
76.69
53.69
47.75
35.71
19.55
CF3
O
Ph–N
f1 (ppm)

4f

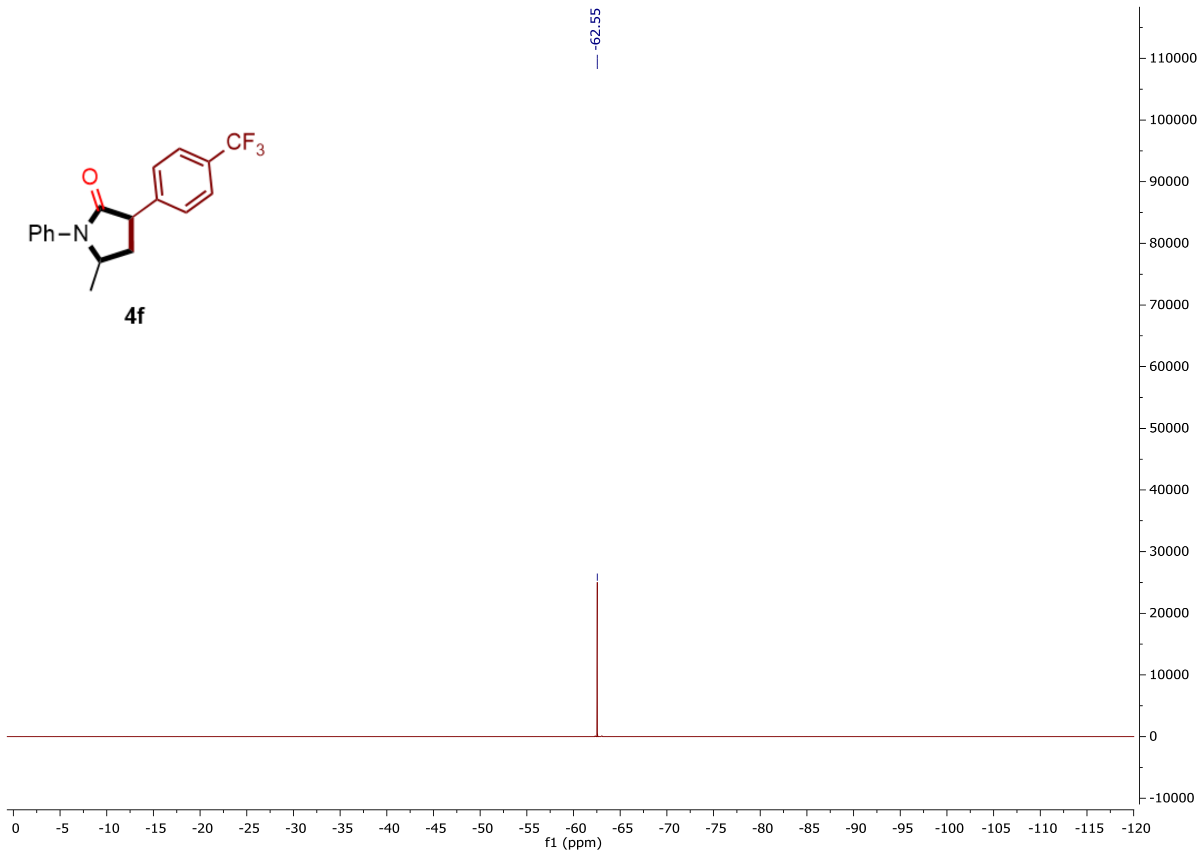
-62.55
CF3
O
Ph–N
4f
f1 (ppm)

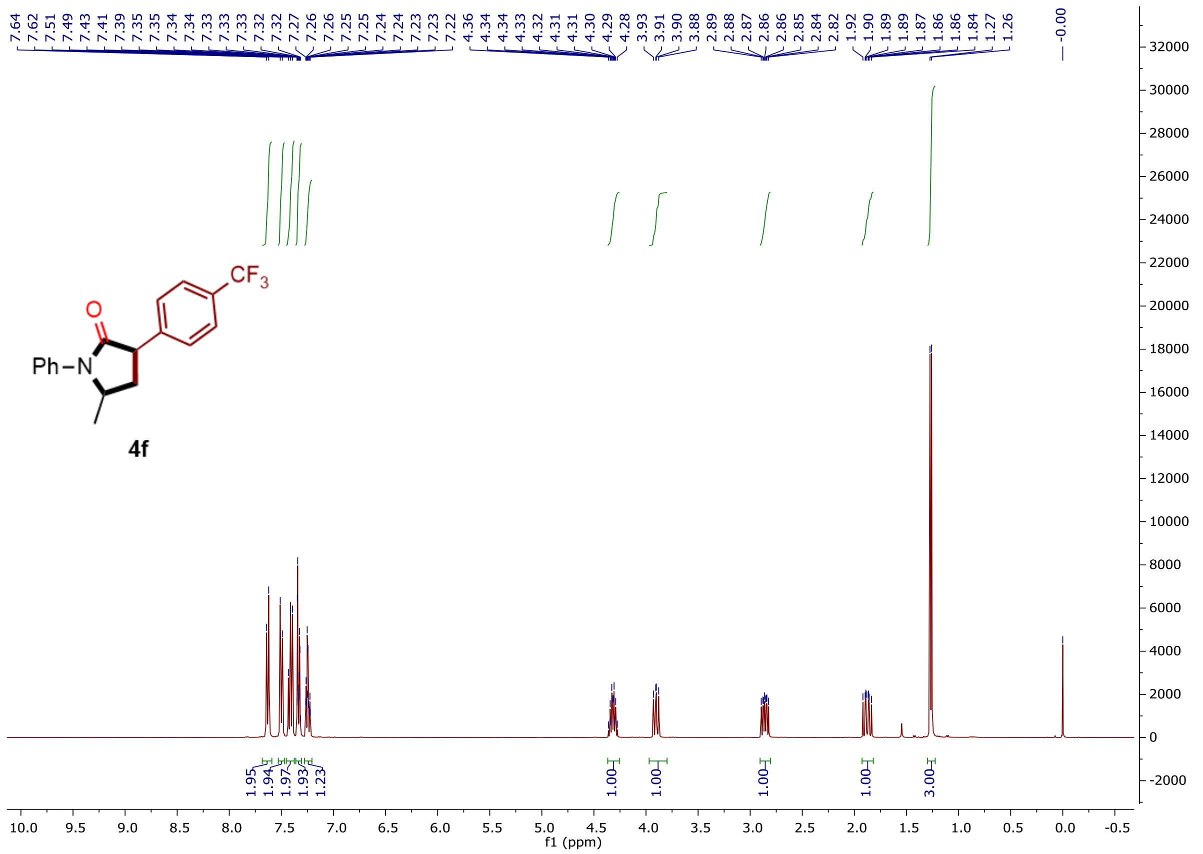
7.64 7.62 7.51 7.49 7.43 7.41 7.39 7.35 7.35 7.34 7.34 7.33 7.33 7.33 7.32 7.32 7.27 7.26 7.26 7.25 7.25 7.24 7.24 7.23 7.23 7.22 4.36 4.34 4.34 4.33 4.32 4.31 4.31 4.30 4.29 4.28 3.93 3.91 3.90 3.88 2.89 2.88 2.87 2.86 2.86 2.85 2.84 2.82 1.92 1.90 1.89 1.89 1.87 1.86 1.86 1.84 1.27 1.26
-0.00
CF3
O
Ph–N
4f
1.95 1.94 1.97 1.93 1.23
1.00
1.00
1.00
1.00
3.00
f1 (ppm)

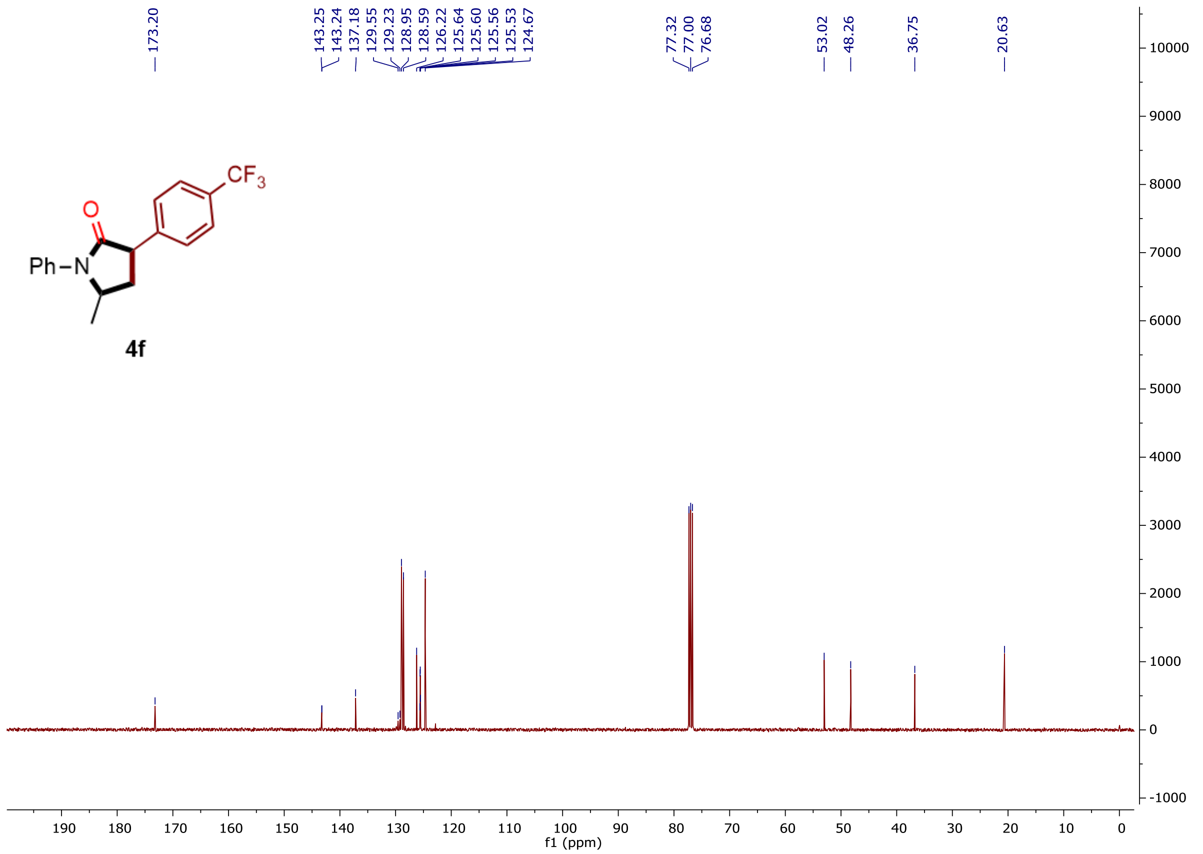
173.20
143.25
143.24
137.18
129.55
129.23
128.95
128.59
126.22
125.64
125.60
125.56
125.53
124.67
77.32
77.00
76.68
53.02
48.26
36.75
20.63
CF3
O
Ph−N
f1 (ppm)

4f

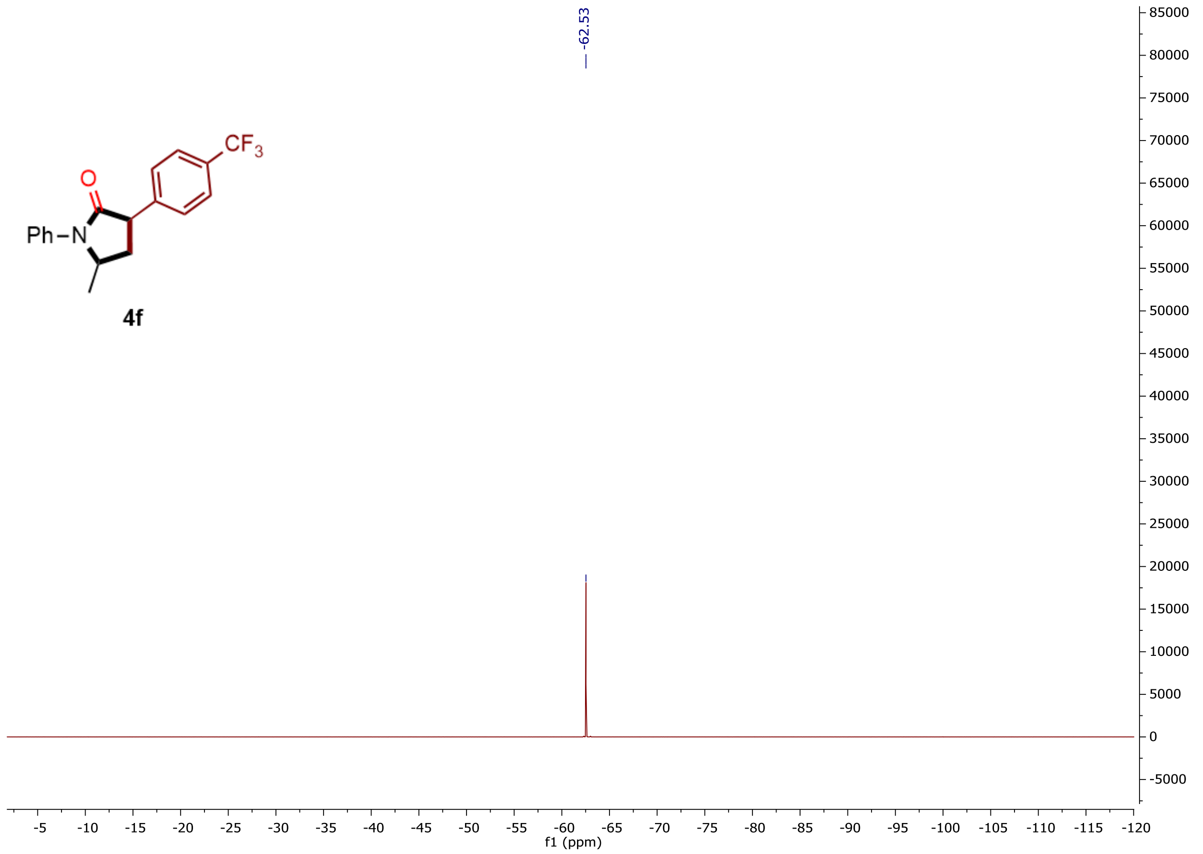
-62.53
CF3
O
Ph−N
f1 (ppm)

4f

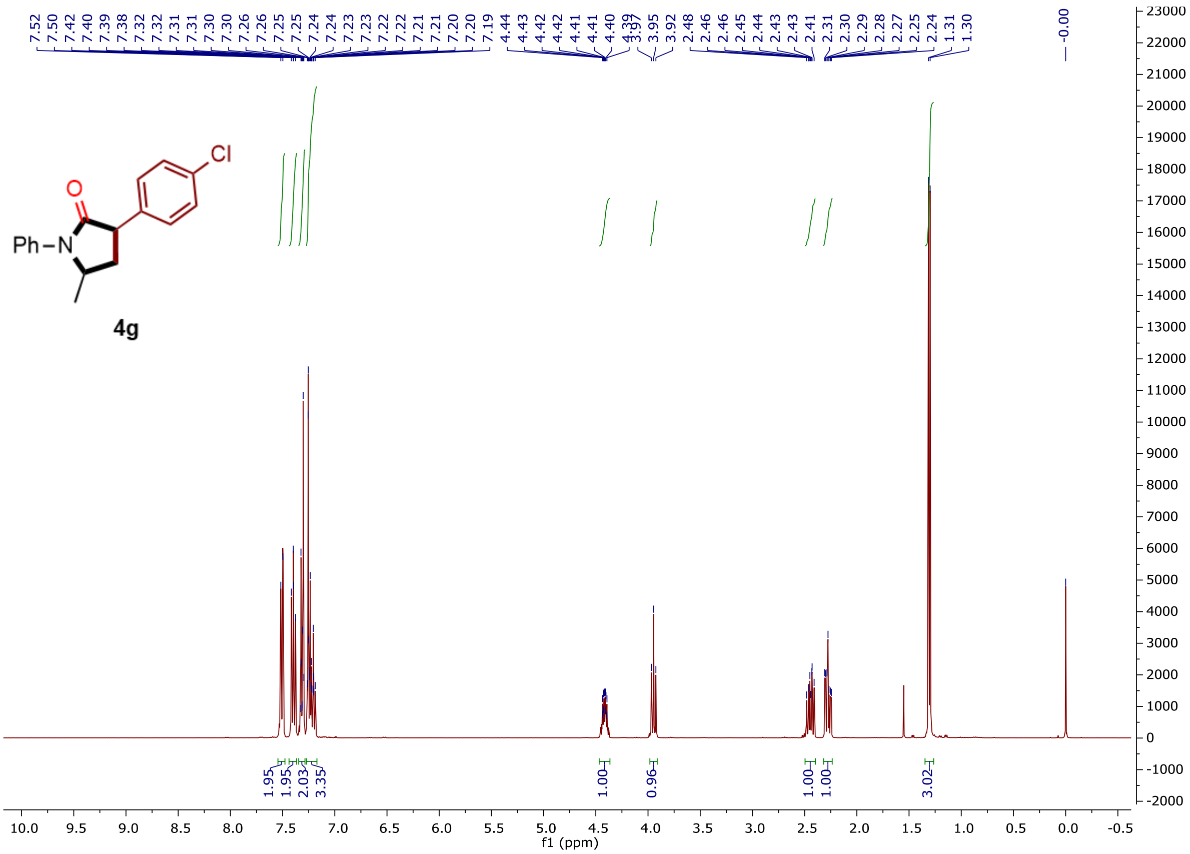

Cl
O
Ph−N
4g
7.52
7.50
7.42
7.40
7.39
7.38
7.32
7.32
7.31
7.31
7.30
7.30
7.26
7.25
7.25
7.24
7.24
7.23
7.23
7.22
7.22
7.21
7.21
7.20
7.20
7.19
4.44
4.43
4.42
4.42
4.41
4.41
4.40
4.39
3.97
3.95
3.92
2.48
2.46
2.46
2.45
2.44
2.43
2.43
2.41
2.31
2.30
2.29
2.28
2.27
2.25
2.24
1.31
1.30
-0.00
1.95
1.95
2.03
3.35
1.00
0.96
1.00
1.00
3.02
f1 (ppm)

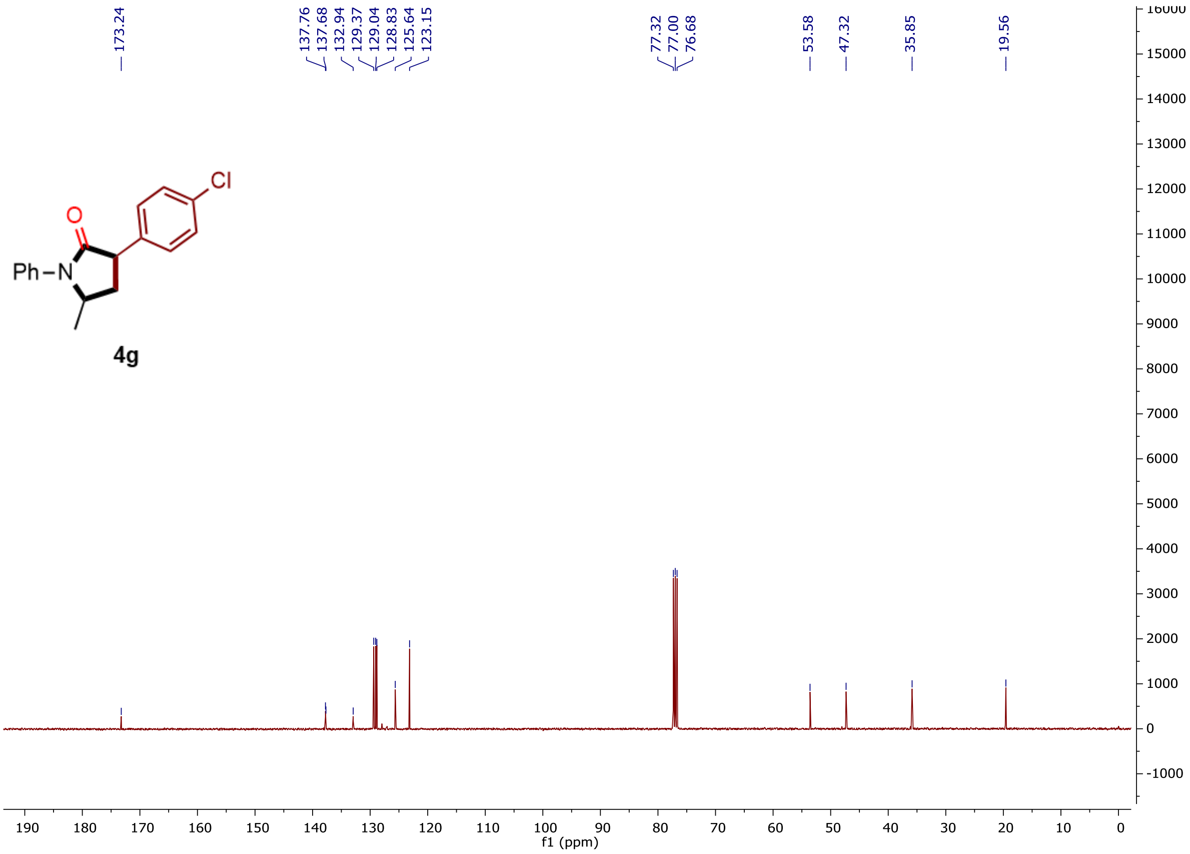

Cl
O
Ph−N
4g
173.24
137.76
137.68
132.94
129.37
129.04
128.83
125.64
123.15
77.32
77.00
76.68
53.58
47.32
35.85
19.56
f1 (ppm)

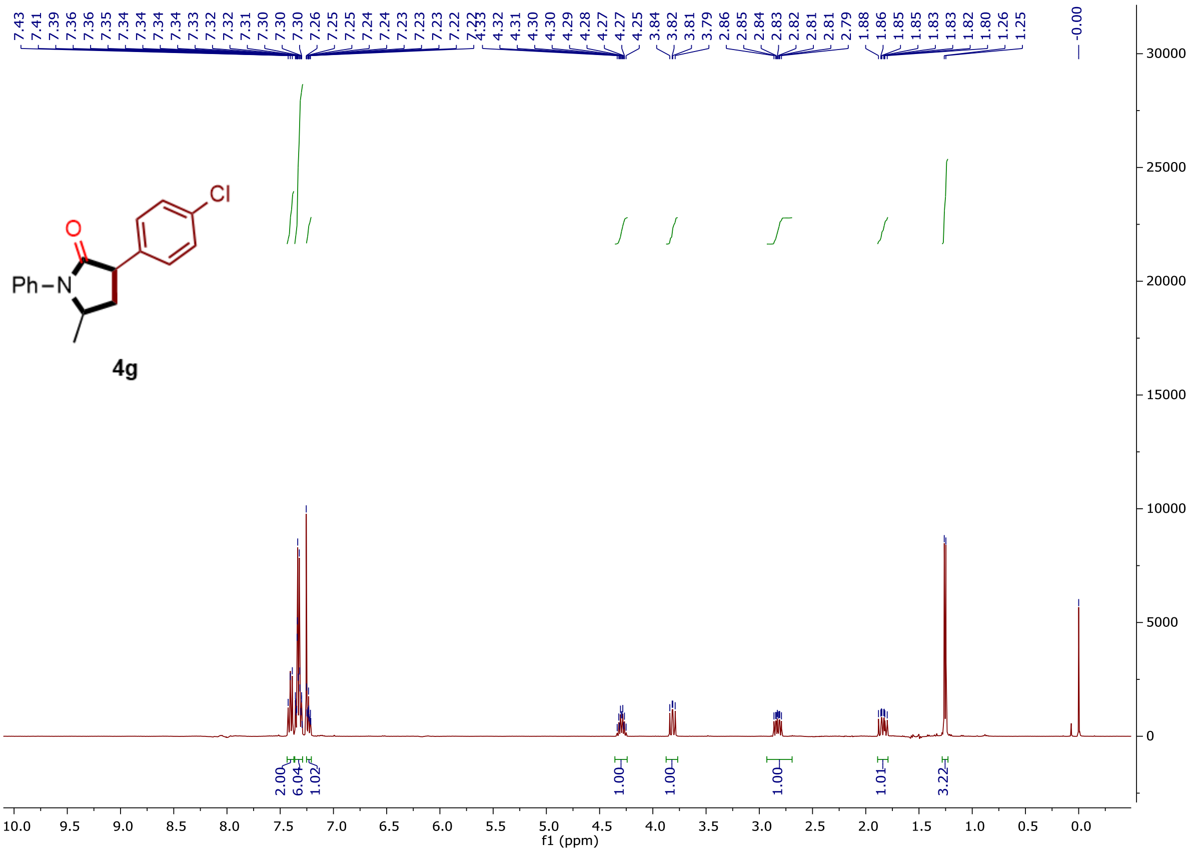
7.43 7.41 7.39 7.36 7.36 7.35 7.34 7.34 7.34 7.34 7.33 7.32 7.32 7.31 7.30 7.30 7.30 7.26 7.25 7.25 7.24 7.24 7.23 7.23 7.23 7.22 7.22 4.33 4.32 4.31 4.30 4.30 4.29 4.28 4.27 4.27 4.25 3.84 3.82 3.81 3.79 2.86 2.85 2.84 2.83 2.82 2.81 2.81 2.79 1.88 1.86 1.85 1.85 1.83 1.83 1.82 1.80 1.26 1.25
-0.00
2.00 6.04 1.02 1.00 1.00 1.00 1.01 3.22
Ph–N
O
Cl
4g
f1 (ppm)

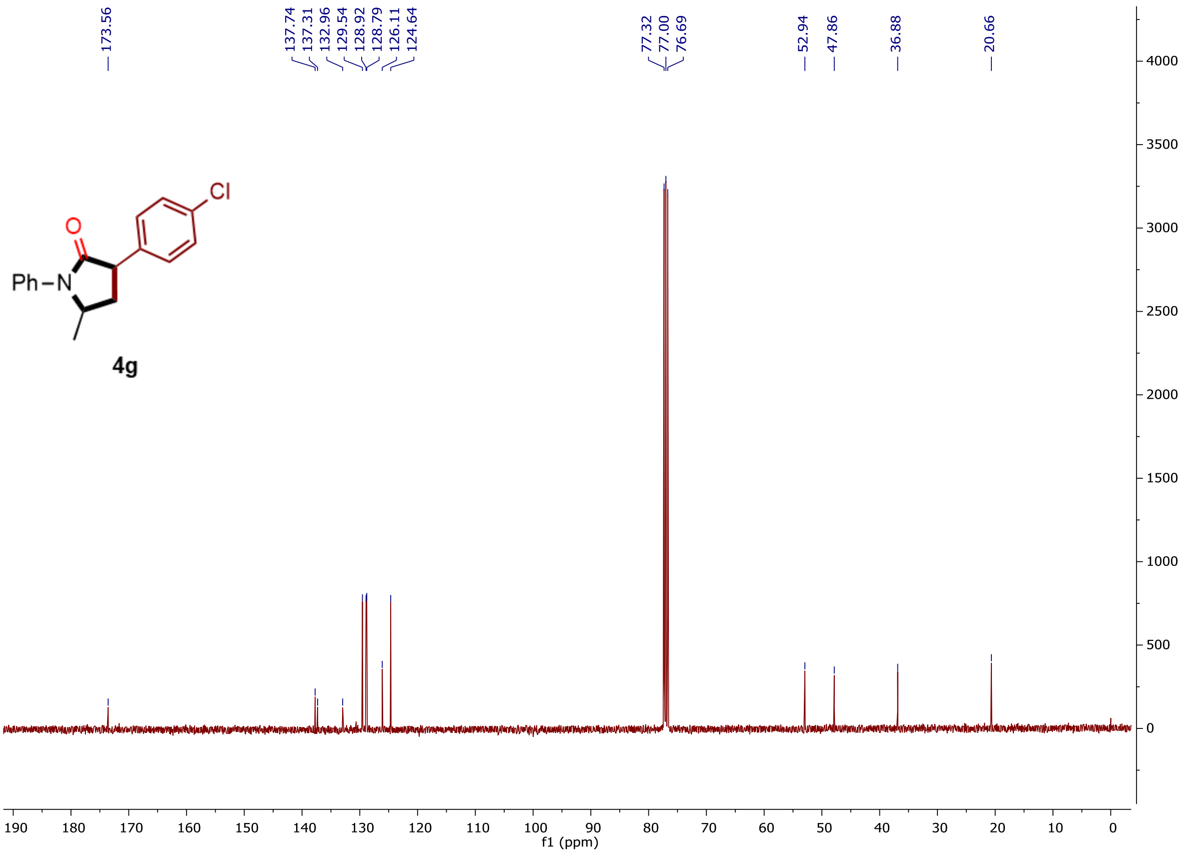
173.56
137.74 137.31 132.96 129.54 128.92 128.79 126.11 124.64
77.32 77.00 76.69
52.94
47.86
36.88
20.66
Ph–N
O
Cl
4g
f1 (ppm)

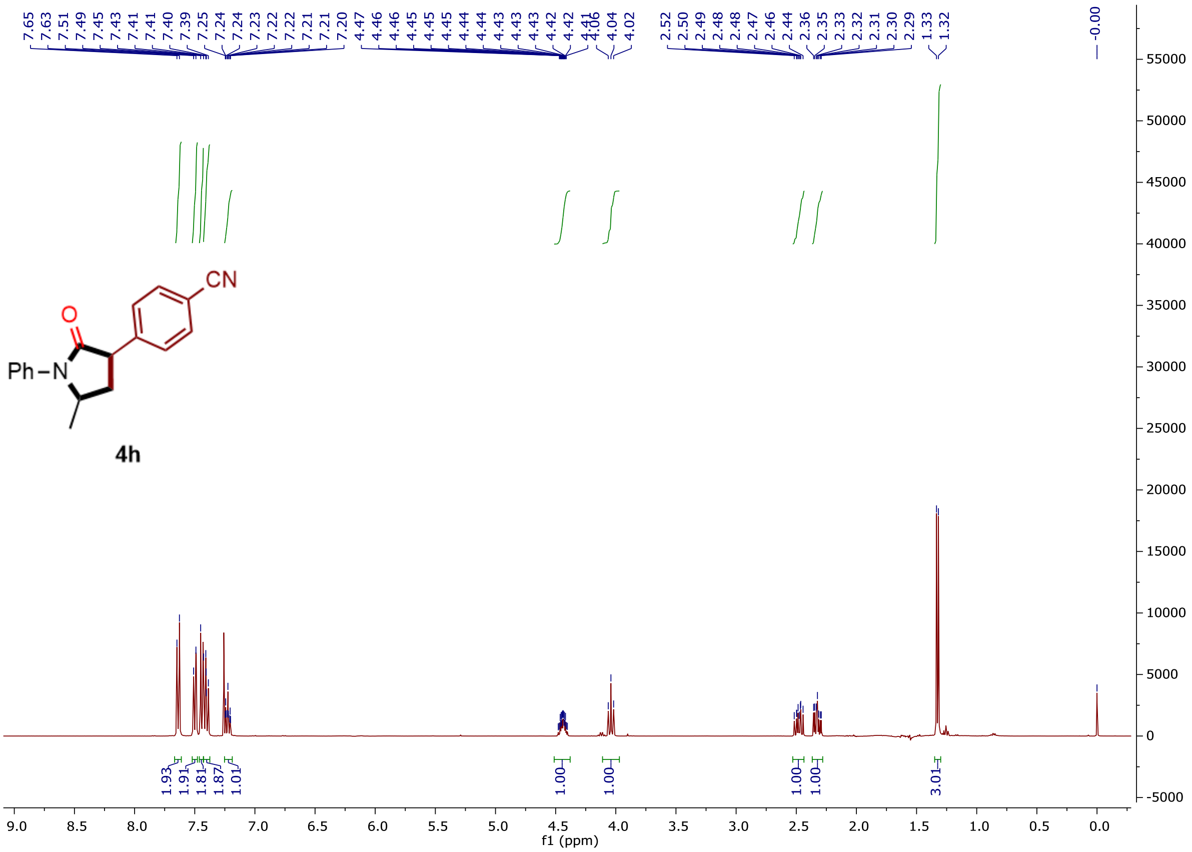
7.65 7.63 7.51 7.49 7.45 7.43 7.41 7.40 7.39 7.25 7.24 7.24 7.23 7.22 7.22 7.21 7.21 7.20 4.47 4.46 4.46 4.45 4.45 4.45 4.44 4.44 4.43 4.43 4.43 4.42 4.42 4.06 4.04 4.02 2.52 2.50 2.49 2.48 2.47 2.46 2.44 2.36 2.35 2.33 2.32 2.31 2.30 2.29 1.33 1.32 -0.00
CN
O
Ph–N
4h
1.93 1.91 1.81 1.87 1.01 1.00 1.00 1.00 1.00 3.01
f1 (ppm)

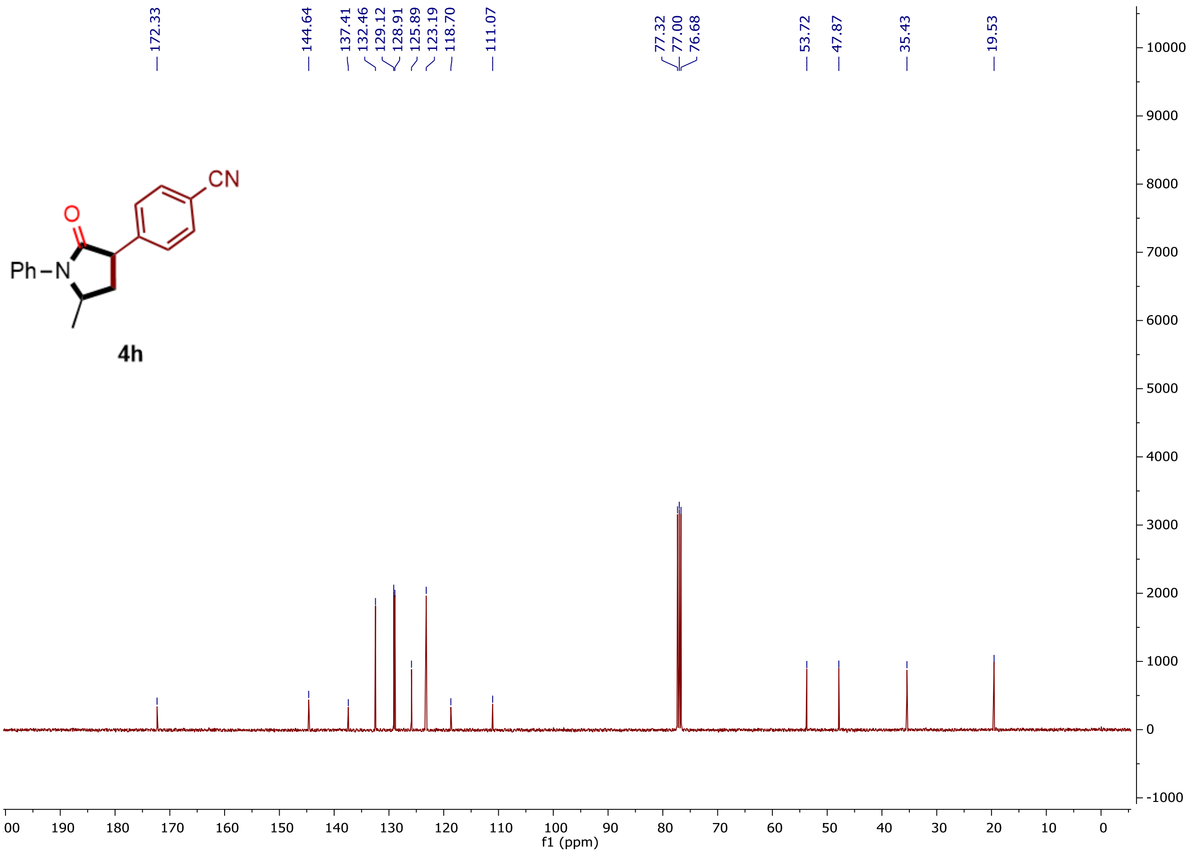
172.33 144.64 137.41 132.46 129.12 128.91 125.89 123.19 118.70 111.07 77.32 77.00 76.68 53.72 47.87 35.43 19.53
CN
O
Ph–N
4h
f1 (ppm)

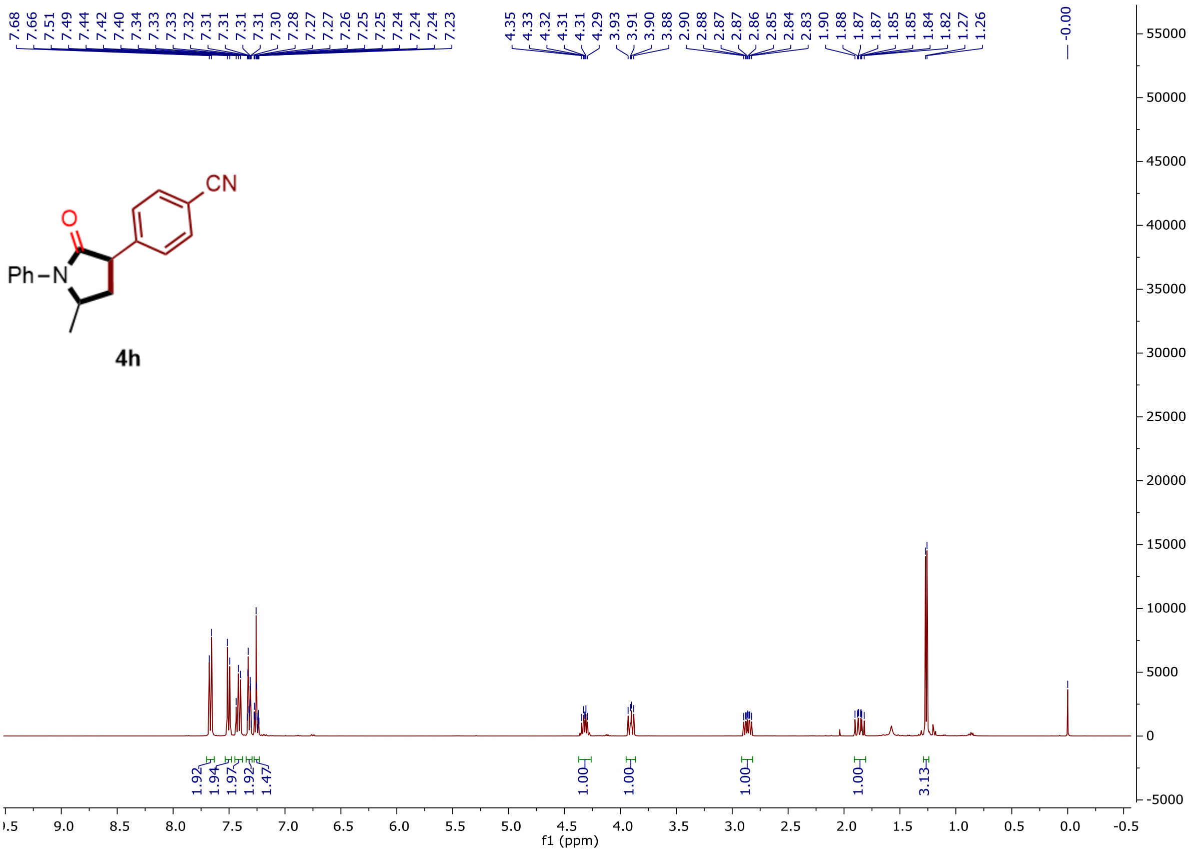
7.68 7.66 7.51 7.49 7.44 7.42 7.40 7.34 7.33 7.33 7.32 7.31 7.31 7.31 7.31 7.30 7.28 7.27 7.27 7.26 7.25 7.25 7.24 7.24 7.24 7.23
4.35 4.33 4.32 4.31 4.31 4.29 3.93 3.91 3.90 3.88 2.90 2.88 2.87 2.87 2.86 2.85 2.84 2.83 1.90 1.88 1.87 1.87 1.85 1.85 1.84 1.82 1.27 1.26
-0.00
CN
O
Ph−N
4h
1.92 1.94 1.97 1.92 1.47
1.00 1.00 1.00 1.00 3.13
f1 (ppm)

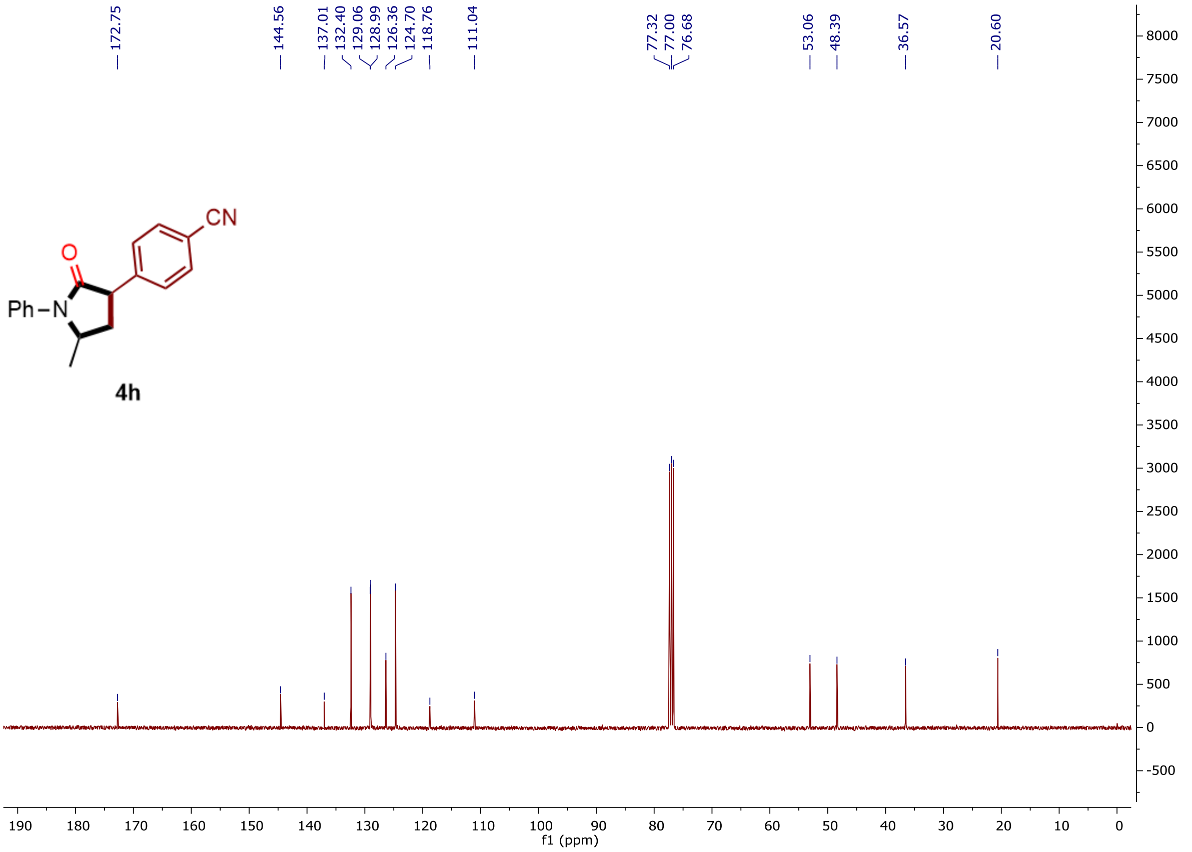
172.75
144.56
137.01 132.40 129.06 128.99 126.36 124.70 118.76
111.04
77.32 77.00 76.68
53.06 48.39
36.57
20.60
CN
O
Ph−N
4h
f1 (ppm)

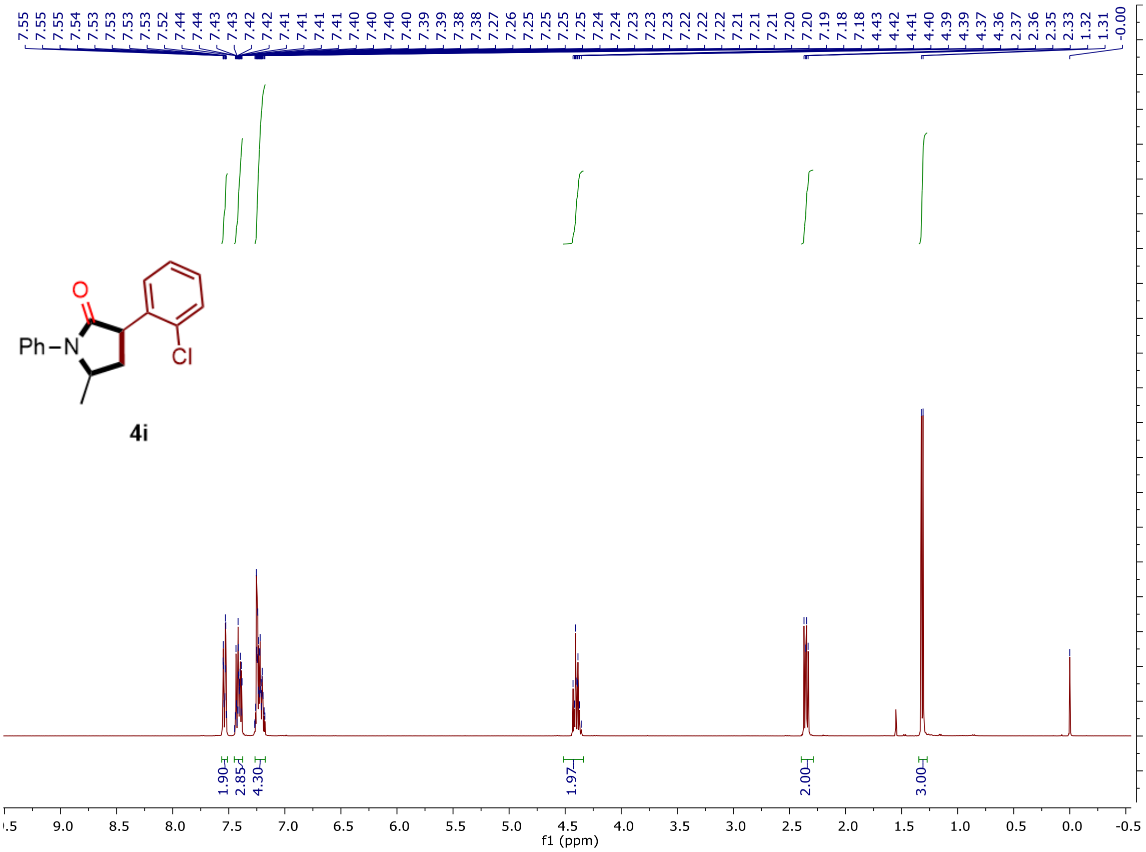

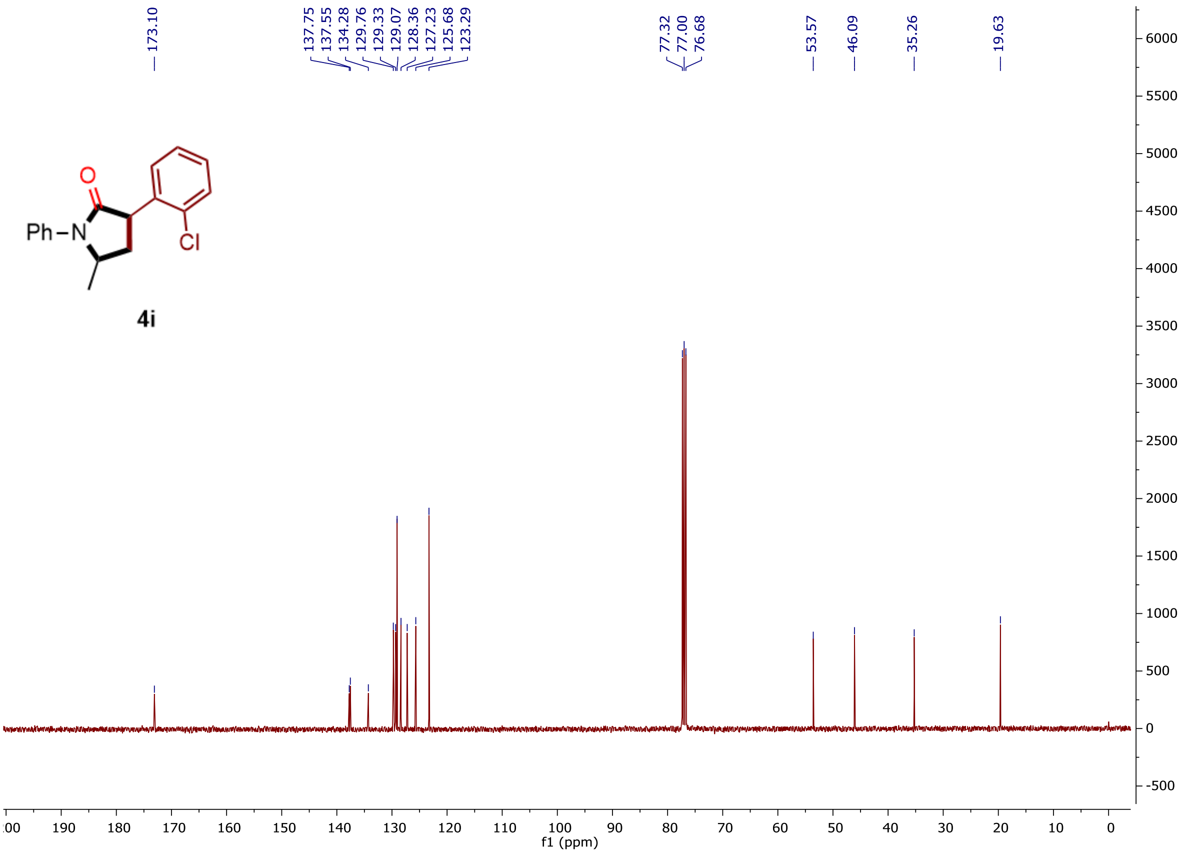

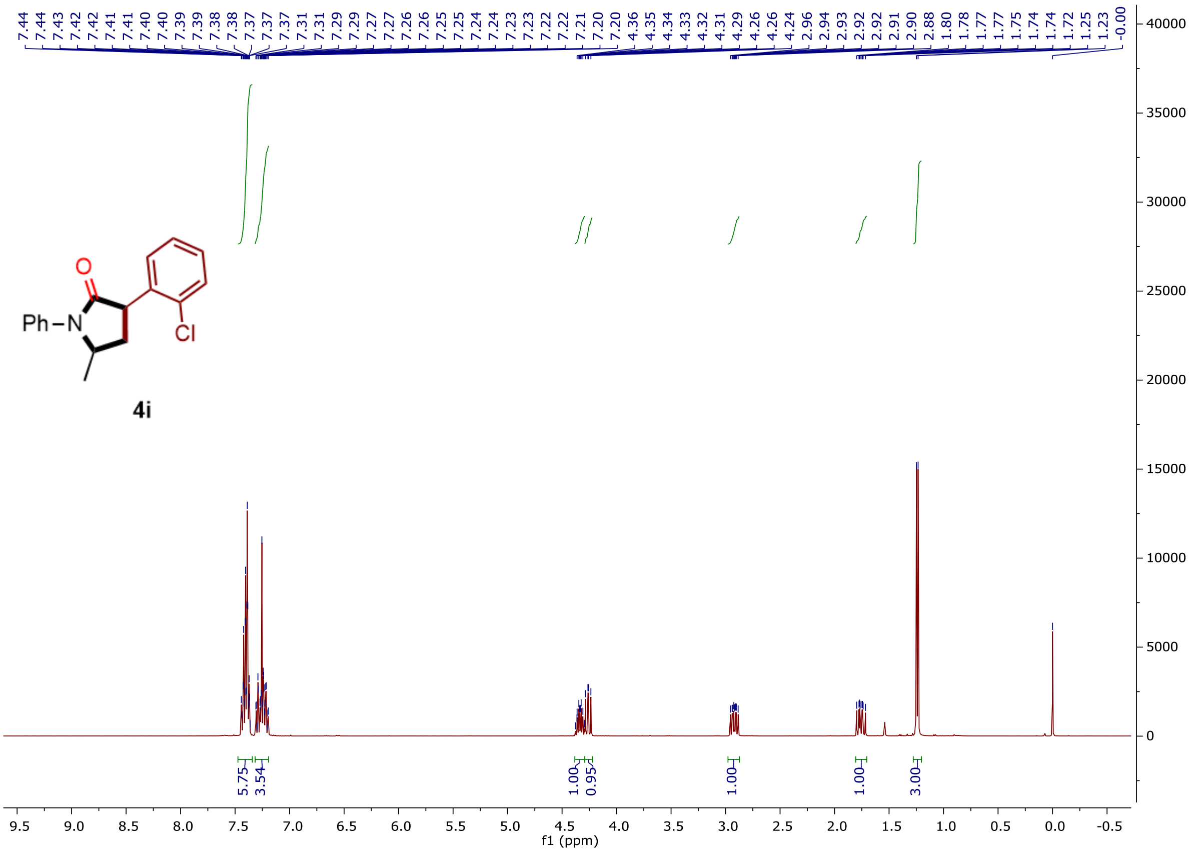
Ph–N
O
Cl
4i
f1 (ppm)

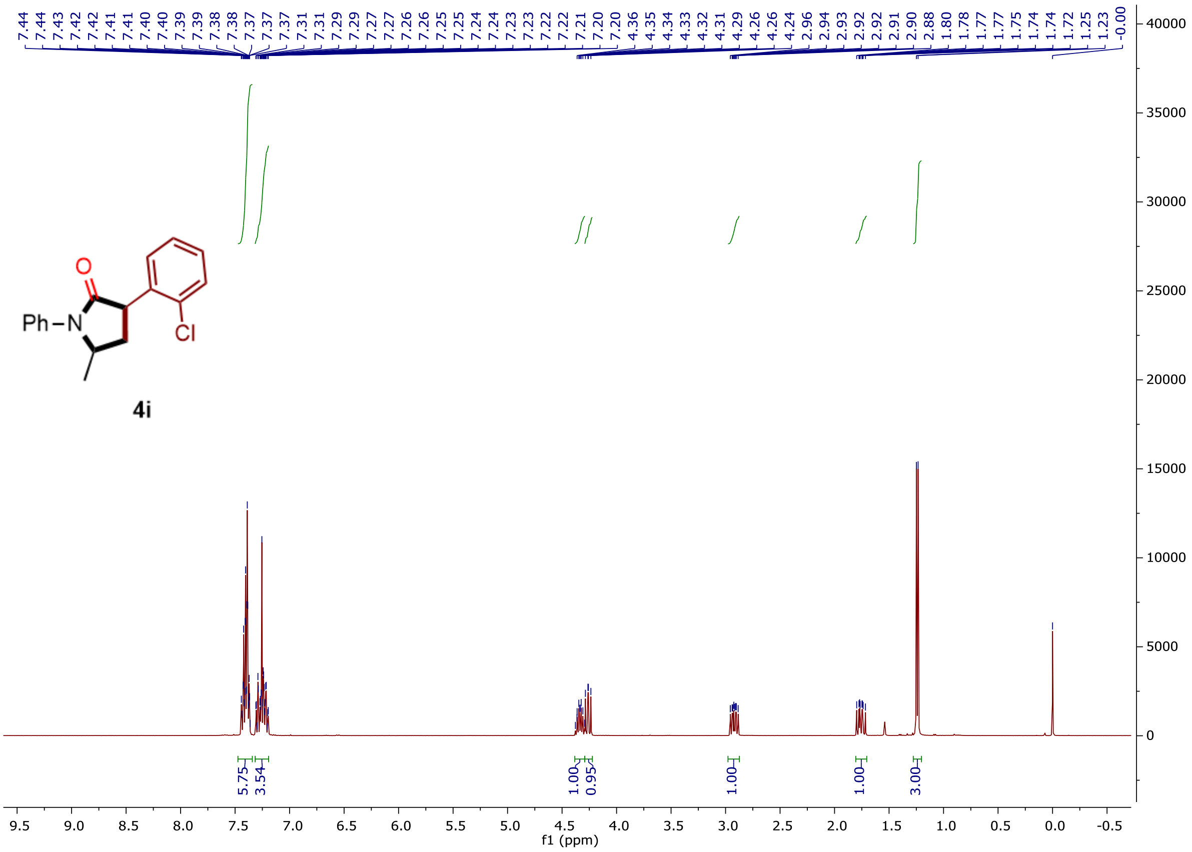
Ph–N
O
Cl
4i
f1 (ppm)

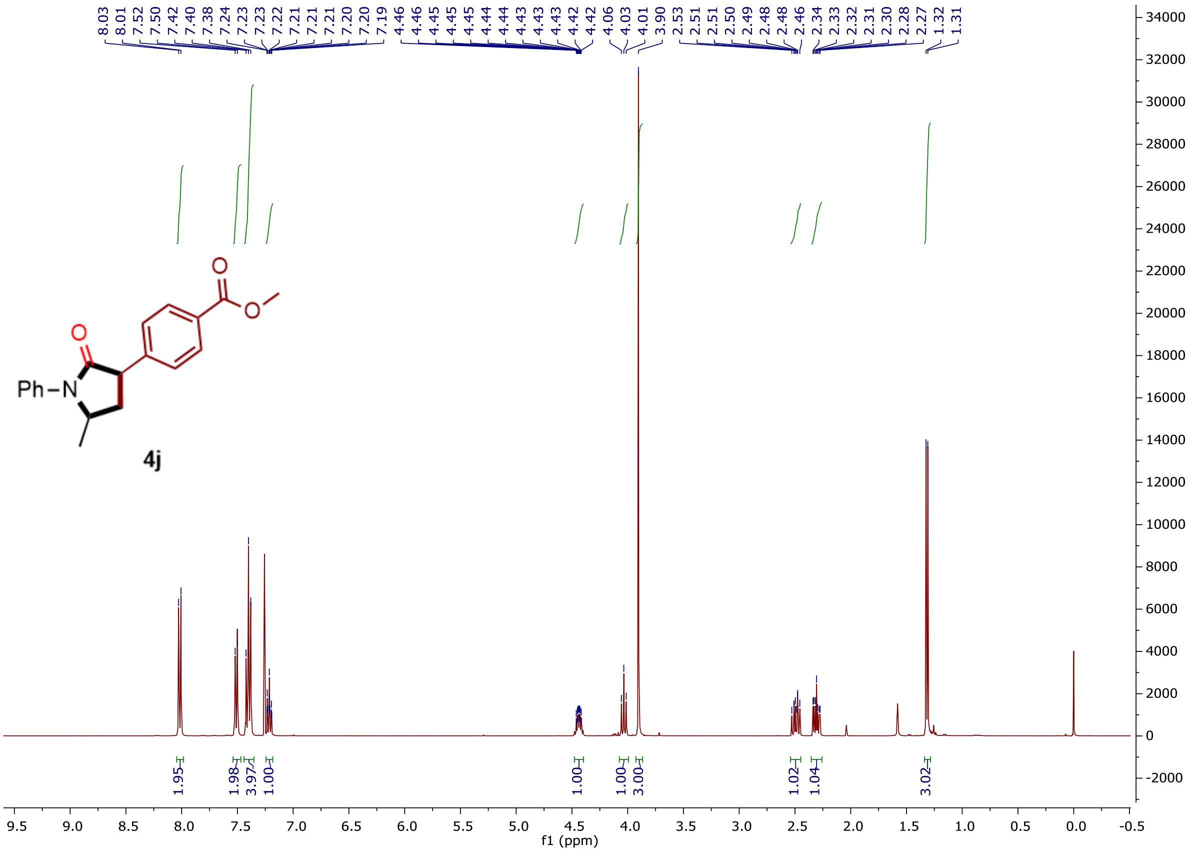
8.03
8.01
7.52
7.50
7.42
7.40
7.38
7.24
7.23
7.23
7.22
7.21
7.21
7.21
7.20
7.19
4.46
4.46
4.45
4.45
4.45
4.44
4.44
4.43
4.43
4.43
4.42
4.42
4.06
4.03
4.01
3.90
2.53
2.51
2.51
2.50
2.49
2.48
2.48
2.46
2.34
2.33
2.32
2.31
2.30
2.28
2.27
1.32
1.31
Ph–N
4j
1.95
1.98
3.97
1.00
1.00
1.00
3.00
1.02
1.04
3.02
f1 (ppm)

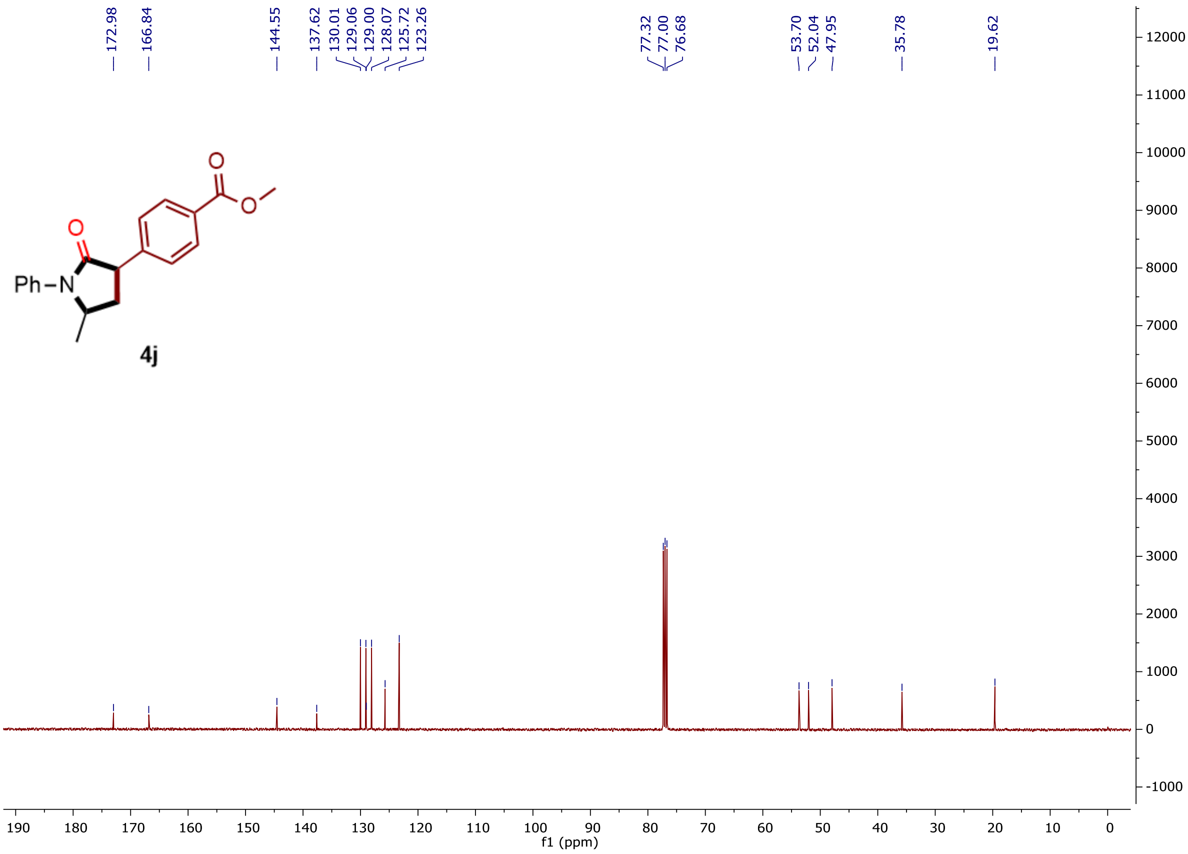
172.98
166.84
144.55
137.62
130.01
129.06
129.00
128.07
125.72
123.26
77.32
77.00
76.68
53.70
52.04
47.95
35.78
19.62
Ph–N
4j
f1 (ppm)

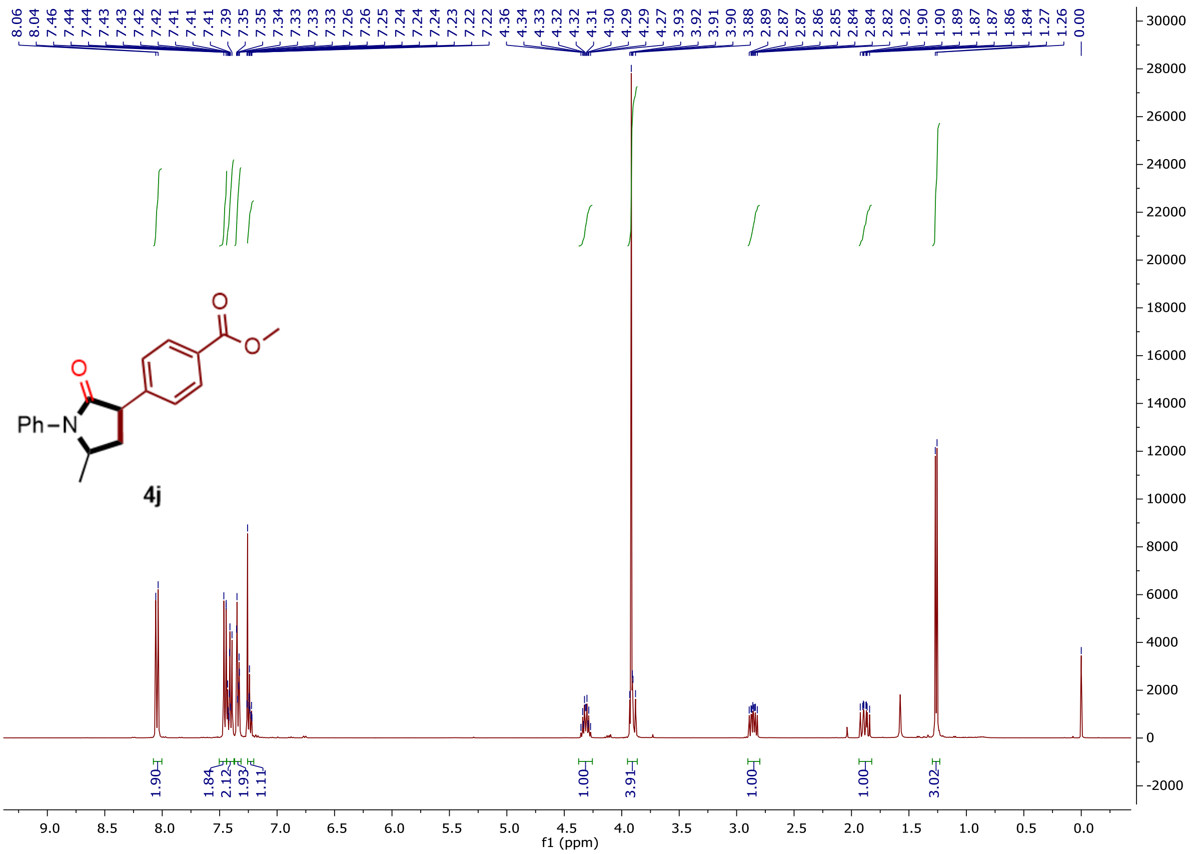
8.06 8.04 7.46 7.44 7.44 7.43 7.43 7.42 7.42 7.41 7.41 7.41 7.39 7.35 7.35 7.34 7.33 7.33 7.33 7.26 7.26 7.25 7.24 7.24 7.24 7.23 7.22 7.22 4.36 4.34 4.33 4.32 4.32 4.31 4.30 4.29 4.29 4.27 3.93 3.92 3.91 3.90 3.88 2.89 2.87 2.87 2.86 2.85 2.84 2.84 2.82 1.92 1.90 1.90 1.89 1.87 1.87 1.86 1.84 1.27 1.26 0.00
Ph–N
O
4j
1.90 1.84 2.12 1.93 1.11 1.00 3.91 1.00 1.00 3.02
f1 (ppm)

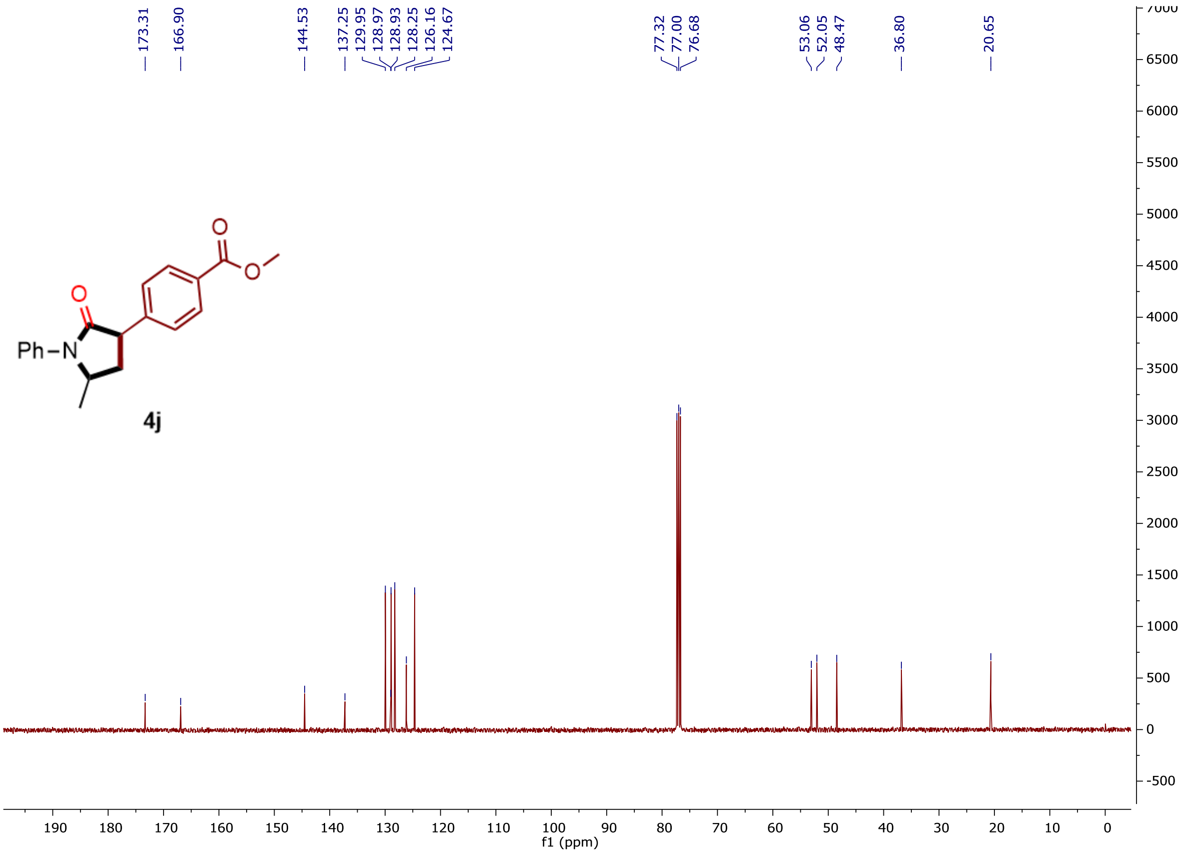
173.31 166.90 144.53 137.25 129.95 128.97 128.93 128.25 126.16 124.67 77.32 77.00 76.68 53.06 52.05 48.47 36.80 20.65
Ph–N
O
4j
f1 (ppm)

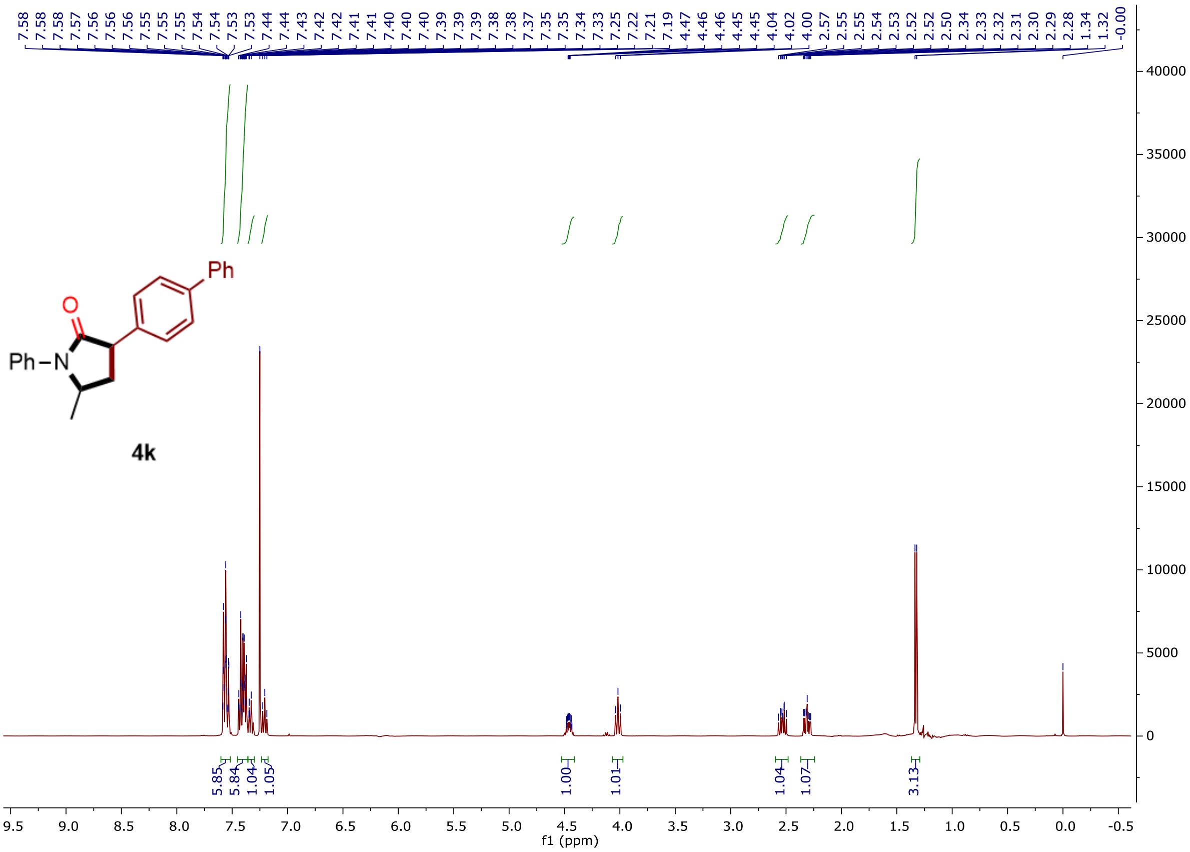
7.58 7.58 7.58 7.57 7.56 7.56 7.56 7.55 7.55 7.55 7.54 7.54 7.53 7.53 7.44 7.44 7.43 7.42 7.42 7.41 7.41 7.40 7.40 7.39 7.39 7.39 7.38 7.38 7.37 7.35 7.35 7.34 7.33 7.25 7.22 7.21 7.19 4.47 4.46 4.46 4.45 4.45 4.04 4.02 4.00 2.57 2.55 2.55 2.54 2.53 2.52 2.52 2.50 2.34 2.33 2.32 2.31 2.30 2.29 2.28 1.34 1.32 -0.00
Ph
O
Ph–N
4k
5.85 5.84 1.04 1.05 1.00 1.01 1.04 1.07 3.13
f1 (ppm)

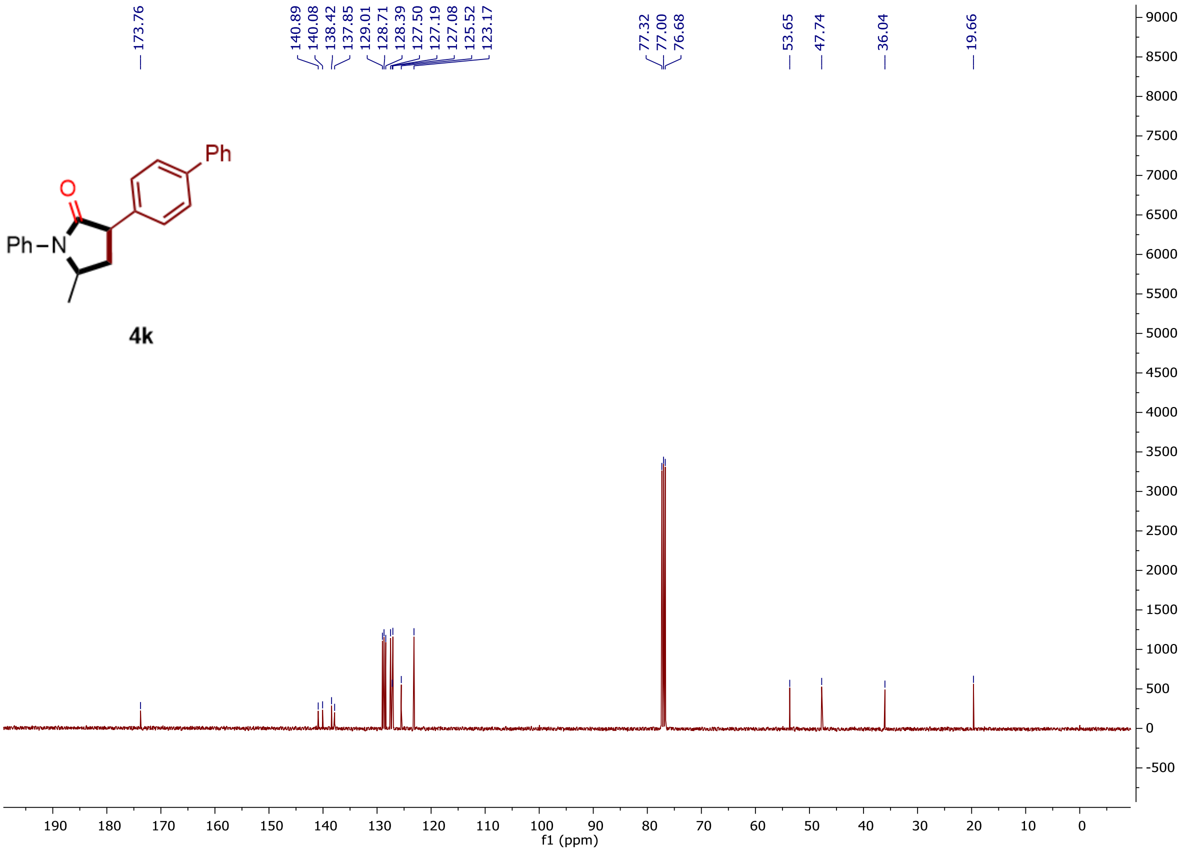
173.76
140.89 140.08 138.42 137.85 129.01 128.71 128.39 127.50 127.19 127.08 125.52 123.17
77.32 77.00 76.68
53.65 47.74 36.04 19.66
Ph
O
Ph–N
4k
f1 (ppm)

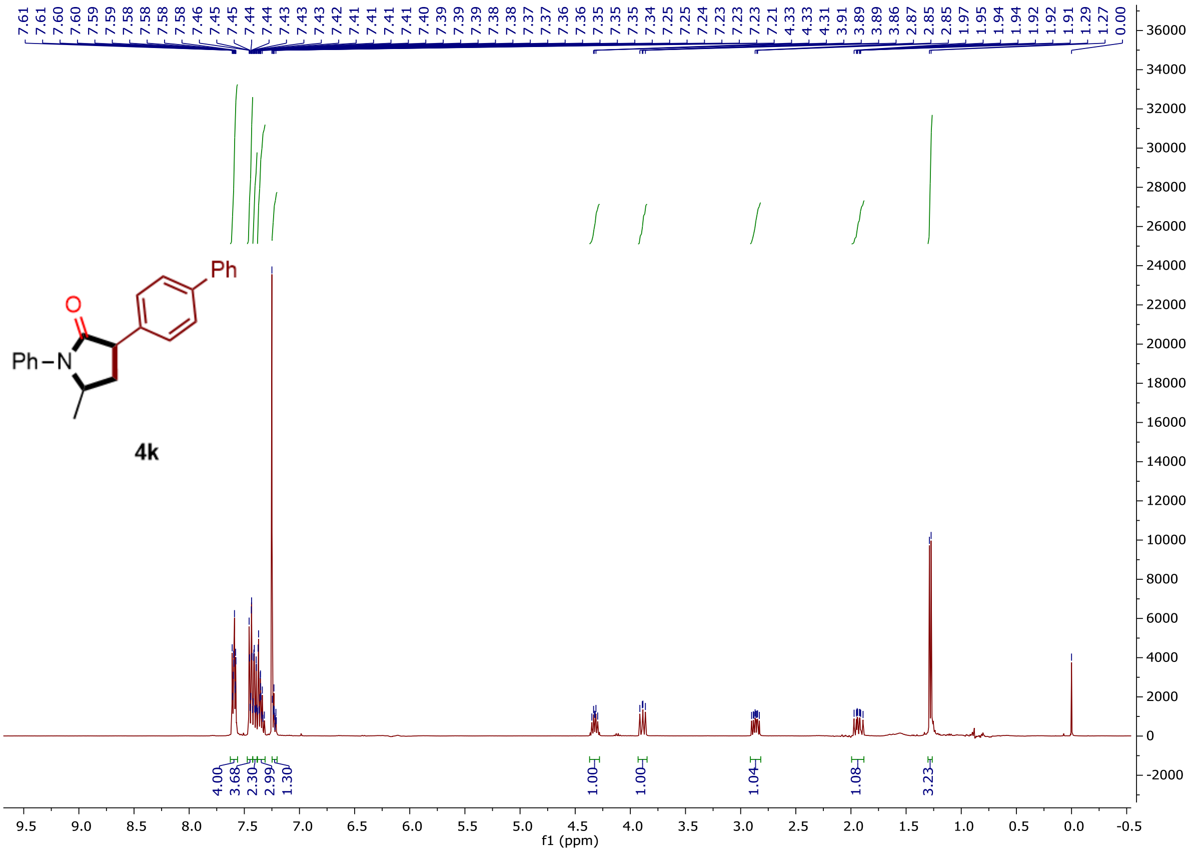
7.61
7.61
7.60
7.59
7.59
7.58
7.58
7.58
7.58
7.46
7.45
7.45
7.44
7.44
7.43
7.43
7.43
7.42
7.41
7.41
7.41
7.40
7.39
7.39
7.39
7.38
7.38
7.37
7.37
7.36
7.36
7.35
7.35
7.35
7.34
7.25
7.25
7.24
7.23
7.23
7.23
7.21
4.33
4.33
4.31
3.91
3.89
3.89
3.86
2.87
2.85
2.85
1.97
1.95
1.94
1.94
1.92
1.92
1.91
1.29
1.27
0.00
Ph
O
Ph–N
4.00
3.68
2.30
2.99
1.30
1.00
1.00
1.04
1.08
3.23
f1 (ppm)

4k

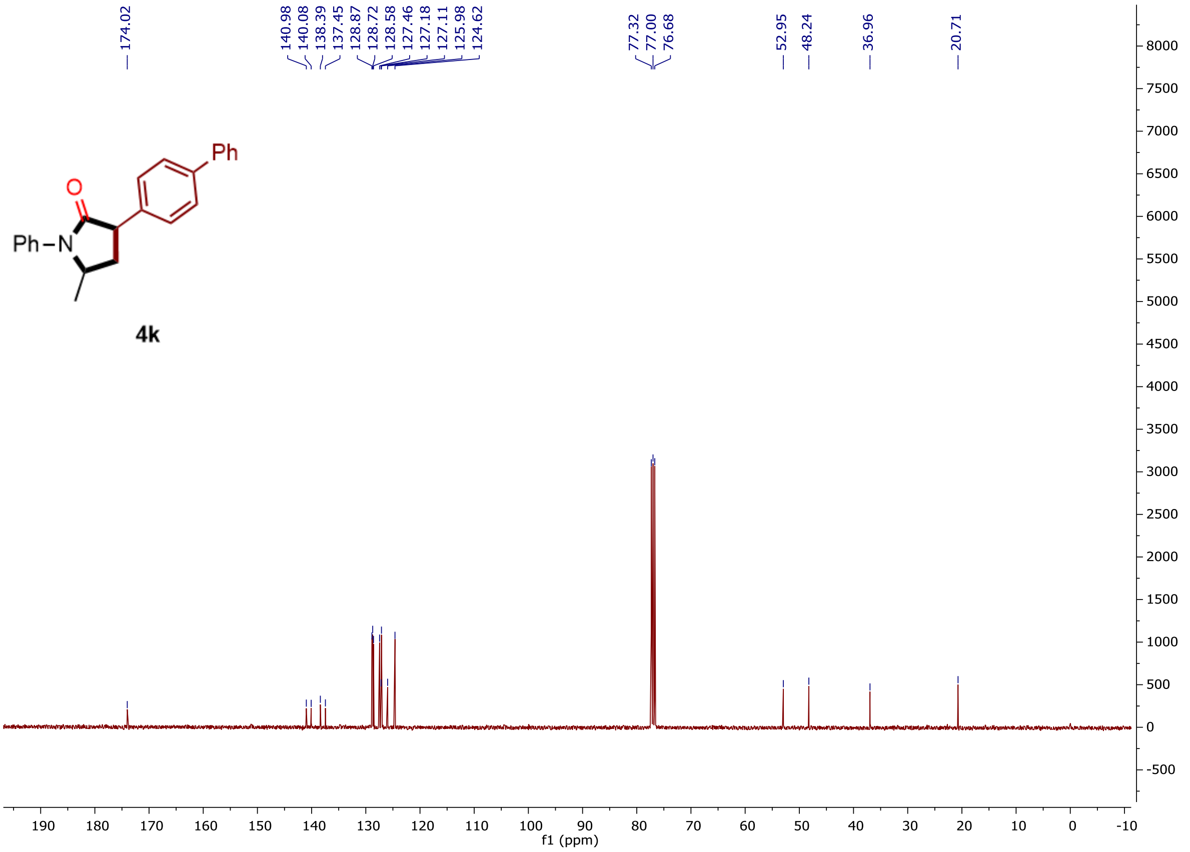
174.02
140.98
140.08
138.39
137.45
128.87
128.72
128.58
127.46
127.18
127.11
125.98
124.62
77.32
77.00
76.68
52.95
48.24
36.96
20.71
Ph
O
Ph–N
f1 (ppm)

4k

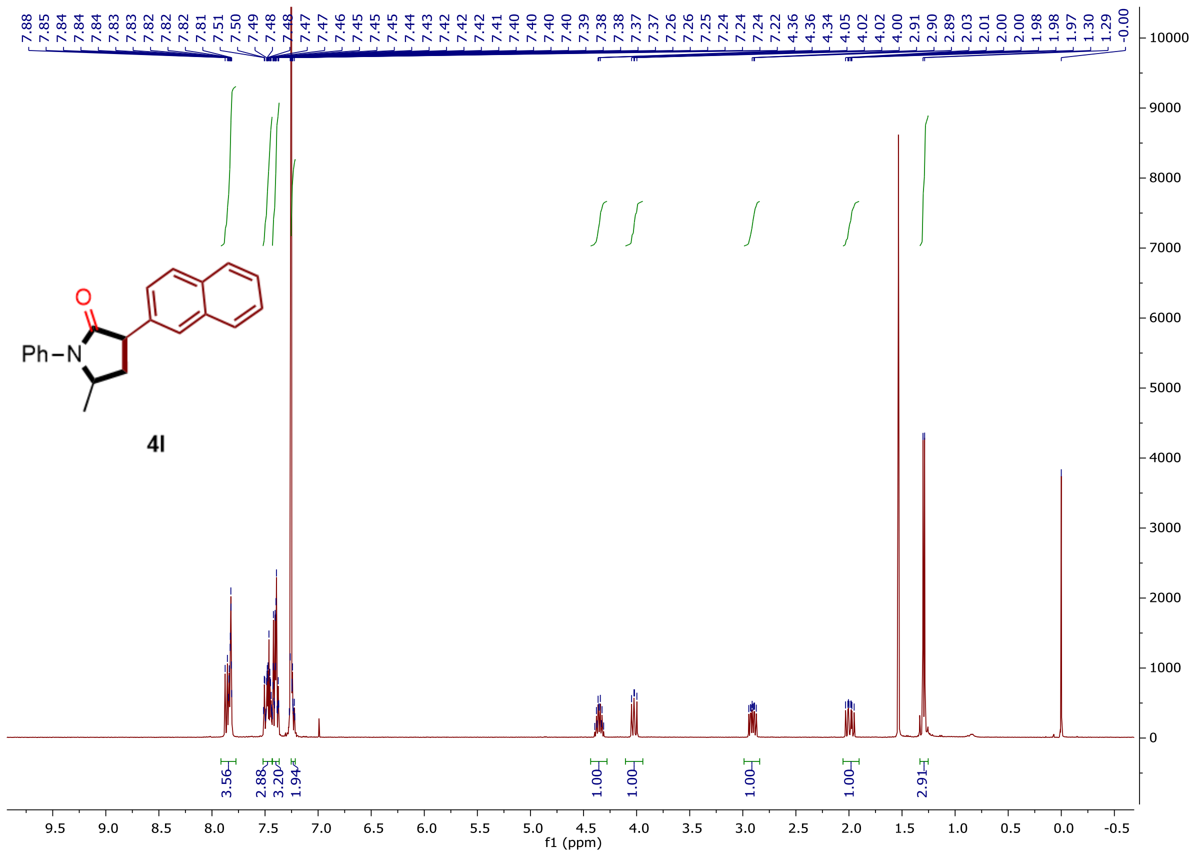
4l
f1 (ppm)

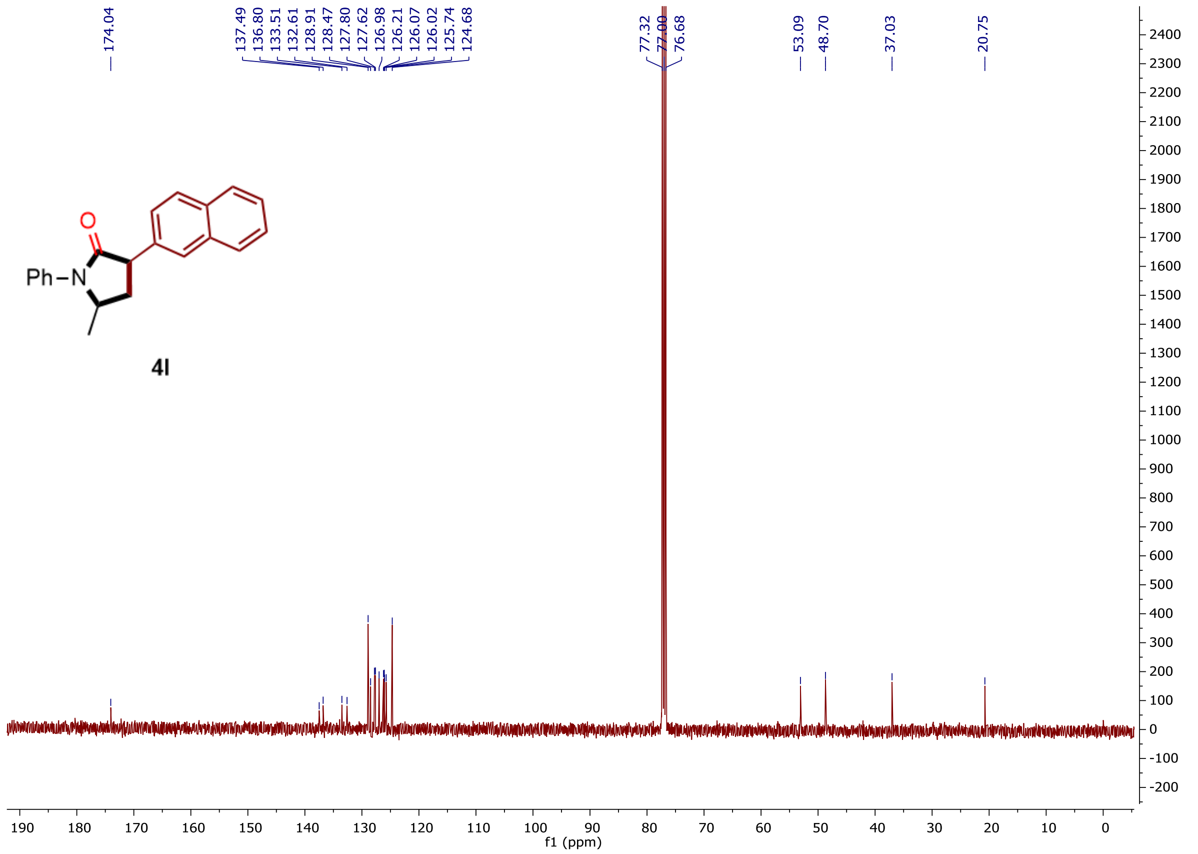
4l
f1 (ppm)

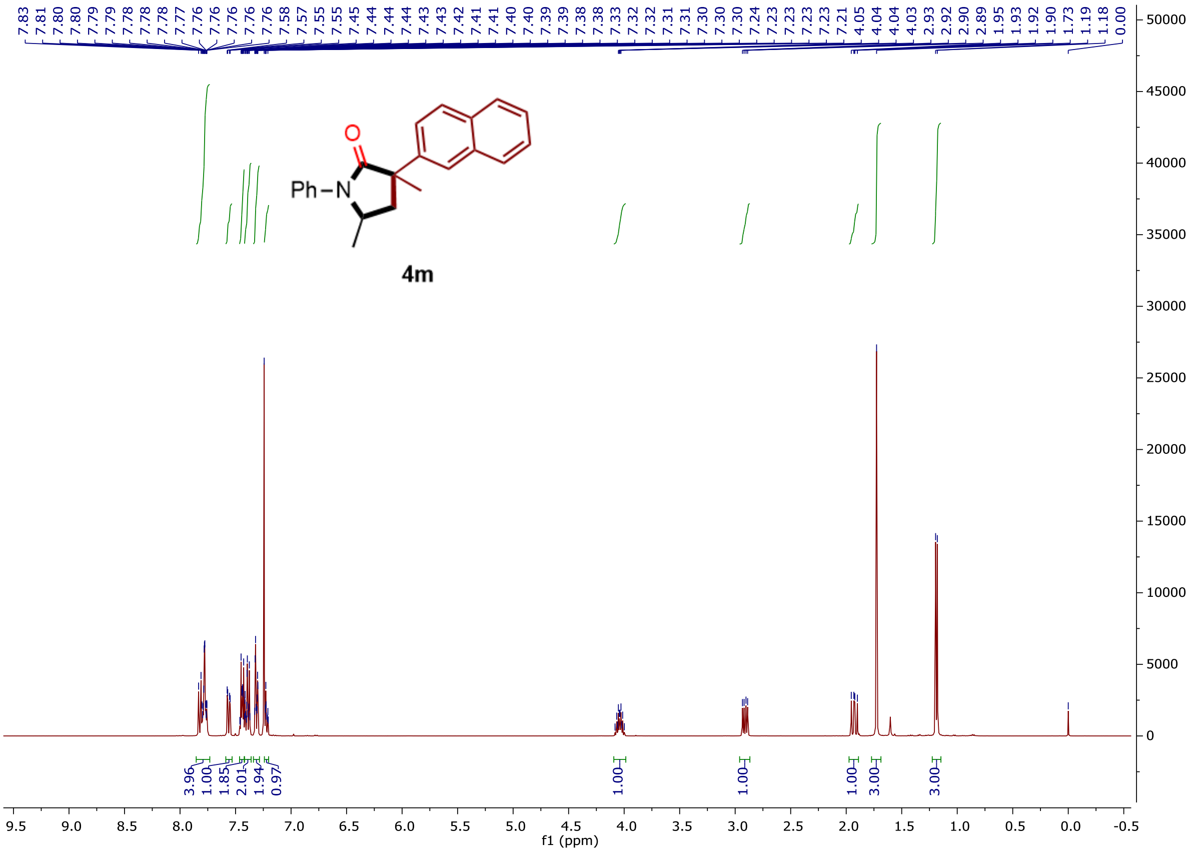
7.83 7.81 7.80 7.79 7.79 7.78 7.78 7.78 7.77 7.76 7.76 7.76 7.76 7.76 7.58 7.57 7.55 7.55 7.45 7.44 7.44 7.44 7.43 7.43 7.42 7.41 7.41 7.40 7.40 7.39 7.39 7.38 7.38 7.33 7.32 7.32 7.31 7.31 7.30 7.30 7.30 7.24 7.23 7.23 7.23 7.23 7.21 4.05 4.04 4.04 4.03 2.93 2.92 2.90 2.89 1.95 1.93 1.92 1.90 1.73 1.19 1.18 0.00
O
Ph–N
4m
3.96 1.00 1.85 2.01 1.94 0.97 1.00 1.00 1.00 3.00 3.00
f1 (ppm)

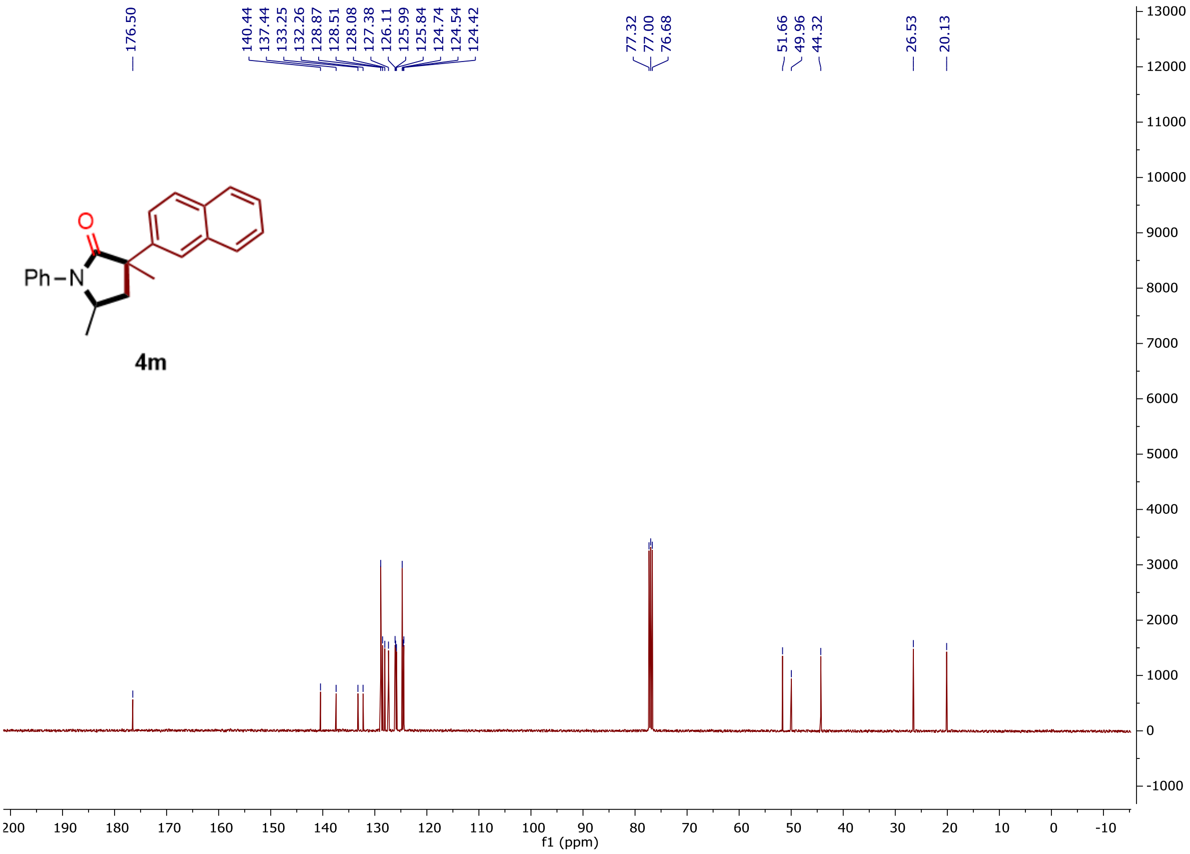
176.50
140.44 137.44 133.25 132.26 128.87 128.51 128.08 127.38 126.11 125.99 125.84 124.74 124.54 124.42
77.32 77.00 76.68
51.66 49.96 44.32
26.53 20.13
O
Ph–N
4m
f1 (ppm)

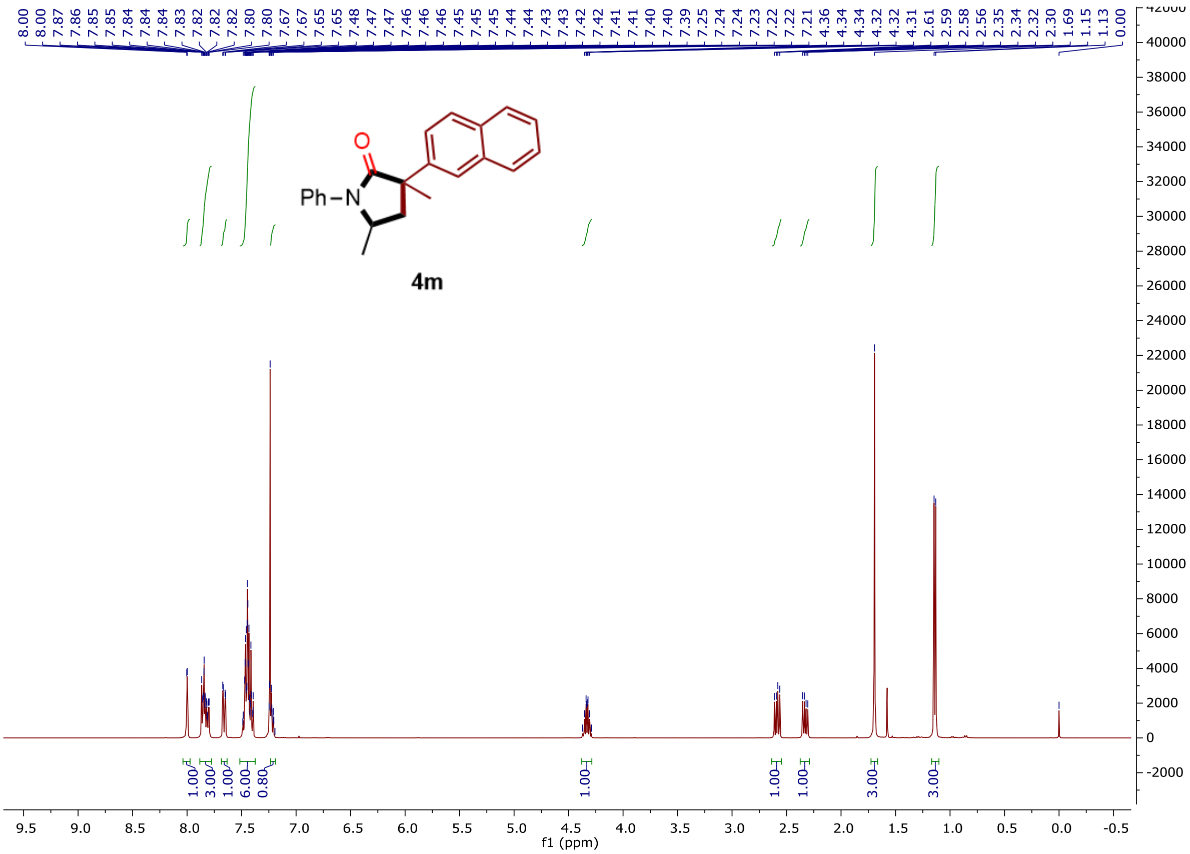
8.00
8.00
7.87
7.86
7.85
7.85
7.84
7.84
7.83
7.82
7.82
7.82
7.80
7.80
7.67
7.65
7.65
7.48
7.47
7.47
7.46
7.46
7.45
7.45
7.45
7.44
7.44
7.43
7.43
7.42
7.42
7.41
7.41
7.40
7.40
7.39
7.25
7.24
7.24
7.23
7.22
7.22
7.21
4.36
4.34
4.34
4.32
4.32
4.31
2.61
2.59
2.58
2.56
2.35
2.34
2.32
2.30
1.69
1.15
1.13
0.00
Ph
N
O
4m
1.00
3.00
1.00
6.00
0.80
1.00
1.00
1.00
3.00
3.00
f1 (ppm)

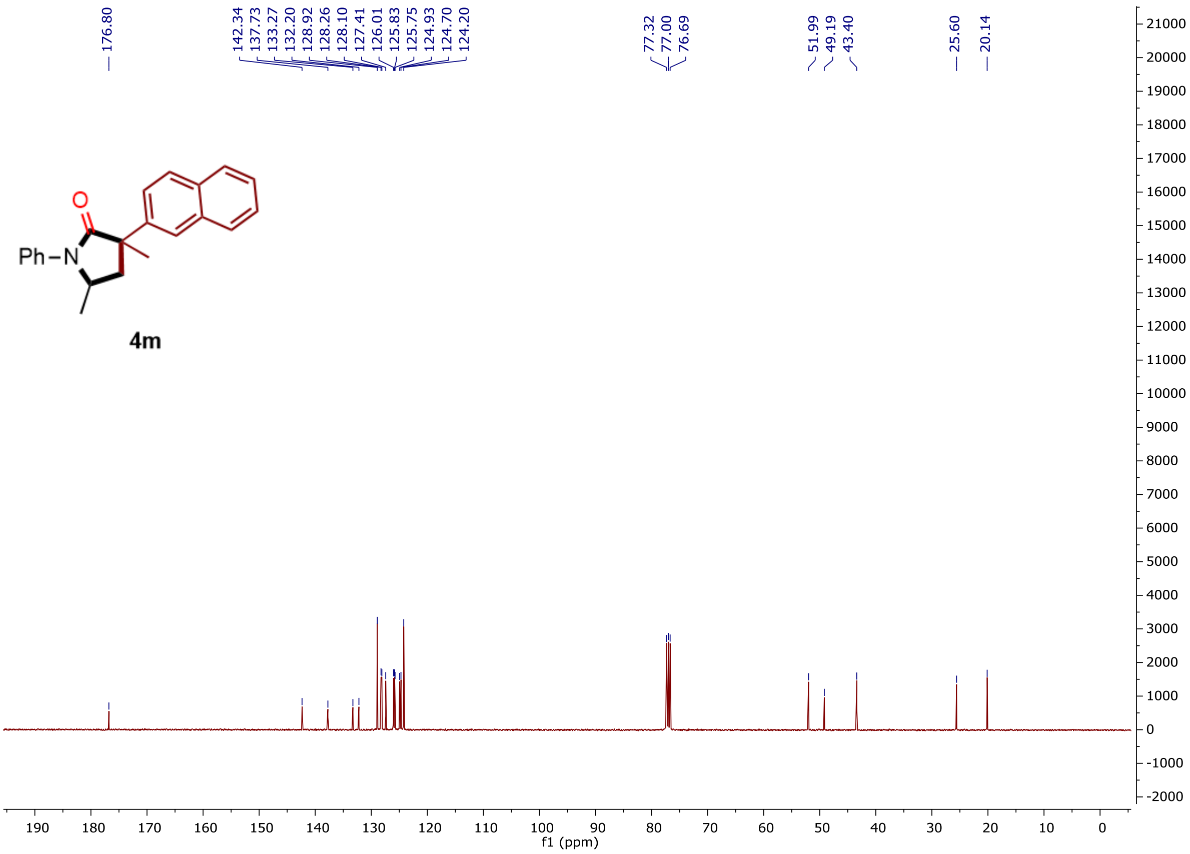
176.80
142.34
137.73
133.27
132.20
128.92
128.26
128.10
127.41
126.01
125.83
125.75
124.93
124.70
124.20
77.32
77.00
76.69
51.99
49.19
43.40
25.60
20.14
Ph
N
O
4m
f1 (ppm)

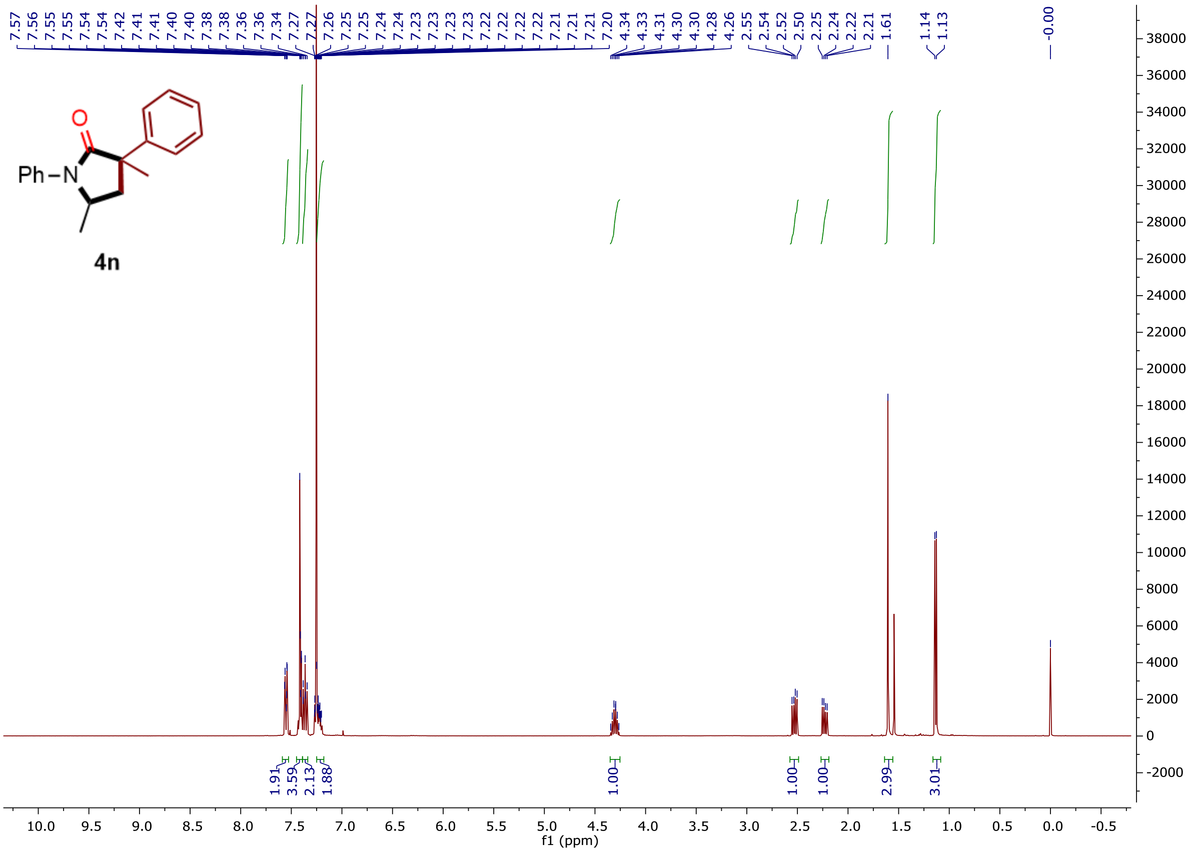

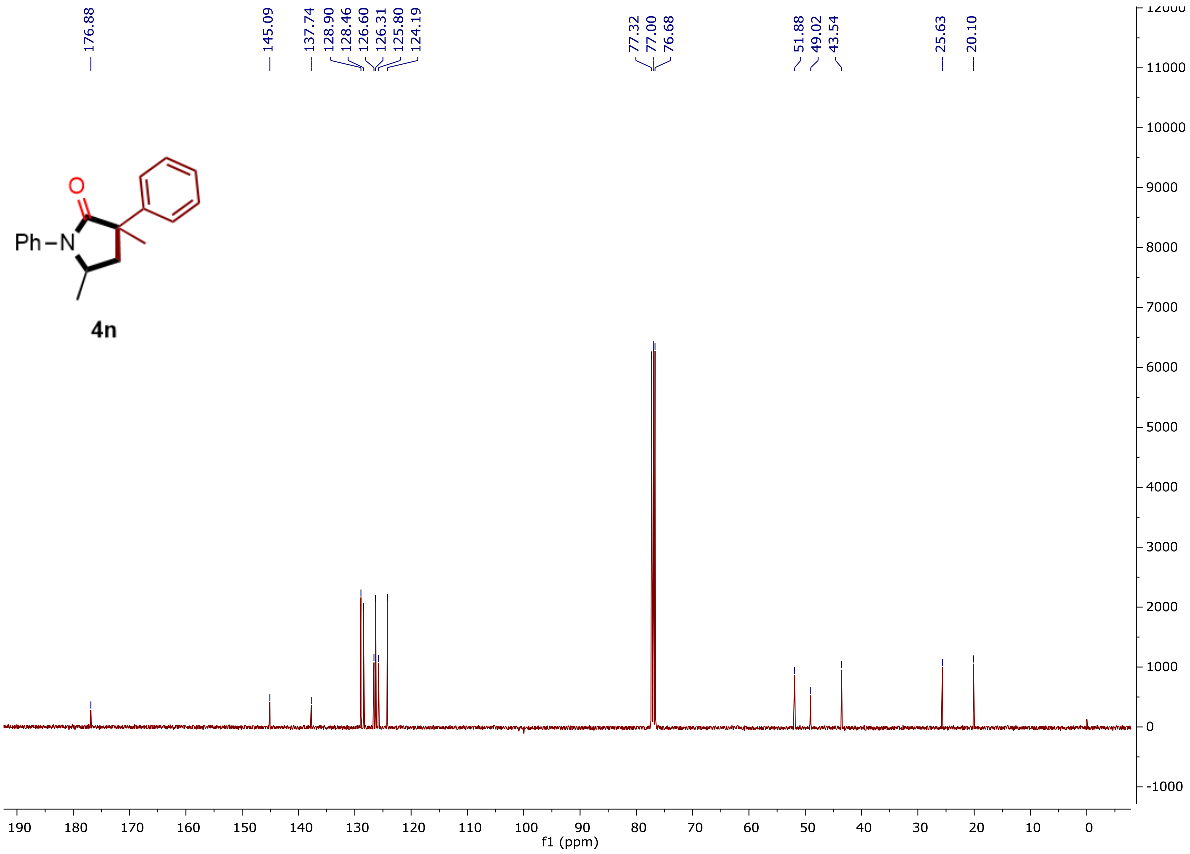

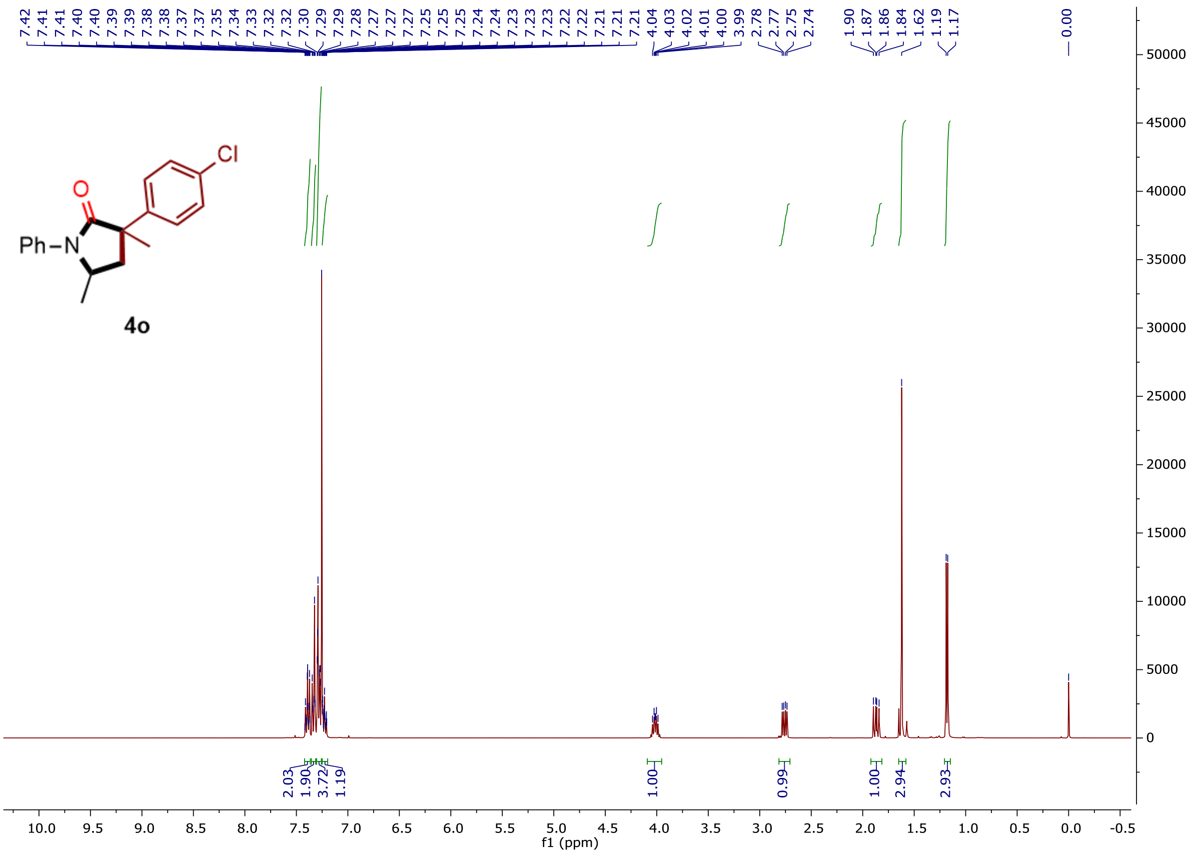

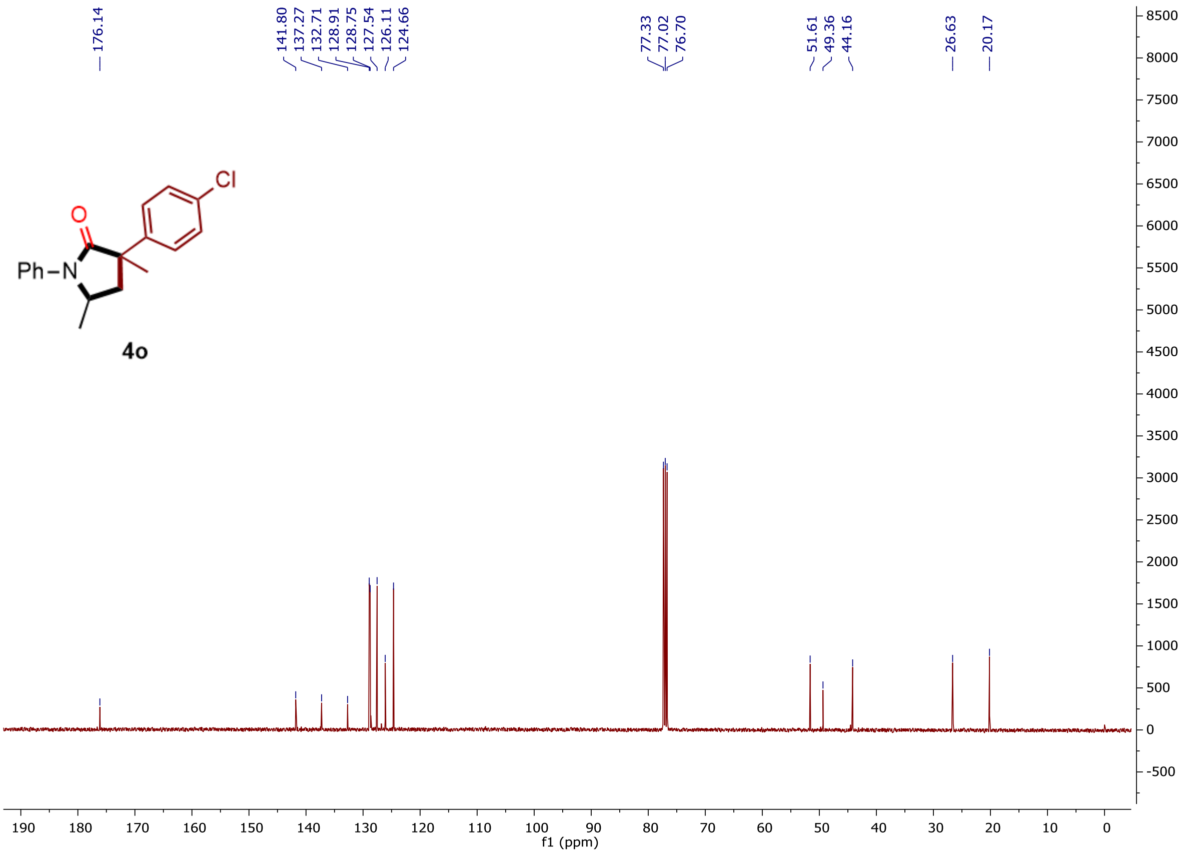

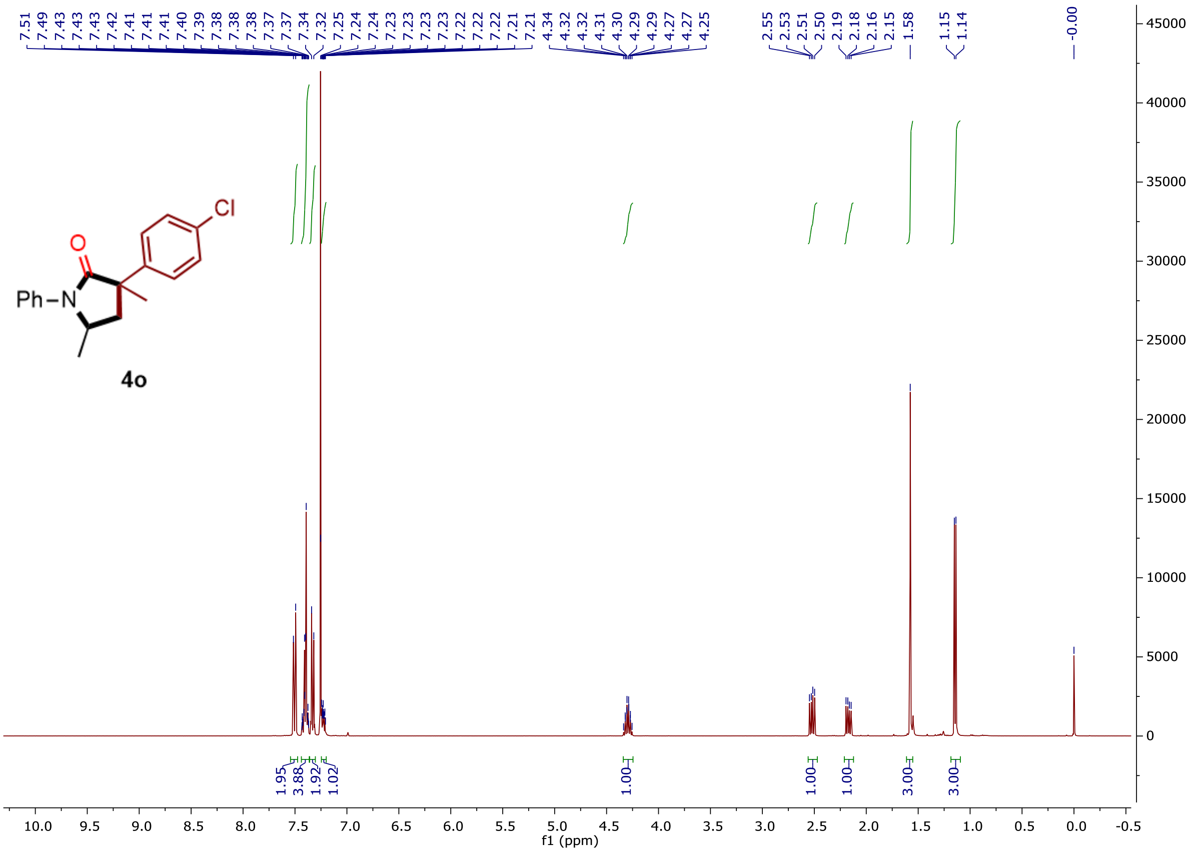
7.51
7.49
7.43
7.43
7.43
7.42
7.41
7.41
7.40
7.39
7.38
7.38
7.38
7.37
7.37
7.34
7.32
7.25
7.24
7.24
7.23
7.23
7.23
7.23
7.22
7.22
7.22
7.21
7.21
4.34
4.32
4.32
4.31
4.30
4.29
4.29
4.27
4.27
4.25
2.55
2.53
2.51
2.50
2.19
2.18
2.16
2.15
1.58
1.15
1.14
-0.00
Cl
O
Ph–N
1.95
3.88
1.92
1.02
1.00
1.00
1.00
3.00
3.00
f1 (ppm)

4o

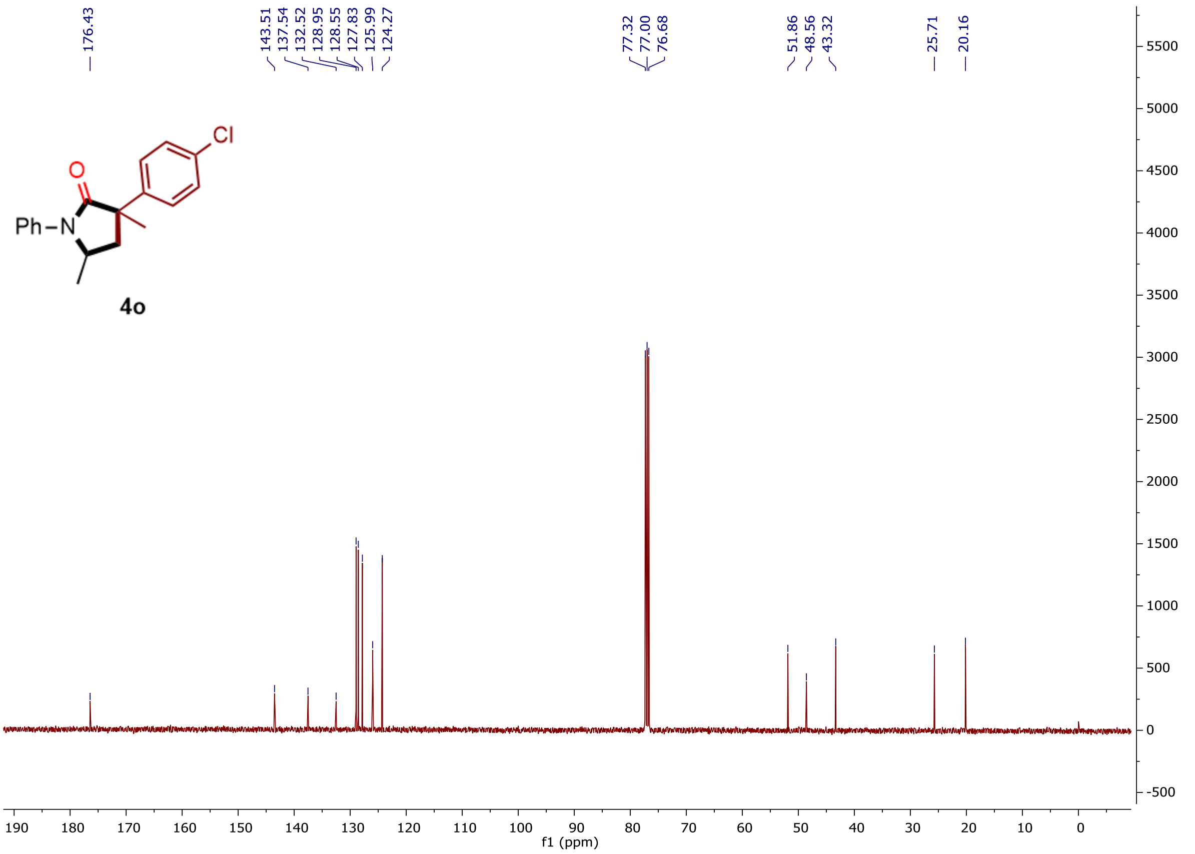
176.43
143.51
137.54
132.52
128.95
128.55
127.83
125.99
124.27
77.32
77.00
76.68
51.86
48.56
43.32
25.71
20.16
Cl
O
Ph–N
f1 (ppm)

4o

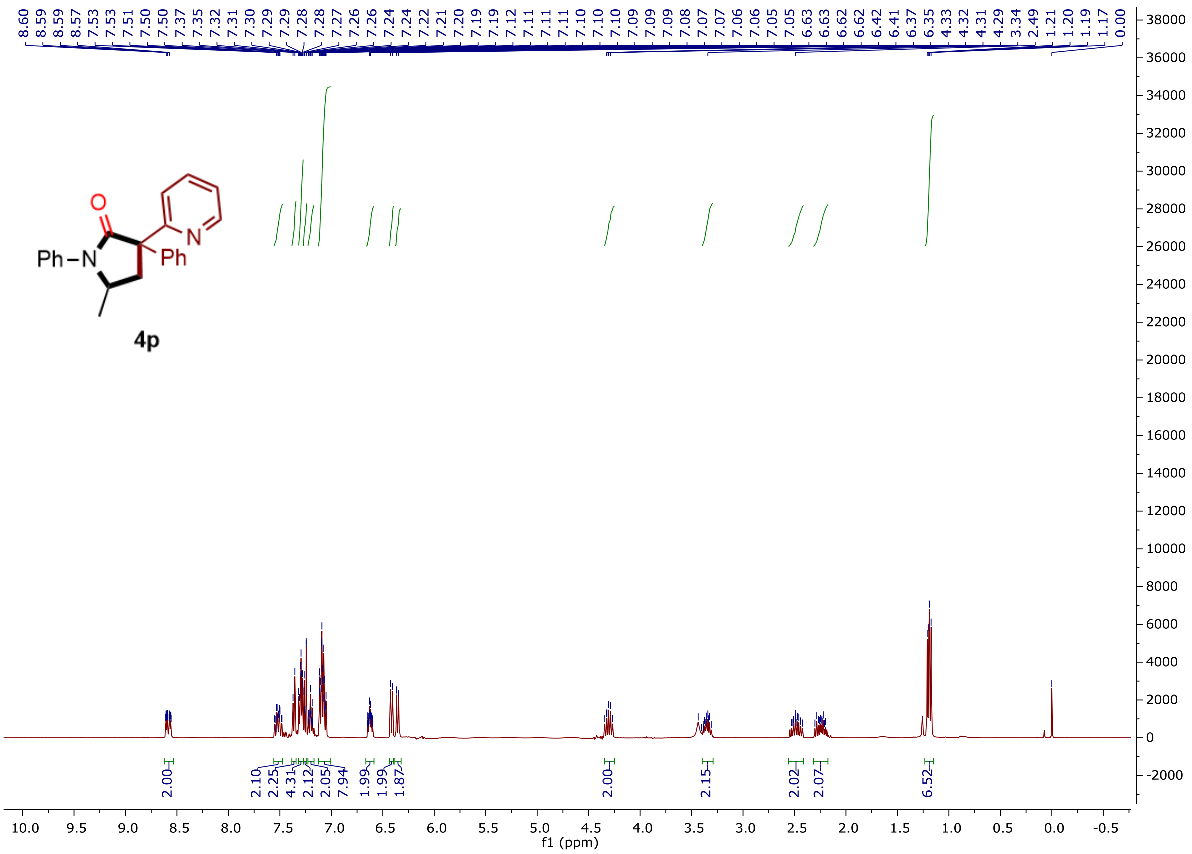
8.60 8.59 8.59 8.57 7.53 7.53 7.51 7.50 7.50 7.37 7.35 7.32 7.31 7.30 7.29 7.29 7.28 7.28 7.27 7.26 7.26 7.24 7.24 7.22 7.21 7.20 7.19 7.19 7.12 7.11 7.11 7.11 7.10 7.10 7.09 7.09 7.09 7.08 7.07 7.07 7.06 7.05 7.05 6.63 6.63 6.62 6.62 6.42 6.41 6.37 6.35 4.33 4.32 4.31 4.29 3.34 2.49 1.21 1.20 1.19 1.17 0.00
O
N
Ph–N
Ph
4p
2.00 2.10 2.25 4.31 2.12 2.05 7.94 1.99 1.99 1.87 2.00 2.15 2.02 2.07 6.52
f1 (ppm)

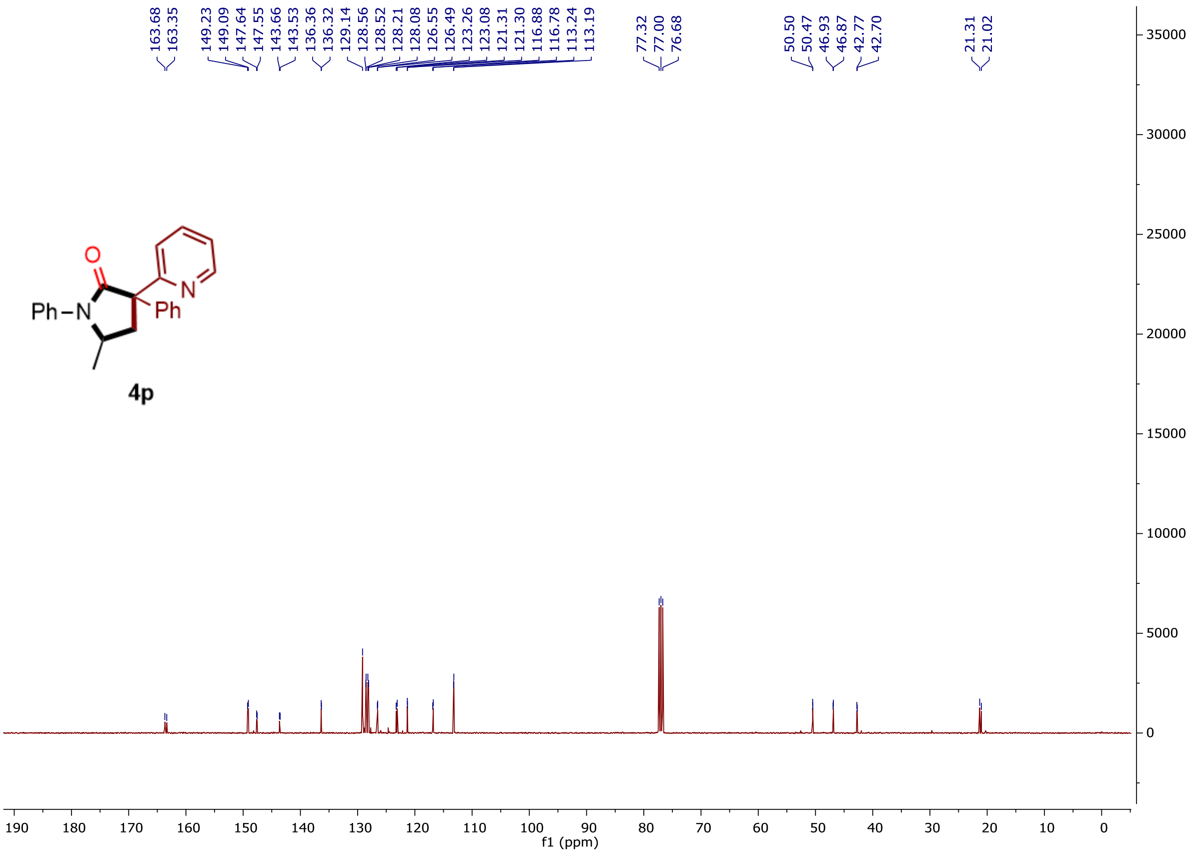
163.68 163.35 149.23 149.09 147.64 147.55 143.66 143.53 136.36 136.32 129.14 128.56 128.52 128.21 128.08 126.55 126.49 123.26 123.08 121.31 121.30 116.88 116.78 113.24 113.19 77.32 77.00 76.68 50.50 50.47 46.93 46.87 42.77 42.70 21.31 21.02
O
N
Ph–N
Ph
4p
f1 (ppm)

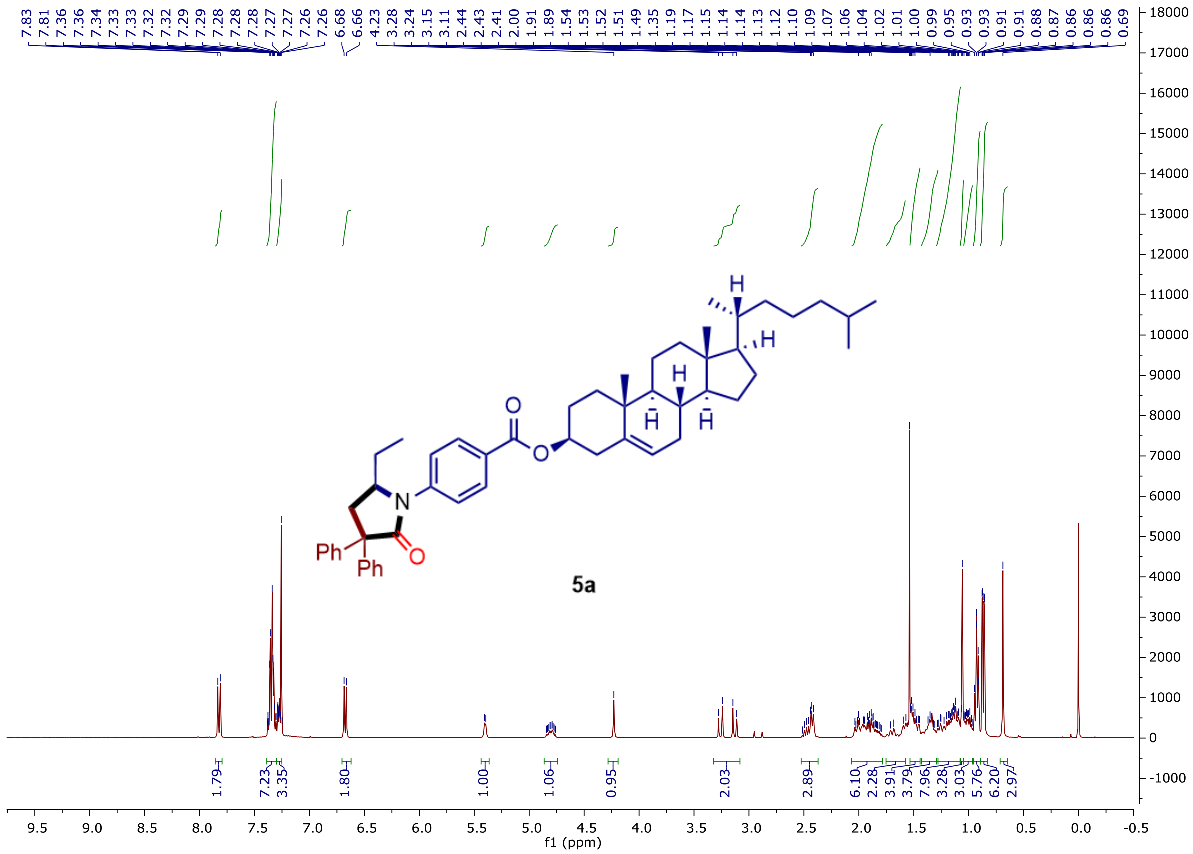

5a
f1 (ppm)

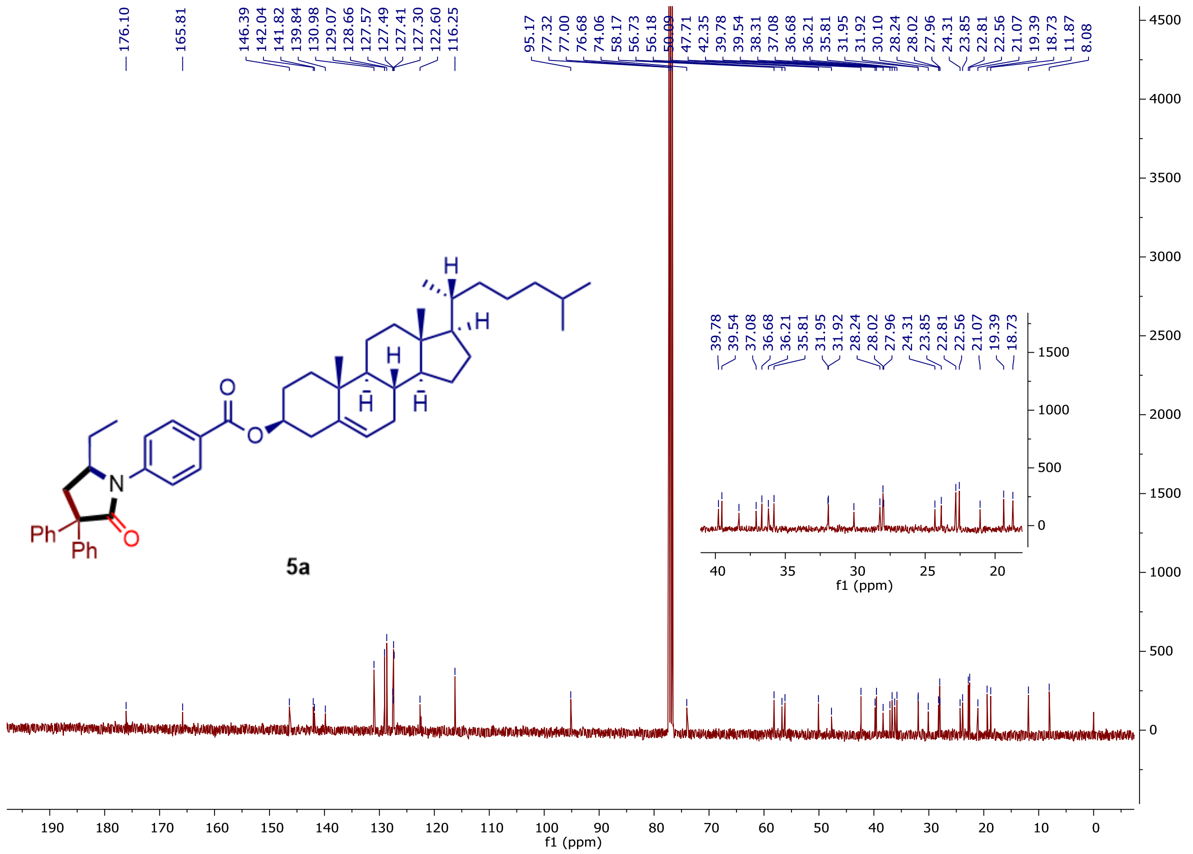

5a
f1 (ppm)

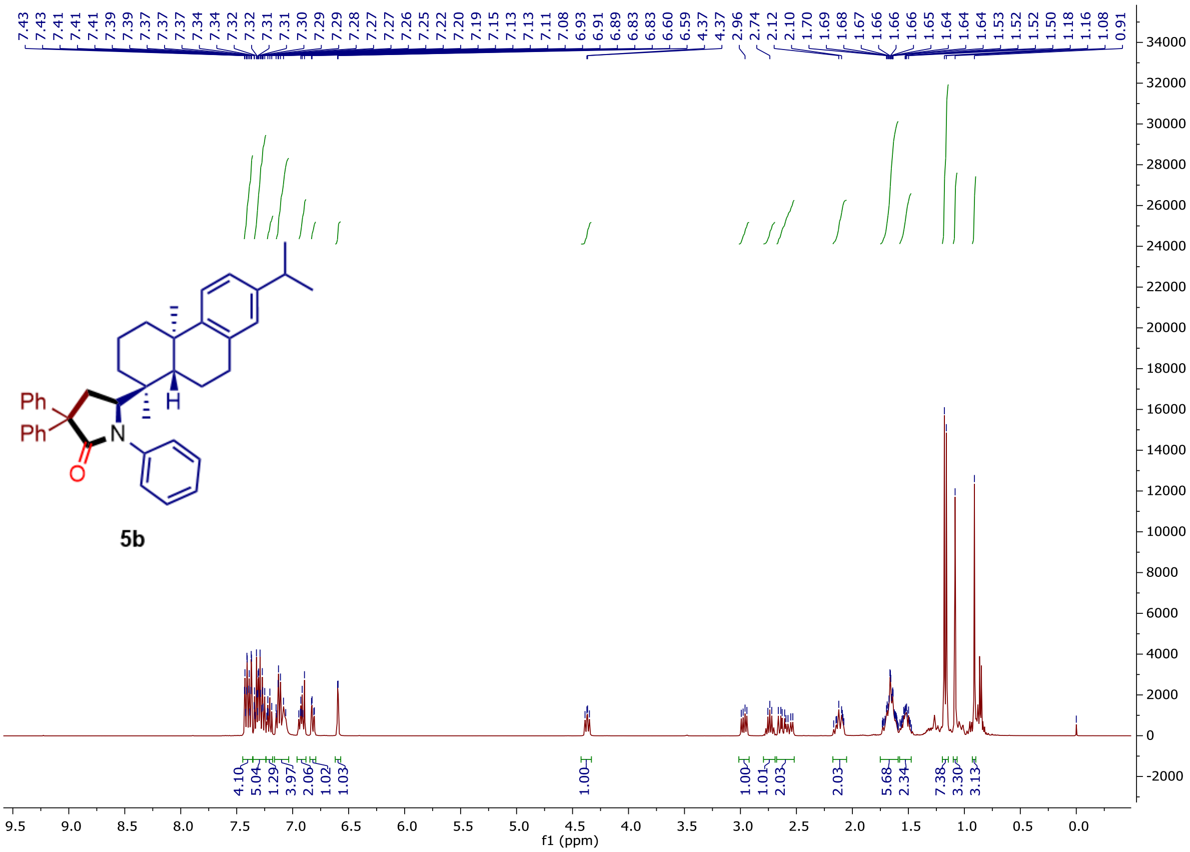

5b

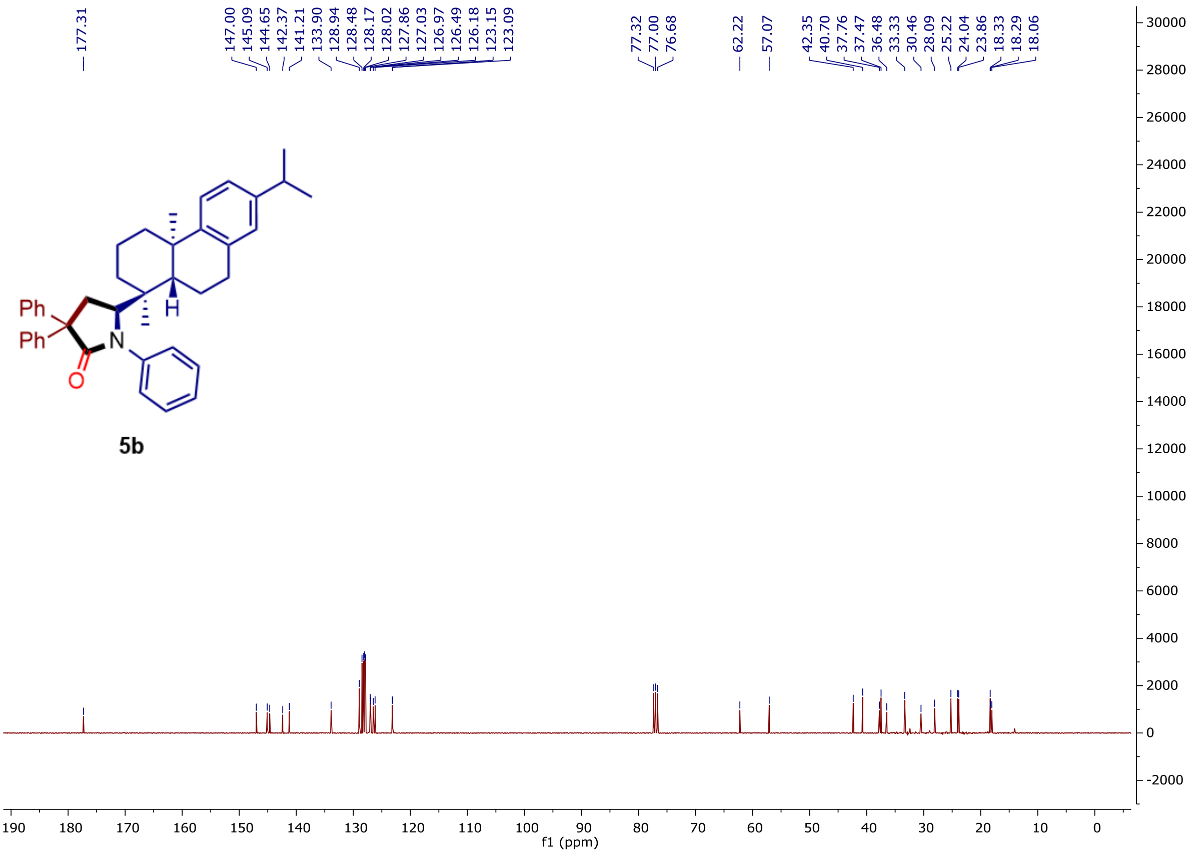

5b

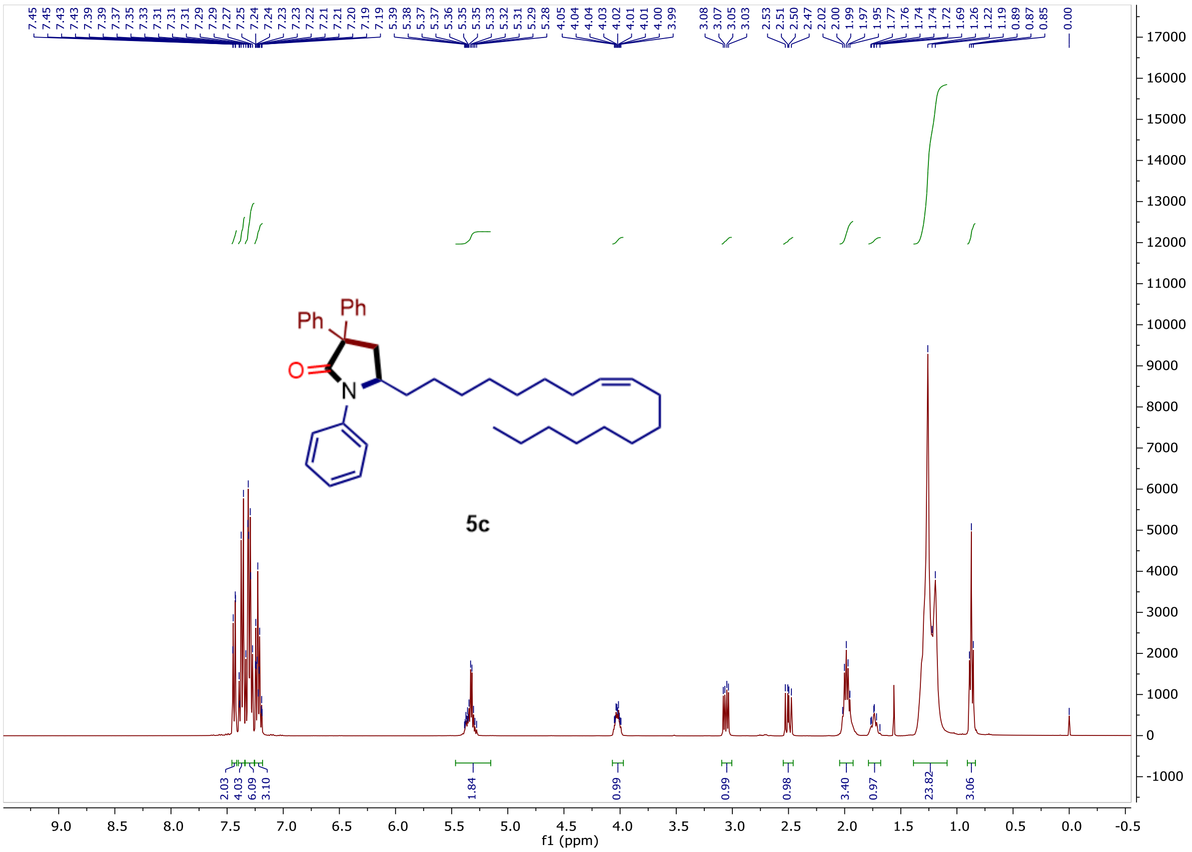
7.45 7.45 7.43 7.39 7.39 7.37 7.35 7.33 7.31 7.31 7.31 7.29 7.29 7.27 7.25 7.24 7.24 7.23 7.23 7.22 7.21 7.21 7.20 7.19 7.19 5.39 5.38 5.37 5.37 5.36 5.35 5.35 5.33 5.32 5.31 5.29 5.28 4.05 4.04 4.03 4.02 4.01 4.01 4.00 3.99 3.08 3.07 3.05 3.03 2.53 2.51 2.50 2.47 2.02 2.00 1.99 1.97 1.95 1.77 1.76 1.74 1.74 1.72 1.69 1.26 1.22 1.19 0.89 0.87 0.85 0.00
Ph
Ph
O
N
5c
2.03 4.03 6.09 3.10 1.84 0.99 0.99 0.98 3.40 0.97 23.82 3.06
f1 (ppm)

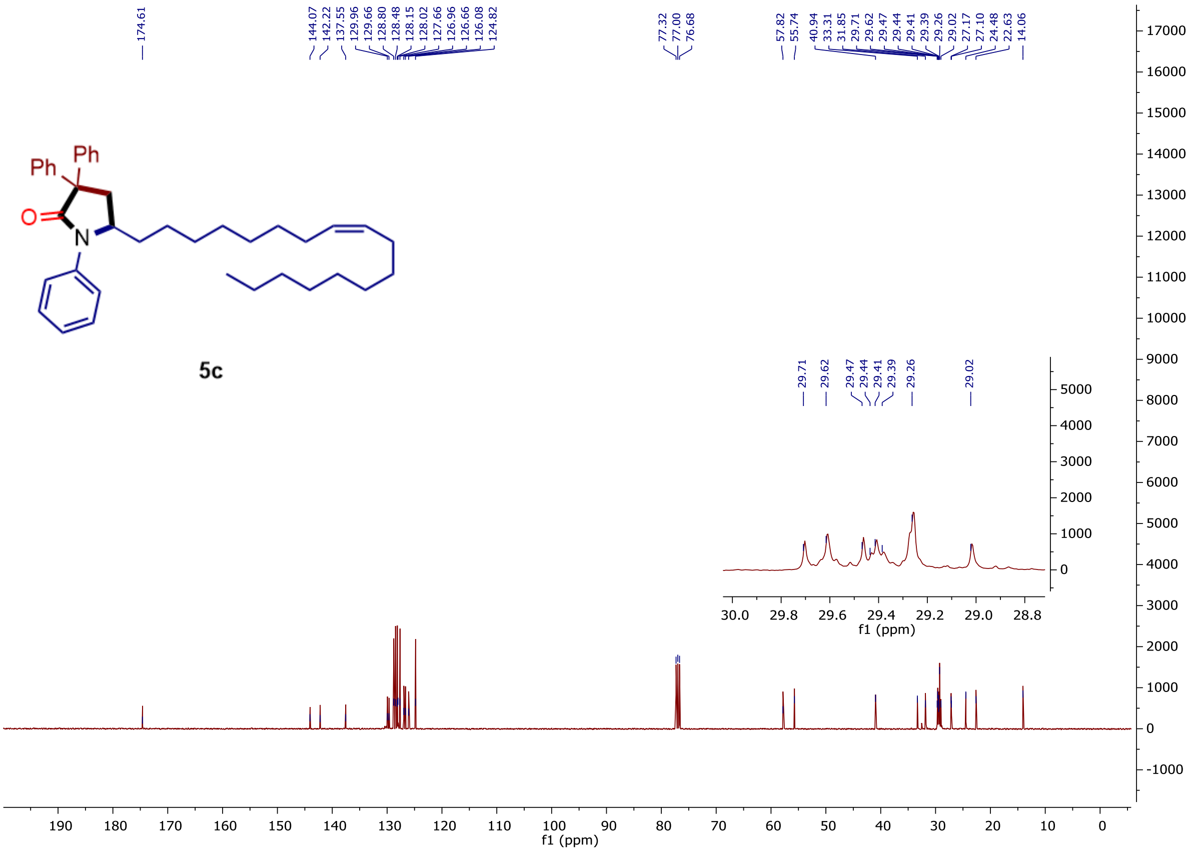
174.61
144.07 142.22 137.55 129.96 129.66 128.80 128.48 128.15 128.02 127.66 126.96 126.66 126.08 124.82
77.32 77.00 76.68
57.82 55.74 40.94 33.31 31.85 29.71 29.62 29.47 29.44 29.41 29.39 29.26 29.02 27.17 27.10 24.48 22.63 14.06
Ph
Ph
O
N
5c
29.71 29.62 29.47 29.44 29.41 29.39 29.26 29.02
f1 (ppm)
f1 (ppm)

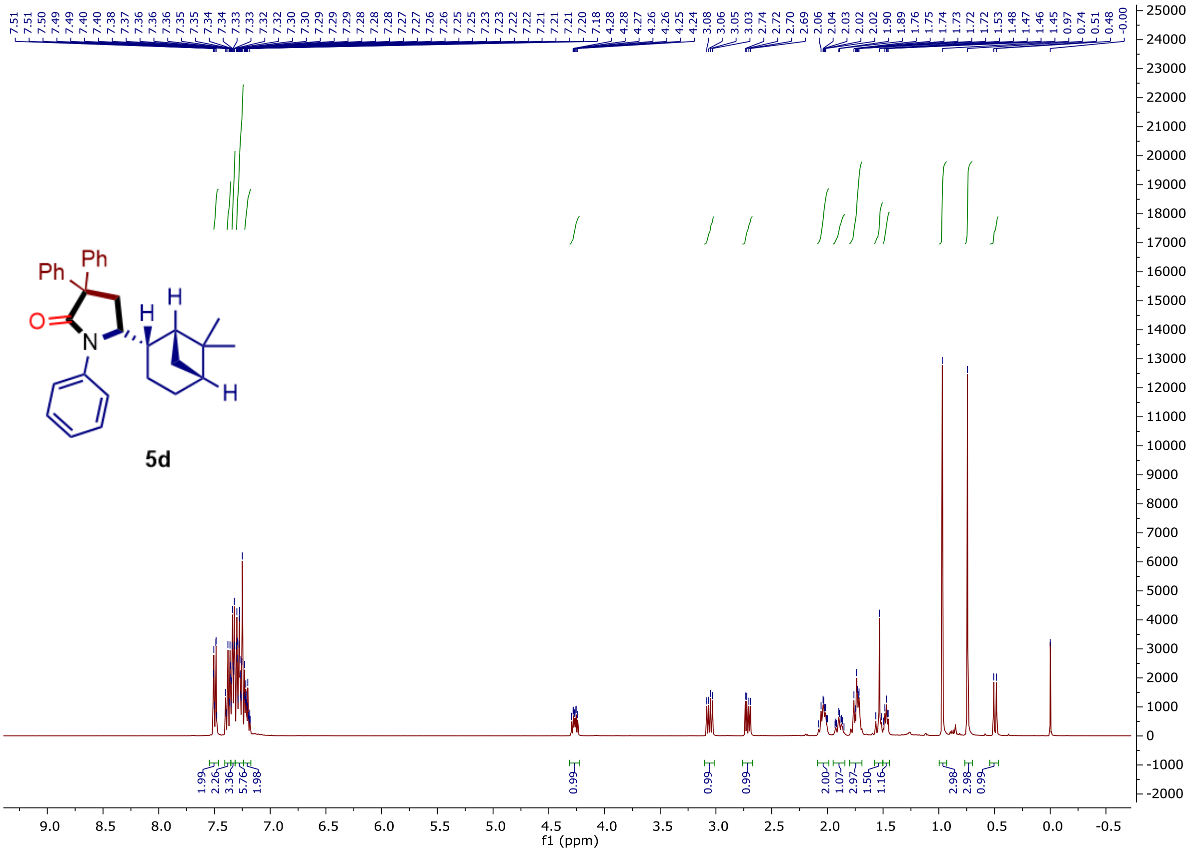
Ph
Ph
O
N
H
H
H
5d
f1 (ppm)

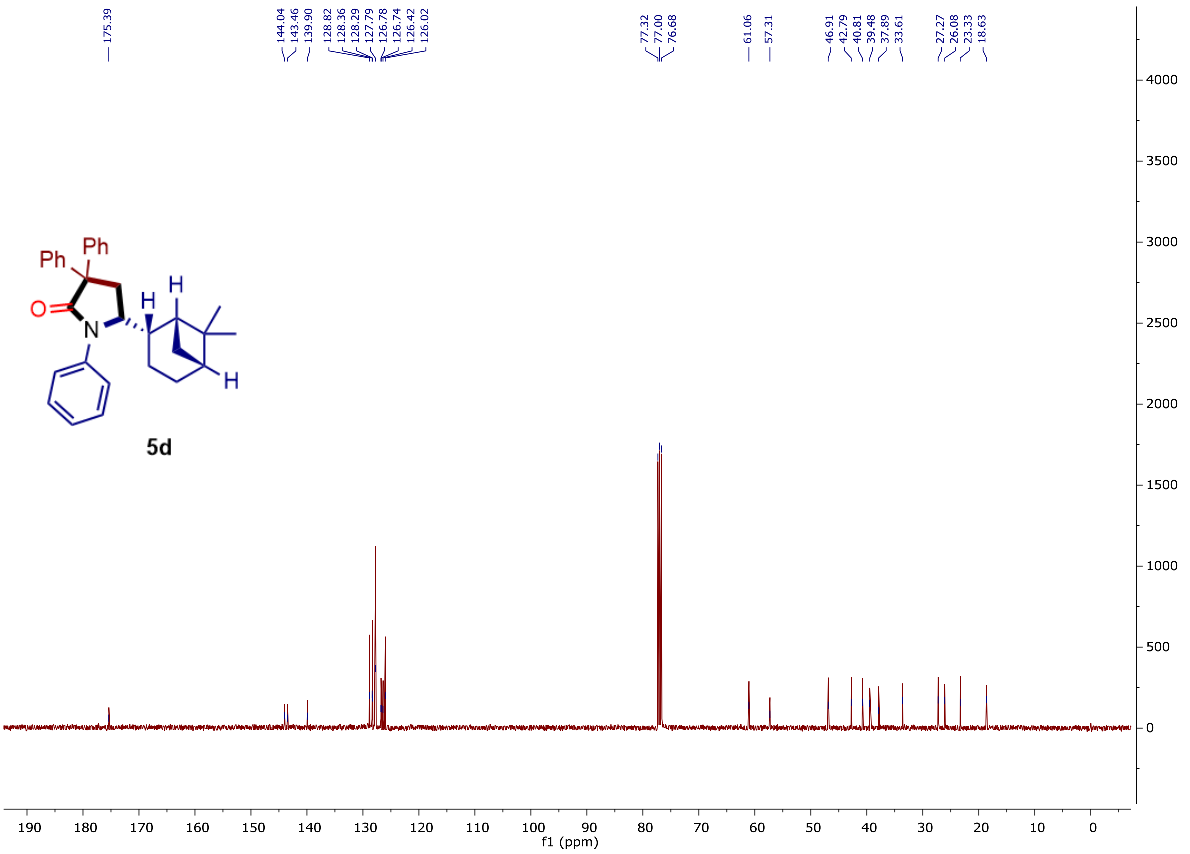
175.39
144.04
143.46
139.90
128.82
128.36
128.29
127.79
126.78
126.74
126.42
126.02
77.32
77.00
76.68
61.06
57.31
46.91
42.79
40.81
39.48
37.89
33.61
27.27
26.08
23.33
18.63
Ph
Ph
O
N
H
H
H
5d
f1 (ppm)

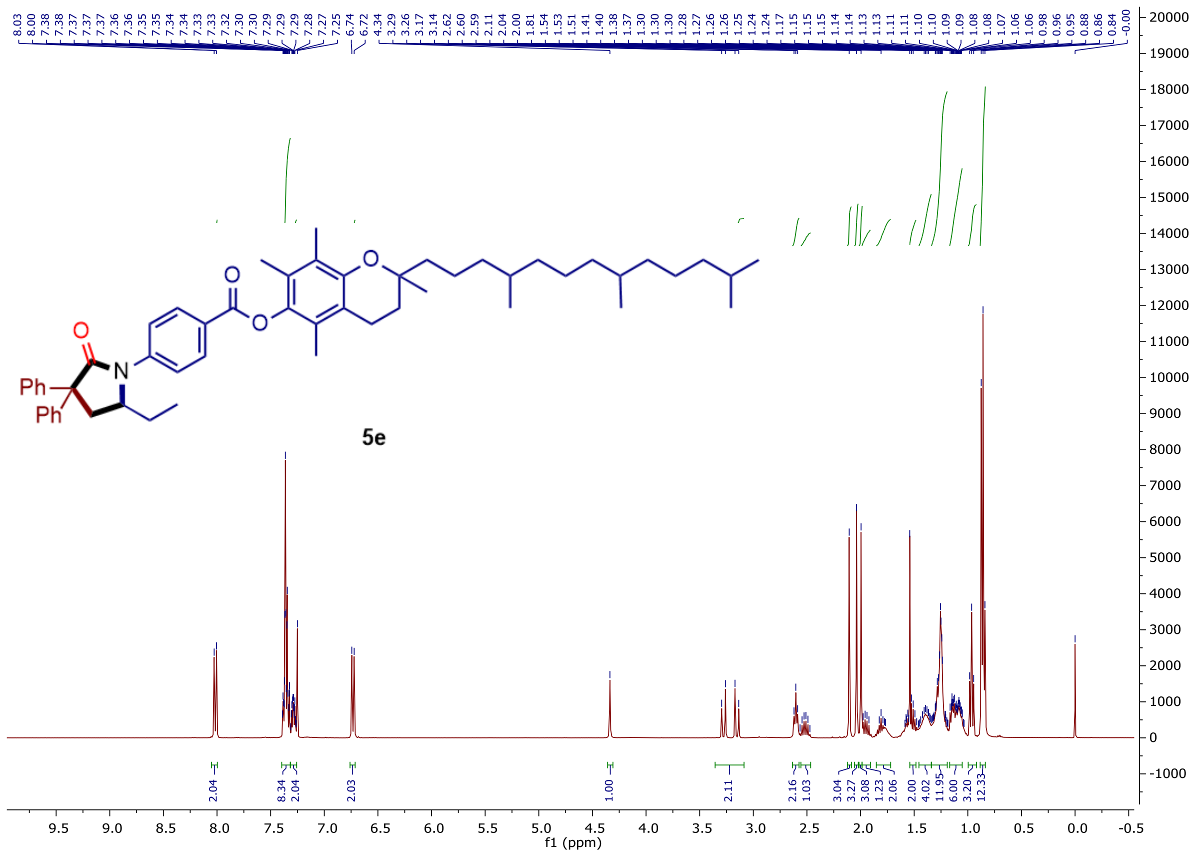

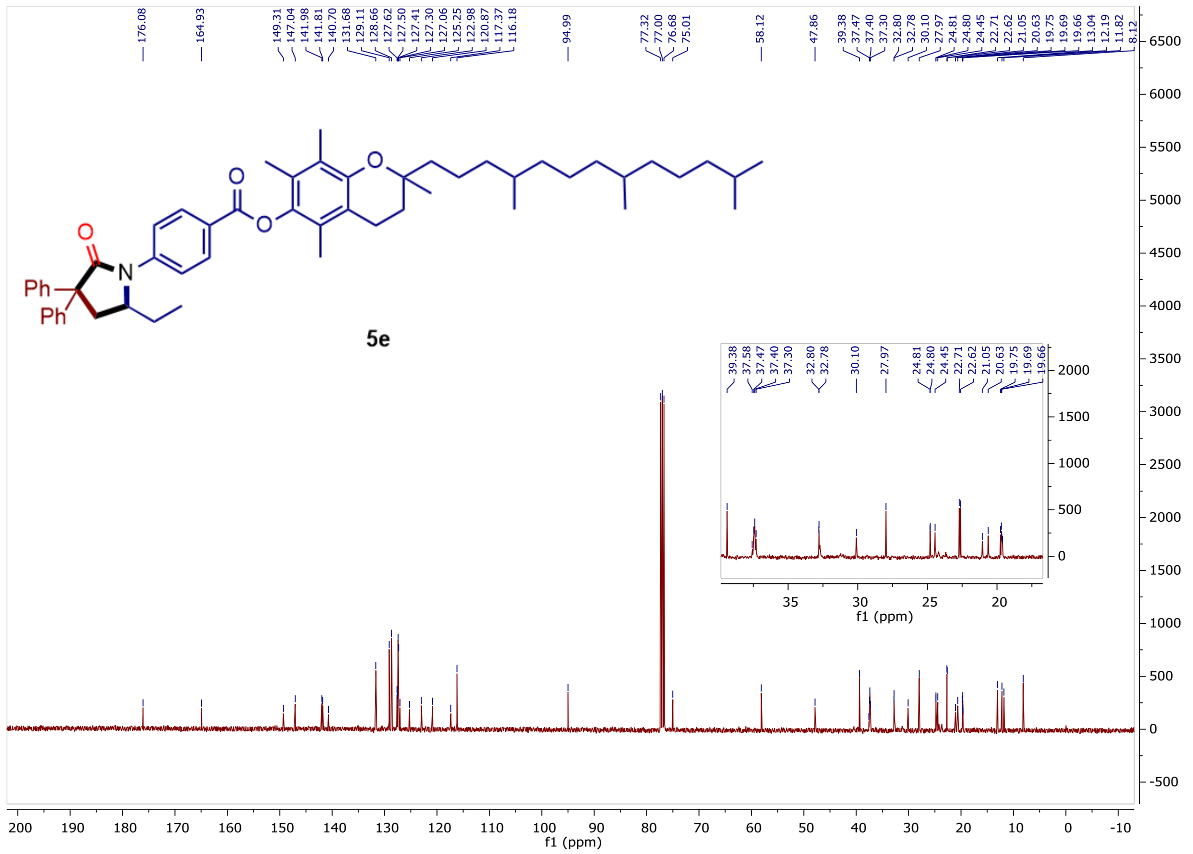

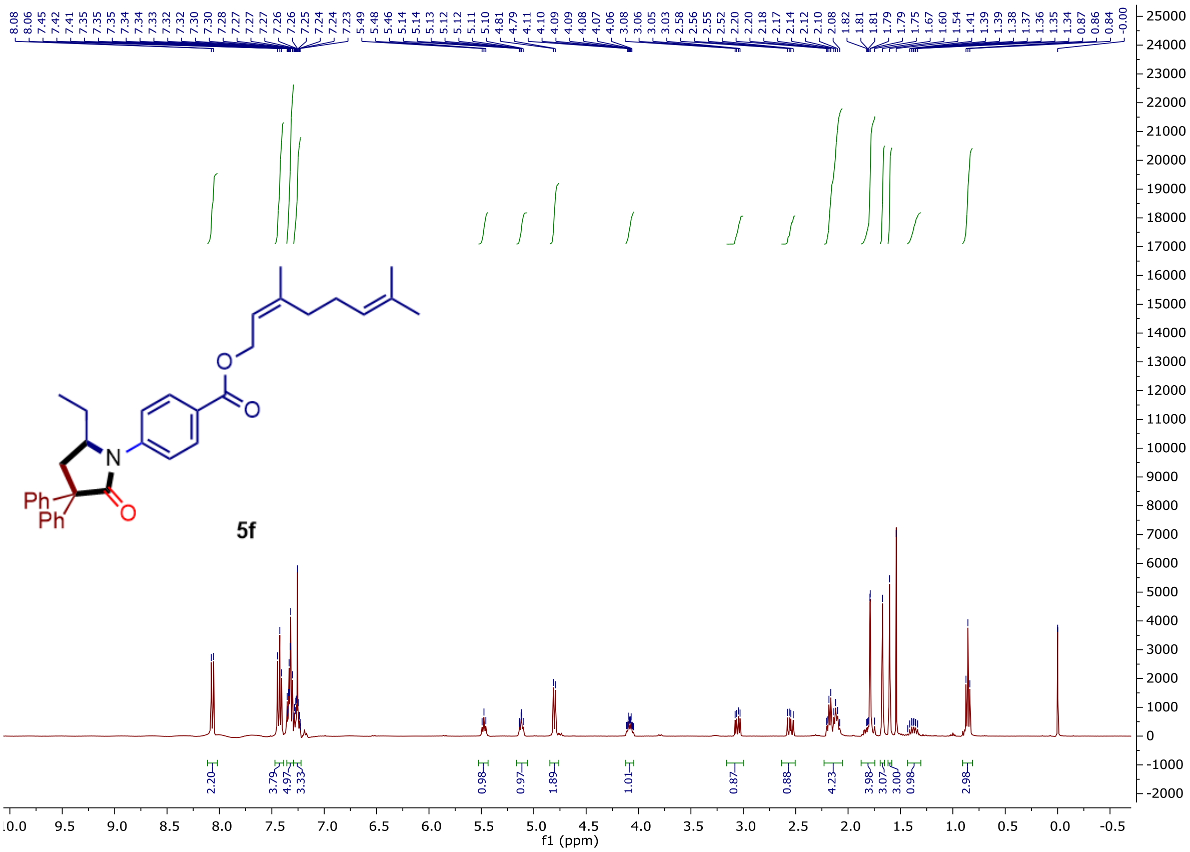
5f
f1 (ppm)

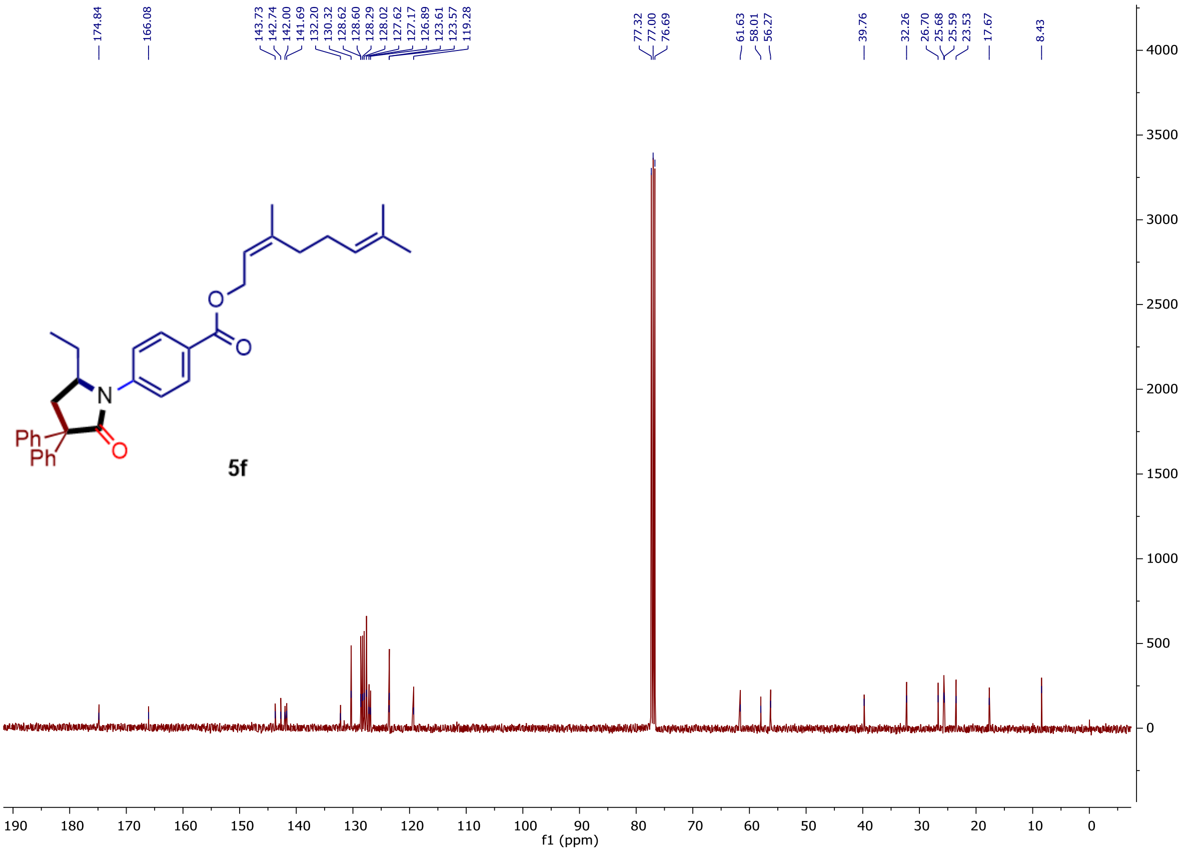
5f
f1 (ppm)

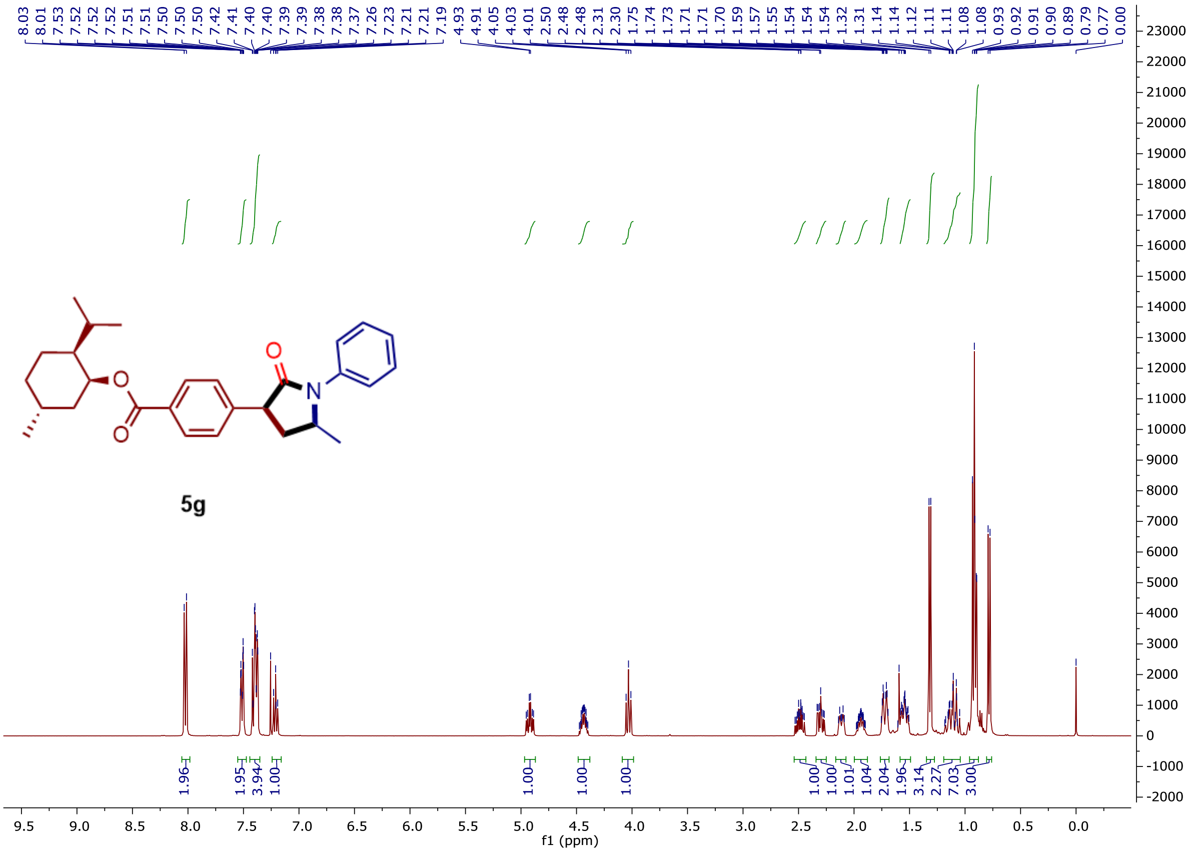

5g
f1 (ppm)

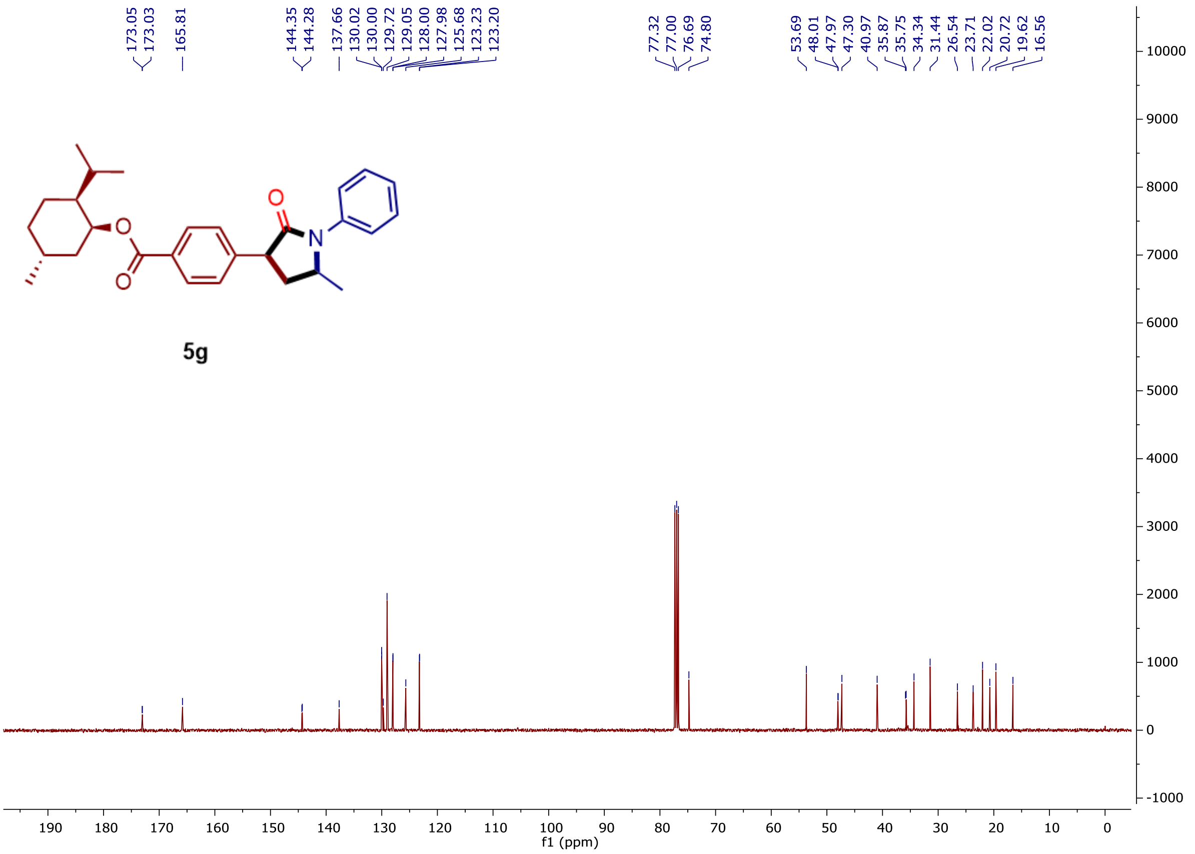

5g
f1 (ppm)

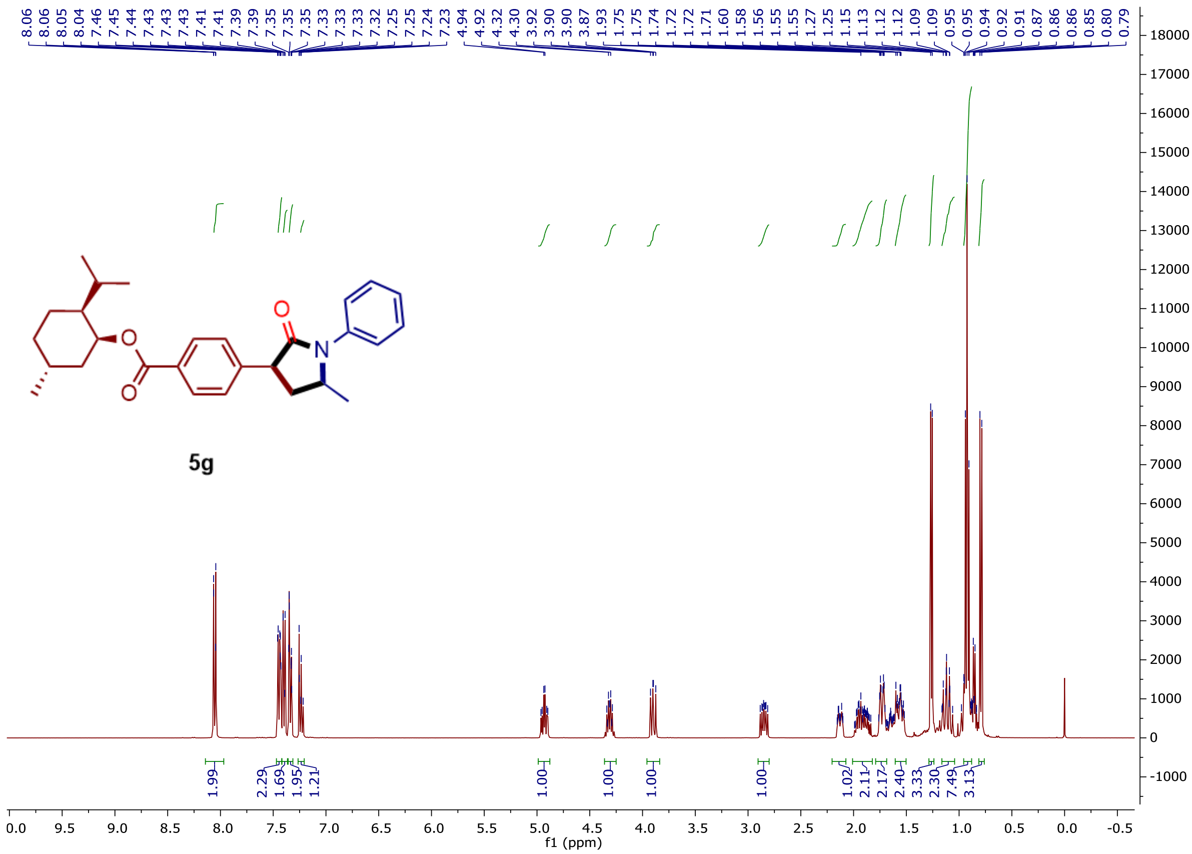
5g
f1 (ppm)

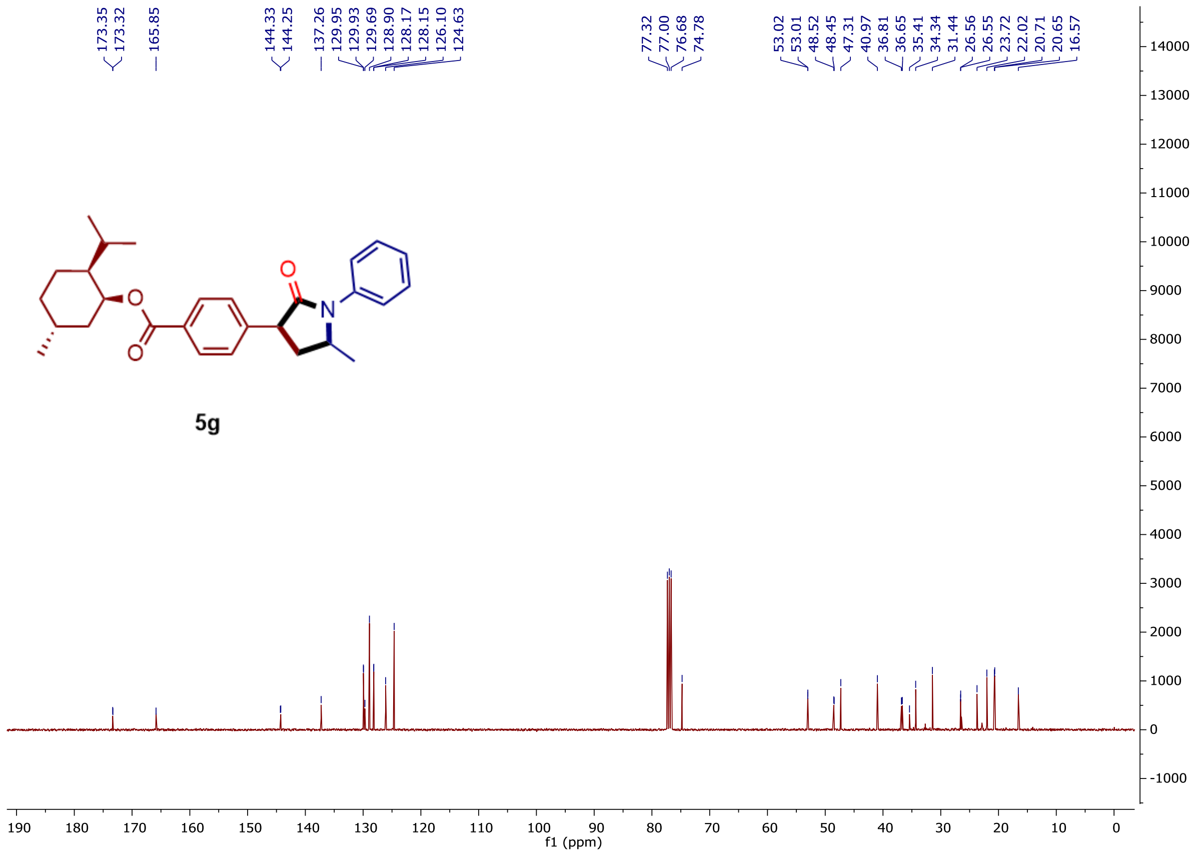
5g
f1 (ppm)

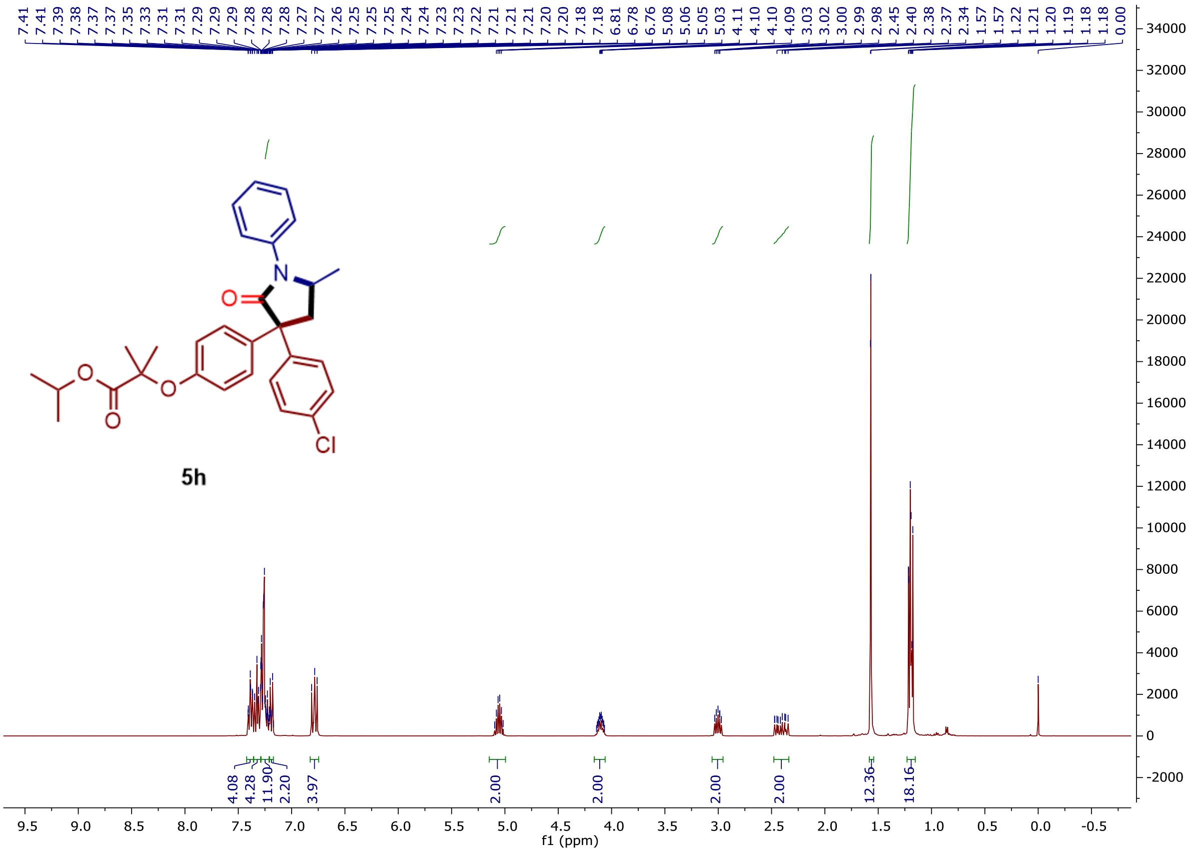
7.41 7.41 7.39 7.38 7.37 7.37 7.35 7.33 7.31 7.31 7.29 7.29 7.29 7.28 7.28 7.28 7.27 7.27 7.26 7.25 7.25 7.25 7.24 7.24 7.23 7.23 7.22 7.21 7.21 7.21 7.20 7.20 7.18 7.18 6.81 6.78 6.76 5.08 5.06 5.05 5.03 4.11 4.10 4.09 3.03 3.02 3.00 2.99 2.98 2.45 2.40 2.38 2.37 2.34 1.57 1.57 1.22 1.21 1.20 1.19 1.18 1.18 0.00
O
N
O
O
O
Cl
5h
4.08 4.28 11.90 2.20 3.97 2.00 2.00 2.00 2.00 12.36 18.16
f1 (ppm)

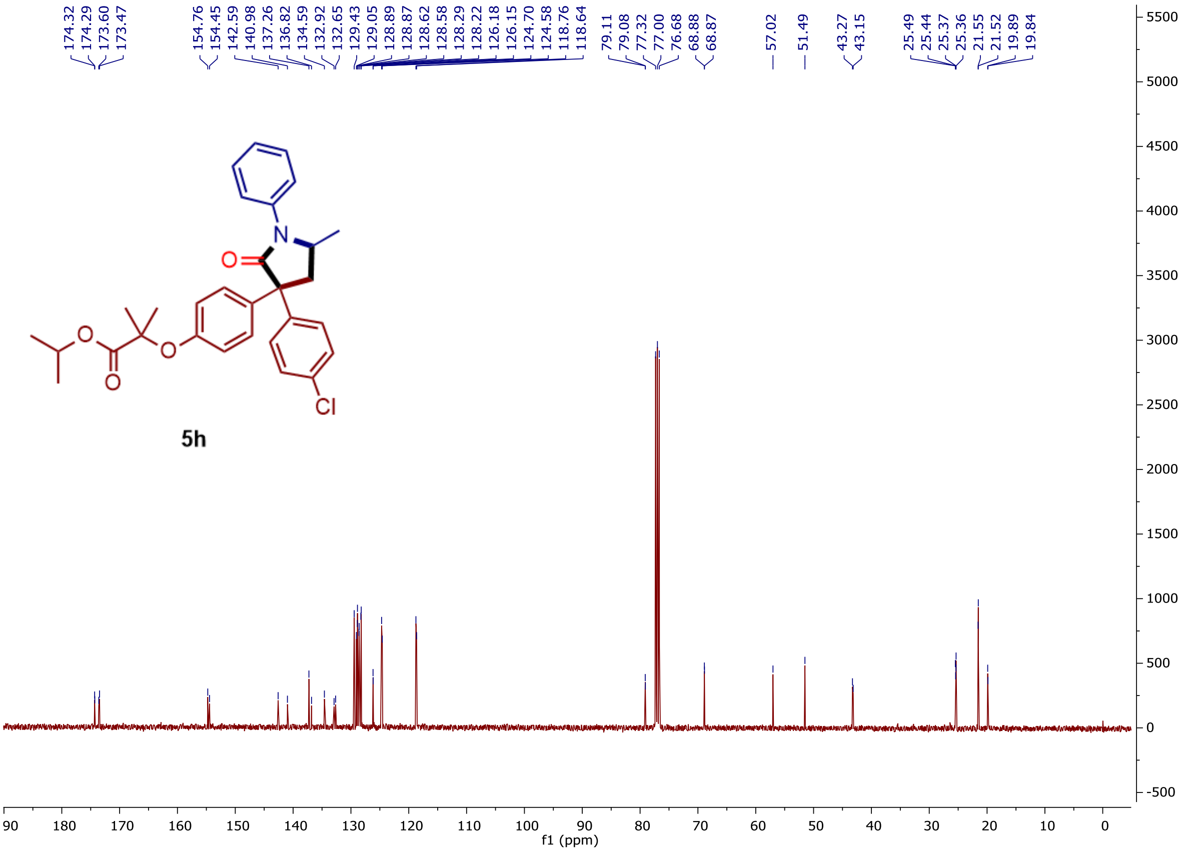
174.32 174.29 173.60 173.47 154.76 154.45 142.59 140.98 137.26 136.82 134.59 132.92 132.65 129.43 129.05 128.89 128.87 128.62 128.58 128.29 128.22 126.18 126.15 124.70 124.58 118.76 118.64 79.11 79.08 77.32 77.00 76.68 68.88 68.87 57.02 51.49 43.27 43.15 25.49 25.44 25.37 25.36 21.55 21.52 19.89 19.84
O
N
O
O
O
Cl
5h
f1 (ppm)

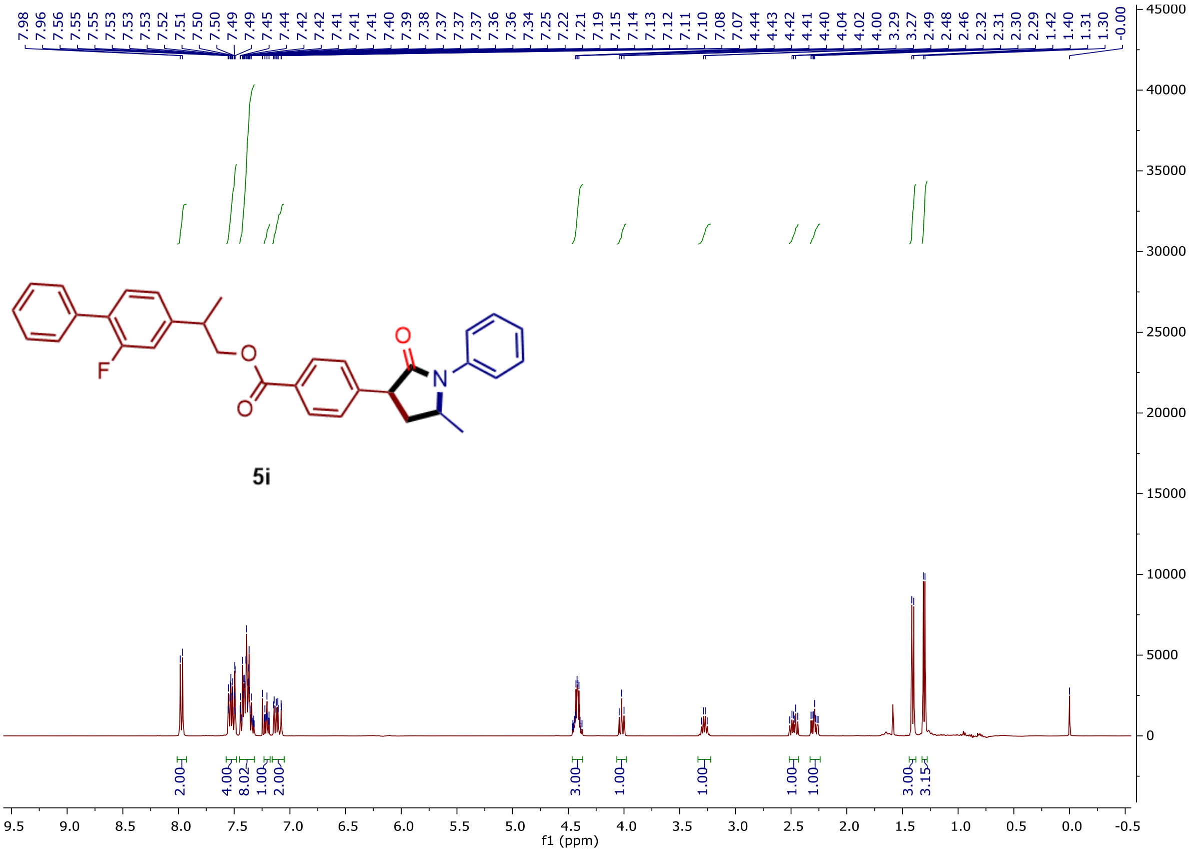
7.98 7.96 7.56 7.55 7.55 7.53 7.53 7.53 7.52 7.51 7.50 7.50 7.49 7.49 7.45 7.44 7.42 7.42 7.41 7.41 7.41 7.40 7.39 7.38 7.37 7.37 7.37 7.36 7.36 7.34 7.25 7.22 7.21 7.19 7.15 7.14 7.13 7.12 7.11 7.10 7.08 7.07 4.44 4.43 4.42 4.41 4.40 4.04 4.02 4.00 3.29 3.27 2.49 2.48 2.46 2.32 2.31 2.30 2.29 1.42 1.40 1.31 1.30 -0.00
F
O
O
O
N
5i
2.00 4.00 8.02 1.00 2.00 3.00 1.00 1.00 1.00 1.00 3.00 3.15
f1 (ppm)

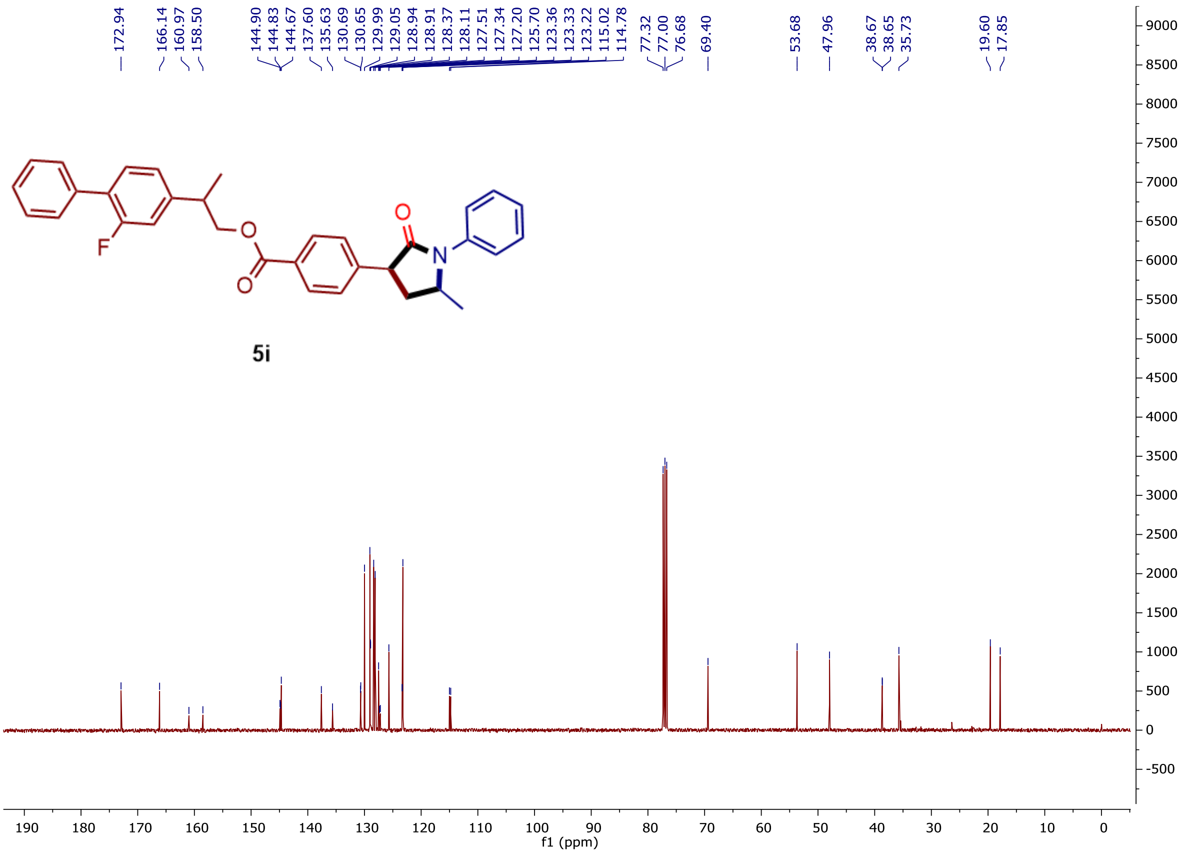
172.94 166.14 160.97 158.50 144.90 144.83 144.67 137.60 135.63 130.69 130.65 129.99 129.05 128.94 128.91 128.37 128.11 127.51 127.34 127.20 125.70 123.36 123.33 123.22 115.02 114.78 77.32 77.00 76.68 69.40 53.68 47.96 38.67 38.65 35.73 19.60 17.85
F
O
O
O
N
5i
f1 (ppm)

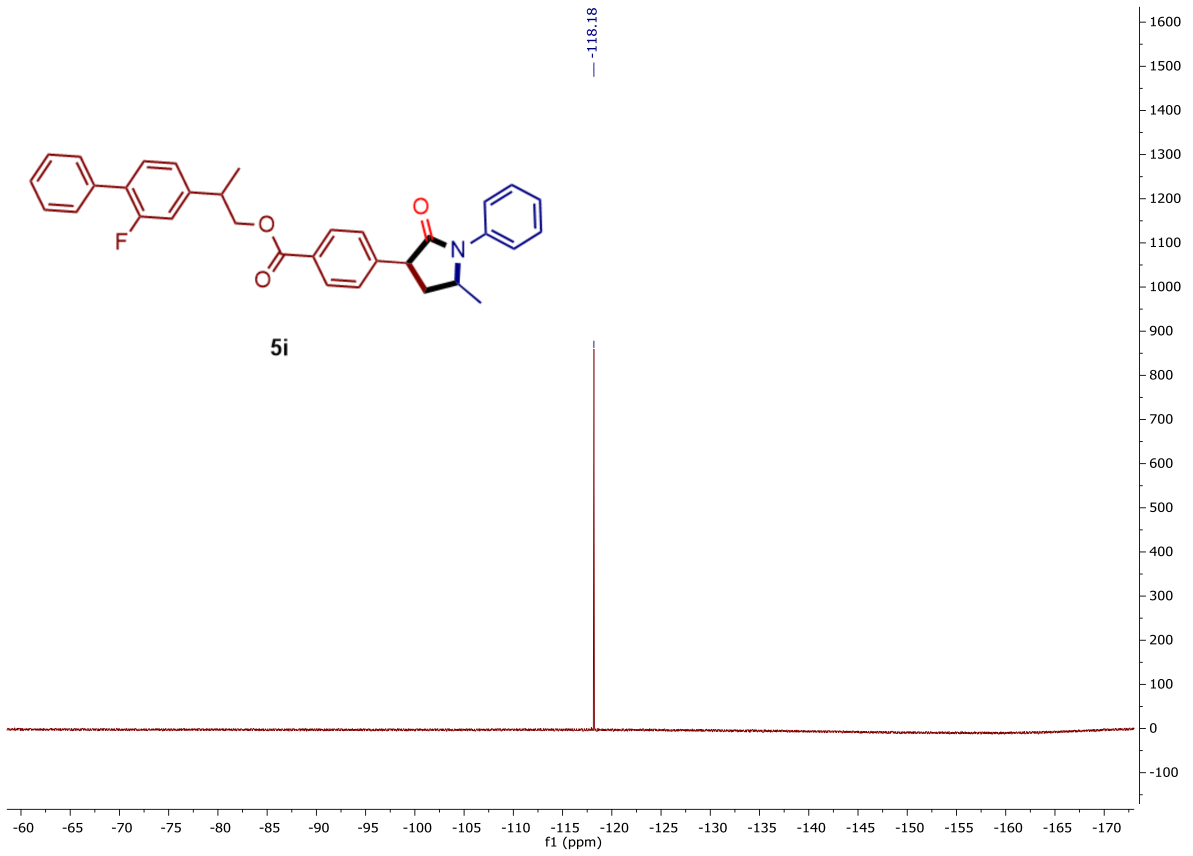

-118.18
5i
f1 (ppm)

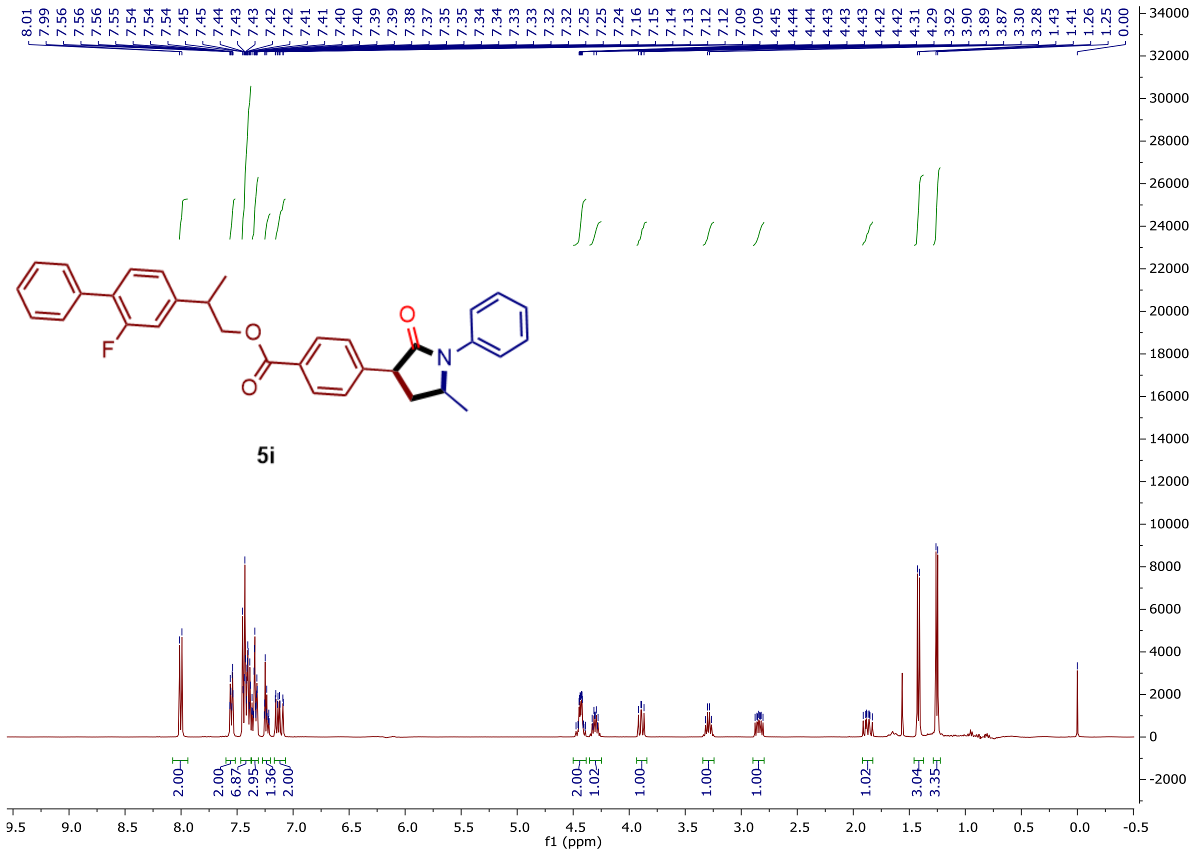

5i
f1 (ppm)

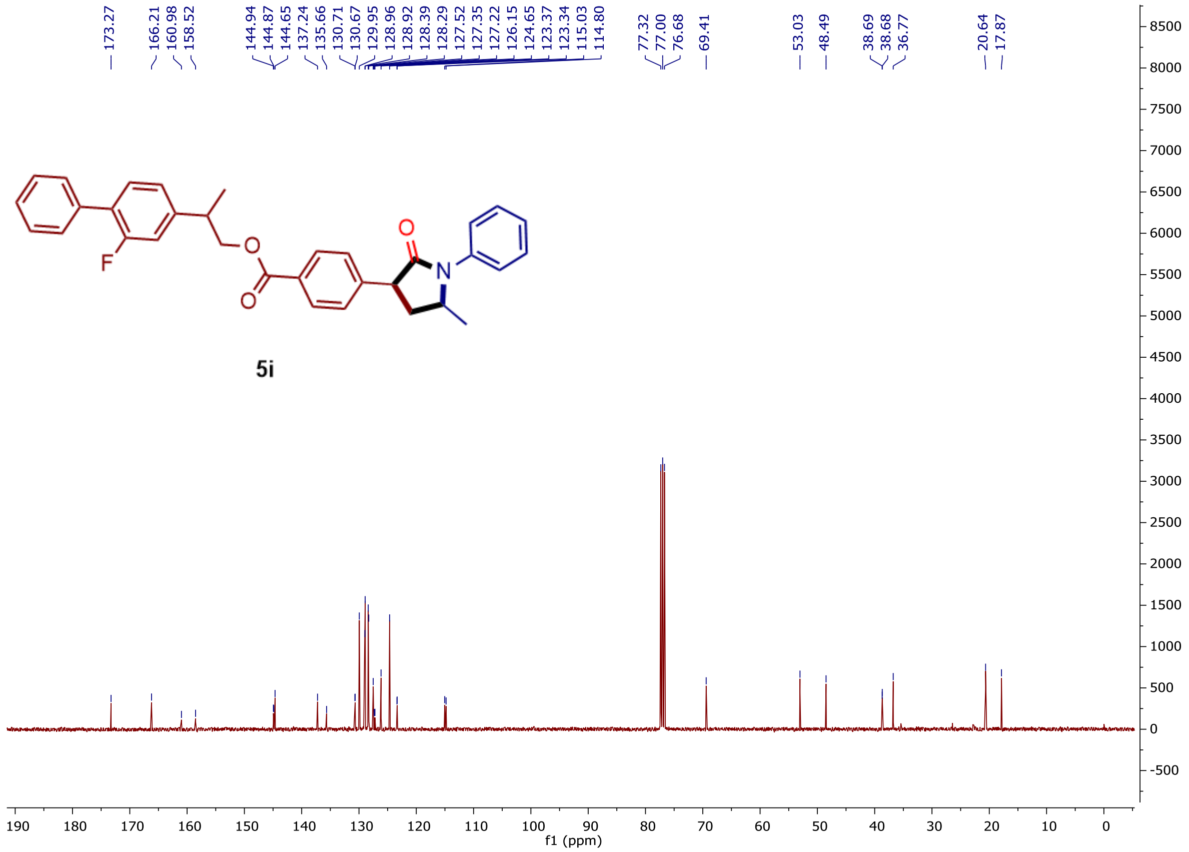
173.27
166.21
160.98
158.52
144.94
144.87
144.65
137.24
135.66
130.71
130.67
129.95
128.96
128.92
128.39
128.29
127.52
127.35
127.22
126.15
124.65
123.37
123.34
115.03
114.80
77.32
77.00
76.68
69.41
53.03
48.49
38.69
38.68
36.77
20.64
17.87
O
N
O
O
F
5i
f1 (ppm)

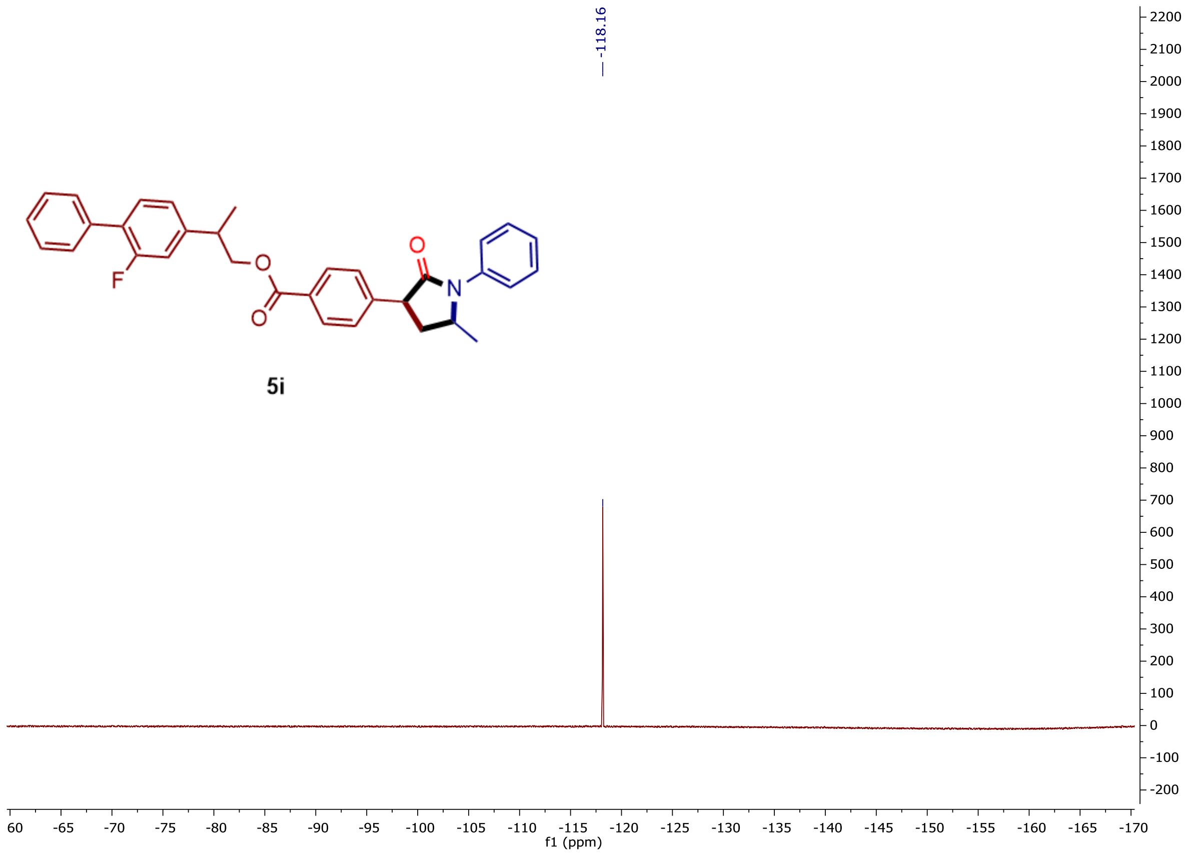
-118.16
O
N
O
O
F
5i
f1 (ppm)

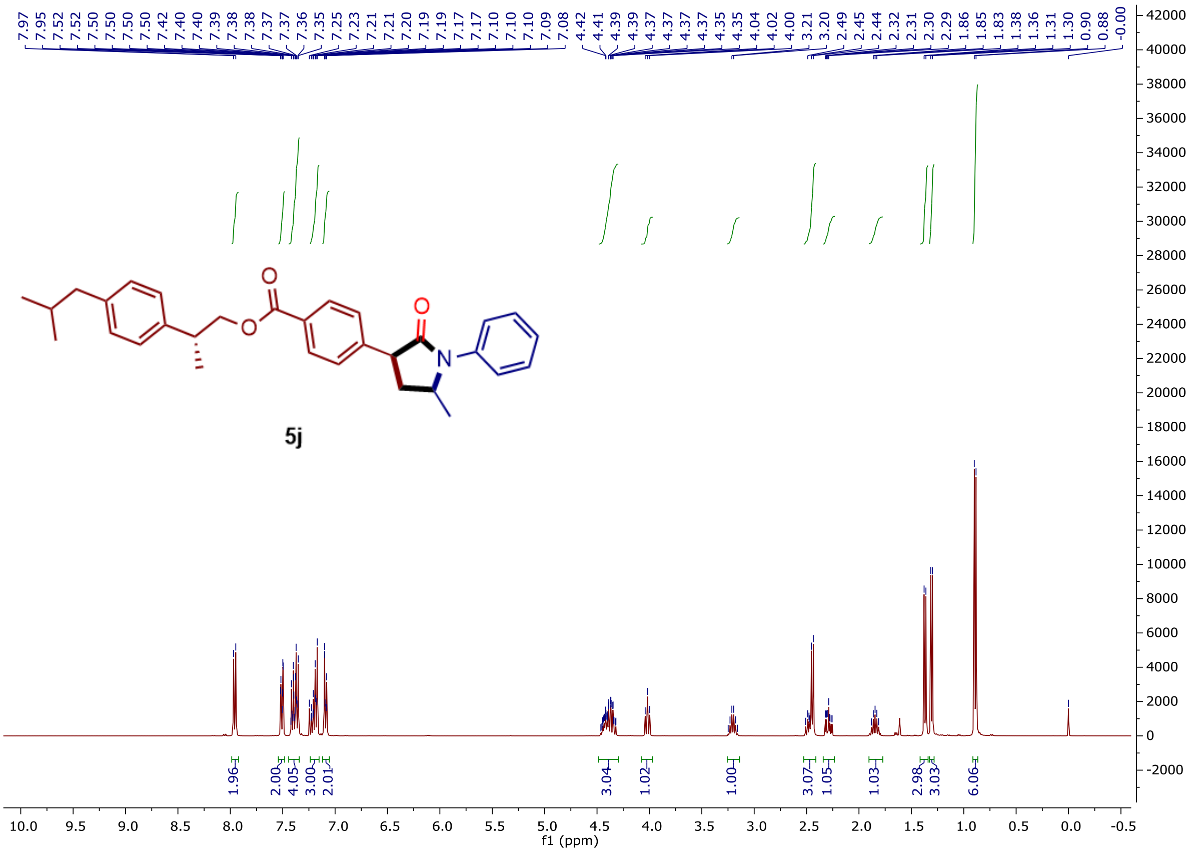
5j
f1 (ppm)

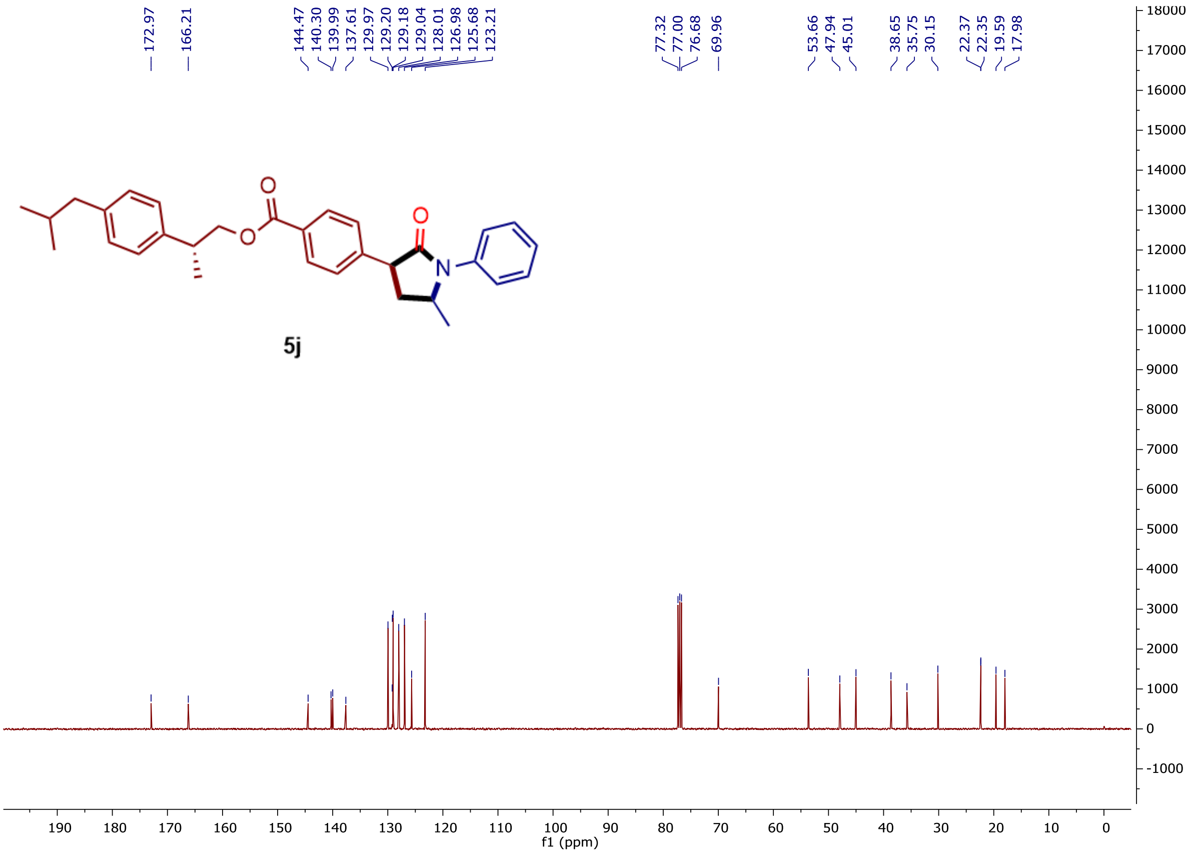
5j
f1 (ppm)

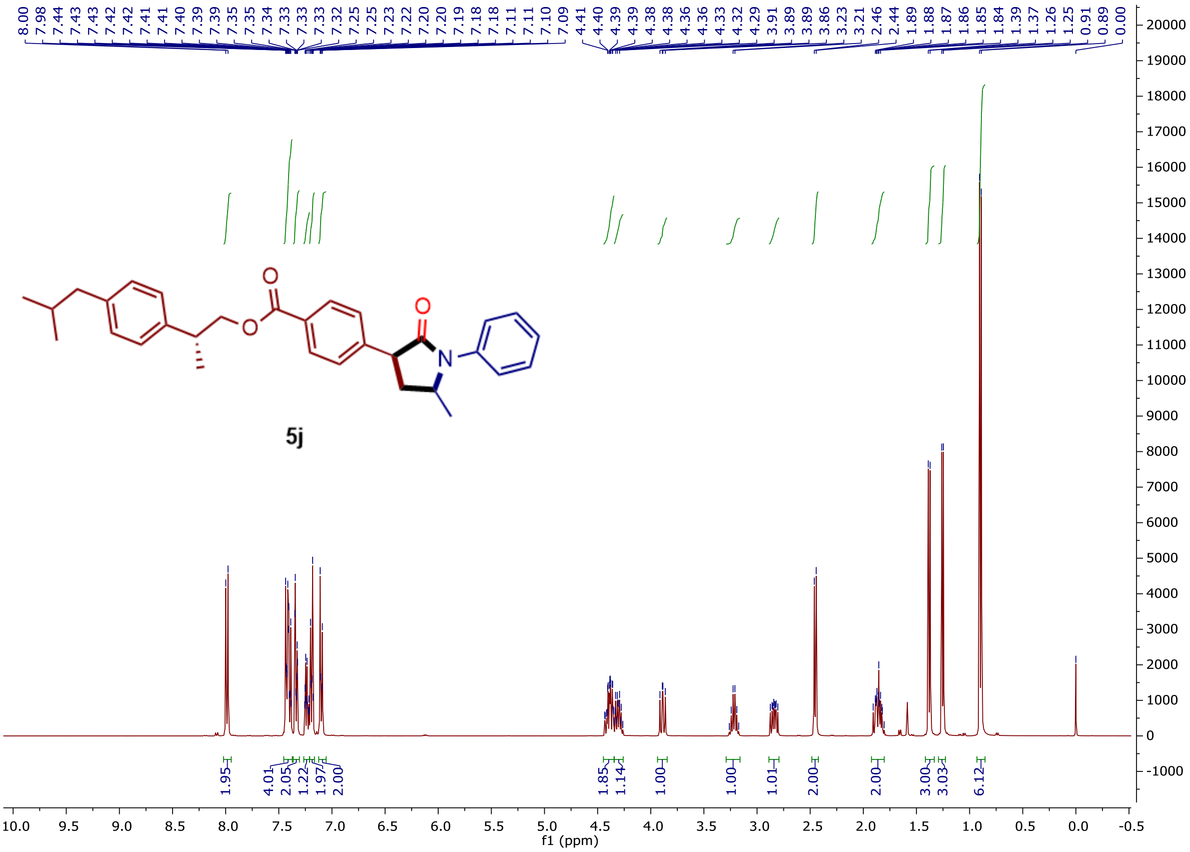
8.00 7.98 7.44 7.43 7.43 7.42 7.42 7.41 7.41 7.40 7.39 7.39 7.35 7.35 7.34 7.33 7.33 7.33 7.32 7.25 7.25 7.23 7.22 7.20 7.20 7.19 7.18 7.11 7.11 7.10 7.09
4.41 4.40 4.39 4.39 4.38 4.38 4.36 4.36 4.33 4.32 4.29 3.91 3.89 3.89 3.86 3.23 3.21 2.46 2.44 1.89 1.88 1.87 1.86 1.85 1.84 1.39 1.37 1.26 1.25 0.91 0.89 0.00
O
N
5j
1.95 4.01 2.05 1.22 1.97 2.00 1.85 1.14 1.00 1.00 1.01 2.00 2.00 3.00 3.03 6.12
f1 (ppm)

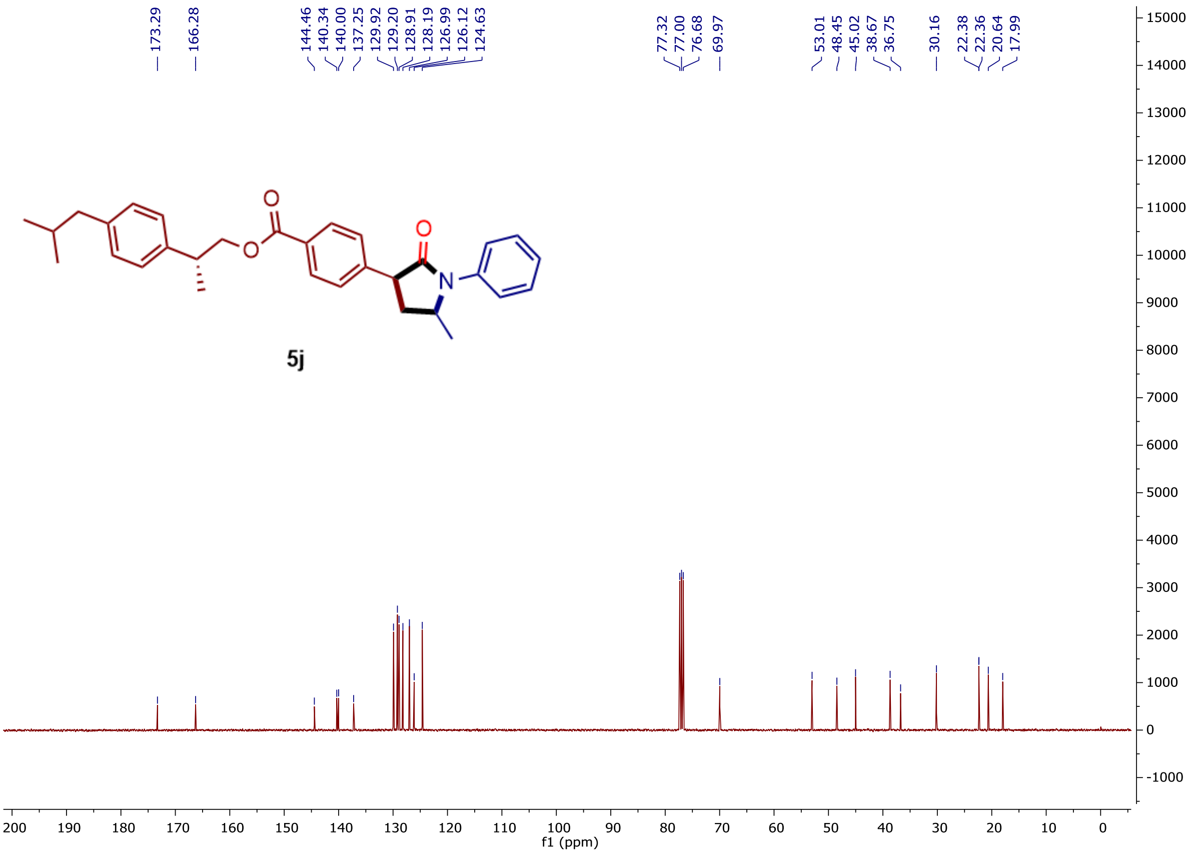
173.29 166.28 144.46 140.34 140.00 137.25 129.92 129.20 128.91 128.19 126.99 126.12 124.63
77.32 77.00 76.68 69.97 53.01 48.45 45.02 38.67 36.75 30.16 22.38 22.36 20.64 17.99
O
N
5j
f1 (ppm)

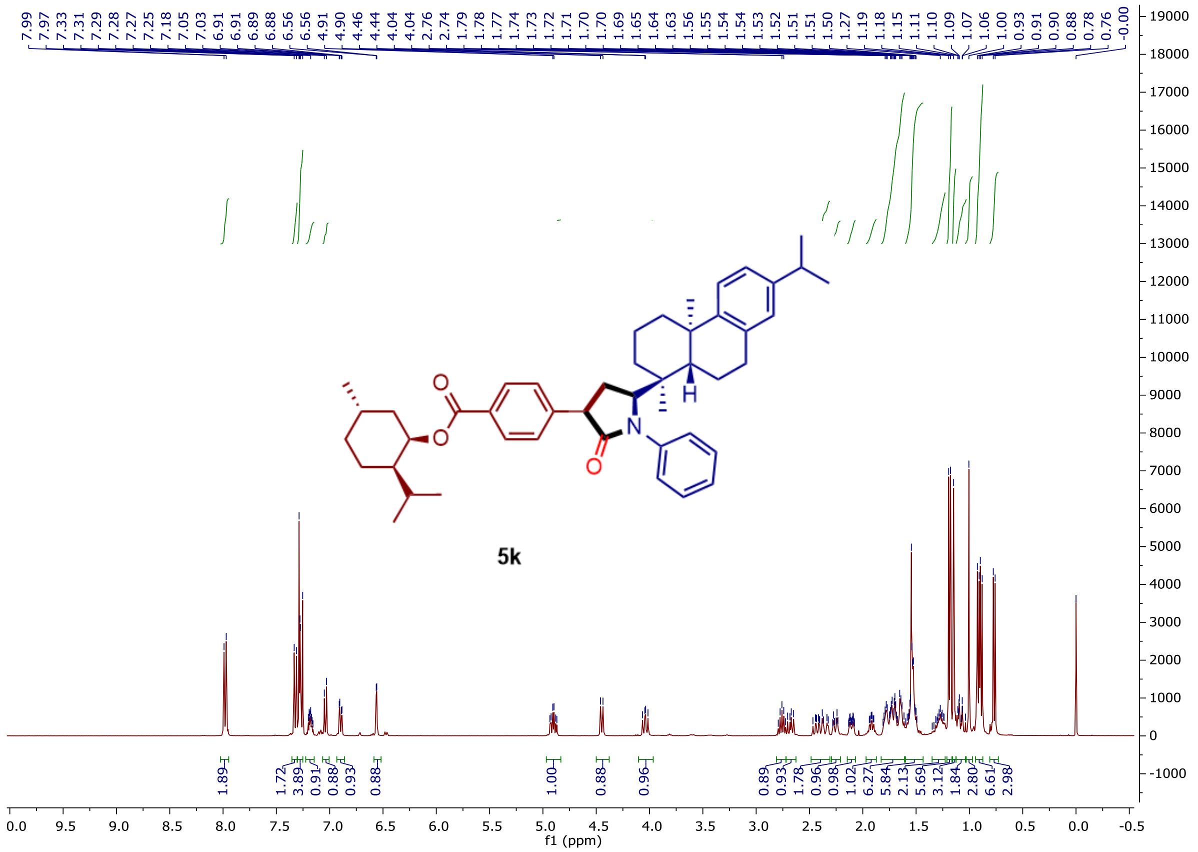
7.99 7.97 7.33 7.31 7.29 7.28 7.27 7.25 7.18 7.05 7.03 6.91 6.91 6.89 6.88 6.56 6.56 4.91 4.90 4.46 4.44 4.04 4.04 2.76 2.74 1.79 1.78 1.77 1.74 1.73 1.72 1.71 1.70 1.69 1.65 1.64 1.63 1.56 1.55 1.54 1.54 1.53 1.52 1.51 1.51 1.50 1.27 1.19 1.18 1.15 1.11 1.10 1.09 1.07 1.06 1.00 0.93 0.91 0.90 0.88 0.78 0.76 -0.00
5k
1.89 1.72 3.89 0.91 0.88 0.93 0.88 1.00 0.88 0.96 0.89 0.93 1.78 0.96 0.98 1.02 6.27 5.84 2.13 5.69 3.12 1.84 2.80 6.61 2.98
f1 (ppm)

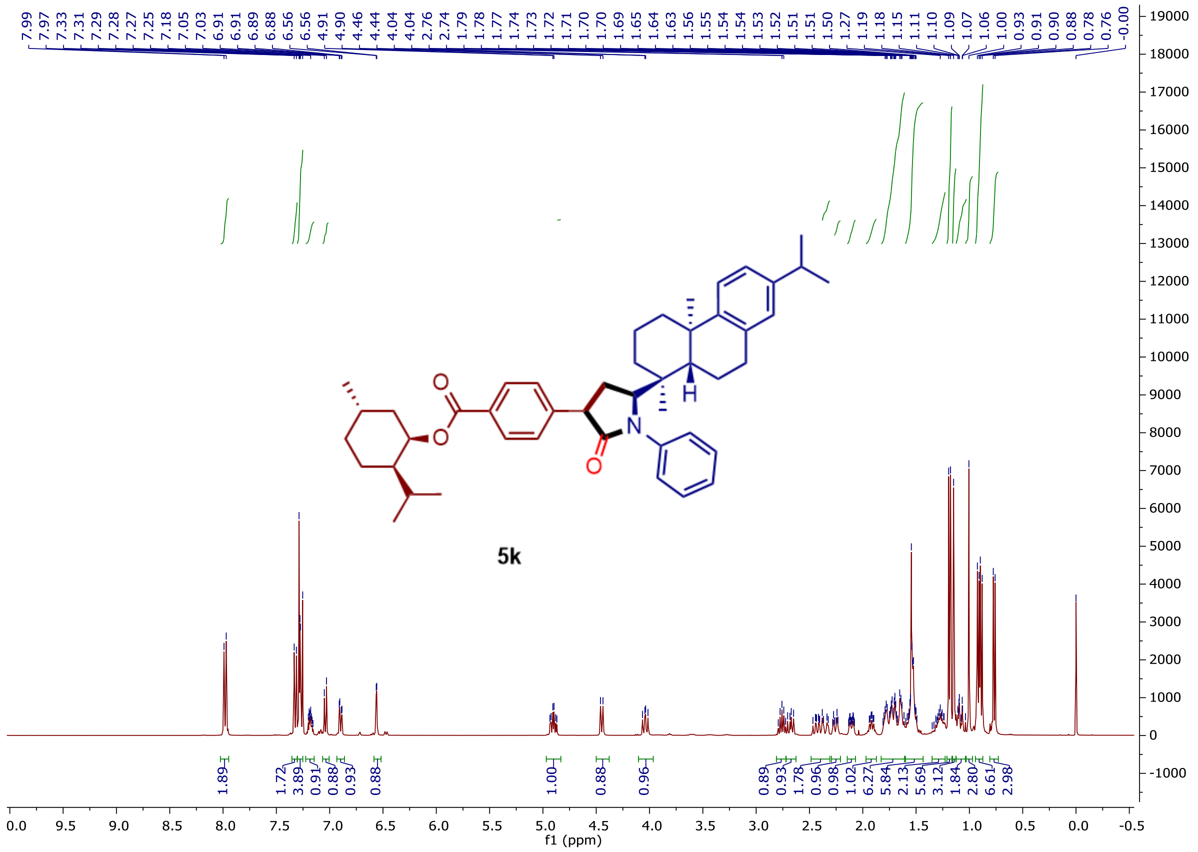
7.99 7.97 7.33 7.31 7.29 7.28 7.27 7.25 7.18 7.05 7.03 6.91 6.91 6.89 6.88 6.56 6.56 4.91 4.90 4.46 4.44 4.04 4.04 2.76 2.74 1.79 1.78 1.77 1.74 1.73 1.72 1.71 1.70 1.69 1.65 1.64 1.63 1.56 1.55 1.54 1.54 1.53 1.52 1.51 1.51 1.50 1.27 1.19 1.18 1.15 1.11 1.10 1.09 1.07 1.06 1.00 0.93 0.91 0.90 0.88 0.78 0.76 -0.00
5k
1.89 1.72 3.89 0.91 0.88 0.93 0.88 1.00 0.88 0.96 0.89 0.93 1.78 0.96 0.98 1.02 6.27 5.84 2.13 5.69 3.12 1.84 2.80 6.61 2.98
f1 (ppm)

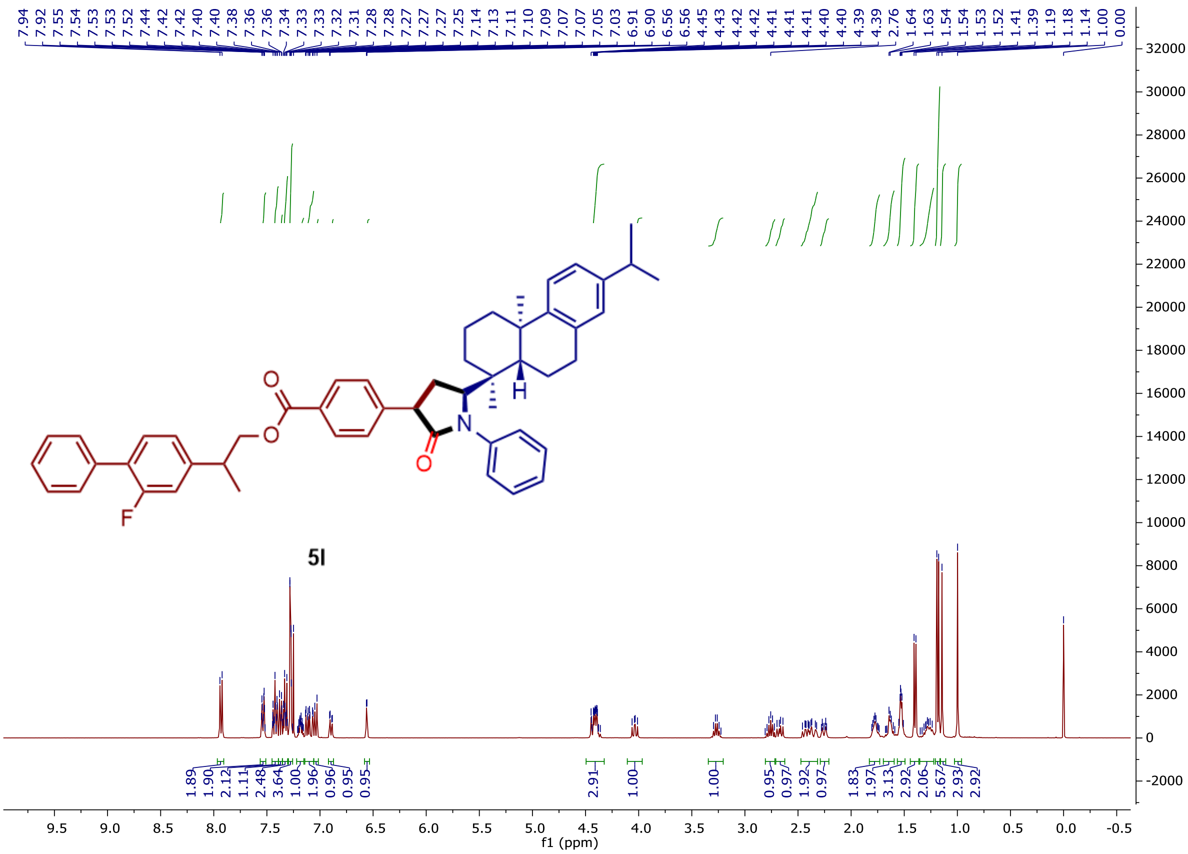

5l

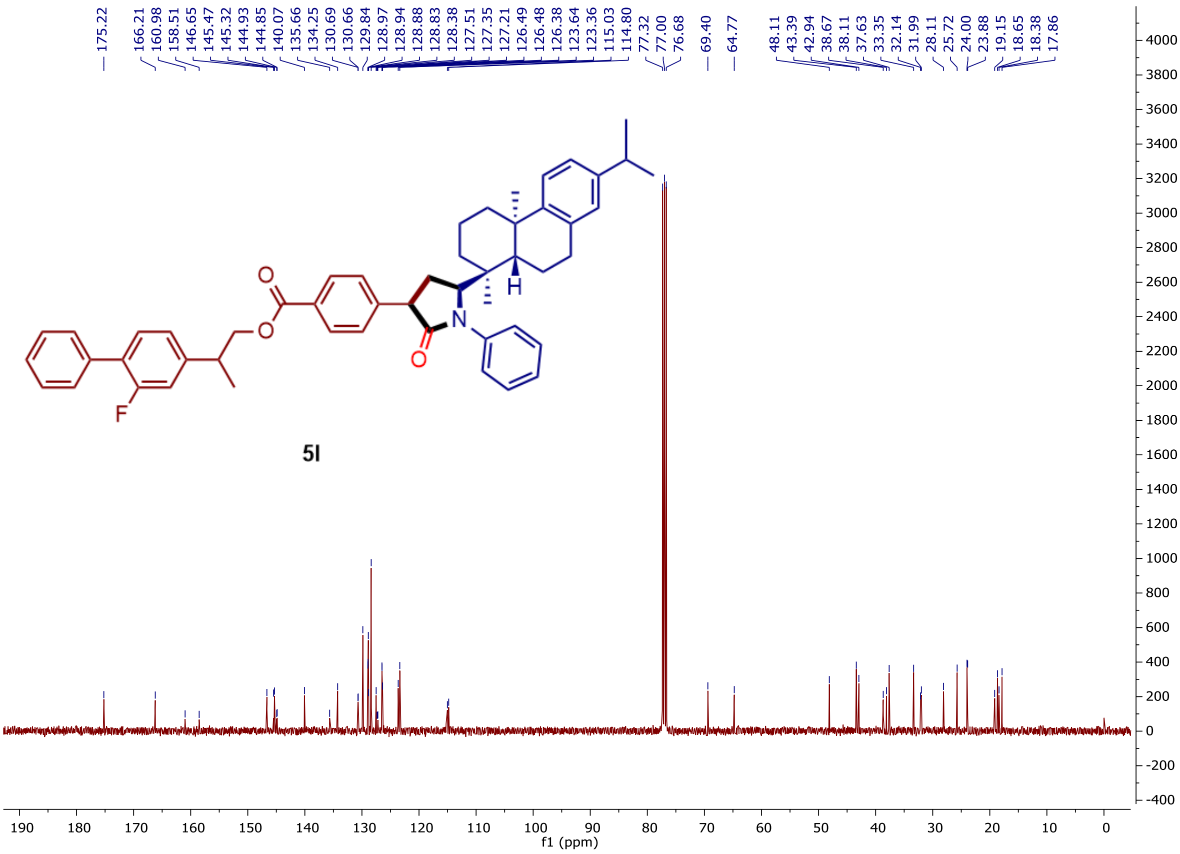

5l

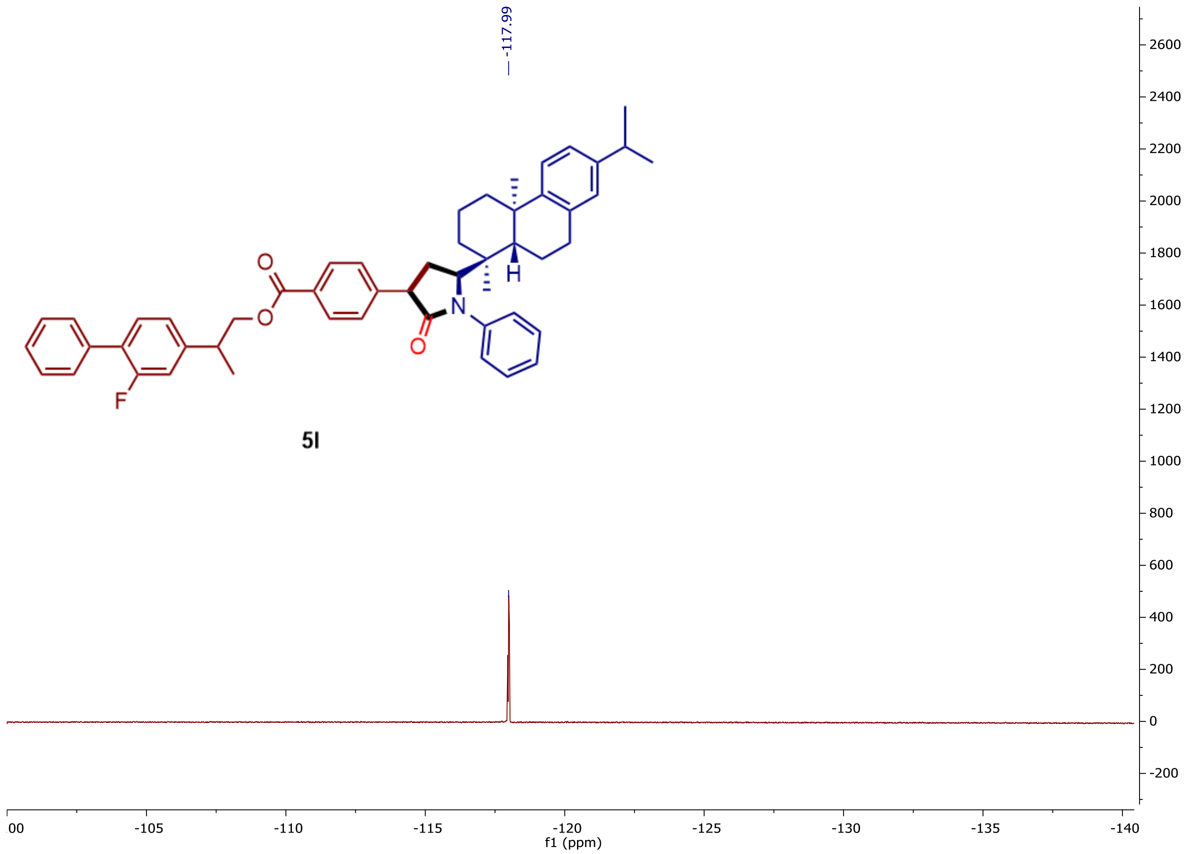
-117.99
5l
F
O
N
H
f1 (ppm)

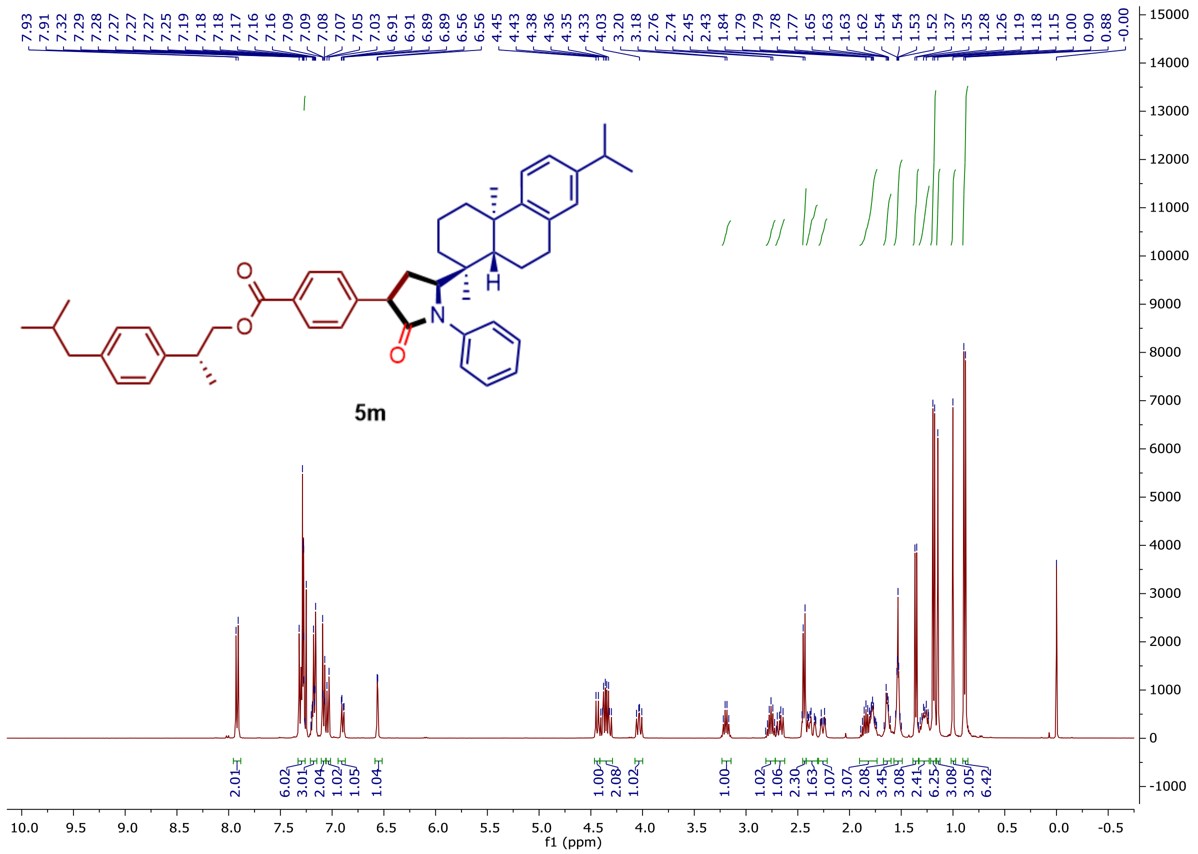
7.93 7.91 7.32 7.29 7.28 7.27 7.27 7.25 7.19 7.18 7.18 7.17 7.16 7.16 7.09 7.08 7.07 7.05 7.03 6.91 6.91 6.89 6.89 6.56 4.45 4.43 4.38 4.36 4.35 4.33 4.03 3.20 3.18 2.76 2.74 2.45 2.43 1.84 1.79 1.78 1.77 1.65 1.63 1.63 1.62 1.54 1.54 1.53 1.52 1.37 1.35 1.28 1.26 1.19 1.18 1.15 1.00 0.90 0.88 -0.00
5m
O
N
H
2.01 6.02 3.01 2.04 1.02 1.05 1.04 1.00 2.08 1.02 1.00 1.02 1.06 2.30 1.63 1.07 3.07 2.08 3.45 3.08 2.41 6.25 3.08 3.05 6.42
f1 (ppm)

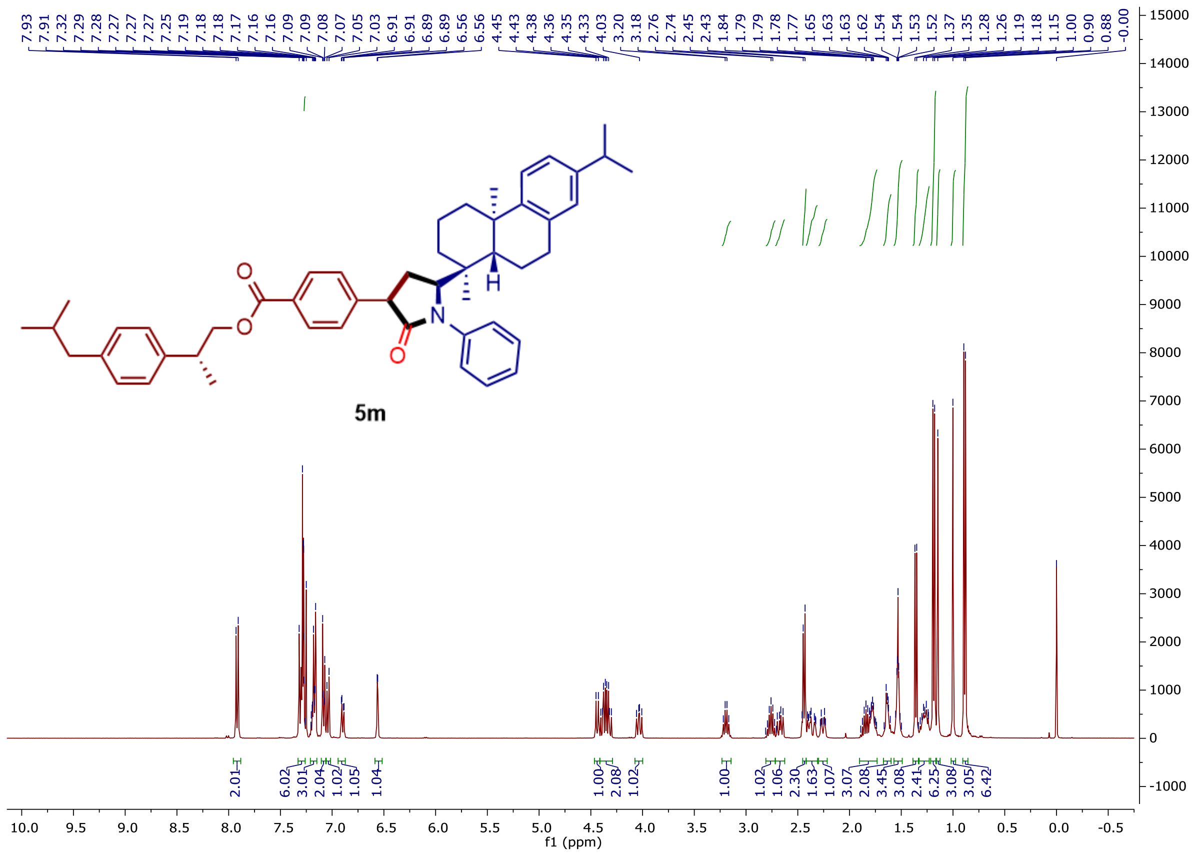

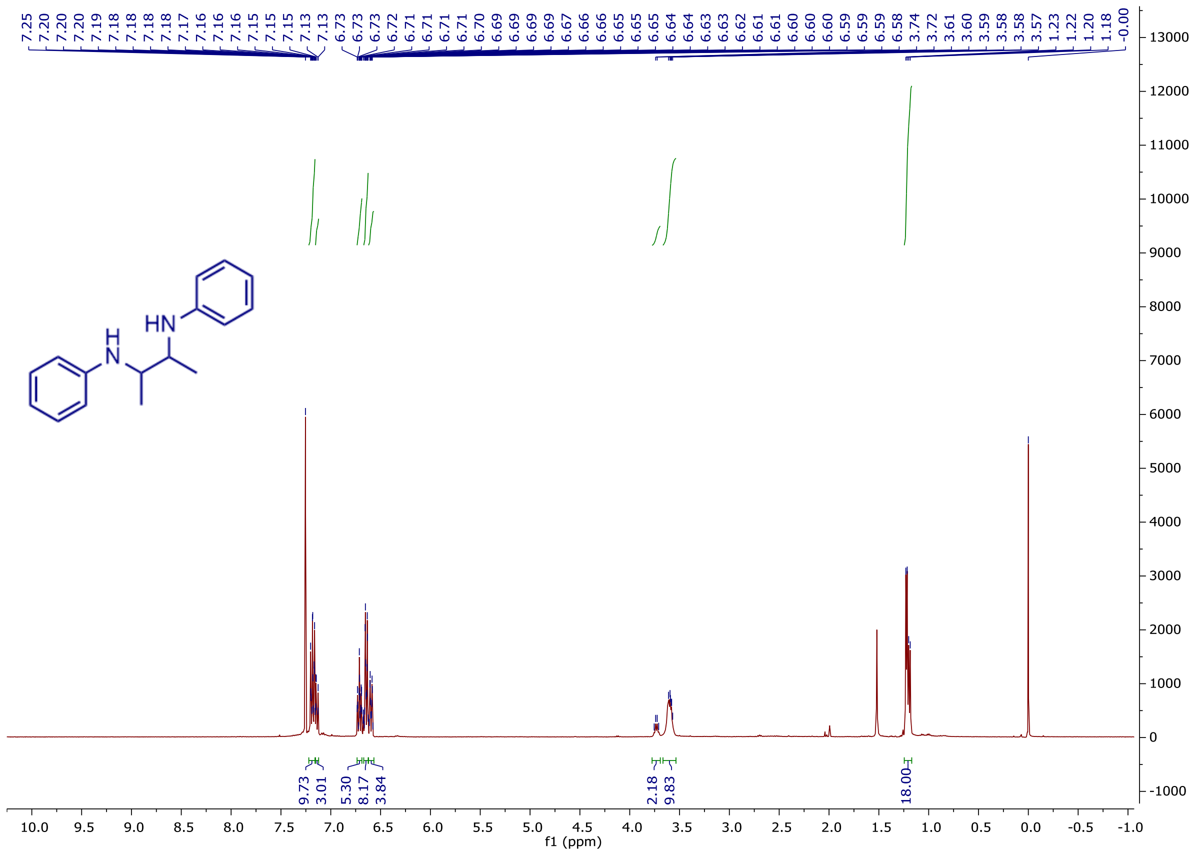

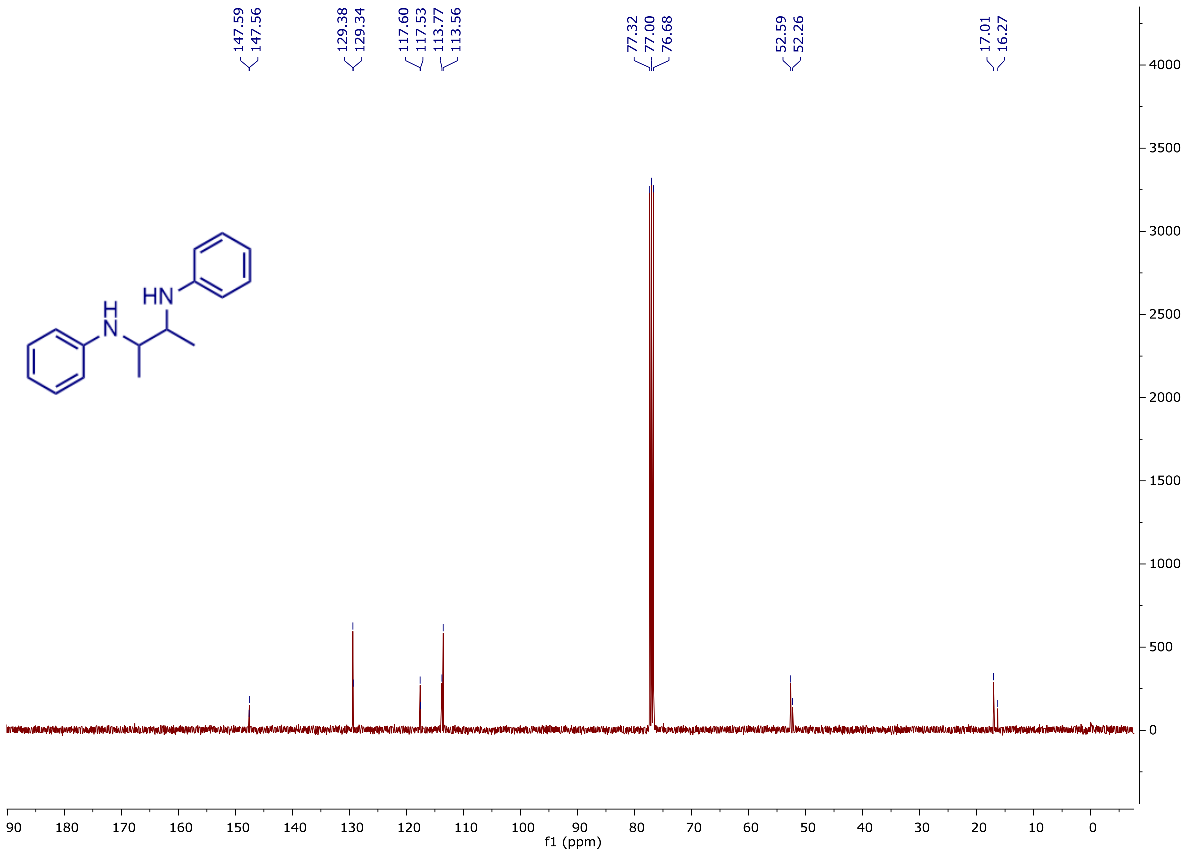
147.59
147.56
129.38
129.34
117.60
117.53
113.77
113.56
77.32
77.00
76.68
52.59
52.26
17.01
16.27
HN
H
N
f1 (ppm)

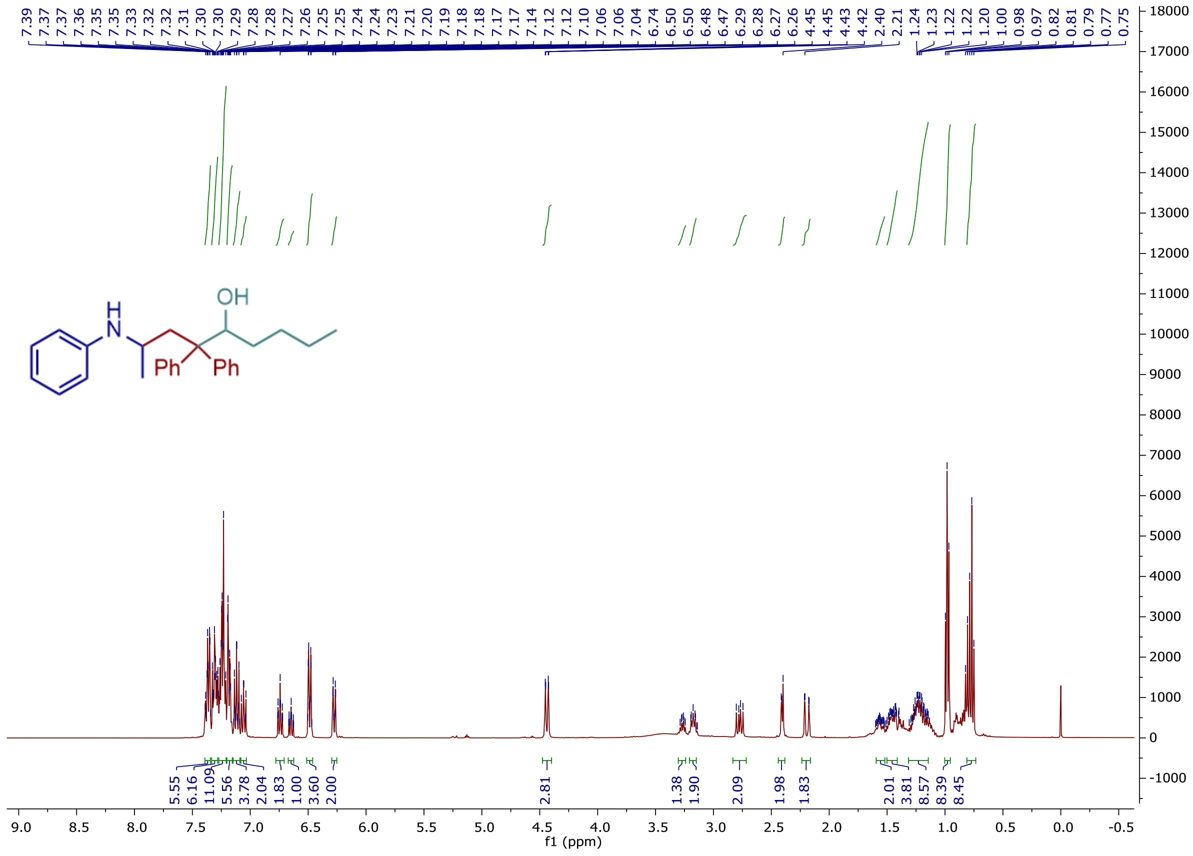
7.39 7.37 7.37 7.36 7.35 7.35 7.33 7.32 7.32 7.31 7.30 7.30 7.29 7.28 7.28 7.27 7.26 7.25 7.25 7.24 7.24 7.23 7.21 7.20 7.19 7.18 7.18 7.17 7.17 7.14 7.12 7.12 7.10 7.06 7.04 6.74 6.50 6.50 6.48 6.47 6.29 6.28 6.27 6.26 4.45 4.45 4.43 4.42 2.40 2.21 1.24 1.23 1.22 1.22 1.20 1.00 0.98 0.97 0.82 0.81 0.79 0.77 0.75
H
N
OH
Ph
Ph
5.55
6.16
11.09
5.56
3.78
2.04
1.83
1.00
3.60
2.00
2.81
1.38
1.90
2.09
1.98
1.83
2.01
3.81
8.57
8.39
8.45
f1 (ppm)

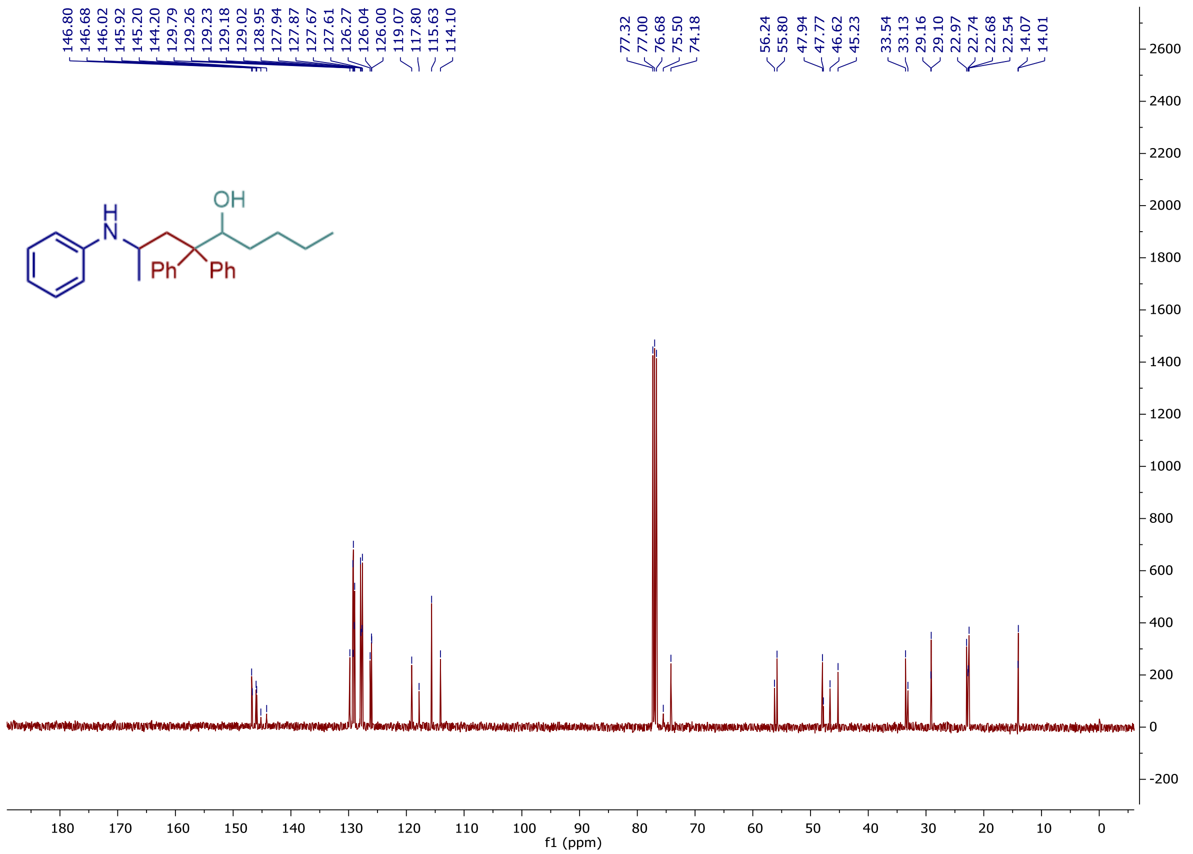

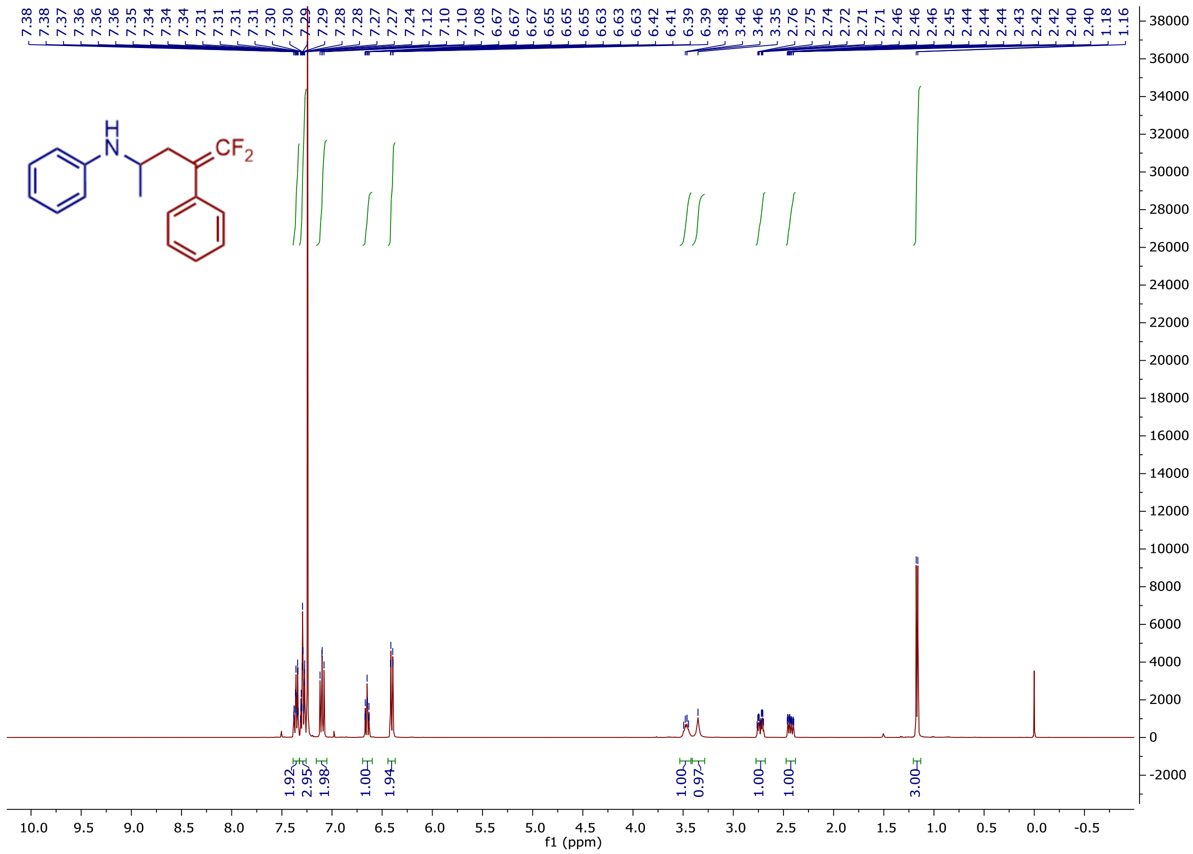

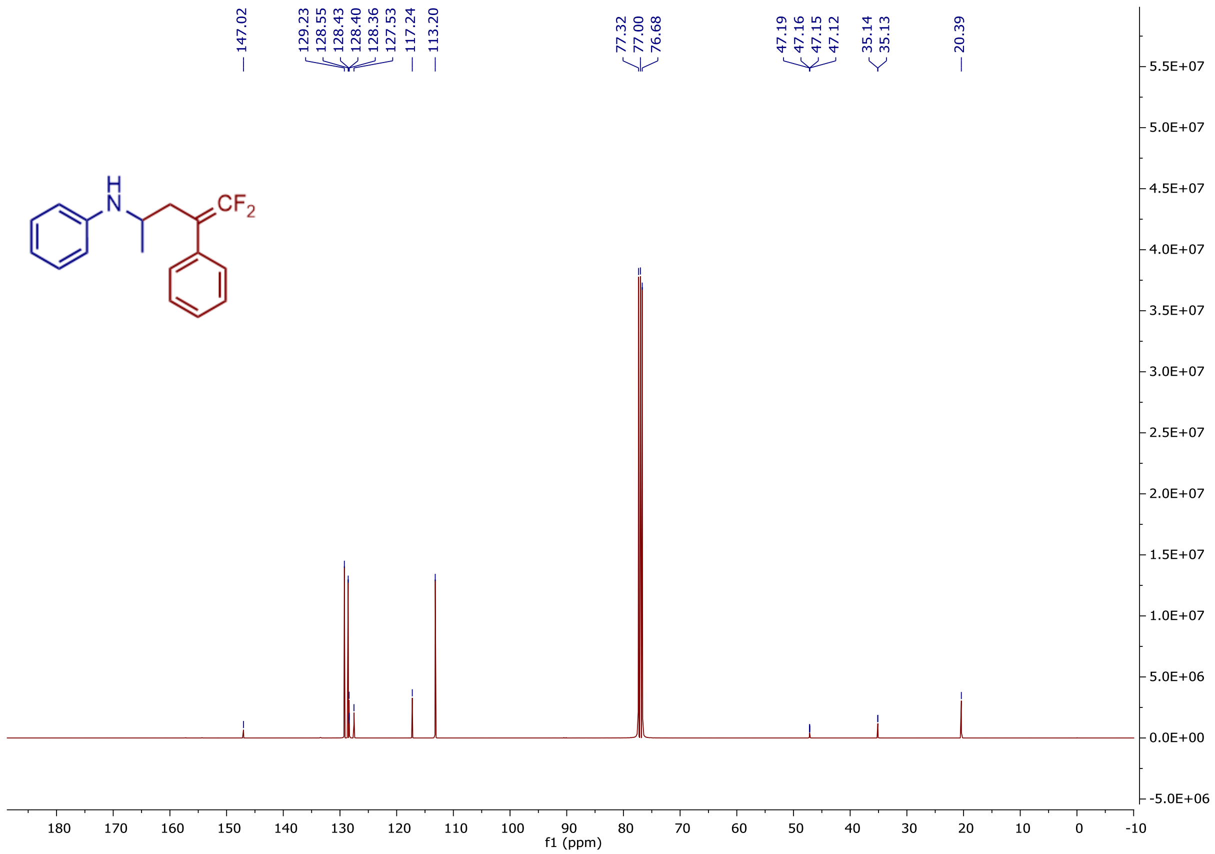
147.02
129.23
128.55
128.43
128.40
128.36
127.53
117.24
113.20
77.32
77.00
76.68
47.19
47.16
47.15
47.12
35.14
35.13
20.39
CF2
H
N
f1 (ppm)

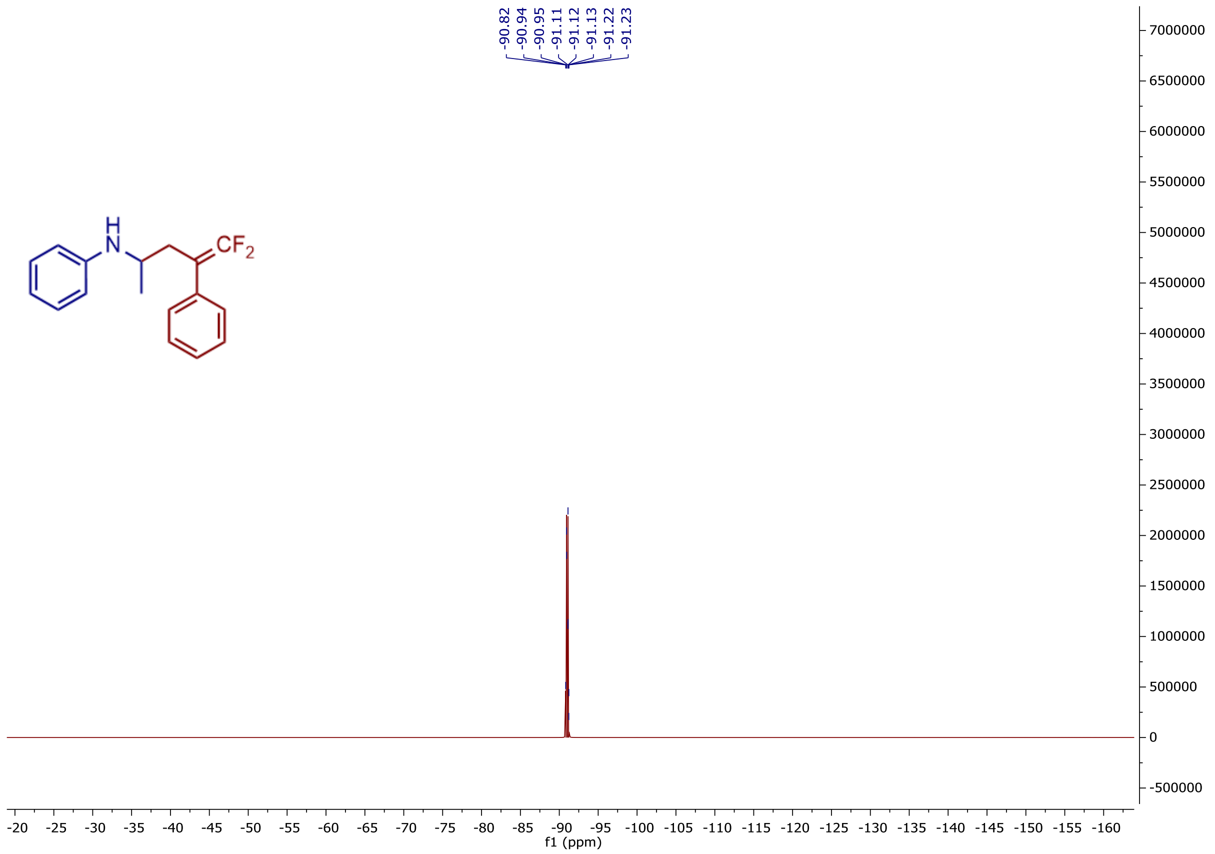
-90.82
-90.94
-90.95
-91.11
-91.12
-91.13
-91.22
-91.23
CF2
H
N
f1 (ppm)
